# Sauropodomorph evolution across the Triassic–Jurassic boundary: body size, locomotion, and their influence on morphological disparity

**DOI:** 10.1038/s41598-021-01120-w

**Published:** 2021-11-18

**Authors:** Cecilia Apaldetti, Diego Pol, Martín D. Ezcurra, Ricardo N. Martínez

**Affiliations:** 1 CONICET-IMCN, Instituto Y Museo de Ciencias Naturales-CIGEOBIO, Universidad Nacional de San Juan, Av. España 400 Norte, 5400 San Juan, Argentina; 2 CONICET-MEF, Museo Paleontológico Egidio Feruglio, Av. Fontana 140, Trelew, Chubut Argentina; 3 CONICET-MACN, Sección Paleontología de Vertebrados, CONICET–Museo Argentino de Ciencias Naturales “Bernardino Rivadavia”, Av. Ángel Gallardo 470, C1405DJR Ciudad Autónoma de Buenos Aires, Argentina; 4 Instituto Y Museo de Ciencias Naturales (IMCN)-CIGEOBIO, Universidad Nacional de San Juan, Av. España 400 Norte, 5400 San Juan, Argentina

**Keywords:** Palaeontology, Phylogenetics, Planetary science

## Abstract

Sauropodomorph dinosaurs were the dominant medium to large-sized herbivores of most Mesozoic continental ecosystems, being characterized by their long necks and reaching a size unparalleled by other terrestrial animals (> 60 tonnes). Our study of morphological disparity across the entire skeleton shows that during the Late Triassic the oldest known sauropodomorphs occupied a small region of morphospace, subsequently diversifying both taxonomically and ecologically, and shifting to a different and broader region of the morphospace. After the Triassic–Jurassic boundary event, there are no substancial changes in sauropodomorph morphospace occupation. Almost all Jurassic sauropodomorph clades stem from ghost lineages that cross the Triassic–Jurassic boundary, indicating that variations after the extinction were more related to changes of pre-existing lineages (massospondylids, non-gravisaurian sauropodiforms) rather than the emergence of distinct clades or body plans. Modifications in the locomotion (bipedal to quadrupedal) and the successive increase in body mass seem to be the main attributes driving sauropodomorph morphospace distribution during the Late Triassic and earliest Jurassic. The extinction of all non-sauropod sauropodomorphs by the Toarcian and the subsequent diversification of gravisaurian sauropods represent a second expansion of the sauropodomorph morphospace, representing the onset of the flourishing of these megaherbivores that subsequently dominated in Middle and Late Jurassic terrestrial assemblages.

The diversification and radiation of dinosaurs in the early Mesozoic (Late Triassic–Early Jurassic, ~ 233–174 million years ago [Ma]) was one of the major biological events in the evolution of terrestrial vertebrates^1–4^. During the Late Triassic, dinosaurs and pseudosuchians exploited similar resources and shared ecological roles in continental ecosystems^5–7^. However, at the end-Triassic occurred a catastrophic event recognized as one of the ‘big five’ Phanerozoic mass extinctions^8,9^, known as the end-Triassic extinction event (ETE). Causal hypotheses of this event range from gradualistic (sea-level fluctuations, widespread aridification) to catastrophic processes (*e.g.*, bolid impact, volcanism)^9–13^. After the Triassic–Jurassic boundary (TJB) all non-crocodylomorph pseudosuchians and non-dinosaurian dinosauromorphs went extinct, whereas most pre-existing dinosaur lineages survived, rapidly diversified, and prevailed during the subsequent 135 Myr of the Mesozoic Era^1,2,4,7,8,14^. The precise pattern of change in terrestrial ecosystems across this boundary is still poorly understood, and current data suggests it was a result of a complex combination of events at different geographical scales, rather than a single mass extinction event^3,15,16^.

Sauropodomorpha was the most diverse and abundant dinosaur clade at the time of the TJB, as this clade was the first dinosaur group that radiated and became both broadly distributed worldwide and dominant in Late Triassic continental ecosystems^17,18^. Early sauropodomorphs first appeared during the Carnian (~ 233–231 Ma) and later diversified to become the most abundant component of dinosaur faunas during approximately 30 million years (Myr) (Norian–Pliensbachian; ca. 220–190 Myr^17,19^. These diverse early sauropodomorph lineages were replaced at the end of the Early Jurassic (Toarcian, ~ 180 Ma) by Gravisauria^20^, a clade that includes giant quadrupedal sauropods that were the largest land vertebrates to inhabit the Earth^18,21–26^. The known taxonomic and morphological diversity of early sauropodomorphs has dramatically increased in recent years^27–33^, providing critical new information on their evolution during a long period of ecological dominance (Norian–Pliensbachian). Research efforts during the last decade yielded certain consensus on the phylogenetic hypotheses of this group^27–37^, but global ecomorphological aspects have been explored only recently ^38–40^. Here, we evaluate quantitatively the evolutionary history of Sauropodomorpha through a comprehensive morphological disparity analysis and regression-based phylogenetic comparative methods of the entire skeleton that allows us characterizing certain aspects of the macroevolution of the group during the Late Triassic and Early Jurassic.

## Results

**Figure 1:**
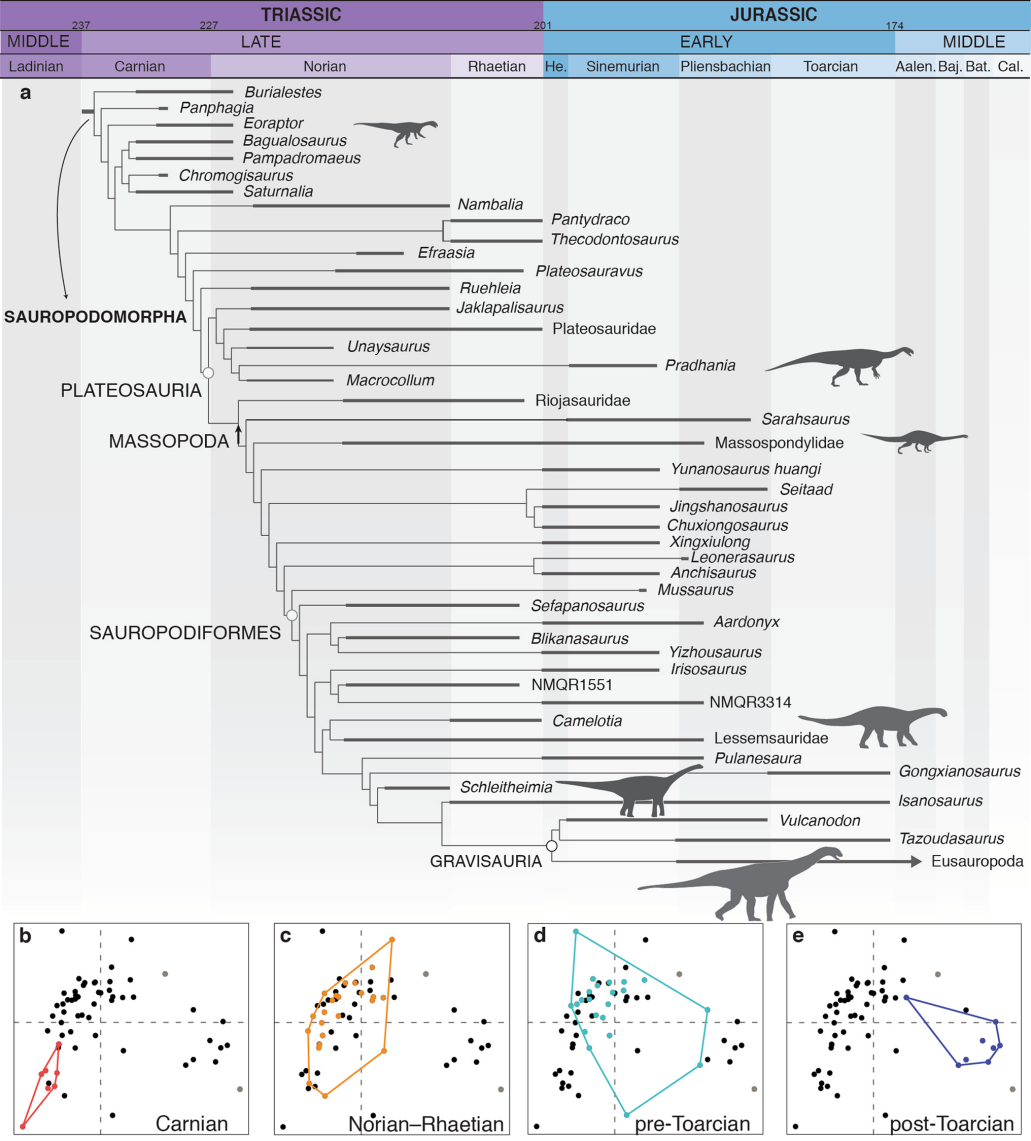
Morphospaces and diversity of Sauropodomorpha during the Triassic-Early Jurassic. (**a**) Randomly selected, time calibrated MPT and morphospace occupation of Sauropodomorpha during (**b**) Carnian, (**c**) Norian-Rhaetian, (**d**) pre-Toarcian, and (**e**) post-Toarcian periods. Each plot shows the first two principal coordinate axes, with a variance of 9.82% (PCo 1, x axis) and 3.90% (PCo 2, y axis) (see Fig. 2b for more details about morphospace plots).

**Figure 2:**
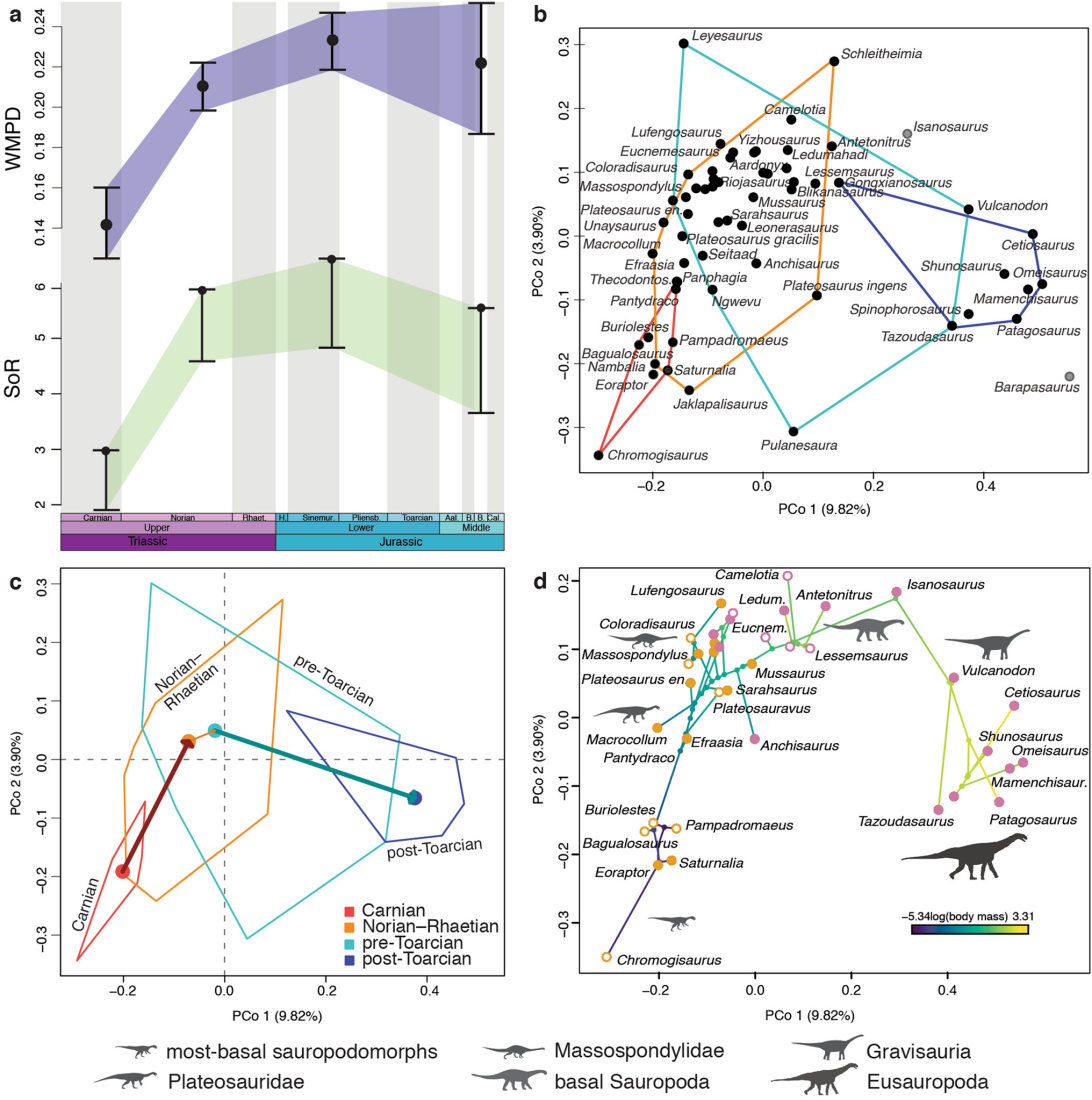
Morphological disparity of Sauropodomorpha during the Triassic-Early Jurassic. (**a**) Weighted mean pairwaise dissimilarity (WMPD) and Sum of Ranges (SoR) with their respective 95% confidence intervals. (**b**) Morphospace distribution in the first two PCOs. *Barapasaurus* and *Isanosaurus* (greys circles) were excluded from time bin groups because their temporal uncertainty. (**c**) Displacement from the centroid of the previous time bin, in which the thickness of the arrows is proportional to the multidimensional distance of the displacement. (**d**) Phylomorphospace showing locomotion style (mapped in orange circles for bipedal and pink circles for quadrupedal) and body mass optimized through time (colours of branches). Empty circles represent assumed locomotion style based on Fitch optimization in the phylogenetic trees.

Carnian sauropodomorphs had an initial diversification restricted to a limited and well-differentiated region of the morphospace, representing the small, bipedal and highly plesiomorphic sauropodomorph body plan (Fig. 1a,b). Our results (significant differences between disparity metrics were calculated after determining the non-overlapping of 95% confidence intervals generated from 9,999 bootstrap replicates; see “Methods”) show an increase in morphospace size and density from the origin of the group (Carnian) towards the Jurassic (Figs. 1, 2). This is shown by the increase in weighted mean pairwise dissimilarity (WMPD, a pre-ordination metric that captures mainly morphospace density) as well as in the Sum of Ranges (SoR) and Sum of Variances (SoV) (post-ordination metrics that captures morphospace size and density, Table 1; see “Methods”).

**Table 1:** Selected results of the morphological disparity analyses (all other results are reported in the supplementary information). The upper and lower limits were calculated after building 95% confidence intervals calculated from 9999 bootstrap replicates of the distance (WMPD) and ordinated matrices (other measures). Abbreviations: MJ, Middle Jurassic.

Bin	WMPD	Lower limit	Upper limit
**WMPD all taxa**			
Carnian	0.1419317	0.1246697	0.1591733
Norian–Rhaetian	0.2143386	0.2021589	0.2257176
Pre-Toarcian	0.2494814	0.2339706	0.2639456
Toarcian–MJ	0.2188413	0.1898161	0.2449658
**WMPD locomotion**			
Bipeds	0.2231127	0.2143411	0.2320984
Quadrupeds	0.3280687	0.3117793	0.3442396

Bin	SoV	Lower limit	Upper limit
**SoV all taxa**			
Carnian	0.1057576	0.09002641	0.1590102
Norian–Rhaetian	0.1580963	0.12401888	0.2046489
Pre-Toarcian	0.1831581	0.14149178	0.2421437
Toarcian–MJ	0.2246976	0.21185220	0.2951077
**SoV locomotion**			
Bipeds	0.1356129	0.1088801	0.1708456
Quadrupeds	0.2089300	0.1845635	0.2493610

Bin	SoR	Lower limit	Upper limit
**SoR all taxa**			
Carnian	3.658997	2.220012	3.658997
Norian–Rhaetian	6.750165	5.186897	6.750165
Pre-Toarcian	7.360617	5.563492	7.360617
Toarcian–MJ	6.327877	4.421535	6.327877
**SoR locomotion**			
Bipeds	6.760765	4.988128	6.760765
Quadrupeds	7.959643	6.582425	7.959643

Bin	Displacement	Lower limit	Upper limit
**Displacement from centroid all taxa**			
Carnian	1.518088	1.452325	2.312874
Norian–Rhaetian	1.063757	1.035988	1.162953
Pre-Toarcian	1.045143	1.016389	1.130591
Toarcian–MJ	1.364435	1.279873	1.751107
**Displacement from previous time bin**			
Carnian	1.000000	1.000000	1.000000
Norian–Rhaetian	1.425361	1.337646	1.581720
Pre-Toarcian	1.092128	1.058417	1.189740
Toarcian–MJ	1.376450	1.292433	1.774152
**Displacement from centroid of locomotion styles**			
Bipeds	1.115236	1.082434	1.206832
Quadrupeds	1.018242	1.010159	1.074852

The morphological disparity (WMPD) and the amount of morphospace occupied (SoR) significantly increase by the Norian–Rhaetian (Figs. 1a,c, 2a,b; Table 1), documenting the main diversification of early sauropodomorphs. Most early sauropodomorph lineages (plateosaurids, riojasaurids, massospondylids, sauropodiforms) appeared between the mid-Norian (~ 215 Ma) and the end-Triassic (~ 201 Ma)^33,34,38,41^. This large diversification of sauropodomorphs and the associated anatomical novelties position these forms in a previously unexplored region of the morphospace. This position shift is documented by significant results in the displacements from the centroid, which is a post-ordination metric that captures differences in morphospace position (Fig. 2c; Table 1), and a permutational multivariate analysis of variance (*p* < 0.01 in a PERMANOVA) between the first two analysed time bins. Extremes of the different body shapes and sizes recorded during the Norian–Rhaetian include the facultatively bipedal early plateosaurians (*i.e*., *Plateosaurus*, *Unaysaurus*)^42^, and the larger, robust, and quadrupedal sauropodiforms (e.g., lessemsaurids)^28,29,37,43,44^. The gracile *Nambalia* and *Jaklapalisaurus* enlarge the region of the morphospace overlapping with some Carnian forms in the first two principal coordinates (i.e., *Eoraptor*, *Saturnalia*), whereas some Rhaetian forms (*Thecodontosaurus*, *Pantydraco*) are closely positioned to *Panphagia* in these same coordinates (Fig. 2b).

Immediately after the TJB the morphological disparity (WMPD) of Sauropodomorpha has a minor increase (which is only marginally significant after the exclusion of taxa that had to be trimmed for the ordination of the dataset; see Methods), coupled with a slight increase of the amount of morphospace occupied (SoR) (Figs. 1a,d, 2a; Table 1). The diversity of the earliest Jurassic sauropodomorphs includes members of most early massopodan clades and a large number of ghost lineages subtending Early Jurassic taxa^28,29,33,38^, which indicates that no major clades originated at this time (Fig. 1). In agreement, the morphospace occupied by Early Jurassic sauropodomorphs largely overlaps that of the latest Triassic forms and there is a minor, marginally significant occupation of new areas of the morphospace during this period (displacements from centroid and p = 0.0117 in a PERMANOVA; Fig. 2c; Table 1). This result is in accordance with previous results based on a subset of the taxa analysed here^38^ and two-dimensional geometric morphometrics of the cranium and pelvic girdle^45^.

Post-Toarcian sauropodomorphs occupy a significantly different area of the morphospace with respect to that of previous time bins (as reflected in the displacements from centroid and p < 0.001 in PERMANOVAs; Table 1). There is also a slight decrease in the morphospace size and density (SoR and SoV) (Figs. 1a,e, 2b,c; Table 1). The new region of the morphospace occupied during the Toarcian–Middle Jurassic (Fig. 2c) represents the emergence of new lineages distinguished by a unique body plan (e.g., Vulcanodontidae, Gravisauria, Eusauropoda) of obligatory quadrupedal stance, elongated forelimbs, and columnar limbs, all features interpreted as related to their large body mass^20,22,38^ (Figs. 1a,e, 2d).

Procrustes-distance-based phylogenetic regressions indicate that these early evolutionary patterns of Sauropodomorpha are strongly influenced by the evolution of body mass (R^2^ = 0.4670 and *p* < 0.05 in all the regressions of the first three PCos versus log(body mass); see Supplementary Information). A wide range of body shapes and sizes are recorded in Sauropodomorpha between the Carnian and the Middle Jurassic, including the gracile and bipedal early forms (*Saturnalia, Eoraptor*, < 50 kg), the facultatively bipedal early plateosaurians (*e*.*g.*, *Efraasia*, *Unaysaurus*; ~ 100–1000 kg), the large and robust quadrupedal sauropodiforms (*e*.*g.*, *Antetonitrus*, *Lessemsaurus, Ledumahadi*; > 5 tn), and the giant gravisaurian sauropods (*e.g.*, *Vulcanodon*, *Bagualia*, *Patagosaurus*; > 10 tn) (Fig. 2d; Supplementary Information). A second variable that also explains a lower, but nonetheless considerable, amount of variance is the present-day continent occurrence of taxa (R^2^ = 0.3746 and *p* < 0.05 in all the regressions of the first three PCos versus biogeography; see Supplementary Information). However, the interaction model between body mass and biogeography performed considerably worse than each variable independently (R^2^ = 0.2103 and *p* < 0.05 for log(body size) and R^2^ = 0.1243 and *p* > 0.05 for biogeography in all the regressions of the first three PCos versus log(body size) + biogeography; see Supplementary Information).

When the dataset was divided into four anatomical partitions (skull + dentition, vertebrae, pectoral girdle + forelimb, and pelvic girdle + hindlimb), a pattern of disparity increase through time (as captured by WMPD) is also recovered for skull + dentition and vertebrae, but the Toarcian–Middle Jurassic time bin was not significantly different from the pre-Toarcian bin (Supplementary Information). Interestingly, these two anatomical partitions have Jurassic WMPD values significantly higher than those of the Triassic, contrasting with the analyses for the whole skeleton that found non-significant changes across the TJB. The pectoral girdle + forelimb dataset has a relatively constant, non-significantly different, disparity (WMPD) from the Carnian to the Pliensbachian, but it drops significantly during the Toarcian–Middle Jurassic (Supplementary Information). A similar pattern is recovered for the pelvic girdle + hindlimb, in which the Toarcian–Middle Jurassic disparity (WMPD) is significantly lower and marginally lower than the pre-Toarcian and Norian–Rhaetian time bins, respectively. The Carnian WMPD value of the pelvic girdle + hindlimb region is significantly lower than that around the TJB, but non-significantly lower than during the Toarcian–Middle Jurassic.

## Discussion and conclusion

At the time of its first appearance in the fossil record (middle–late Carnian), sauropodomorphs were first restricted to a few small species (so far exclusively found in South America and preliminary reported in Africa^33,46^). This early stage in sauropodomorph evolution is characterized by low morphological disparity (Figs. 1, 2a; Table 1). However, small and bipedal taxa with a plesiomorphic body plan were not restricted to the Carnian, as the Norian *Nambalia*^47^ and the Rhaetian *Thecodontosaurus* and *Pantydraco*^48^ share many features and occupy a similar region of the morphospace (Figs. 1, 2b). These Rhaetian taxa are also positioned in a similar region of the morphospace even when only feeding-related features of the jaw apparatus are assessed^40^.

Body size explains approximately half of the variance in our analysis (first three PCos) and, in congruence, the Carnian-Norian shift detected in the morphospace of Sauropodomorpha (Figs. 1a–c, 2c) is consistent with the Norian rapid initial diversification^3,33,38,49^ or early burst pattern inferred in studies of dinosaurian body size evolution^25,50^; sauropodomorph body mass increase from around < 50 kg in Carnian forms to > 5 tonnes in some Norian sauropodiforms^28^. This early shift likely involved an increase in morphological evolutionary rates that allowed the group to diversify, developing different body plans and occupying disparate ecological niches^38,39,50^, after the Carnian–Norian boundary. A significant shift in morphospace between the Carnian and Norian is also congruent with changes in feeding behaviour inferred between the Carnian taxa (e.g., possible faunivorous taxa as *Saturnalia* or *Buriolestes*) and the omnivorous to herbivorous lifestyle adaptations present in later Triassic taxa (e.g., plateosaurians) ^38–41,51^. The wide expansion of the Norian morphospace highlights the main diversification of early sauropodomorphs during mid-Norian and the end-Triassic, when most early linages appeared (plateosaurids, riojasaurids, massospondylids, sauropodiforms) ^33,34,38,41^_._ This great expansion of morphospace of Sauropodomorpha during the Norian probably evidence the occupation of new niches that were exploited by other herbivorous groups previous to the TJB (such as rhynchosaurs, large traversodontid cynodonts, and dicynodonts^1,7,14,38,52,53^).

The major radiation of Sauropodomorpha in the Norian^3,50,54^ is evidenced not only in the increase in their taxonomic diversity and geographic distribution, but also in adaptive changes that goes beyond dietary habits. A marked increase in body size occurs during this stage, coupled with the acquisition of the early sauropodomorph body plan, characterized by small head, elongated neck, three sacral vertebrae, and a modified first manual digit^17,24,33,38,39,55^. All these changes explain the shift and dramatic expansion of their morphological disparity during Norian times (Figs. 1, 2a–c). In parallel to these changes, sauropodomorphs became numerically dominant in terrestrial ecosystems^1,3,19,38,54^ and were not restricted mostly to South America anymore, occurring with several species in Europe, India and Africa^2–4,19,38,47,56^. As a result of this and the subsequent appearance of sauropodomorphs in North America and Asia only after the TJB, our regression analyses find that the biogeographic distribution of species partially matches the morphospace pattern. The anatomical changes that underlie the major disparity excursions of sauropodomorphs in this transition seem to have also strongly influenced disparity changes detected in Dinosauria as a whole^7,14^.

Sauropodomorpha was the most diverse group of terrestrial herbivores across the TJB and our analyses show there were no major changes in their ecomorphology throughout that time (Figs. 1 and 2a). A similar result was also obtained in analyses of morphological disparity and biomechanical properties of sauropodomorph jaws^39,45^_._ Other analyses, based on a subset of the taxa analysed here, recovered only a slight increase or a shift in the morphospace occupation of Sauropodomorpha across the TJB^38,40^. Sauropodomorph diversity has been regarded as decreasing worldwide after the TJB^14,57–60^, but recent discoveries in the Early Jurassic^27,29,31,32^ indicate a higher diversity to levels similar to those of the Late Triassic. Moreover, the greater diversity of sauropodomorphs now known for the upper Elliot Formation of South Africa reflect their abundance and increase in morphological disparity after the TJB^19,38^, suggesting that the mass extinction did not negatively affect Sauropodomorpha^29,37,61^. Indeed, the increase in skull and vertebral disparity from the Late Triassic to the earliest Jurassic in our results indicates there were changes probably related more diverse trophic habits and an increase in the complexity of vertebral lamination and pneumaticity, which are anatomical regions that were already subject to numerous evolutionary changes during the Triassic in sauropodomorphs^28,41,62,63^. Thus, the events across the TJB in sauropodomorph evolution likely were a continuity of evolutionary trends in pre-existing lineages (massospondylids, non-gravisaurian sauropodiforms) rather than representing the emergence of key evolutionary novelties or new, considerably different, body plans. A noteworthy difference across the boundary is the disappearance of small-bodied sauropodomorphs (extended from the earliest sauropodomorphs as *Panphagia* to the latest Triassic forms as *Thecodontosaurus* or *Pantydraco*)^61^.

Finally, a major event in sauropodomorph evolution is the diversification of gravisaurian sauropods, implying numerous changes in their body plan, locomotion, feeding behaviour, and extremely large body size^20,22–24,39,40,55^. This event represents a second expansion of morphospace towards previously unexplored regions (Figs. 1, 2c), and reflects the significant body plan changes of Sauropodomorpha at this time. At the same time, the significantly lower Toarcian–Middle Jurassic disparity values of the pelvic girdle and hindlimb is probably related to the extinction of non-sauropodan sauropodomorphs by the late Pliensbachian^20,38^ that left gravisaurian sauropods as the only surviving sauropodomorphs. This same pattern has been recovered for the pelvic girdle using geometric morphometrics, but not for the cranium^45^. The large size, obligatory quadrupedality and graviportality of sauropods likely imposed morphofunctional constraints that restricted the morphospace occupation in this anatomical region. From an ecological point of view, it has been noted that the diversification of “broad-crowned” early sauropods implied a functional shift of the feeding apparatus toward greater cranial robustness and bite force^20,39,41^. This is consistent with the significantly higher skull disparity values after the TJB and the adoption of obligate high-fiber herbivory^39^, in comparison to the condition present in earlier Sauropodiformes.

The change in locomotion style (bipedal to quadrupedal) and the successive increase in body mass that characterizes Sauropodomorpha over time (Fig. 2d), associated with the acquisition of a fully herbivorous diet (with increased bite force and cranial robustness)^39,40,51,64^, have been found here to be the main attributes responsible for the distribution of Triassic to Middle Jurassic species in the morphospace. A clear pattern is that the disparity of sauropodomorphs radically shifted twice: first after the Carnian/Norian boundary, when plateosaurians radiated^33,38^, and later by the end of the Early Jurassic^22,40,65^, when all non-sauropod sauropodomorphs went extinct and gravisaurians became the predominant megaherbivores during the rest of the Mesozoic Era^20^.

## Methods

### Data matrix and phylogenetic analysis

Our analyses are based on a comprehensive phylogenetic dataset available for Late Triassic–Early Jurassic Sauropodomorpha^33^, composed of 79 taxa and 419 characters. An equally weighted parsimony analysis in TNT 1.5^66^ resulted in 820 most parsimonious trees (MPTs) of 1693 steps (see electronic supplementary material).

### Time bins and calibrated phylogenies

In order to analyse evolutionary patterns of Sauropodomorpha across the TJB we used four time bins: Carnian (233.2–225.7 Ma), Norian–Rhaetian (225.7–201.3 Ma), pre-Toarcian Jurassic (201.3–182.7 Ma), and Toarcian–Middle Jurassic (182.7–163.5 Ma). All the 820 MPTs were time-calibrated with the timePaleoPhy() function of the R package paleotree^67^, using the ‘mbl’ method^68^ with a minimum branch length of 0.1 (Supplementary Data File). These time-calibrated trees were used for the regression analyses and the phylomorphospace plots (see below). We have not used other alternative minimum branch lengths or stochastic calibration methods (e.g., ‘cal3’) because of computational time limitations; 31 regressions, each with 999 iterations, were conducted for each of the 820 trees, resulting in a total of > 25,000,000 runs.

### Morphological disparity analyses

Morphological diversity (disparity) was quantified using the R package Claddis v0.6.3^69^. All non-sauropodomorph terminals and the suprageneric Neosauropoda were excluded before the analyses, resulting in a final dataset of 67 species-level terminals. Morphological disparity was compared between time bins, biped/quadruped locomotion categories (first, using a reduced sample of taxa with locomotion style inferred only from humerus-femur circumference ratio^29^, and second, a broader sample including the latters plus other taxa whose locomotion style was estimated here from a Fitch optimization in the most parsimonious trees), and skull + dentition/vertebrae/pectoral girdle + forelimb/pelvic girdle + hindlimb categories. Three terminals occur across two or three of our a priori selected time bins because of chronostratigraphic uncertainty of the rocks units where they were found (*Isanosaurus*, *Barapasaurus* and *Tazoudasaurus*). These taxa were considered in all possible time bins in the disparity analyses, but sensitivity analyses pruning *Isanosaurus* and *Barapasaurus* were conducted because of their more distant position to where most species occur in the first three axes of the ordinated morphospace. The distance matrix was generated from the taxon-character data matrix using the Maximum Observable Rescaled Distance (MORD^69^) because the commonly used Generalized Euclidean Distance (GED) may produce a strong methodological bias in matrices with a moderate to high amount of missing data^70–72^. Indeed, this is the case in our dataset, in which there is a significant correlation (*p* < 0.001; Pearson's product-moment correlation) between the distance from the centroid and the amount of missing data of each taxon. The distance matrix was used to calculate the pre-ordination metric weighted mean pairwise dissimilarity (WMPD) for all temporal, locomotion, and skeletal groups in order to capture density of each analysed group^73^. We used WMPD because it allows analysing the dataset with all taxa regardless of trimmings necessary for the ordination of the distance matrix.

An ordination of the distance matrix was performed using a Principal Coordinates Analysis (PCoA) with Lingoes correction because of the presence of negative eigenvalues. Six species (*Xixipiosaurus suni*: 84.2% missing data; *Chuxiongosaurus lufengensis*; 84.7%; *Pradhania gracilis*: 94.5%; *Ingentia prima*: 90.4%; *Meroktenos thabanensis*: 90.2%; *Glacialisaurus hammeri*: 92.8%) had to be trimmed to conduct the ordination because of the absence of overlapping scored characters between pairs of taxa. MORD may generate a methodological artefact of repulsion from the centroid of terminals with high amount of missing data^72,74^. We visually identified four of these ‘outliers’ in the first four coordinates (*Chromogisaurus*: 86.16% missing data; *Jaklapallisaurus*: 91.65%; *Plateosaurus ingens*: 94.75%; *Pulanesaura*: 83.53%). We calculated four post-ordination disparity measures with the intention to comprehensively describe the multidimensional morphospace: Sum of Variances (SoV), Sum of Ranges (SoR), and mean displacements from the centroid either with respect to the centroid of the complete dataset or with respect to the previous time bin. We tested how each of these measures captures three different aspects of the morphospace (size, density and position) using the test.metric function of the dispRity package^73,75^, following the simulations of Guillerme et al.^73^. Sum of Variance successfully captured both size (inner size slope = 1.590743e−03 and outer size slope = -1.499010e−03) and density (higher density slope = 1.501165e−03 and lower density slope = −1.888993e−03), but not position (top position slope = −7.066390e−04 and bottom position slope = −1.137366e−04). Sum of Ranges also successfully captured size (inner size slope = 0.07676521 and outer size slope = 0.02702388) and density (higher density slope = 0.07122427 and lower density slope = 0.01671752), but not position (top position slope = 0.03571710 and bottom position slope = 0.03657908). In particular, the proportionally higher inner size slope of Sum of Ranges indicates that this metric was more sensitive to size contraction than Sum of Variances. Finally, the mean displacements from the centroid of all the dataset did not show a good performance for any particular morphospace trait, but it detected position differences when 30% or more of the original data was considered. As a result, in order to complement this metric, the presence of different morphospace positions was tested with PERMANOVA tests among the different temporal categories (with 9,999 permutations, using the ‘euclidean’ method, and excluding *Barapasaurus* and *Isanosaurus*). All the post-ordination measures were calculated with functions of the dispRity package^73,75^ and the PERMANOVA was conducted with the adonis function of the vegan package^76^. All these calculations and tests were conducted using the first 17 coordinates (45.91% of accumulated variance) that were chosen by graphically exploring the scree plot of percentages of explained variance for each coordinate and detecting the last major change of slope in the curve. All the post-ordination metrics were calculated for the complete ordinated data set, and the ordinated data set without ‘outliers’ and without ‘outliers’ and *Barapasaurus* and *Isanosaurus*. In order to compare the pre-ordination and post-ordination results, we calculated the WMPD also for the dataset without the six species that were necessary to trim to conduct the ordination and without *Barapasaurus* and *Isanosaurus*.

Statistical significance between groups was assessed through the non-overlap of 95% confidence intervals calculated from 9999 bootstrap replicates (boot.matrix() function of dispRity) of the dissimilarity matrix for WMPD and the ordinated matrix for the post-ordination disparity metrics, and the subsequent recalculation of the measures. We used an odd number of bootstrap replicates in order to have all values within the two population tails. The 95% confidence intervals were generated using the two tails (0.025%) of the population of values recovered from the resampled matrix. This procedure was followed by all the disparity metrics with the exception of Sum of Ranges, because in this one the resampled values cannot be higher than the original value of the non-resampled dataset. As a result, in the case of Sum of Ranges we used only one tail (0.05%) of the population of resampled values to build the 95% confidence intervals. In addition, we calculated the rarefied and non-rarefied (to seven elements, which is the minimum taxonomic sample among the time bins) confidence intervals for the three post-ordination disparity measures, when analysing the complete dataset, in order to explore how sensitive our dataset is to differences in sample. We have not detected differences in the significance of the results between both methods. These resamples of the original dataset and the analyses including and excluding outlier taxa allow accounting for sample size differences among the different dataset partitions.

Morphospace and phylomorphospace bivariate plots (PCo1 versus PCo2) were generated based on the results of the PCoA (see electronic supplementary material). Phylomorphospaces were created using one, randomly selected time-calibrated tree and the continuous character log(body mass) was mapped, using a maximum likelihood optimization, in one of these phylomorphospaces using the contMap() and phylomorphospace() functions of the R package phytools^77^.

### Regression-based phylogenetic comparative analyses

The relationship between the first three coordinates of the morphospace and different explanatory variables, and several of their combinations (multi-model), were assessed using Procrustes-distance-based phylogenetic regressions^78^ via the procD.pgls function (using type II sum of squares and 999 iterations) of the R package geomorph^79^. We choose to use the first three PCos (16.87% of accumulated variance) because they are the only ones that explain more than 3% of the variance and the curve of the scree plot has its major inflexion point between the PCo3 and PCo4. This regression-based analyses are very sensitive to conflictive information that usually occur in larger PCos with a low amount of explained variance and as a result we decided to exclude them from these tests. These regressions were conducted for each of the 820 time-calibrated MPTs. These analyses included four numerical explanatory variables: (1) log(femoral length), (2) log(body mass), (3) log(humeral shaft circumference)/log(femoral shaft circumference), and (4) tree shape (calculated as the diagonal of the phylogenetic variance–covariance matrix of trees with all branch lengths equal to 1); and two categorical variables: (5) continent of occurrence and (6) locomotion style (biped | quadruped). Locomotion style and humeral-femoral circumferences ratio were used in a first (N = 24 and 15 different models) and a second (N = 36 and 16 different models) series of regressions, but not together, because the former variable has been used to infer the latter^29^. Similarly, in the first series of regressions, log(femoral length) and log(body mass) were not considered together because both variables may covariate^80^. In these regressions, we used the locomotion style of taxa inferred from the humeral-femoral circumference ratio^29^, as well as those estimated here after a Fitch optimization. Regarding topological structure (or phylogenetic structure) as one of the explanatory variables, it is worth to explain that the diagonal of the phylogenetic variance–covariance matrix of a tree with all equal length branches produces a vector with the number of nodes between each terminal taxon and the root of the tree. As a result, this vector describes quantitatively the topology of the phylogenetic tree and it is not equivalent to the diagonal of the variance–covariance matrix generated from the time-calibrated tree that was used to describe the heteroscedasticity of the dataset. The output of the procD.pgls function does not allow to calculate the Akaike’s information criterion; thus, the different models were compared between each other based on their R^2^ and *p* values (see electronic supplementary material).

## Supplementary Information

Supplementary Information 1.

Supplementary Information 2.

Supplementary Information 3.

Supplementary Information 4.

Supplementary Information 5.

Supplementary Information 6.

Supplementary Information 7.

Supplementary Information 8.

Supplementary Information 9.

## Data Availability

Morphological disparity analyses, R scripts and data matrices are available in the Supplementary data files.
