# Supplementary material for: Sauropodomorph evolution across the Triassic–Jurassic boundary: body size, locomotion, and their influence on morphological disparity
Source: Sci Rep. 2021 Nov 18;11:22534. doi: 10.1038/s41598-021-01120-w (PMC8602272; doi:10.1038/s41598-021-01120-w)
Supplement: Supplementary file 1 — Supplementary Information 1. [file 41598_2021_1120_MOESM1_ESM.docx]

**Captions of Supplementary files**

**S1- Supplementary Information**

Supplementary Information for Morphological disparity of Sauropodomorpha- Apaldetti et al

**S2- Ages_R1**

Dataset to temporally calibrate the MPTs (complete taxon sampling).

**S3- Disparity_support_disparity_measure_functions_R**

Disparity functions written for R

**S4- FADLADalltax_R1**

Dataset with temporal data, femoral length, mass estimate, femur/humerus circumference ratio, biogeographic area of occurrence, and locomotion features of Sauropodomorpha

**S5- Function_for_convex_hulls_R**

Function for convex hulls written for R

**S6- Matrix_saurop_only_nex**

Morphological phylogenetic dataset of sauropodomorphs from Tr-Jr, modified from Pol et al.

**S7- Script_for_disparity_FINAL_for_Claddis_061_R1**

Script written for R to calculate morphological disparity of Sauropodomorpha from Tr-Jr

**S8- trees.tre**

MPTs recovered from Pol et al. matrix
