# Supplementary material for: Sauropodomorph evolution across the Triassic–Jurassic boundary: body size, locomotion, and their influence on morphological disparity
Source: Sci Rep. 2021 Nov 18;11:22534. doi: 10.1038/s41598-021-01120-w (PMC8602272; doi:10.1038/s41598-021-01120-w)
Supplement: Supplementary file 2 — Supplementary Information 2. [file 41598_2021_1120_MOESM2_ESM.doc]

Electronic Supplementary material

**Sauropodomorph evolution across the Triassic–Jurassic boundary: body size, locomotion, and their influence on morphological disparity**

Cecilia Apaldetti*^1^, Diego Pol^2^, Martín D. Ezcurra^3^ and Ricardo N. Martínez^4^

**Content**

**1- Percentage of explained variance in each PCo**

**2- Results of the disparity analyses**

**3- Results of the regression-based phylogenetic comparative analyses**

**4- FAD, LAD, Body mass, and Locomotion data for all taxa**

**5- Morphological character list (Pol et al 2021)**

**6- References**

**1- Percentage of explained variance in each PCo**

| PCo axis | % of explained variance | % of accumulated variance |
| --- | --- | --- |
| 1 | 9.8185364 | 9.8185364 |
| 2 | 3.9012558 | 13.7197922 |
| 3 | 3.154572 | 16.8743642 |
| 4 | 2.7217568 | 19.596121 |
| 5 | 2.6163984 | 22.2125194 |
| 6 | 2.4126406 | 24.62516 |
| 7 | 2.2230225 | 26.855385 |
| 8 | 2.1363547 | 28.99174 |
| 9 | 2.098154 | 31.089894 |
| 10 | 2.0350582 | 33.1249522 |
| 11 | 1.9810184 | 35.1059706 |
| 12 | 1.9554477 | 37.0614183 |
| 13 | 1.8580356 | 38.9194539 |
| 14 | 1.8400357 | 40.7594896 |
| 15 | 1.7591754 | 42.518665 |
| 16 | 1.7084063 | 44.2270713 |
| 17 | 1.686414 | 45.9134853 |
| 18 | 1.6635991 | 47.5770844 |
| 19 | 1.6552592 | 49.2323436 |
| 20 | 1.6199031 | 50.8522467 |
| 21 | 1.5850411 | 52.4372878 |
| 22 | 1.5774545 | 54.0147423 |
| 23 | 1.5614069 | 55.5761492 |
| 24 | 1.5513316 | 57.1274808 |
| 25 | 1.5304387 | 58.6579195 |
| 26 | 1.4986447 | 60.1565642 |
| 27 | 1.4918483 | 61.6484125 |
| 28 | 1.4903834 | 63.1387959 |
| 29 | 1.4726561 | 64.611452 |
| 30 | 1.4650102 | 66.0764622 |
| 31 | 1.4539801 | 67.5304423 |
| 32 | 1.4459021 | 68.9763444 |
| 33 | 1.4355018 | 70.4118462 |
| 34 | 1.4248209 | 71.8366671 |
| 35 | 1.4177606 | 73.2544277 |
| 36 | 1.4001716 | 74.6545993 |
| 37 | 1.3849926 | 76.0395919 |
| 38 | 1.3789721 | 77.418564 |
| 39 | 1.3709016 | 78.7894656 |
| 40 | 1.3542638 | 80.1437294 |
| 41 | 1.3435752 | 81.4873046 |
| 42 | 1.336163 | 82.8234676 |
| 43 | 1.3230368 | 84.1465044 |
| 44 | 1.3021301 | 85.4486345 |
| 45 | 1.2858037 | 86.7344382 |
| 46 | 1.2479965 | 87.9824347 |
| 47 | 1.2187269 | 89.2011616 |
| 48 | 1.2118674 | 90.413029 |
| 49 | 1.1731402 | 91.5861692 |
| 50 | 1.1373909 | 92.7235601 |
| 51 | 1.0895769 | 93.813137 |
| 52 | 1.0090497 | 94.8221867 |
| 53 | 0.9916757 | 95.8138624 |
| 54 | 0.893856 | 96.7077184 |
| 55 | 0.8393978 | 97.5471162 |
| 56 | 0.7715269 | 98.3186431 |
| 57 | 0.6854765 | 99.0041196 |
| 58 | 0.5425867 | 99.5467063 |
| 59 | 0.4532939 | 100.000000 |
| 60 | <0.00001 | 100.000000 |
| 61 | <0.00001 | 100.000000 |

**2** **-** **Results of the disparity analyses**

**WMPD all taxa**

Bin WMPD LowerLimit UpperLimit

1 Carnian 0.1419317 0.1246697 0.1591733

2 Norian_Rhaetian 0.2143386 0.2021589 0.2257176

3 pre-Toarcian 0.2494814 0.2339706 0.2639456

4 Toarcian-MJ 0.2188413 0.1898161 0.2449658

**WMPD skull+dentition**

Bin WMPD LowerLimit UpperLimit

1 Carnian 0.1333385 0.1021532 0.1593702

2 Norian_Rhaetian 0.1762757 0.1466695 0.2047809

3 pre-Toarcian 0.2315110 0.2189657 0.2436427

4 Toarcian-MJ 0.1894968 0.1437118 0.2269634

**WMPD vertebrae**

Bin WMPD LowerLimit UpperLimit

1 Carnian 0.1076730 0.07667318 0.1372549

2 Norian_Rhaetian 0.1617679 0.14866349 0.1737576

3 pre-Toarcian 0.2482904 0.22506233 0.2703501

4 Toarcian-MJ 0.2729273 0.23910856 0.3035582

**WMPD shoulder girdle and forelimb**

Bin WMPD LowerLimit UpperLimit

1 Carnian 0.3322809 0.2311212 0.4085134

2 Norian_Rhaetian 0.2617819 0.2416379 0.2812974

3 pre-Toarcian 0.2802169 0.2619181 0.2976315

4 Toarcian-MJ 0.1592199 0.1271022 0.1907008

**WMPD pelvic girdle and hindlimb**

Bin WMPD LowerLimit UpperLimit

1 Carnian 0.1477213 0.1275359 0.1699439

2 Norian_Rhaetian 0.2407149 0.2284395 0.2526654

3 pre-Toarcian 0.2551985 0.2363741 0.2731567

4 Toarcian-MJ 0.1977270 0.1587052 0.2324585

**WMPD without *Barapasaurus* and *Isanosaurus***

Bin WMPD LowerLimit UpperLimit

1 Carnian 0.1419317 0.1251569 0.1595313

2 Norian_Rhaetian 0.2101479 0.1983372 0.2216266

3 pre-Toarcian 0.2329387 0.2186482 0.2464831

4 Toarcian-MJ 0.2220402 0.1866554 0.2512040

**WMPD without Barapasaurus, Isanosaurus, and trimmed taxa for the ordination**

Bin WMPD LowerLimit UpperLimit

1 Carnian 0.1419317 0.1250419 0.1592525

2 Norian_Rhaetian 0.2048743 0.1928166 0.2163910

3 pre-Toarcian 0.2349692 0.2196301 0.2494553

4 Toarcian-MJ 0.2220402 0.1869596 0.2518910

**WMPD locomotion**

Bin WMPD LowerLimit UpperLimit

1 Bipeds 0.2231127 0.2143411 0.2320984

2 Quadrupeds 0.3280687 0.3117793 0.3442396

**WMPD locomotion with reduced sample (only taxa inferred to be biped/quadruped based on humerus/femoral circumferences ratio)**

Bin WMPD LowerLimit UpperLimit

1 Bipeds 0.2236080 0.2043348 0.2423023

2 Quadrupeds 0.3495831 0.3171359 0.3827430

**WMPD locomotion without *Barapasaurus* and *Isanosaurus*, and with reduced sample (only taxa inferred to be biped/quadruped based on humerus/femoral circumferences ratio)**

Bin WMPD LowerLimit UpperLimit

1 Bipeds 0.2236080 0.2046775 0.2418729

2 Quadrupeds 0.3503652 0.3166648 0.3839312

**SoV all taxa**

Bin SoV LowerLimit UpperLimit

1 Carnian 0.1057576 0.09002641 0.1590102

2 Norian_Rhaetian 0.1580963 0.12401888 0.2046489

3 pre-Toarcian 0.1831581 0.14149178 0.2421437

4 Toarcian-MJ 0.2246976 0.21185220 0.2951077

**SoV all taxa with rarefied matrices**

Bin SoV LowerLimit UpperLimit

1 Carnian 0.1057576 0.05748824 0.1266242

2 Norian-Rhaetian 0.1580963 0.11709799 0.2318754

3 pre-Toarcian 0.1831581 0.13352684 0.2650308

4 Toarcian-MJ 0.2246976 0.16509688 0.2554559

**SoR all taxa**

Bin SoR LowerLimit UpperLimit

1 Carnian 3.658997 2.220012 3.658997

2 Norian-Rhaetian 6.750165 5.186897 6.750165

3 pre-Toarcian 7.360617 5.563492 7.360617

4 Toarcian-MJ 6.327877 4.421535 6.327877

**SoR all taxa with rarefaction**

Bin SoR LowerLimit UpperLimit

1 Carnian 3.658997 2.220012 3.658997

2 Norian-Rhaetian 6.750165 5.199508 6.750165

3 pre-Toarcian 7.360617 5.595495 7.360617

4 Toarcian-MJ 6.327877 4.374677 6.327877

**SoV without *Barapasaurus* and *Isanosaurus***

Bin SoV LowerLimit UpperLimit

1 Carnian 0.1057576 0.09002644 0.1577945

2 Norian_Rhaetian 0.1480460 0.11566705 0.1927461

3 pre-Toarcian 0.1589005 0.12183925 0.2106781

4 Toarcian-MJ 0.2088811 0.19617264 0.2878914

**SoR without *Barapasaurus* and *Isanosaurus***

Bin SoR LowerLimit UpperLimit

1 Carnian 3.658997 2.220012 3.658997

2 Norian-Rhaetian 6.409770 4.942242 6.409770

3 pre-Toarcian 6.754225 5.012988 6.754225

4 Toarcian-MJ 5.540536 3.557292 5.540536

**SoV locomotion**

Bin SoV LowerLimit UpperLimit

1 Bipeds 0.1356129 0.1088801 0.1708456

2 Quadrupeds 0.2089300 0.1845635 0.2493610

**SoR locomotion**

Bin SoR LowerLimit UpperLimit

1 Bipeds 6.760765 4.988128 6.760765

2 Quadrupeds 7.959643 6.582425 7.959643

**SoV locomotion with reduced sample (only taxa inferred to be biped/quadruped based on humerus/femoral circumferences ratio)**

Bin SoV LowerLimit UpperLimit

1 Bipeds 0.09982669 0.08137764 0.1376196

2 Quadrupeds 0.21450370 0.20190155 0.2616040

**SoR locomotion with reduced sample (only taxa inferred to be biped/quadruped based on humerus/femoral circumferences ratio)**

Bin SoR LowerLimit UpperLimit

1 Bipeds 3.989991 2.863437 3.989991

2 Quadrupeds 6.419047 5.041879 6.419047

**Displacement from centroid all taxa**

Bin Disp LowerLimit UpperLimit

1 Carnian 1.518088 1.452325 2.312874

2 Norian-Rhaetian 1.063757 1.035988 1.162953

3 pre-Toarcian 1.045143 1.016389 1.130591

4 Toarcian-MJ 1.364435 1.279873 1.751107

**Displacement from centroid all taxa with rarefied matrices**

Bin Disp LowerLimit UpperLimit

1 Carnian 1.518088 1.456125 2.316467

2 Norian-Rhaetian 1.063757 1.035874 1.166847

3 pre-Toarcian 1.045143 1.015806 1.133105

4 Toarcian-MJ 1.364435 1.277244 1.753936

**Displacement from centroid without *Barapasaurus* and *Isanosaurus***

Bin Disp LowerLimit UpperLimit

1 Carnian 1.518088 1.451681 2.316703

2 Norian-Rhaetian 1.076769 1.045589 1.184296

3 pre-Toarcian 1.065124 1.033994 1.164753

4 Toarcian-MJ 1.414241 1.301476 2.042278

**Displacement from previous time bin**

Bin Disp LowerLimit UpperLimit

1 Carnian 1.000000 1.000000 1.000000

2 Norian-Rhaetian 1.425361 1.337646 1.581720

3 pre-Toarcian 1.092128 1.058417 1.189740

4 Toarcian-MJ 1.376450 1.292433 1.774152

**Displacements from previous time bin with rarefied matrices**

Bin Disp LowerLimit UpperLimit

1 Carnian 1.000000 1.000000 1.000000

2 Norian-Rhaetian 1.425361 1.342553 1.577387

3 pre-Toarcian 1.092128 1.058689 1.186708

4 Toarcian-MJ 1.376450 1.292921 1.782071

**Displacement from previous time bin without *Barapasaurus* and *Isanosaurus***

Bin Disp LowerLimit UpperLimit

1 Carnian 1.000000 1.000000 1.000000

2 Norian-Rhaetian 1.444826 1.357890 1.609705

3 pre-Toarcian 1.113187 1.074995 1.227018

4 Toarcian-MJ 1.486488 1.359422 2.116749

**Displacement from centroid of locomotion styles**

Bin Disp LowerLimit UpperLimit

1 Bipeds 1.115236 1.082434 1.206832

2 Quadrupeds 1.018242 1.010159 1.074852

**Displacement from centroid of locomotion styles with reduced sample (only taxa inferred to be biped/quadruped based on humerus/femoral circumferences ratio)**

Bin Disp LowerLimit UpperLimit

1 Bipeds 1.168304 1.124985 1.455898

2 Quadrupeds 1.057024 1.013352 1.284395

**SoV without outliers**

Bin SoV LowerLimit UpperLimit

1 Carnian 0.07926822 0.07719247 0.1157956

2 Norian-Rhaetian 0.14544616 0.11271997 0.1913123

3 pre-Toarcian 0.17497980 0.13337832 0.2319511

4 Toarcian-MJ 0.22469761 0.21285878 0.2919118

**SoR without outliers**

Bin SoR LowerLimit UpperLimit

1 Carnian 2.979611 1.837679 2.979611

2 Norian-Rhaetian 6.220500 4.777762 6.220500

3 pre-Toarcian 7.116181 5.339989 7.116181

4 Toarcian-MJ 6.327877 4.384372 6.327877

**SoV locomotion without outliers**

Bin SoV LowerLimit UpperLimit

1 Bipeds 0.1228465 0.09810701 0.1554002

2 Quadrupeds 0.2040029 0.17969956 0.2470713

**SoR locomotion without outliers**

Bin SoR LowerLimit UpperLimit

1 Bipeds 5.989866 4.514736 5.989866

2 Quadrupeds 7.676636 6.401863 7.676636

**SoV locomotion without outliers with reduced sample (only taxa inferred to be biped/quadruped based on humerus/femoral circumferences ratio)**

Bin SoV LowerLimit UpperLimit

1 Bipeds 0.09982669 0.08171342 0.1375852

2 Quadrupeds 0.21450370 0.20122270 0.2623653

**SoR locomotion without outliers with reduced sample (only taxa inferred to be biped/quadruped based on humerus/femoral circumferences ratio)**

Bin SoR LowerLimit UpperLimit

1 Bipeds 3.989991 2.871366 3.989991

2 Quadrupeds 6.419047 5.037426 6.419047

**SoV without outliers and *Barapasaurus* and *Isanosaurus***

Bin SoV LowerLimit UpperLimit

1 Carnian 0.07926822 0.07719247 0.1148629

2 Norian-Rhaetian 0.13379547 0.10350484 0.1760770

3 pre-Toarcian 0.14898445 0.11354624 0.1982026

4 Toarcian-MJ 0.20888108 0.19565169 0.2866729

**SoR without outliers and *Barapasaurus* and *Isanosaurus***

Bin SoR LowerLimit UpperLimit

1 Carnian 2.979611 1.837679 2.979611

2 Norian-Rhaetian 5.880106 4.520106 5.880106

3 pre-Toarcian 6.434586 4.748810 6.434586

4 Toarcian-MJ 5.540536 3.557292 5.540536

**SoV locomotion without outliers and *Barapasaurus* and *Isanosaurus***

Bin SoV LowerLimit UpperLimit

1 Bipeds 0.1228465 0.09795607 0.1553331

2 Quadrupeds 0.1929811 0.16960442 0.2345779

**SoR locomotion without outliers and *Barapasaurus* and *Isanosaurus***

Bin SoR LowerLimit UpperLimit

1 Bipeds 5.989866 4.520256 5.989866

2 Quadrupeds 7.349750 6.104456 7.349750

**SoV locomotion without outliers and *Barapasaurus* and *Isanosaurus* and with reduced sample (only taxa inferred to be biped/quadruped based on humerus/femoral circumferences ratio)**

Bin SoV LowerLimit UpperLimit

1 Bipeds 0.09982669 0.08091275 0.1382326

2 Quadrupeds 0.20923610 0.19668146 0.2574835

**SoR locomotion without outliers and *Barapasaurus* and *Isanosaurus* and with reduced sample (only taxa inferred to be biped/quadruped based on humerus/femoral circumferences ratio)**

Bin SoR LowerLimit UpperLimit

1 Bipeds 3.989991 2.864500 3.989991

2 Quadrupeds 6.240201 4.810943 6.240201

**Displacement from centroid without outliers**

Bin Disp LowerLimit UpperLimit

1 Carnian 1.625650 1.581678 2.857609

2 Norian-Rhaetian 1.067872 1.040405 1.183833

3 pre-Toarcian 1.050253 1.019406 1.149835

4 Toarcian-MJ 1.364435 1.278028 1.758212

**Displacement from centroid without outliers and *Barapasaurus* and *Isanosaurus***

Bin Disp LowerLimit UpperLimit

1 Carnian 1.625650 1.579905 2.921286

2 Norian-Rhaetian 1.082406 1.053370 1.209875

3 pre-Toarcian 1.073757 1.039936 1.184768

4 Toarcian-MJ 1.414241 1.299803 2.034114

**Displacement from centroid of previous time bin without outliers**

Bin Disp LowerLimit UpperLimit

1 Carnian 1.000000 1.000000 1.000000

2 Norian-Rhaetian 1.440383 1.362028 1.614018

3 pre-Toarcian 1.093226 1.058046 1.199238

4 Toarcian-MJ 1.382411 1.296918 1.788011

**Displacement from centroid of previous time bin without outliers and *Barapasaurus* and *Isanosaurus***

Bin Disp LowerLimit UpperLimit

1 Carnian 1.000000 1.000000 1.000000

2 Norian-Rhaetian 1.462168 1.385847 1.642342

3 pre-Toarcian 1.117463 1.078800 1.238744

4 Toarcian-MJ 1.498310 1.379025 2.144795

**Displacement from centroid of locomotion styles without outliers**

Bin Disp LowerLimit UpperLimit

1 Bipeds 1.127865 1.094952 1.226136

2 Quadrupeds 1.020209 1.011746 1.078902

**Displacement from centroid of locomotion styles without outliers and with reduced sample (only taxa inferred to be biped/quadruped based on humerus/femoral circumferences ratio)**

Bin Disp LowerLimit UpperLimit

1 Bipeds 1.168304 1.123935 1.464610

2 Quadrupeds 1.057024 1.013629 1.288526

**Displacement from centroid of locomotion styles without outliers and *Barapasaurus* and *Isanosaurus***

Bin Disp LowerLimit UpperLimit

1 Bipeds 1.127865 1.095581 1.227695

2 Quadrupeds 1.022285 1.013153 1.085445

**Displacement from centroid of locomotion styles without outliers and *Barapasaurus* and *Isanosaurus* and with reduced sample (only taxa inferred to be biped/quadruped based on humerus/femoral circumferences ratio)**

Bin Disp LowerLimit UpperLimit

1 Bipeds 1.168304 1.124641 1.458248

2 Quadrupeds 1.053697 1.011171 1.303821

**3** **-** **Results of the regression-based phylogenetic comparative analyses**

**Mean of R2, standard deviation of R2,** **and proportion of significant p.values over the total of the Procrustes-distance-based phylogenetic regressions for each model and sampled combination of coordinates using humerus/femoral circumferences ratio.** **Abbreviations: BM, body mass; FL, femoral length; ratio, humerus/femoral circumferences ratio; biogeo, biogeographic occurrence; phylo, phylogenetic structure.**

| Model | Variable | Mean_R2 | Sd_R2 | p<0.05_ratio |
| --- | --- | --- | --- | --- |
| PCOs~FL | femoral_length | 0.388457 | 0.005404 | 1 |
| PCOs~BM | body_mass | 0.467015 | 0.005317 | 1 |
| PCOs~ratio | circumference_ratio | 0.062654 | 0.001248 | 0 |
| PCOs~biogeo | biogeography | 0.374563 | 0.002992 | 1 |
| PCOs~phylo | phylogeny | 0.079383 | 0.002777 | 0 |
| PCOs~FL+ratio | circumference_ratio | 0.062642 | 0.001122 | 0 |
|  | femoral_length | 0.388445 | 0.005348 | 1 |
| PCOs~FL+biogeo | femoral_length | 0.150419 | 0.002125 | 1 |
|  | biogeography | 0.136525 | 0.00656 | 0 |
| PCOs~FL+phylo | femoral_length | 0.340508 | 0.006367 | 1 |
|  | phylogeny | 0.031433 | 0.006439 | 0 |
| PCOs~FL+biogeo+phylo | femoral_length | 0.08347 | 0.001936 | 0.907317 |
|  | biogeography | 0.126002 | 0.004233 | 0 |
|  | phylogeny | 0.02091 | 0.006265 | 0 |
| PCOs~BM+ratio | circumference_ratio | 0.063795 | 0.00087 | 0.224390 |
|  | body_mass | 0.468157 | 0.00506 | 1 |
| PCOs~BM+biogeo | body_mass | 0.221894 | 0.00241 | 1 |
|  | biogeography | 0.129442 | 0.00619 | 0 |
| PCOs~BM+phylo | body_mass | 0.403330 | 0.004954 | 1 |
|  | phylogeny | 0.015698 | 0.004041 | 0 |
| PCOs~BM+ratio+biogeo | circumference_ratio | 0.024100 | 0.001376 | 0 |
|  | body_mass | 0.203031 | 0.001634 | 1 |
|  | biogeography | 0.089747 | 0.005572 | 0 |
| PCOs~BM+ratio+phylo | circumference_ratio | 0.064112 | 0.001063 | 0.146341 |
|  | body_mass | 0.403872 | 0.005827 | 1 |
|  | phylogeny | 0.016015 | 0.004966 | 0 |
| PCOs~biogeo+phylo | biogeography | 0.38304 | 0.005911 | 1 |
|  | phylogeny | 0.087859 | 0.005206 | 0 |

**Mean of R2, standard deviation of R2, and proportion of significant p.values over the total of the Procrustes-distance-based phylogenetic regressions for each model and sampled combination of coordinates using type of locomotion. Abbreviations: BM, body mass; FL, femoral length; ratio, humerus/femoral circumferences ratio; biogeo, biogeographic occurrence; phylo, phylogenetic structure.**

| Model | Variable | Mean_R2 | Sd_R2 | p<0.05_ratio |
| --- | --- | --- | --- | --- |
| PCOs~FL | femoral_length | 0.1463997 | 0.015696 | 1 |
| PCOs~BM | body_mass | 0.179529 | 0.024762 | 1 |
| PCOs~biogeo | biogeography | 0.2304209 | 0.007288 | 0.936585 |
| PCOs~phylo | phylogeny | 0.0831165 | 0.023627 | 0.5 |
| PCOs~locomotion | locomotion | 0.0667844 | 0.004423 | 0 |
| PCOs~FL+biogeo | femoral_length | 0.0736818 | 0.014016 | 0.8 |
|  | biogeography | 0.1577029 | 0.004778 | 0 |
| PCOs~FL+phylo | femoral_length | 0.1094324 | 0.011485 | 1 |
|  | phylogeny | 0.0461491 | 0.018798 | 0.090244 |
| PCOs~FL+locomotion | femoral_length | 0.1333050 | 0.014986 | 1 |
|  | locomotion | 0.0536898 | 0.003673 | 0 |
| PCOs~FL+biogeo+phylo | femoral_length | 0.0438456 | 0.009962 | 0.002439 |
|  | biogeography | 0.1750104 | 0.013139 | 0.265854 |
|  | phylogeny | 0.0634566 | 0.02191 | 0.404878 |
| PCOs~FL+phylo+locomotion | femoral_length | 0.110817 | 0.011244 | 1 |
|  | phylogeny | 0.037692 | 0.014699 | 0 |
|  | locomotion | 0.0452324 | 0.007656 | 0 |
| PCOs~BM+biogeo | body_mass | 0.1183906 | 0.026606 | 0.902439 |
|  | biogeography | 0.1692824 | 0.003193 | 0 |
| PCOs~BM+phylo | body_mass | 0.1288829 | 0.014451 | 1 |
|  | phylogeny | 0.0324703 | 0.015938 | 0.012195 |
| PCOs~BM+locomotion | body_mass | 0.1444920 | 0.022983 | 1 |
|  | locomotion | 0.0317474 | 0.003504 | 0 |
| PCOs~biogeo+phylo | biogeography | 0.2405971 | 0.010331 | 1 |
|  | phylogeny | 0.0932927 | 0.024514 | 0.841463 |
| PCOs~biogeo+locomotion | biogeography | 0.2412179 | 0.007140 | 1 |
|  | locomotion | 0.0775815 | 0.004569 | 0.324390 |
| PCOs~phylo+locomotion | phylogeny | 0.0601795 | 0.020129 | 0.165854 |
|  | locomotion | 0.0438475 | 0.007802 | 0 |

**4** **- FAD, LAD, Body mass, and Locomotion data for all taxa.**

Data for the following table have been compiled from the following sources:

**FAD-LAD**. FAD and LAD were modified from Pol et al. (2021), with the update of the following taxa: FAD of *Nambalia*, *Ruehleia*, *Jaklapalisaurus*, *P. gracilis*, *P. engelhardti* changed from 227 Ma to 225.4 Ma. This change is based on the radioisotopic date of Langer et al. (2019) for the Caturrita Formation (*Riograndia* AZ) that marks the onset of vertebrate assemblages dominated by early sauropodomorphs and has been correlated with other Norian beds of Europe and India (see Novas et al., 2021: fig 3). FAD of *M. carinatus*, *Aardonyx*, NMQR3314, *Antetonitrus*, *Ledumahadi* and *Pulanesaura* from 202.3 to 201.3 based on Bordy et al. (2020). LAD of *Glacialisaurus* from 182.0 to 182.7 based on Elliot et al. (2016).

**Femoral length**. These measurements follow those compiled by Pradelli et al. (2021). Femoral length values estimated by Pradelli et al. (in press) or other authors (see Pradelli et al. in press: supplementary information) based on other femoral or long bone measurements are indicated with an asterisk.

**Body mass**. Body mass calculated using the equation of Campione et al. (2014) for estimating body mass in bipeds [function bipeds using the cQE.eq = raw option of the package MASSTIMATE version 2.0-1; Campione, 2020). This equation uses the femoral circumference to infer body mass, which has been taken from McPhee et al. (2018) for most sauropodomorphs, except for the following bipedal or inferred as bipedal taxa:

*Adeopapposaurus*: femoral minimum circumference of 90.0 mm (PVSJ 610; Roland Sookias pers. comm.).

*Bagualosaurus*: femoral minimum circumference of 71.5 mm (UFRGS-PV-1099-T; pers. obs.).

*Buriolestes*: femoral minimum circumference of 39 mm (ULBRA-PVT280; pers. obs.).

*Chromogisaurus*: femoral minimum circumference of 56 mm (McPhee et al. 2018).

*Coloradisaurus*: femoral minimum circumference of 200.8 mm (PVL 5904; pers. obs.).

*Efraasia*: femoral length of 627 mm (Yates 2003; SMNS 12843) and femoral minimum circumference of 145.4 mm (SMNS 12354; pers. obs.).

*Macrocollum*: femoral minimum circumference of 111.5 mm (CAPPA/UFSM 0001b; pers. obs.).

*Massospondylus*: femoral minimum circumference of 214 mm (Chapelle et al. 2020).

*Pampadromaeus*: femoral minimum circumference of 48 mm (McPhee et al. 2018).

*Pantydraco*: we excluded this taxon from analyses considering femoral length or body mass because the only known specimen represents a juvenile that was likely far from the adult body size.

*Ruehleia*: femoral minimum circumference of 264 mm (MB.R.4718; pers. obs.).

**Locomotion**. The postural data for the locomotion categories was taken from McPhee et al. (2018). All sauropodomorph taxa listed as bipedal or quadrupedal in the column *Locomotion_red* are those inferred to have the corresponding type of locomotion in McPhee et al. (2018) based on the femoral to humeral circumference ratio. The data listed in the column *Locomotion* is the most parsimonious inference on the type of locomotion obtained through the Fitch optimization of the character bipedal vs quadrupedal using the information from the column *Locomotion_red* (McPhee et al. 2018). The data in the column *Locomotion* therefore contains the same information as *Locomotion_red* for many taxa but has additional information after inferring the most parsimonious mode of locomotion in the phylogenetic trees.

| Taxon | FAD | LAD | FL | Mass | log(Hc)/log(Fc) | Area | Locomotion | Locomotion_red |
| --- | --- | --- | --- | --- | --- | --- | --- | --- |
| Eoraptor | 231.7 | 226.27 | 15.2 | 0.0173 | 0.86 | SA | Biped | Biped |
| Saturnalia | 233.84 | 226.27 | 15.7 | 0.0106 | 0.88 | SA | Biped | Biped |
| Panphagia | 231.7 | 231.1 | 19.0* | NA | NA | SA | Biped | NA |
| Chromogisaurus | 231.7 | 231.1 | 17.3 | 0.0130 | NA | SA | Biped | NA |
| Buriolestes | 233.84 | 226.27 | 13.6 | 0.0048 | NA | SA | Biped | NA |
| Pampadromaeus | 233.84 | 226.27 | 14.5 | 0.0085 | NA | SA | Biped | NA |
| Bagualosaurus | 233.84 | 226.27 | 21.5 | 0.0255 | NA | SA | Biped | NA |
| Nambalia | 225.4 | 208.5 | 27.3 | NA | NA | IN | Biped | NA |
| Thecodontosaurus | 208.5 | 201.3 | 21.0* | NA | NA | EU | Biped | NA |
| Pantydraco | 208.5 | 201.3 | NA | NA | NA | EU | Biped | NA |
| Efraasia | 215.56 | 212 | 62.7 | 0.1797 | 0.92 | EU | Biped | Biped |
| Ruehleia | 225.4 | 208.5 | 80.0 | 0.9263 | 0.93 | EU | Biped | Biped |
| Jaklapalisaurus | 225.4 | 208.5 | 40.9* | NA | NA | IN | NA | NA |
| Macrocollum | 225.67 | 220 | 33.4 | 0.0866 | 0.91 | SA | Biped | Biped |
| Unaysaurus | 225.67 | 220 | 27.0* | NA | NA | SA | Biped | NA |
| Plateosaurus_ingens | 212 | 205.6 | NA | NA | NA | EU | Biped | NA |
| Plateosaurus_gracilis | 225.4 | 208.5 | 54.3 | NA | NA | EU | Biped | NA |
| Plateosaurus_engelhardti | 225.4 | 201.3 | 93.0 | 0.917 | 0.91 | EU | Biped | Biped |
| Pradhania | 199.3 | 190.8 | NA | NA | NA | IN | Biped | NA |
| Glacialisaurus | 199 | 182.7 | 60.0* | NA | NA | ANT | Biped | NA |
| Coloradisaurus | 220 | 213 | 51.93 | 0.437 | NA | SA | Biped | NA |
| Yunnanosaurus_huangi | 201.3 | 190.8 | NA | 0.574 | 0.94 | AS | Biped | Biped |
| Lufengosaurus | 201.3 | 190.8 | 78.0 | 0.481 | 0.92 | AS | Biped | Biped |
| Xixipiosaurus | 201.3 | 190.8 | 42.8 | 0.341 | NA | AS | Biped | NA |
| Massospondylus_carinatus | 201.3 | 187.5 | 55.0* | 0.520 | 0.92 | AF | Biped | Biped |
| Adeopapposaurus | 201.3 | 190.8 | 22.7 | 0.0481 | NA | SA | Biped | NA |
| Leyesaurus | 201.3 | 190.8 | 38.31* | NA | NA | SA | Biped | NA |
| Plateosauravus | 219.6 | 202.3 | 60.0 | 1.350 | NA | AF | Biped | NA |
| Riojasaurus | 220 | 213 | 60.8 | 2.230 | 0.96 | SA | Quadruped | Quadruped |
| Eucnemesaurus_fortis | 219.6 | 202.3 | 47.9* | 1.428 | 0.97 | AF | Quadruped | NA |
| Eucnemesaurus_entaxonis | 219.6 | 202.3 | 53.3 | NA | NA | AF | Quadruped | NA |
| Seitaad | 190.8 | 182.7 | 32.6* | NA | NA | NAM | NA | NA |
| Anchisaurus | 201.3 | 190.8 | 28.0 | 0.260 | 0.98 | NAM | Quadruped | Quadruped |
| Chuxiongosaurus | 201.3 | 190.8 | NA | NA | NA | AS | Quadruped | NA |
| Jingshanosaurus | 201.3 | 190.8 | 84.5 | 3.105 | 0.96 | AS | Quadruped | Quadruped |
| Xingxiulong | 201.3 | 190.8 | 61.4 | 1.910 | NA | AS | Quadruped | Quadruped |
| Sarahsaurus | 199.3 | 183.7 | 34.3 | 0.1617 | 0.92 | NAM | Biped | Biped |
| Yizhousaurus | 201.3 | 190.8 | 81.5* | NA | NA | AS | Quadruped | NA |
| Kholumolumo | 219.6 | 202.3 | 75.5 | 3.500 | NA | AF | Quadruped | NA |
| Mussaurus | 192.7 | 192.6 | 80.0 | 2.850 | 0.89 | SA | Biped | Biped |
| Leonerasaurus | 189 | 188.8 | 35.3* | NA | NA | SA | Quadruped | NA |
| Sefapanosaurus | 219.6 | 202.3 | 64.22* | NA | NA | AF | Quadruped | NA |
| Aardonyx | 201.3 | 187.5 | 68.1 | NA | NA | AF | Quadruped | NA |
| Meroktenos | 219.6 | 202.3 | NA | NA | NA | AF | Quadruped | NA |
| NMQR3314 | 201.3 | 187.5 | 57 | NA | NA | AF | Quadruped | Quadruped |
| NMQR1551 | 219.6 | 202.3 | 64 | 1.650 | NA | AF | Quadruped | NA |
| Ingentia | 213 | 201.3 | 105.3 | NA | NA | SA | Quadruped | NA |
| Lessemsaurus | 220 | 213 | 87.5 | 7.000 | 0.87 | SA | Quadruped | NA |
| Antetonitrus | 201.3 | 187.5 | 79.4 | 5.640 | 0.95 | AF | Quadruped | Quadruped |
| Ledumahadi | 201.3 | 187.5 | 104.6* | 12.000 | NA | AF | Quadruped | Quadruped |
| Blikanasaurus | 219.6 | 202.3 | 49.95* | NA | NA | AF | Quadruped | NA |
| Camelotia | 208.5 | 201.3 | 100.8 | 3.800 | 0.97 | EU | Quadruped | NA |
| Pulanesaura | 201.3 | 187.5 | 92.2* | NA | NA | AF | Quadruped | NA |
| Gongxianosaurus | 182.7 | 174.1 | 116.4 | NA | NA | AS | Quadruped | NA |
| Isanosaurus | 208.5 | 174.1 | 76.0 | 3.504 | 0.97 | AS | Quadruped | Quadruped |
| Tazoudasaurus | 190.8 | 174.1 | 123.0 | 10.300 | 0.97 | AF | Quadruped | Quadruped |
| Vulcanodon | 199.3 | 182.7 | 110.0 | 10.300 | 0.95 | AF | Quadruped | Quadruped |
| Shunosaurus | 161 | 157 | 125 | 6.532 | 0.95 | AS | Quadruped | Quadruped |
| Spinophorosaurus | 174.1 | 163.5 | NA | 10.057 | 0.98 | AF | Quadruped | Quadruped |
| Patagosaurus | 178.7 | 178.1 | 127.0 | 24.385 | 0.97 | SA | Quadruped | Quadruped |
| Barapasaurus | 190.8 | 170.3 | 136.5 | NA | NA | IN | Quadruped | NA |
| Cetiosaurus | 170.3 | 163.5 | NA | 27.300 | 0.98 | EU | Quadruped | Quadruped |
| Omeisaurus | 161 | 157 | 131 | 7.800 | 0.97 | AS | Quadruped | Quadruped |
| Mamenchisaurus | 163.5 | 152.1 | 123 | 6.240 | 0.98 | AS | Quadruped | Quadruped |
| Ngwevu_intloko | 201.3 | 187.5 | 28.5 | NA | NA | AF | Biped | NA |
| Irisosaurus_yimenensis | 201.3 | 190.8 | NA | NA | NA | AS | Quadruped | NA |
| Schleitheimia_schutzi | 213 | 208.5 | 69.0 | NA | NA | EU | Quadruped | NA |

**5- Morphological character list (Pol et al. 2021)**

The complete list of characters used in the phylogenetic analysis is that provided by Pol et al. (2021). The following multistate characters were treated as ordered: 8, 13, 19, 23, 40, 57, 69, 92, 102, 108, 117, 121, 134, 144, 147, 149, 150, 157, 167, 170, 171, 177, 183, 205, 207, 214, 222, 227, 242, 251, 254, 277, 299, 336, 342, 349, 353, 370, 393, 404, and 409.

This character list include the characters listed by Yates (2007) and the characters added by Smith and Pol (2007), Ezcurra (2010), Novas et al. (2011), Apaldetti el al*.* (2013), Otero and Pol (2013), Otero et al. (2015), McPhee et al. (2015, 2017), McPhee and Choiniere (2017), Bronzati and Rauhut (2017), Bronzati et al. (2018), Cerda et al. (2017), Chapelle and Choiniere (2018).

1. Skull to femur ratio: greater than (0), or less than (1), 0.6 (modified from Gauthier 1986).

2. Lateral plates appressed to the labial side of the premaxillary, maxillary and dentary teeth: absent (0) or present (1) (Upchurch, 1995).

3. Relative height of the rostrum at the posterior margin of the naris: more than (0), or less than (1), 0.6 of the height of the skull at the middle of the orbit (Langer, 2004).

4. Foramen on the lateral surface of the premaxillary body: absent (0) or present (1).

5. Distal end of the dorsal premaxillary process: tapered (0) or transversely expanded (1) (Sereno, 1999).

6. Profile of premaxilla: convex (0) or with an inflection at the base of the dorsal process (1) (Upchurch, 1995).

7. Size and position of the posterolateral process of premaxilla: large and lateral to the anterior process of the maxilla (0) or small and medial to the anterior process of the maxilla (1).

8. Relationship between posterolateral process of the premaxilla and the anteroventral process of the nasal: broad sutured contact (0), point contact (1), or briefly separated by maxilla (2), or well separated from each other by the entire posterior margin of the external nares (3) (modified from Gauthier, 1986). Ordered.

9. Posteromedial process of the premaxilla: absent (0) or present (1) (Rauhut, 2003).

10. Shape of the anteromedial process of the maxilla: narrow, elongated and projecting anterior to lateral premaxilla-maxilla suture (0) or short, broad and level with lateral premaxilla-maxilla suture (1).

11. Development of external narial fossa: absent to weak (0) or well developed with sharp posterior and anteroventral rims (1).

12. Development of narial fossa on the anterior ramus of the maxilla: weak and orientated laterally to dorsolaterally (0) or well developed and forming a horizontal shelf (1) (modified from Upchurch, 1995).

13. Size and position of subnarial foramen: absent (0), small (no larger than adjacent maxillary neurovascular foramina) and positioned outside of narial fossa (1), or large and on the rim of, or inside, the narial fossa (2) (modified from Sereno *el al.,* 1993). Ordered.

14. Shape of subnarial foramen: rounded (0) or slot-shaped (1).

15. Maxillary contribution to the margin of the narial fossa: absent (0) or present (1).

16. Diameter of external naris: less than (0), or greater than (1), 0.5 of the orbital diameter (Wilson & Sereno, 1998).

17. Shape of the external naris (in adults): rounded (0) or subtriangular with an acute posteroventral corner (1) (Galton & Upchurch, 2004).

18. Level of the anterior margin of the external naris: anterior to (0) or posterior to (1) the midlength of the premaxillary body (Rauhut, 2003).

19. Level of the posterior margin of external naris: anterior to, or level with the premaxilla-maxilla suture (0), posterior to the first maxillary alveolus (1), or posterior to the midlength of the maxillary tooth row and the anterior margin of the antorbital fenestra (2) (modified from Wilson & Sereno, 1998). Ordered.

20. Dorsal profile of the snout: straight to gently convex (0) or with a depression behind the naris (1).

21. Elongate median nasal depression: absent (0) or present (1) (Sereno, 1999).

22. Width of anteroventral process of nasal at its base: less than (0) or greater than (1) width of anterodorsal process at its base (modified from Sereno, 1999).

23. Nasal relationship with dorsal margin of antorbital fossa: not contributing to the margin of the antorbital fossa (0), lateral margin overhangs the antorbital fossa and forms its dorsal margin (1), overhang extensive, obscuring the dorsal lachrymal-maxilla contact in lateral view (2) (modified from Sereno, 1999).

24. Pointed caudolateral process of the nasal overlapping the lachrymal: absent (0) or present (1) (Sereno, 1999).

25. Anterior profile of the maxilla: slopes continuously towards the rostral tip (0) or with a strong inflection at the base of the ascending ramus, creating a rostral ramus with parallel dorsal and ventral margins (1) (Sereno *el al.,* 1996).

26. Length of rostral ramus of the maxilla: less than (0), or greater than (1), its dorsoventral depth (Sereno *el al*., 1996).

27. Shape of the main body of the maxilla: tapering posteriorly (0) or dorsal and ventral margins parallel for most of their length (1).

28. Shape of the ascending ramus of the maxilla in lateral view: tapering dorsally (0) or with an anteroposterior expansion at the dorsal end (1).

29. Rostrocaudal length of the antorbital fossa: greater (0), or less (1), than that of the orbit (Yates, 2003a).

30. Posteroventral extent of medial wall of antorbital fossa: reaching (0), or terminating anterior to (1), the anterior tip of the jugal (modified from Galton & Upchurch, 2004).

31. Development of the antorbital fossa on the ascending ramus of the maxilla: deeply impressed and delimited by a sharp, scarp-like rim (0) or weakly impressed and delimited by a rounded rim or a change in slope (1).

32. Shape of the antorbital fossa: crescentic with a strongly concave posterior margin that is roughly parallel to the rostral margin of the antorbital fossa (0), subtriangular with a straight to gently concave posterior margin (1), or antorbital fossa absent (2) (modified from Galton, 1985).

33. Size of the neurovascular foramen at the caudal end of the lateral maxillary row: not larger than the others (0) or distinctly larger than the others in the row (1) (Yates, 2003a).

34. Direction that the neurovascular foramen at the caudal end of the lateral maxillary row opens: caudally (0) or rostrally, ventrally or laterally (1) (modified from Sereno, 1999).

35. Arrangement of lateral maxillary neurovascular foramina: linear (0) or irregular (1) (modified from Sereno, 1999).

36. Longitudinal ridge on the posterior lateral surface of the maxilla: absent (0) or present (1) (Barrett , Upchurch & Wang, 2005).

37. Dorsal exposure of the lachrymal: present (0) or absent (1) (Gauthier, 1986).

38. Shape of the lachrymal: dorsoventrally short and blocks shaped (0) or dorsoventrally elongate and shaped like an inverted L (1) (Rauhut, 2003).

39. Orientation of the lachrymal orbital margin: strongly sloping anterodorsally (0) or erect and close to vertical (1).

40. Length of the anterior ramus of the lachrymal: greater than (0), or less than (1), half the length of the ventral ramus, or absent altogether (2) (modified from Galton, 1990). Ordered.

41. Web of bone spanning junction between anterior and ventral rami of lachrymal: absent and antorbital fossa laterally exposed (0) or present, obscuring posterodorsal corner of antorbital fossa (1).

42. Extension of the antorbital fossa onto the ventral end of the lachrymal: present (0) or absent (1) (modified from Wilson & Sereno, 1998).

43. Length of the caudal process of the prefrontal: short (0), or elongated (1), so that total prefrontal length is equal to the rostrocaudal diameter of the orbit (Galton, 1985).

44. Ventral process of prefrontal extending down the posteromedial side of the lachrymal: present (0) or absent (1) (Wilson & Sereno, 1998).

45. Maximum transverse width of the prefrontal: less than (0), or more than (1), 0.25 of the skull width at that level (modified from Galton, 1990).

46. Shape of the orbit: subcircular (0) or ventrally constricted making the orbit subtriangular (1) (Wilson & Sereno, 1998).

47. Slender anterior process of the frontal intruding between the prefrontal and the nasal: absent (0) or present (1) (modified from Sereno, 1999).

48. Jugal-lachrymal relationship: lachrymal overlapping lateral surface of jugal or abutting it dorsally (0), or jugal overlapping lachrymal laterally (1) (Sereno *el al.,* 1993).

49. Shape of the suborbital region of the jugal: an anteroposteriorly elongate bar (0) or an anteroposteriorly shortened plate (1).

50. Jugal contribution to the antorbital fenestra: present (0) or absent (1) (Holtz, 1994).

51. Dorsal process of the anterior jugal: present (0) or absent (1) (modified from Rauhut 2003a).

52. Ratio of the minimum depth of the jugal below the orbit to the distance between the rostral end of the jugal and the rostroventral corner of the infratemporal fenestra: less than (0), or greater than (1), 0.2 (modified from Galton, 1985).

53. Transverse width of the ventral ramus of the postorbital: less than (0), or greater than (1), its rostrocaudal width at midshaft (Wilson & Sereno, 1998).

54. Shape of the dorsal margin of postorbital in lateral view: straight to gently curved (0) or with a distinct embayment between the anterior and posterior dorsal processes (1).

55. Height of the postorbital rim of the orbit: flush with the posterior lateral process of the postorbital (0) or raised so that it projects laterally to the posterior dorsal process (1).

56. Postfrontal bone: present (0) or absent (1) (Sereno *el al.,* 1993).

57. Position of the rostral margin of the infratemporal fenestra: behind the orbit (0), extends under the rear half of the orbit (1), or extends as far forward as the midlength of the orbit (2) (modified from Upchurch, 1995). Ordered.

58. Frontal contribution to the supratemporal fenestra: present (0) or absent (1) (modified from Gauthier, 1986).

59. Orientation of the long axis of the supratemporal fenestra: longitudinal (0) or transverse (1) (Wilson & Sereno, 1998).

60. Medial margin of supratemporal fossa: simple smooth curve (0) or with a projection at the frontal ⁄ postorbital-parietal suture producing a scalloped margin (1) (Leal *el al.,* 2004).

61. Length of the quadratojugal ramus of the squamosal relative to the width at its base: less than (0), or greater than (1), four times its width (Sereno, 1999).

62. Proportion of infratemporal fenestra bordered by squamosal: more than (0), or less than (1), 0.5 of the depth of the infratemporal fenestra.

63. Squamosal-quadratojugal contact: present (0) or absent (1) (Gauthier, 1986).

64. Angle of divergence between jugal and squamosal rami of quadratojugal: close to 90° (0) or close to parallel (1).

65. Length of jugal ramus of quadratojugal: no longer than (0), or longer than (1), the squamosal ramus (Wilson & Sereno, 1998).

66. Shape of the rostral end of the jugal ramus of the quadratojugal: tapered (0) or dorsoventrally expanded (1) (Wilson & Sereno, 1998).

67. Relationship of quadratojugal to jugal: jugal overlaps the lateral surface of the quadratojugal (0), quadratojugal overlaps the lateral surface of the jugal (1), or quadratojugal sutures along the ventrolateral margin of the jugal (2). Unordered.

68. Position of the quadrate foramen: on the quadrate-quadratojugal suture (0), deeply incised into, and partly encircled by, the quadrate (1), or on the quadrate-squamosal suture, just below the quadrate head (2) (modified from Rauhut, 2003). Unordered.

69. Shape of posterolateral margin of quadrate: sloping anterolaterally from posteromedial ridge (0), everted posteriorly creating a posteriorly facing fossa (1), posterior fossa deeply excavated, invading quadrate body (2) (Wilson & Sereno, 1998). Ordered.

70. Exposure of the lateral surface of the quadrate head: absent, covered by lateral sheet of the squamosal (0) or present (1) (Sereno *el al.,* 1993).

71. Proportion of the length of the quadrate that is occupied by the pterygoid wing: at least 70% (0) or less than 70% (1) (Yates, 2003a).

72. Depth of the occipital wing of the parietal: less than (0), or more than (1), 1.5 times the depth of the foramen magnum (Wilson & Sereno, 1998).

73. Position of foramina for mid-cerebral vein on occiput: between supraoccipital and parietal (0) or on the supraoccipital (1) (modified from Yates, 2003a).

74. Postparietal fenestra between supraoccipital and parietals: absent (0) or present (1).

75. Shape of the supraoccipital: diamond-shaped, at least as high as wide (0), or semilunate and wider than high (1) (Yates, 2003b).

76. Orientation of the supraoccipital plate: erect to gently sloping (0) or strongly sloping forward so that the dorsal tip lies level with the basipterygoid processes (1) (Galton & Upchurch, 2004).

77. Orientation of the paroccipital processes in occipital view: slightly dorsolaterally directed to horizontal (0) or ventrolaterally directed (1) (Rauhut, 2003).

78. Orientation of the paroccipital processes in dorsal view: posterolateral forming a V-shaped occiput (0) or lateral forming a flat occiput (1) (Wilson, 2002).

79. Size of the post-temporal fenestra: large fenestra (0) or a small hole that is much less than half the depth of the paroccipital process (1).

80. Exit of the mid-cerebral vein: through trigeminal foramen (0) or through a separate foramen (1) (Rauhut, 2003).

81. Shape of the floor of the braincase in lateral view: relatively straight with the basal tuberae, basipterygoid processes and parasphenoid rostrum roughly aligned (0), bent with the basipterygoid processes and the parasphenoid rostrum below the level of the basioccipital condyle and the basal tuberae (1), or bent with the basal tuberae lowered below the level of the basioccipital and the parasphenoid rostrum raised above it (2) (modified from Galton 1990). Unordered.

82. Basioccipital component of basal tubera, medial component in relation to the parabasisphenoidal components: present (0), or absent (1) (Bronzati and Rauhut, 2017).

83. Length of the basipterygoid processes (from the top of the parasphenoid to the tip of the process): less than (0), or greater than (1), the height of the braincase (from the top of the parasphenoid to the top of the supraoccipital) (Benton *el al.,* 2000).

84. Basioccipital-parabasisphenoid junction on the ventral surface of the bones: straight line (0), or U/V-shaped (1) (Bronzati and Rauhut, 2017).

85. Subsellar recess: maximum width equal or greater than the dorsoventral height (0) or maximum width smaller than the dorsoventral height (1) (Bronzati and Rauhut, 2017).

86. Dorsoventral depth of the parasphenoid rostrum: much less than (0), or about equal to (1), the transverse width (Yates, 2003a).

87. Shape of jugal process of ectopterygoid: gently curved (0) or strongly recurved and hook-like (1) (Yates, 2003).

88. Pneumatic fossa on the ventral surface of the ectopterygoid: absent (0) or present (1) (Sereno *el al.,* 1996).

89. Relationship of the ectopterygoid to the pterygoid: ectopterygoid overlapping the ventral (0), or dorsal (1), surface of the pterygoid (Sereno *el al.,* 1993).

90. Position of the maxillary articular surface of the palatine: along the lateral margin of the bone (0) or at the end of a narrow anterolateral process due to the absence of the posterolateral process (1) (Wilson & Sereno, 1998).

91. Centrally located tubercle on the ventral surface of palatine: absent (0) or present (1).

92. Medial process of the pterygoid forming a hook around the basipterygoid process: absent (0), flat and blunt-ended (1), or bent upward and pointed (2) (modified from Wilson & Sereno, 1998). Ordered.

93. Length of the vomers: less than (0), or more than (1), 0.25 of the total skull length.

94. Position of jaw joint: no lower than the level of the dorsal margin of the dentary (0) or depressed well below this level (1) (Sereno, 1999).

95. Shape of upper jaws in ventral view: narrow with an acute rostral apex (0) or broad and U-shaped (1) (Wilson & Sereno, 1998).

96. Length of the external mandibular fenestra: more than (0), or less than (1), 0.1 of the length of the mandible (modified from Upchurch, 1995).

97. Caudal end of dentary tooth row medially inset with a thick lateral ridge on the dentary forming a buccal emargination: absent (0) or present (1) (Gauthier, 1986).

98. Height: length ratio of the dentary: less than (0), or greater than (1), 0.2 (modified from Benton *el al.,* 2000).

99. Orientation of the symphyseal end of the dentary: in line with the long axis of the dentary (0) or strongly curved ventrally (1) (Sereno, 1999).

100. Position of first dentary tooth: adjacent to symphysis (0) or inset one tooth’s width from the symphysis (1) (Sereno, 1999).

101. Dorsoventral expansion at the symphyseal end of the dentary: absent (0) or present (1) (Wilson & Sereno, 1998).

102. Splenial foramen: absent (0), present and enclosed (1), or present and open anteriorly (2) (Rauhut, 2003). Ordered.

103. Splenial-angular joint: flattened sutured contact (0), synovial joint surface between tongue-like process of angular fitting in groove of the splenial (1) (Sereno *el al.,* 1993).

104. A stout, triangular, medial process of the articular, behind the glenoid: present (0) or absent (1) (Yates, 2003a).

105. Length of the retroarticular process: less than (0), or greater than (1), than the depth of the mandible below the glenoid (Yates, 2003).

106. Strong medial embayment behind glenoid of the articular in dorsal view: absent (0), or present (1) (Yates & Kitching, 2003).

107. Number of premaxillary teeth: four (0) or more than four (1) (Galton, 1990).

108. Number of dentary teeth (in adults): less than 18 (0) or 18 or more (1) (modified from Wilson & Sereno, 1998).

109. Arrangement of teeth within the jaws: linearly placed, crowns not overlapping (0) or imbricated with distal side of tooth overlapping mesial side of the succeeding tooth (1).

110. Orientation of the maxillary tooth crowns: erect (0) or procumbent (1) (modified from Gauthier, 1986).

111. Orientation of the dentary tooth crowns: erect (0) or procumbent (1) (modified from Gauthier, 1986).

112. Teeth with basally constricted crowns: absent (0) or present (1) (Gauthier, 1986).

113. Tooth–tooth occlusal wear facets: absent (0) or present (1) (Wilson & Sereno, 1998).

114. Mesial and distal serrations of the mid-posterior teeth: fine and set at right angles to the margin of the tooth (0) or coarse and angled upwards at an angle of 45° to the margin of the tooth (1) (Benton *el al.,* 2000).

115. Distribution of serrations on the maxillary and mid-posterior dentary teeth: present on both the mesial and distal carinae (0), absent on the posterior carinae (1), or absent on both carinae (2) (Wilson, 2002). Unordered.

116. Long axis of the tooth crowns distally recurved: present (0) or absent (1) (Gauthier, 1986).

117. Texture of the enamel surface: entirely smooth (0), finely wrinkled in some patches (1), or extensively and coarsely wrinkled (2) (modified from Wilson & Sereno, 1998).

118. Lingual concavities of the teeth: absent (0) or present (1) (Upchurch, 1995).

119. Longitudinal labial grooves on the teeth: absent (0) or present (1) (Upchurch, 1998).

120. Distribution of the serrations along the mesial and distal carinae of the mid-posterior teeth: extend along most of the length of the crown (0) or are restricted to the upper half of the crown (1) (Yates, 2003a).

Vertebrae

121. Number of cervical vertebrae: eight or fewer (0), 9–10 (1), 12–13 (2), or more than 13 (3) (modified from Wilson & Sereno, 1998). Ordered.

122. Shallow, dorsally facing fossa on the atlantal neurapophysis bordered by a dorsally everted lateral margin: absent (0) or present (1) (Yates & Kitching, 2003).

123. Width of axial intercentrum: less than (0), or greater than (1), width of axial centrum (Sereno, 1999).

124. Position of axial prezygapophyses: on the anterolateral surface of the neural arch (0) or mounted on anteriorly projecting pedicels (1).

125. Posterior margin of the axial postzygapophyses: overhang the axial centrum (0) or are flush with the caudal face of the axial centrum (1) (Sereno, 1999).

126. Length of the axial centrum: less than (0), or at least (1), three times the height of the centrum.

127. Length of the anterior cervical centra (cervicals 3–5): no more than (0), or greater than (1), the length of the axial centrum.

128. Length of middle to posterior cervical centra (cervicals 6–8): no more than (0), or greater than (1), the length of the axial centrum.

129. Dorsal excavation of the cervical parapophyses: absent (0) or present (1) (Upchurch, 1998).

130. Lateral compression of the anterior cervical vertebrae: centra are no higher than they are wide (0) or are approximately 1.25 times higher than wide (1) (Upchurch, 1998).

131. Relative elongation of the anterior cervical centra (cervicals 3–5): lengths of the centra are less than 2.5 times the height of their anterior faces (0), lengths are 2.5–4 times the height of their anterior faces (1) or the length of at least cervical 4 or 5 exceeds 4 times the anterior centrum height (2) (modified from Sereno, 1999). Ordered.

132. Ventral keels on cranial cervical centra: present (0) or absent (1) (modified from Upchurch, 1998).

133. Height of the mid cervical neural arches: no more than (0), or greater than (1), height of the posterior centrum face.

134. Cervical epipophyses on the dorsal surface of the postzygapophyses: absent (0), or present on at least some cervical vertebrae (1).

135. Caudal ends of cranial, postaxial epipophyses: with a free pointed tip (0) or joined to the postzygapophysis along their entire length (1).

136. Shape of the epipophyses: tall ridges (0) or flattened, horizontal plates (1) (Yates, 2003a).

137. Epipophyses overhanging the rear margin of the postzygapophyses: absent (0), or present in at least some postaxial cervical vertebrae (1) (Sereno *el al.,* 1993).

138. Anterior spur-like projections on mid-cervical neural spines: absent (0) or present (1).

139. Shape of mid cervical neural spines: less than (0), or at least (1), twice as long as high.

140. Shape of cervical rib shafts: short and posteroventrally directed (0) or longer than the length of their centra and extending parallel to cervical column (1) (Sereno, 1999).

141. Position of the base of the cervical rib shaft: level with, or higher than the ventral margin of the cervical centrum (0) or located below the ventral margin due to a ventrally extended parapophysis (1) (Wilson & Sereno, 1998).

142. Postzygodiapophyseal lamina in cervical neural arches 4–8: present (0) or absent (1) (Yates, 2003a).

143. Laminae of the cervical neural arches 4–8: well-developed tall laminae (0) or weakly developed low ridges (1) (Wilson & Sereno, 1998).

144. Shape of anterior centrum face in cervical centra: concave (0), flat (1), or convex (2) (modified from Gauthier, 1986). Ordered.

145. Ventral surface of the centra in the cervicodorsal transition: transversely rounded (0) or with longitudinal keels (1) (Rauhut, 2003).

146. Number of vertebrae between cervicodorsal transition and primordial sacral vertebrae: 15–16 (0) or no more than 14 (1) (modified from Wilson & Sereno, 1998).

147. Lateral surfaces of the dorsal centra: with at most vague, shallow depressions (0), with deep fossae that approach the midline (1), or with invasive, sharp-rimmed pleurocoels (2) (Gauthier, 1986). Ordered.

148. Oblique ridge dividing pleural fossa of cervical vertebrae: absent (0) or present (1) (Wilson & Sereno, 1998).

149. Laterally expanded tables at the midlength of the dorsal surface of the neural spines: absent in all vertebrae (0), present on the pectoral vertebrae (1) or present on the pectoral and cervical vertebrae (2) (Yates & Kitching, 2003). Ordered.

150. Dorsal centra: entirely amphicoelous to amphiplatyan (0), first two dorsals are opisthocoelous (1), or cranial half of dorsal column is opisthocoelous (2) (Wilson & Sereno, 1998). Ordered.

151. Shape of the posterior dorsal centra: relatively elongated for their size (0) strongly axially compressed for their size (1) (modified from Novas, 1993).

152. Laminae bounding triangular infradiapophyseal fossae (chonae) on dorsal neural arches: absent (0) or present (1) (Wilson, 1999).

153. Location of parapophysis in first two dorsals: at the anterior end of the centrum (0), or located at the mid-length of the centrum, within the middle chonos (1).

154. Parapophyses of the dorsal column completely shift from the centrum to the neural arch: anterior (0), or posterior (1), to the thirteenth presacral vertebra (Langer, 2004).

155. Orientation of the transverse processes of the dorsal vertebrae: most horizontally directed (0) or all upwardly directed (1) (Upchurch, 1998).

156. Contribution of the paradiapophyseal lamina to the margin of the anterior chonos in mid-dorsal vertebrae: present (0) or prevented by high placement of parapophysis (1).

157. Hyposphenes in the dorsal vertebrae: absent (0), present but less than the height of the neural canal (1), or present and equal to the height of the neural canal (2) (modified from Gauthier, 1986). Ordered.

158. Prezygodiapophyseal lamina and associated anterior triangular fossa (chonos): present on all dorsals (0) or absent in mid-dorsals (1) (Yates, 2003a).

159. Anterior centroparapophyseal lamina in dorsal vertebrae: absent (0) or present (1) (Wilson 2002).

160. Prezygoparapophyseal lamina in dorsal vertebrae: absent (0) or present (1).

161. Accessory lamina dividing posterior chonos from postzygapophysis: absent (0) or present (1).

162. Lateral pneumatic fenestra in middle chonos of middle and posterior dorsal vertebrae opening into neural cavity: absent (0) or present (1) (Wilson & Sereno, 1998).

163. Separation of lateral surfaces of anterior dorsal neural arches under transverse processes: widely spaced (0) or only separated by a thin midline septum (1) (Upchurch *el al.,* 2004).

164. Height of dorsal neural arches, from neurocentral suture to level of zygapophyseal facets: much less than (0), or subequal to or greater than (1), height of centrum.

165. Form of anterior surface of neural arch: simple centroprezygopophyseal ridge (0) or broad anteriorly facing surface bounded laterally by centroprezygopophyseal lamina (1) (Bonaparte, 1999).

166. Shape of posterior dorsal neural canal: subcircular (0) or slit-shaped (1) (Wilson & Sereno, 1998).

167. Height of middle dorsal neural spines: less than the length of the base (0), higher than the length of the base but less than 1.5 times the length of the base (1) or greater than 1.5 times the length of the base (2) (modified from Bonaparte, 1986). Ordered.

168. Shape of anterior dorsal neural spines: lateral margins parallel in anterior view (0) or transversely expanding towards dorsal end (1).

169. Cross-sectional shape of dorsal neural spines: transversely compressed (0), broad and triangular (1), or square-shaped in posterior vertebrae (2) (modified from Bonaparte, 1986).

170. Spinodiapophyseal lamina on dorsal vertebrae: absent (0), present and separated from spinopostzygapophyseal lamina (1) or present and joining spinopostzygapophyseal lamina to create a composite posterolateral spinal lamina (Wilson & Sereno, 1998).

171. Well-developed, sheet-like suprapostzygapophyseal laminae: absent (0), present on at least the caudal dorsal vertebrae (1) (Bonaparte, 1986).

172. Shape of the spinopostzygapophyseal lamina in middle and posterior dorsal vertebrae: singular (0) or bifurcated at its distal end (1) (Wilson, 2002).

173. Shape of posterior margin of middle dorsal neural spines in lateral view: approximately straight (0) or concave with a projecting posterodorsal corner (1) (Yates, 2003b).

174. Transversely expanded plate-like summits of posterior dorsal neural spines: absent (0) or present (1) (Novas, 1993).

175. Last presacral rib: free (0) or fused to vertebra (1).

176. Sacral rib much narrower than the transverse process of the first primordial sacral vertebra (and dorsosacral if present) in dorsal view: absent (0) or present (1) (Yates & Kitching 2003).

177. Number of dorsosacral vertebrae: none (0), one (1), or two (2) (modified from Gauthier, 1986). Ordered.

178. Caudosacral vertebra: absent (0) or present (1) (Galton & Upchurch, 2004).

179. Shape of the iliac articular facets of the first primordial sacral rib: singular (0) or divided into dorsal and ventral facets separated by a non-articulating gap (1).

180. Depth of the iliac articular surface of the primordial sacrals: less than (0), or greater than (1), 0.75 of the depth of the ilium (modified from Novas, 1992 by Yates, 2007).

181. Sacral ribs contributing to the rim of the acetabulum: absent (0) or present (1) (Wilson, 2002).

182. Posterior and anterior expansion of the transverse processes of the first and second primordial sacral vertebrae, respectively, partly roofing the intercostal space: absent (0) or present (1) (Langer, 2004).

183. Length of first caudal centrum: greater than its height (0), or less than its height (1), or highly compressed (dorsoventral height at least twice anteroposterior length) (2) (modified from Yates, 2003a by McPhee & Choiniere, 2017). Ordered.

184. Length of base of the proximal caudal neural spines: less than (0), or greater than (1), half the length of the neural arch (Gauthier, 1986).

185. Position of postzygapophyses in proximal caudal vertebrae: protruding with an interpostzygapophyseal notch visible in dorsal view (0) or placed on either side of the caudal end of the base of the neural spine without any interpostzygapophyseal notch (1) (Yates, 2003a).

186. A hyposphenal ridge on caudal vertebrae: absent (0) or present (1) (Upchurch, 1995).

187. Depth of the bases of the proximal caudal transverse processes: shallow, restricted to the neural arches (0), deep, extending from the centrum to the neural arch (1) (Upchurch, 1998).

188. Position of last caudal vertebra with a protruding transverse process: distal (0), or proximal (1), to caudal 16 (Wilson, 2002).

189. Orientation of posterior margin of proximal caudal neural spines: sloping posterodorsally (0) or vertical (1) (Novas, 1992).

190. Longitudinal ventral sulcus on proximal and middle caudal vertebrae: present (0) or absent (1) (modified from Upchurch , 1995).

191. Length of midcaudal centra: greater than (0), or less than (1), twice the height of their anterior faces (Yates, 2003a).

192. Cross-sectional shape of the distal caudal centra: oval with rounded lateral and ventral sides (0) or square-shaped with flattened lateral and ventral sides (1).

193. Length of distal caudal prezygapophyses: short, not overlapping the preceding centrum by more than a quarter (0) or long and overlapping the preceding the centrum by more than a quarter (Gauthier, 1986).

194. Shape of the terminal caudal vertebrae: unfused, size decreasing toward tip (0) or expanded and fused to form a club-shaped tail (1) (Upchurch, 1995).

195. Length of the longest chevron: is less than (0), or greater than (1), twice the length of the preceding centrum (modified from Yates, 2003a).

196. Anteroventral process on distal chevrons: absent (0) or present (1) (Upchurch, 1995).

197. Mid-caudal chevrons with a ventral slit: absent (0) or present (1) (Upchurch, 1995).

Appendicular

198. Longitudinal ridge on the dorsal surface of the sternal plate: absent (0) or present (1) (Upchurch, 1998).

199. Craniocaudal length of the acromion process of the scapula: less than (0), or greater than (1), 1.5 times the minimum width of the scapula blade (Wilson & Sereno, 1998).

200. Minimum width of the scapula: less than (0), or greater than (1), 20% of its length (Gauthier, 1986).

201. Caudal margin of the acromion process of the scapula: rises from the blade at angle that is less than (0), or greater than (1), 65° from the long axis of the scapula, at its steepest point (modified from Novas, 1992).

202. Width of dorsal expansion of the scapula: less than (0), or equal to (1), the width of the ventral end of the scapula (Pol and Powell, 2007).

203. Flat caudoventrally facing surface on the coracoid between glenoid and coracoid tubercle: absent (0) or present (1) (Yates & Kitching, 2003).

204. Coracoid tubercle: present (0) or absent (1) (modified from Pérez-Moreno *el al.,* 1994).

205. Length of the humerus: less than 55% (0), 55–65% (1), 65–70% (2), or more than 70% (3), of the length of the femur (modified from Gauthier, 1986). Ordered.

206. Shape of the deltopectoral crest: subtriangular (0) or subrectangular (1) (Gauthier, 1986).

207. Length of the deltopectoral crest of the humerus: less than 30% (0), 30–50% (1), or greater than 50% (2), of the length of the humerus (modified from Sereno *el al.,* 1993). Ordered.

208. Shape of the anterolateral margin of the deltopectoral crest of the humerus: straight (0) or strongly sinuous (1) (Yates, 2003a).

209. Rugose pit centrally located on the lateral surface of the deltopectoral crest: absent (0) or present (1).

210. Well-defined fossa on the distal flexor surface of the humerus: present (0) or absent (1) (Yates & Kitching 2003).

211. Transverse width of the distal humerus: is less than (0), or greater than (1), 33% of the length of the humerus (Langer 2004).

212. Shape of the entepicondyle of the distal humerus: rounded process (0) or with a flat distomedially facing surface bounded by a sharp proximal margin (1).

213. Length of the radius: greater than (0), or less than (1), 80% of the humerus (Langer, 2004).

214. Deep radial fossa, bounded by an anterolateral process, on proximal ulna: absent (0), or present but poorly defined (1), or a well-defined recess, deeper than the transverse width of the anterior end of the anterior process (2) (modified from Wilson & Sereno, 1998 by McPhee & Choiniere, 2017). Ordered.

215. Olecranon process on proximal ulna: present (0) or absent (1) (Wilson & Sereno, 1998).

216. Maximum linear dimensions of the ulnare and radiale: exceed that of at least one of the first three distal carpals (0) or are less than any of the distal carpals (1) (Yates, 2003a).

217. Transverse width of the first distal carpal: less than (0), or greater than (1), 120% of the transverse width of the second distal carpal (Sereno, 1999).

218. Sulcus across the medial end of the first distal carpal: absent (0) or present (1).

219. Lateral end of first distal carpal: abuts (0), or overlaps (1), second distal carpal (Yates, 2003a).

220. Second distal carpal: does (0), or does not (1), completely cover the proximal end of the second metacarpal (Yates & Kitching, 2003).

221. Ossification of the fifth distal carpal: present (0) or absent (1).

222. Length of the manus: less than 38% (0), 38–45% (1), or greater than 45% (2), of the humerus + radius (modified from Sereno *el al.,* 1993). Ordered.

223. Shape of metacarpus: flattened to gently curved and spreading (0) or a colonnade of subparallel metacarpals tightly curved into a U-shape (1) (Wilson & Sereno, 1998).

224. Proximal width of first metacarpal: less than (0), or greater than (1), the proximal width of the second metacarpal (modified from Gauthier, 1986).

225. Minimum transverse shaft width of first metacarpal: less than (0), or greater than (1), twice the minimum transverse shaft width of second metacarpal.

226. Proximal end of first metacarpal: flush with other metacarpals (0) or inset into the carpus (1) (Sereno, 1999).

227. Shape of the first metacarpal: proximal width less than 65% (0), 65–80% (1), 80–100% (2), or greater than 100% (3), of its length (modified from Sereno, 1999). Ordered.

228. Strong asymmetry in the lateral and medial distal condyles of the first metacarpal: absent (0) or present (1) (Gauthier, 1986).

229. Deep distal extensor pits on the second and third metacarpals: absent (0) or present (1) (Novas, 1993).

230. Shape of the distal ends of second and third metacarpals: subrectangular in distal view (0) or trapezoidal with flexor rims of distal collateral ligament pits flaring beyond extensor rims (1).

231. Shape of the fifth metacarpal: longer than wide at the proximal end with a flat proximal surface (0) or close to as wide as it is long with a strongly convex proximal articulation surface (1) (Yates, 2003a).

232. Length of the fifth metacarpal: less than (0), or greater than (1), 75% of the length of the third metacarpal (Upchurch, 1998).

233. Length of manual digit one: less than (0), or greater than (1), the length of manual digit two (Yates, 2003a).

234. Ventrolateral twisting of the transverse axis of the distal end of the first phalanx of manual digit one relative to its proximal end: absent (0), present but much less than 60° (1), or 60° (2) (Sereno, 1999). Ordered.

235. Length of the first phalanx of manual digit one: less than (0), or greater than (1), the length of the first metacarpal (Gauthier, 1986).

236. Shape of the proximal articular surface of the first phalanx of manual digit one: rounded (0) or with an embayment on the medial side (1) (modified from Sereno, 1999).

237. Shape of the first phalanx of manual digit one: elongate and subcylindrical (0) or strongly proximodistally compressed and wedge-shaped (1) (Wilson, 2002).

238. Length of the penultimate phalanx of manual digit two: less than (0), or greater than (1), the length of the second metacarpal (Rauhut, 2003).

239. Length of the penultimate phalanx of manual digit three: less than (0), or greater than (1), the length of the third metacarpal (Rauhut, 2003).

240. Shape of non-terminal phalanges of manual digits two and three: longer than wide (0) or as long as wide (1) (Yates, 2003a).

241. Shape of the unguals of manual digits two and three: straight (0), or strongly curved with tips projecting well below flexor margin of proximal articular surface (1) (Sereno *el al.,* 1993).

242. Length of the ungual of manual digit two: greater than the length of the ungual of manual digit one (0), 75–100% of the ungual of manual digit one (1), less than 75% of the ungual of manual digit one (2), or the ungual of manual digit two is absent (3) (modified from Gauthier, 1986). Ordered.

243. Phalangeal formula of manual digits two and three: three and four, respectively (0), or with at least one phalanx missing from each digit (1) (modified from Wilson & Sereno 1998).

244. Phalangeal formula of manual digits four and five: greater than (0), or less than (1), 2–0, respectively (Gauthier 1986).

245. Strongly convex dorsal margin of the ilium: absent (0) or present (1) (Gauthier 1986).

246. Cranial extent of preacetabular process of ilium: does not (0), or does (1), project further forward than cranial end of the pubic peduncle (Yates 2003a).

247. Shape of the preacetabular process: blunt and rectangular (0) or with a pointed, projecting cranioventral corner and a rounded dorsum (1) (modified from Sereno 1999).

248. Depth of the preacetabular process of the ilium: much less than (0), or subequal to (1), the depth of the ilium above the acetabulum (modified from Gauthier 1986).

249. Length of preacetabular process of the ilium: less than (0), or greater than (1), twice its depth.

250. Buttress between preacetabular process and the supraacetabular crest of the ilium: present (0) or absent (1) (Gauthier, 1986).

251. Medial wall of acetabulum: fully closing acetabulum with a triangular ventral process between the pubic and ischial peduncles (0), partially open acetabulum with a straight ventral margin between the peduncles (1), partially open acetabulum with a concave ventral margin between the peduncles (2), or fully open acetabulum with medial ventral margin closely approximating lateral rim of acetabulum (3) (modified from Gauthier 1986). Ordered.

252. Length of the pubic peduncle of the ilium: less than (0), or greater than (1), twice the craniocaudal width of its distal end (Sereno, 1999).

253. Caudally projecting ‘heel’ at the distal end of the ischial peduncle: absent (0) or present (1) (Yates, 2003b).

254. Length of the ischial peduncle of the ilium: similar to pubic peduncle (0), much shorter than pubic peduncle (1), or virtually absent so that the chord connecting the distal end of the pubic peduncle with the ischial articular surface contacts the postacetabular process (2) (Upchurch *el al.,* 2004). Ordered.

255. Length of the postacetabular process of the ilium: between 40% and 100% of the distance between the pubic and ischial peduncles (0), less than 40% of this distance (1), or more than 100% of this distance (2). Unordered.

256. Well developed brevis fossa with sharp margins on the ventral surface of the postacetabular process of the ilium: absent (0) or present (1) (Gauthier, 1986).

257. Anterior end of ventrolateral ridge bounding brevis fossa: not connected to (0), or joining (1) supracetabular crest (1).

258. Shape of the caudal margin of the postacetabular process of the ilium: rounded to bluntly pointed (0), square ended (1), or with a pointed ventral corner and a rounded caudodorsal margin (2) (Yates, 2003b). Unordered.

259. Width of the conjoined pubes: less than (0), or greater than (1), 75% of their length (Cooper, 1984).

260. Pubic tubercle on the lateral surface of the proximal pubis: present (0) or absent (1) (Yates, 2003a).

261. Proximal anterior profile of pubis: anterior margin of pubic apron smoothly confluent with anterior margin of iliac pedicel (0) or iliac pedicel set anterior to the pubic apron creating a prominent inflection in the proximal anterior profile of the pubis (1).

262. Minimum transverse width of the pubic apron: much more than (0), or less than (1), 40% of the width across the iliac peduncles of the ilium.

263. Position of the obturator foramen of the pubis: at least partially occluded by the iliac pedicel (0), or completely visible (1), in anterior view (Galton & Upchurch, 2004).

264. Lateral margins of the pubic apron in anterior view: straight (0) or concave (1) (Yates & Kitching, 2003).

265. Orientation of distal third of the blades of the pubic apron: confluent with the proximal part of the pubic apron (0) or twisted posterolaterally relative to proximal section so that the anterior surface turns to face laterally (1) (Langer, 2004).

266. Orientation of the entire blades of the pubic apron: transverse (0) or twisted posteromedially (1) (Wilson & Sereno, 1998).

267. Craniocaudal expansion of the distal pubis: absent (0), less than 15% (1), or greater than 15% (2), of the length of the pubis (modified from Gauthier, 1986). Ordered.

268. Notch separating posteroventral end of the ischial obturator plate from the ischial shaft: present (0) or absent (1) (Rauhut, 2003).

269. Elongate interischial fenestra: absent (0) or present (1) (Yates, 2003b).

270. Longitudinal dorsolateral sulcus on proximal ischium: absent (0) or present (1) (Yates, 2003a).

271. Shape of distal ischium: broad and plate-like, not distinct from obturator region (0) or with a discrete rod-like distal shaft (1).

272. Length of ischium: less than (0) or greater than (1) that of the pubis (Salgado, Coria & Calvo, 1997).

273. Ischial component of acetabular rim: larger than (0), or equal to (1), the pubic component (Galton & Upchurch, 2004).

274. Shape of the transverse section of the ischial shaft: ovoid to subrectangular (0) or triangular (1) (Sereno, 1999).

275. Orientation of the long axes of the transverse section of the distal ischia: meet at an angle (0) or are coplanar (1) (Wilson & Sereno, 1998).

276. Depth of the transverse section of the ischial shaft: much less than (0) at least as great as (1), the transverse width of the section (Wilson & Sereno, 1998).

277. Distal ischial expansion: absent (0) or present (1) (Holtz, 1994).

278. Transverse width of the conjoined distal ischial expansions: greater than (0), or less than (1), their sagittal depth (Yates, 2003a).

279. Length of the hindlimb: greater than (0), or less than (1), the length of the trunk (Gauthier, 1986).

280. Longitudinal axis of the femur in lateral view: strongly bent with an offset between the proximal and distal axes greater than 15° (0), weakly bent with an offset of less than 10° (1), or straight (2) (Cooper, 1984). Ordered.

281. Shape of the cross-section of the mid-shaft of the femur: subcircular (0) or strongly elliptical (eccentricity > 1.5) with the long axis orientated mediolaterally (1) (Wilson & Sereno, 1998).

282. Angle between the long axis of the femoral head and the transverse axis of the distal femur: about 30° (0) or close to 0° (1) (Carrano, 2000).

283. Shape of femoral head: roughly rectangular in profile with a sharp medial distal corner (0) or roughly hemispherical with no sharp medial distal corner (1). This character only applies to taxa with a medially, or anteromedially protruding femoral head. It does not apply to outgroup taxa (*Euparkeria* or Crurotarsi) with proximally directed femoral heads and is coded as unknown in these taxa.

284. Posterior proximal tubercle on femur: well developed (0) or indistinct to absent (1) (Novas, 1996).

285. Shape of the lesser trochanter: small rounded tubercle (0), proximodistally orientated, elongate ridge (1), or absent (2) (modified from Gauthier, 1986). Unordered.

286. Position of proximal tip of lesser trochanter: level with (0), or distal to (1), the femoral head (Galton & Upchurch, 2004).

287. Projection of the lesser trochanter: just a scar upon the femoral surface (0) or a raised process (1).

288. Transverse ridge extending laterally from the lesser trochanter: absent (0) or present (1) (Rowe, 1989).

289. Height of the lesser trochanter in cross section: less than (0), or at least as high as (1), basal width (modified from Galton, 1990).

290. Position of the lesser trochanter: near the centre of the anterior face (0), or close to the lateral margin (1), of the femoral shaft in anterior view.

291. Visibility of the lesser trochanter in posterior view: not visible (0) or visible (1) (Galton & Upchurch, 2004).

292. Height of the fourth trochanter: low rugose ridge (0) or a tall crest (1) (Gauthier, 1986).

293. Position of the fourth trochanter along the length of the femur: in the proximal half (0) or straddling the midpoint (1) (Galton, 1990).

294. Symmetry of the profile of the fourth trochanter of the femur: subsymmetrical without a sharp distal corner (0) or asymmetrical with a steeper distal slope than the proximal slope and a distinct distal corner (1) or symmetrical, almost rectangular in lateral view with proximal and distal corners approaching an angle of 90 degrees (2) (modified from Langer, 2004).

295. Shape of the profile of the fourth trochanter of the femur: rounded (0) or subrectangular (1).

296. Position of fourth trochanter along the mediolateral axis of the femur: centrally located (0) on the medial margin (1) (Galton, 1990).

297. Extensor depression on anterior surface of the distal end of the femur: absent (0) or present (1) (Molnar, Kurzanov & Dong Zhiming, 1990).

298. Size of the medial condyle of the distal femur: subequal to (0), or larger than (1), the fibular + lateral condyles (modified from Wilson, 2002).

299. Tibia : femur length ratio: greater than 1.0 (0), between 0.6 and 1.0 (1) or less than 0.6 (2) (modified from Gauthier, 1986). Ordered.

300. Orientation of cnemial crest: projects anteriorly to anterolaterally (0) or projecting laterally (1) (Wilson & Sereno, 1998).

301. Paramarginal ridge on lateral surface of cnemial crest: absent (0) or present (1).

302. Position of the tallest point of the cnemial crest: close to the proximal end of the crest (0) or about half-way along the length of the crest, creating an anterodorsally sloping proximal margin of the crest (1).

303. Proximal end of tibia with a flange of bone that contacts the fibula: absent (0) or present (1) (Gauthier, 1986).

304. Position of the posterior end of the fibular condyle on the proximal articular surface tibia: anterior to (0) or level with (1), the posterior margin of proximal articular surface.

305. Shape of the proximal articular surface of the tibia: ovoid, anteroposteriorly longer than transversely wide (0) or subcircular and as wide transversely as anteroposteriorly long (1) (Wilson & Sereno, 1998).

306. Transverse width of the distal tibia: subequal to (0), or greater than (1), its craniocaudal length (Gauthier, 1986).

307. Anteroposterior width of the lateral side of the distal articular surface of the tibia: as wide (0), or narrower than (1), the anteroposterior width of the medial side.

308. Relationship of the posterolateral process of the distal end of the tibia with the fibula: not flaring laterally and not making significant contact with the fibula (0) or flaring laterally and backing the fibula (1).

309. Shape of the distal articular end of the tibia in distal view: ovoid (0) or subrectangular (1).

310. Shape of the anteromedial corner of the distal articular surface of the tibia: forming a right angle (0) or forming an acute angle (1) (Langer, 2004).

311. Position of the lateral margin of descending caudoventral process of the distal end of the tibia: protrudes laterally at least as far as (0), or set well back from (1), the craniolateral corner of the distal tibia (Wilson & Sereno, 1998).

312. A triangular rugose area on the medial side of the fibula: absent (0) or present (1) (Wilson & Sereno, 1998).

313. Transverse width of the midshaft of the fibula: greater than 0.75 (0), between 0.5 and 0.75 (1), or less than 0.5 (2), of the transverse width of the midshaft of the tibia (Langer, 2004). Ordered.

314. Position of fibula trochanter: on anterior surface of fibula (0), laterally facing (1), or anteriorly facing but with strong lateral bulge (2) (modified from Wilson & Sereno, 1998).

315. Depth of the medial end of the astragalar body in cranial view: roughly equal to the lateral end (0) or much shallower creating a wedge-shaped astragalar body (1) (Wilson & Sereno, 1998).

316. Shape of the posteromedial margin of the astragalus in dorsal view: forming a moderately sharp corner of a subrectangular astragalus (0) or evenly rounded without formation of a caudomedial corner (1) (Wilson & Sereno, 1998).

317. Dorsally facing horizontal shelf forming part of the fibular facet of the astragalus: present (0) or absent with a largely vertical fibular facet (1) (Sereno, 1999).

318. Pyramidal dorsal process on the posteromedial corner of the astragalus: absent (0) or present (1).

319. Shape of the ascending process of the astragalus: anteroposteriorly deeper than transversely wide (0) or transversely wider than anteroposteriorly deep (1).

320. Posterior extent of ascending process of the astragalus: well anterior to (0), or close to the posterior margin of (1), the astragalus (Wilson and Sereno, 1998).

321. Sharp medial margin around the depression posterior to the ascending process of the astragalus: absent (0) or present (1) (Novas 1996).

322. Buttress dividing posterior fossa of astragalus and supporting ascending process: absent (0) or present (1) (Wilson & Sereno, 1998).

323. Vascular foramina set in a fossa at the base of the ascending process of the astragalus: present (0) or absent (1) (Wilson & Sereno, 1998).

324. Transverse width of the calcaneum: greater than (0), or less than (1), 30% of the transverse width of the astragalus (Yates & Kitching, 2003).

325. Lateral surface of calcaneum: simple (0) or with a fossa (1).

326. Medial peg of calcaneum fitting into astragalus: present, even if rudimentary (0) or absent (1) (Sereno *el al.,* 1993).

327. Calcaneal tuber: large and well developed (0) or highly reduced to absent (1).

328. Shape of posteromedial heel of distal tarsal four (lateral distal tarsal): proximodistally deepest part of the bone (0) or no deeper than the rest of the bone (1) (Sereno *el al.,* 1993).

329. Shape of posteromedial process of distal tarsal four in proximal view: rounded (0) or pointed (1) (Langer, 2004).

330. Ossified distal tarsals: present (0) or absent (1) (Gauthier, 1986).

331. Proximal width of the first metatarsal: is less than (0), or at least as great as (1), the proximal width of the second metatarsal (modified from Wilson & Sereno, 1998).

332. Orientation of proximal articular surface of metatarsal one: horizontal (0) or sloping proximolaterally relative to the long axis of the bone (1) (Wilson, 2002).

333. Orientation of the transverse axis of the distal end of metatarsal one: horizontal (0) or angled proximomedially (1) (Wilson, 2002).

334. Shape of the medial margin of the proximal surface of the second metatarsal: straight (0) or concave (1) (modified from Sereno, 1999).

335. Shape of the lateral margin of the proximal surface of the second metatarsal: straight (0) or concave (1) (modified from Sereno, 1999).

336. Length of the third metatarsal: greater than (0), or less than (1), 40% of the length of the tibia (Gauthier, 1986).

337. Minimum transverse shaft diameters of third and fourth metatarsals: greater than (0), or less than (1), 60% of the minimum tansverse shaft diameter of the second metatarsal (Wilson & Sereno, 1998).

338. Transverse width of the proximal end of the fourth metatarsal: less than (0), or at least (1), twice the anteroposterior depth of the proximal end (modified from Sereno, 1999).

339. Transverse width of the proximal end of the fifth metatarsal: less than 25% (0), between 30% and 49% (1), or greater than 50% (2), of the length of the fifth metatarsal (modified from Sereno, 1999). Ordered.

340. Transverse width of distal articular surface of metatarsal four in distal view: greater (0), or less than (1), anteroposterior depth (Sereno, 1999).

341. Pedal digit five: reduced, non-weight bearing (0) or large (fifth metatarsal at least 70% of fourth metatarsal), robust and weight bearing (1) (Wilson & Sereno, 1998).

342. Length of non-terminal pedal phalanges: all longer than wide (0), proximalmost phalanges longer than wide while more distal phalanges are as wide as long (1), or all non-terminal phalanges are as wide, if not wider, than long (2) modified from Wilson & Sereno, 1998). Ordered.

343. Length of the first phalanx of pedal digit one: greater than (0), or less than (1), the length of the ungual of pedal digit one (Yates & Kitching, 2003).

344. Length of the ungual of pedal digit one: less than at least some non-terminal phalanges (0) or longer than all non-terminal phalanges (1) or longer than first metatarsal (2). Ordered. (modified from Yates, 2007).

345. Shape of the ungual of pedal digit one: shallow, pointed, with convex sides and a broad ventral surface (0) or deep, abruptly tapering, with flattened sides and a narrow ventral surface (1) (Wilson & Sereno, 1998).

346. Shape of proximal articular surface of pedal unguals: proximally facing, visible on medial and lateral sides (0) or proximomedially facing and visible only in medial view, causing medial deflection of pedal unguals in articulation (1) (Wilson & Sereno, 1998).

347. Penultimate phalanges of pedal digits two and three: well-developed (0) or reduced disc-shaped elements if they are ossified at all (1) (Wilson & Sereno, 1998).

348. Shape of the unguals of pedal digits two and three: dorsoventrally deep with a proximal articulating surface that is at least as deep as it is wide (0) or dorsoventrally flattened with a proximal articulating surface that is wider than deep (1) (Wilson & Sereno, 1998).

349. Length of the ungual of pedal digit two: greater than (0), between 90% and 100% of (1), or less than 90% of (2), the length of the ungual of pedal digit one (modified from Gauthier, 1986). Ordered.

350. Size of the ungual of pedal digit three: greater than (0), or less than (1), 85% of the ungual of pedal digit two in all linear dimensions (Yates, 2003a).

351. Number of phalanges in pedal digit four: four (0) or fewer than four (1) (Gauthier 1986).

352. Phalanges of pedal digit five: present (0) or absent (1) (Gauthier, 1986).

353. Femoral length: less than 200 mm (0), between 200 and 399 mm (1), between 400 and 599 mm (2), between 600 and 799 mm (3), between 800 and 1000 mm (4), or greater than 1000 mm (modified from Yates, 2004). Ordered.

354. Lateral extent of ventrolateral flange on plantar surface of MT II in proximal aspect: similar in development to ventromedial flange (0), well−developed, extending further laterally than ventromedial flange extends medially (1) (Smith and Pol, 2007).

355. Distal articular surface of astragalus: relatively flat or weakly convex (0), extremely convex and “roller−shaped” (1) (Smith and Pol, 2007).

356. Distal surface of tibiofibular crest: as deep anteroposteriorly as wide mediolaterally or deeper (0), wider mediolaterally than deep anteroposteriorly (1) (Smith and Pol, 2007).

357. Well−developed facet on proximolateral corner of plantar ventrolateral flange of

MT II for articulation with medial distal tarsal: absent (0), present (1) (Smith and Pol, 2007).

358. Proximal outline of metatarsal III: subtriangular with acute or rounded posterior border (0), subtrapezoidal, with posterior border broadly exposed in plantar view (1).

359. Angle formed by the anterior and anteromedial borders of metatarsal IV: obtuse (0), right angle, or acute (1) (Smith and Pol, 2007).

360. Well−developed tibiofibular crest on distal femur: absent (0), present (1) (Smith and Pol, 2007).

361. Shaft of metatarsal I: closely appressed to metatarsal II throughout its length (0), or only closely appressed proximally, with a space between metatarsals I and II distally (1) (Smith and Pol, 2007).

362. Posterior margin of astragalus: straight (0), convex (1) (Otero & Pol, 2013).

363. Ventromedial ridge of scapula: absent (0), present (1) (Otero & Pol, 2013).

364. Mediolateral surface of distal astragalus straight (0), concave (1), or convex (2). (Otero & Pol, 2013). Unordered.

365. Anterior fossa on the proximal region of the pubic apron: absent (0), present (1). (Apaldetti *el al.,* 2013).

366. Proximal end of the tibia with a transverse/anteroposterior length ratio: narrow

(ratio less than 0.7) (0), or broad (more than 0.7) (1). (Apaldetti *el al.,* 2013).

367. Caudodistal tubercle of the radius: absent (0), present (1). (Otero et al., 2015).

368. Biceps tubercle of the radius: absent (0), present (1). (Otero et al., 2015).

369. Ventromedial margin of first metacarpal: poorly concave (0), deeply concave (1). (Otero et al., 2015).

370. Length of first phalanx of manual digit 1: much greater than (0), subequal or equal to (1), or much less than (2) its mediolateral width at proximal end. (Otero et al., 2015).

371. Muscle origin areas (*Mm. flexor tibialis* and *iliotibialis*) on the posterior portion of the postacetabular process of the ilium: smooth or as a rectangular rugosity (0), strong trapezoidal rugosity extended along the whole height of the posterior third of the process (1). (Ezcurra, 2010).

372. Supraacetabular crest of ilium: not extended along the pubic peduncle or only at the base of the peduncle (0), extended along the pubic peduncle as a faint ridge (1), extended along the entire pubic peduncle and contacts the distal end as a well developed crest (2). (Ezcurra 2010).

373. Subnarial gap (i.e. posterior part of premaxillary alveolar margin edentelous, resulting in an interruption of the upper tooth row): absent (0), present (1). (Gauthier 1986).

374. Alveolar margin of anterior-most maxilla: relatively straight or slightly convex (0), strongly but gradually upturned from an extension of more than three teeth along the alveolar margin and orienting the first maxillary alveolus anteroventrally (1), sharply mediodorsally upturned in the anterior-most tip of the maxilla and orienting the first maxillary alveolus anteroventrally (2). (Rowe 1989, Tykoski 1998, Ezcurra and Novas 2007b).

375. Anterior margin of maxillary antorbital fossa: rounded or pointed (0), squared (1). (Rauhut 2003).

376. Dorsoventrally compressed ridge on lateral surface of maxilla, forming the ventral border of the antorbital fossa (alveolar ridge): absent (0), present (1). (Rowe and Gauthier 1990).

377. Exposition of the lacrimal antorbital fossa in lateral view: lateral lamina of bone covering most of the bone, with antorbital fossa exposed only at the distal end of the vertical process (0), lateral lamina of bone only interrupting the fossa near the proximal end of the ventral ramus and ventrally restricted to posterior margin of the ventral ramus, with antorbital fossa laterally exposed along most of the lacrimal (1). (Modified from Ezcurra and Novas 2007b).

378. Medial distal condyle of metacarpal I: of the same size (0) or dorsoventrally smaller (1) than the lateral distal condyle. (Ezcurra 2010).

379. Metacarpals IV and V ventral to metacarpals I-III: absent (0), present (1). (Sereno 1993)

380. Supraacetabular crest of ilium: present as a weakly developed ridge (0), present as a well developed raised shelf (1), flares lateroventrally to form a hood-like overhang that hides anterodorsal half of acetabulum in lateral view (2). (Langer 2004, Tykoski 2005).

381. Iliac blade in dorsal view: straight or slightly laterally curved along the whole of its anteroposterior extension (0), strongly laterally curved, with a deeply concave lateral border (1). (Ezcurra 2010).

382. Pubic shaft: posteriorly bowed (0), nearly straight (1). (Sereno 1999, Ezcurra and Novas 2007b).

383. Femoral head: weakly developed and slightly inturned, oriented at more than 120◦ from the main axis of the femoral shaft (0), strongly inturned, oriented at less than 120◦ from the main axis of the femoral head, and distinctively separated from the shaft by a well developed femoral neck (1). (Ezcurra 2006).

384. Posterolateral corner of the distal end of the tibia: convex (0), concave (1). (Ezcurra 2006)

385. Ungual of pedal digit II: shorter or equal in length to (0), or longer than (1), pedal phalanx II-2. (Ezcurra 2010).

386. Distal outline of ischium: roughly semicircular (0), sub-triangular (1). (Sereno 1999).

387. Orientation of the pubic shaft: anteroventral (0), ventral, almost perpendicular to the longitudinal axis of the ilium, or slightly posteroventral (1), strongly posteroventral, with the pubic shaft parallel to the ischial shaft (2). (Modified from Sereno 1986 and Novas 1992).

388. Femoral distal transverse width: equal or lesser (0), greater (1) than 1·4 times its largest anteroposterior depth across the fibular condyle. (Novas et al. 2011).

389. Astragalus with medial condyle anteroposterior depth: less (0), equal or more (1) than 1.6 times the depth of the lateral condyle. (Novas et al. 2011)

390. Prezygadiapophyseal laminae on anterior caudal vertebrae: absent (0), present (1). (McPhee et al. 2015).

391. ‘Weaponized’ dermal spikes on tail: absent (0), present (1). (McPhee et al. 2015).

392. Shape of the humeral head: weakly developed, rounded in anterior-posterior view but minimally expanded perpendicular to the latter axis (0), flat in anterior-posterior view with only a slightly expanded lateral component (1), domed, being convex/hemispherical in anterior-posterior view with a strong lateral incursion onto the humeral shaft (2). (McPhee et al., 2015).

393. Size of first metatarsal: maximum proximal breadth less than 0.4 times its proximodistal length (0), maximum proximal breadth between 0.4 and 0.7 times its proximodistal length (1), maximum proximal breadth greater than 0.7 times its proximodistal length (2). (McPhee et al., 2015). Ordered.

394. Laminae/ridges extending from the basipterygoid process onto the parasphenoid rostrum: extend parallel until they fade into the ventral margin of the cultriform process (0), or converge anteromedially on the ventral surface of the cultriform process (1) (Bronzati and Rauhut, 2017).

395. Angle between basipterygoid process and cultriform process of the parabasisphenoid: < 90° (0), 90° (1), or > 90° (2) (Bronzati and Rauhut 2017). Unordered.

396. Length of the basisphenoid (from the basipterygoid process to the basisphenoidal component of the basal tubera) in relation to the length of the basioccipital (from the basioccipital component of the basal tubera to posterior limit of the condyle): longer or equal (0), or shorter (1). (Bronzati and Rauhut 2017).

397. Notch in the posterodorsal margin of the lateral portion of the parabasisphenoid: absent (0), or present (1). (Bronzati and Rauhut 2017).

398. Number of foramina in the otoccipital between the exoccipital pillar (excluding the foramina for the hypoglossal nerve) posteriorly and fenestra ovalis anteriorly: one (0), or two (1). (Bronzati and Rauhut 2017).

399. Unossified gap between the basioccipital and basisphenoidal component of the basal tubera and ventral ramus of the opistothic: absent (0), or present (1). (Bronzati and Rauhut 2017).

400. Otosphenoidal crest: low and not projecting posterolaterally (i.e. does not cover the fenestra ovalis with the braincase in lateral view) (0), or developed as a lamina projecting posterolaterally (i.e. cover the fenestra ovalis with the braincase in lateral view) (1). (Bronzati et al. 2018).

401. Frontal, anteroposterior length: approximately twice (0), or less than minimum transverse breadth (1). (Wilson 2002).

402. Parietal, distance separating supratemporal fenestrae: less than (0), or twice the long axis of supratemporal fenestra (1). (Wilson 2002).

403. Supratemporal region, anteroposterior length: temporal bar longer (0), or shorter anteroposteriorly than transversely (1). (Wilson 2002).

404. Orientation of the anterior to middle cervical postzygapophyses: planar (minimally offset) with respect to the prezygapophyses (0), or dorsally raised roughly 20° relative to the coronal plane (1), or dorsally raised at least 30° or more relative to the coronal plane (2). (McPhee and Choiniere 2017). Ordered.

405. Dorsoventral height of the lacrimal ramus (ventral process) of the prefrontal: more than 0.5 times that of the jugal ramus (ventral ramus) of lacrimal (0), or less than 0.5 times that of the jugal ramus (ventral ramus) of the lacrimal (1) (Chapelle and Choiniere 2018).

406. Distal end of frontal ramus of postorbital: single rounded process (0), or forked into parietal and frontal processes with a distinct concave notch between them (1) (Chapelle and Choiniere 2018).

407. Anterior portion of supratemporal fossa on posterior end of dorsal surface of frontal: weakly excavated (0), or deeply excavated forming a scarp-like margin (1) (Chapelle and Choiniere 2018).

408. Squamosal-quadratojugal contact: point contact or a dorsoventrally oriented short contact (0), or broad contact anteroposteriorly or obliquely oriented (1) (Chapelle and Choiniere, 2018).

409. Angle between quadratojugal and pterygoid rami of quadrate: acute angle, between 0 and 30 degrees (0), or between 30 degrees and 90 degrees (1), or greater tan 90 degrees (2) (Chapelle and Choiniere 2018). Ordered.

410. Ventral extent of quadrate condyles: both condyles extend to the same ventral level (0), or medial condyle extends farther ventrally (1), or lateral condyle extends farther ventrally (2) (Chapelle and Choiniere 2018).

411. Shape of anteroventral portion of prootic: rectangular, anteroposteriorly longer than dorsoventrally high (0), or bulbous, almost as anteroposteriorly long as dorsoventrally high (1) (Chapelle and Choiniere 2018).

412. Angle separating the long axes of the basiperygoid processes in anterior view: 60 degrees or less (0), or more than 60 degrees (1) (Chapelle and Choiniere 2018).

413. Length of postorbital ramus of laterosphenoid: short, subequal to supraoccipital ramus (0), or  more than 10% longer than supraoccipital ramus (1) (Chapelle and Choiniere 2018).

414. Orientation of postorbital ramus of laterosphenoid: extends laterally (0), or extends anterodorsally (1) (Chapelle and Choiniere 2018).

415. Orientation of frontal ramus of laterosphenoid: extends medially (0), or extends anteromedially (1) (Chapelle and Choiniere 2018).

416. Distal end of jugal ramus of ectopterygoid: tapering (0), or broad subrectangular distal end (1), or expanded anteroposteriorly, forming a T-shaped dorsal and ventral profile (2) (Chapelle and Choiniere 2018).

417. Serrations on premaxillary teeth: present (0), absent (1).

418. Growth marks in long bones: present in the whole cortex (0), or growth marks absent or only formed in the outer cortex (1). (Cerda et al. 2017)

419. Relative abundance of woven fibered WFB versus parallel fibered bone PFB in the primary compact bone: PFB > WFB (0), WFB > PFB (1). (Cerda et al. 2017).

**6** **-** **References**

Apaldetti, C., Pol, D. & Yates, A. M. The postcranial anatomy of *Coloradisaurus brevis* (Dinosauria: Sauropodomorpha) from the Late Triassic of Argentina and its phylogenetic implications. *Palaeontology* **56**, 277–301 (2013).

Bordy, E. M. *et al*. A chronostratigraphic framework for the upper Stormberg Group: implications for the Triassic-Jurassic boundary in southern Africa. *Earth Sci. Rev*. **203**, 103120; 10.1016/j.earscirev.2020.103120 (2020).

Bronzati, M. & Rauhut, O.W. Braincase redescription of *Efraasia minor* Huene, 1908 (Dinosauria: Sauropodomorpha) from the Late Triassic of Germany, with comments on the evolution of the sauropodomorph braincase. *Zool. J. Linn. Soc.* **182**, 173–224 (2017).

Bronzati, M., Benson, R. B. J. & Rauhut O. W. M. Rapid transformation in the braincase of sauropod dinosaurs: integrated evolution of the braincase and neck in early sauropods? *Palaeontology* **61**, 289–302 (2018).

Campione, N. E. MASSTIMATE: Body Mass Estimation Equations for Vertebrates. R package version 2.0-1. <https://CRAN.R-project.org/package=MASSTIMATE> (2020).

Campione, N. E., Evans, D. C., Brown, C. M. & Carrano, M. T. Body mass estimation in non-avian bipeds using a theoretical conversion to quadruped stylopodial proportions. *Meth. Ecol. Evol.* **5**, 913–923 (2014).

Cerda, I. A. *et al.* Novel insight into the origin of the growth dynamics of sauropod dinosaurs. PLoS ONE **12**, e0179707; 10.1371/journal.pone.0179707 (2017).

Chapelle K. E. & Choiniere, J. N. A revised cranial description of *Massospondylus carinatus* Owen (Dinosauria: Sauropodomorpha) based on computed tomographic scans and a review of cranial characters for basal Sauropodomorpha. *PeerJ* **6**, e4224; 10.7717/peerj.4224 (2018).

Chapelle, K., E. *et al*. A quantitative method for inferring locomotory shifts in amniotes during ontogeny, its application to dinosaurs and its bearing on the evolution of posture. *Palaeontology* **63**, 229–242 (2020)

Elliot, D., Larsen, D., Fanning, C., Fleming, T. & Vervoort, J. The Lower Jurassic Hanson Formation of the Transantarctic Mountains: implications for the Antarctic sector of the Gondwana plate margin. *Geol. Mag.* **154**, 777–803 (2017).

Ezcurra, M. D. A new early dinosaur (Saurischia: Sauropodomorpha) from the Late Triassic of Argentina: a reassessment of dinosaur origin and phylogeny. *J. Syst. Paleontol.* **8**, 371–425 (2010).

Ezcurra, M. D. & Butler, R. J. The rise of the ruling reptiles and ecosystem recovery from the Permo-Triassic mass extinction. *Proc. R. Soc. B: Biol. Sci.* **285**, 20180361 (2018).

Flannery Sutherland, J. T., Moon, B. C., Stubbs, T. L. & Benton, M. J. Does exceptional preservation distort our view of disparity in the fossil record? *Proc. R. Soc. B* **286**, 20190091 (2019).

Langer, M. C., McPhee, B. W., Marsola, J. C. A., Roberto-da-Silva, L. & Cabreira, S. F. Anatomy of the dinosaur *Pampadromaeus barberenai* (Saurischia—Sauropodomorpha) from the Late Triassic Santa Maria Formation of Southern Brazil. *PloS One* **14** (2), e0212543 (2019).

Lehmann, O. E. & Ezcurra, M. D. Desafíos en los análisis de disparidad con taxones incompletos: el caso de la Máxima Distancia Observada Rescalada (MORD). *RCAPA, Libro de Resúmenes*, 125–126 (2020).

Lehmann, O. E., Ezcurra, M. D., Butler, R. J. & Lloyd, G. T. Biases with the Generalized Euclidean Distance measure in disparity analyses with high levels of missing data. *Palaeontology* **62**, 837–849 (2019).

Lloyd, G. T. Estimating morphological diversity and tempo with discrete character-taxon matrices: implementation, challenges, progress, and future directions. *Biol. J. Linn. Soc.* **118**, 131–151 (2016).

McPhee, B. W. & Choiniere, J. N. The osteology of *Pulanesaura eocollum*: implications for the inclusivity of Sauropoda (Dinosauria). *Zool. J. Linn. Soc.* **182**, 830–861 (2017).

McPhee, B. W., Bordy, E. M., Sciscio, L. & Choiniere, J. N. The sauropodomorph biostratigraphy of the Elliot Formation of southern Africa: Tracking the evolution of Sauropodomorpha across the Triassic-Jurassic boundary. *Acta Palaeontol. Pol.* **62**, 441–465 (2017).

McPhee, B. W., Benson, R. B. J., Botha-Brink, J., Bordy, E. M. & Choiniere, J. N. A giant dinosaur from the earliest Jurassic of South Africa and the transition to quadrupedality in Early Sauropodomorphs. *Curr. Biol.* **8**, 15179; 10.1016/j.cub.2018.07.063 (2018).

McPhee, B. W., Bonnan, M. F., Yates, A. M., Neveling, J. & Choiniere, J. N. A new basal sauropod from the pre-Toarcian Jurassic of South Africa: evidence of niche-partitioning at the sauropodomorph–sauropod boundary? *Sci. Rep.* **5**, 13224; 10.1038/srep13224 (2015).

Novas, F. E., Ezcurra, M. D., Chatterjee, S. & Kutty, T. S. New dinosaur species from the Upper Triassic Upper Maleri and Lower Dharmaram formations of Central India. *Earth Environ. Sci. Trans. R. Soc. Edinb.* **101**, 333–349 (2011).

Novas, F., *et al*. Review of the fossil record of early dinosaurs from South America, and its phylogenetic implications. *J. S. Am. Earth Sci.* **110**, 103341; [10.1016/j.jsames.2021.103341](https://doi.org/10.1016/j.jsames.2021.103341) (2021).

Otero, A. & Pol, D. Postcranial anatomy and phylogenetic relationships of *Mussaurus patagonicus* (Dinosauria, Sauropodomorpha). *J. Vertebr. Paleontol.* **33**, 1138–1168 (2013).

Otero, A., Krupandan, E., Pol, D., Chinsamy, A. & Choiniere, J. N. A new basal sauropodiform from South Africa and the phylogenetic relationships of basal sauropodomorphs. *Zool. J. Linn. Soc.* **174**, 589–634 (2015).

Pol, D., Otero, A., Apaldetti, C. & Martínez, R. N. Triassic sauropodomorph dinosaurs from South America and the origin and diversification of dinosaur dominated herbivorous faunas. *J. of South Am. Ear. Sc.* **107**, 103145; 10.1016/j.jsames.2020.103145 (2021).

Pradelli, L. A., Leardi, J. M. & Ezcurra, M. D. Body size disparity of the Archosauromorph reptiles during the first 90 million years of their evolution. *Ameghiniana*, 3441; 10.5710/AMGH.16.09.2021.3441 (2021).

Smith, N., D. & Pol, D. Anatomy of a basal sauropodomorph dinosaur from the Early Jurassic Hanson Formation of Antarctica. *Acta Palaeontol. Pol.* **52**, 657–674 (2007).

Yates, A., M. The first complete skull of the Triassic dinosaur *Melanorosaurus* Haughton (Sauropodomorpha: Anchisauria). *Spec. Pap. Palaeont.* **77**, 9–5 (2007).
