# Supplementary material for: Sauropodomorph evolution across the Triassic–Jurassic boundary: body size, locomotion, and their influence on morphological disparity
Source: Sci Rep. 2021 Nov 18;11:22534. doi: 10.1038/s41598-021-01120-w (PMC8602272; doi:10.1038/s41598-021-01120-w)
Supplement: Supplementary file 3 — Supplementary Information 3. [file 41598_2021_1120_MOESM3_ESM.docx]

FAD LAD

Euparkeria 247.2 242

Crurotarsi 247.2 145.5

Marasuchus 236.1 233.7

Ornithischia 231.4 145.5

Agnosphitys 208.5 201.3

Silesaurus 236 227

Neotheropoda 227 145.5

Staurikosaurus 235 221.5

Chindesaurus 227 208.5

Herrerasaurus 231.4 225.9

Eoraptor 231.4 225.9

Saturnalia 233.2 225.4

Panphagia 231.4 225.9

Chromogisaurus 231.4 225.9

Buriolestes 233.2 225.4

Guaibasaurus 225.4 220

Pampadromaeus 233.2 225.4

Bagualosaurus 233.2 225.4

Nambalia 225.4 208.5

Thecodontosaurus 208.5 201.3

Pantydraco 208.5 201.3

Efraasia 215.56 212

Ruehleia 225.4 208.5

Jaklapalisaurus 225.4 208.5

Macrocollum 225.4 220

Unaysaurus 225.4 220

Plateosaurus_ingens 212 205.6

Plateosaurus_gracilis 225.4 208.5

Plateosaurus_engelhardti 225.4 201.3

Pradhania 199.3 190.8

Glacialisaurus 199 182.7

Coloradisaurus 220 213

Yunnanosaurus_huangi 201.3 190.8

Lufengosaurus 201.3 190.8

Xixipiosaurus 201.3 190.8

Massospondylus_carinatus 201.3 187.5

Adeopapposaurus 201.3 190.8

Leyesaurus 201.3 190.8

Plateosauravus 219.6 202.3

Riojasaurus 220 213

Eucnemesaurus_fortis 219.6 202.3

Eucnemesaurus_entaxonis 219.6 202.3

Seitaad 190.8 182.7

Anchisaurus 201.3 190.8

Chuxiongosaurus 201.3 190.8

Jingshanosaurus 201.3 190.8

Xingxiulong 201.3 190.8

Sarahsaurus 199.3 183.7

Yizhousaurus 201.3 190.8

Kholumolumo 219.6 202.3

Mussaurus 192.7 192.6

Leonerasaurus 189 188.8

Sefapanosaurus 219.6 202.3

Aardonyx 201.3 187.5

Meroktenos 219.6 202.3

NMRQ3314 201.3 187.5

NMQR1551 219.6 202.3

Ingentia 213 201.3

Lessemsaurus 220 213

Antetonitrus 201.3 187.5

Ledumahadi 201.3 187.5

Blikanasaurus 219.6 202.3

Camelotia 208.5 201.3

Pulanesaura 201.3 187.5

Gongxianosaurus 182.7 174.1

Isanosaurus 208.5 174.1

Tazoudasaurus 190.8 174.1

Vulcanodon 199.3 182.7

Shunosaurus 161 157

Spinophorosaurus 174.1 163.5

Patagosaurus 178.7 178.1

Barapasaurus 190.8 170.3

Cetiosaurus 170.3 163.5

Omeisaurus 161 157

Mamenchisaurus 163.5 152.1

Neosauropoda 166.1 145.5

Ngwevu 201.3 187.5

Irisosaurus 201.3 190.8

Schleitheimia 213 208.5
