# Supplementary material for: Sauropodomorph evolution across the Triassic–Jurassic boundary: body size, locomotion, and their influence on morphological disparity
Source: Sci Rep. 2021 Nov 18;11:22534. doi: 10.1038/s41598-021-01120-w (PMC8602272; doi:10.1038/s41598-021-01120-w)
Supplement: Supplementary file 4 — Supplementary Information 4. [file 41598_2021_1120_MOESM4_ESM.docx]

MPDBootstrap <- function (DistanceMatrix,

Bins = NULL,

Replicates = 1001,

ConfidenceLevel = 0.95) {

if (is.null(Bins)) {

SampleMean <- mean(as.dist(DistanceMatrix$distance_matrix), na.rm = TRUE)

r <- replicate(n = Replicates,

expr = {sample(x = as.dist(DistanceMatrix$distance_matrix),

size = length(as.dist(DistanceMatrix$distance_matrix)),

replace = TRUE)})

DeltaStars <- colMeans(r, na.rm = TRUE) - SampleMean

rs <- sort(SampleMean - quantile(DeltaStars,

c((1 - ConfidenceLevel) / 2,

1 - ((1 - ConfidenceLevel) / 2)),

names = FALSE))

res <- data.frame("Bin" = "All",

"MPD" = SampleMean,

"LowerLimit" = rs[1],

"UpperLimit" = rs[2])

return(res)

} else {

if (any(unlist(lapply(Bins, length), use.names = FALSE) <= 2)) {

stop("At least one bin contains two or fewer taxa. MPD cannot be calculated.")

}

res <- data.frame("Bin" = names(Bins),

"MPD" = rep(0, length(Bins)),

"LowerLimit" = rep(0, length(Bins)),

"UpperLimit" = rep(0, length(Bins)))

for (i in seq_along(Bins)) {

SampleMean <- mean(as.dist(DistanceMatrix$distance_matrix[Bins[[i]], Bins[[i]]]),

na.rm = TRUE)

r <- replicate(n = Replicates,

expr = {sample(x = as.dist(DistanceMatrix$distance_matrix[Bins[[i]], Bins[[i]]]),

size = length(as.dist(DistanceMatrix$distance_matrix[Bins[[i]], Bins[[i]]])),

replace = TRUE)})

DeltaStars <- colMeans(r, na.rm = TRUE) - SampleMean

rs <- sort(SampleMean - quantile(DeltaStars,

c((1 - ConfidenceLevel) / 2,

1 - ((1 - ConfidenceLevel) / 2)),

names = FALSE,

na.rm = TRUE))

res[i, "MPD"] <- SampleMean

res[i, "LowerLimit"] <- rs[1]

res[i, "UpperLimit"] <- rs[2]

}

return(res)

}

}

WMPDBootstrap <- function (DistanceMatrix,

Bins = NULL,

Replicates = 1001,

ConfidenceLevel = 0.95) {

if (is.null(Bins)) {

d <- as.dist(DistanceMatrix$distance_matrix)

cmp <- as.dist(DistanceMatrix$comparable_character_matrix)

sn <- replicate(n = Replicates, expr = {sample(x = seq_along(d),

size = length(d),

replace = TRUE)})

SampleWMPD <- (sum(d * cmp, na.rm = TRUE)) / sum(cmp)

DeltaStars <- apply(X = sn,

MARGIN = 2,

FUN = function(x) {((sum(d[x] * cmp[x], na.rm = TRUE)) /

sum(cmp[x])) - SampleWMPD})

rs <- sort(SampleWMPD - quantile(DeltaStars,

c((1 - ConfidenceLevel) / 2,

1 - ((1 - ConfidenceLevel) / 2)),

names = FALSE))

res <- data.frame("Bin" = "All",

"WMPD" = SampleWMPD,

"LowerLimit" = rs[1],

"UpperLimit" = rs[2])

return(res)

} else {

if (any(unlist(lapply(Bins, length), use.names = FALSE) <= 2)) {

stop("At least one bin contains two or fewer taxa. WMPD cannot be calculated.")

}

res <- data.frame("Bin" = names(Bins),

"WMPD" = rep(0, length(Bins)),

"LowerLimit" = rep(0, length(Bins)),

"UpperLimit" = rep(0, length(Bins)))

for (i in seq_along(Bins)) {

d <- as.dist(DistanceMatrix$distance_matrix[Bins[[i]], Bins[[i]]])

cmp <- as.dist(DistanceMatrix$comparable_character_matrix[Bins[[i]], Bins[[i]]])

sn <- replicate(n = Replicates, expr = {sample(x = seq_along(d),

size = length(d),

replace = TRUE)})

SampleWMPD <- (sum(d * cmp, na.rm = TRUE)) / sum(cmp)

DeltaStars <- apply(X = sn,

MARGIN = 2,

FUN = function(x) {((sum(d[x] * cmp[x], na.rm = TRUE)) /

sum(cmp[x])) - SampleWMPD})

rs <- sort(SampleWMPD - quantile(DeltaStars,

c((1 - ConfidenceLevel) / 2,

1 - ((1 - ConfidenceLevel) / 2)),

names = FALSE,

na.rm = TRUE))

res[i, "WMPD"] <- SampleWMPD

res[i, "LowerLimit"] <- rs[1]

res[i, "UpperLimit"] <- rs[2]

}

return(res)

}

}

get_dist_to_centroid <- function(x) {

mtx <- rbind(rep(0, times = ncol(x)), x)

dists <- dist(mtx, method = "euclidean")

res <- as.matrix(dists)[1, -1]

return(res)

}

sov_bootstrap <- function (Vectors,

Bins = NULL,

Replicates = 1001,

ConfidenceLevel = 0.95) {

if (is.null(Bins)) {

sn <- replicate(n = Replicates, expr = {sample(x = seq_len(nrow(Vectors)),

size = nrow(Vectors),

replace = TRUE)})

SampleSoV <- sum(apply(X = Vectors, MARGIN = 2, FUN = var))

DeltaStars <- apply(X = sn,

MARGIN = 2,

FUN = function(x) {sum(apply(Vectors[x, ], 2, var)) -

SampleSoV})

rs <- sort(SampleSoV - quantile(DeltaStars,

c((1 - ConfidenceLevel) / 2,

1 - ((1 - ConfidenceLevel) / 2)),

names = FALSE))

res <- data.frame("Bin" = "All",

"SoV" = SampleSoV,

"LowerLimit" = rs[1],

"UpperLimit" = rs[2])

return(res)

} else {

if (any(unlist(lapply(Bins, length), use.names = FALSE) <= 2)) {

stop("At least one bin contains two or fewer taxa. WMPD cannot be calculated.")

}

res <- data.frame("Bin" = names(Bins),

"SoV" = rep(0, length(Bins)),

"LowerLimit" = rep(0, length(Bins)),

"UpperLimit" = rep(0, length(Bins)))

for (i in seq_along(Bins)) {

sn <- replicate(n = Replicates,

expr = {sample(x = seq_len(nrow(Vectors[Bins[[i]], ])),

size = nrow(Vectors[Bins[[i]], ]),

replace = TRUE)})

SampleSoV <- sum(apply(X = Vectors[Bins[[i]], ], MARGIN = 2, FUN = var))

DeltaStars <- apply(X = sn,

MARGIN = 2,

FUN = function(x) {sum(apply(Vectors[Bins[[i]][x], ],

MARGIN = 2,

FUN = var)) -

SampleSoV})

rs <- sort(SampleSoV - quantile(DeltaStars,

c((1 - ConfidenceLevel) / 2,

1 - ((1 - ConfidenceLevel) / 2)),

names = FALSE))

res[i, "SoV"] <- SampleSoV

res[i, "LowerLimit"] <- rs[1]

res[i, "UpperLimit"] <- rs[2]

}

return(res)

}

}
