# Supplementary material for: Sauropodomorph evolution across the Triassic–Jurassic boundary: body size, locomotion, and their influence on morphological disparity
Source: Sci Rep. 2021 Nov 18;11:22534. doi: 10.1038/s41598-021-01120-w (PMC8602272; doi:10.1038/s41598-021-01120-w)
Supplement: Supplementary file 5 — Supplementary Information 5. [file 41598_2021_1120_MOESM5_ESM.docx]

Taxon FAD LAD FL Mass log(Hc)/log(Fc) Area Locomotion Locomotion_red

Eoraptor 231.7 226.27 15.2 0.0173 0.86 SA Biped Biped

Saturnalia 233.84 226.27 15.7 0.0106 0.88 SA Biped Biped

Panphagia 231.7 231.1 19.0 NA NA SA Biped NA

Chromogisaurus 231.7 231.1 17.3 0.0130 NA SA Biped NA

Buriolestes 233.84 226.27 13.6 0.0048 NA SA Biped NA

Pampadromaeus 233.84 226.27 14.5 0.0085 NA SA Biped NA

Bagualosaurus 233.84 226.27 21.5 0.0255 NA SA Biped NA

Nambalia 225.4 208.5 27.3 NA NA IN Biped NA

Thecodontosaurus 208.5 201.3 21.0 NA NA EU Biped NA

Pantydraco 208.5 201.3 NA NA NA EU Biped NA

Efraasia 215.56 212 62.7 0.1797 0.92 EU Biped Biped

Ruehleia 225.4 208.5 80.0 0.9263 0.93 EU Biped Biped

Jaklapalisaurus 225.4 208.5 40.9 NA NA IN NA NA

Macrocollum 225.67 220 33.4 0.0866 0.91 SA Biped Biped

Unaysaurus 225.67 220 27.0 NA NA SA Biped NA

Plateosaurus_ingens 212 205.6 NA NA NA EU Biped NA

Plateosaurus_gracilis 225.4 208.5 54.3 NA NA EU Biped NA

Plateosaurus_engelhardti 225.4 201.3 93.0 0.917 0.91 EU Biped Biped

Pradhania 199.3 190.8 NA NA NA IN Biped NA

Glacialisaurus 199 182.7 60.0 NA NA ANT Biped NA

Coloradisaurus 220 213 51.93 0.437 NA SA Biped NA

Yunnanosaurus_huangi 201.3 190.8 NA 0.574 0.94 AS Biped Biped

Lufengosaurus 201.3 190.8 78.0 0.481 0.92 AS Biped Biped

Xixipiosaurus 201.3 190.8 42.8 0.341 NA AS Biped NA

Massospondylus_carinatus 201.3 187.5 55.0 0.520 0.92 AF Biped Biped

Adeopapposaurus 201.3 190.8 22.7 0.0481 NA SA Biped NA

Leyesaurus 201.3 190.8 38.31 NA NA SA Biped NA

Plateosauravus 219.6 202.3 60.0 1.350 NA AF Biped NA

Riojasaurus 220 213 60.8 2.230 0.96 SA Quadruped Quadruped

Eucnemesaurus_fortis 219.6 202.3 47.9 1.428 0.97 AF Quadruped NA

Eucnemesaurus_entaxonis 219.6 202.3 53.3 NA NA AF Quadruped NA

Seitaad 190.8 182.7 32.6 NA NA NAM NA NA

Anchisaurus 201.3 190.8 28.0 0.260 0.98 NAM Quadruped Quadruped

Chuxiongosaurus 201.3 190.8 NA NA NA AS Quadruped NA

Jingshanosaurus 201.3 190.8 84.5 3.105 0.96 AS Quadruped Quadruped

Xingxiulong 201.3 190.8 61.4 1.910 NA AS Quadruped Quadruped

Sarahsaurus 199.3 183.7 34.3 0.1617 0.92 NAM Biped Biped

Yizhousaurus 201.3 190.8 81.5 NA NA AS Quadruped NA

Kholumolumo 219.6 202.3 75.5 3.500 NA AF Quadruped NA

Mussaurus 192.7 192.6 80.0 2.850 0.89 SA Biped Biped

Leonerasaurus 189 188.8 35.3 NA NA SA Quadruped NA

Sefapanosaurus 219.6 202.3 64.22 NA NA AF Quadruped NA

Aardonyx 201.3 187.5 68.1 NA NA AF Quadruped NA

Meroktenos 219.6 202.3 NA NA NA AF Quadruped NA

NMRQ3314 201.3 187.5 57 NA NA AF Quadruped Quadruped

NMQR1551 219.6 202.3 64 1.650 NA AF Quadruped NA

Ingentia 213 201.3 105.3 NA NA SA Quadruped NA

Lessemsaurus 220 213 87.5 7.000 0.87 SA Quadruped NA

Antetonitrus 201.3 187.5 79.4 5.640 0.95 AF Quadruped Quadruped

Ledumahadi 201.3 187.5 104.6 12.000 NA AF Quadruped Quadruped

Blikanasaurus 219.6 202.3 49.95 NA NA AF Quadruped NA

Camelotia 208.5 201.3 100.8 3.800 0.97 EU Quadruped NA

Pulanesaura 201.3 187.5 92.2 NA NA AF Quadruped NA

Gongxianosaurus 182.7 174.1 116.4 NA NA AS Quadruped NA

Isanosaurus 208.5 174.1 76.0 3.504 0.97 AS Quadruped Quadruped

Tazoudasaurus 190.8 174.1 123.0 10.300 0.97 AF Quadruped Quadruped

Vulcanodon 199.3 182.7 110.0 10.300 0.95 AF Quadruped Quadruped

Shunosaurus 161 157 125 6.532 0.95 AS Quadruped Quadruped

Spinophorosaurus 174.1 163.5 NA 10.057 0.98 AF Quadruped Quadruped

Patagosaurus 178.7 178.1 127.0 24.385 0.97 SA Quadruped Quadruped

Barapasaurus 190.8 170.3 136.5 NA NA IN Quadruped NA

Cetiosaurus 170.3 163.5 NA 27.300 0.98 EU Quadruped Quadruped

Omeisaurus 161 157 131 7.800 0.97 AS Quadruped Quadruped

Mamenchisaurus 163.5 152.1 123 6.240 0.98 AS Quadruped Quadruped

Ngwevu_intloko 201.3 187.5 28.5 NA NA AF Biped NA

Irisosaurus_yimenensis 201.3 190.8 NA NA NA AS Quadruped NA

Schleitheimia_schutzi 213 208.5 69.0 NA NA EU Quadruped NA
