# Supplementary material for: Sauropodomorph evolution across the Triassic–Jurassic boundary: body size, locomotion, and their influence on morphological disparity
Source: Sci Rep. 2021 Nov 18;11:22534. doi: 10.1038/s41598-021-01120-w (PMC8602272; doi:10.1038/s41598-021-01120-w)
Supplement: Supplementary file 6 — Supplementary Information 6. [file 41598_2021_1120_MOESM6_ESM.docx]

### Plotting function to plot convex hulls

### Filename: Plot_ConvexHull.R

### Notes:

############################################################################

# INPUTS:

# xcoords: x-coordinates of point data

# ycoords: y-coordinates of point data

# lcolor: line color

# OUTPUTS:

# convex hull around data points in a particular color (specified by lcolor)

# FUNCTION:

Plot_ConvexHull<-function(xcoord, ycoord, lcolor){

hpts <- chull(x = xcoord, y = ycoord)

hpts <- c(hpts, hpts[1])

lines(xcoord[hpts], ycoord[hpts], col = lcolor)

}

# END OF FUNCTION
