# Supplementary material for: Sauropodomorph evolution across the Triassic–Jurassic boundary: body size, locomotion, and their influence on morphological disparity
Source: Sci Rep. 2021 Nov 18;11:22534. doi: 10.1038/s41598-021-01120-w (PMC8602272; doi:10.1038/s41598-021-01120-w)
Supplement: Supplementary file 7 — Supplementary Information 7. [file 41598_2021_1120_MOESM7_ESM.docx]

#NEXUS

BEGIN DATA;

DIMENSIONS NTAX=67 NCHAR=419 ;

FORMAT DATATYPE=STANDARD SYMBOLS=" 0 1 2 3 4 5" MISSING=? GAP=- ;

MATRIX

Eoraptor 0001?001?0001000011000111010000010000110100000?101100?0100?01100001101???11?1????????????????10000010???0?0100?1010000001?????110000000000?1011??000??00?00???00?000??00000011??10???100??001?00??000?0000??1?10??00101??????20?00011?00010?0000010110100010002101??0??00?0???10?1011?10??0?0111000001100000?1?0011010??1?0??0?????01????000???00?0010000000??010????????????00100020011110111100110???10?0?????0?00?0?0?1?1???1011

Saturnalia 10????????????????????????1??1????00?100?0??????????????????10?????????0???0?0??0101?0??????????0?0?0??????000010{01}000000????????0010010010??01101?00000100001100100000000000000001000101??0??1000??0??10101111100010100?????????????????????????????00?0001100210100000000100110010010000001001100000110001000000010100010010010100000001000000001011000?000?0010000000000000000??12??000??11111?1100??101?00?0000?0?????????????01

Panphagia ?0???????????????????0?1???????????????????0??1????????1?1?0???????10?0?111????0?????????????1?0?0?1010000?10?0101000000????????00000100011?01101?000?01???01?10000?0?0000000??0???00??????????00??0??000???????????????????????????????????????????0?10?011002101?00??000????1??0??10?????????????????????001000110100???010?1010?????????????0??????????????????????????????????01???????111?1?0?????????????00??0??1?211????????

Chromogisaurus ????????????????????????????????????????????????????????????????????????????????????????????????????????????????????????????????????????????????????????????????????????????????01000??1100??1???????????????????????0??????????????????????????????0????011002101??????????????????????0??????1???00110??1000000010100010????????????????????????0??0???000???10?????????????????12???????11?11?????0?????????????????????????????

Buriolestes 0?01000210101010011000111000000000000100100000?10110010100001?00001?01100110000???0?0??1??00100000010100000100010000000010100010010001001011?1101000000100011?00000000000000000?0?00010?0?000?000??0???1????111010011?0???????????01????????????????00000?10002100?0??0?001?011?0001101000000111000001101000?1?000?01?0?1??1?0?????0100???000??00?0100000000??0100?10??10????????????????????????????0010?{01}????????0?01?{12}??1???????

Pampadromaeus 0001000210101?10?11000?11000??00100001100000????0?10010??0??1?????110?1??????????????????000???000000??00001100101000000??10001????????????1??????0??001??0?1?10??0000000000{01}0?0??0001?10?0??1000??0??1110??1?101010000?????????????????????????????0000011110?102?????????0?1?????????00001011100010110?0?00?00???????01?????????????????000???0??1?0???0???????0??????0??????????10?100??????????????00???????0??????????????????

Bagualosaurus 10?1?00{12}??0?{12}0???1??????001?000?1000?10??0???0?10010?????????????0??????????????????????????010001010?????01?00101000?00??????????????????????????0??001??0?1???10?0?0000000?0??01?001???????01???0??????????????????????????????????????????????????000011100?102?00?00?0?????????????000??0111000101110?10000000?01??010????????????????0?1??00?0110?????????11??0??110??????????10??0????????????????0???????????????????????0??

Nambalia ??????????????????????????????????????????????????????????????????????????????????????????????????????????????????????????????????????????????????????????????????????????????????????????????000????????????????????????????????????????0?10??0110??????02?0??10??0??????????1????111?0100110101001011010???????1101?0???011010??00101???00101?011110??00????00100000010????????????????11?10?0?00????????????????????????????????

Thecodontosaurus ?0????????????????????????1??1??1000??????0?0?0?0???000?????????????0???0?1010?10011?0???????00?10010??????1100101010000?1??????000101010?1?01101?000001??00110000000000000000?1000?0101100?01000??00?010011211000101001100012010001100001010000010100?00121000001???0?000??011??01010?000011110000001100010000000101100??011010100????110001110?1?000??00?0????1????????????1??00020000??01?110???000?20?000?10???0???????????????

Pantydraco ?0???0????????????{01}00?0???1??1????000100100000010?100001?00????????00?000?1?10??00110011?000?00?00010??0000?1001010100001001??1100?10101001101101?00???1????1???????????????????????0??11?00010000100?????11?1?00???????????????????????????????????00100121100001?????????0011??10110?00??????????0?1?000?0000000?01?001?????????????????001110011?000000001001?0??00000????0????0200??1??1????0??0??0??1100??00??0?0??2101?1?00??

Efraasia 100?1001?000??1?111?112?1110?100???00100100000??0?10??01?0??1??????10?000?1????10011100??????10010010??11?011001010100001???1?11001101001011011010000001??001?00000000000000000110000101000?0110000?0?010011212000111001101012010?11000001110100010000100131000101000010000001100101101000011110000001100010000001101?001?011010?0?01?1110001??0011?0010000010002??0???000?10001000100001??1111??1000002011001?00??0????2?11?0?????

Ruehleia ???????????????????????????????????????????????????????????????????????????????????????????????????????????????????????????????10???0????01???101?002001??00110010000011000000?1100001?00?0?0110??????01001111200011100?0100??01011101000???0??00???001001311000020??11000110110?10110?101011010000002100010?00?011011001?000010100?????????????????????????????3?????????0?00010??????????????????0???1???????????0???????????????

Jaklapalisaurus ??????????????????????????????????????????????????????????????????????????????????????????????????????????????????????????????????????????????????0???0??????????????????????????????????????1??????????????????????????????????????????????????????????????????????????????????????????????????????????00?000010110110???011010??0???????00??1?0??1?????????????00??101???????????????????????1???????????????????????????????????

Macrocollum 1001100210002111111111211110010010000100100000110010010100001000001101??????101???????0?100??1001011010?1?0110010101000011?0111100200100111101101000000111011101000000000000100110?0010?0000001000100?01001111200001100110111201010101000211000001000010012100010200?0?0001?01100101110100000110000101100010000{01}111011001001101010001011100010100111000000001000100000011001?00000?????????????????110010???????0?00?1?????????????

Unaysaurus ?001100?1010??1?111??1??1010??011000????????????0??00?????01???000??0?0?????????????10????????0?10110?????01100101010000???0?1?????0?1????????????0??0011100110000000000000???????????????????1000?00?010001?1101001100??????10?0?110????1110??0?????????????????????????????????????????????????????????????????110110???01?010?00?????????11???????0???0???????0????????????????????????????????????01?110??????????1????1???????

Plateosaurus_ingens ????????????????????????????????????????????????????????????????????????????????????????????????????????????????????????????????????????????????????????????????????????????????01??01????????10???????????????????????????????????????????????????????????????????????????????????????????????????????????00?010????????????????????????????????????{01}???0?0????4????????00??00??????????????110???????????????????????????????????

Plateosaurus_gracilis ?00??001?0002?110111?1??1110?101??00?10?1010?0??0?10001100??10??????????????????1?????????????0??0110?????11100101010?001?????1100??01???01101101?00?001?100110000000000000010?1010001010000001000?00????????1200?11100?1011?10101010000???10?????0?001001311001020010100011111001011??00001111000000110?010000?0??0110????????????????????0?????????0?0????????{23}????????001000?000?00001101111?1?01?00????????????0????????????0??

Plateosaurus_engelhardti 1001100110002011{01}11111211110010110000100101010110011001100011001001101100101101110011000101111001011010111111001010100001100111100110100{01}011011010002001110011000000000000001011010001{01}000000010000000010011112000111{01}0110111101011100100201000001000010013110010200101000111110010111100001111000000110001000010111110010011010100010111000110001110010000010004000000111010000000{12}000001011110?1010001002000100000001?01111102001

Pradhania ????????????????????????0?1???????00??????????????????????????????????????00??????????????????0???1?1??????????1?1010?????????????20??????????10??????????????????????????????????????????????????????????????????????????????????01?????20?0??????????????????????????????????????????????????????????????????????????????????????????????????????????????????????????????????????????????????????????????????????????????????????

Glacialisaurus ????????????????????????????????????????????????????????????????????????????????????????????????????????????????????????????????????????????????????????????????????????????????????????????????????????????????????????????????????????????????????????????????????????????????????????0????????????????0????????????????0???1010{01}????11010111?01???????????????101110111?0????????????????????????0???0??????????????????????????

Coloradisaurus ?00?1002??002?111111?1??1110??111000?10??01?10?101110111?000100100?1011011011010100111?????1?1001011010011??000101010001?11?001100200100101?01100?00?001?1001100000000000000101????00?10100?001???????00001011210011?1???????????????1???????????????0?0?131100???011?11002001100101110000011010000002111010000001101100??011110?00??????0001110012000110000210021111101100111????0?0000??????????0?00?101100?100000?1002100001??00

Yunnanosaurus_huangi 100?1002??00??10?110?1??111?111????00101111?00??0?1101111?001000?01?01?0?00010???????????????10010010???1???00010?21000?????111100110110100?01101000?0011?00110000000000000010?0101101{01}0000?00?0??0??000?0??11200?11110??01??0011?2101100201000{01}020?0010013100000001101{01}00{12}0?110010111?0010110100000011010101000011011001?01101010001111?010111001????1?0000110?2?0???11??0??1??010100000?01?1???00??0?10??????????0?000????????1??

Lufengosaurus 100???02???0211?1?11?1111?111110100101011010000101110101100010?1????0110??01{01}0??1001?1001001010010????0111?11001010100011?00011100100110111?0110100020011100110000000000000010011010011000000{01}10000000001011112{01}1011110110111101113100101201000102000010013110000201101100210110010110000101101000000211101010000110110010011110?0001?1110101110012000110000{12}{01}00410111011???00?0000100000??1????1????0010??0????????111??1?????0???

Xixipiosaurus 100?10?????0??111?11?1111?1111?0??000101?11000?10?11?1111?0?1001????01?0??01?01??????????????1?01?010???1??11001???1?????????????????????????????????????????????????????????????????????????????????????????????????????????????????????????????????????????????????????????????????????????????????????????????????????????????????????????????????????????????????????????????????????????????????????????????????00????????????

Massospondylus_carinatus 1001100210002111111111211011111010000101101000110{01}11011110001000102?01101100{01}0110001{01}1?01000010010110101010110{01}1010100011100?111002001001111011010002001110011000000000000001011101001{01}00000000000000?01{01}011112010111{01}01101111011121001012110000020000100131000100011011001001100101110001011010000002101010000001101100100111101000111110001110012000100000210020?00?011001000001?100000????????1???0010100111000000000{01}1000010000

Adeopapposaurus 1001100210002111011111011011111010000101101000110111111111001000102101{01}01100101100011100101000001011{01}10101011111110{01}00011100111100200100011101101100200111001100000000000000100110100100000000010000000000101110001110011011110111210110011100000200001001311001000110110010?11001011000000110100000021010100000011011001001111010001110100011100120001100001100100000011010010000??00000??1?11?11???00101001?100000?010?11100?0000

Leyesaurus ?001?00?1000211??11?????1010?11010000101101?00?1011111111?00100010{02}1010?????????00?11???0??1?00?10110??1010?111101000001?1??1111002001000111011????0???????????????????????????????????0000?001???000??????????????????????????????????????????????????????????????????100?????????????????????????????????????????????????????????????010???????12000????0???0??????00?????????????000??????????????0??0?00??1?0?00?0??11?1?????00

Plateosauravus ???????????????????????????????????????????????????????????????????????????????????????????????????????????????????????????????10???0100101?01101?00{12}0011?00110000000001000000??10??011???0??110????????0???{23}1110011110????????????1????????????????00100131100101?01????????11??10110?00001111000000110001000000110110??????0???????????????11?0????0??????????4????????????000???????????????????0???2???????????0???????????????

Riojasaurus 1001?00??0002011?110??00111011011000?100001000?10010?00110001000001101?0?100101?0001???????1?001000?1??101?100010101000010??11110011010010110110100010011100110000000000000010?1101001{01}00000011000??0?01001121210111100111111?010111011001010001020?0010013110010201101000110110010110010110111010100201101000000110110010011010100??011?00011100121011{01}00001??13????????0?10100010100000?011110?00110020??0?????000?????????????00

Eucnemesaurus_fortis ??????????????????????????????????????????????????????????????????????????????????????????????????????????????????????????????????????????????????0???01??001100{01}10000??00?0??????????10000?01100?????????11?????????1?????????????????????????????????????????????110???0??????????????0110111010?0010110?000001110110?????????????????????????????????????????{23}??????????????????????????????????1?0?????????????????????????????

Eucnemesaurus_entaxonis ??????????????????????????????????????????????????????????????????????????????????????????????????????????????????????????????????????????????????0???01??0????0???000??0000?00?1011?0100?0?01???????????????????????1??????????????????????????????0????13??{01}0100??1??0?0?0?11?010110?1011011101000010110?0?????110111?1?0??0?????0??????00111?01{12}?0?11000?????20???0?????????????????????????????1???????????????????????????????

Seitaad ??????????????????????????????????????????????????????????????????????????????????????????????????????????????????????????????????????????????????00?{01}?1??00??00?001????0000?????????????????????????0000011?11000111{01}011?11100101310?00?2110??0???0??????????????0?1000001????????????????????????????????0?00?01101?0???01101010?0??11?00?1??001???11{01}0000010???0???0?10??0??001??????????????????????0??????????????????????????

Anchisaurus 10???00??0002?1??11?????111011?010?001010?100011001111011?0?10??????01?001101?1?2001110??????10?10?00??10000000101?1{12}?0111?0011???1001001111011?10000?010?001100?000??0000000??0100?0??0?01?00?10?????10{01}0??211001011?0??0??1101011100100201000002000{01}10113100000?0111100010011?001010000101101000000{01}11101000000110111?1?01?010???10111?0?01??0011?0010000010011??0????10??0?0?01020000??01?11?110100?20?10????000??01????1???????

Chuxiongosaurus ?00??002??00??111111?1{01}?11101{01}10??00?11100???1??0?1101111?0?1000????01???????????????????????101?00?0??111?1100?0101??????????????????????????????????????????????????????????????????????????????????????????????????????????????????????????????????????????????????????????????????????????????????????????????????????????????????????????????????????????????????????????????????????????????????????????????????0????????????

Jingshanosaurus 1001?002??00211111100???11101{01}101100?11?001??1?10?11011110001000????01100000101?2?1??1000??1?10110010??11101100101011001????111??010??????1???10100??0011100?1???00000000000?0??10??01100000001000000?0010??11201011110??00??0011?3100100?010001020000100131000002011001002??110010110?00001101000000111101010000110110?12011010?00??????01011110120011{12}0000010?4????????????????1???????????????????0010??????????????????1????1??

Xingxiulong ??????????????????????1???1?111????0?10?00???0110?11001100001000101?01?00100101?2??1?1???????0?????????0{01}1???00?????????1100111100100110001?011010002001110011000000000100001100110??11?1000001????00?0000???120?011110????1??????1101??????????????001001311000020?1011002??11??0001000000111100000011010?00000{01}101110010011010?0?10110001011000121011100002100200000?11????????????????????????????0??0??????????0?00????????????

Sarahsaurus 10?1000?10?11010?11?1?1?0110110100000100001010010?110001100010?0???101000111001???01?1001??1?10?10010?????011001010100001?1011110110011101110111110010010100101110000010000010111000011000010010000001000010102100111111101111011021111?011100001202100001311101021001110011111011011102?1001100010001110010000010{01}01000100011101001?1111011?110011000?1000111001??0???11010??000????????????????????0010??????????0010?0??1?1010??

Yizhousaurus 110110021010211111101111111111011000011100100011001111111100??00???10?100?00101?2?????000001010110010??0000110{01}1010110011?10111100100110101?011010{01}000011100{12}10000000001000010?01000011?000?01???????001001111100011110??????0010?21011011010001020000100?31100000010011002?0110010110?1110111100001011110??????????????????????????????????????????????????????{34}??1???1?????????????????????????????0?2???????????0???????????????

Kholumolumo ????????????????????????????????????????????????????011?????????????????????????????????????????????????????????????????????????0101?1?1????0??01?00?0011???2??0000101??????????????0110??0?111100????010????11000{01}??00???????????2101???1?11???????10000131110000010?10001????????????00011110000001110?1?0010010001000?1?????????????????0111??1200???????????31?000011?1?001010???????????????????????????????????????????????01

Mussaurus 10011002?000??111111?11?11101110100001010{01}?000?10011?1011000{01}0?0?01?0?1??????????????????????10101111???1?0??0?1?1111001????????00100????0?10110??0000?1?000100000000?00000010??111?011?1?0001110?????0010?011100111110?1?11?00101210110120100000200???0?13100010?011??1001?011??10110?10101101000000111101000000110111011011010001??????01011100120011100001100300?00111111?011010{12}???????111??11?????10??????????0?01?????????1{01}{01}

Leonerasaurus ?0????????????????????????????????????????????????????????????????????????????????????????????????0?0???????1?11010010011???001??010?1???????1100?00?0?10000110000000??00000100?111001????????????????0000???1100000?????????????????????????????????1101?3100?????????0?????11??1?1??????????????????????????????????????????????????????10111??????????0???????0??0???1?0????????1???????1???????????????????????{01}????????????100

Sefapanosaurus ????????????????????????????????????????????????????????????????????????????????????????????????????????????????????????????????00??01100???0?10??00?001????1?00000001??0000????1?????11?00??011????0?0?0010?1?00????10?1011??010131010??1010???????????????????????????????????????????0?111110000??11{01}???000000??????0?00110101010001??0???11?0?2?0????????????00?0????011?01111?????????????????????????????????????????????????

Aardonyx 1101?00210002?11?11?????1110?1011000??????1010??0?11010??10?1????????1?0???????????????????1??0?0?1?0?????11?00101011001?1??????0010?110100101101?0000011?00110000000020000010?1100?0110000?01110??00?????11?????????10??00??????13101???1?10??10?????????????????1110100010011??10110?101?111100000111000???????1101110?0???01??????????010111?01?0?2??00?0???0{34}???00011???0?111????????????????????0??0??????????0???????????????

Meroktenos ????????????????????????????????????????????????????????????????????????????????????????????????????????????????????????????????????????????????????????????????????????????????????????????????????????????????????????????????????????????????????0??0?131?00001011?00001????????????111111100?000111110???????????????????????????????????01??????????????????1??0???????0??????????????????????????????????????????????????????

NMRQ3314 1001?1031000211111101?1110101111???1010101101011011101011?00000010220110110010????0??10011011001?00101?1000110?10021100110?01011001?01101001011{01}10000001??0??100??0?0?10000000??111001??010?0?1??0?????1????{23}1100?0?110??????0010?2100101{12}0?0001020000100?31000{01}0{12}????????????1??10110?????????????1???11010000011?011?01?0??0?0???0011100101111012?011{12}00001100301100?11????????????????????????????0002?{01}0???????000?????1???????

NMQR1551 ?????????????????????????????????????????????????????????????????????????????????????????????????????????????????????????????????????????????11???00?001??00210000000010000000??2?10012?000?0{01}1100????0100??2????000110???????????21????????????????00100?3100000{01}011010001????????????11101111001110{01}111010000011101100100?10101000??????1011100????1120000????3001?0?11????????????????????????????00?1??????????????????????????

Ingentia ???????????????????????????????????????????????????????????????????????????????????????????????????????????????????????????????????111????01011?0??0?????????????????????????????????????????????????????????11{01}0010110?1?10?00111310110???????????0??????????????????????????????????????????????????????????????????????????????????????????????????????????????????????????111????????????????????????????????????????????????01

Lessemsaurus ????????????????????????????????????????????????????????????????????????????????????????????????????????????????????????????????0?011110100??1101?0000011100{12}10000010121001000????????????????????????0001???11{01}0010110???????0?1?310?????0?0??1????0010013100000?011110001???1??10110?111011?10???01111?0?000000100101???011011101??????????11??12101??00??0???311?????10?201??1001?????1?1111??1010????????????????????????????01

Antetonitrus ?1?????????????????????????????????????????????????????????????????????????????????????????????????????????????1?1012000????????0?{01}0???????????00?000001??00{12}10001010121001000???1????100?0?01100???0?0011??311000{01}0110??????0011?3101???1010???????00100?3???0002011010001????????????11101111001101111101000000100111010????????????????1111110????{12}1200?0????31?100??1?0?00111????????????110??0????2???????????????????????????

Ledumahadi ????????????????????????????????????????????????????????????????????????????????????????????????????????????????????????????????????????????????1?0??0011???{12}?0000010121001000????1???1??00?01???????????????????????10???????????{23}1????????????????????????????????????????????????????0???????????????????????????????????????????????????????????????0???????{45}??0???1?????????????????????????????0?????????????0???????????????

Blikanasaurus ???????????????????????????????????????????????????????????????????????????????????????????????????????????????????????????????????????????????????????????????????????????????????????????????????????????????????????????????????????????????????????????????????????????????????????????????????????????0000?0110111?1?0100101001011100101111112102120000100?211?0?1?11?0?0??????????????????????0???0??????????????????????????

Camelotia ???????????????????????????????????????????????????????????????????????????????????????????????????????????????????????????????????1???????????0??0??001??002100000000??00?0??????????1???0??011???????????????????????????????????????????????????????????????????1??????????1??00111?111011110111?1??110?000000????????????????????????????????????{12}???000????5??????????????????????????????????1???????????????????????????????

Pulanesaura ???????????????????????????????????????????????????????????????????????????????????????????????????????????????10?012011????????001?11?0100??11?????0??1??00?0010?01?0001000????????112?11011???0??????????????10??1?????????????????????????????????????????????????????0?11??1?11???????????????????????00000100101?????????????????????????????????1????????????0?????????????????????????????????1?????????????1???????????????

Gongxianosaurus 1?????0??????????12???????????????????????????????????????????????????????????????????????????????????????00???1??2121????????????0????????????1??0??001??0??????0?0??00000????????????0??0?0?1?0?000?011???2110????1?1?????????????????????????????0?100????0?????????????????????????{12}111?2??????0???1101??????1?0??1???0?1?1????1?11??010???10?2?1112000020005??????????????????????????????????????22??????????????????????????

Isanosaurus ????????????????????????????????????????????????????????????????????????????????????????????????????????????????????????????????0??0???????????2??10??0???????????????2?011000???????????????????????101?0?????????????????????????????????????????????????????????????????????????????211112??0???001?1?0??????????????????????????????????????????????????????3?110??1??????????????????????0????1???????????????????????????????

Tazoudasaurus 11??????????????????????????????????????????????????010??1?????????{02}011???1???1??????????????00?00000???0??10?1111012101???000??001010??????0002??100001??00200100011020121000????????211?1?111????11?????01?1100100?2????????010?210000100?0001?310?1???03101??0?111001002??11??1??1??21100111001111011?0?0010000101010?1111011111?011??????????????1??10?1????51??0????1?0??1001?????????????????10??????????????2???????????????

Vulcanodon ????????????????????????????????????????????????????????????????????????????????????????????????????????????????????????????????????????????????????????????{12}????????????????????1??????0111?0?00?0?0??01???2110?10?021???????????????????????????????????310{12}?00?01111000100111110010?111011110010111?1??20?10001?0???11?111011?111011??11?01010120111110?121?05?1??00?11?00000?????????????1???11?01?21??????????????????????????

Shunosaurus 11000113?110201101200000001011?20100111201010101001101012000011011121010001011?1200?????110200100000120100011111112121112??01011?1001?1?10010012?1100001011021?00001?0201210000?11?00?100111011001?111?1?000211001100?11000?1011001?000110000??1?3101111013101100011111?01100?111100100211112??0???100?01121010?01?0????110?101???11011??1111102002?12111?1021105???????????0?00020?00000??1?10???0??1121???0??11?12?0???1?101??1??

Spinophorosaurus 1????1?????????????????????????????????2????????????0????01?????????0??101111111{12}?0???????????1?????????????100111012?112?????11?11011?0?01?0002?110??01????{12}?11???1?12?1010?0????????{12}1??11011?00?1??1110?03?{12}0?10??{12}???????????????????????????????????????{12}????1?1???01{12}??011??001??211111110?1101??0?020?100???0??11?10??0?0?01???????????????????????????????????????????????????????????????????1???21?0?1?010??1????1???????

Patagosaurus 11????????????????????????????????????????????????????????????????????????????????????????????1?00001??????0??0111012111??????1100101100000110020?2001010?00201?01111120121?00?0210011{12}00?1?0?100???0?111?00?1100100?{12}1?????????????????????????????1111013101000011111001100111110010?211112??0???1011111?101000??0????????????????????????????????????????????5???????????0?????02???????1?11???110??????????????2?????????????11

Barapasaurus ???????????????????????????????????????????????????????????????????????????????????????????????????????????????1?111211???????????1?1????00??002??100101??00201101111120121100?0{12}1001?10?11??1????????101?00?1???????11?????????????????????????????11110131011000111110011???11110010???????????????????1?1?1?????????1?1???????1?????????0???????????2?1??????5????????0000?000?01???????0?11?1?1?01?????????????????????????????

Cetiosaurus 1????????????????????????????????????????????????????????11?????????????????????????????????????????????????????????????2??0011110111110100100020?2001?10?1020??1011102010100???{12}?????2001??00100???011110103110010002????????1?????????????????????1111013?0210?0110?1?011??1101?00???211112??0???110?111210100010010111???????????????????????????????????????5?????????1?000??????????????????????1?2???????????0???????????????

Omeisaurus 110001131111201101200000101011?201001112010101011111010121100000112221110010111???0??0???1???11101001?0?00011101111121113??001110120011010111002?12101010110201100011020121100?021??1?20011100100?011111100031100100021??????0100001000110001001?31011110131011000111110011000111100100211012??0???110?01021010011?0??111?011010?11??1????1111021020121111102?105?????????0?0?0?0???00000??1?1????1??1?2???????????0?0???1?????????

Mamenchisaurus 11000113?11120110120000000101112??001112010101011111110121100000111221010000111?201??0?????2?11{01}0000100100011111111121113?0001110121010000110002?1110101010020110001102012100000110111211111011001011111100031100100120110011011000100011?0?10?1?31?11110131011?00111110011000110100100211112??0???110?01121010?1??0??????101011111??????1101??21?2?121111102??05?1?????????0?00?????????????????????11211?1?0??1010?0?1?1?0???????

Ngwevu_intloko 1-01010??10021111111-???111-10101000?10110-000?10-110111111-0001100101001111001--00--111?001011?11010?????011011010100011????111?020?11001110?1??1?00?0???0??????0???0000?0010000??????-????????????????0???1121?101??0???????????11?????2010?????????????????????0?1?1100?????????????1010?101000010??1??1???????????0??????0?0?????????0001??0??2?00??000???0?--0?????1--------0----------------------------------011011010100---

Irisosaurus_yimenensis ?????????011??1???1?????10????1??????????????????????????????????????????????????-?--??-?????????????????????0?10?21100?????-??-00-????--????110??00?001-??-??-00-0???0?0?00???????-??--??-???????????-1-010??1??01?1-0????-?1010121011012010000020??????-?????-???????????-?-1??10110?--??-???????-?-??????????-??????????-?????????????????????????????0?0?????-???????----------------------------??0-----------?---------------

Schleitheimia_schutzi ?????????????????????????????????????????????????-???????????????????????????????-?--??-????????????????????????????????????????0??????????????01?10?0111?0???????000??1??????????????-???1??110?????????????????0?0?-???????????????????????????????????-3111{02}-02?????????????????????????????????-?01110??????-???????????????????????????????????????0???????4-?1???-?????-????-------------------????--------------------------

;

END;

BEGIN ASSUMPTIONS;

OPTIONS DEFTYPE=unord PolyTcount=MINSTEPS ;

TYPESET * UNTITLED = ord: 8 13 19 23 40 57 69 92 102 108 117 121 134 144 147 149 150 157 167 170 171 177 183 205 207 214 222 227 242 251 254 277 299 336 342 349 353 370 393 404 409, unord: 1-7 9-12 14-18 20-22 24-39 41-56 58-68 70-91 93-101 103-107 109-116 118-120 122-133 135-143 145 146 148 151-156 158-166 168 169 172-176 178-182 184-204 206 208-213 215-221 223-226 228-241 243-250 252 253 255-276 278-298 300-335 337-341 343-348 350-352 354-369 371-392 394-403 405-408 410-419;

END;
