# Supplementary material for: Sauropodomorph evolution across the Triassic–Jurassic boundary: body size, locomotion, and their influence on morphological disparity
Source: Sci Rep. 2021 Nov 18;11:22534. doi: 10.1038/s41598-021-01120-w (PMC8602272; doi:10.1038/s41598-021-01120-w)
Supplement: Supplementary file 8 — Supplementary Information 8. [file 41598_2021_1120_MOESM8_ESM.docx]

set.seed(2020)

library("Claddis")

library("dplyr")

library("dispRity")

library("ggplot2")

library("nlme")

library("ape")

library("paleotree")

library("phytools")

library("fBasics")

library("geomorph")

library("vegan")

#Import functions to calculate disparity measures and draw convex hulls

source(file = "Disparity_support_disparity_measure_functions.R")

source(file = "Function_for_convex_hulls.R")

#Import matrix in nexus format

matrix <- read_nexus_matrix(file_name = "matrix_saurop_only.nex")

matrix

#Load temporal data

data <- read.table("FADLADalltax_R1.txt",

header = TRUE,

sep = "\t")

#Create temporal data

temp_data <- data[,c(1:3)]

#Check match between taxon names of matrix and temporal data

setdiff(names(matrix$matrix_1$matrix[,1]),temp_data$Taxon)

#Calculate proportion of missing data in each taxon

miss_data <- apply(X = matrix$matrix_1$matrix,

MARGIN = 1,

FUN = function(x) mean(is.na(x)))

#Check taxa that have more than 70% of missing data

miss_data_higher_0.7 <- miss_data[miss_data>0.7]

#Assign taxa to each time bin

Carnian <- temp_data[temp_data$FAD>225.67 & temp_data$LAD<233.2,]

Norian_Rhaetian <- temp_data[temp_data$FAD>201.3 & temp_data$LAD<225.67,]

pre_Toarcian <- temp_data[temp_data$FAD>182.7 & temp_data$LAD<201.3,]

Toarcian_MJ <- temp_data[temp_data$FAD>66 & temp_data$LAD<182.7,]

intersect(Carnian$Taxon,Norian_Rhaetian$Taxon)

intersect(Norian_Rhaetian$Taxon,pre_Toarcian$Taxon)

intersect(pre_Toarcian$Taxon,Toarcian_MJ$Taxon)

#Create MORD matrix.

dist_matrix <- calculate_morphological_distances(cladistic_matrix = matrix,

distance_metric = "mord",

distance_transformation = "none")

#Check if we need to trim taxa to generate the ordination of the distance matrix

trim_dist_matrix <- trim_matrix(distance_matrix = dist_matrix$distance_matrix)

#Take a look to the proportion of missing data present in trimmed taxa

miss_data[trim_dist_matrix$removed_taxa]

#Conduct PCoA without correction

pcoa_result <- pcoa(D = trim_dist_matrix$distance_matrix, correction = "none")

#Check for negative eigenvalues

pcoa_result$values$Eigenvalues<0

#Conduct PCoA with Lingoes correction

pcoa_result <- pcoa(D = trim_dist_matrix$distance_matrix, correction = "lingoes")

#Observation of eigenvalues

plot(pcoa_result$values$Rel_corr_eig*100)

sum(pcoa_result$values$Rel_corr_eig[1:3]*100)

#A graphical exploration of the scree plot indicates that the last change in slope is between PCOs 17 and 18

#Thus, we will use the first 17 PCOs for subsequent disparity analyses

sum(pcoa_result$values$Rel_corr_eig[1:17]*100)

#Calculate distance from centroid for each taxon

dist_2_centroid <- get_dist_to_centroid(pcoa_result$vectors.cor[,1:17])

#Explore correlation between distance to centroid and proportion of missing data

plot(miss_data[rownames(trim_dist_matrix$distance_matrix)], dist_2_centroid, pch=19)

cor.test(miss_data[rownames(trim_dist_matrix$distance_matrix)], dist_2_centroid)

###It is a significant correlation, we have to keep an eye on this...

#Assign non-trimmed taxa to each time bin (from A to D)

A <- intersect(Carnian$Taxon,

rownames(pcoa_result$vectors.cor))

B <- intersect(Norian_Rhaetian$Taxon,

rownames(pcoa_result$vectors.cor))

C <- intersect(pre_Toarcian$Taxon,

rownames(pcoa_result$vectors.cor))

D <- intersect(Toarcian_MJ$Taxon,

rownames(pcoa_result$vectors.cor))

bins.trim <- list(A, B, C, D)

#Plot preliminary morphospace

#pdf(file = "graphics/Morphospace_with_labels.pdf", width = 6, height = 6)

plot(x = pcoa_result$vectors.cor[, 1],

y = pcoa_result$vectors.cor[, 2],

xlim = c(min(pcoa_result$vectors.cor[, 1]), max(pcoa_result$vectors.cor[, 1])),

ylim = c(min(pcoa_result$vectors.cor[, 2]), max(pcoa_result$vectors.cor[, 2])),

pch = 19,

xlab = "PCo 1 (9.82%)",

ylab = "PCo 2 (3.90%)")

text(x = pcoa_result$vectors.cor[, 1],

y = pcoa_result$vectors.cor[, 2],

rownames(pcoa_result$vectors.cor),

cex = 0.7)

##dev.off()

#There are five probable outliers because of bias of MORD

outliers <- c("Chromogisaurus","Jaklapalisaurus","Leyesaurus","Plateosaurus_ingens","Pulanesaura")

miss_data[outliers]

####Leyesaurus has a 64% of missing data, it is not extremely high.

####So, I don't consider it an outlier.

outliers <- c("Chromogisaurus","Jaklapalisaurus","Plateosaurus_ingens","Pulanesaura")

miss_data[outliers]

#Graphic morphospaces

#pdf(file = "graphics/Morphospaces.pdf", width = 6, height = 6)

plot(x = NULL,

y = NULL,

xlim = c(min(pcoa_result$vectors.cor[, 1]), max(pcoa_result$vectors.cor[, 1])),

ylim = c(min(pcoa_result$vectors.cor[, 2]), max(pcoa_result$vectors.cor[, 2])),

xlab = "PCo 1 (9.82%)",

ylab = "PCo 2 (3.90%)")

abline(h = 0, lty = 2)

abline(v = 0, lty = 2)

points(x = pcoa_result$vectors.cor[A, 1],

y = pcoa_result$vectors.cor[A, 2],

col = "brown1",

pch = 19)

Plot_ConvexHull(xcoord = pcoa_result$vectors.cor[A, 1], ycoord = pcoa_result$vectors.cor[A, 2], lcolor = "brown1")

points(x = pcoa_result$vectors.cor[B, 1],

y = pcoa_result$vectors.cor[B, 2],

col = "chartreuse3",

pch = 19)

Plot_ConvexHull(xcoord = pcoa_result$vectors.cor[B, 1], ycoord = pcoa_result$vectors.cor[B, 2], lcolor = "chartreuse3")

points(x = pcoa_result$vectors.cor[C, 1],

y = pcoa_result$vectors.cor[C, 2],

col = "cyan3",

pch = 19)

Plot_ConvexHull(xcoord = pcoa_result$vectors.cor[C, 1], ycoord = pcoa_result$vectors.cor[C, 2], lcolor = "cyan3")

points(x = pcoa_result$vectors.cor[D, 1],

y = pcoa_result$vectors.cor[D, 2],

col = "darkorchid1",

pch = 19)

Plot_ConvexHull(xcoord = pcoa_result$vectors.cor[D, 1], ycoord = pcoa_result$vectors.cor[D, 2], lcolor = "darkorchid1")

legend(0.28, 0.32, legend=c("Carnian", "Norian-Rhaetian","pre-Toarcian","Toarcian-middle Jurassic"),

col=c("brown1","chartreuse3","cyan3","darkorchid1"), pch=19, cex=0.70, box.lty=0, bty = "n")

plot(x = NULL,

y = NULL,

xlim = c(min(pcoa_result$vectors.cor[, 1]), max(pcoa_result$vectors.cor[, 1])),

ylim = c(min(pcoa_result$vectors.cor[, 3]), max(pcoa_result$vectors.cor[, 3])),

xlab = "PCo 1 (9.82%)",

ylab = "PCo 3 (3.15%)")

abline(h = 0, lty = 2)

abline(v = 0, lty = 2)

points(x = pcoa_result$vectors.cor[A, 1],

y = pcoa_result$vectors.cor[A, 3],

col = "brown1",

pch = 19)

Plot_ConvexHull(xcoord = pcoa_result$vectors.cor[A, 1], ycoord = pcoa_result$vectors.cor[A, 3], lcolor = "brown1")

points(x = pcoa_result$vectors.cor[B, 1],

y = pcoa_result$vectors.cor[B, 3],

col = "chartreuse3",

pch = 19)

Plot_ConvexHull(xcoord = pcoa_result$vectors.cor[B, 1], ycoord = pcoa_result$vectors.cor[B, 3], lcolor = "chartreuse3")

points(x = pcoa_result$vectors.cor[C, 1],

y = pcoa_result$vectors.cor[C, 3],

col = "cyan3",

pch = 19)

Plot_ConvexHull(xcoord = pcoa_result$vectors.cor[C, 1], ycoord = pcoa_result$vectors.cor[C, 3], lcolor = "cyan3")

points(x = pcoa_result$vectors.cor[D, 1],

y = pcoa_result$vectors.cor[D, 3],

col = "darkorchid1",

pch = 19)

Plot_ConvexHull(xcoord = pcoa_result$vectors.cor[D, 1], ycoord = pcoa_result$vectors.cor[D, 3], lcolor = "darkorchid1")

legend(0.27, -0.25, legend=c("Carnian", "Norian-Rhaetian","pre-Toarcian","Toarcian-middle Jurassic"),

col=c("brown1","chartreuse3","cyan3","darkorchid1"), pch=19, cex=0.70, box.lty=0,bty = "n")

#dev.off()

##Generate character groups to test different disparity patterns among different anatomical regions

skull <- c(1:120,373:377,394:403,405:417)

vertebrae <- c(121:197,390:391,404)

shoulder_girdle_and_forelimb <- c(198:244,363,367:370,378:379,392)

pelvic_girdle_and_hindlimb <- c(245:362,364:366,371:372,380:389,393)

##Prune matrix for each anatomical group

matrix_skull <- matrix

matrix_skull$matrix_1$matrix <- matrix_skull$matrix_1$matrix[,skull]

matrix_skull$matrix_1$ordering <- matrix_skull$matrix_1$ordering[skull]

matrix_skull$matrix_1$character_weights <- matrix_skull$matrix_1$character_weights[skull]

matrix_skull$matrix_1$minimum_values <- matrix_skull$matrix_1$minimum_values[skull]

matrix_skull$matrix_1$maximum_values <- matrix_skull$matrix_1$maximum_values[skull]

matrix_vertebrae <- matrix

matrix_vertebrae$matrix_1$matrix <- matrix_vertebrae$matrix_1$matrix[,vertebrae]

matrix_vertebrae$matrix_1$ordering <- matrix_vertebrae$matrix_1$ordering[vertebrae]

matrix_vertebrae$matrix_1$character_weights <- matrix_vertebrae$matrix_1$character_weights[vertebrae]

matrix_vertebrae$matrix_1$minimum_values <- matrix_vertebrae$matrix_1$minimum_values[vertebrae]

matrix_vertebrae$matrix_1$maximum_values <- matrix_vertebrae$matrix_1$maximum_values[vertebrae]

matrix_shoulder_girdle_and_forelimb <- matrix

matrix_shoulder_girdle_and_forelimb$matrix_1$matrix <- matrix_shoulder_girdle_and_forelimb$matrix_1$matrix[,shoulder_girdle_and_forelimb]

matrix_shoulder_girdle_and_forelimb$matrix_1$ordering <- matrix_shoulder_girdle_and_forelimb$matrix_1$ordering[shoulder_girdle_and_forelimb]

matrix_shoulder_girdle_and_forelimb$matrix_1$character_weights <- matrix_shoulder_girdle_and_forelimb$matrix_1$character_weights[shoulder_girdle_and_forelimb]

matrix_shoulder_girdle_and_forelimb$matrix_1$minimum_values <- matrix_shoulder_girdle_and_forelimb$matrix_1$minimum_values[shoulder_girdle_and_forelimb]

matrix_shoulder_girdle_and_forelimb$matrix_1$maximum_values <- matrix_shoulder_girdle_and_forelimb$matrix_1$maximum_values[shoulder_girdle_and_forelimb]

matrix_pelvic_girdle_and_hindlimb <- matrix

matrix_pelvic_girdle_and_hindlimb$matrix_1$matrix <- matrix_pelvic_girdle_and_hindlimb$matrix_1$matrix[,pelvic_girdle_and_hindlimb]

matrix_pelvic_girdle_and_hindlimb$matrix_1$ordering <- matrix_pelvic_girdle_and_hindlimb$matrix_1$ordering[pelvic_girdle_and_hindlimb]

matrix_pelvic_girdle_and_hindlimb$matrix_1$character_weights <- matrix_pelvic_girdle_and_hindlimb$matrix_1$character_weights[pelvic_girdle_and_hindlimb]

matrix_pelvic_girdle_and_hindlimb$matrix_1$minimum_values <- matrix_pelvic_girdle_and_hindlimb$matrix_1$minimum_values[pelvic_girdle_and_hindlimb]

matrix_pelvic_girdle_and_hindlimb$matrix_1$maximum_values <- matrix_pelvic_girdle_and_hindlimb$matrix_1$maximum_values[pelvic_girdle_and_hindlimb]

###Build MORD matrix for each character group

dist_matrix_skull <- calculate_morphological_distances(cladistic_matrix = matrix_skull,

distance_metric = "mord",

distance_transformation = "none")

dist_matrix_vertebrae <- calculate_morphological_distances(cladistic_matrix = matrix_vertebrae,

distance_metric = "mord",

distance_transformation = "none")

dist_matrix_shoulder_girdle_and_forelimb <- calculate_morphological_distances(cladistic_matrix = matrix_shoulder_girdle_and_forelimb,

distance_metric = "mord",

distance_transformation = "none")

dist_matrix_pelvic_girdle_and_hindlimb <- calculate_morphological_distances(cladistic_matrix = matrix_pelvic_girdle_and_hindlimb,

distance_metric = "mord",

distance_transformation = "none")

##Calculate WMPD (pre-ordination metric) for the skull dataset

#Prune taxa with all NAs

no.comparisons_skull.names <- names(which(colSums(dist_matrix_skull$comparable_character_matrix)==0))

no.comparisons_skull <- as.vector(which(colSums(dist_matrix_skull$comparable_character_matrix)==0))

dist_matrix_skull_reduced <- dist_matrix_skull

dist_matrix_skull_reduced$distance_matrix <- dist_matrix_skull_reduced$distance_matrix[-c(no.comparisons_skull),-c(no.comparisons_skull)]

dist_matrix_skull_reduced$comparable_character_matrix <- dist_matrix_skull_reduced$comparable_character_matrix[-c(no.comparisons_skull),-c(no.comparisons_skull)]

#Calculate WMPD for the skull

WMPD_result_skull <- WMPDBootstrap(DistanceMatrix = dist_matrix_skull_reduced,

Replicates = 9999,

list("Carnian" = setdiff(Carnian$Taxon,no.comparisons_skull.names),

"Norian_Rhaetian" = setdiff(Norian_Rhaetian$Taxon,no.comparisons_skull.names),

"pre-Toarcian" = setdiff(pre_Toarcian$Taxon,no.comparisons_skull.names),

"Toarcian-MJ" = setdiff(Toarcian_MJ$Taxon,no.comparisons_skull.names)))

#pdf(file = "graphics/WMPD_skull.pdf", width = 6, height = 6)

WMPD_result_skull$Bin <- factor(WMPD_result_skull$Bin, levels = c("Carnian","Norian_Rhaetian","pre-Toarcian","Toarcian-MJ"))

ggplot(WMPD_result_skull) +

geom_errorbar(aes(x = Bin, ymin = LowerLimit, ymax = UpperLimit, col = Bin),

width = 0.25, lwd = 1) +

geom_point(aes(x = Bin, y = WMPD, col = Bin)) +

theme_bw() +

theme(legend.position = "none")

#dev.off()

##Calculate WMPD (pre-ordination metric) for the vertebrae dataset

#Prune taxa with all NAs

no.comparisons_vertebrae.names <- names(which(colSums(dist_matrix_vertebrae$comparable_character_matrix)==0))

no.comparisons_vertebrae <- as.vector(which(colSums(dist_matrix_vertebrae$comparable_character_matrix)==0))

dist_matrix_vertebrae_reduced <- dist_matrix_vertebrae

dist_matrix_vertebrae_reduced$distance_matrix <- dist_matrix_vertebrae_reduced$distance_matrix[-c(no.comparisons_vertebrae),-c(no.comparisons_vertebrae)]

dist_matrix_vertebrae_reduced$comparable_character_matrix <- dist_matrix_vertebrae_reduced$comparable_character_matrix[-c(no.comparisons_vertebrae),-c(no.comparisons_vertebrae)]

#Calculate WMPD for the vertebrae

WMPD_result_vertebrae <- WMPDBootstrap(DistanceMatrix = dist_matrix_vertebrae_reduced,

Replicates = 9999,

list("Carnian" = setdiff(Carnian$Taxon,no.comparisons_vertebrae.names),

"Norian_Rhaetian" = setdiff(Norian_Rhaetian$Taxon,no.comparisons_vertebrae.names),

"pre-Toarcian" = setdiff(pre_Toarcian$Taxon,no.comparisons_vertebrae.names),

"Toarcian-MJ" = setdiff(Toarcian_MJ$Taxon,no.comparisons_vertebrae.names)))

#pdf(file = "graphics/WMPD_vertebrae.pdf", width = 6, height = 6)

WMPD_result_vertebrae$Bin <- factor(WMPD_result_vertebrae$Bin, levels = c("Carnian","Norian_Rhaetian","pre-Toarcian","Toarcian-MJ"))

ggplot(WMPD_result_vertebrae) +

geom_errorbar(aes(x = Bin, ymin = LowerLimit, ymax = UpperLimit, col = Bin),

width = 0.25, lwd = 1) +

geom_point(aes(x = Bin, y = WMPD, col = Bin)) +

theme_bw() +

theme(legend.position = "none")

#dev.off()

##Calculate WMPD (pre-ordination metric) for the shoulder_girdle_and_forelimb dataset

#Prune taxa with all NAs

no.comparisons_shoulder_girdle_and_forelimb.names <- names(which(colSums(dist_matrix_shoulder_girdle_and_forelimb$comparable_character_matrix)==0))

no.comparisons_shoulder_girdle_and_forelimb <- as.vector(which(colSums(dist_matrix_shoulder_girdle_and_forelimb$comparable_character_matrix)==0))

dist_matrix_shoulder_girdle_and_forelimb_reduced <- dist_matrix_shoulder_girdle_and_forelimb

dist_matrix_shoulder_girdle_and_forelimb_reduced$distance_matrix <- dist_matrix_shoulder_girdle_and_forelimb_reduced$distance_matrix[-c(no.comparisons_shoulder_girdle_and_forelimb),-c(no.comparisons_shoulder_girdle_and_forelimb)]

dist_matrix_shoulder_girdle_and_forelimb_reduced$comparable_character_matrix <- dist_matrix_shoulder_girdle_and_forelimb_reduced$comparable_character_matrix[-c(no.comparisons_shoulder_girdle_and_forelimb),-c(no.comparisons_shoulder_girdle_and_forelimb)]

#Calculate WMPD for the shoulder_girdle_and_forelimb

WMPD_result_shoulder_girdle_and_forelimb <- WMPDBootstrap(DistanceMatrix = dist_matrix_shoulder_girdle_and_forelimb_reduced,

Replicates = 9999,

list("Carnian" = setdiff(Carnian$Taxon,no.comparisons_shoulder_girdle_and_forelimb.names),

"Norian_Rhaetian" = setdiff(Norian_Rhaetian$Taxon,no.comparisons_shoulder_girdle_and_forelimb.names),

"pre-Toarcian" = setdiff(pre_Toarcian$Taxon,no.comparisons_shoulder_girdle_and_forelimb.names),

"Toarcian-MJ" = setdiff(Toarcian_MJ$Taxon,no.comparisons_shoulder_girdle_and_forelimb.names)))

#pdf(file = "graphics/WMPD_shoulder_girdle_and_forelimb.pdf", width = 6, height = 6)

WMPD_result_shoulder_girdle_and_forelimb$Bin <- factor(WMPD_result_shoulder_girdle_and_forelimb$Bin, levels = c("Carnian","Norian_Rhaetian","pre-Toarcian","Toarcian-MJ"))

ggplot(WMPD_result_shoulder_girdle_and_forelimb) +

geom_errorbar(aes(x = Bin, ymin = LowerLimit, ymax = UpperLimit, col = Bin),

width = 0.25, lwd = 1) +

geom_point(aes(x = Bin, y = WMPD, col = Bin)) +

theme_bw() +

theme(legend.position = "none")

#dev.off()

##Calculate WMPD (pre-ordination metric) for the pelvic_girdle_and_hindlimb dataset

#Prune taxa with all NAs

no.comparisons_pelvic_girdle_and_hindlimb.names <- names(which(colSums(dist_matrix_pelvic_girdle_and_hindlimb$comparable_character_matrix)==0))

no.comparisons_pelvic_girdle_and_hindlimb <- as.vector(which(colSums(dist_matrix_pelvic_girdle_and_hindlimb$comparable_character_matrix)==0))

dist_matrix_pelvic_girdle_and_hindlimb_reduced <- dist_matrix_pelvic_girdle_and_hindlimb

dist_matrix_pelvic_girdle_and_hindlimb_reduced$distance_matrix <- dist_matrix_pelvic_girdle_and_hindlimb_reduced$distance_matrix[-c(no.comparisons_pelvic_girdle_and_hindlimb),-c(no.comparisons_pelvic_girdle_and_hindlimb)]

dist_matrix_pelvic_girdle_and_hindlimb_reduced$comparable_character_matrix <- dist_matrix_pelvic_girdle_and_hindlimb_reduced$comparable_character_matrix[-c(no.comparisons_pelvic_girdle_and_hindlimb),-c(no.comparisons_pelvic_girdle_and_hindlimb)]

#Calculate WMPD for the pelvic_girdle_and_hindlimb

WMPD_result_pelvic_girdle_and_hindlimb <- WMPDBootstrap(DistanceMatrix = dist_matrix_pelvic_girdle_and_hindlimb_reduced,

Replicates = 9999,

list("Carnian" = setdiff(Carnian$Taxon,no.comparisons_pelvic_girdle_and_hindlimb.names),

"Norian_Rhaetian" = setdiff(Norian_Rhaetian$Taxon,no.comparisons_pelvic_girdle_and_hindlimb.names),

"pre-Toarcian" = setdiff(pre_Toarcian$Taxon,no.comparisons_pelvic_girdle_and_hindlimb.names),

"Toarcian-MJ" = setdiff(Toarcian_MJ$Taxon,no.comparisons_pelvic_girdle_and_hindlimb.names)))

#pdf(file = "graphics/WMPD_pelvic_girdle_and_hindlimb.pdf", width = 6, height = 6)

WMPD_result_pelvic_girdle_and_hindlimb$Bin <- factor(WMPD_result_pelvic_girdle_and_hindlimb$Bin, levels = c("Carnian","Norian_Rhaetian","pre-Toarcian","Toarcian-MJ"))

ggplot(WMPD_result_pelvic_girdle_and_hindlimb) +

geom_errorbar(aes(x = Bin, ymin = LowerLimit, ymax = UpperLimit, col = Bin),

width = 0.25, lwd = 1) +

geom_point(aes(x = Bin, y = WMPD, col = Bin)) +

theme_bw() +

theme(legend.position = "none")

#dev.off()

#Calculate WMPD (pre-ordination metric) for the complete dataset

WMPD_result <- WMPDBootstrap(DistanceMatrix = dist_matrix,

Replicates = 9999,

list("Carnian" = Carnian$Taxon,

"Norian_Rhaetian" = Norian_Rhaetian$Taxon,

"pre-Toarcian" = pre_Toarcian$Taxon,

"Toarcian-MJ" = Toarcian_MJ$Taxon))

#pdf(file = "graphics/WMPD.pdf", width = 6, height = 6)

WMPD_result$Bin <- factor(WMPD_result$Bin, levels = c("Carnian","Norian_Rhaetian","pre-Toarcian","Toarcian-MJ"))

ggplot(WMPD_result) +

geom_errorbar(aes(x = Bin, ymin = LowerLimit, ymax = UpperLimit, col = Bin),

width = 0.25, lwd = 1) +

geom_point(aes(x = Bin, y = WMPD, col = Bin)) +

theme_bw() +

theme(legend.position = "none")

#dev.off()

#Calculate WMPD (pre-ordination metric) without Barapasaurus and Isanosaurus

Barapa_Isano <- c("Barapasaurus","Isanosaurus")

Carnian_Barapa_Isano <- setdiff(Carnian$Taxon, Barapa_Isano)

Norian_Rhaetian_Barapa_Isano <- setdiff(Norian_Rhaetian$Taxon, Barapa_Isano)

pre_Toarcian_Barapa_Isano <- setdiff(pre_Toarcian$Taxon, Barapa_Isano)

Toarcian_MJ_Barapa_Isano <- setdiff(Toarcian_MJ$Taxon, Barapa_Isano)

WMPD_result_Barapa_Isano <- WMPDBootstrap(DistanceMatrix = dist_matrix,

Replicates = 9999,

list("Carnian" = Carnian_Barapa_Isano,

"Norian_Rhaetian" = Norian_Rhaetian_Barapa_Isano,

"pre-Toarcian" = pre_Toarcian_Barapa_Isano,

"Toarcian-MJ" = Toarcian_MJ_Barapa_Isano))

#pdf(file = "graphics/WMPD_without_Barapa_Isano.pdf", width = 6, height = 6)

WMPD_result_Barapa_Isano$Bin <- factor(WMPD_result_Barapa_Isano$Bin, levels = c("Carnian","Norian_Rhaetian","pre-Toarcian","Toarcian-MJ"))

ggplot(WMPD_result_Barapa_Isano) +

geom_errorbar(aes(x = Bin, ymin = LowerLimit, ymax = UpperLimit, col = Bin),

width = 0.25, lwd = 1) +

geom_point(aes(x = Bin, y = WMPD, col = Bin)) +

theme_bw() +

theme(legend.position = "none")

#dev.off()

meanagesWMPD <- c(229.4,213.5,192.0,167.4)

#pdf(file = "graphics/WMPD_without_Barapa_Isano_geoscale.pdf", width = 6, height = 6)

geoscalePlot(data=WMPD_result_Barapa_Isano[,2], ages=meanagesWMPD, units=c("Age" ,"Epoch", "Period"),

boxes="Age", age.lim=c(233.5,150),pch = 21, cex.age = 0.8,cex.ts = 0.8, data.lim=c(min(WMPD_result_Barapa_Isano$LowerLimit),max(WMPD_result_Barapa_Isano$UpperLimit)),

label="WMPD with 95% confidence interval")

lines(rbind(meanagesWMPD, meanagesWMPD, NA),rbind(WMPD_result_Barapa_Isano[,3],WMPD_result_Barapa_Isano[,4],NA))

#dev.off()

#Calculate WMPD (pre-ordination metric) without Barapasaurus and Isanosaurus and without

#the taxa that were trimmed for the ordination of the dataset

Carnian_Barapa_Isano_trimmed <- setdiff(Carnian$Taxon,

c(Barapa_Isano, trim_dist_matrix$removed_taxa))

Norian_Rhaetian_Barapa_Isano_trimmed <- setdiff(Norian_Rhaetian$Taxon,

c(Barapa_Isano, trim_dist_matrix$removed_taxa))

pre_Toarcian_Barapa_Isano_trimmed <- setdiff(pre_Toarcian$Taxon,

c(Barapa_Isano, trim_dist_matrix$removed_taxa))

Toarcian_MJ_Barapa_Isano_trimmed <- setdiff(Toarcian_MJ$Taxon,

c(Barapa_Isano, trim_dist_matrix$removed_taxa))

WMPD_result_Barapa_Isano_trimmed <- WMPDBootstrap(DistanceMatrix = dist_matrix,

Replicates = 9999,

list("Carnian" = Carnian_Barapa_Isano_trimmed,

"Norian_Rhaetian" = Norian_Rhaetian_Barapa_Isano_trimmed,

"pre-Toarcian" = pre_Toarcian_Barapa_Isano_trimmed,

"Toarcian-MJ" = Toarcian_MJ_Barapa_Isano_trimmed))

#pdf(file = "graphics/WMPD_without_Barapa_Isano_trimmed.pdf", width = 6, height = 6)

WMPD_result_Barapa_Isano_trimmed$Bin <- factor(WMPD_result_Barapa_Isano_trimmed$Bin, levels = c("Carnian","Norian_Rhaetian","pre-Toarcian","Toarcian-MJ"))

ggplot(WMPD_result_Barapa_Isano_trimmed) +

geom_errorbar(aes(x = Bin, ymin = LowerLimit, ymax = UpperLimit, col = Bin),

width = 0.25, lwd = 1) +

geom_point(aes(x = Bin, y = WMPD, col = Bin)) +

theme_bw() +

theme(legend.position = "none")

#dev.off()

#Generate one subset of biped taxa and another of quadruped taxa

bipeds <- na.omit(data$Taxon[data$Locomotion=="Biped"])

quadrupeds <- na.omit(data$Taxon[data$Locomotion=="Quadruped"])

bipeds.PCoA <- intersect(bipeds,

rownames(pcoa_result$vectors.cor))

quadrupeds.PCoA <- intersect(quadrupeds,

rownames(pcoa_result$vectors.cor))

locomotions <- list(bipeds.PCoA,quadrupeds.PCoA)

#Generate one subset bipeds versus quadruped taxa but based on discriminant analysis inference

bipeds_red <- na.omit(data$Taxon[data$Locomotion_red=="Biped"])

quadrupeds_red <- na.omit(data$Taxon[data$Locomotion_red=="Quadruped"])

bipeds.PCoA_red <- intersect(bipeds_red,

rownames(pcoa_result$vectors.cor))

quadrupeds.PCoA_red <- intersect(quadrupeds_red,

rownames(pcoa_result$vectors.cor))

locomotions_red <- list(bipeds.PCoA_red,quadrupeds.PCoA_red)

#Calculate WMPD for different types of locomotion

WMPD_result_locomotion <- WMPDBootstrap(DistanceMatrix = dist_matrix,

Replicates = 9999,

list("Bipeds" = bipeds,

"Quadrupeds" = quadrupeds))

#pdf(file = "graphics/WMPD_locomotion.pdf", width = 6, height = 6)

WMPD_result_locomotion$Bin <- factor(WMPD_result_locomotion$Bin, levels = c("Bipeds","Quadrupeds"))

ggplot(WMPD_result_locomotion) +

geom_errorbar(aes(x = Bin, ymin = LowerLimit, ymax = UpperLimit, col = Bin),

width = 0.25, lwd = 1) +

geom_point(aes(x = Bin, y = WMPD, col = Bin)) +

theme_bw() +

theme(legend.position = "none")

#dev.off()

#Calculate WMPD for different types of locomotion reduced sample

WMPD_result_locomotion_red <- WMPDBootstrap(DistanceMatrix = dist_matrix,

Replicates = 9999,

list("Bipeds" = bipeds_red,

"Quadrupeds" = quadrupeds_red))

#pdf(file = "graphics/WMPD_locomotion_reduced_sample.pdf", width = 6, height = 6)

WMPD_result_locomotion_red$Bin <- factor(WMPD_result_locomotion_red$Bin, levels = c("Bipeds","Quadrupeds"))

ggplot(WMPD_result_locomotion_red) +

geom_errorbar(aes(x = Bin, ymin = LowerLimit, ymax = UpperLimit, col = Bin),

width = 0.25, lwd = 1) +

geom_point(aes(x = Bin, y = WMPD, col = Bin)) +

theme_bw() +

theme(legend.position = "none")

#dev.off()

#Calculate WMPD for different types of locomotion without Barpasaurus and Isanosaurus and with reduced sample

bipeds_Barapa_Isano_red <- setdiff(bipeds_red,c("Barapasaurus","Isanosaurus"))

quadrupeds_Barapa_Isano_red <- setdiff(quadrupeds_red,c("Barapasaurus","Isanosaurus"))

WMPD_result_locomotion_Barapa_Isano_red <- WMPDBootstrap(DistanceMatrix = dist_matrix,

Replicates = 9999,

list("Bipeds" = bipeds_Barapa_Isano_red,

"Quadrupeds" = quadrupeds_Barapa_Isano_red))

#pdf(file = "graphics/WMPD_locomotion_without_Barapasaurus_and_Isanosaurus_and_reduced_sample.pdf", width = 6, height = 6)

WMPD_result_locomotion_Barapa_Isano_red$Bin <- factor(WMPD_result_locomotion_Barapa_Isano_red$Bin, levels = c("Bipeds","Quadrupeds"))

ggplot(WMPD_result_locomotion_Barapa_Isano_red) +

geom_errorbar(aes(x = Bin, ymin = LowerLimit, ymax = UpperLimit, col = Bin),

width = 0.25, lwd = 1) +

geom_point(aes(x = Bin, y = WMPD, col = Bin)) +

theme_bw() +

theme(legend.position = "none")

#dev.off()

#Generate new set without Barapsaurus and Isanosaurus

A_Barapa_Isano <- setdiff(A, Barapa_Isano)

B_Barapa_Isano <- setdiff(B, Barapa_Isano)

C_Barapa_Isano <- setdiff(C, Barapa_Isano)

D_Barapa_Isano <- setdiff(D, Barapa_Isano)

bins.trim_Barapa_Isano <- list(A_Barapa_Isano, B_Barapa_Isano, C_Barapa_Isano, D_Barapa_Isano)

pcoa_result_Barapa_Isano <- pcoa_result

pcoa_result_Barapa_Isano$vectors.cor <- pcoa_result$vectors.cor[setdiff(names(pcoa_result$vectors.cor[,1]),Barapa_Isano),]

#Test different positions in morphospace

cat_Carnian_Norian <- c(rep(1,each=length(A_Barapa_Isano)),rep(2,each=length(B_Barapa_Isano)))

cat_Norian_preToarcian <- c(rep(1,each=length(B_Barapa_Isano)),rep(2,each=length(C_Barapa_Isano)))

cat_preToarcian_Toarcian <- c(rep(1,each=length(C_Barapa_Isano)),rep(2,each=length(D_Barapa_Isano)))

cat_Carnian_Toarcian <- c(rep(1,each=length(A_Barapa_Isano)),rep(2,each=length(D_Barapa_Isano)))

cat_Norian_Toarcian <- c(rep(1,each=length(B_Barapa_Isano)),rep(2,each=length(D_Barapa_Isano)))

permanova_Carnian_Norian <- adonis(rbind(pcoa_result_Barapa_Isano$vectors.cor[A_Barapa_Isano,1:17],pcoa_result_Barapa_Isano$vectors.cor[B_Barapa_Isano,1:17])~cat_Carnian_Norian,

permutations=9999,

method="euclidean")

permanova_Norian_preToarcian <- adonis(rbind(pcoa_result_Barapa_Isano$vectors.cor[B_Barapa_Isano,1:17],pcoa_result_Barapa_Isano$vectors.cor[C_Barapa_Isano,1:17])~cat_Norian_preToarcian,

permutations=9999,

method="euclidean")

permanova_preToarcian_Toarcian <- adonis(rbind(pcoa_result_Barapa_Isano$vectors.cor[C_Barapa_Isano,1:17],pcoa_result_Barapa_Isano$vectors.cor[D_Barapa_Isano,1:17])~cat_preToarcian_Toarcian,

permutations=9999,

method="euclidean")

permanova_Carnian_Toarcian <- adonis(rbind(pcoa_result_Barapa_Isano$vectors.cor[A_Barapa_Isano,1:17],pcoa_result_Barapa_Isano$vectors.cor[D_Barapa_Isano,1:17])~cat_Carnian_Toarcian,

permutations=9999,

method="euclidean")

permanova_Norian_Toarcian <- adonis(rbind(pcoa_result_Barapa_Isano$vectors.cor[B_Barapa_Isano,1:17],pcoa_result_Barapa_Isano$vectors.cor[D_Barapa_Isano,1:17])~cat_Norian_Toarcian,

permutations=9999,

method="euclidean")

##Explore how different metrics capture morphospace traits

sum_variances_test <- test.metric(pcoa_result$vectors.cor[,1:17], metric = c(sum, variances),

shifts = c("random", "size", "density", "position"))

summary(sum_variances_test)

#pdf(file = "graphics/SoV_test.pdf", width = 6, height = 6)

plot(sum_variances_test)

#dev.off()

sum_ranges_test <- test.metric(pcoa_result$vectors.cor[,1:17], metric = c(sum, ranges),

shifts = c("random", "size", "density", "position"))

summary(sum_ranges_test)

#pdf(file = "graphics/SoR_test.pdf", width = 6, height = 6)

plot(sum_ranges_test)

#dev.off()

displacements_test <- test.metric(pcoa_result$vectors.cor[,1:17], metric = c(mean, displacements),

shifts = c("random", "size", "density", "position"))

summary(displacements_test)

#pdf(file = "graphics/Displacements_test.pdf", width = 6, height = 6)

plot(displacements_test)

#dev.off()

####################

#Calculate Sum of Variances

sov_result <- sov_bootstrap(Vectors = pcoa_result$vectors.cor[,1:17],

Replicates = 9999,

Bins = list("Carnian" = A,

"Norian_Rhaetian" = B,

"pre-Toarcian" = C,

"Toarcian-MJ" = D))

#pdf(file = "graphics/SoV.pdf", width = 6, height = 6)

sov_result$Bin <- factor(sov_result$Bin, levels = c("Carnian","Norian_Rhaetian","pre-Toarcian","Toarcian-MJ"))

ggplot(sov_result) +

geom_errorbar(aes(x = Bin, ymin = LowerLimit, ymax = UpperLimit, col = Bin),

width = 0.25, lwd = 1) +

geom_point(aes(x = Bin, y = SoV, col = Bin)) +

theme_bw() +

theme(legend.position = "none")

#dev.off()

#Calculate Sum of Variances with rarefied matrices

SoV_rarefied <- list()

for (i in 1:length(bins.trim)) {

SoV_rarefied[[i]] <- sum(variances(pcoa_result$vectors.cor[bins.trim[[i]], 1:17]))

}

boot_SoV_rarefied <- list()

SoV_ds_rarefied <- list()

q_SoV_rarefied <- list()

for (j in 1:length(bins.trim)) {

boot_SoV_rarefied[[j]] <- boot.matrix(data = pcoa_result$vectors.cor[bins.trim[[j]], 1:17],

bootstraps = 9999,

rarefaction = 7)

SoV_ds_rarefied[[j]] <- apply(X = boot_SoV_rarefied[[j]]$subsets[[1]][[2]],

MARGIN = 2,

FUN = function(x) sum(variances(pcoa_result$vectors.cor[bins.trim[[j]], ][x, 1:17])))

q_SoV_rarefied[[j]] <- sort(quantile(SoV_ds_rarefied[[j]], c(0.05, 1), names = FALSE))

}

SoV_result_rarefied <- data.frame(Bin = c("Carnian", "Norian-Rhaetian", "pre-Toarcian", "Toarcian-MJ"),

SoV = c(SoV_rarefied[[1]], SoV_rarefied[[2]], SoV_rarefied[[3]], SoV_rarefied[[4]]),

LowerLimit = c(q_SoV_rarefied[[1]][1], q_SoV_rarefied[[2]][1], q_SoV_rarefied[[3]][1], q_SoV_rarefied[[4]][1]),

UpperLimit = c(q_SoV_rarefied[[1]][2], q_SoV_rarefied[[2]][2], q_SoV_rarefied[[3]][2], q_SoV_rarefied[[4]][2]))

#pdf(file = "graphics/SoV_rarefied.pdf", width = 6, height = 6)

SoV_result_rarefied$Bin <- factor(SoV_result_rarefied$Bin, levels = c("Carnian","Norian-Rhaetian","pre-Toarcian","Toarcian-MJ"))

ggplot(SoV_result_rarefied) +

geom_errorbar(aes(x = Bin, ymin = LowerLimit, ymax = UpperLimit, col = Bin),

width = 0.25, lwd = 1) +

geom_point(aes(x = Bin, y = SoV, col = Bin)) +

theme_bw() +

theme(legend.position = "none")

#dev.off()

#Calculate Sum of Ranges

SoR <- list()

for (i in 1:length(bins.trim)) {

SoR[[i]] <- sum(ranges(pcoa_result$vectors.cor[bins.trim[[i]], 1:17]))

}

boot_SoR <- list()

SoR_ds <- list()

q_SoR <- list()

for (j in 1:length(bins.trim)) {

boot_SoR[[j]] <- boot.matrix(data = pcoa_result$vectors.cor[bins.trim[[j]], 1:17],

bootstraps = 9999)

SoR_ds[[j]] <- apply(X = boot_SoR[[j]]$subsets[[1]][[2]],

MARGIN = 2,

FUN = function(x) sum(ranges(pcoa_result$vectors.cor[bins.trim[[j]], ][x, 1:17])))

q_SoR[[j]] <- sort(quantile(SoR_ds[[j]], c(0.05, 1), names = FALSE))

}

SoR_result <- data.frame(Bin = c("Carnian", "Norian-Rhaetian", "pre-Toarcian", "Toarcian-MJ"),

SoR = c(SoR[[1]], SoR[[2]], SoR[[3]], SoR[[4]]),

LowerLimit = c(q_SoR[[1]][1], q_SoR[[2]][1], q_SoR[[3]][1], q_SoR[[4]][1]),

UpperLimit = c(q_SoR[[1]][2], q_SoR[[2]][2], q_SoR[[3]][2], q_SoR[[4]][2]))

#pdf(file = "graphics/SoR.pdf", width = 6, height = 6)

SoR_result$Bin <- factor(SoR_result$Bin, levels = c("Carnian","Norian-Rhaetian","pre-Toarcian","Toarcian-MJ"))

ggplot(SoR_result) +

geom_errorbar(aes(x = Bin, ymin = LowerLimit, ymax = UpperLimit, col = Bin),

width = 0.25, lwd = 1) +

geom_point(aes(x = Bin, y = SoR, col = Bin)) +

theme_bw() +

theme(legend.position = "none")

#dev.off()

#Calculate Sum of Ranges with rarefied matrices

boot_SoR_rarefied <- list()

SoR_ds_rarefied <- list()

q_SoR_rarefied <- list()

for (j in 1:length(bins.trim)) {

boot_SoR_rarefied[[j]] <- boot.matrix(data = pcoa_result$vectors.cor[bins.trim[[j]], 1:17],

bootstraps = 9999,

rarefaction = 7)

SoR_ds_rarefied[[j]] <- apply(X = boot_SoR_rarefied[[j]]$subsets[[1]][[2]],

MARGIN = 2,

FUN = function(x) sum(ranges(pcoa_result$vectors.cor[bins.trim[[j]], ][x, 1:17])))

q_SoR_rarefied[[j]] <- sort(quantile(SoR_ds_rarefied[[j]], c(0.05, 1), names = FALSE))

}

SoR_result_rarefied <- data.frame(Bin = c("Carnian", "Norian-Rhaetian", "pre-Toarcian", "Toarcian-MJ"),

SoR = c(SoR[[1]], SoR[[2]], SoR[[3]], SoR[[4]]),

LowerLimit = c(q_SoR_rarefied[[1]][1], q_SoR_rarefied[[2]][1], q_SoR_rarefied[[3]][1], q_SoR_rarefied[[4]][1]),

UpperLimit = c(q_SoR_rarefied[[1]][2], q_SoR_rarefied[[2]][2], q_SoR_rarefied[[3]][2], q_SoR_rarefied[[4]][2]))

#pdf(file = "graphics/SoR_rarefied.pdf", width = 6, height = 6)

SoR_result_rarefied$Bin <- factor(SoR_result_rarefied$Bin, levels = c("Carnian","Norian-Rhaetian","pre-Toarcian","Toarcian-MJ"))

ggplot(SoR_result_rarefied) +

geom_errorbar(aes(x = Bin, ymin = LowerLimit, ymax = UpperLimit, col = Bin),

width = 0.25, lwd = 1) +

geom_point(aes(x = Bin, y = SoR, col = Bin)) +

theme_bw() +

theme(legend.position = "none")

#dev.off()

#Calculate Sum of Variance without Barapasaurus and Isanosaurus

sov_result_Barapa_Isano <- sov_bootstrap(Vectors = pcoa_result_Barapa_Isano$vectors.cor[,1:17],

Replicates = 9999,

Bins = list("Carnian" = A_Barapa_Isano,

"Norian_Rhaetian" = B_Barapa_Isano,

"pre-Toarcian" = C_Barapa_Isano,

"Toarcian-MJ" = D_Barapa_Isano))

#pdf(file = "graphics/SoV_without_Barapasaurus_and_Isanosaurus.pdf", width = 6, height = 6)

sov_result_Barapa_Isano$Bin <- factor(sov_result_Barapa_Isano$Bin, levels = c("Carnian","Norian_Rhaetian","pre-Toarcian","Toarcian-MJ"))

ggplot(sov_result_Barapa_Isano) +

geom_errorbar(aes(x = Bin, ymin = LowerLimit, ymax = UpperLimit, col = Bin),

width = 0.25, lwd = 1) +

geom_point(aes(x = Bin, y = SoV, col = Bin)) +

theme_bw() +

theme(legend.position = "none")

#dev.off()

#Calculate Sum of Ranges without Barapasaurus and Isanosaurus

SoR_Barapa_Isano <- list()

for (i in 1:length(bins.trim)) {

SoR_Barapa_Isano[[i]] <- sum(ranges(pcoa_result_Barapa_Isano$vectors.cor[bins.trim_Barapa_Isano[[i]], 1:17]))

}

boot_SoR_Barapa_Isano <- list()

SoR_ds_Barapa_Isano <- list()

q_SoR_Barapa_Isano <- list()

for (j in 1:length(bins.trim)) {

boot_SoR_Barapa_Isano[[j]] <- boot.matrix(data = pcoa_result_Barapa_Isano$vectors.cor[bins.trim_Barapa_Isano[[j]], 1:17],

bootstraps = 9999)

SoR_ds_Barapa_Isano[[j]] <- apply(X = boot_SoR_Barapa_Isano[[j]]$subsets[[1]][[2]],

MARGIN = 2,

FUN = function(x) sum(ranges(pcoa_result_Barapa_Isano$vectors.cor[bins.trim_Barapa_Isano[[j]], ][x, 1:17])))

q_SoR_Barapa_Isano[[j]] <- sort(quantile(SoR_ds_Barapa_Isano[[j]], c(0.05, 1), names = FALSE))

}

SoR_result_Barapa_Isano <- data.frame(Bin = c("Carnian", "Norian-Rhaetian", "pre-Toarcian", "Toarcian-MJ"),

SoR = c(SoR_Barapa_Isano[[1]], SoR_Barapa_Isano[[2]], SoR_Barapa_Isano[[3]], SoR_Barapa_Isano[[4]]),

LowerLimit = c(q_SoR_Barapa_Isano[[1]][1], q_SoR_Barapa_Isano[[2]][1], q_SoR_Barapa_Isano[[3]][1], q_SoR_Barapa_Isano[[4]][1]),

UpperLimit = c(q_SoR_Barapa_Isano[[1]][2], q_SoR_Barapa_Isano[[2]][2], q_SoR_Barapa_Isano[[3]][2], q_SoR_Barapa_Isano[[4]][2]))

#pdf(file = "graphics/SoR_without_Barapasaurus_and_Isanosaurus.pdf", width = 6, height = 6)

SoR_result_Barapa_Isano$Bin <- factor(SoR_result_Barapa_Isano$Bin, levels = c("Carnian","Norian-Rhaetian","pre-Toarcian","Toarcian-MJ"))

ggplot(SoR_result_Barapa_Isano) +

geom_errorbar(aes(x = Bin, ymin = LowerLimit, ymax = UpperLimit, col = Bin),

width = 0.25, lwd = 1) +

geom_point(aes(x = Bin, y = SoR, col = Bin)) +

theme_bw() +

theme(legend.position = "none")

#dev.off()

#Calculate Sum of Variance comparing locomotions

sov_result_locomotion <- sov_bootstrap(Vectors = pcoa_result$vectors.cor[,1:17],

Replicates = 9999,

Bins = list("Bipeds" = bipeds.PCoA,

"Quadrupeds" = quadrupeds.PCoA))

#pdf(file = "graphics/SoV_locomotion.pdf", width = 6, height = 6)

sov_result_locomotion$Bin <- factor(sov_result_locomotion$Bin, levels = c("Bipeds","Quadrupeds"))

ggplot(sov_result_locomotion) +

geom_errorbar(aes(x = Bin, ymin = LowerLimit, ymax = UpperLimit, col = Bin),

width = 0.25, lwd = 1) +

geom_point(aes(x = Bin, y = SoV, col = Bin)) +

theme_bw() +

theme(legend.position = "none")

#dev.off()

#Calcuate Sum of Ranges for locomotion styles

SoR_locomotion <- list()

for (i in 1:length(locomotions)) {

SoR_locomotion[[i]] <- sum(ranges(pcoa_result$vectors.cor[locomotions[[i]], 1:17]))

}

boot_SoR_locomotion <- list()

SoR_ds_locomotion <- list()

q_SoR_locomotion <- list()

for (j in 1:length(locomotions)) {

boot_SoR_locomotion[[j]] <- boot.matrix(data = pcoa_result$vectors.cor[locomotions[[j]], 1:17],

bootstraps = 9999)

SoR_ds_locomotion[[j]] <- apply(X = boot_SoR_locomotion[[j]]$subsets[[1]][[2]],

MARGIN = 2,

FUN = function(x) sum(ranges(pcoa_result$vectors.cor[locomotions[[j]], ][x, 1:17])))

q_SoR_locomotion[[j]] <- sort(quantile(SoR_ds_locomotion[[j]], c(0.05, 1), names = FALSE))

}

SoR_result_locomotion <- data.frame(Bin = c("Bipeds", "Quadrupeds"),

SoR = c(SoR_locomotion[[1]], SoR_locomotion[[2]]),

LowerLimit = c(q_SoR_locomotion[[1]][1], q_SoR_locomotion[[2]][1]),

UpperLimit = c(q_SoR_locomotion[[1]][2], q_SoR_locomotion[[2]][2]))

#pdf(file = "graphics/SoR_locomotion.pdf", width = 6, height = 6)

SoR_result_locomotion$Bin <- factor(SoR_result_locomotion$Bin, levels = c("Bipeds", "Quadrupeds"))

ggplot(SoR_result_locomotion) +

geom_errorbar(aes(x = Bin, ymin = LowerLimit, ymax = UpperLimit, col = Bin),

width = 0.25, lwd = 1) +

geom_point(aes(x = Bin, y = SoR, col = Bin)) +

theme_bw() +

theme(legend.position = "none")

#dev.off()

#Calculate Sum of Variance comparing locomotions with reduced sample

sov_result_locomotion_red <- sov_bootstrap(Vectors = pcoa_result$vectors.cor[,1:17],

Replicates = 9999,

Bins = list("Bipeds" = bipeds.PCoA_red,

"Quadrupeds" = quadrupeds.PCoA_red))

#pdf(file = "graphics/SoV_locomotion_reduced_sample.pdf", width = 6, height = 6)

sov_result_locomotion_red$Bin <- factor(sov_result_locomotion_red$Bin, levels = c("Bipeds","Quadrupeds"))

ggplot(sov_result_locomotion_red) +

geom_errorbar(aes(x = Bin, ymin = LowerLimit, ymax = UpperLimit, col = Bin),

width = 0.25, lwd = 1) +

geom_point(aes(x = Bin, y = SoV, col = Bin)) +

theme_bw() +

theme(legend.position = "none")

#dev.off()

#Calculate Sum of Ranges comparing locomotions with reduced sample

SoR_locomotion_red <- list()

for (i in 1:length(locomotions_red)) {

SoR_locomotion_red[[i]] <- sum(ranges(pcoa_result$vectors.cor[locomotions_red[[i]], 1:17]))

}

boot_SoR_locomotion_red <- list()

SoR_ds_locomotion_red <- list()

q_SoR_locomotion_red <- list()

for (j in 1:length(locomotions_red)) {

boot_SoR_locomotion_red[[j]] <- boot.matrix(data = pcoa_result$vectors.cor[locomotions_red[[j]], 1:17],

bootstraps = 9999)

SoR_ds_locomotion_red[[j]] <- apply(X = boot_SoR_locomotion_red[[j]]$subsets[[1]][[2]],

MARGIN = 2,

FUN = function(x) sum(ranges(pcoa_result$vectors.cor[locomotions_red[[j]], ][x, 1:17])))

q_SoR_locomotion_red[[j]] <- sort(quantile(SoR_ds_locomotion_red[[j]], c(0.05, 1), names = FALSE))

}

SoR_result_locomotion_red <- data.frame(Bin = c("Bipeds", "Quadrupeds"),

SoR = c(SoR_locomotion_red[[1]], SoR_locomotion_red[[2]]),

LowerLimit = c(q_SoR_locomotion_red[[1]][1], q_SoR_locomotion_red[[2]][1]),

UpperLimit = c(q_SoR_locomotion_red[[1]][2], q_SoR_locomotion_red[[2]][2]))

#pdf(file = "graphics/SoR_locomotion_reduced_sample.pdf", width = 6, height = 6)

SoR_result_locomotion_red$Bin <- factor(SoR_result_locomotion_red$Bin, levels = c("Bipeds", "Quadrupeds"))

ggplot(SoR_result_locomotion_red) +

geom_errorbar(aes(x = Bin, ymin = LowerLimit, ymax = UpperLimit, col = Bin),

width = 0.25, lwd = 1) +

geom_point(aes(x = Bin, y = SoR, col = Bin)) +

theme_bw() +

theme(legend.position = "none")

#dev.off()

#Calculate displacements from centroid

displ <- list()

for (i in 1:length(bins.trim)) {

displ[[i]] <- mean(displacements(pcoa_result$vectors.cor[bins.trim[[i]], 1:17]))

}

boot_displ <- list()

disp_ds <- list()

q_displ <- list()

for (j in 1:length(bins.trim)) {

boot_displ[[j]] <- boot.matrix(data = pcoa_result$vectors.cor[bins.trim[[j]], 1:17],

bootstraps = 9999,rarefaction = "min")

disp_ds[[j]] <- apply(X = boot_displ[[j]]$subsets[[1]][[2]],

MARGIN = 2,

FUN = function(x) mean(displacements(pcoa_result$vectors.cor[bins.trim[[j]], ][x, 1:17])))

q_displ[[j]] <- sort(quantile(disp_ds[[j]], c(0.025, 0.975), names = FALSE))

}

Displacements_result <- data.frame(Bin = c("Carnian", "Norian-Rhaetian", "pre-Toarcian", "Toarcian-MJ"),

Disp = c(displ[[1]], displ[[2]], displ[[3]], displ[[4]]),

LowerLimit = c(q_displ[[1]][1], q_displ[[2]][1], q_displ[[3]][1], q_displ[[4]][1]),

UpperLimit = c(q_displ[[1]][2], q_displ[[2]][2], q_displ[[3]][2], q_displ[[4]][2]))

#pdf(file = "graphics/Displacements_from_centroid.pdf", width = 6, height = 6)

Displacements_result$Bin <- factor(Displacements_result$Bin, levels = c("Carnian","Norian-Rhaetian","pre-Toarcian","Toarcian-MJ"))

ggplot(Displacements_result) +

geom_errorbar(aes(x = Bin, ymin = LowerLimit, ymax = UpperLimit, col = Bin),

width = 0.25, lwd = 1) +

geom_point(aes(x = Bin, y = Disp, col = Bin)) +

theme_bw() +

theme(legend.position = "none")

#dev.off()

#Calculate displacements from centroid with rarefied matrices

displ_rarefied <- list()

for (i in 1:length(bins.trim)) {

displ_rarefied[[i]] <- mean(displacements(pcoa_result$vectors.cor[bins.trim[[i]], 1:17]))

}

boot_displ_rarefied <- list()

disp_rarefied_ds <- list()

q_displ_rarefied <- list()

for (j in 1:length(bins.trim)) {

boot_displ_rarefied[[j]] <- boot.matrix(data = pcoa_result$vectors.cor[bins.trim[[j]], 1:17],

bootstraps = 9999,

rarefaction = 7)

disp_rarefied_ds[[j]] <- apply(X = boot_displ_rarefied[[j]]$subsets[[1]][[2]],

MARGIN = 2,

FUN = function(x) mean(displacements(pcoa_result$vectors.cor[bins.trim[[j]], ][x, 1:17])))

q_displ_rarefied[[j]] <- sort(quantile(disp_ds[[j]], c(0.025, 0.975), names = FALSE))

}

displacements_rarefied_result <- data.frame(Bin = c("Carnian", "Norian-Rhaetian", "pre-Toarcian", "Toarcian-MJ"),

Disp = c(displ_rarefied[[1]], displ_rarefied[[2]], displ_rarefied[[3]], displ_rarefied[[4]]),

LowerLimit = c(q_displ_rarefied[[1]][1], q_displ_rarefied[[2]][1], q_displ_rarefied[[3]][1], q_displ_rarefied[[4]][1]),

UpperLimit = c(q_displ_rarefied[[1]][2], q_displ_rarefied[[2]][2], q_displ_rarefied[[3]][2], q_displ_rarefied[[4]][2]))

#pdf(file = "graphics/displacements_from_centroid_rarefied_result.pdf", width = 6, height = 6)

displacements_rarefied_result$Bin <- factor(displacements_rarefied_result$Bin, levels = c("Carnian","Norian-Rhaetian","pre-Toarcian","Toarcian-MJ"))

ggplot(displacements_rarefied_result) +

geom_errorbar(aes(x = Bin, ymin = LowerLimit, ymax = UpperLimit, col = Bin),

width = 0.25, lwd = 1) +

geom_point(aes(x = Bin, y = Disp, col = Bin)) +

theme_bw() +

theme(legend.position = "none")

#dev.off()

#Calculate displacements from centroid without Barapasaurus and Isanosaurus

displ_Barapa_Isano <- list()

for (i in 1:length(bins.trim_Barapa_Isano)) {

displ_Barapa_Isano[[i]] <- mean(displacements(pcoa_result_Barapa_Isano$vectors.cor[bins.trim_Barapa_Isano[[i]], 1:17]))

}

boot_displ_Barapa_Isano <- list()

disp_ds_Barapa_Isano <- list()

q_displ_Barapa_Isano <- list()

for (j in 1:length(bins.trim_Barapa_Isano)) {

boot_displ_Barapa_Isano[[j]] <- boot.matrix(data = pcoa_result_Barapa_Isano$vectors.cor[bins.trim_Barapa_Isano[[j]], 1:17],

bootstraps = 9999)

disp_ds_Barapa_Isano[[j]] <- apply(X = boot_displ_Barapa_Isano[[j]]$subsets[[1]][[2]],

MARGIN = 2,

FUN = function(x) mean(displacements(pcoa_result_Barapa_Isano$vectors.cor[bins.trim_Barapa_Isano[[j]], ][x, 1:17])))

q_displ_Barapa_Isano[[j]] <- sort(quantile(disp_ds_Barapa_Isano[[j]], c(0.025, 0.975), names = FALSE))

}

Displacements_result_Barapa_Isano <- data.frame(Bin = c("Carnian", "Norian-Rhaetian", "pre-Toarcian", "Toarcian-MJ"),

Disp = c(displ_Barapa_Isano[[1]], displ_Barapa_Isano[[2]], displ_Barapa_Isano[[3]], displ_Barapa_Isano[[4]]),

LowerLimit = c(q_displ_Barapa_Isano[[1]][1], q_displ_Barapa_Isano[[2]][1], q_displ_Barapa_Isano[[3]][1], q_displ_Barapa_Isano[[4]][1]),

UpperLimit = c(q_displ_Barapa_Isano[[1]][2], q_displ_Barapa_Isano[[2]][2], q_displ_Barapa_Isano[[3]][2], q_displ_Barapa_Isano[[4]][2]))

#pdf(file = "graphics/Displacements_from_centroid_without_Barapasaurus_and_Isanosaurus.pdf", width = 6, height = 6)

Displacements_result_Barapa_Isano$Bin <- factor(Displacements_result_Barapa_Isano$Bin, levels = c("Carnian","Norian-Rhaetian","pre-Toarcian","Toarcian-MJ"))

ggplot(Displacements_result_Barapa_Isano) +

geom_errorbar(aes(x = Bin, ymin = LowerLimit, ymax = UpperLimit, col = Bin),

width = 0.25, lwd = 1) +

geom_point(aes(x = Bin, y = Disp, col = Bin)) +

theme_bw() +

theme(legend.position = "none")

#dev.off()

#Displacements from the centroid of previous time bin

centroid <- list()

for (k in 1:length(bins.trim)) {

centroid[[k]] <- apply(pcoa_result$vectors.cor[bins.trim[[k]], 1:17], 2, mean)

}

displ_A_bin <- mean(displacements(pcoa_result$vectors.cor[bins.trim[[1]], 1:17],

reference = centroid[[1]]))

qA_displ_bin <- c(displ_A_bin,displ_A_bin)

displ_bin <- list()

boot_displ_bin <- list()

disp_ds_bin <- list()

q_displ_bin <- list()

for (i in 2:length(bins.trim)) {

displ_bin[[i]] <- mean(displacements(pcoa_result$vectors.cor[bins.trim[[i]], 1:17],

reference = centroid[[i-1]]))

boot_displ_bin[[i]] <- boot.matrix(data = pcoa_result$vectors.cor[bins.trim[[i]], 1:17],

bootstraps = 9999)

disp_ds_bin[[i]] <- apply(X = boot_displ_bin[[i]]$subsets[[1]][[2]],

MARGIN = 2,

FUN = function(x) mean(displacements(pcoa_result$vectors.cor[bins.trim[[i]], ][x, 1:17],

reference = centroid[[i-1]])))

q_displ_bin[[i]] <- sort(quantile(disp_ds_bin[[i]], c(0.025, 0.975), names = FALSE))

}

Displacements_previous_bin_result <- data.frame(Bin = c("Carnian", "Norian-Rhaetian", "pre-Toarcian", "Toarcian-MJ"),

Disp = c(displ_A_bin, displ_bin[[2]], displ_bin[[3]], displ_bin[[4]]),

LowerLimit = c(qA_displ_bin[1], q_displ_bin[[2]][1], q_displ_bin[[3]][1], q_displ_bin[[4]][1]),

UpperLimit = c(qA_displ_bin[2], q_displ_bin[[2]][2], q_displ_bin[[3]][2], q_displ_bin[[4]][2]))

#pdf(file = "graphics/Displacements_from_previous_time_bin.pdf", width = 6, height = 6)

Displacements_previous_bin_result$Bin <- factor(Displacements_previous_bin_result$Bin, levels = c("Carnian","Norian-Rhaetian","pre-Toarcian","Toarcian-MJ"))

ggplot(Displacements_previous_bin_result) +

geom_errorbar(aes(x = Bin, ymin = LowerLimit, ymax = UpperLimit, col = Bin),

width = 0.25, lwd = 1) +

geom_point(aes(x = Bin, y = Disp, col = Bin)) +

theme_bw() +

theme(legend.position = "none")

#dev.off()

#Displacements from the centroid of previous time bin with rarefied matrices

centroid_rarefied <- list()

for (k in 1:length(bins.trim)) {

centroid_rarefied[[k]] <- apply(pcoa_result$vectors.cor[bins.trim[[k]], 1:17], 2, mean)

}

displ_rarefied_A_bin <- mean(displacements(pcoa_result$vectors.cor[bins.trim[[1]], 1:17],

reference = centroid_rarefied[[1]]))

qA_displ_rarefied_bin <- c(displ_rarefied_A_bin,displ_rarefied_A_bin)

displ_rarefied_bin <- list()

boot_displ_rarefied_bin <- list()

disp_rarefied_ds_bin <- list()

q_displ_rarefied_bin <- list()

for (i in 2:length(bins.trim)) {

displ_rarefied_bin[[i]] <- mean(displacements(pcoa_result$vectors.cor[bins.trim[[i]], 1:17],

reference = centroid_rarefied[[i-1]]))

boot_displ_rarefied_bin[[i]] <- boot.matrix(data = pcoa_result$vectors.cor[bins.trim[[i]], 1:17],

bootstraps = 9999,

rarefaction = 7)

disp_rarefied_ds_bin[[i]] <- apply(X = boot_displ_rarefied_bin[[i]]$subsets[[1]][[2]],

MARGIN = 2,

FUN = function(x) mean(displacements(pcoa_result$vectors.cor[bins.trim[[i]], ][x, 1:17],

reference = centroid_rarefied[[i-1]])))

q_displ_rarefied_bin[[i]] <- sort(quantile(disp_rarefied_ds_bin[[i]], c(0.025, 0.975), names = FALSE))

}

Displacements_previous_bin_rarefied_result <- data.frame(Bin = c("Carnian", "Norian-Rhaetian", "pre-Toarcian", "Toarcian-MJ"),

Disp = c(displ_rarefied_A_bin, displ_rarefied_bin[[2]], displ_rarefied_bin[[3]], displ_rarefied_bin[[4]]),

LowerLimit = c(qA_displ_rarefied_bin[1], q_displ_rarefied_bin[[2]][1], q_displ_rarefied_bin[[3]][1], q_displ_rarefied_bin[[4]][1]),

UpperLimit = c(qA_displ_rarefied_bin[2], q_displ_rarefied_bin[[2]][2], q_displ_rarefied_bin[[3]][2], q_displ_rarefied_bin[[4]][2]))

#pdf(file = "graphics/Displacements_from_previous_time_rarefied_bin.pdf", width = 6, height = 6)

Displacements_previous_bin_rarefied_result$Bin <- factor(Displacements_previous_bin_rarefied_result$Bin, levels = c("Carnian","Norian-Rhaetian","pre-Toarcian","Toarcian-MJ"))

ggplot(Displacements_previous_bin_rarefied_result) +

geom_errorbar(aes(x = Bin, ymin = LowerLimit, ymax = UpperLimit, col = Bin),

width = 0.25, lwd = 1) +

geom_point(aes(x = Bin, y = Disp, col = Bin)) +

theme_bw() +

theme(legend.position = "none")

#dev.off()

#Displacements from the centroid of previous time bin without Barapasaurus and Isanosaurus

centroid_Barapa_Isano <- list()

for (k in 1:length(bins.trim_Barapa_Isano)) {

centroid_Barapa_Isano[[k]] <- apply(pcoa_result_Barapa_Isano$vectors.cor[bins.trim_Barapa_Isano[[k]], 1:17], 2, mean)

}

displ_A_bin_Barapa_Isano <- mean(displacements(pcoa_result_Barapa_Isano$vectors.cor[bins.trim_Barapa_Isano[[1]], 1:17],

reference = centroid_Barapa_Isano[[1]]))

qA_displ_bin_Barapa_Isano <- c(displ_A_bin_Barapa_Isano,displ_A_bin_Barapa_Isano)

displ_bin_Barapa_Isano <- list()

boot_displ_bin_Barapa_Isano <- list()

disp_ds_bin_Barapa_Isano <- list()

q_displ_bin_Barapa_Isano <- list()

for (i in 2:length(bins.trim_Barapa_Isano)) {

displ_bin_Barapa_Isano[[i]] <- mean(displacements(pcoa_result_Barapa_Isano$vectors.cor[bins.trim_Barapa_Isano[[i]], 1:17],

reference = centroid_Barapa_Isano[[i-1]]))

boot_displ_bin_Barapa_Isano[[i]] <- boot.matrix(data = pcoa_result_Barapa_Isano$vectors.cor[bins.trim_Barapa_Isano[[i]], 1:17],

bootstraps = 9999)

disp_ds_bin_Barapa_Isano[[i]] <- apply(X = boot_displ_bin_Barapa_Isano[[i]]$subsets[[1]][[2]],

MARGIN = 2,

FUN = function(x) mean(displacements(pcoa_result_Barapa_Isano$vectors.cor[bins.trim_Barapa_Isano[[i]], ][x, 1:17],

reference = centroid_Barapa_Isano[[i-1]])))

q_displ_bin_Barapa_Isano[[i]] <- sort(quantile(disp_ds_bin_Barapa_Isano[[i]], c(0.025, 0.975), names = FALSE))

}

Displacements_previous_bin_result_Barapa_Isano <- data.frame(Bin = c("Carnian", "Norian-Rhaetian", "pre-Toarcian", "Toarcian-MJ"),

Disp = c(displ_A_bin_Barapa_Isano, displ_bin_Barapa_Isano[[2]], displ_bin_Barapa_Isano[[3]], displ_bin_Barapa_Isano[[4]]),

LowerLimit = c(qA_displ_bin_Barapa_Isano[1], q_displ_bin_Barapa_Isano[[2]][1], q_displ_bin_Barapa_Isano[[3]][1], q_displ_bin_Barapa_Isano[[4]][1]),

UpperLimit = c(qA_displ_bin_Barapa_Isano[2], q_displ_bin_Barapa_Isano[[2]][2], q_displ_bin_Barapa_Isano[[3]][2], q_displ_bin_Barapa_Isano[[4]][2]))

#pdf(file = "graphics/Displacements_from_previous_time_bin_without_Barapasaurus_and_Isanosaurus.pdf", width = 6, height = 6)

Displacements_previous_bin_result_Barapa_Isano$Bin <- factor(Displacements_previous_bin_result_Barapa_Isano$Bin, levels = c("Carnian","Norian-Rhaetian","pre-Toarcian","Toarcian-MJ"))

ggplot(Displacements_previous_bin_result_Barapa_Isano) +

geom_errorbar(aes(x = Bin, ymin = LowerLimit, ymax = UpperLimit, col = Bin),

width = 0.25, lwd = 1) +

geom_point(aes(x = Bin, y = Disp, col = Bin)) +

theme_bw() +

theme(legend.position = "none")

#dev.off()

#Calculate displacement locomotion styles from centroid

displ_locomotion <- list()

for (i in 1:length(locomotions)) {

displ_locomotion[[i]] <- mean(displacements(pcoa_result$vectors.cor[locomotions[[i]], 1:17]))

}

boot_displ_locomotion <- list()

disp_ds_locomotion <- list()

q_displ_locomotion <- list()

for (j in 1:length(locomotions)) {

boot_displ_locomotion[[j]] <- boot.matrix(data = pcoa_result$vectors.cor[locomotions[[j]], 1:17],

bootstraps = 9999)

disp_ds_locomotion[[j]] <- apply(X = boot_displ_locomotion[[j]]$subsets[[1]][[2]],

MARGIN = 2,

FUN = function(x) mean(displacements(pcoa_result$vectors.cor[locomotions[[j]], ][x, 1:17])))

q_displ_locomotion[[j]] <- sort(quantile(disp_ds_locomotion[[j]], c(0.025, 0.975), names = FALSE))

}

Displacements_result_locomotion <- data.frame(Bin = c("Bipeds","Quadrupeds"),

Disp = c(displ_locomotion[[1]], displ_locomotion[[2]]),

LowerLimit = c(q_displ_locomotion[[1]][1], q_displ_locomotion[[2]][1]),

UpperLimit = c(q_displ_locomotion[[1]][2], q_displ_locomotion[[2]][2]))

#pdf(file = "graphics/Displacements_from_centroid_locomotion.pdf", width = 6, height = 6)

Displacements_result_locomotion$Bin <- factor(Displacements_result_locomotion$Bin, levels = c("Bipeds","Quadrupeds"))

ggplot(Displacements_result_locomotion) +

geom_errorbar(aes(x = Bin, ymin = LowerLimit, ymax = UpperLimit, col = Bin),

width = 0.25, lwd = 1) +

geom_point(aes(x = Bin, y = Disp, col = Bin)) +

theme_bw() +

theme(legend.position = "none")

#dev.off()

#Calculate displacement locomotion styles from centroid with reduced sample

displ_locomotion_red <- list()

for (i in 1:length(locomotions_red)) {

displ_locomotion_red[[i]] <- mean(displacements(pcoa_result$vectors.cor[locomotions_red[[i]], 1:17]))

}

boot_displ_locomotion_red <- list()

disp_ds_locomotion_red <- list()

q_displ_locomotion_red <- list()

for (j in 1:length(locomotions_red)) {

boot_displ_locomotion_red[[j]] <- boot.matrix(data = pcoa_result$vectors.cor[locomotions_red[[j]], 1:17],

bootstraps = 9999)

disp_ds_locomotion_red[[j]] <- apply(X = boot_displ_locomotion_red[[j]]$subsets[[1]][[2]],

MARGIN = 2,

FUN = function(x) mean(displacements(pcoa_result$vectors.cor[locomotions_red[[j]], ][x, 1:17])))

q_displ_locomotion_red[[j]] <- sort(quantile(disp_ds_locomotion_red[[j]], c(0.025, 0.975), names = FALSE))

}

Displacements_result_locomotion_red <- data.frame(Bin = c("Bipeds","Quadrupeds"),

Disp = c(displ_locomotion_red[[1]], displ_locomotion_red[[2]]),

LowerLimit = c(q_displ_locomotion_red[[1]][1], q_displ_locomotion_red[[2]][1]),

UpperLimit = c(q_displ_locomotion_red[[1]][2], q_displ_locomotion_red[[2]][2]))

#pdf(file = "graphics/Displacements_from_centroid_locomotion_reduced_sample.pdf", width = 6, height = 6)

Displacements_result_locomotion_red$Bin <- factor(Displacements_result_locomotion_red$Bin, levels = c("Bipeds","Quadrupeds"))

ggplot(Displacements_result_locomotion_red) +

geom_errorbar(aes(x = Bin, ymin = LowerLimit, ymax = UpperLimit, col = Bin),

width = 0.25, lwd = 1) +

geom_point(aes(x = Bin, y = Disp, col = Bin)) +

theme_bw() +

theme(legend.position = "none")

#dev.off()

#Delete outliers from the ordered dataset, time bins, and locomotion styles

A_prun <- setdiff(A,outliers)

B_prun <- setdiff(B,outliers)

C_prun <- setdiff(C,outliers)

D_prun <- setdiff(D,outliers)

bins_prun <- list(A_prun,B_prun,C_prun,D_prun)

bipeds.PCoA_prun <- setdiff(bipeds.PCoA,outliers)

quadrupeds.PCoA_prun <- setdiff(quadrupeds.PCoA,outliers)

locomotions.PCoA_prun <- list(bipeds.PCoA_prun, quadrupeds.PCoA_prun)

bipeds.PCoA_prun_red <- setdiff(bipeds.PCoA_red,outliers)

quadrupeds.PCoA_prun_red <- setdiff(quadrupeds.PCoA_red,outliers)

locomotions.PCoA_prun_red <- list(bipeds.PCoA_prun_red, quadrupeds.PCoA_prun_red)

pcoa_result_prun <- pcoa_result

pcoa_result_prun$vectors.cor <- pcoa_result$vectors.cor[setdiff(names(pcoa_result$vectors.cor[,1]),outliers),]

#Delete Barapsaurus and Isanosaurus from the dataset without outliers

A_prun_Barapa_Isano <- setdiff(A_prun, Barapa_Isano)

B_prun_Barapa_Isano <- setdiff(B_prun, Barapa_Isano)

C_prun_Barapa_Isano <- setdiff(C_prun, Barapa_Isano)

D_prun_Barapa_Isano <- setdiff(D_prun, Barapa_Isano)

bins_prun_Barapa_Isano <- list(A_prun_Barapa_Isano,B_prun_Barapa_Isano,C_prun_Barapa_Isano,D_prun_Barapa_Isano)

bipeds.PCoA_prun_Barapa_Isano <- setdiff(bipeds.PCoA_prun,Barapa_Isano)

quadrupeds.PCoA_prun_Barapa_Isano <- setdiff(quadrupeds.PCoA_prun,Barapa_Isano)

locomotions.PCoA_prun_Barapa_Isano <- list(bipeds.PCoA_prun_Barapa_Isano, quadrupeds.PCoA_prun_Barapa_Isano)

bipeds.PCoA_prun_Barapa_Isano_red <- setdiff(bipeds.PCoA_prun_red,Barapa_Isano)

quadrupeds.PCoA_prun_Barapa_Isano_red <- setdiff(quadrupeds.PCoA_prun_red,Barapa_Isano)

locomotions.PCoA_prun_Barapa_Isano_red <- list(bipeds.PCoA_prun_Barapa_Isano_red, quadrupeds.PCoA_prun_Barapa_Isano_red)

pcoa_result_prun_Barapa_Isano <- pcoa_result_prun

pcoa_result_prun_Barapa_Isano$vectors.cor <- pcoa_result_prun$vectors.cor[setdiff(names(pcoa_result_prun$vectors.cor[,1]),Barapa_Isano),]

#Calculate Sum of Variance without outliers

sov_result_prun <- sov_bootstrap(Vectors = pcoa_result_prun$vectors.cor[,1:17],

Replicates = 9999,

Bins = list("Carnian" = A_prun,

"Norian-Rhaetian" = B_prun,

"pre-Toarcian" = C_prun,

"Toarcian-MJ" = D_prun))

#pdf(file = "graphics/SoV_without_outliers.pdf", width = 6, height = 6)

sov_result_prun$Bin <- factor(sov_result_prun$Bin, levels = c("Carnian","Norian-Rhaetian","pre-Toarcian","Toarcian-MJ"))

ggplot(sov_result_prun) +

geom_errorbar(aes(x = Bin, ymin = LowerLimit, ymax = UpperLimit, col = Bin),

width = 0.25, lwd = 1) +

geom_point(aes(x = Bin, y = SoV, col = Bin)) +

theme_bw() +

theme(legend.position = "none")

#dev.off()

#Calculate Sum of Ranges without outliers

SoR_prun <- list()

for (i in 1:length(bins_prun)) {

SoR_prun[[i]] <- sum(ranges(pcoa_result_prun$vectors.cor[bins_prun[[i]], 1:17]))

}

boot_SoR_prun <- list()

SoR_ds_prun <- list()

q_SoR_prun <- list()

for (j in 1:length(bins_prun)) {

boot_SoR_prun[[j]] <- boot.matrix(data = pcoa_result_prun$vectors.cor[bins_prun[[j]], 1:17],

bootstraps = 9999)

SoR_ds_prun[[j]] <- apply(X = boot_SoR_prun[[j]]$subsets[[1]][[2]],

MARGIN = 2,

FUN = function(x) sum(ranges(pcoa_result_prun$vectors.cor[bins_prun[[j]], ][x, 1:17])))

q_SoR_prun[[j]] <- sort(quantile(SoR_ds_prun[[j]], c(0.05, 1), names = FALSE))

}

SoR_result_prun <- data.frame(Bin = c("Carnian","Norian-Rhaetian","pre-Toarcian","Toarcian-MJ"),

SoR = c(SoR_prun[[1]], SoR_prun[[2]], SoR_prun[[3]], SoR_prun[[4]]),

LowerLimit = c(q_SoR_prun[[1]][1], q_SoR_prun[[2]][1], q_SoR_prun[[3]][1], q_SoR_prun[[4]][1]),

UpperLimit = c(q_SoR_prun[[1]][2], q_SoR_prun[[2]][2], q_SoR_prun[[3]][2], q_SoR_prun[[4]][2]))

#pdf(file = "graphics/SoR_without_outliers.pdf", width = 6, height = 6)

SoR_result_prun$Bin <- factor(SoR_result_prun$Bin, levels = c("Carnian","Norian-Rhaetian","pre-Toarcian","Toarcian-MJ"))

ggplot(SoR_result_prun) +

geom_errorbar(aes(x = Bin, ymin = LowerLimit, ymax = UpperLimit, col = Bin),

width = 0.25, lwd = 1) +

geom_point(aes(x = Bin, y = SoR, col = Bin)) +

theme_bw() +

theme(legend.position = "none")

#dev.off()

#Calculate Sum of Variance for locomotion styles without outliers

sov_result_prun_locomotion <- sov_bootstrap(Vectors = pcoa_result_prun$vectors.cor[,1:17],

Replicates = 9999,

Bins = list("Bipeds" = bipeds.PCoA_prun,

"Quadrupeds" = quadrupeds.PCoA_prun))

#pdf(file = "graphics/SoV_locomotion_without_outliers.pdf", width = 6, height = 6)

sov_result_prun_locomotion$Bin <- factor(sov_result_prun_locomotion$Bin, levels = c("Bipeds","Quadrupeds"))

ggplot(sov_result_prun_locomotion) +

geom_errorbar(aes(x = Bin, ymin = LowerLimit, ymax = UpperLimit, col = Bin),

width = 0.25, lwd = 1) +

geom_point(aes(x = Bin, y = SoV, col = Bin)) +

theme_bw() +

theme(legend.position = "none")

#dev.off()

#Calculate Sum of Ranges for locomotion styles without outliers

SoR_prun_locomotion <- list()

for (i in 1:length(locomotions.PCoA_prun)) {

SoR_prun_locomotion[[i]] <- sum(ranges(pcoa_result_prun$vectors.cor[locomotions.PCoA_prun[[i]], 1:17]))

}

boot_SoR_prun_locomotion <- list()

SoR_ds_prun_locomotion <- list()

q_SoR_prun_locomotion <- list()

for (j in 1:length(locomotions.PCoA_prun)) {

boot_SoR_prun_locomotion[[j]] <- boot.matrix(data = pcoa_result_prun$vectors.cor[locomotions.PCoA_prun[[j]], 1:17],

bootstraps = 9999)

SoR_ds_prun_locomotion[[j]] <- apply(X = boot_SoR_prun_locomotion[[j]]$subsets[[1]][[2]],

MARGIN = 2,

FUN = function(x) sum(ranges(pcoa_result_prun$vectors.cor[locomotions.PCoA_prun[[j]], ][x, 1:17])))

q_SoR_prun_locomotion[[j]] <- sort(quantile(SoR_ds_prun_locomotion[[j]], c(0.05, 1), names = FALSE))

}

SoR_result_prun_locomotion <- data.frame(Bin = c("Bipeds","Quadrupeds"),

SoR = c(SoR_prun_locomotion[[1]], SoR_prun_locomotion[[2]]),

LowerLimit = c(q_SoR_prun_locomotion[[1]][1], q_SoR_prun_locomotion[[2]][1]),

UpperLimit = c(q_SoR_prun_locomotion[[1]][2], q_SoR_prun_locomotion[[2]][2]))

#pdf(file = "graphics/SoR_locomotion_without_outliers.pdf", width = 6, height = 6)

SoR_result_prun_locomotion$Bin <- factor(SoR_result_prun_locomotion$Bin, levels = c("Bipeds","Quadrupeds"))

ggplot(SoR_result_prun_locomotion) +

geom_errorbar(aes(x = Bin, ymin = LowerLimit, ymax = UpperLimit, col = Bin),

width = 0.25, lwd = 1) +

geom_point(aes(x = Bin, y = SoR, col = Bin)) +

theme_bw() +

theme(legend.position = "none")

#dev.off()

#Calculate Sum of Variance for locomotion styles without outliers and reduced sample

sov_result_prun_locomotion_red <- sov_bootstrap(Vectors = pcoa_result_prun$vectors.cor[,1:17],

Replicates = 9999,

Bins = list("Bipeds" = bipeds.PCoA_prun_red,

"Quadrupeds" = quadrupeds.PCoA_prun_red))

#pdf(file = "graphics/SoV_locomotion_without_outliers_reduced_sample.pdf", width = 6, height = 6)

sov_result_prun_locomotion_red$Bin <- factor(sov_result_prun_locomotion_red$Bin, levels = c("Bipeds","Quadrupeds"))

ggplot(sov_result_prun_locomotion_red) +

geom_errorbar(aes(x = Bin, ymin = LowerLimit, ymax = UpperLimit, col = Bin),

width = 0.25, lwd = 1) +

geom_point(aes(x = Bin, y = SoV, col = Bin)) +

theme_bw() +

theme(legend.position = "none")

#dev.off()

#Calculate Sum of Ranges for locomotion styles without outliers and reduced sample

SoR_prun_locomotion_red <- list()

for (i in 1:length(locomotions.PCoA_prun_red)) {

SoR_prun_locomotion_red[[i]] <- sum(ranges(pcoa_result_prun$vectors.cor[locomotions.PCoA_prun_red[[i]], 1:17]))

}

boot_SoR_prun_locomotion_red <- list()

SoR_ds_prun_locomotion_red <- list()

q_SoR_prun_locomotion_red <- list()

for (j in 1:length(locomotions.PCoA_prun_red)) {

boot_SoR_prun_locomotion_red[[j]] <- boot.matrix(data = pcoa_result_prun$vectors.cor[locomotions.PCoA_prun_red[[j]], 1:17],

bootstraps = 9999)

SoR_ds_prun_locomotion_red[[j]] <- apply(X = boot_SoR_prun_locomotion_red[[j]]$subsets[[1]][[2]],

MARGIN = 2,

FUN = function(x) sum(ranges(pcoa_result_prun$vectors.cor[locomotions.PCoA_prun_red[[j]], ][x, 1:17])))

q_SoR_prun_locomotion_red[[j]] <- sort(quantile(SoR_ds_prun_locomotion_red[[j]], c(0.05, 1), names = FALSE))

}

SoR_result_prun_locomotion_red <- data.frame(Bin = c("Bipeds","Quadrupeds"),

SoR = c(SoR_prun_locomotion_red[[1]], SoR_prun_locomotion_red[[2]]),

LowerLimit = c(q_SoR_prun_locomotion_red[[1]][1], q_SoR_prun_locomotion_red[[2]][1]),

UpperLimit = c(q_SoR_prun_locomotion_red[[1]][2], q_SoR_prun_locomotion_red[[2]][2]))

#pdf(file = "graphics/SoR_locomotion_without_outliers_reduced_sample.pdf", width = 6, height = 6)

SoR_result_prun_locomotion_red$Bin <- factor(SoR_result_prun_locomotion_red$Bin, levels = c("Bipeds","Quadrupeds"))

ggplot(SoR_result_prun_locomotion_red) +

geom_errorbar(aes(x = Bin, ymin = LowerLimit, ymax = UpperLimit, col = Bin),

width = 0.25, lwd = 1) +

geom_point(aes(x = Bin, y = SoR, col = Bin)) +

theme_bw() +

theme(legend.position = "none")

#dev.off()

#Calculate Sum of Variance without outliers and Barapasaurus and Isanosaurus

sov_result_prun_Barapa_Isano <- sov_bootstrap(Vectors = pcoa_result_prun_Barapa_Isano$vectors.cor[,1:17],

Replicates = 9999,

Bins = list("Carnian" = A_prun_Barapa_Isano,

"Norian-Rhaetian" = B_prun_Barapa_Isano,

"pre-Toarcian" = C_prun_Barapa_Isano,

"Toarcian-MJ" = D_prun_Barapa_Isano))

#pdf(file = "graphics/SoV_without_outliers_and_Barapasaurus_Isanosaurus.pdf", width = 6, height = 6)

sov_result_prun_Barapa_Isano$Bin <- factor(sov_result_prun_Barapa_Isano$Bin, levels = c("Carnian","Norian-Rhaetian","pre-Toarcian","Toarcian-MJ"))

ggplot(sov_result_prun_Barapa_Isano) +

geom_errorbar(aes(x = Bin, ymin = LowerLimit, ymax = UpperLimit, col = Bin),

width = 0.25, lwd = 1) +

geom_point(aes(x = Bin, y = SoV, col = Bin)) +

theme_bw() +

theme(legend.position = "none")

#dev.off()

#Calculate Sum of Ranges without outliers and Barapasaurus and Isanosaurus

SoR_prun_Barapa_Isano <- list()

for (i in 1:length(bins_prun_Barapa_Isano)) {

SoR_prun_Barapa_Isano[[i]] <- sum(ranges(pcoa_result_prun_Barapa_Isano$vectors.cor[bins_prun_Barapa_Isano[[i]], 1:17]))

}

boot_SoR_prun_Barapa_Isano <- list()

SoR_ds_prun_Barapa_Isano <- list()

q_SoR_prun_Barapa_Isano <- list()

for (j in 1:length(bins_prun_Barapa_Isano)) {

boot_SoR_prun_Barapa_Isano[[j]] <- boot.matrix(data = pcoa_result_prun_Barapa_Isano$vectors.cor[bins_prun_Barapa_Isano[[j]], 1:17],

bootstraps = 9999)

SoR_ds_prun_Barapa_Isano[[j]] <- apply(X = boot_SoR_prun_Barapa_Isano[[j]]$subsets[[1]][[2]],

MARGIN = 2,

FUN = function(x) sum(ranges(pcoa_result_prun_Barapa_Isano$vectors.cor[bins_prun_Barapa_Isano[[j]], ][x, 1:17])))

q_SoR_prun_Barapa_Isano[[j]] <- sort(quantile(SoR_ds_prun_Barapa_Isano[[j]], c(0.05, 1), names = FALSE))

}

SoR_result_prun_Barapa_Isano <- data.frame(Bin = c("Carnian","Norian-Rhaetian","pre-Toarcian","Toarcian-MJ"),

SoR = c(SoR_prun_Barapa_Isano[[1]], SoR_prun_Barapa_Isano[[2]], SoR_prun_Barapa_Isano[[3]], SoR_prun_Barapa_Isano[[4]]),

LowerLimit = c(q_SoR_prun_Barapa_Isano[[1]][1], q_SoR_prun_Barapa_Isano[[2]][1], q_SoR_prun_Barapa_Isano[[3]][1], q_SoR_prun_Barapa_Isano[[4]][1]),

UpperLimit = c(q_SoR_prun_Barapa_Isano[[1]][2], q_SoR_prun_Barapa_Isano[[2]][2], q_SoR_prun_Barapa_Isano[[3]][2], q_SoR_prun_Barapa_Isano[[4]][2]))

#pdf(file = "graphics/SoR_without_outliers_Barapasaurs_Isanosaurus.pdf", width = 6, height = 6)

SoR_result_prun_Barapa_Isano$Bin <- factor(SoR_result_prun_Barapa_Isano$Bin, levels = c("Carnian","Norian-Rhaetian","pre-Toarcian","Toarcian-MJ"))

ggplot(SoR_result_prun_Barapa_Isano) +

geom_errorbar(aes(x = Bin, ymin = LowerLimit, ymax = UpperLimit, col = Bin),

width = 0.25, lwd = 1) +

geom_point(aes(x = Bin, y = SoR, col = Bin)) +

theme_bw() +

theme(legend.position = "none")

#dev.off()

#pdf(file = "graphics/SoR_without_outliers_Barapasaurs_Isanosaurus_geoscale.pdf", width = 6, height = 6)

geoscalePlot(data=SoR_result_prun_Barapa_Isano[,2], ages=meanagesWMPD, units=c("Age" ,"Epoch", "Period"),

boxes="Age", age.lim=c(233.5,150),pch = 21, cex.age = 0.8,cex.ts = 0.8, data.lim=c(min(SoR_result_prun_Barapa_Isano$LowerLimit),max(SoR_result_prun_Barapa_Isano$UpperLimit)),

label="SoR with 95% confidence interval")

lines(rbind(meanagesWMPD, meanagesWMPD, NA),rbind(SoR_result_prun_Barapa_Isano[,3],SoR_result_prun_Barapa_Isano[,4],NA))

#dev.off()

#Calculate Sum of Variance for locomotion styles without outliers and Barapasaurus and Isanosaurus

sov_result_locomotion_prun_Barapa_Isano <- sov_bootstrap(Vectors = pcoa_result_prun_Barapa_Isano$vectors.cor[,1:17],

Replicates = 9999,

Bins = list("Bipeds" = bipeds.PCoA_prun_Barapa_Isano,

"Quadrupeds" = quadrupeds.PCoA_prun_Barapa_Isano))

#pdf(file = "graphics/SoV_locomotion_without_outliers_and_Barapasaurus_Isanosaurus.pdf", width = 6, height = 6)

sov_result_locomotion_prun_Barapa_Isano$Bin <- factor(sov_result_locomotion_prun_Barapa_Isano$Bin, levels = c("Bipeds","Quadrupeds"))

ggplot(sov_result_locomotion_prun_Barapa_Isano) +

geom_errorbar(aes(x = Bin, ymin = LowerLimit, ymax = UpperLimit, col = Bin),

width = 0.25, lwd = 1) +

geom_point(aes(x = Bin, y = SoV, col = Bin)) +

theme_bw() +

theme(legend.position = "none")

#dev.off()

#Calculate Sum of Ranges for locomotion styles without outliers and Barapasaurus and Isanosaurus

SoR_prun_locomotion_Barapa_Isano <- list()

for (i in 1:length(locomotions.PCoA_prun_Barapa_Isano)) {

SoR_prun_locomotion_Barapa_Isano[[i]] <- sum(ranges(pcoa_result_prun_Barapa_Isano$vectors.cor[locomotions.PCoA_prun_Barapa_Isano[[i]], 1:17]))

}

boot_SoR_prun_locomotion_Barapa_Isano <- list()

SoR_ds_prun_locomotion_Barapa_Isano <- list()

q_SoR_prun_locomotion_Barapa_Isano <- list()

for (j in 1:length(locomotions.PCoA_prun_Barapa_Isano)) {

boot_SoR_prun_locomotion_Barapa_Isano[[j]] <- boot.matrix(data = pcoa_result_prun_Barapa_Isano$vectors.cor[locomotions.PCoA_prun_Barapa_Isano[[j]], 1:17],

bootstraps = 9999)

SoR_ds_prun_locomotion_Barapa_Isano[[j]] <- apply(X = boot_SoR_prun_locomotion_Barapa_Isano[[j]]$subsets[[1]][[2]],

MARGIN = 2,

FUN = function(x) sum(ranges(pcoa_result_prun_Barapa_Isano$vectors.cor[locomotions.PCoA_prun_Barapa_Isano[[j]], ][x, 1:17])))

q_SoR_prun_locomotion_Barapa_Isano[[j]] <- sort(quantile(SoR_ds_prun_locomotion_Barapa_Isano[[j]], c(0.05, 1), names = FALSE))

}

SoR_result_prun_locomotion_Barapa_Isano <- data.frame(Bin = c("Bipeds","Quadrupeds"),

SoR = c(SoR_prun_locomotion_Barapa_Isano[[1]], SoR_prun_locomotion_Barapa_Isano[[2]]),

LowerLimit = c(q_SoR_prun_locomotion_Barapa_Isano[[1]][1], q_SoR_prun_locomotion_Barapa_Isano[[2]][1]),

UpperLimit = c(q_SoR_prun_locomotion_Barapa_Isano[[1]][2], q_SoR_prun_locomotion_Barapa_Isano[[2]][2]))

#pdf(file = "graphics/SoR_locomotion_without_outliers_and_Barapasaurus_Isanosaurus.pdf", width = 6, height = 6)

SoR_result_prun_locomotion_Barapa_Isano$Bin <- factor(SoR_result_prun_locomotion_Barapa_Isano$Bin, levels = c("Bipeds","Quadrupeds"))

ggplot(SoR_result_prun_locomotion_Barapa_Isano) +

geom_errorbar(aes(x = Bin, ymin = LowerLimit, ymax = UpperLimit, col = Bin),

width = 0.25, lwd = 1) +

geom_point(aes(x = Bin, y = SoR, col = Bin)) +

theme_bw() +

theme(legend.position = "none")

#dev.off()

#Calculate Sum of Variance for locomotion styles without outliers and Barapasaurus and Isanosaurus and reduced sample

sov_result_locomotion_prun_Barapa_Isano_red <- sov_bootstrap(Vectors = pcoa_result_prun_Barapa_Isano$vectors.cor[,1:17],

Replicates = 9999,

Bins = list("Bipeds" = bipeds.PCoA_prun_Barapa_Isano_red,

"Quadrupeds" = quadrupeds.PCoA_prun_Barapa_Isano_red))

#pdf(file = "graphics/SoV_locomotion_without_outliers_and_Barapasaurus_Isanosaurus_reduced_sample.pdf", width = 6, height = 6)

sov_result_locomotion_prun_Barapa_Isano_red$Bin <- factor(sov_result_locomotion_prun_Barapa_Isano_red$Bin, levels = c("Bipeds","Quadrupeds"))

ggplot(sov_result_locomotion_prun_Barapa_Isano_red) +

geom_errorbar(aes(x = Bin, ymin = LowerLimit, ymax = UpperLimit, col = Bin),

width = 0.25, lwd = 1) +

geom_point(aes(x = Bin, y = SoV, col = Bin)) +

theme_bw() +

theme(legend.position = "none")

#dev.off()

#Calculate Sum of Variance for locomotion styles without outliers and Barapasaurus and Isanosaurus and reduced sample

SoR_prun_locomotion_Barapa_Isano_red <- list()

for (i in 1:length(locomotions.PCoA_prun_Barapa_Isano_red)) {

SoR_prun_locomotion_Barapa_Isano_red[[i]] <- sum(ranges(pcoa_result_prun_Barapa_Isano$vectors.cor[locomotions.PCoA_prun_Barapa_Isano_red[[i]], 1:17]))

}

boot_SoR_prun_locomotion_Barapa_Isano_red <- list()

SoR_ds_prun_locomotion_Barapa_Isano_red <- list()

q_SoR_prun_locomotion_Barapa_Isano_red <- list()

for (j in 1:length(locomotions.PCoA_prun_Barapa_Isano_red)) {

boot_SoR_prun_locomotion_Barapa_Isano_red[[j]] <- boot.matrix(data = pcoa_result_prun_Barapa_Isano$vectors.cor[locomotions.PCoA_prun_Barapa_Isano_red[[j]], 1:17],

bootstraps = 9999)

SoR_ds_prun_locomotion_Barapa_Isano_red[[j]] <- apply(X = boot_SoR_prun_locomotion_Barapa_Isano_red[[j]]$subsets[[1]][[2]],

MARGIN = 2,

FUN = function(x) sum(ranges(pcoa_result_prun_Barapa_Isano$vectors.cor[locomotions.PCoA_prun_Barapa_Isano_red[[j]], ][x, 1:17])))

q_SoR_prun_locomotion_Barapa_Isano_red[[j]] <- sort(quantile(SoR_ds_prun_locomotion_Barapa_Isano_red[[j]], c(0.05, 1), names = FALSE))

}

SoR_result_prun_locomotion_Barapa_Isano_red <- data.frame(Bin = c("Bipeds","Quadrupeds"),

SoR = c(SoR_prun_locomotion_Barapa_Isano_red[[1]], SoR_prun_locomotion_Barapa_Isano_red[[2]]),

LowerLimit = c(q_SoR_prun_locomotion_Barapa_Isano_red[[1]][1], q_SoR_prun_locomotion_Barapa_Isano_red[[2]][1]),

UpperLimit = c(q_SoR_prun_locomotion_Barapa_Isano_red[[1]][2], q_SoR_prun_locomotion_Barapa_Isano_red[[2]][2]))

#pdf(file = "graphics/SoR_locomotion_without_outliers_and_Barapasaurus_Isanosaurus_reduced_sample.pdf", width = 6, height = 6)

SoR_result_prun_locomotion_Barapa_Isano_red$Bin <- factor(SoR_result_prun_locomotion_Barapa_Isano_red$Bin, levels = c("Bipeds","Quadrupeds"))

ggplot(SoR_result_prun_locomotion_Barapa_Isano_red) +

geom_errorbar(aes(x = Bin, ymin = LowerLimit, ymax = UpperLimit, col = Bin),

width = 0.25, lwd = 1) +

geom_point(aes(x = Bin, y = SoR, col = Bin)) +

theme_bw() +

theme(legend.position = "none")

#dev.off()

#Calculate displacements from centroid without outliers

displ_prun <- list()

for (i in 1:length(bins_prun)) {

displ_prun[[i]] <- mean(displacements(pcoa_result_prun$vectors.cor[bins_prun[[i]], 1:17]))

}

boot_displ_prun <- list()

disp_ds_prun <- list()

q_displ_prun <- list()

for (j in 1:length(bins_prun)) {

boot_displ_prun[[j]] <- boot.matrix(data = pcoa_result_prun$vectors.cor[bins_prun[[j]], 1:17],

bootstraps = 9999)

disp_ds_prun[[j]] <- apply(X = boot_displ_prun[[j]]$subsets[[1]][[2]],

MARGIN = 2,

FUN = function(x) mean(displacements(pcoa_result_prun$vectors.cor[bins_prun[[j]], ][x, 1:17])))

q_displ_prun[[j]] <- sort(quantile(disp_ds_prun[[j]], c(0.025, 0.975), names = FALSE))

}

Displacements_result_prun <- data.frame(Bin = c("Carnian", "Norian-Rhaetian", "pre-Toarcian", "Toarcian-MJ"),

Disp = c(displ_prun[[1]], displ_prun[[2]], displ_prun[[3]], displ_prun[[4]]),

LowerLimit = c(q_displ_prun[[1]][1], q_displ_prun[[2]][1], q_displ_prun[[3]][1], q_displ_prun[[4]][1]),

UpperLimit = c(q_displ_prun[[1]][2], q_displ_prun[[2]][2], q_displ_prun[[3]][2], q_displ_prun[[4]][2]))

#pdf(file = "graphics/Displacements_from_centroid_without_outliers.pdf", width = 6, height = 6)

Displacements_result_prun$Bin <- factor(Displacements_result_prun$Bin, levels = c("Carnian","Norian-Rhaetian","pre-Toarcian","Toarcian-MJ"))

ggplot(Displacements_result_prun) +

geom_errorbar(aes(x = Bin, ymin = LowerLimit, ymax = UpperLimit, col = Bin),

width = 0.25, lwd = 1) +

geom_point(aes(x = Bin, y = Disp, col = Bin)) +

theme_bw() +

theme(legend.position = "none")

#dev.off()

#Calculate displacements from centroid without outliers and Barapasaurus and Isanosaurus

displ_prun_Barapa_Isano <- list()

for (i in 1:length(bins_prun_Barapa_Isano)) {

displ_prun_Barapa_Isano[[i]] <- mean(displacements(pcoa_result_prun_Barapa_Isano$vectors.cor[bins_prun_Barapa_Isano[[i]], 1:17]))

}

boot_displ_prun_Barapa_Isano <- list()

disp_ds_prun_Barapa_Isano <- list()

q_displ_prun_Barapa_Isano <- list()

for (j in 1:length(bins_prun_Barapa_Isano)) {

boot_displ_prun_Barapa_Isano[[j]] <- boot.matrix(data = pcoa_result_prun_Barapa_Isano$vectors.cor[bins_prun_Barapa_Isano[[j]], 1:17],

bootstraps = 9999)

disp_ds_prun_Barapa_Isano[[j]] <- apply(X = boot_displ_prun_Barapa_Isano[[j]]$subsets[[1]][[2]],

MARGIN = 2,

FUN = function(x) mean(displacements(pcoa_result_prun_Barapa_Isano$vectors.cor[bins_prun_Barapa_Isano[[j]], ][x, 1:17])))

q_displ_prun_Barapa_Isano[[j]] <- sort(quantile(disp_ds_prun_Barapa_Isano[[j]], c(0.025, 0.975), names = FALSE))

}

Displacements_result_prun_Barapa_Isano <- data.frame(Bin = c("Carnian", "Norian-Rhaetian", "pre-Toarcian", "Toarcian-MJ"),

Disp = c(displ_prun_Barapa_Isano[[1]], displ_prun_Barapa_Isano[[2]], displ_prun_Barapa_Isano[[3]], displ_prun_Barapa_Isano[[4]]),

LowerLimit = c(q_displ_prun_Barapa_Isano[[1]][1], q_displ_prun_Barapa_Isano[[2]][1], q_displ_prun_Barapa_Isano[[3]][1], q_displ_prun_Barapa_Isano[[4]][1]),

UpperLimit = c(q_displ_prun_Barapa_Isano[[1]][2], q_displ_prun_Barapa_Isano[[2]][2], q_displ_prun_Barapa_Isano[[3]][2], q_displ_prun_Barapa_Isano[[4]][2]))

#pdf(file = "graphics/Displacements_from_centroid_without_outliers_and_Barapasaurus_and_Isanosaurus.pdf", width = 6, height = 6)

Displacements_result_prun_Barapa_Isano$Bin <- factor(Displacements_result_prun_Barapa_Isano$Bin, levels = c("Carnian","Norian-Rhaetian","pre-Toarcian","Toarcian-MJ"))

ggplot(Displacements_result_prun_Barapa_Isano) +

geom_errorbar(aes(x = Bin, ymin = LowerLimit, ymax = UpperLimit, col = Bin),

width = 0.25, lwd = 1) +

geom_point(aes(x = Bin, y = Disp, col = Bin)) +

theme_bw() +

theme(legend.position = "none")

#dev.off()

#Displacements from the centroid of previous time bin without outliers

centroid_prun <- list()

for (k in 1:length(bins_prun)) {

centroid_prun[[k]] <- apply(pcoa_result_prun$vectors.cor[bins_prun[[k]], 1:17], 2, mean)

}

displ_A_bin_prun <- mean(displacements(pcoa_result_prun$vectors.cor[bins_prun[[1]], 1:17],

reference = centroid_prun[[1]]))

qA_displ_bin_prun <- c(displ_A_bin_prun,displ_A_bin_prun)

displ_bin_prun <- list()

boot_displ_bin_prun <- list()

disp_ds_bin_prun <- list()

q_displ_bin_prun <- list()

for (i in 2:length(bins_prun)) {

displ_bin_prun[[i]] <- mean(displacements(pcoa_result_prun$vectors.cor[bins_prun[[i]], 1:17],

reference = centroid_prun[[i-1]]))

boot_displ_bin_prun[[i]] <- boot.matrix(data = pcoa_result_prun$vectors.cor[bins_prun[[i]], 1:17],

bootstraps = 9999)

disp_ds_bin_prun[[i]] <- apply(X = boot_displ_bin_prun[[i]]$subsets[[1]][[2]],

MARGIN = 2,

FUN = function(x) mean(displacements(pcoa_result_prun$vectors.cor[bins_prun[[i]], ][x, 1:17],

reference = centroid_prun[[i-1]])))

q_displ_bin_prun[[i]] <- sort(quantile(disp_ds_bin_prun[[i]], c(0.025, 0.975), names = FALSE))

}

Displacements_previous_bin_result_prun <- data.frame(Bin = c("Carnian", "Norian-Rhaetian", "pre-Toarcian", "Toarcian-MJ"),

Disp = c(displ_A_bin_prun, displ_bin_prun[[2]], displ_bin_prun[[3]], displ_bin_prun[[4]]),

LowerLimit = c(qA_displ_bin_prun[1], q_displ_bin_prun[[2]][1], q_displ_bin_prun[[3]][1], q_displ_bin_prun[[4]][1]),

UpperLimit = c(qA_displ_bin_prun[2], q_displ_bin_prun[[2]][2], q_displ_bin_prun[[3]][2], q_displ_bin_prun[[4]][2]))

#pdf(file = "graphics/Displacements_from_previous_time_bin_without_outliers.pdf", width = 6, height = 6)

Displacements_previous_bin_result_prun$Bin <- factor(Displacements_previous_bin_result_prun$Bin, levels = c("Carnian","Norian-Rhaetian","pre-Toarcian","Toarcian-MJ"))

ggplot(Displacements_previous_bin_result_prun) +

geom_errorbar(aes(x = Bin, ymin = LowerLimit, ymax = UpperLimit, col = Bin),

width = 0.25, lwd = 1) +

geom_point(aes(x = Bin, y = Disp, col = Bin)) +

theme_bw() +

theme(legend.position = "none")

#dev.off()

#Displacements from the centroid of previous time bin without outliers and Barapasaurus and Isanosaurus

centroid_prun_Barapa_Isano <- list()

for (k in 1:length(bins_prun_Barapa_Isano)) {

centroid_prun_Barapa_Isano[[k]] <- apply(pcoa_result_prun_Barapa_Isano$vectors.cor[bins_prun_Barapa_Isano[[k]], 1:17], 2, mean)

}

displ_A_bin_prun_Barapa_Isano <- mean(displacements(pcoa_result_prun_Barapa_Isano$vectors.cor[bins_prun_Barapa_Isano[[1]], 1:17],

reference = centroid_prun_Barapa_Isano[[1]]))

qA_displ_bin_prun_Barapa_Isano <- c(displ_A_bin_prun_Barapa_Isano,displ_A_bin_prun_Barapa_Isano)

displ_bin_prun_Barapa_Isano <- list()

boot_displ_bin_prun_Barapa_Isano <- list()

disp_ds_bin_prun_Barapa_Isano <- list()

q_displ_bin_prun_Barapa_Isano <- list()

for (i in 2:length(bins_prun_Barapa_Isano)) {

displ_bin_prun_Barapa_Isano[[i]] <- mean(displacements(pcoa_result_prun_Barapa_Isano$vectors.cor[bins_prun_Barapa_Isano[[i]], 1:17],

reference = centroid_prun_Barapa_Isano[[i-1]]))

boot_displ_bin_prun_Barapa_Isano[[i]] <- boot.matrix(data = pcoa_result_prun_Barapa_Isano$vectors.cor[bins_prun_Barapa_Isano[[i]], 1:17],

bootstraps = 9999)

disp_ds_bin_prun_Barapa_Isano[[i]] <- apply(X = boot_displ_bin_prun_Barapa_Isano[[i]]$subsets[[1]][[2]],

MARGIN = 2,

FUN = function(x) mean(displacements(pcoa_result_prun_Barapa_Isano$vectors.cor[bins_prun_Barapa_Isano[[i]], ][x, 1:17],

reference = centroid_prun_Barapa_Isano[[i-1]])))

q_displ_bin_prun_Barapa_Isano[[i]] <- sort(quantile(disp_ds_bin_prun_Barapa_Isano[[i]], c(0.025, 0.975), names = FALSE))

}

Displacements_previous_bin_result_prun_Barapa_Isano <- data.frame(Bin = c("Carnian", "Norian-Rhaetian", "pre-Toarcian", "Toarcian-MJ"),

Disp = c(displ_A_bin_prun_Barapa_Isano, displ_bin_prun_Barapa_Isano[[2]], displ_bin_prun_Barapa_Isano[[3]], displ_bin_prun_Barapa_Isano[[4]]),

LowerLimit = c(qA_displ_bin_prun_Barapa_Isano[1], q_displ_bin_prun_Barapa_Isano[[2]][1], q_displ_bin_prun_Barapa_Isano[[3]][1], q_displ_bin_prun_Barapa_Isano[[4]][1]),

UpperLimit = c(qA_displ_bin_prun_Barapa_Isano[2], q_displ_bin_prun_Barapa_Isano[[2]][2], q_displ_bin_prun_Barapa_Isano[[3]][2], q_displ_bin_prun_Barapa_Isano[[4]][2]))

#pdf(file = "graphics/Displacements_from_previous_time_bin_without_outliers_and_Barapasaurus_Isanosaurus.pdf", width = 6, height = 6)

Displacements_previous_bin_result_prun_Barapa_Isano$Bin <- factor(Displacements_previous_bin_result_prun_Barapa_Isano$Bin, levels = c("Carnian","Norian-Rhaetian","pre-Toarcian","Toarcian-MJ"))

ggplot(Displacements_previous_bin_result_prun_Barapa_Isano) +

geom_errorbar(aes(x = Bin, ymin = LowerLimit, ymax = UpperLimit, col = Bin),

width = 0.25, lwd = 1) +

geom_point(aes(x = Bin, y = Disp, col = Bin)) +

theme_bw() +

theme(legend.position = "none")

#dev.off()

#Calculate displacements of locomotion styles from centroid without outliers

displ_locomotion_prun <- list()

for (i in 1:length(locomotions.PCoA_prun)) {

displ_locomotion_prun[[i]] <- mean(displacements(pcoa_result_prun$vectors.cor[locomotions.PCoA_prun[[i]], 1:17]))

}

boot_displ_locomotion_prun <- list()

disp_ds_locomotion_prun <- list()

q_displ_locomotion_prun <- list()

for (j in 1:length(locomotions.PCoA_prun)) {

boot_displ_locomotion_prun[[j]] <- boot.matrix(data = pcoa_result_prun$vectors.cor[locomotions.PCoA_prun[[j]], 1:17],

bootstraps = 9999)

disp_ds_locomotion_prun[[j]] <- apply(X = boot_displ_locomotion_prun[[j]]$subsets[[1]][[2]],

MARGIN = 2,

FUN = function(x) mean(displacements(pcoa_result_prun$vectors.cor[locomotions.PCoA_prun[[j]], ][x, 1:17])))

q_displ_locomotion_prun[[j]] <- sort(quantile(disp_ds_locomotion_prun[[j]], c(0.025, 0.975), names = FALSE))

}

Displacements_result_locomotion_prun <- data.frame(Bin = c("Bipeds","Quadrupeds"),

Disp = c(displ_locomotion_prun[[1]], displ_locomotion_prun[[2]]),

LowerLimit = c(q_displ_locomotion_prun[[1]][1], q_displ_locomotion_prun[[2]][1]),

UpperLimit = c(q_displ_locomotion_prun[[1]][2], q_displ_locomotion_prun[[2]][2]))

#pdf(file = "graphics/Displacements_from_centroid_locomotion_without_outliers.pdf", width = 6, height = 6)

Displacements_result_locomotion_prun$Bin <- factor(Displacements_result_locomotion_prun$Bin, levels = c("Bipeds","Quadrupeds"))

ggplot(Displacements_result_locomotion_prun) +

geom_errorbar(aes(x = Bin, ymin = LowerLimit, ymax = UpperLimit, col = Bin),

width = 0.25, lwd = 1) +

geom_point(aes(x = Bin, y = Disp, col = Bin)) +

theme_bw() +

theme(legend.position = "none")

#dev.off()

#Calculate displacements of locomotion styles from centroid without outliers and with reduced sample

displ_locomotion_prun_red <- list()

for (i in 1:length(locomotions.PCoA_prun_red)) {

displ_locomotion_prun_red[[i]] <- mean(displacements(pcoa_result_prun$vectors.cor[locomotions.PCoA_prun_red[[i]], 1:17]))

}

boot_displ_locomotion_prun_red <- list()

disp_ds_locomotion_prun_red <- list()

q_displ_locomotion_prun_red <- list()

for (j in 1:length(locomotions.PCoA_prun_red)) {

boot_displ_locomotion_prun_red[[j]] <- boot.matrix(data = pcoa_result_prun$vectors.cor[locomotions.PCoA_prun_red[[j]], 1:17],

bootstraps = 9999)

disp_ds_locomotion_prun_red[[j]] <- apply(X = boot_displ_locomotion_prun_red[[j]]$subsets[[1]][[2]],

MARGIN = 2,

FUN = function(x) mean(displacements(pcoa_result_prun$vectors.cor[locomotions.PCoA_prun_red[[j]], ][x, 1:17])))

q_displ_locomotion_prun_red[[j]] <- sort(quantile(disp_ds_locomotion_prun_red[[j]], c(0.025, 0.975), names = FALSE))

}

Displacements_result_locomotion_prun_red <- data.frame(Bin = c("Bipeds","Quadrupeds"),

Disp = c(displ_locomotion_prun_red[[1]], displ_locomotion_prun_red[[2]]),

LowerLimit = c(q_displ_locomotion_prun_red[[1]][1], q_displ_locomotion_prun_red[[2]][1]),

UpperLimit = c(q_displ_locomotion_prun_red[[1]][2], q_displ_locomotion_prun_red[[2]][2]))

#pdf(file = "graphics/Displacements_from_centroid_locomotion_without_outliers_reduced_sample.pdf", width = 6, height = 6)

Displacements_result_locomotion_prun_red$Bin <- factor(Displacements_result_locomotion_prun_red$Bin, levels = c("Bipeds","Quadrupeds"))

ggplot(Displacements_result_locomotion_prun_red) +

geom_errorbar(aes(x = Bin, ymin = LowerLimit, ymax = UpperLimit, col = Bin),

width = 0.25, lwd = 1) +

geom_point(aes(x = Bin, y = Disp, col = Bin)) +

theme_bw() +

theme(legend.position = "none")

#dev.off()

#Calculate displacements of locomotion styles from centroid without outliers and Barapasaurus and Isanosaurus

displ_locomotion_prun_Barapa_Isano <- list()

for (i in 1:length(locomotions.PCoA_prun_Barapa_Isano)) {

displ_locomotion_prun_Barapa_Isano[[i]] <- mean(displacements(pcoa_result_prun_Barapa_Isano$vectors.cor[locomotions.PCoA_prun_Barapa_Isano[[i]], 1:17]))

}

boot_displ_locomotion_prun_Barapa_Isano <- list()

disp_ds_locomotion_prun_Barapa_Isano <- list()

q_displ_locomotion_prun_Barapa_Isano <- list()

for (j in 1:length(locomotions.PCoA_prun_Barapa_Isano)) {

boot_displ_locomotion_prun_Barapa_Isano[[j]] <- boot.matrix(data = pcoa_result_prun_Barapa_Isano$vectors.cor[locomotions.PCoA_prun_Barapa_Isano[[j]], 1:17],

bootstraps = 9999)

disp_ds_locomotion_prun_Barapa_Isano[[j]] <- apply(X = boot_displ_locomotion_prun_Barapa_Isano[[j]]$subsets[[1]][[2]],

MARGIN = 2,

FUN = function(x) mean(displacements(pcoa_result_prun_Barapa_Isano$vectors.cor[locomotions.PCoA_prun_Barapa_Isano[[j]], ][x, 1:17])))

q_displ_locomotion_prun_Barapa_Isano[[j]] <- sort(quantile(disp_ds_locomotion_prun_Barapa_Isano[[j]], c(0.025, 0.975), names = FALSE))

}

Displacements_result_locomotion_prun_Barapa_Isano <- data.frame(Bin = c("Bipeds","Quadrupeds"),

Disp = c(displ_locomotion_prun_Barapa_Isano[[1]], displ_locomotion_prun_Barapa_Isano[[2]]),

LowerLimit = c(q_displ_locomotion_prun_Barapa_Isano[[1]][1], q_displ_locomotion_prun_Barapa_Isano[[2]][1]),

UpperLimit = c(q_displ_locomotion_prun_Barapa_Isano[[1]][2], q_displ_locomotion_prun_Barapa_Isano[[2]][2]))

#pdf(file = "graphics/Displacements_from_centroid_locomotion_without_outliers_and_Barapasaurus_Isanosaurus.pdf", width = 6, height = 6)

Displacements_result_locomotion_prun_Barapa_Isano$Bin <- factor(Displacements_result_locomotion_prun_Barapa_Isano$Bin, levels = c("Bipeds","Quadrupeds"))

ggplot(Displacements_result_locomotion_prun_Barapa_Isano) +

geom_errorbar(aes(x = Bin, ymin = LowerLimit, ymax = UpperLimit, col = Bin),

width = 0.25, lwd = 1) +

geom_point(aes(x = Bin, y = Disp, col = Bin)) +

theme_bw() +

theme(legend.position = "none")

#dev.off()

#Calculate displacements of locomotion styles from centroid without outliers and Barapasaurus and Isanosaurus with reduced sample

displ_locomotion_prun_Barapa_Isano_red <- list()

for (i in 1:length(locomotions.PCoA_prun_Barapa_Isano_red)) {

displ_locomotion_prun_Barapa_Isano_red[[i]] <- mean(displacements(pcoa_result_prun_Barapa_Isano$vectors.cor[locomotions.PCoA_prun_Barapa_Isano_red[[i]], 1:17]))

}

boot_displ_locomotion_prun_Barapa_Isano_red <- list()

disp_ds_locomotion_prun_Barapa_Isano_red <- list()

q_displ_locomotion_prun_Barapa_Isano_red <- list()

for (j in 1:length(locomotions.PCoA_prun_Barapa_Isano_red)) {

boot_displ_locomotion_prun_Barapa_Isano_red[[j]] <- boot.matrix(data = pcoa_result_prun_Barapa_Isano$vectors.cor[locomotions.PCoA_prun_Barapa_Isano_red[[j]], 1:17],

bootstraps = 9999)

disp_ds_locomotion_prun_Barapa_Isano_red[[j]] <- apply(X = boot_displ_locomotion_prun_Barapa_Isano_red[[j]]$subsets[[1]][[2]],

MARGIN = 2,

FUN = function(x) mean(displacements(pcoa_result_prun_Barapa_Isano$vectors.cor[locomotions.PCoA_prun_Barapa_Isano_red[[j]], ][x, 1:17])))

q_displ_locomotion_prun_Barapa_Isano_red[[j]] <- sort(quantile(disp_ds_locomotion_prun_Barapa_Isano_red[[j]], c(0.025, 0.975), names = FALSE))

}

Displacements_result_locomotion_prun_Barapa_Isano_red <- data.frame(Bin = c("Bipeds","Quadrupeds"),

Disp = c(displ_locomotion_prun_Barapa_Isano_red[[1]], displ_locomotion_prun_Barapa_Isano_red[[2]]),

LowerLimit = c(q_displ_locomotion_prun_Barapa_Isano_red[[1]][1], q_displ_locomotion_prun_Barapa_Isano_red[[2]][1]),

UpperLimit = c(q_displ_locomotion_prun_Barapa_Isano_red[[1]][2], q_displ_locomotion_prun_Barapa_Isano_red[[2]][2]))

#pdf(file = "graphics/Displacements_from_centroid_locomotion_without_outliers_and_Barapasaurus_Isanosaurus_reduced_sample.pdf", width = 6, height = 6)

Displacements_result_locomotion_prun_Barapa_Isano_red$Bin <- factor(Displacements_result_locomotion_prun_Barapa_Isano_red$Bin, levels = c("Bipeds","Quadrupeds"))

ggplot(Displacements_result_locomotion_prun_Barapa_Isano_red) +

geom_errorbar(aes(x = Bin, ymin = LowerLimit, ymax = UpperLimit, col = Bin),

width = 0.25, lwd = 1) +

geom_point(aes(x = Bin, y = Disp, col = Bin)) +

theme_bw() +

theme(legend.position = "none")

#dev.off()

###################################################################

#Regression-based phylogenetic comparisons

trees <- read.tree(file = "trees.tre")

ages <- read.table("ages_R1.txt",

row.names = 1,

header = TRUE,

sep = "\t") #This file also has ages of outgroups

time.trees <- list()

for (i in 1:length(trees)) {

time.trees[[i]] <- timePaleoPhy(tree = trees[[i]],

timeData = ages,

type="mbl",

vartime=0.1)

}

###Prune non-sauropodomorph taxa

time.trees.drop <- list()

for (j in 1:length(trees)) {

time.trees.drop[[j]] <- drop.tip(phy = time.trees[[j]],

tip = c("Euparkeria","Crurotarsi","Marasuchus","Ornithischia","Agnosphitys","Silesaurus","Neotheropoda","Staurikosaurus","Chindesaurus","Herrerasaurus","Guaibasaurus","Neosauropoda"))

}

###Prune trimmed taxa before the ordination

for (k in 1:length(trees)) {

time.trees.drop[[k]] <- drop.tip(phy = time.trees.drop[[k]],

tip = trim_dist_matrix$removed_taxa)

}

###Generate trees with all branch lengths equal to 1

time.trees.1branch <- time.trees.drop

for (m in 1:length(trees)) {

time.trees.1branch[[m]]$edge.length <- rep(1,length(time.trees.1branch[[m]]$edge.length))

}

###Generate model data for femoral length, body mass, log(Hc)/log(Fc) and area

model_data <- data[,c(1,4:7)]

model_data_red <- na.omit(model_data)

length_data <- log(model_data_red$FL)

names(length_data) <- model_data_red$Taxon

mass_data <- log(model_data_red$Mass)

names(mass_data) <- model_data_red$Taxon

circum.ratio_data <- model_data_red$log.Hc..log.Fc.

names(circum.ratio_data) <- model_data_red$Taxon

biogeo_data <- model_data_red$Area

names(biogeo_data) <- model_data_red$Taxon

pcoa_result_vectors_prun <- pcoa_result$vectors.cor[intersect(names(pcoa_result$vectors.cor[,1]),model_data_red$Taxon),]

dataset <- data.frame("PCo1" = pcoa_result_vectors_prun[,1],

"FL" = length_data,

"BM" = mass_data,

"ratio" = circum.ratio_data,

"Area" = biogeo_data)

NA_taxa <- setdiff(time.trees.drop[[1]]$tip.label,model_data_red$Taxon)

for (l in 1:length(trees)) {

time.trees.drop[[l]] <- drop.tip(phy = time.trees.drop[[l]],

tip = NA_taxa)

}

NA_taxa_1my <- setdiff(time.trees.1branch[[1]]$tip.label,model_data_red$Taxon)

time.trees.1branch_drop <- NULL

for (l in 1:length(trees)) {

time.trees.1branch_drop[[l]] <- drop.tip(phy = time.trees.1branch[[l]],

tip = NA_taxa_1my)

}

for (m in 1:length(trees)) {

time.trees.1branch_drop[[m]]$edge.length <- rep(1,length(time.trees.1branch_drop[[m]]$edge.length))

}

###########LOAD RESULTS IF YOU HAVE ALREADY RUN THE pGLSs#######################

load("./output/PCos_FL.RData")

load("./output/PCos_BM.RData")

load("./output/PCos_ratio.RData")

load("./output/PCos_BIOGEO.RData")

load("./output/PCos_phylo.RData")

load("./output/PCos_FL_ratio.RData")

load("./output/PCos_FL_BIOGEO.RData")

load("./output/PCos_FL_phylo.RData")

load("./output/PCos_FL_BIOGEO_phylo.RData")

load("./output/PCos_BM_ratio.RData")

load("./output/PCos_BM_biogeo.RData")

load("./output/PCos_BM_phylo.RData")

load("./output/PCos_BM_ratio_biog.RData")

load("./output/PCos_BM_ratio_phy.RData")

load("./output/PCos_biogeo_phy.RData")

load("./output/2PCos_FL.RData")

load("./output/2PCos_BM.RData")

load("./output/2PCos_BIOGEO.RData")

load("./output/2PCos_phylo.RData")

load("./output/2PCos_locom.RData")

load("./output/2PCos_FL_BIOGEO.RData")

load("./output/2PCos_FL_phylo.RData")

load("./output/2PCos_FL_locom.RData")

load("./output/2PCos_FL_BIOGEO_phylo.RData")

load("./output/2PCos_FL_phylo_locom.RData")

load("./output/2PCos_BM_biogeo.RData")

load("./output/2PCos_BM_phylo.RData")

load("./output/2PCos_BM_locom.RData")

load("./output/2PCos_biogeo_phy.RData")

load("./output/2PCos_biogeo_locom.RData")

load("./output/2PCos_phy_locom.RData")

########################################

###Test PCos versus femoral length

pgls.fit_PCos_FL = list()

for (m in 1:length(trees)) {

pgls.fit_PCos_FL[[m]] <- procD.pgls(pcoa_result_vectors_prun[,1:3]~length_data,

phy = time.trees.drop[[m]],

data = dataset,

iter = 999,

SS.type = "II")

}

Rsq_PCos_FL <- matrix(ncol = dim(pgls.fit_PCos_FL[[1]]$aov.table)[1]-2,

nrow = length(trees))

colnames(Rsq_PCos_FL) <- rownames(pgls.fit_PCos_FL[[1]]$aov.table)[1:ncol(Rsq_PCos_FL)]

for (p in 1:ncol(Rsq_PCos_FL)) {

for (n in 1:length(trees)) {

Rsq_PCos_FL[n,p] <- pgls.fit_PCos_FL[[n]]$aov.table$Rsq[p]

}

}

p.value_PCos_FL <- matrix(ncol = dim(pgls.fit_PCos_FL[[1]]$aov.table)[1]-2,

nrow = length(trees))

count.p.value_PCos_FL <- matrix(ncol = dim(pgls.fit_PCos_FL[[1]]$aov.table)[1]-2,

nrow = 1)

ratio_p.value_PCos_FL <- matrix(ncol = dim(pgls.fit_PCos_FL[[1]]$aov.table)[1]-2,

nrow = 1)

colnames(ratio_p.value_PCos_FL) <- rownames(pgls.fit_PCos_FL[[1]]$aov.table)[1:ncol(Rsq_PCos_FL)]

for (p in 1:ncol(Rsq_PCos_FL)) {

for (o in 1:length(trees)) {

p.value_PCos_FL[o,p] <- pgls.fit_PCos_FL[[o]]$aov.table$`Pr(>F)`[p]<0.05

}

count.p.value_PCos_FL[,p] <- sum(p.value_PCos_FL[,p], na.rm=TRUE)

ratio_p.value_PCos_FL[,p] <- count.p.value_PCos_FL[,p]/length(trees)

}

#save(Rsq_PCos_FL, count.p.value_PCos_FL, ratio_p.value_PCos_FL, file = "./output/PCos_FL.RData")

#load("./output/PCos_FL.RData")

###Test PCos versus body mass

pgls.fit_PCos_BM = list()

for (m in 1:length(trees)) {

pgls.fit_PCos_BM[[m]] <- procD.pgls(pcoa_result_vectors_prun[,1:3]~mass_data,

phy = time.trees.drop[[m]],

data = dataset,

iter = 999,

SS.type = "II")

}

Rsq_PCos_BM <- matrix(ncol = dim(pgls.fit_PCos_BM[[1]]$aov.table)[1]-2,

nrow = length(trees))

colnames(Rsq_PCos_BM) <- rownames(pgls.fit_PCos_BM[[1]]$aov.table)[1:ncol(Rsq_PCos_BM)]

for (p in 1:ncol(Rsq_PCos_BM)) {

for (n in 1:length(trees)) {

Rsq_PCos_BM[n,p] <- pgls.fit_PCos_BM[[n]]$aov.table$Rsq[p]

}

}

p.value_PCos_BM <- matrix(ncol = dim(pgls.fit_PCos_BM[[1]]$aov.table)[1]-2,

nrow = length(trees))

count.p.value_PCos_BM <- matrix(ncol = dim(pgls.fit_PCos_BM[[1]]$aov.table)[1]-2,

nrow = 1)

ratio_p.value_PCos_BM <- matrix(ncol = dim(pgls.fit_PCos_BM[[1]]$aov.table)[1]-2,

nrow = 1)

colnames(ratio_p.value_PCos_BM) <- rownames(pgls.fit_PCos_BM[[1]]$aov.table)[1:ncol(Rsq_PCos_BM)]

for (p in 1:ncol(Rsq_PCos_BM)) {

for (o in 1:length(trees)) {

p.value_PCos_BM[o,p] <- pgls.fit_PCos_BM[[o]]$aov.table$`Pr(>F)`[p]<0.05

}

count.p.value_PCos_BM[,p] <- sum(p.value_PCos_BM[,p], na.rm=TRUE)

ratio_p.value_PCos_BM[,p] <- count.p.value_PCos_BM[,p]/length(trees)

}

#save(Rsq_PCos_BM, count.p.value_PCos_BM, ratio_p.value_PCos_BM, file = "./output/PCos_BM.RData")

#load("./output/PCos_BM.RData")

###Test PCos versus circumference ratio

pgls.fit_PCos_ratio = list()

for (m in 1:length(trees)) {

pgls.fit_PCos_ratio[[m]] <- procD.pgls(pcoa_result_vectors_prun[,1:3]~circum.ratio_data,

phy = time.trees.drop[[m]],

data = dataset,

iter = 999,

SS.type = "II")

}

Rsq_PCos_ratio <- matrix(ncol = dim(pgls.fit_PCos_ratio[[1]]$aov.table)[1]-2,

nrow = length(trees))

colnames(Rsq_PCos_ratio) <- rownames(pgls.fit_PCos_ratio[[1]]$aov.table)[1:ncol(Rsq_PCos_ratio)]

for (p in 1:ncol(Rsq_PCos_ratio)) {

for (n in 1:length(trees)) {

Rsq_PCos_ratio[n,p] <- pgls.fit_PCos_ratio[[n]]$aov.table$Rsq[p]

}

}

p.value_PCos_ratio <- matrix(ncol = dim(pgls.fit_PCos_ratio[[1]]$aov.table)[1]-2,

nrow = length(trees))

count.p.value_PCos_ratio <- matrix(ncol = dim(pgls.fit_PCos_ratio[[1]]$aov.table)[1]-2,

nrow = 1)

ratio_p.value_PCos_ratio <- matrix(ncol = dim(pgls.fit_PCos_ratio[[1]]$aov.table)[1]-2,

nrow = 1)

colnames(ratio_p.value_PCos_ratio) <- rownames(pgls.fit_PCos_ratio[[1]]$aov.table)[1:ncol(Rsq_PCos_ratio)]

for (p in 1:ncol(Rsq_PCos_ratio)) {

for (o in 1:length(trees)) {

p.value_PCos_ratio[o,p] <- pgls.fit_PCos_ratio[[o]]$aov.table$`Pr(>F)`[p]<0.05

}

count.p.value_PCos_ratio[,p] <- sum(p.value_PCos_ratio[,p], na.rm=TRUE)

ratio_p.value_PCos_ratio[,p] <- count.p.value_PCos_ratio[,p]/length(trees)

}

#save(Rsq_PCos_ratio, count.p.value_PCos_ratio, ratio_p.value_PCos_ratio, file = "./output/PCos_ratio.RData")

#load("./output/PCos_ratio.RData")

###Test PCos versus biogeographic data

pgls.fit_PCos_BIOGEO = list()

for (m in 1:length(trees)) {

pgls.fit_PCos_BIOGEO[[m]] <- procD.pgls(pcoa_result_vectors_prun[,1:3]~biogeo_data,

phy = time.trees.drop[[m]],

data = dataset,

iter = 999,

SS.type = "II")

}

Rsq_PCos_BIOGEO <- matrix(ncol = dim(pgls.fit_PCos_BIOGEO[[1]]$aov.table)[1]-2,

nrow = length(trees))

colnames(Rsq_PCos_BIOGEO) <- rownames(pgls.fit_PCos_BIOGEO[[1]]$aov.table)[1:ncol(Rsq_PCos_BIOGEO)]

for (p in 1:ncol(Rsq_PCos_BIOGEO)) {

for (n in 1:length(trees)) {

Rsq_PCos_BIOGEO[n,p] <- pgls.fit_PCos_BIOGEO[[n]]$aov.table$Rsq[p]

}

}

p.value_PCos_BIOGEO <- matrix(ncol = dim(pgls.fit_PCos_BIOGEO[[1]]$aov.table)[1]-2,

nrow = length(trees))

count.p.value_PCos_BIOGEO <- matrix(ncol = dim(pgls.fit_PCos_BIOGEO[[1]]$aov.table)[1]-2,

nrow = 1)

ratio_p.value_PCos_BIOGEO <- matrix(ncol = dim(pgls.fit_PCos_BIOGEO[[1]]$aov.table)[1]-2,

nrow = 1)

colnames(ratio_p.value_PCos_BIOGEO) <- rownames(pgls.fit_PCos_BIOGEO[[1]]$aov.table)[1:ncol(Rsq_PCos_BIOGEO)]

for (p in 1:ncol(Rsq_PCos_BIOGEO)) {

for (o in 1:length(trees)) {

p.value_PCos_BIOGEO[o,p] <- pgls.fit_PCos_BIOGEO[[o]]$aov.table$`Pr(>F)`[p]<0.05

}

count.p.value_PCos_BIOGEO[,p] <- sum(p.value_PCos_BIOGEO[,p], na.rm=TRUE)

ratio_p.value_PCos_BIOGEO[,p] <- count.p.value_PCos_BIOGEO[,p]/length(trees)

}

#save(Rsq_PCos_BIOGEO, count.p.value_PCos_BIOGEO, ratio_p.value_PCos_BIOGEO, file = "./output/PCos_BIOGEO.RData")

#load("./output/PCos_BIOGEO.RData")

###Test PCos versus phylogeny

phylo_structure <- NULL

pgls.fit_PCos_phylo = list()

for (m in 1:length(trees)) {

phylo_structure <- diag(vcv.phylo(time.trees.1branch_drop[[m]]))

pgls.fit_PCos_phylo[[m]] <- procD.pgls(pcoa_result_vectors_prun[,1:3]~phylo_structure,

phy = time.trees.drop[[m]],

data = dataset,

iter = 999,

SS.type = "II")

}

Rsq_PCos_phylo <- matrix(ncol = dim(pgls.fit_PCos_phylo[[1]]$aov.table)[1]-2,

nrow = length(trees))

colnames(Rsq_PCos_phylo) <- rownames(pgls.fit_PCos_phylo[[1]]$aov.table)[1:ncol(Rsq_PCos_phylo)]

for (p in 1:ncol(Rsq_PCos_phylo)) {

for (n in 1:length(trees)) {

Rsq_PCos_phylo[n,p] <- pgls.fit_PCos_phylo[[n]]$aov.table$Rsq[p]

}

}

p.value_PCos_phylo <- matrix(ncol = dim(pgls.fit_PCos_phylo[[1]]$aov.table)[1]-2,

nrow = length(trees))

count.p.value_PCos_phylo <- matrix(ncol = dim(pgls.fit_PCos_phylo[[1]]$aov.table)[1]-2,

nrow = 1)

ratio_p.value_PCos_phylo <- matrix(ncol = dim(pgls.fit_PCos_phylo[[1]]$aov.table)[1]-2,

nrow = 1)

colnames(ratio_p.value_PCos_phylo) <- rownames(pgls.fit_PCos_phylo[[1]]$aov.table)[1:ncol(Rsq_PCos_phylo)]

for (p in 1:ncol(Rsq_PCos_phylo)) {

for (o in 1:length(trees)) {

p.value_PCos_phylo[o,p] <- pgls.fit_PCos_phylo[[o]]$aov.table$`Pr(>F)`[p]<0.05

}

count.p.value_PCos_phylo[,p] <- sum(p.value_PCos_phylo[,p], na.rm=TRUE)

ratio_p.value_PCos_phylo[,p] <- count.p.value_PCos_phylo[,p]/length(trees)

}

#save(Rsq_PCos_phylo, count.p.value_PCos_phylo, ratio_p.value_PCos_phylo, file = "./output/PCos_phylo.RData")

#load("./output/PCos_phylo.RData")

###Test PCos versus femoral length+ratio

pgls.fit_PCos_FL_ratio = list()

for (m in 1:length(trees)) {

pgls.fit_PCos_FL_ratio[[m]] <- procD.pgls(pcoa_result_vectors_prun[,1:3]~circum.ratio_data+length_data,

phy = time.trees.drop[[m]],

data = dataset,

iter = 999,

SS.type = "II")

}

Rsq_PCos_FL_ratio <- matrix(ncol = dim(pgls.fit_PCos_FL_ratio[[1]]$aov.table)[1]-2,

nrow = length(trees))

colnames(Rsq_PCos_FL_ratio) <- rownames(pgls.fit_PCos_FL_ratio[[1]]$aov.table)[1:ncol(Rsq_PCos_FL_ratio)]

for (p in 1:ncol(Rsq_PCos_FL_ratio)) {

for (n in 1:length(trees)) {

Rsq_PCos_FL_ratio[n,p] <- pgls.fit_PCos_FL_ratio[[n]]$aov.table$Rsq[p]

}

}

p.value_PCos_FL_ratio <- matrix(ncol = dim(pgls.fit_PCos_FL_ratio[[1]]$aov.table)[1]-2,

nrow = length(trees))

count.p.value_PCos_FL_ratio <- matrix(ncol = dim(pgls.fit_PCos_FL_ratio[[1]]$aov.table)[1]-2,

nrow = 1)

ratio_p.value_PCos_FL_ratio <- matrix(ncol = dim(pgls.fit_PCos_FL_ratio[[1]]$aov.table)[1]-2,

nrow = 1)

colnames(ratio_p.value_PCos_FL_ratio) <- rownames(pgls.fit_PCos_FL_ratio[[1]]$aov.table)[1:ncol(Rsq_PCos_FL_ratio)]

for (p in 1:ncol(Rsq_PCos_FL_ratio)) {

for (o in 1:length(trees)) {

p.value_PCos_FL_ratio[o,p] <- pgls.fit_PCos_FL_ratio[[o]]$aov.table$`Pr(>F)`[p]<0.05

}

count.p.value_PCos_FL_ratio[,p] <- sum(p.value_PCos_FL_ratio[,p], na.rm=TRUE)

ratio_p.value_PCos_FL_ratio[,p] <- count.p.value_PCos_FL_ratio[,p]/length(trees)

}

#save(Rsq_PCos_FL_ratio, count.p.value_PCos_FL_ratio, ratio_p.value_PCos_FL_ratio, file = "./output/PCos_FL_ratio.RData")

#load("./output/PCos_FL_ratio.RData")

###Test PCos versus femoral length+biogeographic data

pgls.fit_PCos_FL_BIOGEO = list()

for (m in 1:length(trees)) {

pgls.fit_PCos_FL_BIOGEO[[m]] <- procD.pgls(pcoa_result_vectors_prun[,1:3]~length_data+biogeo_data,

phy = time.trees.drop[[m]],

data = dataset,

iter = 999,

SS.type = "II")

}

Rsq_PCos_FL_BIOGEO <- matrix(ncol = dim(pgls.fit_PCos_FL_BIOGEO[[1]]$aov.table)[1]-2,

nrow = length(trees))

colnames(Rsq_PCos_FL_BIOGEO) <- rownames(pgls.fit_PCos_FL_BIOGEO[[1]]$aov.table)[1:ncol(Rsq_PCos_FL_BIOGEO)]

for (p in 1:ncol(Rsq_PCos_FL_BIOGEO)) {

for (n in 1:length(trees)) {

Rsq_PCos_FL_BIOGEO[n,p] <- pgls.fit_PCos_FL_BIOGEO[[n]]$aov.table$Rsq[p]

}

}

p.value_PCos_FL_BIOGEO <- matrix(ncol = dim(pgls.fit_PCos_FL_BIOGEO[[1]]$aov.table)[1]-2,

nrow = length(trees))

count.p.value_PCos_FL_BIOGEO <- matrix(ncol = dim(pgls.fit_PCos_FL_BIOGEO[[1]]$aov.table)[1]-2,

nrow = 1)

ratio_p.value_PCos_FL_BIOGEO <- matrix(ncol = dim(pgls.fit_PCos_FL_BIOGEO[[1]]$aov.table)[1]-2,

nrow = 1)

colnames(ratio_p.value_PCos_FL_BIOGEO) <- rownames(pgls.fit_PCos_FL_BIOGEO[[1]]$aov.table)[1:ncol(Rsq_PCos_FL_BIOGEO)]

for (p in 1:ncol(Rsq_PCos_FL_BIOGEO)) {

for (o in 1:length(trees)) {

p.value_PCos_FL_BIOGEO[o,p] <- pgls.fit_PCos_FL_BIOGEO[[o]]$aov.table$`Pr(>F)`[p]<0.05

}

count.p.value_PCos_FL_BIOGEO[,p] <- sum(p.value_PCos_FL_BIOGEO[,p], na.rm=TRUE)

ratio_p.value_PCos_FL_BIOGEO[,p] <- count.p.value_PCos_FL_BIOGEO[,p]/length(trees)

}

#save(Rsq_PCos_FL_BIOGEO, count.p.value_PCos_FL_BIOGEO, ratio_p.value_PCos_FL_BIOGEO, file = "./output/PCos_FL_BIOGEO.RData")

#load("./output/PCos_FL_BIOGEO.RData")

###Test PCos versus femoral length+phylogeny

phylo_structure <- NULL

pgls.fit_PCos_FL_phylo = list()

for (m in 1:length(trees)) {

phylo_structure <- diag(vcv.phylo(time.trees.1branch_drop[[m]]))

pgls.fit_PCos_FL_phylo[[m]] <- procD.pgls(pcoa_result_vectors_prun[,1:3]~length_data+phylo_structure,

phy = time.trees.drop[[m]],

data = dataset,

iter = 999,

SS.type = "II")

}

Rsq_PCos_FL_phylo <- matrix(ncol = dim(pgls.fit_PCos_FL_phylo[[1]]$aov.table)[1]-2,

nrow = length(trees))

colnames(Rsq_PCos_FL_phylo) <- rownames(pgls.fit_PCos_FL_phylo[[1]]$aov.table)[1:ncol(Rsq_PCos_FL_phylo)]

for (p in 1:ncol(Rsq_PCos_FL_phylo)) {

for (n in 1:length(trees)) {

Rsq_PCos_FL_phylo[n,p] <- pgls.fit_PCos_FL_phylo[[n]]$aov.table$Rsq[p]

}

}

p.value_PCos_FL_phylo <- matrix(ncol = dim(pgls.fit_PCos_FL_phylo[[1]]$aov.table)[1]-2,

nrow = length(trees))

count.p.value_PCos_FL_phylo <- matrix(ncol = dim(pgls.fit_PCos_FL_phylo[[1]]$aov.table)[1]-2,

nrow = 1)

ratio_p.value_PCos_FL_phylo <- matrix(ncol = dim(pgls.fit_PCos_FL_phylo[[1]]$aov.table)[1]-2,

nrow = 1)

colnames(ratio_p.value_PCos_FL_phylo) <- rownames(pgls.fit_PCos_FL_phylo[[1]]$aov.table)[1:ncol(Rsq_PCos_FL_phylo)]

for (p in 1:ncol(Rsq_PCos_FL_phylo)) {

for (o in 1:length(trees)) {

p.value_PCos_FL_phylo[o,p] <- pgls.fit_PCos_FL_phylo[[o]]$aov.table$`Pr(>F)`[p]<0.05

}

count.p.value_PCos_FL_phylo[,p] <- sum(p.value_PCos_FL_phylo[,p], na.rm=TRUE)

ratio_p.value_PCos_FL_phylo[,p] <- count.p.value_PCos_FL_phylo[,p]/length(trees)

}

#save(Rsq_PCos_FL_phylo, count.p.value_PCos_FL_phylo, ratio_p.value_PCos_FL_phylo, file = "./output/PCos_FL_phylo.RData")

#load("./output/PCos_FL_phylo.RData")

###Test PCos versus femoral length+biogeographic data+phylogeny

phylo_structure <- NULL

pgls.fit_PCos_FL_BIOGEO_phylo = list()

for (m in 1:length(trees)) {

phylo_structure <- diag(vcv.phylo(time.trees.1branch_drop[[m]]))

pgls.fit_PCos_FL_BIOGEO_phylo[[m]] <- procD.pgls(pcoa_result_vectors_prun[,1:3]~length_data+biogeo_data+phylo_structure,

phy = time.trees.drop[[m]],

iter = 999,

SS.type = "II")

}

Rsq_PCos_FL_BIOGEO_phylo <- matrix(ncol = dim(pgls.fit_PCos_FL_BIOGEO_phylo[[1]]$aov.table)[1]-2,

nrow = length(trees))

colnames(Rsq_PCos_FL_BIOGEO_phylo) <- rownames(pgls.fit_PCos_FL_BIOGEO_phylo[[1]]$aov.table)[1:ncol(Rsq_PCos_FL_BIOGEO_phylo)]

for (p in 1:ncol(Rsq_PCos_FL_BIOGEO_phylo)) {

for (n in 1:length(trees)) {

Rsq_PCos_FL_BIOGEO_phylo[n,p] <- pgls.fit_PCos_FL_BIOGEO_phylo[[n]]$aov.table$Rsq[p]

}

}

p.value_PCos_FL_BIOGEO_phylo <- matrix(ncol = dim(pgls.fit_PCos_FL_BIOGEO_phylo[[1]]$aov.table)[1]-2,

nrow = length(trees))

count.p.value_PCos_FL_BIOGEO_phylo <- matrix(ncol = dim(pgls.fit_PCos_FL_BIOGEO_phylo[[1]]$aov.table)[1]-2,

nrow = 1)

ratio_p.value_PCos_FL_BIOGEO_phylo <- matrix(ncol = dim(pgls.fit_PCos_FL_BIOGEO_phylo[[1]]$aov.table)[1]-2,

nrow = 1)

colnames(ratio_p.value_PCos_FL_BIOGEO_phylo) <- rownames(pgls.fit_PCos_FL_BIOGEO_phylo[[1]]$aov.table)[1:ncol(Rsq_PCos_FL_BIOGEO_phylo)]

for (p in 1:ncol(Rsq_PCos_FL_BIOGEO_phylo)) {

for (o in 1:length(trees)) {

p.value_PCos_FL_BIOGEO_phylo[o,p] <- pgls.fit_PCos_FL_BIOGEO_phylo[[o]]$aov.table$`Pr(>F)`[p]<0.05

}

count.p.value_PCos_FL_BIOGEO_phylo[,p] <- sum(p.value_PCos_FL_BIOGEO_phylo[,p], na.rm=TRUE)

ratio_p.value_PCos_FL_BIOGEO_phylo[,p] <- count.p.value_PCos_FL_BIOGEO_phylo[,p]/length(trees)

}

#save(Rsq_PCos_FL_BIOGEO_phylo, count.p.value_PCos_FL_BIOGEO_phylo, ratio_p.value_PCos_FL_BIOGEO_phylo, file = "./output/PCos_FL_BIOGEO_phylo.RData")

#load("./output/PCos_FL_BIOGEO_phylo.RData")

###Test PCos versus body mass+ratio

pgls.fit_PCos_BM_ratio = list()

for (m in 1:length(trees)) {

pgls.fit_PCos_BM_ratio[[m]] <- procD.pgls(pcoa_result_vectors_prun[,1:3]~circum.ratio_data+mass_data,

phy = time.trees.drop[[m]],

data = dataset,

iter = 999,

SS.type = "II")

}

residuals_PCos_BM_ratio <- pgls.fit_PCos_BM_ratio[[1]]$pgls.residuals

Rsq_PCos_BM_ratio <- matrix(ncol = dim(pgls.fit_PCos_BM_ratio[[1]]$aov.table)[1]-2,

nrow = length(trees))

colnames(Rsq_PCos_BM_ratio) <- rownames(pgls.fit_PCos_BM_ratio[[1]]$aov.table)[1:ncol(Rsq_PCos_BM_ratio)]

for (p in 1:ncol(Rsq_PCos_BM_ratio)) {

for (n in 1:length(trees)) {

Rsq_PCos_BM_ratio[n,p] <- pgls.fit_PCos_BM_ratio[[n]]$aov.table$Rsq[p]

}

}

p.value_PCos_BM_ratio <- matrix(ncol = dim(pgls.fit_PCos_BM_ratio[[1]]$aov.table)[1]-2,

nrow = length(trees))

count.p.value_PCos_BM_ratio <- matrix(ncol = dim(pgls.fit_PCos_BM_ratio[[1]]$aov.table)[1]-2,

nrow = 1)

ratio_p.value_PCos_BM_ratio <- matrix(ncol = dim(pgls.fit_PCos_BM_ratio[[1]]$aov.table)[1]-2,

nrow = 1)

colnames(ratio_p.value_PCos_BM_ratio) <- rownames(pgls.fit_PCos_BM_ratio[[1]]$aov.table)[1:ncol(Rsq_PCos_BM_ratio)]

for (p in 1:ncol(Rsq_PCos_BM_ratio)) {

for (o in 1:length(trees)) {

p.value_PCos_BM_ratio[o,p] <- pgls.fit_PCos_BM_ratio[[o]]$aov.table$`Pr(>F)`[p]<0.05

}

count.p.value_PCos_BM_ratio[,p] <- sum(p.value_PCos_BM_ratio[,p], na.rm=TRUE)

ratio_p.value_PCos_BM_ratio[,p] <- count.p.value_PCos_BM_ratio[,p]/length(trees)

}

#save(Rsq_PCos_BM_ratio, count.p.value_PCos_BM_ratio, ratio_p.value_PCos_BM_ratio, residuals_PCos_BM_ratio, file = "./output/PCos_BM_ratio.RData")

#load("./output/PCos_BM_ratio.RData")

###Test PCos versus body mass+biogeography

pgls.fit_PCos_BM_biogeo = list()

for (m in 1:length(trees)) {

pgls.fit_PCos_BM_biogeo[[m]] <- procD.pgls(pcoa_result_vectors_prun[,1:3]~mass_data+biogeo_data,

phy = time.trees.drop[[m]],

data = dataset,

iter = 999,

SS.type = "II")

}

Rsq_PCos_BM_biogeo <- matrix(ncol = dim(pgls.fit_PCos_BM_biogeo[[1]]$aov.table)[1]-2,

nrow = length(trees))

colnames(Rsq_PCos_BM_biogeo) <- rownames(pgls.fit_PCos_BM_biogeo[[1]]$aov.table)[1:ncol(Rsq_PCos_BM_biogeo)]

for (p in 1:ncol(Rsq_PCos_BM_biogeo)) {

for (n in 1:length(trees)) {

Rsq_PCos_BM_biogeo[n,p] <- pgls.fit_PCos_BM_biogeo[[n]]$aov.table$Rsq[p]

}

}

p.value_PCos_BM_biogeo <- matrix(ncol = dim(pgls.fit_PCos_BM_biogeo[[1]]$aov.table)[1]-2,

nrow = length(trees))

count.p.value_PCos_BM_biogeo <- matrix(ncol = dim(pgls.fit_PCos_BM_biogeo[[1]]$aov.table)[1]-2,

nrow = 1)

ratio_p.value_PCos_BM_biogeo <- matrix(ncol = dim(pgls.fit_PCos_BM_biogeo[[1]]$aov.table)[1]-2,

nrow = 1)

colnames(ratio_p.value_PCos_BM_biogeo) <- rownames(pgls.fit_PCos_BM_biogeo[[1]]$aov.table)[1:ncol(Rsq_PCos_BM_biogeo)]

for (p in 1:ncol(Rsq_PCos_BM_biogeo)) {

for (o in 1:length(trees)) {

p.value_PCos_BM_biogeo[o,p] <- pgls.fit_PCos_BM_biogeo[[o]]$aov.table$`Pr(>F)`[p]<0.05

}

count.p.value_PCos_BM_biogeo[,p] <- sum(p.value_PCos_BM_biogeo[,p], na.rm=TRUE)

ratio_p.value_PCos_BM_biogeo[,p] <- count.p.value_PCos_BM_biogeo[,p]/length(trees)

}

#save(Rsq_PCos_BM_biogeo, count.p.value_PCos_BM_biogeo, ratio_p.value_PCos_BM_biogeo, file = "./output/PCos_BM_biogeo.RData")

#load("./output/PCos_BM_biogeo.RData")

###Test PCos versus body mass+phylogeny

phylo_structure <- NULL

pgls.fit_PCos_BM_phylo = list()

for (m in 1:length(trees)) {

phylo_structure <- diag(vcv.phylo(time.trees.1branch_drop[[m]]))

pgls.fit_PCos_BM_phylo[[m]] <- procD.pgls(pcoa_result_vectors_prun[,1:3]~mass_data+phylo_structure,

phy = time.trees.drop[[m]],

data = dataset,

iter = 999,

SS.type = "II")

}

Rsq_PCos_BM_phylo <- matrix(ncol = dim(pgls.fit_PCos_BM_phylo[[1]]$aov.table)[1]-2,

nrow = length(trees))

colnames(Rsq_PCos_BM_phylo) <- rownames(pgls.fit_PCos_BM_phylo[[1]]$aov.table)[1:ncol(Rsq_PCos_BM_phylo)]

for (p in 1:ncol(Rsq_PCos_BM_phylo)) {

for (n in 1:length(trees)) {

Rsq_PCos_BM_phylo[n,p] <- pgls.fit_PCos_BM_phylo[[n]]$aov.table$Rsq[p]

}

}

p.value_PCos_BM_phylo <- matrix(ncol = dim(pgls.fit_PCos_BM_phylo[[1]]$aov.table)[1]-2,

nrow = length(trees))

count.p.value_PCos_BM_phylo <- matrix(ncol = dim(pgls.fit_PCos_BM_phylo[[1]]$aov.table)[1]-2,

nrow = 1)

ratio_p.value_PCos_BM_phylo <- matrix(ncol = dim(pgls.fit_PCos_BM_phylo[[1]]$aov.table)[1]-2,

nrow = 1)

colnames(ratio_p.value_PCos_BM_phylo) <- rownames(pgls.fit_PCos_BM_phylo[[1]]$aov.table)[1:ncol(Rsq_PCos_BM_phylo)]

for (p in 1:ncol(Rsq_PCos_BM_phylo)) {

for (o in 1:length(trees)) {

p.value_PCos_BM_phylo[o,p] <- pgls.fit_PCos_BM_phylo[[o]]$aov.table$`Pr(>F)`[p]<0.05

}

count.p.value_PCos_BM_phylo[,p] <- sum(p.value_PCos_BM_phylo[,p], na.rm=TRUE)

ratio_p.value_PCos_BM_phylo[,p] <- count.p.value_PCos_BM_phylo[,p]/length(trees)

}

#save(Rsq_PCos_BM_phylo, count.p.value_PCos_BM_phylo, ratio_p.value_PCos_BM_phylo, file = "./output/PCos_BM_phylo.RData")

#load("./output/PCos_BM_phylo.RData")

###Test PCos versus body mass+ratio+biogeography

pgls.fit_PCos_BM_ratio_biog = list()

for (m in 1:length(trees)) {

pgls.fit_PCos_BM_ratio_biog[[m]] <- procD.pgls(pcoa_result_vectors_prun[,1:3]~circum.ratio_data+mass_data+biogeo_data,

phy = time.trees.drop[[m]],

data = dataset,

iter = 999,

SS.type = "II")

}

Rsq_PCos_BM_ratio_biog <- matrix(ncol = dim(pgls.fit_PCos_BM_ratio_biog[[1]]$aov.table)[1]-2,

nrow = length(trees))

colnames(Rsq_PCos_BM_ratio_biog) <- rownames(pgls.fit_PCos_BM_ratio_biog[[1]]$aov.table)[1:ncol(Rsq_PCos_BM_ratio_biog)]

for (p in 1:ncol(Rsq_PCos_BM_ratio_biog)) {

for (n in 1:length(trees)) {

Rsq_PCos_BM_ratio_biog[n,p] <- pgls.fit_PCos_BM_ratio_biog[[n]]$aov.table$Rsq[p]

}

}

p.value_PCos_BM_ratio_biog <- matrix(ncol = dim(pgls.fit_PCos_BM_ratio_biog[[1]]$aov.table)[1]-2,

nrow = length(trees))

count.p.value_PCos_BM_ratio_biog <- matrix(ncol = dim(pgls.fit_PCos_BM_ratio_biog[[1]]$aov.table)[1]-2,

nrow = 1)

ratio_p.value_PCos_BM_ratio_biog <- matrix(ncol = dim(pgls.fit_PCos_BM_ratio_biog[[1]]$aov.table)[1]-2,

nrow = 1)

colnames(ratio_p.value_PCos_BM_ratio_biog) <- rownames(pgls.fit_PCos_BM_ratio_biog[[1]]$aov.table)[1:ncol(Rsq_PCos_BM_ratio_biog)]

for (p in 1:ncol(Rsq_PCos_BM_ratio_biog)) {

for (o in 1:length(trees)) {

p.value_PCos_BM_ratio_biog[o,p] <- pgls.fit_PCos_BM_ratio_biog[[o]]$aov.table$`Pr(>F)`[p]<0.05

}

count.p.value_PCos_BM_ratio_biog[,p] <- sum(p.value_PCos_BM_ratio_biog[,p], na.rm=TRUE)

ratio_p.value_PCos_BM_ratio_biog[,p] <- count.p.value_PCos_BM_ratio_biog[,p]/length(trees)

}

#save(Rsq_PCos_BM_ratio_biog, count.p.value_PCos_BM_ratio_biog, ratio_p.value_PCos_BM_ratio_biog, file = "./output/PCos_BM_ratio_biog.RData")

#load("./output/PCos_BM_ratio_biog.RData")

###Test PCos versus body mass+ratio+phylogeny

phylo_structure <- NULL

pgls.fit_PCos_BM_ratio_phy = list()

for (m in 1:length(trees)) {

phylo_structure <- diag(vcv.phylo(time.trees.1branch_drop[[m]]))

pgls.fit_PCos_BM_ratio_phy[[m]] <- procD.pgls(pcoa_result_vectors_prun[,1:3]~circum.ratio_data+mass_data+phylo_structure,

phy = time.trees.drop[[m]],

data = dataset,

iter = 999,

SS.type = "II")

}

Rsq_PCos_BM_ratio_phy <- matrix(ncol = dim(pgls.fit_PCos_BM_ratio_phy[[1]]$aov.table)[1]-2,

nrow = length(trees))

colnames(Rsq_PCos_BM_ratio_phy) <- rownames(pgls.fit_PCos_BM_ratio_phy[[1]]$aov.table)[1:ncol(Rsq_PCos_BM_ratio_phy)]

for (p in 1:ncol(Rsq_PCos_BM_ratio_phy)) {

for (n in 1:length(trees)) {

Rsq_PCos_BM_ratio_phy[n,p] <- pgls.fit_PCos_BM_ratio_phy[[n]]$aov.table$Rsq[p]

}

}

p.value_PCos_BM_ratio_phy <- matrix(ncol = dim(pgls.fit_PCos_BM_ratio_phy[[1]]$aov.table)[1]-2,

nrow = length(trees))

count.p.value_PCos_BM_ratio_phy <- matrix(ncol = dim(pgls.fit_PCos_BM_ratio_phy[[1]]$aov.table)[1]-2,

nrow = 1)

ratio_p.value_PCos_BM_ratio_phy <- matrix(ncol = dim(pgls.fit_PCos_BM_ratio_phy[[1]]$aov.table)[1]-2,

nrow = 1)

colnames(ratio_p.value_PCos_BM_ratio_phy) <- rownames(pgls.fit_PCos_BM_ratio_phy[[1]]$aov.table)[1:ncol(Rsq_PCos_BM_ratio_phy)]

for (p in 1:ncol(Rsq_PCos_BM_ratio_phy)) {

for (o in 1:length(trees)) {

p.value_PCos_BM_ratio_phy[o,p] <- pgls.fit_PCos_BM_ratio_phy[[o]]$aov.table$`Pr(>F)`[p]<0.05

}

count.p.value_PCos_BM_ratio_phy[,p] <- sum(p.value_PCos_BM_ratio_phy[,p], na.rm=TRUE)

ratio_p.value_PCos_BM_ratio_phy[,p] <- count.p.value_PCos_BM_ratio_phy[,p]/length(trees)

}

#save(Rsq_PCos_BM_ratio_phy, count.p.value_PCos_BM_ratio_phy, ratio_p.value_PCos_BM_ratio_phy, file = "./output/PCos_BM_ratio_phy.RData")

#load("./output/PCos_BM_ratio_phy.RData")

###Test PCos versus biogeography+phylogeny

phylo_structure <- NULL

pgls.fit_PCos_biogeo_phy = list()

for (m in 1:length(trees)) {

phylo_structure <- diag(vcv.phylo(time.trees.1branch_drop[[m]]))

pgls.fit_PCos_biogeo_phy[[m]] <- procD.pgls(pcoa_result_vectors_prun[,1:3]~biogeo_data+phylo_structure,

phy = time.trees.drop[[m]],

data = dataset,

iter = 999,

SS.type = "II")

}

Rsq_PCos_biogeo_phy <- matrix(ncol = dim(pgls.fit_PCos_biogeo_phy[[1]]$aov.table)[1]-2,

nrow = length(trees))

colnames(Rsq_PCos_biogeo_phy) <- rownames(pgls.fit_PCos_biogeo_phy[[1]]$aov.table)[1:ncol(Rsq_PCos_biogeo_phy)]

for (p in 1:ncol(Rsq_PCos_biogeo_phy)) {

for (n in 1:length(trees)) {

Rsq_PCos_biogeo_phy[n,p] <- pgls.fit_PCos_biogeo_phy[[n]]$aov.table$Rsq[p]

}

}

p.value_PCos_biogeo_phy <- matrix(ncol = dim(pgls.fit_PCos_biogeo_phy[[1]]$aov.table)[1]-2,

nrow = length(trees))

count.p.value_PCos_biogeo_phy <- matrix(ncol = dim(pgls.fit_PCos_biogeo_phy[[1]]$aov.table)[1]-2,

nrow = 1)

ratio_p.value_PCos_biogeo_phy <- matrix(ncol = dim(pgls.fit_PCos_biogeo_phy[[1]]$aov.table)[1]-2,

nrow = 1)

colnames(ratio_p.value_PCos_biogeo_phy) <- rownames(pgls.fit_PCos_biogeo_phy[[1]]$aov.table)[1:ncol(Rsq_PCos_biogeo_phy)]

for (p in 1:ncol(Rsq_PCos_biogeo_phy)) {

for (o in 1:length(trees)) {

p.value_PCos_biogeo_phy[o,p] <- pgls.fit_PCos_biogeo_phy[[o]]$aov.table$`Pr(>F)`[p]<0.05

}

count.p.value_PCos_biogeo_phy[,p] <- sum(p.value_PCos_biogeo_phy[,p], na.rm=TRUE)

ratio_p.value_PCos_biogeo_phy[,p] <- count.p.value_PCos_biogeo_phy[,p]/length(trees)

}

#save(Rsq_PCos_biogeo_phy, count.p.value_PCos_biogeo_phy, ratio_p.value_PCos_biogeo_phy, file = "./output/PCos_biogeo_phy.RData")

#load("./output/PCos_biogeo_phy.RData")

############

Rsqs_PCos <- data.frame("FL" = Rsq_PCos_FL,

"BM" = Rsq_PCos_BM,

"ratio" = Rsq_PCos_ratio,

"biog" = Rsq_PCos_BIOGEO,

"phy" = Rsq_PCos_phylo,

"FL+ratio" = Rsq_PCos_FL_ratio,

"FL+biog" = Rsq_PCos_FL_BIOGEO,

"FL+phy" = Rsq_PCos_FL_phylo,

"FL+biog+phy" = Rsq_PCos_FL_BIOGEO_phylo,

"BM+ratio" = Rsq_PCos_BM_ratio,

"BM+biog" = Rsq_PCos_BM_biogeo,

"BM+phy" = Rsq_PCos_BM_phylo,

"BM+ratio+biog" = Rsq_PCos_BM_ratio_biog,

"BM+ratio+phy" = Rsq_PCos_BM_ratio_phy,

"biog+phy" = Rsq_PCos_biogeo_phy)

p.values_PCos <- data.frame("FL" = ratio_p.value_PCos_FL,

"BM" = ratio_p.value_PCos_BM,

"ratio" = ratio_p.value_PCos_ratio,

"biog" = ratio_p.value_PCos_BIOGEO,

"phy" = ratio_p.value_PCos_phylo,

"FL+ratio" = ratio_p.value_PCos_FL_ratio,

"FL+biog" = ratio_p.value_PCos_FL_BIOGEO,

"FL+phy" = ratio_p.value_PCos_FL_phylo,

"FL+biog+phy" = ratio_p.value_PCos_FL_BIOGEO_phylo,

"BM+ratio" = ratio_p.value_PCos_BM_ratio,

"BM+biog" = ratio_p.value_PCos_BM_biogeo,

"BM+phy" = ratio_p.value_PCos_BM_phylo,

"BM+ratio+biog" = ratio_p.value_PCos_BM_ratio_biog,

"BM+ratio+phy" = ratio_p.value_PCos_BM_ratio_phy,

"biog+phy" = ratio_p.value_PCos_biogeo_phy)

#pdf(file = "./graphics/Model_test_PCos.pdf", width = 21, height = 10)

boxplot(Rsqs_PCos, ylab = "Rsq",

xlab = "Models PCos",

las = 2)

#dev.off()

###Generate model data for femoral length, body mass, locomotion, and area

#We will call "2" this new data set without ratio

model_data2 <- data[,c(4,5,7,8)]

rownames(model_data2) <- data$Taxon

model_data_red2 <- na.omit(model_data2)

model_data_red2 <- model_data_red2[setdiff(rownames(model_data_red2),trim_dist_matrix$removed_taxa),]

length_data2 <- log(model_data_red2$FL)

names(length_data2) <- rownames(model_data_red2)

mass_data2 <- log(model_data_red2$Mass)

names(mass_data2) <- rownames(model_data_red2)

biogeo_data2 <- model_data_red2$Area

names(biogeo_data2) <- rownames(model_data_red2)

locomotion_data2 <- model_data_red2$Locomotion

names(locomotion_data2) <- rownames(model_data_red2)

pcoa_result_vectors_prun2 <- pcoa_result$vectors.cor[intersect(names(pcoa_result$vectors.cor[,1]),rownames(model_data_red2)),]

setdiff(names(length_data2),names(pcoa_result_vectors_prun2[,1]))

dataset2 <- data.frame("PCo1" = pcoa_result_vectors_prun2[,1],

"FL" = length_data2,

"BM" = mass_data2,

"Area" = biogeo_data2,

"Locomotion" = locomotion_data2)

#Prune non-sauropodomorph taxa

time.trees.drop2 <- list()

for (j in 1:length(trees)) {

time.trees.drop2[[j]] <- drop.tip(phy = time.trees[[j]],

tip = c("Euparkeria","Crurotarsi","Marasuchus","Ornithischia","Agnosphitys","Silesaurus","Neotheropoda","Staurikosaurus","Chindesaurus","Herrerasaurus","Guaibasaurus","Neosauropoda"))

}

#Prune trimmed taxa before the ordination

for (k in 1:length(trees)) {

time.trees.drop2[[k]] <- drop.tip(phy = time.trees.drop2[[k]],

tip = trim_dist_matrix$removed_taxa)

}

NA_taxa2 <- setdiff(time.trees.drop2[[1]]$tip.label,rownames(model_data_red2))

for (l in 1:length(trees)) {

time.trees.drop2[[l]] <- drop.tip(phy = time.trees.drop2[[l]],

tip = NA_taxa2)

}

NA_taxa2_1my <- setdiff(time.trees.1branch[[1]]$tip.label,rownames(model_data_red2))

time.trees.1branch_drop2 <- NULL

for (l in 1:length(trees)) {

time.trees.1branch_drop2[[l]] <- drop.tip(phy = time.trees.1branch[[l]],

tip = NA_taxa2_1my)

}

for (m in 1:length(trees)) {

time.trees.1branch_drop2[[m]]$edge.length <- rep(1,length(time.trees.1branch_drop2[[m]]$edge.length))

}

###Test 2PCos versus femoral length

pgls.fit_2PCos_FL = list()

for (m in 1:length(trees)) {

pgls.fit_2PCos_FL[[m]] <- procD.pgls(pcoa_result_vectors_prun2[,1:3]~length_data2,

phy = time.trees.drop2[[m]],

data = dataset2,

iter = 999,

SS.type = "II")

}

Rsq_2PCos_FL <- matrix(ncol = dim(pgls.fit_2PCos_FL[[1]]$aov.table)[1]-2,

nrow = length(trees))

colnames(Rsq_2PCos_FL) <- rownames(pgls.fit_2PCos_FL[[1]]$aov.table)[1:ncol(Rsq_2PCos_FL)]

for (p in 1:ncol(Rsq_2PCos_FL)) {

for (n in 1:length(trees)) {

Rsq_2PCos_FL[n,p] <- pgls.fit_2PCos_FL[[n]]$aov.table$Rsq[p]

}

}

p.value_2PCos_FL <- matrix(ncol = dim(pgls.fit_2PCos_FL[[1]]$aov.table)[1]-2,

nrow = length(trees))

count.p.value_2PCos_FL <- matrix(ncol = dim(pgls.fit_2PCos_FL[[1]]$aov.table)[1]-2,

nrow = 1)

ratio_p.value_2PCos_FL <- matrix(ncol = dim(pgls.fit_2PCos_FL[[1]]$aov.table)[1]-2,

nrow = 1)

colnames(ratio_p.value_2PCos_FL) <- rownames(pgls.fit_2PCos_FL[[1]]$aov.table)[1:ncol(Rsq_2PCos_FL)]

for (p in 1:ncol(Rsq_2PCos_FL)) {

for (o in 1:length(trees)) {

p.value_2PCos_FL[o,p] <- pgls.fit_2PCos_FL[[o]]$aov.table$`Pr(>F)`[p]<0.05

}

count.p.value_2PCos_FL[,p] <- sum(p.value_2PCos_FL[,p], na.rm=TRUE)

ratio_p.value_2PCos_FL[,p] <- count.p.value_2PCos_FL[,p]/length(trees)

}

#save(Rsq_2PCos_FL, count.p.value_2PCos_FL, ratio_p.value_2PCos_FL, file = "./output/2PCos_FL.RData")

#load("./output/2PCos_FL.RData")

###Test 2PCos versus body mass

pgls.fit_2PCos_BM = list()

for (m in 1:length(trees)) {

pgls.fit_2PCos_BM[[m]] <- procD.pgls(pcoa_result_vectors_prun2[,1:3]~mass_data2,

phy = time.trees.drop2[[m]],

data = dataset2,

iter = 999,

SS.type = "II")

}

Rsq_2PCos_BM <- matrix(ncol = dim(pgls.fit_2PCos_BM[[1]]$aov.table)[1]-2,

nrow = length(trees))

colnames(Rsq_2PCos_BM) <- rownames(pgls.fit_2PCos_BM[[1]]$aov.table)[1:ncol(Rsq_2PCos_BM)]

for (p in 1:ncol(Rsq_2PCos_BM)) {

for (n in 1:length(trees)) {

Rsq_2PCos_BM[n,p] <- pgls.fit_2PCos_BM[[n]]$aov.table$Rsq[p]

}

}

p.value_2PCos_BM <- matrix(ncol = dim(pgls.fit_2PCos_BM[[1]]$aov.table)[1]-2,

nrow = length(trees))

count.p.value_2PCos_BM <- matrix(ncol = dim(pgls.fit_2PCos_BM[[1]]$aov.table)[1]-2,

nrow = 1)

ratio_p.value_2PCos_BM <- matrix(ncol = dim(pgls.fit_2PCos_BM[[1]]$aov.table)[1]-2,

nrow = 1)

colnames(ratio_p.value_2PCos_BM) <- rownames(pgls.fit_2PCos_BM[[1]]$aov.table)[1:ncol(Rsq_2PCos_BM)]

for (p in 1:ncol(Rsq_2PCos_BM)) {

for (o in 1:length(trees)) {

p.value_2PCos_BM[o,p] <- pgls.fit_2PCos_BM[[o]]$aov.table$`Pr(>F)`[p]<0.05

}

count.p.value_2PCos_BM[,p] <- sum(p.value_2PCos_BM[,p], na.rm=TRUE)

ratio_p.value_2PCos_BM[,p] <- count.p.value_2PCos_BM[,p]/length(trees)

}

#save(Rsq_2PCos_BM, count.p.value_2PCos_BM, ratio_p.value_2PCos_BM, file = "./output/2PCos_BM.RData")

#load("./output/2PCos_BM.RData")

###Test 2PCos versus biogeographic data

pgls.fit_2PCos_BIOGEO = list()

for (m in 1:length(trees)) {

pgls.fit_2PCos_BIOGEO[[m]] <- procD.pgls(pcoa_result_vectors_prun2[,1:3]~biogeo_data2,

phy = time.trees.drop2[[m]],

data = dataset2,

iter = 999,

SS.type = "II")

}

Rsq_2PCos_BIOGEO <- matrix(ncol = dim(pgls.fit_2PCos_BIOGEO[[1]]$aov.table)[1]-2,

nrow = length(trees))

colnames(Rsq_2PCos_BIOGEO) <- rownames(pgls.fit_2PCos_BIOGEO[[1]]$aov.table)[1:ncol(Rsq_2PCos_BIOGEO)]

for (p in 1:ncol(Rsq_2PCos_BIOGEO)) {

for (n in 1:length(trees)) {

Rsq_2PCos_BIOGEO[n,p] <- pgls.fit_2PCos_BIOGEO[[n]]$aov.table$Rsq[p]

}

}

p.value_2PCos_BIOGEO <- matrix(ncol = dim(pgls.fit_2PCos_BIOGEO[[1]]$aov.table)[1]-2,

nrow = length(trees))

count.p.value_2PCos_BIOGEO <- matrix(ncol = dim(pgls.fit_2PCos_BIOGEO[[1]]$aov.table)[1]-2,

nrow = 1)

ratio_p.value_2PCos_BIOGEO <- matrix(ncol = dim(pgls.fit_2PCos_BIOGEO[[1]]$aov.table)[1]-2,

nrow = 1)

colnames(ratio_p.value_2PCos_BIOGEO) <- rownames(pgls.fit_2PCos_BIOGEO[[1]]$aov.table)[1:ncol(Rsq_2PCos_BIOGEO)]

for (p in 1:ncol(Rsq_2PCos_BIOGEO)) {

for (o in 1:length(trees)) {

p.value_2PCos_BIOGEO[o,p] <- pgls.fit_2PCos_BIOGEO[[o]]$aov.table$`Pr(>F)`[p]<0.05

}

count.p.value_2PCos_BIOGEO[,p] <- sum(p.value_2PCos_BIOGEO[,p], na.rm=TRUE)

ratio_p.value_2PCos_BIOGEO[,p] <- count.p.value_2PCos_BIOGEO[,p]/length(trees)

}

#save(Rsq_2PCos_BIOGEO, count.p.value_2PCos_BIOGEO, ratio_p.value_2PCos_BIOGEO, file = "./output/2PCos_BIOGEO.RData")

#load("./output/2PCos_BIOGEO.RData")

###Test 2PCos versus phylogeny

phylo_structure2 <- NULL

pgls.fit_2PCos_phylo = list()

for (m in 1:length(trees)) {

phylo_structure2 <- diag(vcv.phylo(time.trees.1branch_drop2[[m]]))

pgls.fit_2PCos_phylo[[m]] <- procD.pgls(pcoa_result_vectors_prun2[,1:3]~phylo_structure2,

phy = time.trees.drop2[[m]],

data = dataset2,

iter = 999,

SS.type = "II")

}

Rsq_2PCos_phylo <- matrix(ncol = dim(pgls.fit_2PCos_phylo[[1]]$aov.table)[1]-2,

nrow = length(trees))

colnames(Rsq_2PCos_phylo) <- rownames(pgls.fit_2PCos_phylo[[1]]$aov.table)[1:ncol(Rsq_2PCos_phylo)]

for (p in 1:ncol(Rsq_2PCos_phylo)) {

for (n in 1:length(trees)) {

Rsq_2PCos_phylo[n,p] <- pgls.fit_2PCos_phylo[[n]]$aov.table$Rsq[p]

}

}

p.value_2PCos_phylo <- matrix(ncol = dim(pgls.fit_2PCos_phylo[[1]]$aov.table)[1]-2,

nrow = length(trees))

count.p.value_2PCos_phylo <- matrix(ncol = dim(pgls.fit_2PCos_phylo[[1]]$aov.table)[1]-2,

nrow = 1)

ratio_p.value_2PCos_phylo <- matrix(ncol = dim(pgls.fit_2PCos_phylo[[1]]$aov.table)[1]-2,

nrow = 1)

colnames(ratio_p.value_2PCos_phylo) <- rownames(pgls.fit_2PCos_phylo[[1]]$aov.table)[1:ncol(Rsq_2PCos_phylo)]

for (p in 1:ncol(Rsq_2PCos_phylo)) {

for (o in 1:length(trees)) {

p.value_2PCos_phylo[o,p] <- pgls.fit_2PCos_phylo[[o]]$aov.table$`Pr(>F)`[p]<0.05

}

count.p.value_2PCos_phylo[,p] <- sum(p.value_2PCos_phylo[,p], na.rm=TRUE)

ratio_p.value_2PCos_phylo[,p] <- count.p.value_2PCos_phylo[,p]/length(trees)

}

#save(Rsq_2PCos_phylo, count.p.value_2PCos_phylo, ratio_p.value_2PCos_phylo, file = "./output/2PCos_phylo.RData")

#load("./output/2PCos_phylo.RData")

###Test 2PCos versus locomotion style

pgls.fit_2PCos_locom = list()

for (m in 1:length(trees)) {

pgls.fit_2PCos_locom[[m]] <- procD.pgls(pcoa_result_vectors_prun2[,1:3]~locomotion_data2,

phy = time.trees.drop2[[m]],

data = dataset2,

iter = 999,

SS.type = "II")

}

Rsq_2PCos_locom <- matrix(ncol = dim(pgls.fit_2PCos_locom[[1]]$aov.table)[1]-2,

nrow = length(trees))

colnames(Rsq_2PCos_locom) <- rownames(pgls.fit_2PCos_locom[[1]]$aov.table)[1:ncol(Rsq_2PCos_locom)]

for (p in 1:ncol(Rsq_2PCos_locom)) {

for (n in 1:length(trees)) {

Rsq_2PCos_locom[n,p] <- pgls.fit_2PCos_locom[[n]]$aov.table$Rsq[p]

}

}

p.value_2PCos_locom <- matrix(ncol = dim(pgls.fit_2PCos_locom[[1]]$aov.table)[1]-2,

nrow = length(trees))

count.p.value_2PCos_locom <- matrix(ncol = dim(pgls.fit_2PCos_locom[[1]]$aov.table)[1]-2,

nrow = 1)

ratio_p.value_2PCos_locom <- matrix(ncol = dim(pgls.fit_2PCos_locom[[1]]$aov.table)[1]-2,

nrow = 1)

colnames(ratio_p.value_2PCos_locom) <- rownames(pgls.fit_2PCos_locom[[1]]$aov.table)[1:ncol(Rsq_2PCos_locom)]

for (p in 1:ncol(Rsq_2PCos_locom)) {

for (o in 1:length(trees)) {

p.value_2PCos_locom[o,p] <- pgls.fit_2PCos_locom[[o]]$aov.table$`Pr(>F)`[p]<0.05

}

count.p.value_2PCos_locom[,p] <- sum(p.value_2PCos_locom[,p], na.rm=TRUE)

ratio_p.value_2PCos_locom[,p] <- count.p.value_2PCos_locom[,p]/length(trees)

}

#save(Rsq_2PCos_locom, count.p.value_2PCos_locom, ratio_p.value_2PCos_locom, file = "./output/2PCos_locom.RData")

#load("./output/2PCos_locom.RData")

###Test 2PCos versus femoral length+biogeographic data

pgls.fit_2PCos_FL_BIOGEO = list()

for (m in 1:length(trees)) {

pgls.fit_2PCos_FL_BIOGEO[[m]] <- procD.pgls(pcoa_result_vectors_prun2[,1:3]~length_data2+biogeo_data2,

phy = time.trees.drop2[[m]],

data = dataset2,

iter = 999,

SS.type = "II")

}

Rsq_2PCos_FL_BIOGEO <- matrix(ncol = dim(pgls.fit_2PCos_FL_BIOGEO[[1]]$aov.table)[1]-2,

nrow = length(trees))

colnames(Rsq_2PCos_FL_BIOGEO) <- rownames(pgls.fit_2PCos_FL_BIOGEO[[1]]$aov.table)[1:ncol(Rsq_2PCos_FL_BIOGEO)]

for (p in 1:ncol(Rsq_2PCos_FL_BIOGEO)) {

for (n in 1:length(trees)) {

Rsq_2PCos_FL_BIOGEO[n,p] <- pgls.fit_2PCos_FL_BIOGEO[[n]]$aov.table$Rsq[p]

}

}

p.value_2PCos_FL_BIOGEO <- matrix(ncol = dim(pgls.fit_2PCos_FL_BIOGEO[[1]]$aov.table)[1]-2,

nrow = length(trees))

count.p.value_2PCos_FL_BIOGEO <- matrix(ncol = dim(pgls.fit_2PCos_FL_BIOGEO[[1]]$aov.table)[1]-2,

nrow = 1)

ratio_p.value_2PCos_FL_BIOGEO <- matrix(ncol = dim(pgls.fit_2PCos_FL_BIOGEO[[1]]$aov.table)[1]-2,

nrow = 1)

colnames(ratio_p.value_2PCos_FL_BIOGEO) <- rownames(pgls.fit_2PCos_FL_BIOGEO[[1]]$aov.table)[1:ncol(Rsq_2PCos_FL_BIOGEO)]

for (p in 1:ncol(Rsq_2PCos_FL_BIOGEO)) {

for (o in 1:length(trees)) {

p.value_2PCos_FL_BIOGEO[o,p] <- pgls.fit_2PCos_FL_BIOGEO[[o]]$aov.table$`Pr(>F)`[p]<0.05

}

count.p.value_2PCos_FL_BIOGEO[,p] <- sum(p.value_2PCos_FL_BIOGEO[,p], na.rm=TRUE)

ratio_p.value_2PCos_FL_BIOGEO[,p] <- count.p.value_2PCos_FL_BIOGEO[,p]/length(trees)

}

#save(Rsq_2PCos_FL_BIOGEO, count.p.value_2PCos_FL_BIOGEO, ratio_p.value_2PCos_FL_BIOGEO, file = "./output/2PCos_FL_BIOGEO.RData")

#load("./output/2PCos_FL_BIOGEO.RData")

###Test 2PCos versus femoral length+phylogeny

phylo_structure2 <- NULL

pgls.fit_2PCos_FL_phylo = list()

for (m in 1:length(trees)) {

phylo_structure2 <- diag(vcv.phylo(time.trees.1branch_drop2[[m]]))

pgls.fit_2PCos_FL_phylo[[m]] <- procD.pgls(pcoa_result_vectors_prun2[,1:3]~length_data2+phylo_structure2,

phy = time.trees.drop2[[m]],

data = dataset2,

iter = 999,

SS.type = "II")

}

Rsq_2PCos_FL_phylo <- matrix(ncol = dim(pgls.fit_2PCos_FL_phylo[[1]]$aov.table)[1]-2,

nrow = length(trees))

colnames(Rsq_2PCos_FL_phylo) <- rownames(pgls.fit_2PCos_FL_phylo[[1]]$aov.table)[1:ncol(Rsq_2PCos_FL_phylo)]

for (p in 1:ncol(Rsq_2PCos_FL_phylo)) {

for (n in 1:length(trees)) {

Rsq_2PCos_FL_phylo[n,p] <- pgls.fit_2PCos_FL_phylo[[n]]$aov.table$Rsq[p]

}

}

p.value_2PCos_FL_phylo <- matrix(ncol = dim(pgls.fit_2PCos_FL_phylo[[1]]$aov.table)[1]-2,

nrow = length(trees))

count.p.value_2PCos_FL_phylo <- matrix(ncol = dim(pgls.fit_2PCos_FL_phylo[[1]]$aov.table)[1]-2,

nrow = 1)

ratio_p.value_2PCos_FL_phylo <- matrix(ncol = dim(pgls.fit_2PCos_FL_phylo[[1]]$aov.table)[1]-2,

nrow = 1)

colnames(ratio_p.value_2PCos_FL_phylo) <- rownames(pgls.fit_2PCos_FL_phylo[[1]]$aov.table)[1:ncol(Rsq_2PCos_FL_phylo)]

for (p in 1:ncol(Rsq_2PCos_FL_phylo)) {

for (o in 1:length(trees)) {

p.value_2PCos_FL_phylo[o,p] <- pgls.fit_2PCos_FL_phylo[[o]]$aov.table$`Pr(>F)`[p]<0.05

}

count.p.value_2PCos_FL_phylo[,p] <- sum(p.value_2PCos_FL_phylo[,p], na.rm=TRUE)

ratio_p.value_2PCos_FL_phylo[,p] <- count.p.value_2PCos_FL_phylo[,p]/length(trees)

}

#save(Rsq_2PCos_FL_phylo, count.p.value_2PCos_FL_phylo, ratio_p.value_2PCos_FL_phylo, file = "./output/2PCos_FL_phylo.RData")

#load("./output/2PCos_FL_phylo.RData")

###Test 2PCos versus femoral length+locomotion

pgls.fit_2PCos_FL_locom = list()

for (m in 1:length(trees)) {

pgls.fit_2PCos_FL_locom[[m]] <- procD.pgls(pcoa_result_vectors_prun2[,1:3]~length_data2+locomotion_data2,

phy = time.trees.drop2[[m]],

data = dataset2,

iter = 999,

SS.type = "II")

}

Rsq_2PCos_FL_locom <- matrix(ncol = dim(pgls.fit_2PCos_FL_locom[[1]]$aov.table)[1]-2,

nrow = length(trees))

colnames(Rsq_2PCos_FL_locom) <- rownames(pgls.fit_2PCos_FL_locom[[1]]$aov.table)[1:ncol(Rsq_2PCos_FL_locom)]

for (p in 1:ncol(Rsq_2PCos_FL_locom)) {

for (n in 1:length(trees)) {

Rsq_2PCos_FL_locom[n,p] <- pgls.fit_2PCos_FL_locom[[n]]$aov.table$Rsq[p]

}

}

p.value_2PCos_FL_locom <- matrix(ncol = dim(pgls.fit_2PCos_FL_locom[[1]]$aov.table)[1]-2,

nrow = length(trees))

count.p.value_2PCos_FL_locom <- matrix(ncol = dim(pgls.fit_2PCos_FL_locom[[1]]$aov.table)[1]-2,

nrow = 1)

ratio_p.value_2PCos_FL_locom <- matrix(ncol = dim(pgls.fit_2PCos_FL_locom[[1]]$aov.table)[1]-2,

nrow = 1)

colnames(ratio_p.value_2PCos_FL_locom) <- rownames(pgls.fit_2PCos_FL_locom[[1]]$aov.table)[1:ncol(Rsq_2PCos_FL_locom)]

for (p in 1:ncol(Rsq_2PCos_FL_locom)) {

for (o in 1:length(trees)) {

p.value_2PCos_FL_locom[o,p] <- pgls.fit_2PCos_FL_locom[[o]]$aov.table$`Pr(>F)`[p]<0.05

}

count.p.value_2PCos_FL_locom[,p] <- sum(p.value_2PCos_FL_locom[,p], na.rm=TRUE)

ratio_p.value_2PCos_FL_locom[,p] <- count.p.value_2PCos_FL_locom[,p]/length(trees)

}

#save(Rsq_2PCos_FL_locom, count.p.value_2PCos_FL_locom, ratio_p.value_2PCos_FL_locom, file = "./output/2PCos_FL_locom.RData")

#load("./output/2PCos_FL_locom.RData")

###Test 2PCos versus femoral length+biogeographic data+phylogeny

pgls.fit_2PCos_FL_BIOGEO_phylo = list()

phylo_structure2 <- NULL

for (m in 1:length(trees)) {

phylo_structure2 <- diag(vcv.phylo(time.trees.1branch_drop2[[m]]))

pgls.fit_2PCos_FL_BIOGEO_phylo[[m]] <- procD.pgls(pcoa_result_vectors_prun2[,1:3]~length_data2+biogeo_data2+phylo_structure2,

phy = time.trees.drop2[[m]],

data = dataset2,

iter = 999,

SS.type = "II")

}

Rsq_2PCos_FL_BIOGEO_phylo <- matrix(ncol = dim(pgls.fit_2PCos_FL_BIOGEO_phylo[[1]]$aov.table)[1]-2,

nrow = length(trees))

colnames(Rsq_2PCos_FL_BIOGEO_phylo) <- rownames(pgls.fit_2PCos_FL_BIOGEO_phylo[[1]]$aov.table)[1:ncol(Rsq_2PCos_FL_BIOGEO_phylo)]

for (p in 1:ncol(Rsq_2PCos_FL_BIOGEO_phylo)) {

for (n in 1:length(trees)) {

Rsq_2PCos_FL_BIOGEO_phylo[n,p] <- pgls.fit_2PCos_FL_BIOGEO_phylo[[n]]$aov.table$Rsq[p]

}

}

p.value_2PCos_FL_BIOGEO_phylo <- matrix(ncol = dim(pgls.fit_2PCos_FL_BIOGEO_phylo[[1]]$aov.table)[1]-2,

nrow = length(trees))

count.p.value_2PCos_FL_BIOGEO_phylo <- matrix(ncol = dim(pgls.fit_2PCos_FL_BIOGEO_phylo[[1]]$aov.table)[1]-2,

nrow = 1)

ratio_p.value_2PCos_FL_BIOGEO_phylo <- matrix(ncol = dim(pgls.fit_2PCos_FL_BIOGEO_phylo[[1]]$aov.table)[1]-2,

nrow = 1)

colnames(ratio_p.value_2PCos_FL_BIOGEO_phylo) <- rownames(pgls.fit_2PCos_FL_BIOGEO_phylo[[1]]$aov.table)[1:ncol(Rsq_2PCos_FL_BIOGEO_phylo)]

for (p in 1:ncol(Rsq_2PCos_FL_BIOGEO_phylo)) {

for (o in 1:length(trees)) {

p.value_2PCos_FL_BIOGEO_phylo[o,p] <- pgls.fit_2PCos_FL_BIOGEO_phylo[[o]]$aov.table$`Pr(>F)`[p]<0.05

}

count.p.value_2PCos_FL_BIOGEO_phylo[,p] <- sum(p.value_2PCos_FL_BIOGEO_phylo[,p], na.rm=TRUE)

ratio_p.value_2PCos_FL_BIOGEO_phylo[,p] <- count.p.value_2PCos_FL_BIOGEO_phylo[,p]/length(trees)

}

#save(Rsq_2PCos_FL_BIOGEO_phylo, count.p.value_2PCos_FL_BIOGEO_phylo, ratio_p.value_2PCos_FL_BIOGEO_phylo, file = "./output/2PCos_FL_BIOGEO_phylo.RData")

#load("./output/2PCos_FL_BIOGEO_phylo.RData")

###Test 2PCos versus femoral length+phylogeny+locomotion

pgls.fit_2PCos_FL_phylo_locom = list()

phylo_structure2 <- NULL

for (m in 1:length(trees)) {

phylo_structure2 <- diag(vcv.phylo(time.trees.1branch_drop2[[m]]))

pgls.fit_2PCos_FL_phylo_locom[[m]] <- procD.pgls(pcoa_result_vectors_prun2[,1:3]~length_data2+phylo_structure2+locomotion_data2,

phy = time.trees.drop2[[m]],

data = dataset2,

iter = 999,

SS.type = "II")

}

Rsq_2PCos_FL_phylo_locom <- matrix(ncol = dim(pgls.fit_2PCos_FL_phylo_locom[[1]]$aov.table)[1]-2,

nrow = length(trees))

colnames(Rsq_2PCos_FL_phylo_locom) <- rownames(pgls.fit_2PCos_FL_phylo_locom[[1]]$aov.table)[1:ncol(Rsq_2PCos_FL_phylo_locom)]

for (p in 1:ncol(Rsq_2PCos_FL_phylo_locom)) {

for (n in 1:length(trees)) {

Rsq_2PCos_FL_phylo_locom[n,p] <- pgls.fit_2PCos_FL_phylo_locom[[n]]$aov.table$Rsq[p]

}

}

p.value_2PCos_FL_phylo_locom <- matrix(ncol = dim(pgls.fit_2PCos_FL_phylo_locom[[1]]$aov.table)[1]-2,

nrow = length(trees))

count.p.value_2PCos_FL_phylo_locom <- matrix(ncol = dim(pgls.fit_2PCos_FL_phylo_locom[[1]]$aov.table)[1]-2,

nrow = 1)

ratio_p.value_2PCos_FL_phylo_locom <- matrix(ncol = dim(pgls.fit_2PCos_FL_phylo_locom[[1]]$aov.table)[1]-2,

nrow = 1)

colnames(ratio_p.value_2PCos_FL_phylo_locom) <- rownames(pgls.fit_2PCos_FL_phylo_locom[[1]]$aov.table)[1:ncol(Rsq_2PCos_FL_phylo_locom)]

for (p in 1:ncol(Rsq_2PCos_FL_phylo_locom)) {

for (o in 1:length(trees)) {

p.value_2PCos_FL_phylo_locom[o,p] <- pgls.fit_2PCos_FL_phylo_locom[[o]]$aov.table$`Pr(>F)`[p]<0.05

}

count.p.value_2PCos_FL_phylo_locom[,p] <- sum(p.value_2PCos_FL_phylo_locom[,p], na.rm=TRUE)

ratio_p.value_2PCos_FL_phylo_locom[,p] <- count.p.value_2PCos_FL_phylo_locom[,p]/length(trees)

}

#save(Rsq_2PCos_FL_phylo_locom, count.p.value_2PCos_FL_phylo_locom, ratio_p.value_2PCos_FL_phylo_locom, file = "./output/2PCos_FL_phylo_locom.RData")

#load("./output/2PCos_FL_phylo_locom.RData")

###Test 2PCos versus body mass+biogeography

pgls.fit_2PCos_BM_biogeo = list()

for (m in 1:length(trees)) {

pgls.fit_2PCos_BM_biogeo[[m]] <- procD.pgls(pcoa_result_vectors_prun2[,1:3]~mass_data2+biogeo_data2,

phy = time.trees.drop2[[m]],

data = dataset2,

iter = 999,

SS.type = "II")

}

Rsq_2PCos_BM_biogeo <- matrix(ncol = dim(pgls.fit_2PCos_BM_biogeo[[1]]$aov.table)[1]-2,

nrow = length(trees))

colnames(Rsq_2PCos_BM_biogeo) <- rownames(pgls.fit_2PCos_BM_biogeo[[1]]$aov.table)[1:ncol(Rsq_2PCos_BM_biogeo)]

for (p in 1:ncol(Rsq_2PCos_BM_biogeo)) {

for (n in 1:length(trees)) {

Rsq_2PCos_BM_biogeo[n,p] <- pgls.fit_2PCos_BM_biogeo[[n]]$aov.table$Rsq[p]

}

}

p.value_2PCos_BM_biogeo <- matrix(ncol = dim(pgls.fit_2PCos_BM_biogeo[[1]]$aov.table)[1]-2,

nrow = length(trees))

count.p.value_2PCos_BM_biogeo <- matrix(ncol = dim(pgls.fit_2PCos_BM_biogeo[[1]]$aov.table)[1]-2,

nrow = 1)

ratio_p.value_2PCos_BM_biogeo <- matrix(ncol = dim(pgls.fit_2PCos_BM_biogeo[[1]]$aov.table)[1]-2,

nrow = 1)

colnames(ratio_p.value_2PCos_BM_biogeo) <- rownames(pgls.fit_2PCos_BM_biogeo[[1]]$aov.table)[1:ncol(Rsq_2PCos_BM_biogeo)]

for (p in 1:ncol(Rsq_2PCos_BM_biogeo)) {

for (o in 1:length(trees)) {

p.value_2PCos_BM_biogeo[o,p] <- pgls.fit_2PCos_BM_biogeo[[o]]$aov.table$`Pr(>F)`[p]<0.05

}

count.p.value_2PCos_BM_biogeo[,p] <- sum(p.value_2PCos_BM_biogeo[,p], na.rm=TRUE)

ratio_p.value_2PCos_BM_biogeo[,p] <- count.p.value_2PCos_BM_biogeo[,p]/length(trees)

}

#save(Rsq_2PCos_BM_biogeo, count.p.value_2PCos_BM_biogeo, ratio_p.value_2PCos_BM_biogeo, file = "./output/2PCos_BM_biogeo.RData")

#load("./output/2PCos_BM_biogeo.RData")

###Test 2PCos versus body mass+phylogeny

phylo_structure2 <- NULL

pgls.fit_2PCos_BM_phylo = list()

for (m in 1:length(trees)) {

phylo_structure2 <- diag(vcv.phylo(time.trees.1branch_drop2[[m]]))

pgls.fit_2PCos_BM_phylo[[m]] <- procD.pgls(pcoa_result_vectors_prun2[,1:3]~mass_data2+phylo_structure2,

phy = time.trees.drop2[[m]],

data = dataset2,

iter = 999,

SS.type = "II")

}

Rsq_2PCos_BM_phylo <- matrix(ncol = dim(pgls.fit_2PCos_BM_phylo[[1]]$aov.table)[1]-2,

nrow = length(trees))

colnames(Rsq_2PCos_BM_phylo) <- rownames(pgls.fit_2PCos_BM_phylo[[1]]$aov.table)[1:ncol(Rsq_2PCos_BM_phylo)]

for (p in 1:ncol(Rsq_2PCos_BM_phylo)) {

for (n in 1:length(trees)) {

Rsq_2PCos_BM_phylo[n,p] <- pgls.fit_2PCos_BM_phylo[[n]]$aov.table$Rsq[p]

}

}

p.value_2PCos_BM_phylo <- matrix(ncol = dim(pgls.fit_2PCos_BM_phylo[[1]]$aov.table)[1]-2,

nrow = length(trees))

count.p.value_2PCos_BM_phylo <- matrix(ncol = dim(pgls.fit_2PCos_BM_phylo[[1]]$aov.table)[1]-2,

nrow = 1)

ratio_p.value_2PCos_BM_phylo <- matrix(ncol = dim(pgls.fit_2PCos_BM_phylo[[1]]$aov.table)[1]-2,

nrow = 1)

colnames(ratio_p.value_2PCos_BM_phylo) <- rownames(pgls.fit_2PCos_BM_phylo[[1]]$aov.table)[1:ncol(Rsq_2PCos_BM_phylo)]

for (p in 1:ncol(Rsq_2PCos_BM_phylo)) {

for (o in 1:length(trees)) {

p.value_2PCos_BM_phylo[o,p] <- pgls.fit_2PCos_BM_phylo[[o]]$aov.table$`Pr(>F)`[p]<0.05

}

count.p.value_2PCos_BM_phylo[,p] <- sum(p.value_2PCos_BM_phylo[,p], na.rm=TRUE)

ratio_p.value_2PCos_BM_phylo[,p] <- count.p.value_2PCos_BM_phylo[,p]/length(trees)

}

#save(Rsq_2PCos_BM_phylo, count.p.value_2PCos_BM_phylo, ratio_p.value_2PCos_BM_phylo, file = "./output/2PCos_BM_phylo.RData")

#load("./output/2PCos_BM_phylo.RData")

###Test 2PCos versus body mass+locomotion

pgls.fit_2PCos_BM_locom = list()

for (m in 1:length(trees)) {

pgls.fit_2PCos_BM_locom[[m]] <- procD.pgls(pcoa_result_vectors_prun2[,1:3]~mass_data2+locomotion_data2,

phy = time.trees.drop2[[m]],

data = dataset2,

iter = 999,

SS.type = "II")

}

Rsq_2PCos_BM_locom <- matrix(ncol = dim(pgls.fit_2PCos_BM_locom[[1]]$aov.table)[1]-2,

nrow = length(trees))

colnames(Rsq_2PCos_BM_locom) <- rownames(pgls.fit_2PCos_BM_locom[[1]]$aov.table)[1:ncol(Rsq_2PCos_BM_locom)]

for (p in 1:ncol(Rsq_2PCos_BM_locom)) {

for (n in 1:length(trees)) {

Rsq_2PCos_BM_locom[n,p] <- pgls.fit_2PCos_BM_locom[[n]]$aov.table$Rsq[p]

}

}

p.value_2PCos_BM_locom <- matrix(ncol = dim(pgls.fit_2PCos_BM_locom[[1]]$aov.table)[1]-2,

nrow = length(trees))

count.p.value_2PCos_BM_locom <- matrix(ncol = dim(pgls.fit_2PCos_BM_locom[[1]]$aov.table)[1]-2,

nrow = 1)

ratio_p.value_2PCos_BM_locom <- matrix(ncol = dim(pgls.fit_2PCos_BM_locom[[1]]$aov.table)[1]-2,

nrow = 1)

colnames(ratio_p.value_2PCos_BM_locom) <- rownames(pgls.fit_2PCos_BM_locom[[1]]$aov.table)[1:ncol(Rsq_2PCos_BM_locom)]

for (p in 1:ncol(Rsq_2PCos_BM_locom)) {

for (o in 1:length(trees)) {

p.value_2PCos_BM_locom[o,p] <- pgls.fit_2PCos_BM_locom[[o]]$aov.table$`Pr(>F)`[p]<0.05

}

count.p.value_2PCos_BM_locom[,p] <- sum(p.value_2PCos_BM_locom[,p], na.rm=TRUE)

ratio_p.value_2PCos_BM_locom[,p] <- count.p.value_2PCos_BM_locom[,p]/length(trees)

}

#save(Rsq_2PCos_BM_locom, count.p.value_2PCos_BM_locom, ratio_p.value_2PCos_BM_locom, file = "./output/2PCos_BM_locom.RData")

#load("./output/2PCos_BM_locom.RData")

###Test 2PCos versus biogeography+phylogeny

phylo_structure2 <- NULL

pgls.fit_2PCos_biogeo_phy = list()

for (m in 1:length(trees)) {

phylo_structure2 <- diag(vcv.phylo(time.trees.1branch_drop2[[m]]))

pgls.fit_2PCos_biogeo_phy[[m]] <- procD.pgls(pcoa_result_vectors_prun2[,1:3]~biogeo_data2+phylo_structure2,

phy = time.trees.drop2[[m]],

data = dataset2,

iter = 999,

SS.type = "II")

}

Rsq_2PCos_biogeo_phy <- matrix(ncol = dim(pgls.fit_2PCos_biogeo_phy[[1]]$aov.table)[1]-2,

nrow = length(trees))

colnames(Rsq_2PCos_biogeo_phy) <- rownames(pgls.fit_2PCos_biogeo_phy[[1]]$aov.table)[1:ncol(Rsq_2PCos_biogeo_phy)]

for (p in 1:ncol(Rsq_2PCos_biogeo_phy)) {

for (n in 1:length(trees)) {

Rsq_2PCos_biogeo_phy[n,p] <- pgls.fit_2PCos_biogeo_phy[[n]]$aov.table$Rsq[p]

}

}

p.value_2PCos_biogeo_phy <- matrix(ncol = dim(pgls.fit_2PCos_biogeo_phy[[1]]$aov.table)[1]-2,

nrow = length(trees))

count.p.value_2PCos_biogeo_phy <- matrix(ncol = dim(pgls.fit_2PCos_biogeo_phy[[1]]$aov.table)[1]-2,

nrow = 1)

ratio_p.value_2PCos_biogeo_phy <- matrix(ncol = dim(pgls.fit_2PCos_biogeo_phy[[1]]$aov.table)[1]-2,

nrow = 1)

colnames(ratio_p.value_2PCos_biogeo_phy) <- rownames(pgls.fit_2PCos_biogeo_phy[[1]]$aov.table)[1:ncol(Rsq_2PCos_biogeo_phy)]

for (p in 1:ncol(Rsq_2PCos_biogeo_phy)) {

for (o in 1:length(trees)) {

p.value_2PCos_biogeo_phy[o,p] <- pgls.fit_2PCos_biogeo_phy[[o]]$aov.table$`Pr(>F)`[p]<0.05

}

count.p.value_2PCos_biogeo_phy[,p] <- sum(p.value_2PCos_biogeo_phy[,p], na.rm=TRUE)

ratio_p.value_2PCos_biogeo_phy[,p] <- count.p.value_2PCos_biogeo_phy[,p]/length(trees)

}

#save(Rsq_2PCos_biogeo_phy, count.p.value_2PCos_biogeo_phy, ratio_p.value_2PCos_biogeo_phy, file = "./output/2PCos_biogeo_phy.RData")

#load("./output/2PCos_biogeo_phy.RData")

###Test 2PCos versus biogeography+locomotion

pgls.fit_2PCos_biogeo_locom = list()

for (m in 1:length(trees)) {

pgls.fit_2PCos_biogeo_locom[[m]] <- procD.pgls(pcoa_result_vectors_prun2[,1:3]~biogeo_data2+locomotion_data2,

phy = time.trees.drop2[[m]],

data = dataset2,

iter = 999,

SS.type = "II")

}

Rsq_2PCos_biogeo_locom <- matrix(ncol = dim(pgls.fit_2PCos_biogeo_locom[[1]]$aov.table)[1]-2,

nrow = length(trees))

colnames(Rsq_2PCos_biogeo_locom) <- rownames(pgls.fit_2PCos_biogeo_locom[[1]]$aov.table)[1:ncol(Rsq_2PCos_biogeo_locom)]

for (p in 1:ncol(Rsq_2PCos_biogeo_locom)) {

for (n in 1:length(trees)) {

Rsq_2PCos_biogeo_locom[n,p] <- pgls.fit_2PCos_biogeo_locom[[n]]$aov.table$Rsq[p]

}

}

p.value_2PCos_biogeo_locom <- matrix(ncol = dim(pgls.fit_2PCos_biogeo_locom[[1]]$aov.table)[1]-2,

nrow = length(trees))

count.p.value_2PCos_biogeo_locom <- matrix(ncol = dim(pgls.fit_2PCos_biogeo_locom[[1]]$aov.table)[1]-2,

nrow = 1)

ratio_p.value_2PCos_biogeo_locom <- matrix(ncol = dim(pgls.fit_2PCos_biogeo_locom[[1]]$aov.table)[1]-2,

nrow = 1)

colnames(ratio_p.value_2PCos_biogeo_locom) <- rownames(pgls.fit_2PCos_biogeo_locom[[1]]$aov.table)[1:ncol(Rsq_2PCos_biogeo_locom)]

for (p in 1:ncol(Rsq_2PCos_biogeo_locom)) {

for (o in 1:length(trees)) {

p.value_2PCos_biogeo_locom[o,p] <- pgls.fit_2PCos_biogeo_locom[[o]]$aov.table$`Pr(>F)`[p]<0.05

}

count.p.value_2PCos_biogeo_locom[,p] <- sum(p.value_2PCos_biogeo_locom[,p], na.rm=TRUE)

ratio_p.value_2PCos_biogeo_locom[,p] <- count.p.value_2PCos_biogeo_locom[,p]/length(trees)

}

#save(Rsq_2PCos_biogeo_locom, count.p.value_2PCos_biogeo_locom, ratio_p.value_2PCos_biogeo_locom, file = "./output/2PCos_biogeo_locom.RData")

#load("./output/2PCos_biogeo_locom.RData")

###Test 2PCos versus phylogeny+locomotion

phylo_structure2 <- NULL

pgls.fit_2PCos_phy_locom = list()

for (m in 1:length(trees)) {

phylo_structure2 <- diag(vcv.phylo(time.trees.1branch_drop2[[m]]))

pgls.fit_2PCos_phy_locom[[m]] <- procD.pgls(pcoa_result_vectors_prun2[,1:3]~phylo_structure2+locomotion_data2,

phy = time.trees.drop2[[m]],

data = dataset2,

iter = 999,

SS.type = "II")

}

Rsq_2PCos_phy_locom <- matrix(ncol = dim(pgls.fit_2PCos_phy_locom[[1]]$aov.table)[1]-2,

nrow = length(trees))

colnames(Rsq_2PCos_phy_locom) <- rownames(pgls.fit_2PCos_phy_locom[[1]]$aov.table)[1:ncol(Rsq_2PCos_phy_locom)]

for (p in 1:ncol(Rsq_2PCos_phy_locom)) {

for (n in 1:length(trees)) {

Rsq_2PCos_phy_locom[n,p] <- pgls.fit_2PCos_phy_locom[[n]]$aov.table$Rsq[p]

}

}

p.value_2PCos_phy_locom <- matrix(ncol = dim(pgls.fit_2PCos_phy_locom[[1]]$aov.table)[1]-2,

nrow = length(trees))

count.p.value_2PCos_phy_locom <- matrix(ncol = dim(pgls.fit_2PCos_phy_locom[[1]]$aov.table)[1]-2,

nrow = 1)

ratio_p.value_2PCos_phy_locom <- matrix(ncol = dim(pgls.fit_2PCos_phy_locom[[1]]$aov.table)[1]-2,

nrow = 1)

colnames(ratio_p.value_2PCos_phy_locom) <- rownames(pgls.fit_2PCos_phy_locom[[1]]$aov.table)[1:ncol(Rsq_2PCos_phy_locom)]

for (p in 1:ncol(Rsq_2PCos_phy_locom)) {

for (o in 1:length(trees)) {

p.value_2PCos_phy_locom[o,p] <- pgls.fit_2PCos_phy_locom[[o]]$aov.table$`Pr(>F)`[p]<0.05

}

count.p.value_2PCos_phy_locom[,p] <- sum(p.value_2PCos_phy_locom[,p], na.rm=TRUE)

ratio_p.value_2PCos_phy_locom[,p] <- count.p.value_2PCos_phy_locom[,p]/length(trees)

}

#save(Rsq_2PCos_phy_locom, count.p.value_2PCos_phy_locom, ratio_p.value_2PCos_phy_locom, file = "./output/2PCos_phy_locom.RData")

#load("./output/2PCos_phy_locom.RData")

############

Rsqs_2PCos <- data.frame("FL" = Rsq_2PCos_FL,

"BM" = Rsq_2PCos_BM,

"biog" = Rsq_2PCos_BIOGEO,

"phy" = Rsq_2PCos_phylo,

"locom" = Rsq_2PCos_locom,

"FL+biog" = Rsq_2PCos_FL_BIOGEO,

"FL+phy" = Rsq_2PCos_FL_phylo,

"FL+locom" = Rsq_2PCos_FL_locom,

"FL+biog+phy" = Rsq_2PCos_FL_BIOGEO_phylo,

"FL+phylo+locom" = Rsq_2PCos_FL_phylo_locom,

"BM+biog" = Rsq_2PCos_BM_biogeo,

"BM+phy" = Rsq_2PCos_BM_phylo,

"BM+locom" = Rsq_2PCos_BM_locom,

"biog+phy" = Rsq_2PCos_biogeo_phy,

"biog+locom" = Rsq_2PCos_biogeo_locom,

"phy+locom" = Rsq_2PCos_phy_locom)

p.values_2PCos <- data.frame("FL" = ratio_p.value_2PCos_FL,

"BM" = ratio_p.value_2PCos_BM,

"biog" = ratio_p.value_2PCos_BIOGEO,

"phy" = ratio_p.value_2PCos_phylo,

"locom" = ratio_p.value_2PCos_locom,

"FL+biog" = ratio_p.value_2PCos_FL_BIOGEO,

"FL+phy" = ratio_p.value_2PCos_FL_phylo,

"FL+locom" = ratio_p.value_2PCos_FL_locom,

"FL+biog+phy" = ratio_p.value_2PCos_FL_BIOGEO_phylo,

"FL+phylo+locom" = ratio_p.value_2PCos_FL_phylo_locom,

"BM+biog" = ratio_p.value_2PCos_BM_biogeo,

"BM+phy" = ratio_p.value_2PCos_BM_phylo,

"BM+locom" = ratio_p.value_2PCos_BM_locom,

"biog+phy" = ratio_p.value_2PCos_biogeo_phy,

"biog+locom" = ratio_p.value_2PCos_biogeo_locom,

"phy+locom" = ratio_p.value_2PCos_phy_locom)

#pdf(file = "graphics/Model_test_2PCos.pdf", width = 24, height = 10)

boxplot(Rsqs_2PCos, ylab = "Rsq",

xlab = "Models 2PCos",

las = 2)

#dev.off()

######GRAFICO GGPLOT########

PCO12_mass <- data.frame(PCo1 = pcoa_result_vectors_prun[,1],

PCo2 = pcoa_result_vectors_prun[,2],

log_BM = mass_data)

PCO12_mass_ratio <- data.frame(PCo1 = pcoa_result_vectors_prun[,1],

PCo2 = pcoa_result_vectors_prun[,2],

log_BM_ratio = mass_data+circum.ratio_data)

PCO34_mass_ratio <- data.frame(PCo3 = pcoa_result_vectors_prun[,3],

PCo4 = pcoa_result_vectors_prun[,4],

log_ratio = circum.ratio_data)

PCO12_residuals_PCO1 <- data.frame(PCo1 = pcoa_result_vectors_prun[,1],

PCo2 = pcoa_result_vectors_prun[,2],

residuals = residuals_PCos_BM_ratio[,1])

PCO12_locom <- data.frame(PCo1 = pcoa_result_vectors_prun2[,1],

PCo2 = pcoa_result_vectors_prun2[,2],

locomotion = locomotion_data2)

PCO12_biogeo <- data.frame(PCo1 = pcoa_result_vectors_prun2[,1],

PCo2 = pcoa_result_vectors_prun2[,2],

biogeography = biogeo_data2)

#pdf(file = "graphics/Morphospace_PCo1_vs_PCo2_BM_gradient.pdf", width = 10, height = 6)

ggplot(PCO12_mass, aes(x=PCo1, y=PCo2, color=log_BM)) + geom_point(shape=20,size=3,stroke=1) + scale_color_gradient(low="blue", high="red")

#dev.off()

#pdf(file = "graphics/Morphospace_PCo1_vs_PCo2_BM_ratio_gradient.pdf", width = 10, height = 6)

ggplot(PCO12_mass_ratio, aes(x=PCo1, y=PCo2, color=log_BM_ratio)) + geom_point(shape=20,size=3,stroke=1) + scale_color_gradient(low="blue", high="red")

#dev.off()

#pdf(file = "graphics/Morphospace_PCo3_vs_PCo4_ratio_gradient.pdf", width = 10, height = 6)

ggplot(PCO34_mass_ratio, aes(x=PCo3, y=PCo4, color=log_ratio)) + geom_point(shape=20,size=3,stroke=1) + scale_color_gradient(low="blue", high="red")

#dev.off()

#pdf(file = "graphics/Morphospace_PCo1_vs_PCo2_residuals_BM+ratio_gradient.pdf", width = 10, height = 6)

ggplot(PCO12_residuals_PCO1, aes(x=PCo1, y=PCo2, color=residuals_PCos_BM_ratio[,1])) + geom_point(shape=20,size=3,stroke=1) + scale_color_gradient(low="blue", high="red")

#dev.off()

#pdf(file = "graphics/Morphospace_2PCo1_vs_2PCo2_locomotion.pdf", width = 10, height = 6)

ggplot(PCO12_locom, aes(x=PCo1, y=PCo2, color=locomotion_data2)) + geom_point(shape=20,size=3,stroke=1)

#dev.off()

#pdf(file = "graphics/Morphospace_2PCo1_vs_2PCo2_biogeography.pdf", width = 10, height = 6)

ggplot(PCO12_biogeo, aes(x=PCo1, y=PCo2, color=biogeo_data2)) + geom_point(shape=20,size=3,stroke=1)

#dev.off()

#pdf(file = "graphics/Mapping_residuals_BM+ratio_gradient.pdf", width = 10, height = 6)

contMap(ladderize(time.trees.drop[[1]]),residuals_PCos_BM_ratio[,1],fsize=c(0.7,1))

#dev.off()

#pdf(file = "graphics/Mapping_BM_gradient.pdf", width = 10, height = 6)

contMap(ladderize(time.trees.drop[[1]]),mass_data,fsize=c(0.7,1))

#dev.off()

#############################################

B_red <- setdiff(B,c("Barapasaurus","Isanosaurus"))

C_red <- setdiff(C,c("Barapasaurus","Isanosaurus"))

D_red <- setdiff(D,c("Barapasaurus","Isanosaurus"))

bins_red <- list(A, B_red, C_red, D_red)

#pdf(file = "graphics/Morphospace_PCo1_vs_PCo2_Carnian.pdf", width = 6, height = 6)

plot(x = NULL,

y = NULL,

xlim = c(min(pcoa_result$vectors.cor[, 1]), max(pcoa_result$vectors.cor[, 1])),

ylim = c(min(pcoa_result$vectors.cor[, 2]), max(pcoa_result$vectors.cor[, 2])),

xlab = "PCo 1 (9.82%)",

ylab = "PCo 2 (3.90%)")

abline(h = 0, lty = 2)

abline(v = 0, lty = 2)

points(x = pcoa_result$vectors.cor[, 1],

y = pcoa_result$vectors.cor[, 2],

col = "black",

pch = 19)

points(x = pcoa_result$vectors.cor[c("Isanosaurus","Barapasaurus"), 1],

y = pcoa_result$vectors.cor[c("Isanosaurus","Barapasaurus"), 2],

col = "antiquewhite4",

pch = 19)

points(x = pcoa_result$vectors.cor[A, 1],

y = pcoa_result$vectors.cor[A, 2],

col = "brown1",

pch = 19)

Plot_ConvexHull(xcoord = pcoa_result$vectors.cor[A, 1], ycoord = pcoa_result$vectors.cor[A, 2], lcolor = "brown1")

#dev.off()

#pdf(file = "graphics/Morphospace_PCo1_vs_PCo2_Norian_Rhaetian.pdf", width = 6, height = 6)

plot(x = NULL,

y = NULL,

xlim = c(min(pcoa_result$vectors.cor[, 1]), max(pcoa_result$vectors.cor[, 1])),

ylim = c(min(pcoa_result$vectors.cor[, 2]), max(pcoa_result$vectors.cor[, 2])),

xlab = "PCo 1 (9.82%)",

ylab = "PCo 2 (3.90%)")

abline(h = 0, lty = 2)

abline(v = 0, lty = 2)

points(x = pcoa_result$vectors.cor[, 1],

y = pcoa_result$vectors.cor[, 2],

col = "black",

pch = 19)

points(x = pcoa_result$vectors.cor[c("Isanosaurus","Barapasaurus"), 1],

y = pcoa_result$vectors.cor[c("Isanosaurus","Barapasaurus"), 2],

col = "antiquewhite4",

pch = 19)

points(x = pcoa_result$vectors.cor[B_red, 1],

y = pcoa_result$vectors.cor[B_red, 2],

col = "darkorange",

pch = 19)

Plot_ConvexHull(xcoord = pcoa_result$vectors.cor[B_red, 1], ycoord = pcoa_result$vectors.cor[B_red, 2], lcolor = "darkorange")

#dev.off()

#pdf(file = "graphics/Morphospace_PCo1_vs_PCo2_pre_Toarcian.pdf", width = 6, height = 6)

plot(x = NULL,

y = NULL,

xlim = c(min(pcoa_result$vectors.cor[, 1]), max(pcoa_result$vectors.cor[, 1])),

ylim = c(min(pcoa_result$vectors.cor[, 2]), max(pcoa_result$vectors.cor[, 2])),

xlab = "PCo 1 (9.82%)",

ylab = "PCo 2 (3.90%)")

abline(h = 0, lty = 2)

abline(v = 0, lty = 2)

points(x = pcoa_result$vectors.cor[, 1],

y = pcoa_result$vectors.cor[, 2],

col = "black",

pch = 19)

points(x = pcoa_result$vectors.cor[c("Isanosaurus","Barapasaurus"), 1],

y = pcoa_result$vectors.cor[c("Isanosaurus","Barapasaurus"), 2],

col = "antiquewhite4",

pch = 19)

points(x = pcoa_result$vectors.cor[C_red, 1],

y = pcoa_result$vectors.cor[C_red, 2],

col = "cyan3",

pch = 19)

Plot_ConvexHull(xcoord = pcoa_result$vectors.cor[C_red, 1], ycoord = pcoa_result$vectors.cor[C_red, 2], lcolor = "cyan3")

#dev.off()

#pdf(file = "graphics/Morphospace_PCo1_vs_PCo2_Toarcian_Middle_Jurassic.pdf", width = 6, height = 6)

plot(x = NULL,

y = NULL,

xlim = c(min(pcoa_result$vectors.cor[, 1]), max(pcoa_result$vectors.cor[, 1])),

ylim = c(min(pcoa_result$vectors.cor[, 2]), max(pcoa_result$vectors.cor[, 2])),

xlab = "PCo 1 (9.82%)",

ylab = "PCo 2 (3.90%)")

abline(h = 0, lty = 2)

abline(v = 0, lty = 2)

points(x = pcoa_result$vectors.cor[, 1],

y = pcoa_result$vectors.cor[, 2],

col = "black",

pch = 19)

points(x = pcoa_result$vectors.cor[c("Isanosaurus","Barapasaurus"), 1],

y = pcoa_result$vectors.cor[c("Isanosaurus","Barapasaurus"), 2],

col = "antiquewhite4",

pch = 19)

points(x = pcoa_result$vectors.cor[D_red, 1],

y = pcoa_result$vectors.cor[D_red, 2],

col = "blue3",

pch = 19)

Plot_ConvexHull(xcoord = pcoa_result$vectors.cor[D_red, 1], ycoord = pcoa_result$vectors.cor[D_red, 2], lcolor = "blue3")

#dev.off()

#Graphics for displacements

library("shape")

centroids_PCo1_PCo2 <- list()

for (i in 1:length(bins_red)){

centroids_PCo1_PCo2[[i]] <- c(mean(pcoa_result$vectors.cor[bins_red[[i]], 1]),mean(pcoa_result$vectors.cor[bins_red[[i]], 2]))

}

#pdf(file = "graphics/Morphospace_PCo1_vs_PCo2_displacements2.pdf", width = 9, height = 9)

plot(x = NULL,

y = NULL,

xlim = c(min(pcoa_result$vectors.cor[, 1]), max(pcoa_result$vectors.cor[, 1])),

ylim = c(min(pcoa_result$vectors.cor[, 2]), max(pcoa_result$vectors.cor[, 2])),

xlab = "PCo 1 (9.82%)",

ylab = "PCo 2 (3.90%)")

abline(h = 0, lty = 2)

abline(v = 0, lty = 2)

Plot_ConvexHull(xcoord = pcoa_result$vectors.cor[A, 1], ycoord = pcoa_result$vectors.cor[A, 2], lcolor = "brown1")

Plot_ConvexHull(xcoord = pcoa_result$vectors.cor[B_red, 1], ycoord = pcoa_result$vectors.cor[B_red, 2], lcolor = "darkorange")

Plot_ConvexHull(xcoord = pcoa_result$vectors.cor[C_red, 1], ycoord = pcoa_result$vectors.cor[C_red, 2], lcolor = "cyan3")

Plot_ConvexHull(xcoord = pcoa_result$vectors.cor[D_red, 1], ycoord = pcoa_result$vectors.cor[D_red, 2], lcolor = "blue3")

points(x = centroids_PCo1_PCo2[[1]][1],

y = centroids_PCo1_PCo2[[1]][2],

col = "brown1",

pch = 19,

cex = 3)

points(x = centroids_PCo1_PCo2[[2]][1],

y = centroids_PCo1_PCo2[[2]][2],

col = "darkorange",

pch = 19,

cex = 3)

points(x = centroids_PCo1_PCo2[[3]][1],

y = centroids_PCo1_PCo2[[3]][2],

col = "cyan3",

pch = 19,

cex = 3)

points(x = centroids_PCo1_PCo2[[4]][1],

y = centroids_PCo1_PCo2[[4]][2],

col = "blue3",

pch = 19,

cex = 3)

Arrows(centroids_PCo1_PCo2[[3]][1], centroids_PCo1_PCo2[[3]][2], centroids_PCo1_PCo2[[4]][1], centroids_PCo1_PCo2[[4]][2],

code = 2, arr.type = "triangle", arr.length = 0.1, arr.width = log(Displacements_previous_bin_result_prun_Barapa_Isano$Disp[4]),

arr.adj = 1, col = "cyan4", lwd = log(Displacements_previous_bin_result_prun_Barapa_Isano$Disp[4])*20)

Arrows(centroids_PCo1_PCo2[[2]][1], centroids_PCo1_PCo2[[2]][2], centroids_PCo1_PCo2[[3]][1], centroids_PCo1_PCo2[[3]][2],

code = 2, arr.type = "triangle", arr.length = 0.1, arr.width = log(Displacements_previous_bin_result_prun_Barapa_Isano$Disp[3]),

arr.adj = 1, col = "darkorange2", lwd = log(Displacements_previous_bin_result_prun_Barapa_Isano$Disp[3])*20)

Arrows(centroids_PCo1_PCo2[[1]][1], centroids_PCo1_PCo2[[1]][2], centroids_PCo1_PCo2[[2]][1], centroids_PCo1_PCo2[[2]][2],

code = 2, arr.type = "triangle", arr.length = 0.1, arr.width = log(Displacements_previous_bin_result_prun_Barapa_Isano$Disp[2]),

arr.adj = 1, col = "brown4", lwd = log(Displacements_previous_bin_result_prun_Barapa_Isano$Disp[2])*20)

#dev.off()

#Phylomorphospace

#Prune non-sauropodomorph taxa

time.trees.2plot.drop <- list()

for (j in 1:length(trees)) {

time.trees.2plot.drop[[j]] <- drop.tip(phy = trees[[j]],

tip = c("Euparkeria","Crurotarsi","Marasuchus","Ornithischia","Agnosphitys","Silesaurus","Neotheropoda","Staurikosaurus","Chindesaurus","Herrerasaurus","Guaibasaurus","Neosauropoda"))

}

ages.new <- data[,c(2,3)]

rownames(ages.new) <- data[,1]

rownames(ages.new)[65:67] <- c("Ngwevu", "Irisosaurus", "Schleitheimia")

time.trees2plot <- list()

for (i in 1:length(trees)) {

time.trees2plot[[i]] <- timePaleoPhy(tree = time.trees.2plot.drop[[i]],

timeData = ages.new,

type="mbl",

vartime=0.5)

}

model_data3 <- data[,c(2,5,8)]

rownames(model_data3) <- data$Taxon

model_data_red3 <- na.omit(model_data3)

model_data_red3 <- model_data_red3[setdiff(rownames(model_data_red3),trim_dist_matrix$removed_taxa),]

locomotion_data3 <- model_data_red3$Locomotion

names(locomotion_data3) <- rownames(model_data_red3)

BM_data3 <- log(model_data_red3$Mass)

names(BM_data3) <- rownames(model_data_red3)

FAD_data3 <- model_data_red3$FAD

names(FAD_data3) <- rownames(model_data_red3)

pcoa_result3 <- pcoa_result

pcoa_result3$vectors.cor <- pcoa_result$vectors.cor[intersect(names(pcoa_result$vectors.cor[,1]),names(locomotion_data3)),]

PCO12_locom3 <- data.frame(PCo1 = pcoa_result3$vectors.cor[, 1],

PCo2 = pcoa_result3$vectors.cor[,2],

PCo3 = pcoa_result3$vectors.cor[,3],

FAD = FAD_data3,

locomotion = locomotion_data3,

body_mass = BM_data3)

ggplot(PCO12_locom3, aes(x=PCo1, y=PCo2, color=locomotion)) + geom_point(shape=20,size=3,stroke=1)

to.drop_taxa <- setdiff(time.trees2plot[[23]]$tip.label,names(locomotion_data3))

time.trees2plot_red <- list()

for (k in 1:length(trees)) {

time.trees2plot_red[[k]] <- drop.tip(phy = time.trees2plot[[k]],

tip = to.drop_taxa)

}

AA<-contMap(time.trees2plot_red[[23]],BM_data3)

AA$cols[]<-hcl.colors(1001,palette="viridis")

AA$cols[]<-rainbow(1001,start=0.7,end=0)

plot(AA,legend=FALSE)

dev.off()

phylomorphospace(tree = AA$tree,

X = PCO12_locom3[,c(1,2)],

colors=AA$cols,lwd=3,

label = "off",

node.by.map=TRUE,

xlab="PCo 1 (9.82%)",

ylab="PCo 2 (3.90%)")

add.color.bar(leg = 0.2,

AA$cols,

title="log(body mass)",

lims=c(min(BM_data3),max(BM_data3)),

digits=2)

bipeds3 <- locomotion_data3[locomotion_data3=="Biped"]

quadrupeds3 <- locomotion_data3[locomotion_data3=="Quadruped"]

data[,c(1,9)]

locomotion_meas <- data[,9]

names(locomotion_meas) <- data$Taxon

locomotion_meas <- na.omit(locomotion_meas)

bipeds_meas <- locomotion_meas[locomotion_meas=="Biped"]

quadrupeds_meas <- locomotion_meas[locomotion_meas=="Quadruped"]

bipeds_non_meas <- setdiff(names(bipeds3),names(bipeds_meas))

quadrupeds_non_meas <- setdiff(names(quadrupeds3),names(quadrupeds_meas))

#pdf(file = "graphics/Phylomorphospace_PCo1_vs_PCo2_locomotion_open_fill_circles.pdf", width = 9, height = 9)

phylomorphospace(time.trees2plot_red[[23]],

X = PCO12_locom3[,c(1,2)],

xlab="PCo 1 (9.82%)",

ylab="PCo 2 (3.90%)",

label = "off",

node.by.map=TRUE,

node.size=c(0,0))

points(x = pcoa_result3$vectors.cor[bipeds_non_meas, 1],

y = pcoa_result3$vectors.cor[bipeds_non_meas, 2],

col = "#E69F00",

pch = 21,

bg = "white",

lwd = 2,

cex = 1.5)

points(x = pcoa_result3$vectors.cor[names(bipeds_meas), 1],

y = pcoa_result3$vectors.cor[names(bipeds_meas), 2],

col = "#E69F00",

pch = 19,

cex = 1.5)

points(x = pcoa_result3$vectors.cor[names(quadrupeds3), 1],

y = pcoa_result3$vectors.cor[names(quadrupeds3), 2],

col = "#CC79A7",

pch = 19,

cex = 1.5)

points(x = pcoa_result3$vectors.cor[quadrupeds_non_meas, 1],

y = pcoa_result3$vectors.cor[quadrupeds_non_meas, 2],

col = "#CC79A7",

pch = 21,

bg = "white",

lwd = 2,

cex = 1.5)

text(x = PCO12_locom3[,1],

y = PCO12_locom3[,2],

rownames(PCO12_locom3),

cex = 0.7,

pos = 4)

#dev.off()

phylomorphospace3d(time.trees2plot_red[[23]],

X = PCO12_locom3[,c(1,2,4)],

method="static")
