# Supplementary material for: Sauropodomorph evolution across the Triassic–Jurassic boundary: body size, locomotion, and their influence on morphological disparity
Source: Sci Rep. 2021 Nov 18;11:22534. doi: 10.1038/s41598-021-01120-w (PMC8602272; doi:10.1038/s41598-021-01120-w)
Supplement: Supplementary file 9 — Supplementary Information 9. [file 41598_2021_1120_MOESM9_ESM.docx]

(Euparkeria,(Crurotarsi,(Marasuchus,(Silesaurus,(Ornithischia,((Agnosphitys,((Guaibasaurus,(Neotheropoda,Chindesaurus)),(Buriolestes,(Panphagia,(Eoraptor,((Pampadromaeus,(Saturnalia,Chromogisaurus)),(Bagualosaurus,(Jaklapalisaurus,(Nambalia,((Thecodontosaurus,Pantydraco),(Efraasia,(Plateosauravus,(Ruehleia,(((Unaysaurus,(Pradhania,Macrocollum)),((Sarahsaurus,((Ngwevu,(Xixipiosaurus,((Coloradisaurus,(Glacialisaurus,Lufengosaurus)),(Massospondylus_carinatus,(Adeopapposaurus,Leyesaurus))))),(Yunnanosaurus_huangi,((Seitaad,(Anchisaurus,((Irisosaurus,(Sefapanosaurus,(Yizhousaurus,(Aardonyx,(NMQR1551,(NMRQ3314,(Blikanasaurus,((Camelotia,((Meroktenos,(Ledumahadi,Kholumolumo)),(Lessemsaurus,(Ingentia,Antetonitrus)))),((Schleitheimia,(Isanosaurus,((Tazoudasaurus,Vulcanodon),((Shunosaurus,((Spinophorosaurus,(Omeisaurus,Mamenchisaurus)),(Cetiosaurus,Neosauropoda))),(Patagosaurus,Barapasaurus))))),(Pulanesaura,Gongxianosaurus)))))))))),(Mussaurus,Leonerasaurus)))),(Xingxiulong,(Jingshanosaurus,Chuxiongosaurus)))))),(Eucnemesaurus_fortis,(Riojasaurus,Eucnemesaurus_entaxonis)))),(Plateosaurus_gracilis,(Plateosaurus_ingens,Plateosaurus_engelhardti)))))))))))))))),(Staurikosaurus,Herrerasaurus)))))));

(Euparkeria,(Crurotarsi,(Marasuchus,(Silesaurus,(Ornithischia,((Agnosphitys,((Guaibasaurus,(Neotheropoda,Chindesaurus)),(Buriolestes,(Panphagia,(Eoraptor,((Pampadromaeus,(Saturnalia,Chromogisaurus)),(Bagualosaurus,(Jaklapalisaurus,(Nambalia,((Thecodontosaurus,Pantydraco),(Efraasia,(Plateosauravus,(Ruehleia,(((Unaysaurus,(Pradhania,Macrocollum)),((Sarahsaurus,((Ngwevu,(Xixipiosaurus,((Coloradisaurus,(Glacialisaurus,Lufengosaurus)),(Massospondylus_carinatus,(Adeopapposaurus,Leyesaurus))))),(Yunnanosaurus_huangi,((Seitaad,(Anchisaurus,((Irisosaurus,(Yizhousaurus,((Sefapanosaurus,Aardonyx),(NMQR1551,(NMRQ3314,(Blikanasaurus,((Camelotia,((Meroktenos,(Ledumahadi,Kholumolumo)),(Lessemsaurus,(Ingentia,Antetonitrus)))),((Schleitheimia,(Isanosaurus,((Tazoudasaurus,Vulcanodon),((Shunosaurus,((Spinophorosaurus,(Omeisaurus,Mamenchisaurus)),(Cetiosaurus,Neosauropoda))),(Patagosaurus,Barapasaurus))))),(Pulanesaura,Gongxianosaurus))))))))),(Mussaurus,Leonerasaurus)))),(Xingxiulong,(Jingshanosaurus,Chuxiongosaurus)))))),(Eucnemesaurus_fortis,(Riojasaurus,Eucnemesaurus_entaxonis)))),(Plateosaurus_gracilis,(Plateosaurus_ingens,Plateosaurus_engelhardti)))))))))))))))),(Staurikosaurus,Herrerasaurus)))))));

(Euparkeria,(Crurotarsi,(Marasuchus,(Silesaurus,(Ornithischia,((Agnosphitys,((Guaibasaurus,(Neotheropoda,Chindesaurus)),(Buriolestes,(Panphagia,(Eoraptor,((Pampadromaeus,(Saturnalia,Chromogisaurus)),(Bagualosaurus,(Jaklapalisaurus,(Nambalia,((Thecodontosaurus,Pantydraco),(Efraasia,(Plateosauravus,(Ruehleia,(((Unaysaurus,(Pradhania,Macrocollum)),((Sarahsaurus,((Ngwevu,(Xixipiosaurus,((Coloradisaurus,(Glacialisaurus,Lufengosaurus)),(Massospondylus_carinatus,(Adeopapposaurus,Leyesaurus))))),(Yunnanosaurus_huangi,((Seitaad,(Anchisaurus,((Irisosaurus,(Sefapanosaurus,(Yizhousaurus,(Aardonyx,(NMQR1551,(NMRQ3314,(Blikanasaurus,((Camelotia,((Meroktenos,(Ledumahadi,Kholumolumo)),(Lessemsaurus,(Ingentia,Antetonitrus)))),(Pulanesaura,(Gongxianosaurus,(Schleitheimia,(Isanosaurus,((Tazoudasaurus,Vulcanodon),((Shunosaurus,((Spinophorosaurus,(Omeisaurus,Mamenchisaurus)),(Cetiosaurus,Neosauropoda))),(Patagosaurus,Barapasaurus))))))))))))))),(Mussaurus,Leonerasaurus)))),(Xingxiulong,(Jingshanosaurus,Chuxiongosaurus)))))),(Eucnemesaurus_fortis,(Riojasaurus,Eucnemesaurus_entaxonis)))),(Plateosaurus_gracilis,(Plateosaurus_ingens,Plateosaurus_engelhardti)))))))))))))))),(Staurikosaurus,Herrerasaurus)))))));

(Euparkeria,(Crurotarsi,(Marasuchus,(Silesaurus,(Ornithischia,((Agnosphitys,((Guaibasaurus,(Neotheropoda,Chindesaurus)),(Buriolestes,(Panphagia,(Eoraptor,((Pampadromaeus,(Saturnalia,Chromogisaurus)),(Bagualosaurus,(Jaklapalisaurus,(Nambalia,((Thecodontosaurus,Pantydraco),(Efraasia,(Plateosauravus,(Ruehleia,(((Unaysaurus,(Pradhania,Macrocollum)),((Sarahsaurus,((Ngwevu,(Xixipiosaurus,((Coloradisaurus,(Glacialisaurus,Lufengosaurus)),(Massospondylus_carinatus,(Adeopapposaurus,Leyesaurus))))),(Yunnanosaurus_huangi,((Seitaad,(Anchisaurus,((Irisosaurus,(Sefapanosaurus,(Yizhousaurus,(Aardonyx,(NMQR1551,(NMRQ3314,(Blikanasaurus,((Camelotia,((Meroktenos,(Ledumahadi,Kholumolumo)),(Lessemsaurus,(Ingentia,Antetonitrus)))),((Schleitheimia,(Isanosaurus,((Tazoudasaurus,Vulcanodon),((Shunosaurus,((Spinophorosaurus,Omeisaurus),(Mamenchisaurus,(Cetiosaurus,Neosauropoda)))),(Patagosaurus,Barapasaurus))))),(Pulanesaura,Gongxianosaurus)))))))))),(Mussaurus,Leonerasaurus)))),(Xingxiulong,(Jingshanosaurus,Chuxiongosaurus)))))),(Eucnemesaurus_fortis,(Riojasaurus,Eucnemesaurus_entaxonis)))),(Plateosaurus_gracilis,(Plateosaurus_ingens,Plateosaurus_engelhardti)))))))))))))))),(Staurikosaurus,Herrerasaurus)))))));

(Euparkeria,(Crurotarsi,(Marasuchus,(Silesaurus,(Ornithischia,((Agnosphitys,((Guaibasaurus,(Neotheropoda,Chindesaurus)),(Buriolestes,(Panphagia,(Eoraptor,((Saturnalia,Chromogisaurus),(Pampadromaeus,(Bagualosaurus,(Jaklapalisaurus,(Nambalia,((Thecodontosaurus,Pantydraco),(Efraasia,(Plateosauravus,(Ruehleia,(((Unaysaurus,(Pradhania,Macrocollum)),((Sarahsaurus,((Ngwevu,(Xixipiosaurus,((Coloradisaurus,(Glacialisaurus,Lufengosaurus)),(Massospondylus_carinatus,(Adeopapposaurus,Leyesaurus))))),(Yunnanosaurus_huangi,((Seitaad,(Anchisaurus,((Irisosaurus,(Sefapanosaurus,(Yizhousaurus,(Aardonyx,(NMQR1551,(NMRQ3314,(Blikanasaurus,((Camelotia,((Meroktenos,(Ledumahadi,Kholumolumo)),(Lessemsaurus,(Ingentia,Antetonitrus)))),((Schleitheimia,(Isanosaurus,((Tazoudasaurus,Vulcanodon),((Shunosaurus,((Spinophorosaurus,(Omeisaurus,Mamenchisaurus)),(Cetiosaurus,Neosauropoda))),(Patagosaurus,Barapasaurus))))),(Pulanesaura,Gongxianosaurus)))))))))),(Mussaurus,Leonerasaurus)))),(Xingxiulong,(Jingshanosaurus,Chuxiongosaurus)))))),(Eucnemesaurus_fortis,(Riojasaurus,Eucnemesaurus_entaxonis)))),(Plateosaurus_gracilis,(Plateosaurus_ingens,Plateosaurus_engelhardti))))))))))))))))),(Staurikosaurus,Herrerasaurus)))))));

(Euparkeria,(Crurotarsi,(Marasuchus,(Silesaurus,(Ornithischia,((Agnosphitys,((Guaibasaurus,(Neotheropoda,Chindesaurus)),(Buriolestes,(Panphagia,(Eoraptor,((Pampadromaeus,(Saturnalia,Chromogisaurus)),(Bagualosaurus,(Jaklapalisaurus,(Nambalia,((Thecodontosaurus,Pantydraco),(Efraasia,(Plateosauravus,(Ruehleia,(((Unaysaurus,(Pradhania,Macrocollum)),((Sarahsaurus,((Ngwevu,(Xixipiosaurus,((Coloradisaurus,(Glacialisaurus,Lufengosaurus)),(Massospondylus_carinatus,(Adeopapposaurus,Leyesaurus))))),(Yunnanosaurus_huangi,((Seitaad,(Anchisaurus,((Irisosaurus,(Sefapanosaurus,(Yizhousaurus,(Aardonyx,(NMQR1551,(NMRQ3314,(Blikanasaurus,((Camelotia,((Meroktenos,(Ledumahadi,Kholumolumo)),(Lessemsaurus,(Ingentia,Antetonitrus)))),((Schleitheimia,(Isanosaurus,((Tazoudasaurus,Vulcanodon),((Shunosaurus,(Spinophorosaurus,(Mamenchisaurus,(Omeisaurus,(Cetiosaurus,Neosauropoda))))),(Patagosaurus,Barapasaurus))))),(Pulanesaura,Gongxianosaurus)))))))))),(Mussaurus,Leonerasaurus)))),(Xingxiulong,(Jingshanosaurus,Chuxiongosaurus)))))),(Eucnemesaurus_fortis,(Riojasaurus,Eucnemesaurus_entaxonis)))),(Plateosaurus_gracilis,(Plateosaurus_ingens,Plateosaurus_engelhardti)))))))))))))))),(Staurikosaurus,Herrerasaurus)))))));

(Euparkeria,(Crurotarsi,(Marasuchus,(Silesaurus,(Ornithischia,((Agnosphitys,((Guaibasaurus,(Neotheropoda,Chindesaurus)),(Buriolestes,(Panphagia,(Eoraptor,((Pampadromaeus,(Saturnalia,Chromogisaurus)),(Bagualosaurus,(Jaklapalisaurus,(Nambalia,((Thecodontosaurus,Pantydraco),(Efraasia,(Plateosauravus,(Ruehleia,(((Unaysaurus,(Pradhania,Macrocollum)),((Sarahsaurus,((Ngwevu,(Xixipiosaurus,((Coloradisaurus,(Glacialisaurus,Lufengosaurus)),(Massospondylus_carinatus,(Adeopapposaurus,Leyesaurus))))),(Yunnanosaurus_huangi,((Seitaad,(Anchisaurus,((Irisosaurus,(Sefapanosaurus,(Yizhousaurus,(Aardonyx,(NMQR1551,(NMRQ3314,(Blikanasaurus,((Camelotia,((Meroktenos,(Ledumahadi,Kholumolumo)),(Lessemsaurus,(Ingentia,Antetonitrus)))),((Schleitheimia,(Isanosaurus,((Tazoudasaurus,Vulcanodon),((Shunosaurus,(Spinophorosaurus,(Omeisaurus,(Mamenchisaurus,(Cetiosaurus,Neosauropoda))))),(Patagosaurus,Barapasaurus))))),(Pulanesaura,Gongxianosaurus)))))))))),(Mussaurus,Leonerasaurus)))),(Xingxiulong,(Jingshanosaurus,Chuxiongosaurus)))))),(Eucnemesaurus_fortis,(Riojasaurus,Eucnemesaurus_entaxonis)))),(Plateosaurus_gracilis,(Plateosaurus_ingens,Plateosaurus_engelhardti)))))))))))))))),(Staurikosaurus,Herrerasaurus)))))));

(Euparkeria,(Crurotarsi,(Marasuchus,(Silesaurus,(Ornithischia,((Agnosphitys,((Guaibasaurus,(Neotheropoda,Chindesaurus)),(Buriolestes,(Pampadromaeus,(Panphagia,(Eoraptor,((Saturnalia,Chromogisaurus),(Bagualosaurus,(Jaklapalisaurus,(Nambalia,((Thecodontosaurus,Pantydraco),(Efraasia,(Plateosauravus,(Ruehleia,(((Unaysaurus,(Pradhania,Macrocollum)),((Sarahsaurus,((Ngwevu,(Xixipiosaurus,((Coloradisaurus,(Glacialisaurus,Lufengosaurus)),(Massospondylus_carinatus,(Adeopapposaurus,Leyesaurus))))),(Yunnanosaurus_huangi,((Seitaad,(Anchisaurus,((Irisosaurus,(Sefapanosaurus,(Yizhousaurus,(Aardonyx,(NMQR1551,(NMRQ3314,(Blikanasaurus,((Camelotia,((Meroktenos,(Ledumahadi,Kholumolumo)),(Lessemsaurus,(Ingentia,Antetonitrus)))),((Schleitheimia,(Isanosaurus,((Tazoudasaurus,Vulcanodon),((Shunosaurus,((Spinophorosaurus,(Omeisaurus,Mamenchisaurus)),(Cetiosaurus,Neosauropoda))),(Patagosaurus,Barapasaurus))))),(Pulanesaura,Gongxianosaurus)))))))))),(Mussaurus,Leonerasaurus)))),(Xingxiulong,(Jingshanosaurus,Chuxiongosaurus)))))),(Eucnemesaurus_fortis,(Riojasaurus,Eucnemesaurus_entaxonis)))),(Plateosaurus_gracilis,(Plateosaurus_ingens,Plateosaurus_engelhardti))))))))))))))))),(Staurikosaurus,Herrerasaurus)))))));

(Euparkeria,(Crurotarsi,(Marasuchus,(Silesaurus,(Ornithischia,((Agnosphitys,((Guaibasaurus,(Neotheropoda,Chindesaurus)),(Buriolestes,((Eoraptor,((Saturnalia,Chromogisaurus),(Bagualosaurus,(Jaklapalisaurus,(Nambalia,((Thecodontosaurus,Pantydraco),(Efraasia,(Plateosauravus,(Ruehleia,(((Unaysaurus,(Pradhania,Macrocollum)),((Sarahsaurus,((Ngwevu,(Xixipiosaurus,((Coloradisaurus,(Glacialisaurus,Lufengosaurus)),(Massospondylus_carinatus,(Adeopapposaurus,Leyesaurus))))),(Yunnanosaurus_huangi,((Seitaad,(Anchisaurus,((Irisosaurus,(Sefapanosaurus,(Yizhousaurus,(Aardonyx,(NMQR1551,(NMRQ3314,(Blikanasaurus,((Camelotia,((Meroktenos,(Ledumahadi,Kholumolumo)),(Lessemsaurus,(Ingentia,Antetonitrus)))),((Schleitheimia,(Isanosaurus,((Tazoudasaurus,Vulcanodon),((Shunosaurus,((Spinophorosaurus,(Omeisaurus,Mamenchisaurus)),(Cetiosaurus,Neosauropoda))),(Patagosaurus,Barapasaurus))))),(Pulanesaura,Gongxianosaurus)))))))))),(Mussaurus,Leonerasaurus)))),(Xingxiulong,(Jingshanosaurus,Chuxiongosaurus)))))),(Eucnemesaurus_fortis,(Riojasaurus,Eucnemesaurus_entaxonis)))),(Plateosaurus_gracilis,(Plateosaurus_ingens,Plateosaurus_engelhardti)))))))))))),(Panphagia,Pampadromaeus))))),(Staurikosaurus,Herrerasaurus)))))));

(Euparkeria,(Crurotarsi,(Marasuchus,(Silesaurus,(Ornithischia,((Agnosphitys,((Guaibasaurus,(Neotheropoda,Chindesaurus)),((Panphagia,(Eoraptor,((Saturnalia,Chromogisaurus),(Bagualosaurus,(Jaklapalisaurus,(Nambalia,((Thecodontosaurus,Pantydraco),(Efraasia,(Plateosauravus,(Ruehleia,(((Unaysaurus,(Pradhania,Macrocollum)),((Sarahsaurus,((Ngwevu,(Xixipiosaurus,((Coloradisaurus,(Glacialisaurus,Lufengosaurus)),(Massospondylus_carinatus,(Adeopapposaurus,Leyesaurus))))),(Yunnanosaurus_huangi,((Seitaad,(Anchisaurus,((Irisosaurus,(Sefapanosaurus,(Yizhousaurus,(Aardonyx,(NMQR1551,(NMRQ3314,(Blikanasaurus,((Camelotia,((Meroktenos,(Ledumahadi,Kholumolumo)),(Lessemsaurus,(Ingentia,Antetonitrus)))),((Schleitheimia,(Isanosaurus,((Tazoudasaurus,Vulcanodon),((Shunosaurus,((Spinophorosaurus,(Omeisaurus,Mamenchisaurus)),(Cetiosaurus,Neosauropoda))),(Patagosaurus,Barapasaurus))))),(Pulanesaura,Gongxianosaurus)))))))))),(Mussaurus,Leonerasaurus)))),(Xingxiulong,(Jingshanosaurus,Chuxiongosaurus)))))),(Eucnemesaurus_fortis,(Riojasaurus,Eucnemesaurus_entaxonis)))),(Plateosaurus_gracilis,(Plateosaurus_ingens,Plateosaurus_engelhardti))))))))))))),(Buriolestes,Pampadromaeus)))),(Staurikosaurus,Herrerasaurus)))))));

(Euparkeria,(Crurotarsi,(Marasuchus,(Silesaurus,(Agnosphitys,(Ornithischia,(((Guaibasaurus,(Neotheropoda,Chindesaurus)),(Buriolestes,(Pampadromaeus,(Panphagia,(Eoraptor,((Saturnalia,Chromogisaurus),(Bagualosaurus,(Jaklapalisaurus,(Nambalia,((Thecodontosaurus,Pantydraco),(Efraasia,(Plateosauravus,(Ruehleia,(((Unaysaurus,(Pradhania,Macrocollum)),((Sarahsaurus,((Ngwevu,(Xixipiosaurus,((Coloradisaurus,(Glacialisaurus,Lufengosaurus)),(Massospondylus_carinatus,(Adeopapposaurus,Leyesaurus))))),(Yunnanosaurus_huangi,((Seitaad,(Anchisaurus,((Yizhousaurus,((Sefapanosaurus,Aardonyx),(NMQR1551,(NMRQ3314,(Blikanasaurus,((Camelotia,((Meroktenos,(Ledumahadi,Kholumolumo)),(Lessemsaurus,(Ingentia,Antetonitrus)))),(Pulanesaura,(Gongxianosaurus,(Schleitheimia,(Isanosaurus,((Tazoudasaurus,Vulcanodon),((Shunosaurus,(Spinophorosaurus,(Mamenchisaurus,(Omeisaurus,(Cetiosaurus,Neosauropoda))))),(Patagosaurus,Barapasaurus))))))))))))),(Irisosaurus,(Mussaurus,Leonerasaurus))))),(Xingxiulong,(Jingshanosaurus,Chuxiongosaurus)))))),(Eucnemesaurus_fortis,(Riojasaurus,Eucnemesaurus_entaxonis)))),(Plateosaurus_gracilis,(Plateosaurus_ingens,Plateosaurus_engelhardti)))))))))))))))),(Staurikosaurus,Herrerasaurus))))))));

(Euparkeria,(Crurotarsi,(Marasuchus,(Silesaurus,(Agnosphitys,(Ornithischia,(((Guaibasaurus,(Neotheropoda,Chindesaurus)),(Buriolestes,(Pampadromaeus,(Panphagia,(Eoraptor,((Saturnalia,Chromogisaurus),(Bagualosaurus,(Jaklapalisaurus,(Nambalia,((Thecodontosaurus,Pantydraco),(Efraasia,(Plateosauravus,(Ruehleia,(((Unaysaurus,(Pradhania,Macrocollum)),((Sarahsaurus,((Ngwevu,(Xixipiosaurus,((Coloradisaurus,(Glacialisaurus,Lufengosaurus)),(Massospondylus_carinatus,(Adeopapposaurus,Leyesaurus))))),(Yunnanosaurus_huangi,((Seitaad,(Anchisaurus,((Irisosaurus,(Yizhousaurus,((Sefapanosaurus,Aardonyx),(NMQR1551,(NMRQ3314,(Blikanasaurus,((Camelotia,((Meroktenos,(Ledumahadi,Kholumolumo)),(Lessemsaurus,(Ingentia,Antetonitrus)))),(Pulanesaura,(Gongxianosaurus,(Schleitheimia,(Isanosaurus,((Tazoudasaurus,Vulcanodon),((Shunosaurus,(Spinophorosaurus,(Mamenchisaurus,(Omeisaurus,(Cetiosaurus,Neosauropoda))))),(Patagosaurus,Barapasaurus)))))))))))))),(Mussaurus,Leonerasaurus)))),(Xingxiulong,(Jingshanosaurus,Chuxiongosaurus)))))),(Eucnemesaurus_fortis,(Riojasaurus,Eucnemesaurus_entaxonis)))),(Plateosaurus_gracilis,(Plateosaurus_ingens,Plateosaurus_engelhardti)))))))))))))))),(Staurikosaurus,Herrerasaurus))))))));

(Euparkeria,(Crurotarsi,(Marasuchus,(Silesaurus,(Agnosphitys,(Ornithischia,(((Guaibasaurus,(Neotheropoda,Chindesaurus)),(Buriolestes,(Pampadromaeus,(Panphagia,(Eoraptor,((Saturnalia,Chromogisaurus),(Bagualosaurus,(Jaklapalisaurus,(Nambalia,((Thecodontosaurus,Pantydraco),(Efraasia,(Plateosauravus,(Ruehleia,(((Unaysaurus,(Pradhania,Macrocollum)),((Sarahsaurus,((Ngwevu,(Xixipiosaurus,((Coloradisaurus,(Glacialisaurus,Lufengosaurus)),(Massospondylus_carinatus,(Adeopapposaurus,Leyesaurus))))),(Yunnanosaurus_huangi,((Seitaad,(Anchisaurus,((Yizhousaurus,(Sefapanosaurus,Aardonyx)),((Mussaurus,Leonerasaurus),(Irisosaurus,(NMQR1551,(NMRQ3314,(Blikanasaurus,((Camelotia,((Meroktenos,(Ledumahadi,Kholumolumo)),(Lessemsaurus,(Ingentia,Antetonitrus)))),(Pulanesaura,(Gongxianosaurus,(Schleitheimia,(Isanosaurus,((Tazoudasaurus,Vulcanodon),((Shunosaurus,(Spinophorosaurus,(Mamenchisaurus,(Omeisaurus,(Cetiosaurus,Neosauropoda))))),(Patagosaurus,Barapasaurus)))))))))))))))),(Xingxiulong,(Jingshanosaurus,Chuxiongosaurus)))))),(Eucnemesaurus_fortis,(Riojasaurus,Eucnemesaurus_entaxonis)))),(Plateosaurus_gracilis,(Plateosaurus_ingens,Plateosaurus_engelhardti)))))))))))))))),(Staurikosaurus,Herrerasaurus))))))));

(Euparkeria,(Crurotarsi,(Marasuchus,(Silesaurus,(Agnosphitys,(Ornithischia,(((Guaibasaurus,(Neotheropoda,Chindesaurus)),(Buriolestes,(Pampadromaeus,(Panphagia,(Eoraptor,((Saturnalia,Chromogisaurus),(Bagualosaurus,(Jaklapalisaurus,(Nambalia,((Thecodontosaurus,Pantydraco),(Efraasia,(Plateosauravus,(Ruehleia,(((Unaysaurus,(Pradhania,Macrocollum)),((Sarahsaurus,((Ngwevu,(Xixipiosaurus,((Coloradisaurus,(Glacialisaurus,Lufengosaurus)),(Massospondylus_carinatus,(Adeopapposaurus,Leyesaurus))))),(Yunnanosaurus_huangi,((Seitaad,(Anchisaurus,((Yizhousaurus,((Sefapanosaurus,Aardonyx),(NMQR1551,(NMRQ3314,(Blikanasaurus,((Camelotia,((Meroktenos,(Ledumahadi,Kholumolumo)),(Lessemsaurus,(Ingentia,Antetonitrus)))),((Schleitheimia,(Isanosaurus,((Tazoudasaurus,Vulcanodon),((Shunosaurus,(Spinophorosaurus,(Mamenchisaurus,(Omeisaurus,(Cetiosaurus,Neosauropoda))))),(Patagosaurus,Barapasaurus))))),(Pulanesaura,Gongxianosaurus)))))))),(Irisosaurus,(Mussaurus,Leonerasaurus))))),(Xingxiulong,(Jingshanosaurus,Chuxiongosaurus)))))),(Eucnemesaurus_fortis,(Riojasaurus,Eucnemesaurus_entaxonis)))),(Plateosaurus_gracilis,(Plateosaurus_ingens,Plateosaurus_engelhardti)))))))))))))))),(Staurikosaurus,Herrerasaurus))))))));

(Euparkeria,(Crurotarsi,(Marasuchus,(Silesaurus,(Agnosphitys,(Ornithischia,(((Guaibasaurus,(Neotheropoda,Chindesaurus)),(Buriolestes,(Pampadromaeus,(Panphagia,(Eoraptor,((Saturnalia,Chromogisaurus),(Bagualosaurus,(Jaklapalisaurus,(Nambalia,((Thecodontosaurus,Pantydraco),(Efraasia,(Plateosauravus,(Ruehleia,(((Unaysaurus,(Pradhania,Macrocollum)),((Sarahsaurus,((Ngwevu,(Xixipiosaurus,((Coloradisaurus,(Glacialisaurus,Lufengosaurus)),(Massospondylus_carinatus,(Adeopapposaurus,Leyesaurus))))),(Yunnanosaurus_huangi,((Seitaad,(Anchisaurus,((Yizhousaurus,((Sefapanosaurus,Aardonyx),(NMQR1551,(NMRQ3314,(Blikanasaurus,((Camelotia,((Meroktenos,(Ledumahadi,Kholumolumo)),(Lessemsaurus,(Ingentia,Antetonitrus)))),(Pulanesaura,(Gongxianosaurus,(Schleitheimia,(Isanosaurus,((Tazoudasaurus,Vulcanodon),((Shunosaurus,((Spinophorosaurus,(Omeisaurus,Mamenchisaurus)),(Cetiosaurus,Neosauropoda))),(Patagosaurus,Barapasaurus))))))))))))),(Irisosaurus,(Mussaurus,Leonerasaurus))))),(Xingxiulong,(Jingshanosaurus,Chuxiongosaurus)))))),(Eucnemesaurus_fortis,(Riojasaurus,Eucnemesaurus_entaxonis)))),(Plateosaurus_gracilis,(Plateosaurus_ingens,Plateosaurus_engelhardti)))))))))))))))),(Staurikosaurus,Herrerasaurus))))))));

(Euparkeria,(Crurotarsi,(Marasuchus,(Silesaurus,(Ornithischia,(Agnosphitys,(((Guaibasaurus,(Neotheropoda,Chindesaurus)),(Buriolestes,(Pampadromaeus,(Panphagia,(Eoraptor,((Saturnalia,Chromogisaurus),(Bagualosaurus,(Jaklapalisaurus,(Nambalia,((Thecodontosaurus,Pantydraco),(Efraasia,(Plateosauravus,(Ruehleia,(((Unaysaurus,(Pradhania,Macrocollum)),((Sarahsaurus,((Ngwevu,(Xixipiosaurus,((Coloradisaurus,(Glacialisaurus,Lufengosaurus)),(Massospondylus_carinatus,(Adeopapposaurus,Leyesaurus))))),(Yunnanosaurus_huangi,((Seitaad,(Anchisaurus,((Yizhousaurus,((Sefapanosaurus,Aardonyx),(NMQR1551,(NMRQ3314,(Blikanasaurus,((Camelotia,((Meroktenos,(Ledumahadi,Kholumolumo)),(Lessemsaurus,(Ingentia,Antetonitrus)))),(Pulanesaura,(Gongxianosaurus,(Schleitheimia,(Isanosaurus,((Tazoudasaurus,Vulcanodon),((Shunosaurus,(Spinophorosaurus,(Mamenchisaurus,(Omeisaurus,(Cetiosaurus,Neosauropoda))))),(Patagosaurus,Barapasaurus))))))))))))),(Irisosaurus,(Mussaurus,Leonerasaurus))))),(Xingxiulong,(Jingshanosaurus,Chuxiongosaurus)))))),(Eucnemesaurus_fortis,(Riojasaurus,Eucnemesaurus_entaxonis)))),(Plateosaurus_gracilis,(Plateosaurus_ingens,Plateosaurus_engelhardti)))))))))))))))),(Staurikosaurus,Herrerasaurus))))))));

(Euparkeria,(Crurotarsi,(Marasuchus,(Silesaurus,(Ornithischia,((Agnosphitys,((Guaibasaurus,(Neotheropoda,Chindesaurus)),(Buriolestes,(Pampadromaeus,(Panphagia,(Eoraptor,((Saturnalia,Chromogisaurus),(Bagualosaurus,(Jaklapalisaurus,(Nambalia,((Thecodontosaurus,Pantydraco),(Efraasia,(Plateosauravus,(Ruehleia,(((Unaysaurus,(Pradhania,Macrocollum)),((Sarahsaurus,((Ngwevu,(Xixipiosaurus,((Coloradisaurus,(Glacialisaurus,Lufengosaurus)),(Massospondylus_carinatus,(Adeopapposaurus,Leyesaurus))))),(Yunnanosaurus_huangi,((Seitaad,(Anchisaurus,((Yizhousaurus,((Sefapanosaurus,Aardonyx),(NMQR1551,(NMRQ3314,(Blikanasaurus,((Camelotia,((Meroktenos,(Ledumahadi,Kholumolumo)),(Lessemsaurus,(Ingentia,Antetonitrus)))),(Pulanesaura,(Gongxianosaurus,(Schleitheimia,(Isanosaurus,((Tazoudasaurus,Vulcanodon),((Shunosaurus,(Spinophorosaurus,(Mamenchisaurus,(Omeisaurus,(Cetiosaurus,Neosauropoda))))),(Patagosaurus,Barapasaurus))))))))))))),(Irisosaurus,(Mussaurus,Leonerasaurus))))),(Xingxiulong,(Jingshanosaurus,Chuxiongosaurus)))))),(Eucnemesaurus_fortis,(Riojasaurus,Eucnemesaurus_entaxonis)))),(Plateosaurus_gracilis,(Plateosaurus_ingens,Plateosaurus_engelhardti))))))))))))))))),(Staurikosaurus,Herrerasaurus)))))));

(Euparkeria,(Crurotarsi,(Marasuchus,(Silesaurus,(Agnosphitys,(Ornithischia,(((Guaibasaurus,(Neotheropoda,Chindesaurus)),((Panphagia,(Eoraptor,((Saturnalia,Chromogisaurus),(Bagualosaurus,(Jaklapalisaurus,(Nambalia,((Thecodontosaurus,Pantydraco),(Efraasia,(Plateosauravus,(Ruehleia,(((Unaysaurus,(Pradhania,Macrocollum)),((Sarahsaurus,((Ngwevu,(Xixipiosaurus,((Coloradisaurus,(Glacialisaurus,Lufengosaurus)),(Massospondylus_carinatus,(Adeopapposaurus,Leyesaurus))))),(Yunnanosaurus_huangi,((Seitaad,(Anchisaurus,((Yizhousaurus,((Sefapanosaurus,Aardonyx),(NMQR1551,(NMRQ3314,(Blikanasaurus,((Camelotia,((Meroktenos,(Ledumahadi,Kholumolumo)),(Lessemsaurus,(Ingentia,Antetonitrus)))),(Pulanesaura,(Gongxianosaurus,(Schleitheimia,(Isanosaurus,((Tazoudasaurus,Vulcanodon),((Shunosaurus,(Spinophorosaurus,(Mamenchisaurus,(Omeisaurus,(Cetiosaurus,Neosauropoda))))),(Patagosaurus,Barapasaurus))))))))))))),(Irisosaurus,(Mussaurus,Leonerasaurus))))),(Xingxiulong,(Jingshanosaurus,Chuxiongosaurus)))))),(Eucnemesaurus_fortis,(Riojasaurus,Eucnemesaurus_entaxonis)))),(Plateosaurus_gracilis,(Plateosaurus_ingens,Plateosaurus_engelhardti))))))))))))),(Buriolestes,Pampadromaeus))),(Staurikosaurus,Herrerasaurus))))))));

(Euparkeria,(Crurotarsi,(Marasuchus,(Silesaurus,(Agnosphitys,(Ornithischia,(((Guaibasaurus,(Neotheropoda,Chindesaurus)),(Buriolestes,(Pampadromaeus,(Panphagia,(Eoraptor,((Saturnalia,Chromogisaurus),(Bagualosaurus,(Jaklapalisaurus,(Nambalia,((Thecodontosaurus,Pantydraco),(Efraasia,(Plateosauravus,(Ruehleia,(((Unaysaurus,(Pradhania,Macrocollum)),((Sarahsaurus,((Ngwevu,((Coloradisaurus,(Glacialisaurus,Lufengosaurus)),(Xixipiosaurus,(Massospondylus_carinatus,(Adeopapposaurus,Leyesaurus))))),(Yunnanosaurus_huangi,((Seitaad,(Anchisaurus,((Yizhousaurus,((Sefapanosaurus,Aardonyx),(NMQR1551,(NMRQ3314,(Blikanasaurus,((Camelotia,((Meroktenos,(Ledumahadi,Kholumolumo)),(Lessemsaurus,(Ingentia,Antetonitrus)))),(Pulanesaura,(Gongxianosaurus,(Schleitheimia,(Isanosaurus,((Tazoudasaurus,Vulcanodon),((Shunosaurus,(Spinophorosaurus,(Mamenchisaurus,(Omeisaurus,(Cetiosaurus,Neosauropoda))))),(Patagosaurus,Barapasaurus))))))))))))),(Irisosaurus,(Mussaurus,Leonerasaurus))))),(Xingxiulong,(Jingshanosaurus,Chuxiongosaurus)))))),(Eucnemesaurus_fortis,(Riojasaurus,Eucnemesaurus_entaxonis)))),(Plateosaurus_gracilis,(Plateosaurus_ingens,Plateosaurus_engelhardti)))))))))))))))),(Staurikosaurus,Herrerasaurus))))))));

(Euparkeria,(Crurotarsi,(Marasuchus,(Silesaurus,(Agnosphitys,(Ornithischia,(((Guaibasaurus,(Neotheropoda,Chindesaurus)),(Buriolestes,(Pampadromaeus,(Panphagia,(Eoraptor,((Saturnalia,Chromogisaurus),(Bagualosaurus,(Jaklapalisaurus,(Nambalia,((Thecodontosaurus,Pantydraco),(Efraasia,(Plateosauravus,(Ruehleia,(((Unaysaurus,(Pradhania,Macrocollum)),((Sarahsaurus,((Ngwevu,(Xixipiosaurus,((Coloradisaurus,(Glacialisaurus,Lufengosaurus)),(Massospondylus_carinatus,(Adeopapposaurus,Leyesaurus))))),(Yunnanosaurus_huangi,((Seitaad,(Anchisaurus,((Yizhousaurus,((Sefapanosaurus,Aardonyx),(NMQR1551,(NMRQ3314,(Blikanasaurus,((Camelotia,((Meroktenos,(Ledumahadi,Kholumolumo)),(Lessemsaurus,(Ingentia,Antetonitrus)))),(Pulanesaura,(Gongxianosaurus,(Schleitheimia,(Isanosaurus,((Tazoudasaurus,Vulcanodon),((Shunosaurus,(Spinophorosaurus,(Omeisaurus,(Mamenchisaurus,(Cetiosaurus,Neosauropoda))))),(Patagosaurus,Barapasaurus))))))))))))),(Irisosaurus,(Mussaurus,Leonerasaurus))))),(Xingxiulong,(Jingshanosaurus,Chuxiongosaurus)))))),(Eucnemesaurus_fortis,(Riojasaurus,Eucnemesaurus_entaxonis)))),(Plateosaurus_gracilis,(Plateosaurus_ingens,Plateosaurus_engelhardti)))))))))))))))),(Staurikosaurus,Herrerasaurus))))))));

(Euparkeria,(Crurotarsi,(Marasuchus,(Silesaurus,(Agnosphitys,(Ornithischia,(((Guaibasaurus,(Neotheropoda,Chindesaurus)),((Panphagia,(Eoraptor,((Saturnalia,Chromogisaurus),(Bagualosaurus,(Jaklapalisaurus,(Nambalia,((Thecodontosaurus,Pantydraco),(Efraasia,(Plateosauravus,(Ruehleia,(((Unaysaurus,(Pradhania,Macrocollum)),((Sarahsaurus,((Ngwevu,(Xixipiosaurus,((Coloradisaurus,(Glacialisaurus,Lufengosaurus)),(Massospondylus_carinatus,(Adeopapposaurus,Leyesaurus))))),(Yunnanosaurus_huangi,((Seitaad,(Anchisaurus,((Irisosaurus,(Sefapanosaurus,(Yizhousaurus,(Aardonyx,(NMQR1551,(NMRQ3314,(Blikanasaurus,((Camelotia,((Meroktenos,(Ledumahadi,Kholumolumo)),(Lessemsaurus,(Ingentia,Antetonitrus)))),(Pulanesaura,(Gongxianosaurus,(Schleitheimia,(Isanosaurus,((Tazoudasaurus,Vulcanodon),((Shunosaurus,((Spinophorosaurus,(Omeisaurus,Mamenchisaurus)),(Cetiosaurus,Neosauropoda))),(Patagosaurus,Barapasaurus))))))))))))))),(Mussaurus,Leonerasaurus)))),(Xingxiulong,(Jingshanosaurus,Chuxiongosaurus)))))),(Eucnemesaurus_entaxonis,(Riojasaurus,Eucnemesaurus_fortis)))),(Plateosaurus_gracilis,(Plateosaurus_ingens,Plateosaurus_engelhardti))))))))))))),(Buriolestes,Pampadromaeus))),(Staurikosaurus,Herrerasaurus))))))));

(Euparkeria,(Crurotarsi,(Marasuchus,(Silesaurus,(Agnosphitys,(Ornithischia,(((Guaibasaurus,(Neotheropoda,Chindesaurus)),((Panphagia,(Eoraptor,((Saturnalia,Chromogisaurus),(Bagualosaurus,(Jaklapalisaurus,(Nambalia,((Thecodontosaurus,Pantydraco),(Efraasia,(Plateosauravus,(Ruehleia,(((Unaysaurus,(Pradhania,Macrocollum)),((Sarahsaurus,((Ngwevu,(Xixipiosaurus,((Coloradisaurus,(Glacialisaurus,Lufengosaurus)),(Massospondylus_carinatus,(Adeopapposaurus,Leyesaurus))))),(Yunnanosaurus_huangi,((Seitaad,(Anchisaurus,((Irisosaurus,(Yizhousaurus,((Sefapanosaurus,Aardonyx),(NMQR1551,(NMRQ3314,(Blikanasaurus,((Camelotia,((Meroktenos,(Ledumahadi,Kholumolumo)),(Lessemsaurus,(Ingentia,Antetonitrus)))),(Pulanesaura,(Gongxianosaurus,(Schleitheimia,(Isanosaurus,((Tazoudasaurus,Vulcanodon),((Shunosaurus,((Spinophorosaurus,(Omeisaurus,Mamenchisaurus)),(Cetiosaurus,Neosauropoda))),(Patagosaurus,Barapasaurus)))))))))))))),(Mussaurus,Leonerasaurus)))),(Xingxiulong,(Jingshanosaurus,Chuxiongosaurus)))))),(Eucnemesaurus_entaxonis,(Riojasaurus,Eucnemesaurus_fortis)))),(Plateosaurus_gracilis,(Plateosaurus_ingens,Plateosaurus_engelhardti))))))))))))),(Buriolestes,Pampadromaeus))),(Staurikosaurus,Herrerasaurus))))))));

(Euparkeria,(Crurotarsi,(Marasuchus,(Silesaurus,(Agnosphitys,(Ornithischia,(((Guaibasaurus,(Neotheropoda,Chindesaurus)),((Panphagia,(Eoraptor,((Saturnalia,Chromogisaurus),(Bagualosaurus,(Jaklapalisaurus,(Nambalia,((Thecodontosaurus,Pantydraco),(Efraasia,(Plateosauravus,(Ruehleia,(((Unaysaurus,(Pradhania,Macrocollum)),((Sarahsaurus,((Ngwevu,(Xixipiosaurus,((Coloradisaurus,(Glacialisaurus,Lufengosaurus)),(Massospondylus_carinatus,(Adeopapposaurus,Leyesaurus))))),(Yunnanosaurus_huangi,((Seitaad,(Anchisaurus,((Irisosaurus,(Sefapanosaurus,(Yizhousaurus,(Aardonyx,(NMQR1551,(NMRQ3314,(Blikanasaurus,((Camelotia,((Meroktenos,(Ledumahadi,Kholumolumo)),(Lessemsaurus,(Ingentia,Antetonitrus)))),((Schleitheimia,(Isanosaurus,((Tazoudasaurus,Vulcanodon),((Shunosaurus,((Spinophorosaurus,(Omeisaurus,Mamenchisaurus)),(Cetiosaurus,Neosauropoda))),(Patagosaurus,Barapasaurus))))),(Pulanesaura,Gongxianosaurus)))))))))),(Mussaurus,Leonerasaurus)))),(Xingxiulong,(Jingshanosaurus,Chuxiongosaurus)))))),(Eucnemesaurus_entaxonis,(Riojasaurus,Eucnemesaurus_fortis)))),(Plateosaurus_gracilis,(Plateosaurus_ingens,Plateosaurus_engelhardti))))))))))))),(Buriolestes,Pampadromaeus))),(Staurikosaurus,Herrerasaurus))))))));

(Euparkeria,(Crurotarsi,(Marasuchus,(Silesaurus,(Agnosphitys,(Ornithischia,(((Guaibasaurus,(Neotheropoda,Chindesaurus)),((Panphagia,(Eoraptor,((Saturnalia,Chromogisaurus),(Bagualosaurus,(Jaklapalisaurus,(Nambalia,((Thecodontosaurus,Pantydraco),(Efraasia,(Plateosauravus,(Ruehleia,(((Unaysaurus,(Pradhania,Macrocollum)),((Sarahsaurus,((Ngwevu,(Xixipiosaurus,((Coloradisaurus,(Glacialisaurus,Lufengosaurus)),(Massospondylus_carinatus,(Adeopapposaurus,Leyesaurus))))),(Yunnanosaurus_huangi,((Seitaad,(Anchisaurus,((Irisosaurus,(Sefapanosaurus,(Yizhousaurus,(Aardonyx,(NMQR1551,(NMRQ3314,(Blikanasaurus,((Camelotia,((Meroktenos,(Ledumahadi,Kholumolumo)),(Lessemsaurus,(Ingentia,Antetonitrus)))),(Pulanesaura,(Gongxianosaurus,(Schleitheimia,(Isanosaurus,((Tazoudasaurus,Vulcanodon),((Shunosaurus,((Spinophorosaurus,Omeisaurus),(Mamenchisaurus,(Cetiosaurus,Neosauropoda)))),(Patagosaurus,Barapasaurus))))))))))))))),(Mussaurus,Leonerasaurus)))),(Xingxiulong,(Jingshanosaurus,Chuxiongosaurus)))))),(Eucnemesaurus_entaxonis,(Riojasaurus,Eucnemesaurus_fortis)))),(Plateosaurus_gracilis,(Plateosaurus_ingens,Plateosaurus_engelhardti))))))))))))),(Buriolestes,Pampadromaeus))),(Staurikosaurus,Herrerasaurus))))))));

(Euparkeria,(Crurotarsi,(Marasuchus,(Silesaurus,(Agnosphitys,(Ornithischia,(((Guaibasaurus,(Neotheropoda,Chindesaurus)),(Buriolestes,(Pampadromaeus,(Panphagia,(Eoraptor,((Saturnalia,Chromogisaurus),(Bagualosaurus,(Jaklapalisaurus,(Nambalia,((Thecodontosaurus,Pantydraco),(Efraasia,(Plateosauravus,(Ruehleia,(((Unaysaurus,(Pradhania,Macrocollum)),((Sarahsaurus,((Ngwevu,(Xixipiosaurus,((Coloradisaurus,(Glacialisaurus,Lufengosaurus)),(Massospondylus_carinatus,(Adeopapposaurus,Leyesaurus))))),(Yunnanosaurus_huangi,((Seitaad,(Anchisaurus,((Irisosaurus,(Sefapanosaurus,(Yizhousaurus,(Aardonyx,(NMQR1551,(NMRQ3314,(Blikanasaurus,((Camelotia,((Meroktenos,(Ledumahadi,Kholumolumo)),(Lessemsaurus,(Ingentia,Antetonitrus)))),(Pulanesaura,(Gongxianosaurus,(Schleitheimia,(Isanosaurus,((Tazoudasaurus,Vulcanodon),((Shunosaurus,((Spinophorosaurus,(Omeisaurus,Mamenchisaurus)),(Cetiosaurus,Neosauropoda))),(Patagosaurus,Barapasaurus))))))))))))))),(Mussaurus,Leonerasaurus)))),(Xingxiulong,(Jingshanosaurus,Chuxiongosaurus)))))),(Eucnemesaurus_entaxonis,(Riojasaurus,Eucnemesaurus_fortis)))),(Plateosaurus_gracilis,(Plateosaurus_ingens,Plateosaurus_engelhardti)))))))))))))))),(Staurikosaurus,Herrerasaurus))))))));

(Euparkeria,(Crurotarsi,(Marasuchus,(Silesaurus,(Agnosphitys,(Ornithischia,(((Guaibasaurus,(Neotheropoda,Chindesaurus)),((Panphagia,(Eoraptor,((Saturnalia,Chromogisaurus),(Bagualosaurus,(Jaklapalisaurus,(Nambalia,((Thecodontosaurus,Pantydraco),(Efraasia,(Plateosauravus,(Ruehleia,(((Unaysaurus,(Pradhania,Macrocollum)),((Sarahsaurus,((Ngwevu,(Xixipiosaurus,((Coloradisaurus,(Glacialisaurus,Lufengosaurus)),(Massospondylus_carinatus,(Adeopapposaurus,Leyesaurus))))),(Yunnanosaurus_huangi,((Seitaad,(Anchisaurus,((Irisosaurus,(Sefapanosaurus,(Yizhousaurus,(Aardonyx,(NMQR1551,(NMRQ3314,(Blikanasaurus,((Camelotia,((Meroktenos,(Ledumahadi,Kholumolumo)),(Lessemsaurus,(Ingentia,Antetonitrus)))),(Pulanesaura,(Gongxianosaurus,(Schleitheimia,(Isanosaurus,((Tazoudasaurus,Vulcanodon),((Shunosaurus,(Spinophorosaurus,(Mamenchisaurus,(Omeisaurus,(Cetiosaurus,Neosauropoda))))),(Patagosaurus,Barapasaurus))))))))))))))),(Mussaurus,Leonerasaurus)))),(Xingxiulong,(Jingshanosaurus,Chuxiongosaurus)))))),(Eucnemesaurus_entaxonis,(Riojasaurus,Eucnemesaurus_fortis)))),(Plateosaurus_gracilis,(Plateosaurus_ingens,Plateosaurus_engelhardti))))))))))))),(Buriolestes,Pampadromaeus))),(Staurikosaurus,Herrerasaurus))))))));

(Euparkeria,(Crurotarsi,(Marasuchus,(Silesaurus,(Agnosphitys,(Ornithischia,(((Guaibasaurus,(Neotheropoda,Chindesaurus)),((Panphagia,(Eoraptor,((Saturnalia,Chromogisaurus),(Bagualosaurus,(Jaklapalisaurus,(Nambalia,((Thecodontosaurus,Pantydraco),(Efraasia,(Plateosauravus,(Ruehleia,(((Unaysaurus,(Pradhania,Macrocollum)),((Sarahsaurus,((Ngwevu,(Xixipiosaurus,((Coloradisaurus,(Glacialisaurus,Lufengosaurus)),(Massospondylus_carinatus,(Adeopapposaurus,Leyesaurus))))),(Yunnanosaurus_huangi,((Seitaad,(Anchisaurus,((Irisosaurus,(Sefapanosaurus,(Yizhousaurus,(Aardonyx,(NMQR1551,(NMRQ3314,(Blikanasaurus,((Camelotia,((Meroktenos,(Ledumahadi,Kholumolumo)),(Lessemsaurus,(Ingentia,Antetonitrus)))),(Pulanesaura,(Gongxianosaurus,(Schleitheimia,(Isanosaurus,((Tazoudasaurus,Vulcanodon),((Shunosaurus,(Spinophorosaurus,(Omeisaurus,(Mamenchisaurus,(Cetiosaurus,Neosauropoda))))),(Patagosaurus,Barapasaurus))))))))))))))),(Mussaurus,Leonerasaurus)))),(Xingxiulong,(Jingshanosaurus,Chuxiongosaurus)))))),(Eucnemesaurus_entaxonis,(Riojasaurus,Eucnemesaurus_fortis)))),(Plateosaurus_gracilis,(Plateosaurus_ingens,Plateosaurus_engelhardti))))))))))))),(Buriolestes,Pampadromaeus))),(Staurikosaurus,Herrerasaurus))))))));

(Euparkeria,(Crurotarsi,(Marasuchus,(Silesaurus,(Ornithischia,(Agnosphitys,(((Guaibasaurus,(Neotheropoda,Chindesaurus)),((Panphagia,(Eoraptor,((Saturnalia,Chromogisaurus),(Bagualosaurus,(Jaklapalisaurus,(Nambalia,((Thecodontosaurus,Pantydraco),(Efraasia,(Plateosauravus,(Ruehleia,(((Unaysaurus,(Pradhania,Macrocollum)),((Sarahsaurus,((Ngwevu,(Xixipiosaurus,((Coloradisaurus,(Glacialisaurus,Lufengosaurus)),(Massospondylus_carinatus,(Adeopapposaurus,Leyesaurus))))),(Yunnanosaurus_huangi,((Seitaad,(Anchisaurus,((Irisosaurus,(Sefapanosaurus,(Yizhousaurus,(Aardonyx,(NMQR1551,(NMRQ3314,(Blikanasaurus,((Camelotia,((Meroktenos,(Ledumahadi,Kholumolumo)),(Lessemsaurus,(Ingentia,Antetonitrus)))),(Pulanesaura,(Gongxianosaurus,(Schleitheimia,(Isanosaurus,((Tazoudasaurus,Vulcanodon),((Shunosaurus,((Spinophorosaurus,(Omeisaurus,Mamenchisaurus)),(Cetiosaurus,Neosauropoda))),(Patagosaurus,Barapasaurus))))))))))))))),(Mussaurus,Leonerasaurus)))),(Xingxiulong,(Jingshanosaurus,Chuxiongosaurus)))))),(Eucnemesaurus_entaxonis,(Riojasaurus,Eucnemesaurus_fortis)))),(Plateosaurus_gracilis,(Plateosaurus_ingens,Plateosaurus_engelhardti))))))))))))),(Buriolestes,Pampadromaeus))),(Staurikosaurus,Herrerasaurus))))))));

(Euparkeria,(Crurotarsi,(Marasuchus,(Silesaurus,(Ornithischia,((Agnosphitys,(Staurikosaurus,Herrerasaurus)),((Guaibasaurus,(Neotheropoda,Chindesaurus)),((Panphagia,(Eoraptor,((Saturnalia,Chromogisaurus),(Bagualosaurus,(Jaklapalisaurus,(Nambalia,((Thecodontosaurus,Pantydraco),(Efraasia,(Plateosauravus,(Ruehleia,(((Unaysaurus,(Pradhania,Macrocollum)),((Sarahsaurus,((Ngwevu,(Xixipiosaurus,((Coloradisaurus,(Glacialisaurus,Lufengosaurus)),(Massospondylus_carinatus,(Adeopapposaurus,Leyesaurus))))),(Yunnanosaurus_huangi,((Seitaad,(Anchisaurus,((Irisosaurus,(Sefapanosaurus,(Yizhousaurus,(Aardonyx,(NMQR1551,(NMRQ3314,(Blikanasaurus,((Camelotia,((Meroktenos,(Ledumahadi,Kholumolumo)),(Lessemsaurus,(Ingentia,Antetonitrus)))),(Pulanesaura,(Gongxianosaurus,(Schleitheimia,(Isanosaurus,((Tazoudasaurus,Vulcanodon),((Shunosaurus,((Spinophorosaurus,(Omeisaurus,Mamenchisaurus)),(Cetiosaurus,Neosauropoda))),(Patagosaurus,Barapasaurus))))))))))))))),(Mussaurus,Leonerasaurus)))),(Xingxiulong,(Jingshanosaurus,Chuxiongosaurus)))))),(Eucnemesaurus_entaxonis,(Riojasaurus,Eucnemesaurus_fortis)))),(Plateosaurus_gracilis,(Plateosaurus_ingens,Plateosaurus_engelhardti))))))))))))),(Buriolestes,Pampadromaeus)))))))));

(Euparkeria,(Crurotarsi,(Marasuchus,(Silesaurus,(Agnosphitys,(Ornithischia,(((Guaibasaurus,(Neotheropoda,Chindesaurus)),((Panphagia,(Eoraptor,((Saturnalia,Chromogisaurus),(Bagualosaurus,(Jaklapalisaurus,(Nambalia,((Thecodontosaurus,Pantydraco),(Efraasia,(Plateosauravus,(Ruehleia,(((Unaysaurus,(Pradhania,Macrocollum)),((Sarahsaurus,((Ngwevu,((Coloradisaurus,(Glacialisaurus,Lufengosaurus)),(Xixipiosaurus,(Massospondylus_carinatus,(Adeopapposaurus,Leyesaurus))))),(Yunnanosaurus_huangi,((Seitaad,(Anchisaurus,((Irisosaurus,(Sefapanosaurus,(Yizhousaurus,(Aardonyx,(NMQR1551,(NMRQ3314,(Blikanasaurus,((Camelotia,((Meroktenos,(Ledumahadi,Kholumolumo)),(Lessemsaurus,(Ingentia,Antetonitrus)))),(Pulanesaura,(Gongxianosaurus,(Schleitheimia,(Isanosaurus,((Tazoudasaurus,Vulcanodon),((Shunosaurus,((Spinophorosaurus,(Omeisaurus,Mamenchisaurus)),(Cetiosaurus,Neosauropoda))),(Patagosaurus,Barapasaurus))))))))))))))),(Mussaurus,Leonerasaurus)))),(Xingxiulong,(Jingshanosaurus,Chuxiongosaurus)))))),(Eucnemesaurus_entaxonis,(Riojasaurus,Eucnemesaurus_fortis)))),(Plateosaurus_gracilis,(Plateosaurus_ingens,Plateosaurus_engelhardti))))))))))))),(Buriolestes,Pampadromaeus))),(Staurikosaurus,Herrerasaurus))))))));

(Euparkeria,(Crurotarsi,(Marasuchus,(Silesaurus,(Ornithischia,((Agnosphitys,((Guaibasaurus,(Neotheropoda,Chindesaurus)),(Buriolestes,(Panphagia,(Eoraptor,((Pampadromaeus,(Saturnalia,Chromogisaurus)),(Bagualosaurus,(Jaklapalisaurus,(Nambalia,((Thecodontosaurus,Pantydraco),(Efraasia,(Plateosauravus,(Ruehleia,(((Unaysaurus,(Pradhania,Macrocollum)),((Sarahsaurus,((Ngwevu,((Coloradisaurus,(Glacialisaurus,Lufengosaurus)),(Xixipiosaurus,(Massospondylus_carinatus,(Adeopapposaurus,Leyesaurus))))),(Yunnanosaurus_huangi,((Seitaad,(Anchisaurus,((Irisosaurus,(Sefapanosaurus,(Yizhousaurus,(Aardonyx,(NMQR1551,(NMRQ3314,(Blikanasaurus,((Camelotia,((Meroktenos,(Ledumahadi,Kholumolumo)),(Lessemsaurus,(Ingentia,Antetonitrus)))),((Schleitheimia,(Isanosaurus,((Tazoudasaurus,Vulcanodon),((Shunosaurus,((Spinophorosaurus,(Omeisaurus,Mamenchisaurus)),(Cetiosaurus,Neosauropoda))),(Patagosaurus,Barapasaurus))))),(Pulanesaura,Gongxianosaurus)))))))))),(Mussaurus,Leonerasaurus)))),(Xingxiulong,(Jingshanosaurus,Chuxiongosaurus)))))),(Eucnemesaurus_fortis,(Riojasaurus,Eucnemesaurus_entaxonis)))),(Plateosaurus_gracilis,(Plateosaurus_ingens,Plateosaurus_engelhardti)))))))))))))))),(Staurikosaurus,Herrerasaurus)))))));

(Euparkeria,(Crurotarsi,(Marasuchus,(Silesaurus,(Ornithischia,((Agnosphitys,((Guaibasaurus,(Neotheropoda,Chindesaurus)),(Buriolestes,(Pampadromaeus,(Panphagia,(Eoraptor,((Saturnalia,Chromogisaurus),(Bagualosaurus,(Jaklapalisaurus,(Nambalia,((Thecodontosaurus,Pantydraco),(Efraasia,(Plateosauravus,(Ruehleia,(((Unaysaurus,(Pradhania,Macrocollum)),((Sarahsaurus,((Ngwevu,(Xixipiosaurus,((Coloradisaurus,(Glacialisaurus,Lufengosaurus)),(Massospondylus_carinatus,(Adeopapposaurus,Leyesaurus))))),(Yunnanosaurus_huangi,((Seitaad,(Anchisaurus,((Irisosaurus,(Yizhousaurus,((Sefapanosaurus,Aardonyx),(NMQR1551,(NMRQ3314,(Blikanasaurus,((Camelotia,((Meroktenos,(Ledumahadi,Kholumolumo)),(Lessemsaurus,(Ingentia,Antetonitrus)))),((Schleitheimia,(Isanosaurus,((Tazoudasaurus,Vulcanodon),((Shunosaurus,((Spinophorosaurus,(Omeisaurus,Mamenchisaurus)),(Cetiosaurus,Neosauropoda))),(Patagosaurus,Barapasaurus))))),(Pulanesaura,Gongxianosaurus))))))))),(Mussaurus,Leonerasaurus)))),(Xingxiulong,(Jingshanosaurus,Chuxiongosaurus)))))),(Eucnemesaurus_fortis,(Riojasaurus,Eucnemesaurus_entaxonis)))),(Plateosaurus_gracilis,(Plateosaurus_ingens,Plateosaurus_engelhardti))))))))))))))))),(Staurikosaurus,Herrerasaurus)))))));

(Euparkeria,(Crurotarsi,(Marasuchus,(Silesaurus,(Ornithischia,((Agnosphitys,((Guaibasaurus,(Neotheropoda,Chindesaurus)),(Buriolestes,((Eoraptor,((Saturnalia,Chromogisaurus),(Bagualosaurus,(Jaklapalisaurus,(Nambalia,((Thecodontosaurus,Pantydraco),(Efraasia,(Plateosauravus,(Ruehleia,(((Unaysaurus,(Pradhania,Macrocollum)),((Sarahsaurus,((Ngwevu,(Xixipiosaurus,((Coloradisaurus,(Glacialisaurus,Lufengosaurus)),(Massospondylus_carinatus,(Adeopapposaurus,Leyesaurus))))),(Yunnanosaurus_huangi,((Seitaad,(Anchisaurus,((Irisosaurus,(Yizhousaurus,((Sefapanosaurus,Aardonyx),(NMQR1551,(NMRQ3314,(Blikanasaurus,((Camelotia,((Meroktenos,(Ledumahadi,Kholumolumo)),(Lessemsaurus,(Ingentia,Antetonitrus)))),((Schleitheimia,(Isanosaurus,((Tazoudasaurus,Vulcanodon),((Shunosaurus,((Spinophorosaurus,(Omeisaurus,Mamenchisaurus)),(Cetiosaurus,Neosauropoda))),(Patagosaurus,Barapasaurus))))),(Pulanesaura,Gongxianosaurus))))))))),(Mussaurus,Leonerasaurus)))),(Xingxiulong,(Jingshanosaurus,Chuxiongosaurus)))))),(Eucnemesaurus_fortis,(Riojasaurus,Eucnemesaurus_entaxonis)))),(Plateosaurus_gracilis,(Plateosaurus_ingens,Plateosaurus_engelhardti)))))))))))),(Panphagia,Pampadromaeus))))),(Staurikosaurus,Herrerasaurus)))))));

(Euparkeria,(Crurotarsi,(Marasuchus,(Silesaurus,(Ornithischia,((Agnosphitys,((Guaibasaurus,(Neotheropoda,Chindesaurus)),((Panphagia,(Eoraptor,((Saturnalia,Chromogisaurus),(Bagualosaurus,(Jaklapalisaurus,(Nambalia,((Thecodontosaurus,Pantydraco),(Efraasia,(Plateosauravus,(Ruehleia,(((Unaysaurus,(Pradhania,Macrocollum)),((Sarahsaurus,((Ngwevu,(Xixipiosaurus,((Coloradisaurus,(Glacialisaurus,Lufengosaurus)),(Massospondylus_carinatus,(Adeopapposaurus,Leyesaurus))))),(Yunnanosaurus_huangi,((Seitaad,(Anchisaurus,((Irisosaurus,(Yizhousaurus,((Sefapanosaurus,Aardonyx),(NMQR1551,(NMRQ3314,(Blikanasaurus,((Camelotia,((Meroktenos,(Ledumahadi,Kholumolumo)),(Lessemsaurus,(Ingentia,Antetonitrus)))),((Schleitheimia,(Isanosaurus,((Tazoudasaurus,Vulcanodon),((Shunosaurus,((Spinophorosaurus,(Omeisaurus,Mamenchisaurus)),(Cetiosaurus,Neosauropoda))),(Patagosaurus,Barapasaurus))))),(Pulanesaura,Gongxianosaurus))))))))),(Mussaurus,Leonerasaurus)))),(Xingxiulong,(Jingshanosaurus,Chuxiongosaurus)))))),(Eucnemesaurus_fortis,(Riojasaurus,Eucnemesaurus_entaxonis)))),(Plateosaurus_gracilis,(Plateosaurus_ingens,Plateosaurus_engelhardti))))))))))))),(Buriolestes,Pampadromaeus)))),(Staurikosaurus,Herrerasaurus)))))));

(Euparkeria,(Crurotarsi,(Marasuchus,(Silesaurus,(Ornithischia,((Agnosphitys,((Guaibasaurus,(Neotheropoda,Chindesaurus)),(Buriolestes,(Panphagia,(Eoraptor,((Pampadromaeus,(Saturnalia,Chromogisaurus)),(Bagualosaurus,(Jaklapalisaurus,(Nambalia,((Thecodontosaurus,Pantydraco),(Efraasia,(Plateosauravus,(Ruehleia,(((Unaysaurus,(Pradhania,Macrocollum)),((Sarahsaurus,((Ngwevu,((Coloradisaurus,(Glacialisaurus,Lufengosaurus)),(Xixipiosaurus,(Massospondylus_carinatus,(Adeopapposaurus,Leyesaurus))))),(Yunnanosaurus_huangi,((Seitaad,(Anchisaurus,((Irisosaurus,(Yizhousaurus,((Sefapanosaurus,Aardonyx),(NMQR1551,(NMRQ3314,(Blikanasaurus,((Camelotia,((Meroktenos,(Ledumahadi,Kholumolumo)),(Lessemsaurus,(Ingentia,Antetonitrus)))),((Schleitheimia,(Isanosaurus,((Tazoudasaurus,Vulcanodon),((Shunosaurus,((Spinophorosaurus,(Omeisaurus,Mamenchisaurus)),(Cetiosaurus,Neosauropoda))),(Patagosaurus,Barapasaurus))))),(Pulanesaura,Gongxianosaurus))))))))),(Mussaurus,Leonerasaurus)))),(Xingxiulong,(Jingshanosaurus,Chuxiongosaurus)))))),(Eucnemesaurus_fortis,(Riojasaurus,Eucnemesaurus_entaxonis)))),(Plateosaurus_gracilis,(Plateosaurus_ingens,Plateosaurus_engelhardti)))))))))))))))),(Staurikosaurus,Herrerasaurus)))))));

(Euparkeria,(Crurotarsi,(Marasuchus,(Silesaurus,(Ornithischia,((Agnosphitys,((Guaibasaurus,(Neotheropoda,Chindesaurus)),(Buriolestes,(Panphagia,(Eoraptor,((Pampadromaeus,(Saturnalia,Chromogisaurus)),(Bagualosaurus,(Jaklapalisaurus,(Nambalia,((Thecodontosaurus,Pantydraco),(Efraasia,(Plateosauravus,(Ruehleia,(((Unaysaurus,(Pradhania,Macrocollum)),((Sarahsaurus,((Ngwevu,(Xixipiosaurus,((Coloradisaurus,(Glacialisaurus,Lufengosaurus)),(Massospondylus_carinatus,(Adeopapposaurus,Leyesaurus))))),(Yunnanosaurus_huangi,((Seitaad,(Anchisaurus,((Yizhousaurus,((Sefapanosaurus,Aardonyx),(NMQR1551,(NMRQ3314,(Blikanasaurus,((Camelotia,((Meroktenos,(Ledumahadi,Kholumolumo)),(Lessemsaurus,(Ingentia,Antetonitrus)))),((Schleitheimia,(Isanosaurus,((Tazoudasaurus,Vulcanodon),((Shunosaurus,((Spinophorosaurus,(Omeisaurus,Mamenchisaurus)),(Cetiosaurus,Neosauropoda))),(Patagosaurus,Barapasaurus))))),(Pulanesaura,Gongxianosaurus)))))))),(Leonerasaurus,(Mussaurus,Irisosaurus))))),(Xingxiulong,(Jingshanosaurus,Chuxiongosaurus)))))),(Eucnemesaurus_fortis,(Riojasaurus,Eucnemesaurus_entaxonis)))),(Plateosaurus_gracilis,(Plateosaurus_ingens,Plateosaurus_engelhardti)))))))))))))))),(Staurikosaurus,Herrerasaurus)))))));

(Euparkeria,(Crurotarsi,(Marasuchus,(Silesaurus,(Ornithischia,((Agnosphitys,((Guaibasaurus,(Neotheropoda,Chindesaurus)),(Buriolestes,(Panphagia,(Eoraptor,((Pampadromaeus,(Saturnalia,Chromogisaurus)),(Bagualosaurus,(Jaklapalisaurus,(Nambalia,((Thecodontosaurus,Pantydraco),(Efraasia,(Plateosauravus,(Ruehleia,(((Unaysaurus,(Pradhania,Macrocollum)),((Sarahsaurus,((Ngwevu,(Xixipiosaurus,((Coloradisaurus,(Glacialisaurus,Lufengosaurus)),(Massospondylus_carinatus,(Adeopapposaurus,Leyesaurus))))),(Yunnanosaurus_huangi,((Seitaad,(Anchisaurus,((Irisosaurus,(Yizhousaurus,((Sefapanosaurus,Aardonyx),(NMQR1551,(NMRQ3314,(Blikanasaurus,((Camelotia,((Meroktenos,(Ledumahadi,Kholumolumo)),(Lessemsaurus,(Ingentia,Antetonitrus)))),(Pulanesaura,(Gongxianosaurus,(Schleitheimia,(Isanosaurus,((Tazoudasaurus,Vulcanodon),((Shunosaurus,((Spinophorosaurus,(Omeisaurus,Mamenchisaurus)),(Cetiosaurus,Neosauropoda))),(Patagosaurus,Barapasaurus)))))))))))))),(Mussaurus,Leonerasaurus)))),(Xingxiulong,(Jingshanosaurus,Chuxiongosaurus)))))),(Eucnemesaurus_fortis,(Riojasaurus,Eucnemesaurus_entaxonis)))),(Plateosaurus_gracilis,(Plateosaurus_ingens,Plateosaurus_engelhardti)))))))))))))))),(Staurikosaurus,Herrerasaurus)))))));

(Euparkeria,(Crurotarsi,(Marasuchus,(Silesaurus,(Ornithischia,((Agnosphitys,((Guaibasaurus,(Neotheropoda,Chindesaurus)),(Buriolestes,(Panphagia,(Eoraptor,((Pampadromaeus,(Saturnalia,Chromogisaurus)),(Bagualosaurus,(Jaklapalisaurus,(Nambalia,((Thecodontosaurus,Pantydraco),(Efraasia,(Plateosauravus,(Ruehleia,(((Unaysaurus,(Pradhania,Macrocollum)),((Sarahsaurus,((Ngwevu,(Xixipiosaurus,((Coloradisaurus,(Glacialisaurus,Lufengosaurus)),(Massospondylus_carinatus,(Adeopapposaurus,Leyesaurus))))),(Yunnanosaurus_huangi,((Seitaad,(Anchisaurus,((Irisosaurus,(Yizhousaurus,((Sefapanosaurus,Aardonyx),(NMQR1551,(NMRQ3314,(Blikanasaurus,((Camelotia,((Meroktenos,(Ledumahadi,Kholumolumo)),(Lessemsaurus,(Ingentia,Antetonitrus)))),((Schleitheimia,(Isanosaurus,((Tazoudasaurus,Vulcanodon),((Shunosaurus,((Spinophorosaurus,Omeisaurus),(Mamenchisaurus,(Cetiosaurus,Neosauropoda)))),(Patagosaurus,Barapasaurus))))),(Pulanesaura,Gongxianosaurus))))))))),(Mussaurus,Leonerasaurus)))),(Xingxiulong,(Jingshanosaurus,Chuxiongosaurus)))))),(Eucnemesaurus_fortis,(Riojasaurus,Eucnemesaurus_entaxonis)))),(Plateosaurus_gracilis,(Plateosaurus_ingens,Plateosaurus_engelhardti)))))))))))))))),(Staurikosaurus,Herrerasaurus)))))));

(Euparkeria,(Crurotarsi,(Marasuchus,(Silesaurus,(Ornithischia,((Agnosphitys,((Guaibasaurus,(Neotheropoda,Chindesaurus)),(Buriolestes,(Panphagia,(Eoraptor,((Saturnalia,Chromogisaurus),(Pampadromaeus,(Bagualosaurus,(Jaklapalisaurus,(Nambalia,((Thecodontosaurus,Pantydraco),(Efraasia,(Plateosauravus,(Ruehleia,(((Unaysaurus,(Pradhania,Macrocollum)),((Sarahsaurus,((Ngwevu,(Xixipiosaurus,((Coloradisaurus,(Glacialisaurus,Lufengosaurus)),(Massospondylus_carinatus,(Adeopapposaurus,Leyesaurus))))),(Yunnanosaurus_huangi,((Seitaad,(Anchisaurus,((Irisosaurus,(Yizhousaurus,((Sefapanosaurus,Aardonyx),(NMQR1551,(NMRQ3314,(Blikanasaurus,((Camelotia,((Meroktenos,(Ledumahadi,Kholumolumo)),(Lessemsaurus,(Ingentia,Antetonitrus)))),((Schleitheimia,(Isanosaurus,((Tazoudasaurus,Vulcanodon),((Shunosaurus,((Spinophorosaurus,(Omeisaurus,Mamenchisaurus)),(Cetiosaurus,Neosauropoda))),(Patagosaurus,Barapasaurus))))),(Pulanesaura,Gongxianosaurus))))))))),(Mussaurus,Leonerasaurus)))),(Xingxiulong,(Jingshanosaurus,Chuxiongosaurus)))))),(Eucnemesaurus_fortis,(Riojasaurus,Eucnemesaurus_entaxonis)))),(Plateosaurus_gracilis,(Plateosaurus_ingens,Plateosaurus_engelhardti))))))))))))))))),(Staurikosaurus,Herrerasaurus)))))));

(Euparkeria,(Crurotarsi,(Marasuchus,(Silesaurus,(Ornithischia,((Agnosphitys,((Guaibasaurus,(Neotheropoda,Chindesaurus)),(Buriolestes,(Panphagia,(Eoraptor,((Pampadromaeus,(Saturnalia,Chromogisaurus)),(Bagualosaurus,(Jaklapalisaurus,(Nambalia,((Thecodontosaurus,Pantydraco),(Efraasia,(Plateosauravus,(Ruehleia,(((Unaysaurus,(Pradhania,Macrocollum)),((Sarahsaurus,((Ngwevu,(Xixipiosaurus,((Coloradisaurus,(Glacialisaurus,Lufengosaurus)),(Massospondylus_carinatus,(Adeopapposaurus,Leyesaurus))))),(Yunnanosaurus_huangi,((Seitaad,(Anchisaurus,((Irisosaurus,(Yizhousaurus,((Sefapanosaurus,Aardonyx),(NMQR1551,(NMRQ3314,(Blikanasaurus,((Camelotia,((Meroktenos,(Ledumahadi,Kholumolumo)),(Lessemsaurus,(Ingentia,Antetonitrus)))),((Schleitheimia,(Isanosaurus,((Tazoudasaurus,Vulcanodon),((Shunosaurus,(Spinophorosaurus,(Mamenchisaurus,(Omeisaurus,(Cetiosaurus,Neosauropoda))))),(Patagosaurus,Barapasaurus))))),(Pulanesaura,Gongxianosaurus))))))))),(Mussaurus,Leonerasaurus)))),(Xingxiulong,(Jingshanosaurus,Chuxiongosaurus)))))),(Eucnemesaurus_fortis,(Riojasaurus,Eucnemesaurus_entaxonis)))),(Plateosaurus_gracilis,(Plateosaurus_ingens,Plateosaurus_engelhardti)))))))))))))))),(Staurikosaurus,Herrerasaurus)))))));

(Euparkeria,(Crurotarsi,(Marasuchus,(Silesaurus,(Ornithischia,((Agnosphitys,((Guaibasaurus,(Neotheropoda,Chindesaurus)),(Buriolestes,(Panphagia,(Eoraptor,((Pampadromaeus,(Saturnalia,Chromogisaurus)),(Bagualosaurus,(Jaklapalisaurus,(Nambalia,((Thecodontosaurus,Pantydraco),(Efraasia,(Plateosauravus,(Ruehleia,(((Unaysaurus,(Pradhania,Macrocollum)),((Sarahsaurus,((Ngwevu,(Xixipiosaurus,((Coloradisaurus,(Glacialisaurus,Lufengosaurus)),(Massospondylus_carinatus,(Adeopapposaurus,Leyesaurus))))),(Yunnanosaurus_huangi,((Seitaad,(Anchisaurus,((Irisosaurus,(Yizhousaurus,((Sefapanosaurus,Aardonyx),(NMQR1551,(NMRQ3314,(Blikanasaurus,((Camelotia,((Meroktenos,(Ledumahadi,Kholumolumo)),(Lessemsaurus,(Ingentia,Antetonitrus)))),((Schleitheimia,(Isanosaurus,((Tazoudasaurus,Vulcanodon),((Shunosaurus,(Spinophorosaurus,(Omeisaurus,(Mamenchisaurus,(Cetiosaurus,Neosauropoda))))),(Patagosaurus,Barapasaurus))))),(Pulanesaura,Gongxianosaurus))))))))),(Mussaurus,Leonerasaurus)))),(Xingxiulong,(Jingshanosaurus,Chuxiongosaurus)))))),(Eucnemesaurus_fortis,(Riojasaurus,Eucnemesaurus_entaxonis)))),(Plateosaurus_gracilis,(Plateosaurus_ingens,Plateosaurus_engelhardti)))))))))))))))),(Staurikosaurus,Herrerasaurus)))))));

(Euparkeria,(Crurotarsi,(Marasuchus,(Silesaurus,(Ornithischia,((Agnosphitys,((Guaibasaurus,(Neotheropoda,Chindesaurus)),(Buriolestes,(Pampadromaeus,(Panphagia,(Eoraptor,((Saturnalia,Chromogisaurus),(Bagualosaurus,(Jaklapalisaurus,(Nambalia,((Thecodontosaurus,Pantydraco),(Efraasia,(Plateosauravus,(Ruehleia,(((Unaysaurus,(Pradhania,Macrocollum)),((Sarahsaurus,((Ngwevu,(Xixipiosaurus,((Coloradisaurus,(Glacialisaurus,Lufengosaurus)),(Massospondylus_carinatus,(Adeopapposaurus,Leyesaurus))))),(Yunnanosaurus_huangi,((Seitaad,(Anchisaurus,((Irisosaurus,(Sefapanosaurus,(Yizhousaurus,(Aardonyx,(NMQR1551,(NMRQ3314,(Blikanasaurus,((Camelotia,((Meroktenos,(Ledumahadi,Kholumolumo)),(Lessemsaurus,(Ingentia,Antetonitrus)))),(Pulanesaura,(Gongxianosaurus,(Schleitheimia,(Isanosaurus,((Tazoudasaurus,Vulcanodon),((Shunosaurus,((Spinophorosaurus,(Omeisaurus,Mamenchisaurus)),(Cetiosaurus,Neosauropoda))),(Patagosaurus,Barapasaurus))))))))))))))),(Mussaurus,Leonerasaurus)))),(Xingxiulong,(Jingshanosaurus,Chuxiongosaurus)))))),(Eucnemesaurus_fortis,(Riojasaurus,Eucnemesaurus_entaxonis)))),(Plateosaurus_gracilis,(Plateosaurus_ingens,Plateosaurus_engelhardti))))))))))))))))),(Staurikosaurus,Herrerasaurus)))))));

(Euparkeria,(Crurotarsi,(Marasuchus,(Silesaurus,(Ornithischia,((Agnosphitys,((Guaibasaurus,(Neotheropoda,Chindesaurus)),(Buriolestes,((Eoraptor,((Saturnalia,Chromogisaurus),(Bagualosaurus,(Jaklapalisaurus,(Nambalia,((Thecodontosaurus,Pantydraco),(Efraasia,(Plateosauravus,(Ruehleia,(((Unaysaurus,(Pradhania,Macrocollum)),((Sarahsaurus,((Ngwevu,(Xixipiosaurus,((Coloradisaurus,(Glacialisaurus,Lufengosaurus)),(Massospondylus_carinatus,(Adeopapposaurus,Leyesaurus))))),(Yunnanosaurus_huangi,((Seitaad,(Anchisaurus,((Irisosaurus,(Sefapanosaurus,(Yizhousaurus,(Aardonyx,(NMQR1551,(NMRQ3314,(Blikanasaurus,((Camelotia,((Meroktenos,(Ledumahadi,Kholumolumo)),(Lessemsaurus,(Ingentia,Antetonitrus)))),(Pulanesaura,(Gongxianosaurus,(Schleitheimia,(Isanosaurus,((Tazoudasaurus,Vulcanodon),((Shunosaurus,((Spinophorosaurus,(Omeisaurus,Mamenchisaurus)),(Cetiosaurus,Neosauropoda))),(Patagosaurus,Barapasaurus))))))))))))))),(Mussaurus,Leonerasaurus)))),(Xingxiulong,(Jingshanosaurus,Chuxiongosaurus)))))),(Eucnemesaurus_fortis,(Riojasaurus,Eucnemesaurus_entaxonis)))),(Plateosaurus_gracilis,(Plateosaurus_ingens,Plateosaurus_engelhardti)))))))))))),(Panphagia,Pampadromaeus))))),(Staurikosaurus,Herrerasaurus)))))));

(Euparkeria,(Crurotarsi,(Marasuchus,(Silesaurus,(Ornithischia,((Agnosphitys,((Guaibasaurus,(Neotheropoda,Chindesaurus)),(Buriolestes,(Panphagia,(Eoraptor,((Pampadromaeus,(Saturnalia,Chromogisaurus)),(Bagualosaurus,(Jaklapalisaurus,(Nambalia,((Thecodontosaurus,Pantydraco),(Efraasia,(Plateosauravus,(Ruehleia,(((Unaysaurus,(Pradhania,Macrocollum)),((Sarahsaurus,((Ngwevu,((Coloradisaurus,(Glacialisaurus,Lufengosaurus)),(Xixipiosaurus,(Massospondylus_carinatus,(Adeopapposaurus,Leyesaurus))))),(Yunnanosaurus_huangi,((Seitaad,(Anchisaurus,((Irisosaurus,(Sefapanosaurus,(Yizhousaurus,(Aardonyx,(NMQR1551,(NMRQ3314,(Blikanasaurus,((Camelotia,((Meroktenos,(Ledumahadi,Kholumolumo)),(Lessemsaurus,(Ingentia,Antetonitrus)))),(Pulanesaura,(Gongxianosaurus,(Schleitheimia,(Isanosaurus,((Tazoudasaurus,Vulcanodon),((Shunosaurus,((Spinophorosaurus,(Omeisaurus,Mamenchisaurus)),(Cetiosaurus,Neosauropoda))),(Patagosaurus,Barapasaurus))))))))))))))),(Mussaurus,Leonerasaurus)))),(Xingxiulong,(Jingshanosaurus,Chuxiongosaurus)))))),(Eucnemesaurus_fortis,(Riojasaurus,Eucnemesaurus_entaxonis)))),(Plateosaurus_gracilis,(Plateosaurus_ingens,Plateosaurus_engelhardti)))))))))))))))),(Staurikosaurus,Herrerasaurus)))))));

(Euparkeria,(Crurotarsi,(Marasuchus,(Silesaurus,(Ornithischia,((Agnosphitys,((Guaibasaurus,(Neotheropoda,Chindesaurus)),(Buriolestes,(Panphagia,(Eoraptor,((Pampadromaeus,(Saturnalia,Chromogisaurus)),(Bagualosaurus,(Jaklapalisaurus,(Nambalia,((Thecodontosaurus,Pantydraco),(Efraasia,(Plateosauravus,(Ruehleia,(((Unaysaurus,(Pradhania,Macrocollum)),((Sarahsaurus,((Ngwevu,(Xixipiosaurus,((Coloradisaurus,(Glacialisaurus,Lufengosaurus)),(Massospondylus_carinatus,(Adeopapposaurus,Leyesaurus))))),(Yunnanosaurus_huangi,((Seitaad,(Anchisaurus,((Irisosaurus,(Sefapanosaurus,(Yizhousaurus,(Aardonyx,(NMQR1551,(NMRQ3314,(Blikanasaurus,((Camelotia,((Meroktenos,(Ledumahadi,Kholumolumo)),(Lessemsaurus,(Ingentia,Antetonitrus)))),(Pulanesaura,(Gongxianosaurus,(Schleitheimia,(Isanosaurus,((Tazoudasaurus,Vulcanodon),((Shunosaurus,((Spinophorosaurus,Omeisaurus),(Mamenchisaurus,(Cetiosaurus,Neosauropoda)))),(Patagosaurus,Barapasaurus))))))))))))))),(Mussaurus,Leonerasaurus)))),(Xingxiulong,(Jingshanosaurus,Chuxiongosaurus)))))),(Eucnemesaurus_fortis,(Riojasaurus,Eucnemesaurus_entaxonis)))),(Plateosaurus_gracilis,(Plateosaurus_ingens,Plateosaurus_engelhardti)))))))))))))))),(Staurikosaurus,Herrerasaurus)))))));

(Euparkeria,(Crurotarsi,(Marasuchus,(Silesaurus,(Ornithischia,((Agnosphitys,((Guaibasaurus,(Neotheropoda,Chindesaurus)),(Buriolestes,(Panphagia,(Eoraptor,((Saturnalia,Chromogisaurus),(Pampadromaeus,(Bagualosaurus,(Jaklapalisaurus,(Nambalia,((Thecodontosaurus,Pantydraco),(Efraasia,(Plateosauravus,(Ruehleia,(((Unaysaurus,(Pradhania,Macrocollum)),((Sarahsaurus,((Ngwevu,(Xixipiosaurus,((Coloradisaurus,(Glacialisaurus,Lufengosaurus)),(Massospondylus_carinatus,(Adeopapposaurus,Leyesaurus))))),(Yunnanosaurus_huangi,((Seitaad,(Anchisaurus,((Irisosaurus,(Sefapanosaurus,(Yizhousaurus,(Aardonyx,(NMQR1551,(NMRQ3314,(Blikanasaurus,((Camelotia,((Meroktenos,(Ledumahadi,Kholumolumo)),(Lessemsaurus,(Ingentia,Antetonitrus)))),(Pulanesaura,(Gongxianosaurus,(Schleitheimia,(Isanosaurus,((Tazoudasaurus,Vulcanodon),((Shunosaurus,((Spinophorosaurus,(Omeisaurus,Mamenchisaurus)),(Cetiosaurus,Neosauropoda))),(Patagosaurus,Barapasaurus))))))))))))))),(Mussaurus,Leonerasaurus)))),(Xingxiulong,(Jingshanosaurus,Chuxiongosaurus)))))),(Eucnemesaurus_fortis,(Riojasaurus,Eucnemesaurus_entaxonis)))),(Plateosaurus_gracilis,(Plateosaurus_ingens,Plateosaurus_engelhardti))))))))))))))))),(Staurikosaurus,Herrerasaurus)))))));

(Euparkeria,(Crurotarsi,(Marasuchus,(Silesaurus,(Ornithischia,((Agnosphitys,((Guaibasaurus,(Neotheropoda,Chindesaurus)),(Buriolestes,(Panphagia,(Eoraptor,((Pampadromaeus,(Saturnalia,Chromogisaurus)),(Bagualosaurus,(Jaklapalisaurus,(Nambalia,((Thecodontosaurus,Pantydraco),(Efraasia,(Plateosauravus,(Ruehleia,(((Unaysaurus,(Pradhania,Macrocollum)),((Sarahsaurus,((Ngwevu,(Xixipiosaurus,((Coloradisaurus,(Glacialisaurus,Lufengosaurus)),(Massospondylus_carinatus,(Adeopapposaurus,Leyesaurus))))),(Yunnanosaurus_huangi,((Seitaad,(Anchisaurus,((Irisosaurus,(Sefapanosaurus,(Yizhousaurus,(Aardonyx,(NMQR1551,(NMRQ3314,(Blikanasaurus,((Camelotia,((Meroktenos,(Ledumahadi,Kholumolumo)),(Lessemsaurus,(Ingentia,Antetonitrus)))),(Pulanesaura,(Gongxianosaurus,(Schleitheimia,(Isanosaurus,((Tazoudasaurus,Vulcanodon),((Shunosaurus,(Spinophorosaurus,(Mamenchisaurus,(Omeisaurus,(Cetiosaurus,Neosauropoda))))),(Patagosaurus,Barapasaurus))))))))))))))),(Mussaurus,Leonerasaurus)))),(Xingxiulong,(Jingshanosaurus,Chuxiongosaurus)))))),(Eucnemesaurus_fortis,(Riojasaurus,Eucnemesaurus_entaxonis)))),(Plateosaurus_gracilis,(Plateosaurus_ingens,Plateosaurus_engelhardti)))))))))))))))),(Staurikosaurus,Herrerasaurus)))))));

(Euparkeria,(Crurotarsi,(Marasuchus,(Silesaurus,(Ornithischia,((Agnosphitys,((Guaibasaurus,(Neotheropoda,Chindesaurus)),(Buriolestes,(Panphagia,(Eoraptor,((Pampadromaeus,(Saturnalia,Chromogisaurus)),(Bagualosaurus,(Jaklapalisaurus,(Nambalia,((Thecodontosaurus,Pantydraco),(Efraasia,(Plateosauravus,(Ruehleia,(((Unaysaurus,(Pradhania,Macrocollum)),((Sarahsaurus,((Ngwevu,(Xixipiosaurus,((Coloradisaurus,(Glacialisaurus,Lufengosaurus)),(Massospondylus_carinatus,(Adeopapposaurus,Leyesaurus))))),(Yunnanosaurus_huangi,((Seitaad,(Anchisaurus,((Irisosaurus,(Sefapanosaurus,(Yizhousaurus,(Aardonyx,(NMQR1551,(NMRQ3314,(Blikanasaurus,((Camelotia,((Meroktenos,(Ledumahadi,Kholumolumo)),(Lessemsaurus,(Ingentia,Antetonitrus)))),(Pulanesaura,(Gongxianosaurus,(Schleitheimia,(Isanosaurus,((Tazoudasaurus,Vulcanodon),((Shunosaurus,(Spinophorosaurus,(Omeisaurus,(Mamenchisaurus,(Cetiosaurus,Neosauropoda))))),(Patagosaurus,Barapasaurus))))))))))))))),(Mussaurus,Leonerasaurus)))),(Xingxiulong,(Jingshanosaurus,Chuxiongosaurus)))))),(Eucnemesaurus_fortis,(Riojasaurus,Eucnemesaurus_entaxonis)))),(Plateosaurus_gracilis,(Plateosaurus_ingens,Plateosaurus_engelhardti)))))))))))))))),(Staurikosaurus,Herrerasaurus)))))));

(Euparkeria,(Crurotarsi,(Marasuchus,(Silesaurus,(Ornithischia,((Agnosphitys,((Guaibasaurus,(Neotheropoda,Chindesaurus)),(Buriolestes,(Pampadromaeus,(Panphagia,(Eoraptor,((Saturnalia,Chromogisaurus),(Bagualosaurus,(Jaklapalisaurus,(Nambalia,((Thecodontosaurus,Pantydraco),(Efraasia,(Plateosauravus,(Ruehleia,(((Unaysaurus,(Pradhania,Macrocollum)),((Sarahsaurus,((Ngwevu,(Xixipiosaurus,((Coloradisaurus,(Glacialisaurus,Lufengosaurus)),(Massospondylus_carinatus,(Adeopapposaurus,Leyesaurus))))),(Yunnanosaurus_huangi,((Seitaad,(Anchisaurus,((Irisosaurus,(Sefapanosaurus,(Yizhousaurus,(Aardonyx,(NMQR1551,(NMRQ3314,(Blikanasaurus,((Camelotia,((Meroktenos,(Ledumahadi,Kholumolumo)),(Lessemsaurus,(Ingentia,Antetonitrus)))),((Schleitheimia,(Isanosaurus,((Tazoudasaurus,Vulcanodon),((Shunosaurus,((Spinophorosaurus,Omeisaurus),(Mamenchisaurus,(Cetiosaurus,Neosauropoda)))),(Patagosaurus,Barapasaurus))))),(Pulanesaura,Gongxianosaurus)))))))))),(Mussaurus,Leonerasaurus)))),(Xingxiulong,(Jingshanosaurus,Chuxiongosaurus)))))),(Eucnemesaurus_fortis,(Riojasaurus,Eucnemesaurus_entaxonis)))),(Plateosaurus_gracilis,(Plateosaurus_ingens,Plateosaurus_engelhardti))))))))))))))))),(Staurikosaurus,Herrerasaurus)))))));

(Euparkeria,(Crurotarsi,(Marasuchus,(Silesaurus,(Ornithischia,((Agnosphitys,((Guaibasaurus,(Neotheropoda,Chindesaurus)),(Buriolestes,((Eoraptor,((Saturnalia,Chromogisaurus),(Bagualosaurus,(Jaklapalisaurus,(Nambalia,((Thecodontosaurus,Pantydraco),(Efraasia,(Plateosauravus,(Ruehleia,(((Unaysaurus,(Pradhania,Macrocollum)),((Sarahsaurus,((Ngwevu,(Xixipiosaurus,((Coloradisaurus,(Glacialisaurus,Lufengosaurus)),(Massospondylus_carinatus,(Adeopapposaurus,Leyesaurus))))),(Yunnanosaurus_huangi,((Seitaad,(Anchisaurus,((Irisosaurus,(Sefapanosaurus,(Yizhousaurus,(Aardonyx,(NMQR1551,(NMRQ3314,(Blikanasaurus,((Camelotia,((Meroktenos,(Ledumahadi,Kholumolumo)),(Lessemsaurus,(Ingentia,Antetonitrus)))),((Schleitheimia,(Isanosaurus,((Tazoudasaurus,Vulcanodon),((Shunosaurus,((Spinophorosaurus,Omeisaurus),(Mamenchisaurus,(Cetiosaurus,Neosauropoda)))),(Patagosaurus,Barapasaurus))))),(Pulanesaura,Gongxianosaurus)))))))))),(Mussaurus,Leonerasaurus)))),(Xingxiulong,(Jingshanosaurus,Chuxiongosaurus)))))),(Eucnemesaurus_fortis,(Riojasaurus,Eucnemesaurus_entaxonis)))),(Plateosaurus_gracilis,(Plateosaurus_ingens,Plateosaurus_engelhardti)))))))))))),(Panphagia,Pampadromaeus))))),(Staurikosaurus,Herrerasaurus)))))));

(Euparkeria,(Crurotarsi,(Marasuchus,(Silesaurus,(Ornithischia,((Agnosphitys,((Guaibasaurus,(Neotheropoda,Chindesaurus)),((Panphagia,(Eoraptor,((Saturnalia,Chromogisaurus),(Bagualosaurus,(Jaklapalisaurus,(Nambalia,((Thecodontosaurus,Pantydraco),(Efraasia,(Plateosauravus,(Ruehleia,(((Unaysaurus,(Pradhania,Macrocollum)),((Sarahsaurus,((Ngwevu,(Xixipiosaurus,((Coloradisaurus,(Glacialisaurus,Lufengosaurus)),(Massospondylus_carinatus,(Adeopapposaurus,Leyesaurus))))),(Yunnanosaurus_huangi,((Seitaad,(Anchisaurus,((Irisosaurus,(Sefapanosaurus,(Yizhousaurus,(Aardonyx,(NMQR1551,(NMRQ3314,(Blikanasaurus,((Camelotia,((Meroktenos,(Ledumahadi,Kholumolumo)),(Lessemsaurus,(Ingentia,Antetonitrus)))),((Schleitheimia,(Isanosaurus,((Tazoudasaurus,Vulcanodon),((Shunosaurus,((Spinophorosaurus,Omeisaurus),(Mamenchisaurus,(Cetiosaurus,Neosauropoda)))),(Patagosaurus,Barapasaurus))))),(Pulanesaura,Gongxianosaurus)))))))))),(Mussaurus,Leonerasaurus)))),(Xingxiulong,(Jingshanosaurus,Chuxiongosaurus)))))),(Eucnemesaurus_fortis,(Riojasaurus,Eucnemesaurus_entaxonis)))),(Plateosaurus_gracilis,(Plateosaurus_ingens,Plateosaurus_engelhardti))))))))))))),(Buriolestes,Pampadromaeus)))),(Staurikosaurus,Herrerasaurus)))))));

(Euparkeria,(Crurotarsi,(Marasuchus,(Silesaurus,(Ornithischia,((Agnosphitys,((Guaibasaurus,(Neotheropoda,Chindesaurus)),(Buriolestes,(Panphagia,(Eoraptor,((Pampadromaeus,(Saturnalia,Chromogisaurus)),(Bagualosaurus,(Jaklapalisaurus,(Nambalia,((Thecodontosaurus,Pantydraco),(Efraasia,(Plateosauravus,(Ruehleia,(((Unaysaurus,(Pradhania,Macrocollum)),((Sarahsaurus,((Ngwevu,((Coloradisaurus,(Glacialisaurus,Lufengosaurus)),(Xixipiosaurus,(Massospondylus_carinatus,(Adeopapposaurus,Leyesaurus))))),(Yunnanosaurus_huangi,((Seitaad,(Anchisaurus,((Irisosaurus,(Sefapanosaurus,(Yizhousaurus,(Aardonyx,(NMQR1551,(NMRQ3314,(Blikanasaurus,((Camelotia,((Meroktenos,(Ledumahadi,Kholumolumo)),(Lessemsaurus,(Ingentia,Antetonitrus)))),((Schleitheimia,(Isanosaurus,((Tazoudasaurus,Vulcanodon),((Shunosaurus,((Spinophorosaurus,Omeisaurus),(Mamenchisaurus,(Cetiosaurus,Neosauropoda)))),(Patagosaurus,Barapasaurus))))),(Pulanesaura,Gongxianosaurus)))))))))),(Mussaurus,Leonerasaurus)))),(Xingxiulong,(Jingshanosaurus,Chuxiongosaurus)))))),(Eucnemesaurus_fortis,(Riojasaurus,Eucnemesaurus_entaxonis)))),(Plateosaurus_gracilis,(Plateosaurus_ingens,Plateosaurus_engelhardti)))))))))))))))),(Staurikosaurus,Herrerasaurus)))))));

(Euparkeria,(Crurotarsi,(Marasuchus,(Silesaurus,(Ornithischia,((Agnosphitys,((Guaibasaurus,(Neotheropoda,Chindesaurus)),(Buriolestes,(Panphagia,(Eoraptor,((Saturnalia,Chromogisaurus),(Pampadromaeus,(Bagualosaurus,(Jaklapalisaurus,(Nambalia,((Thecodontosaurus,Pantydraco),(Efraasia,(Plateosauravus,(Ruehleia,(((Unaysaurus,(Pradhania,Macrocollum)),((Sarahsaurus,((Ngwevu,(Xixipiosaurus,((Coloradisaurus,(Glacialisaurus,Lufengosaurus)),(Massospondylus_carinatus,(Adeopapposaurus,Leyesaurus))))),(Yunnanosaurus_huangi,((Seitaad,(Anchisaurus,((Irisosaurus,(Sefapanosaurus,(Yizhousaurus,(Aardonyx,(NMQR1551,(NMRQ3314,(Blikanasaurus,((Camelotia,((Meroktenos,(Ledumahadi,Kholumolumo)),(Lessemsaurus,(Ingentia,Antetonitrus)))),((Schleitheimia,(Isanosaurus,((Tazoudasaurus,Vulcanodon),((Shunosaurus,((Spinophorosaurus,Omeisaurus),(Mamenchisaurus,(Cetiosaurus,Neosauropoda)))),(Patagosaurus,Barapasaurus))))),(Pulanesaura,Gongxianosaurus)))))))))),(Mussaurus,Leonerasaurus)))),(Xingxiulong,(Jingshanosaurus,Chuxiongosaurus)))))),(Eucnemesaurus_fortis,(Riojasaurus,Eucnemesaurus_entaxonis)))),(Plateosaurus_gracilis,(Plateosaurus_ingens,Plateosaurus_engelhardti))))))))))))))))),(Staurikosaurus,Herrerasaurus)))))));

(Euparkeria,(Crurotarsi,(Marasuchus,(Silesaurus,(Ornithischia,((Agnosphitys,((Guaibasaurus,(Neotheropoda,Chindesaurus)),(Buriolestes,(Panphagia,(Eoraptor,((Saturnalia,Chromogisaurus),(Pampadromaeus,(Bagualosaurus,(Jaklapalisaurus,(Nambalia,((Thecodontosaurus,Pantydraco),(Efraasia,(Plateosauravus,(Ruehleia,(((Unaysaurus,(Pradhania,Macrocollum)),((Sarahsaurus,((Ngwevu,((Coloradisaurus,(Glacialisaurus,Lufengosaurus)),(Xixipiosaurus,(Massospondylus_carinatus,(Adeopapposaurus,Leyesaurus))))),(Yunnanosaurus_huangi,((Seitaad,(Anchisaurus,((Irisosaurus,(Sefapanosaurus,(Yizhousaurus,(Aardonyx,(NMQR1551,(NMRQ3314,(Blikanasaurus,((Camelotia,((Meroktenos,(Ledumahadi,Kholumolumo)),(Lessemsaurus,(Ingentia,Antetonitrus)))),((Schleitheimia,(Isanosaurus,((Tazoudasaurus,Vulcanodon),((Shunosaurus,((Spinophorosaurus,(Omeisaurus,Mamenchisaurus)),(Cetiosaurus,Neosauropoda))),(Patagosaurus,Barapasaurus))))),(Pulanesaura,Gongxianosaurus)))))))))),(Mussaurus,Leonerasaurus)))),(Xingxiulong,(Jingshanosaurus,Chuxiongosaurus)))))),(Eucnemesaurus_fortis,(Riojasaurus,Eucnemesaurus_entaxonis)))),(Plateosaurus_gracilis,(Plateosaurus_ingens,Plateosaurus_engelhardti))))))))))))))))),(Staurikosaurus,Herrerasaurus)))))));

(Euparkeria,(Crurotarsi,(Marasuchus,(Silesaurus,(Ornithischia,((Agnosphitys,((Guaibasaurus,(Neotheropoda,Chindesaurus)),(Buriolestes,(Panphagia,(Eoraptor,((Saturnalia,Chromogisaurus),(Pampadromaeus,(Bagualosaurus,(Jaklapalisaurus,(Nambalia,((Thecodontosaurus,Pantydraco),(Efraasia,(Plateosauravus,(Ruehleia,(((Unaysaurus,(Pradhania,Macrocollum)),((Sarahsaurus,((Ngwevu,(Xixipiosaurus,((Coloradisaurus,(Glacialisaurus,Lufengosaurus)),(Massospondylus_carinatus,(Adeopapposaurus,Leyesaurus))))),(Yunnanosaurus_huangi,((Seitaad,(Anchisaurus,((Irisosaurus,(Sefapanosaurus,(Yizhousaurus,(Aardonyx,(NMQR1551,(NMRQ3314,(Blikanasaurus,((Camelotia,((Meroktenos,(Ledumahadi,Kholumolumo)),(Lessemsaurus,(Ingentia,Antetonitrus)))),((Schleitheimia,(Isanosaurus,((Tazoudasaurus,Vulcanodon),((Shunosaurus,(Spinophorosaurus,(Mamenchisaurus,(Omeisaurus,(Cetiosaurus,Neosauropoda))))),(Patagosaurus,Barapasaurus))))),(Pulanesaura,Gongxianosaurus)))))))))),(Mussaurus,Leonerasaurus)))),(Xingxiulong,(Jingshanosaurus,Chuxiongosaurus)))))),(Eucnemesaurus_fortis,(Riojasaurus,Eucnemesaurus_entaxonis)))),(Plateosaurus_gracilis,(Plateosaurus_ingens,Plateosaurus_engelhardti))))))))))))))))),(Staurikosaurus,Herrerasaurus)))))));

(Euparkeria,(Crurotarsi,(Marasuchus,(Silesaurus,(Ornithischia,((Agnosphitys,((Guaibasaurus,(Neotheropoda,Chindesaurus)),(Buriolestes,(Panphagia,(Eoraptor,((Saturnalia,Chromogisaurus),(Pampadromaeus,(Bagualosaurus,(Jaklapalisaurus,(Nambalia,((Thecodontosaurus,Pantydraco),(Efraasia,(Plateosauravus,(Ruehleia,(((Unaysaurus,(Pradhania,Macrocollum)),((Sarahsaurus,((Ngwevu,(Xixipiosaurus,((Coloradisaurus,(Glacialisaurus,Lufengosaurus)),(Massospondylus_carinatus,(Adeopapposaurus,Leyesaurus))))),(Yunnanosaurus_huangi,((Seitaad,(Anchisaurus,((Irisosaurus,(Sefapanosaurus,(Yizhousaurus,(Aardonyx,(NMQR1551,(NMRQ3314,(Blikanasaurus,((Camelotia,((Meroktenos,(Ledumahadi,Kholumolumo)),(Lessemsaurus,(Ingentia,Antetonitrus)))),((Schleitheimia,(Isanosaurus,((Tazoudasaurus,Vulcanodon),((Shunosaurus,(Spinophorosaurus,(Omeisaurus,(Mamenchisaurus,(Cetiosaurus,Neosauropoda))))),(Patagosaurus,Barapasaurus))))),(Pulanesaura,Gongxianosaurus)))))))))),(Mussaurus,Leonerasaurus)))),(Xingxiulong,(Jingshanosaurus,Chuxiongosaurus)))))),(Eucnemesaurus_fortis,(Riojasaurus,Eucnemesaurus_entaxonis)))),(Plateosaurus_gracilis,(Plateosaurus_ingens,Plateosaurus_engelhardti))))))))))))))))),(Staurikosaurus,Herrerasaurus)))))));

(Euparkeria,(Crurotarsi,(Marasuchus,(Silesaurus,(Ornithischia,((Agnosphitys,((Guaibasaurus,(Neotheropoda,Chindesaurus)),(Buriolestes,(Pampadromaeus,(Panphagia,(Eoraptor,((Saturnalia,Chromogisaurus),(Bagualosaurus,(Jaklapalisaurus,(Nambalia,((Thecodontosaurus,Pantydraco),(Efraasia,(Plateosauravus,(Ruehleia,(((Unaysaurus,(Pradhania,Macrocollum)),((Sarahsaurus,((Ngwevu,(Xixipiosaurus,((Coloradisaurus,(Glacialisaurus,Lufengosaurus)),(Massospondylus_carinatus,(Adeopapposaurus,Leyesaurus))))),(Yunnanosaurus_huangi,((Seitaad,(Anchisaurus,((Irisosaurus,(Sefapanosaurus,(Yizhousaurus,(Aardonyx,(NMQR1551,(NMRQ3314,(Blikanasaurus,((Camelotia,((Meroktenos,(Ledumahadi,Kholumolumo)),(Lessemsaurus,(Ingentia,Antetonitrus)))),((Schleitheimia,(Isanosaurus,((Tazoudasaurus,Vulcanodon),((Shunosaurus,(Spinophorosaurus,(Mamenchisaurus,(Omeisaurus,(Cetiosaurus,Neosauropoda))))),(Patagosaurus,Barapasaurus))))),(Pulanesaura,Gongxianosaurus)))))))))),(Mussaurus,Leonerasaurus)))),(Xingxiulong,(Jingshanosaurus,Chuxiongosaurus)))))),(Eucnemesaurus_fortis,(Riojasaurus,Eucnemesaurus_entaxonis)))),(Plateosaurus_gracilis,(Plateosaurus_ingens,Plateosaurus_engelhardti))))))))))))))))),(Staurikosaurus,Herrerasaurus)))))));

(Euparkeria,(Crurotarsi,(Marasuchus,(Silesaurus,(Ornithischia,((Agnosphitys,((Guaibasaurus,(Neotheropoda,Chindesaurus)),(Buriolestes,((Eoraptor,((Saturnalia,Chromogisaurus),(Bagualosaurus,(Jaklapalisaurus,(Nambalia,((Thecodontosaurus,Pantydraco),(Efraasia,(Plateosauravus,(Ruehleia,(((Unaysaurus,(Pradhania,Macrocollum)),((Sarahsaurus,((Ngwevu,(Xixipiosaurus,((Coloradisaurus,(Glacialisaurus,Lufengosaurus)),(Massospondylus_carinatus,(Adeopapposaurus,Leyesaurus))))),(Yunnanosaurus_huangi,((Seitaad,(Anchisaurus,((Irisosaurus,(Sefapanosaurus,(Yizhousaurus,(Aardonyx,(NMQR1551,(NMRQ3314,(Blikanasaurus,((Camelotia,((Meroktenos,(Ledumahadi,Kholumolumo)),(Lessemsaurus,(Ingentia,Antetonitrus)))),((Schleitheimia,(Isanosaurus,((Tazoudasaurus,Vulcanodon),((Shunosaurus,(Spinophorosaurus,(Mamenchisaurus,(Omeisaurus,(Cetiosaurus,Neosauropoda))))),(Patagosaurus,Barapasaurus))))),(Pulanesaura,Gongxianosaurus)))))))))),(Mussaurus,Leonerasaurus)))),(Xingxiulong,(Jingshanosaurus,Chuxiongosaurus)))))),(Eucnemesaurus_fortis,(Riojasaurus,Eucnemesaurus_entaxonis)))),(Plateosaurus_gracilis,(Plateosaurus_ingens,Plateosaurus_engelhardti)))))))))))),(Panphagia,Pampadromaeus))))),(Staurikosaurus,Herrerasaurus)))))));

(Euparkeria,(Crurotarsi,(Marasuchus,(Silesaurus,(Ornithischia,((Agnosphitys,((Guaibasaurus,(Neotheropoda,Chindesaurus)),((Panphagia,(Eoraptor,((Saturnalia,Chromogisaurus),(Bagualosaurus,(Jaklapalisaurus,(Nambalia,((Thecodontosaurus,Pantydraco),(Efraasia,(Plateosauravus,(Ruehleia,(((Unaysaurus,(Pradhania,Macrocollum)),((Sarahsaurus,((Ngwevu,(Xixipiosaurus,((Coloradisaurus,(Glacialisaurus,Lufengosaurus)),(Massospondylus_carinatus,(Adeopapposaurus,Leyesaurus))))),(Yunnanosaurus_huangi,((Seitaad,(Anchisaurus,((Irisosaurus,(Sefapanosaurus,(Yizhousaurus,(Aardonyx,(NMQR1551,(NMRQ3314,(Blikanasaurus,((Camelotia,((Meroktenos,(Ledumahadi,Kholumolumo)),(Lessemsaurus,(Ingentia,Antetonitrus)))),((Schleitheimia,(Isanosaurus,((Tazoudasaurus,Vulcanodon),((Shunosaurus,(Spinophorosaurus,(Mamenchisaurus,(Omeisaurus,(Cetiosaurus,Neosauropoda))))),(Patagosaurus,Barapasaurus))))),(Pulanesaura,Gongxianosaurus)))))))))),(Mussaurus,Leonerasaurus)))),(Xingxiulong,(Jingshanosaurus,Chuxiongosaurus)))))),(Eucnemesaurus_fortis,(Riojasaurus,Eucnemesaurus_entaxonis)))),(Plateosaurus_gracilis,(Plateosaurus_ingens,Plateosaurus_engelhardti))))))))))))),(Buriolestes,Pampadromaeus)))),(Staurikosaurus,Herrerasaurus)))))));

(Euparkeria,(Crurotarsi,(Marasuchus,(Silesaurus,(Ornithischia,((Agnosphitys,((Guaibasaurus,(Neotheropoda,Chindesaurus)),(Buriolestes,(Panphagia,(Eoraptor,((Pampadromaeus,(Saturnalia,Chromogisaurus)),(Bagualosaurus,(Jaklapalisaurus,(Nambalia,((Thecodontosaurus,Pantydraco),(Efraasia,(Plateosauravus,(Ruehleia,(((Unaysaurus,(Pradhania,Macrocollum)),((Sarahsaurus,((Ngwevu,((Coloradisaurus,(Glacialisaurus,Lufengosaurus)),(Xixipiosaurus,(Massospondylus_carinatus,(Adeopapposaurus,Leyesaurus))))),(Yunnanosaurus_huangi,((Seitaad,(Anchisaurus,((Irisosaurus,(Sefapanosaurus,(Yizhousaurus,(Aardonyx,(NMQR1551,(NMRQ3314,(Blikanasaurus,((Camelotia,((Meroktenos,(Ledumahadi,Kholumolumo)),(Lessemsaurus,(Ingentia,Antetonitrus)))),((Schleitheimia,(Isanosaurus,((Tazoudasaurus,Vulcanodon),((Shunosaurus,(Spinophorosaurus,(Mamenchisaurus,(Omeisaurus,(Cetiosaurus,Neosauropoda))))),(Patagosaurus,Barapasaurus))))),(Pulanesaura,Gongxianosaurus)))))))))),(Mussaurus,Leonerasaurus)))),(Xingxiulong,(Jingshanosaurus,Chuxiongosaurus)))))),(Eucnemesaurus_fortis,(Riojasaurus,Eucnemesaurus_entaxonis)))),(Plateosaurus_gracilis,(Plateosaurus_ingens,Plateosaurus_engelhardti)))))))))))))))),(Staurikosaurus,Herrerasaurus)))))));

(Euparkeria,(Crurotarsi,(Marasuchus,(Silesaurus,(Ornithischia,((Agnosphitys,((Guaibasaurus,(Neotheropoda,Chindesaurus)),(Buriolestes,(Pampadromaeus,(Panphagia,(Eoraptor,((Saturnalia,Chromogisaurus),(Bagualosaurus,(Jaklapalisaurus,(Nambalia,((Thecodontosaurus,Pantydraco),(Efraasia,(Plateosauravus,(Ruehleia,(((Unaysaurus,(Pradhania,Macrocollum)),((Sarahsaurus,((Ngwevu,(Xixipiosaurus,((Coloradisaurus,(Glacialisaurus,Lufengosaurus)),(Massospondylus_carinatus,(Adeopapposaurus,Leyesaurus))))),(Yunnanosaurus_huangi,((Seitaad,(Anchisaurus,((Irisosaurus,(Sefapanosaurus,(Yizhousaurus,(Aardonyx,(NMQR1551,(NMRQ3314,(Blikanasaurus,((Camelotia,((Meroktenos,(Ledumahadi,Kholumolumo)),(Lessemsaurus,(Ingentia,Antetonitrus)))),((Schleitheimia,(Isanosaurus,((Tazoudasaurus,Vulcanodon),((Shunosaurus,(Spinophorosaurus,(Omeisaurus,(Mamenchisaurus,(Cetiosaurus,Neosauropoda))))),(Patagosaurus,Barapasaurus))))),(Pulanesaura,Gongxianosaurus)))))))))),(Mussaurus,Leonerasaurus)))),(Xingxiulong,(Jingshanosaurus,Chuxiongosaurus)))))),(Eucnemesaurus_fortis,(Riojasaurus,Eucnemesaurus_entaxonis)))),(Plateosaurus_gracilis,(Plateosaurus_ingens,Plateosaurus_engelhardti))))))))))))))))),(Staurikosaurus,Herrerasaurus)))))));

(Euparkeria,(Crurotarsi,(Marasuchus,(Silesaurus,(Ornithischia,((Agnosphitys,((Guaibasaurus,(Neotheropoda,Chindesaurus)),(Buriolestes,((Eoraptor,((Saturnalia,Chromogisaurus),(Bagualosaurus,(Jaklapalisaurus,(Nambalia,((Thecodontosaurus,Pantydraco),(Efraasia,(Plateosauravus,(Ruehleia,(((Unaysaurus,(Pradhania,Macrocollum)),((Sarahsaurus,((Ngwevu,(Xixipiosaurus,((Coloradisaurus,(Glacialisaurus,Lufengosaurus)),(Massospondylus_carinatus,(Adeopapposaurus,Leyesaurus))))),(Yunnanosaurus_huangi,((Seitaad,(Anchisaurus,((Irisosaurus,(Sefapanosaurus,(Yizhousaurus,(Aardonyx,(NMQR1551,(NMRQ3314,(Blikanasaurus,((Camelotia,((Meroktenos,(Ledumahadi,Kholumolumo)),(Lessemsaurus,(Ingentia,Antetonitrus)))),((Schleitheimia,(Isanosaurus,((Tazoudasaurus,Vulcanodon),((Shunosaurus,(Spinophorosaurus,(Omeisaurus,(Mamenchisaurus,(Cetiosaurus,Neosauropoda))))),(Patagosaurus,Barapasaurus))))),(Pulanesaura,Gongxianosaurus)))))))))),(Mussaurus,Leonerasaurus)))),(Xingxiulong,(Jingshanosaurus,Chuxiongosaurus)))))),(Eucnemesaurus_fortis,(Riojasaurus,Eucnemesaurus_entaxonis)))),(Plateosaurus_gracilis,(Plateosaurus_ingens,Plateosaurus_engelhardti)))))))))))),(Panphagia,Pampadromaeus))))),(Staurikosaurus,Herrerasaurus)))))));

(Euparkeria,(Crurotarsi,(Marasuchus,(Silesaurus,(Ornithischia,((Agnosphitys,((Guaibasaurus,(Neotheropoda,Chindesaurus)),((Panphagia,(Eoraptor,((Saturnalia,Chromogisaurus),(Bagualosaurus,(Jaklapalisaurus,(Nambalia,((Thecodontosaurus,Pantydraco),(Efraasia,(Plateosauravus,(Ruehleia,(((Unaysaurus,(Pradhania,Macrocollum)),((Sarahsaurus,((Ngwevu,(Xixipiosaurus,((Coloradisaurus,(Glacialisaurus,Lufengosaurus)),(Massospondylus_carinatus,(Adeopapposaurus,Leyesaurus))))),(Yunnanosaurus_huangi,((Seitaad,(Anchisaurus,((Irisosaurus,(Sefapanosaurus,(Yizhousaurus,(Aardonyx,(NMQR1551,(NMRQ3314,(Blikanasaurus,((Camelotia,((Meroktenos,(Ledumahadi,Kholumolumo)),(Lessemsaurus,(Ingentia,Antetonitrus)))),((Schleitheimia,(Isanosaurus,((Tazoudasaurus,Vulcanodon),((Shunosaurus,(Spinophorosaurus,(Omeisaurus,(Mamenchisaurus,(Cetiosaurus,Neosauropoda))))),(Patagosaurus,Barapasaurus))))),(Pulanesaura,Gongxianosaurus)))))))))),(Mussaurus,Leonerasaurus)))),(Xingxiulong,(Jingshanosaurus,Chuxiongosaurus)))))),(Eucnemesaurus_fortis,(Riojasaurus,Eucnemesaurus_entaxonis)))),(Plateosaurus_gracilis,(Plateosaurus_ingens,Plateosaurus_engelhardti))))))))))))),(Buriolestes,Pampadromaeus)))),(Staurikosaurus,Herrerasaurus)))))));

(Euparkeria,(Crurotarsi,(Marasuchus,(Silesaurus,(Ornithischia,((Agnosphitys,((Guaibasaurus,(Neotheropoda,Chindesaurus)),(Buriolestes,(Panphagia,(Eoraptor,((Pampadromaeus,(Saturnalia,Chromogisaurus)),(Bagualosaurus,(Jaklapalisaurus,(Nambalia,((Thecodontosaurus,Pantydraco),(Efraasia,(Plateosauravus,(Ruehleia,(((Unaysaurus,(Pradhania,Macrocollum)),((Sarahsaurus,((Ngwevu,((Coloradisaurus,(Glacialisaurus,Lufengosaurus)),(Xixipiosaurus,(Massospondylus_carinatus,(Adeopapposaurus,Leyesaurus))))),(Yunnanosaurus_huangi,((Seitaad,(Anchisaurus,((Irisosaurus,(Sefapanosaurus,(Yizhousaurus,(Aardonyx,(NMQR1551,(NMRQ3314,(Blikanasaurus,((Camelotia,((Meroktenos,(Ledumahadi,Kholumolumo)),(Lessemsaurus,(Ingentia,Antetonitrus)))),((Schleitheimia,(Isanosaurus,((Tazoudasaurus,Vulcanodon),((Shunosaurus,(Spinophorosaurus,(Omeisaurus,(Mamenchisaurus,(Cetiosaurus,Neosauropoda))))),(Patagosaurus,Barapasaurus))))),(Pulanesaura,Gongxianosaurus)))))))))),(Mussaurus,Leonerasaurus)))),(Xingxiulong,(Jingshanosaurus,Chuxiongosaurus)))))),(Eucnemesaurus_fortis,(Riojasaurus,Eucnemesaurus_entaxonis)))),(Plateosaurus_gracilis,(Plateosaurus_ingens,Plateosaurus_engelhardti)))))))))))))))),(Staurikosaurus,Herrerasaurus)))))));

(Euparkeria,(Crurotarsi,(Marasuchus,(Silesaurus,(Ornithischia,((Agnosphitys,((Guaibasaurus,(Neotheropoda,Chindesaurus)),(Buriolestes,(Panphagia,(Eoraptor,((Pampadromaeus,(Saturnalia,Chromogisaurus)),(Bagualosaurus,(Jaklapalisaurus,(Nambalia,((Thecodontosaurus,Pantydraco),(Efraasia,(Plateosauravus,(Ruehleia,(((Unaysaurus,(Pradhania,Macrocollum)),((Sarahsaurus,((Ngwevu,(Xixipiosaurus,((Coloradisaurus,(Glacialisaurus,Lufengosaurus)),(Massospondylus_carinatus,(Adeopapposaurus,Leyesaurus))))),(Yunnanosaurus_huangi,((Seitaad,(Anchisaurus,((Irisosaurus,(Sefapanosaurus,(Yizhousaurus,(Aardonyx,(NMQR1551,(NMRQ3314,((Camelotia,((Meroktenos,(Ledumahadi,Kholumolumo)),(Lessemsaurus,(Blikanasaurus,(Ingentia,Antetonitrus))))),((Schleitheimia,(Isanosaurus,((Tazoudasaurus,Vulcanodon),((Shunosaurus,(Spinophorosaurus,(Omeisaurus,(Mamenchisaurus,(Cetiosaurus,Neosauropoda))))),(Patagosaurus,Barapasaurus))))),(Pulanesaura,Gongxianosaurus))))))))),(Mussaurus,Leonerasaurus)))),(Xingxiulong,(Jingshanosaurus,Chuxiongosaurus)))))),(Eucnemesaurus_fortis,(Riojasaurus,Eucnemesaurus_entaxonis)))),(Plateosaurus_gracilis,(Plateosaurus_ingens,Plateosaurus_engelhardti)))))))))))))))),(Staurikosaurus,Herrerasaurus)))))));

(Euparkeria,(Crurotarsi,(Marasuchus,(Silesaurus,(Agnosphitys,(Ornithischia,(((Guaibasaurus,(Neotheropoda,Chindesaurus)),(Buriolestes,(Pampadromaeus,(Panphagia,(Eoraptor,((Saturnalia,Chromogisaurus),(Bagualosaurus,(Jaklapalisaurus,(Nambalia,((Thecodontosaurus,Pantydraco),(Efraasia,(Plateosauravus,(Ruehleia,(((Unaysaurus,(Pradhania,Macrocollum)),((Sarahsaurus,((Ngwevu,(Xixipiosaurus,((Coloradisaurus,(Glacialisaurus,Lufengosaurus)),(Massospondylus_carinatus,(Adeopapposaurus,Leyesaurus))))),(Yunnanosaurus_huangi,((Seitaad,(Anchisaurus,((Irisosaurus,(Sefapanosaurus,(Yizhousaurus,(Aardonyx,(NMQR1551,(NMRQ3314,(Blikanasaurus,((Camelotia,((Meroktenos,(Ledumahadi,Kholumolumo)),(Lessemsaurus,(Ingentia,Antetonitrus)))),((Schleitheimia,(Isanosaurus,((Tazoudasaurus,Vulcanodon),((Shunosaurus,((Spinophorosaurus,(Omeisaurus,Mamenchisaurus)),(Cetiosaurus,Neosauropoda))),(Patagosaurus,Barapasaurus))))),(Pulanesaura,Gongxianosaurus)))))))))),(Mussaurus,Leonerasaurus)))),(Xingxiulong,(Jingshanosaurus,Chuxiongosaurus)))))),(Eucnemesaurus_fortis,(Riojasaurus,Eucnemesaurus_entaxonis)))),(Plateosaurus_gracilis,(Plateosaurus_ingens,Plateosaurus_engelhardti)))))))))))))))),(Staurikosaurus,Herrerasaurus))))))));

(Euparkeria,(Crurotarsi,(Marasuchus,(Silesaurus,(Ornithischia,(Agnosphitys,(((Guaibasaurus,(Neotheropoda,Chindesaurus)),(Buriolestes,(Pampadromaeus,(Panphagia,(Eoraptor,((Saturnalia,Chromogisaurus),(Bagualosaurus,(Jaklapalisaurus,(Nambalia,((Thecodontosaurus,Pantydraco),(Efraasia,(Plateosauravus,(Ruehleia,(((Unaysaurus,(Pradhania,Macrocollum)),((Sarahsaurus,((Ngwevu,(Xixipiosaurus,((Coloradisaurus,(Glacialisaurus,Lufengosaurus)),(Massospondylus_carinatus,(Adeopapposaurus,Leyesaurus))))),(Yunnanosaurus_huangi,((Seitaad,(Anchisaurus,((Irisosaurus,(Sefapanosaurus,(Yizhousaurus,(Aardonyx,(NMQR1551,(NMRQ3314,(Blikanasaurus,((Camelotia,((Meroktenos,(Ledumahadi,Kholumolumo)),(Lessemsaurus,(Ingentia,Antetonitrus)))),((Schleitheimia,(Isanosaurus,((Tazoudasaurus,Vulcanodon),((Shunosaurus,((Spinophorosaurus,(Omeisaurus,Mamenchisaurus)),(Cetiosaurus,Neosauropoda))),(Patagosaurus,Barapasaurus))))),(Pulanesaura,Gongxianosaurus)))))))))),(Mussaurus,Leonerasaurus)))),(Xingxiulong,(Jingshanosaurus,Chuxiongosaurus)))))),(Eucnemesaurus_fortis,(Riojasaurus,Eucnemesaurus_entaxonis)))),(Plateosaurus_gracilis,(Plateosaurus_ingens,Plateosaurus_engelhardti)))))))))))))))),(Staurikosaurus,Herrerasaurus))))))));

(Euparkeria,(Crurotarsi,(Marasuchus,(Silesaurus,(Ornithischia,((Agnosphitys,((Guaibasaurus,(Neotheropoda,Chindesaurus)),(Buriolestes,(Pampadromaeus,(Panphagia,(Eoraptor,((Saturnalia,Chromogisaurus),(Bagualosaurus,(Jaklapalisaurus,(Nambalia,((Thecodontosaurus,Pantydraco),(Efraasia,(Plateosauravus,(Ruehleia,(((Unaysaurus,(Pradhania,Macrocollum)),((Sarahsaurus,((Ngwevu,((Coloradisaurus,(Glacialisaurus,Lufengosaurus)),(Xixipiosaurus,(Massospondylus_carinatus,(Adeopapposaurus,Leyesaurus))))),(Yunnanosaurus_huangi,((Seitaad,(Anchisaurus,((Irisosaurus,(Sefapanosaurus,(Yizhousaurus,(Aardonyx,(NMQR1551,(NMRQ3314,(Blikanasaurus,((Camelotia,((Meroktenos,(Ledumahadi,Kholumolumo)),(Lessemsaurus,(Ingentia,Antetonitrus)))),((Schleitheimia,(Isanosaurus,((Tazoudasaurus,Vulcanodon),((Shunosaurus,((Spinophorosaurus,(Omeisaurus,Mamenchisaurus)),(Cetiosaurus,Neosauropoda))),(Patagosaurus,Barapasaurus))))),(Pulanesaura,Gongxianosaurus)))))))))),(Mussaurus,Leonerasaurus)))),(Xingxiulong,(Jingshanosaurus,Chuxiongosaurus)))))),(Eucnemesaurus_fortis,(Riojasaurus,Eucnemesaurus_entaxonis)))),(Plateosaurus_gracilis,(Plateosaurus_ingens,Plateosaurus_engelhardti))))))))))))))))),(Staurikosaurus,Herrerasaurus)))))));

(Euparkeria,(Crurotarsi,(Marasuchus,(Silesaurus,(Ornithischia,((Agnosphitys,((Guaibasaurus,(Neotheropoda,Chindesaurus)),((Eoraptor,((Saturnalia,Chromogisaurus),(Bagualosaurus,(Jaklapalisaurus,(Nambalia,((Thecodontosaurus,Pantydraco),(Efraasia,(Plateosauravus,(Ruehleia,(((Unaysaurus,(Pradhania,Macrocollum)),((Sarahsaurus,((Ngwevu,(Xixipiosaurus,((Coloradisaurus,(Glacialisaurus,Lufengosaurus)),(Massospondylus_carinatus,(Adeopapposaurus,Leyesaurus))))),(Yunnanosaurus_huangi,((Seitaad,(Anchisaurus,((Irisosaurus,(Sefapanosaurus,(Yizhousaurus,(Aardonyx,(NMQR1551,(NMRQ3314,(Blikanasaurus,((Camelotia,((Meroktenos,(Ledumahadi,Kholumolumo)),(Lessemsaurus,(Ingentia,Antetonitrus)))),((Schleitheimia,(Isanosaurus,((Tazoudasaurus,Vulcanodon),((Shunosaurus,((Spinophorosaurus,(Omeisaurus,Mamenchisaurus)),(Cetiosaurus,Neosauropoda))),(Patagosaurus,Barapasaurus))))),(Pulanesaura,Gongxianosaurus)))))))))),(Mussaurus,Leonerasaurus)))),(Xingxiulong,(Jingshanosaurus,Chuxiongosaurus)))))),(Eucnemesaurus_fortis,(Riojasaurus,Eucnemesaurus_entaxonis)))),(Plateosaurus_gracilis,(Plateosaurus_ingens,Plateosaurus_engelhardti)))))))))))),(Buriolestes,(Panphagia,Pampadromaeus))))),(Staurikosaurus,Herrerasaurus)))))));

(Euparkeria,(Crurotarsi,(Marasuchus,(Silesaurus,(Ornithischia,((Agnosphitys,((Guaibasaurus,(Neotheropoda,Chindesaurus)),(Buriolestes,((Eoraptor,((Saturnalia,Chromogisaurus),(Bagualosaurus,(Jaklapalisaurus,(Nambalia,((Thecodontosaurus,Pantydraco),(Efraasia,(Plateosauravus,(Ruehleia,(((Unaysaurus,(Pradhania,Macrocollum)),((Sarahsaurus,((Ngwevu,((Coloradisaurus,(Glacialisaurus,Lufengosaurus)),(Xixipiosaurus,(Massospondylus_carinatus,(Adeopapposaurus,Leyesaurus))))),(Yunnanosaurus_huangi,((Seitaad,(Anchisaurus,((Irisosaurus,(Sefapanosaurus,(Yizhousaurus,(Aardonyx,(NMQR1551,(NMRQ3314,(Blikanasaurus,((Camelotia,((Meroktenos,(Ledumahadi,Kholumolumo)),(Lessemsaurus,(Ingentia,Antetonitrus)))),((Schleitheimia,(Isanosaurus,((Tazoudasaurus,Vulcanodon),((Shunosaurus,((Spinophorosaurus,(Omeisaurus,Mamenchisaurus)),(Cetiosaurus,Neosauropoda))),(Patagosaurus,Barapasaurus))))),(Pulanesaura,Gongxianosaurus)))))))))),(Mussaurus,Leonerasaurus)))),(Xingxiulong,(Jingshanosaurus,Chuxiongosaurus)))))),(Eucnemesaurus_fortis,(Riojasaurus,Eucnemesaurus_entaxonis)))),(Plateosaurus_gracilis,(Plateosaurus_ingens,Plateosaurus_engelhardti)))))))))))),(Panphagia,Pampadromaeus))))),(Staurikosaurus,Herrerasaurus)))))));

(Euparkeria,(Crurotarsi,(Marasuchus,(Silesaurus,(Ornithischia,(Agnosphitys,(((Guaibasaurus,(Neotheropoda,Chindesaurus)),((Panphagia,(Eoraptor,((Saturnalia,Chromogisaurus),(Bagualosaurus,(Jaklapalisaurus,(Nambalia,((Thecodontosaurus,Pantydraco),(Efraasia,(Plateosauravus,(Ruehleia,(((Unaysaurus,(Pradhania,Macrocollum)),((Sarahsaurus,((Ngwevu,(Xixipiosaurus,((Coloradisaurus,(Glacialisaurus,Lufengosaurus)),(Massospondylus_carinatus,(Adeopapposaurus,Leyesaurus))))),(Yunnanosaurus_huangi,((Seitaad,(Anchisaurus,((Irisosaurus,(Sefapanosaurus,(Yizhousaurus,(Aardonyx,(NMQR1551,(NMRQ3314,(Blikanasaurus,((Camelotia,((Meroktenos,(Ledumahadi,Kholumolumo)),(Lessemsaurus,(Ingentia,Antetonitrus)))),((Schleitheimia,(Isanosaurus,((Tazoudasaurus,Vulcanodon),((Shunosaurus,((Spinophorosaurus,(Omeisaurus,Mamenchisaurus)),(Cetiosaurus,Neosauropoda))),(Patagosaurus,Barapasaurus))))),(Pulanesaura,Gongxianosaurus)))))))))),(Mussaurus,Leonerasaurus)))),(Xingxiulong,(Jingshanosaurus,Chuxiongosaurus)))))),(Eucnemesaurus_fortis,(Riojasaurus,Eucnemesaurus_entaxonis)))),(Plateosaurus_gracilis,(Plateosaurus_ingens,Plateosaurus_engelhardti))))))))))))),(Buriolestes,Pampadromaeus))),(Staurikosaurus,Herrerasaurus))))))));

(Euparkeria,(Crurotarsi,(Marasuchus,(Silesaurus,(Ornithischia,((Agnosphitys,((Guaibasaurus,(Neotheropoda,Chindesaurus)),((Panphagia,(Eoraptor,((Saturnalia,Chromogisaurus),(Bagualosaurus,(Jaklapalisaurus,(Nambalia,((Thecodontosaurus,Pantydraco),(Efraasia,(Plateosauravus,(Ruehleia,(((Unaysaurus,(Pradhania,Macrocollum)),((Sarahsaurus,((Ngwevu,((Coloradisaurus,(Glacialisaurus,Lufengosaurus)),(Xixipiosaurus,(Massospondylus_carinatus,(Adeopapposaurus,Leyesaurus))))),(Yunnanosaurus_huangi,((Seitaad,(Anchisaurus,((Irisosaurus,(Sefapanosaurus,(Yizhousaurus,(Aardonyx,(NMQR1551,(NMRQ3314,(Blikanasaurus,((Camelotia,((Meroktenos,(Ledumahadi,Kholumolumo)),(Lessemsaurus,(Ingentia,Antetonitrus)))),((Schleitheimia,(Isanosaurus,((Tazoudasaurus,Vulcanodon),((Shunosaurus,((Spinophorosaurus,(Omeisaurus,Mamenchisaurus)),(Cetiosaurus,Neosauropoda))),(Patagosaurus,Barapasaurus))))),(Pulanesaura,Gongxianosaurus)))))))))),(Mussaurus,Leonerasaurus)))),(Xingxiulong,(Jingshanosaurus,Chuxiongosaurus)))))),(Eucnemesaurus_fortis,(Riojasaurus,Eucnemesaurus_entaxonis)))),(Plateosaurus_gracilis,(Plateosaurus_ingens,Plateosaurus_engelhardti))))))))))))),(Buriolestes,Pampadromaeus)))),(Staurikosaurus,Herrerasaurus)))))));

(Euparkeria,(Crurotarsi,(Marasuchus,(Silesaurus,(Agnosphitys,(Ornithischia,(((Guaibasaurus,(Neotheropoda,Chindesaurus)),(Buriolestes,(Pampadromaeus,(Panphagia,(Eoraptor,((Saturnalia,Chromogisaurus),(Bagualosaurus,(Jaklapalisaurus,(Nambalia,((Thecodontosaurus,Pantydraco),(Efraasia,(Plateosauravus,(Ruehleia,(((Unaysaurus,(Pradhania,Macrocollum)),((Sarahsaurus,((Ngwevu,(Xixipiosaurus,((Coloradisaurus,(Glacialisaurus,Lufengosaurus)),(Massospondylus_carinatus,(Adeopapposaurus,Leyesaurus))))),(Yunnanosaurus_huangi,((Seitaad,(Anchisaurus,((Yizhousaurus,((Sefapanosaurus,Aardonyx),(NMQR1551,(NMRQ3314,(Blikanasaurus,((Camelotia,((Meroktenos,(Ledumahadi,Kholumolumo)),(Lessemsaurus,(Ingentia,Antetonitrus)))),(Pulanesaura,(Gongxianosaurus,(Schleitheimia,(Isanosaurus,((Tazoudasaurus,Vulcanodon),((Shunosaurus,((Spinophorosaurus,Omeisaurus),(Mamenchisaurus,(Cetiosaurus,Neosauropoda)))),(Patagosaurus,Barapasaurus))))))))))))),(Irisosaurus,(Mussaurus,Leonerasaurus))))),(Xingxiulong,(Jingshanosaurus,Chuxiongosaurus)))))),(Eucnemesaurus_fortis,(Riojasaurus,Eucnemesaurus_entaxonis)))),(Plateosaurus_gracilis,(Plateosaurus_ingens,Plateosaurus_engelhardti)))))))))))))))),(Staurikosaurus,Herrerasaurus))))))));

(Euparkeria,(Crurotarsi,(Marasuchus,(Silesaurus,(Ornithischia,(Agnosphitys,(((Guaibasaurus,(Neotheropoda,Chindesaurus)),(Buriolestes,(Pampadromaeus,(Panphagia,(Eoraptor,((Saturnalia,Chromogisaurus),(Bagualosaurus,(Jaklapalisaurus,(Nambalia,((Thecodontosaurus,Pantydraco),(Efraasia,(Plateosauravus,(Ruehleia,(((Unaysaurus,(Pradhania,Macrocollum)),((Sarahsaurus,((Ngwevu,(Xixipiosaurus,((Coloradisaurus,(Glacialisaurus,Lufengosaurus)),(Massospondylus_carinatus,(Adeopapposaurus,Leyesaurus))))),(Yunnanosaurus_huangi,((Seitaad,(Anchisaurus,((Irisosaurus,(Yizhousaurus,((Sefapanosaurus,Aardonyx),(NMQR1551,(NMRQ3314,(Blikanasaurus,((Camelotia,((Meroktenos,(Ledumahadi,Kholumolumo)),(Lessemsaurus,(Ingentia,Antetonitrus)))),(Pulanesaura,(Gongxianosaurus,(Schleitheimia,(Isanosaurus,((Tazoudasaurus,Vulcanodon),((Shunosaurus,(Spinophorosaurus,(Mamenchisaurus,(Omeisaurus,(Cetiosaurus,Neosauropoda))))),(Patagosaurus,Barapasaurus)))))))))))))),(Mussaurus,Leonerasaurus)))),(Xingxiulong,(Jingshanosaurus,Chuxiongosaurus)))))),(Eucnemesaurus_fortis,(Riojasaurus,Eucnemesaurus_entaxonis)))),(Plateosaurus_gracilis,(Plateosaurus_ingens,Plateosaurus_engelhardti)))))))))))))))),(Staurikosaurus,Herrerasaurus))))))));

(Euparkeria,(Crurotarsi,(Marasuchus,(Silesaurus,(Ornithischia,((Agnosphitys,((Guaibasaurus,(Neotheropoda,Chindesaurus)),(Buriolestes,(Pampadromaeus,(Panphagia,(Eoraptor,((Saturnalia,Chromogisaurus),(Bagualosaurus,(Jaklapalisaurus,(Nambalia,((Thecodontosaurus,Pantydraco),(Efraasia,(Plateosauravus,(Ruehleia,(((Unaysaurus,(Pradhania,Macrocollum)),((Sarahsaurus,((Ngwevu,(Xixipiosaurus,((Coloradisaurus,(Glacialisaurus,Lufengosaurus)),(Massospondylus_carinatus,(Adeopapposaurus,Leyesaurus))))),(Yunnanosaurus_huangi,((Seitaad,(Anchisaurus,((Irisosaurus,(Yizhousaurus,((Sefapanosaurus,Aardonyx),(NMQR1551,(NMRQ3314,(Blikanasaurus,((Camelotia,((Meroktenos,(Ledumahadi,Kholumolumo)),(Lessemsaurus,(Ingentia,Antetonitrus)))),(Pulanesaura,(Gongxianosaurus,(Schleitheimia,(Isanosaurus,((Tazoudasaurus,Vulcanodon),((Shunosaurus,(Spinophorosaurus,(Mamenchisaurus,(Omeisaurus,(Cetiosaurus,Neosauropoda))))),(Patagosaurus,Barapasaurus)))))))))))))),(Mussaurus,Leonerasaurus)))),(Xingxiulong,(Jingshanosaurus,Chuxiongosaurus)))))),(Eucnemesaurus_fortis,(Riojasaurus,Eucnemesaurus_entaxonis)))),(Plateosaurus_gracilis,(Plateosaurus_ingens,Plateosaurus_engelhardti))))))))))))))))),(Staurikosaurus,Herrerasaurus)))))));

(Euparkeria,(Crurotarsi,(Marasuchus,(Silesaurus,(Agnosphitys,(Ornithischia,(((Guaibasaurus,(Neotheropoda,Chindesaurus)),((Panphagia,(Eoraptor,((Saturnalia,Chromogisaurus),(Bagualosaurus,(Jaklapalisaurus,(Nambalia,((Thecodontosaurus,Pantydraco),(Efraasia,(Plateosauravus,(Ruehleia,(((Unaysaurus,(Pradhania,Macrocollum)),((Sarahsaurus,((Ngwevu,(Xixipiosaurus,((Coloradisaurus,(Glacialisaurus,Lufengosaurus)),(Massospondylus_carinatus,(Adeopapposaurus,Leyesaurus))))),(Yunnanosaurus_huangi,((Seitaad,(Anchisaurus,((Irisosaurus,(Yizhousaurus,((Sefapanosaurus,Aardonyx),(NMQR1551,(NMRQ3314,(Blikanasaurus,((Camelotia,((Meroktenos,(Ledumahadi,Kholumolumo)),(Lessemsaurus,(Ingentia,Antetonitrus)))),(Pulanesaura,(Gongxianosaurus,(Schleitheimia,(Isanosaurus,((Tazoudasaurus,Vulcanodon),((Shunosaurus,(Spinophorosaurus,(Mamenchisaurus,(Omeisaurus,(Cetiosaurus,Neosauropoda))))),(Patagosaurus,Barapasaurus)))))))))))))),(Mussaurus,Leonerasaurus)))),(Xingxiulong,(Jingshanosaurus,Chuxiongosaurus)))))),(Eucnemesaurus_fortis,(Riojasaurus,Eucnemesaurus_entaxonis)))),(Plateosaurus_gracilis,(Plateosaurus_ingens,Plateosaurus_engelhardti))))))))))))),(Buriolestes,Pampadromaeus))),(Staurikosaurus,Herrerasaurus))))))));

(Euparkeria,(Crurotarsi,(Marasuchus,(Silesaurus,(Agnosphitys,(Ornithischia,(((Guaibasaurus,(Neotheropoda,Chindesaurus)),(Buriolestes,(Pampadromaeus,(Panphagia,(Eoraptor,((Saturnalia,Chromogisaurus),(Bagualosaurus,(Jaklapalisaurus,(Nambalia,((Thecodontosaurus,Pantydraco),(Efraasia,(Plateosauravus,(Ruehleia,(((Unaysaurus,(Pradhania,Macrocollum)),((Sarahsaurus,((Ngwevu,((Coloradisaurus,(Glacialisaurus,Lufengosaurus)),(Xixipiosaurus,(Massospondylus_carinatus,(Adeopapposaurus,Leyesaurus))))),(Yunnanosaurus_huangi,((Seitaad,(Anchisaurus,((Irisosaurus,(Yizhousaurus,((Sefapanosaurus,Aardonyx),(NMQR1551,(NMRQ3314,(Blikanasaurus,((Camelotia,((Meroktenos,(Ledumahadi,Kholumolumo)),(Lessemsaurus,(Ingentia,Antetonitrus)))),(Pulanesaura,(Gongxianosaurus,(Schleitheimia,(Isanosaurus,((Tazoudasaurus,Vulcanodon),((Shunosaurus,(Spinophorosaurus,(Mamenchisaurus,(Omeisaurus,(Cetiosaurus,Neosauropoda))))),(Patagosaurus,Barapasaurus)))))))))))))),(Mussaurus,Leonerasaurus)))),(Xingxiulong,(Jingshanosaurus,Chuxiongosaurus)))))),(Eucnemesaurus_fortis,(Riojasaurus,Eucnemesaurus_entaxonis)))),(Plateosaurus_gracilis,(Plateosaurus_ingens,Plateosaurus_engelhardti)))))))))))))))),(Staurikosaurus,Herrerasaurus))))))));

(Euparkeria,(Crurotarsi,(Marasuchus,(Silesaurus,(Agnosphitys,(Ornithischia,(((Guaibasaurus,(Neotheropoda,Chindesaurus)),(Buriolestes,(Pampadromaeus,(Panphagia,(Eoraptor,((Saturnalia,Chromogisaurus),(Bagualosaurus,(Jaklapalisaurus,(Nambalia,((Thecodontosaurus,Pantydraco),(Efraasia,(Plateosauravus,(Ruehleia,(((Unaysaurus,(Pradhania,Macrocollum)),((Sarahsaurus,((Ngwevu,(Xixipiosaurus,((Coloradisaurus,(Glacialisaurus,Lufengosaurus)),(Massospondylus_carinatus,(Adeopapposaurus,Leyesaurus))))),(Yunnanosaurus_huangi,((Seitaad,(Anchisaurus,((Irisosaurus,(Sefapanosaurus,(Yizhousaurus,(Aardonyx,(NMQR1551,(NMRQ3314,(Blikanasaurus,((Camelotia,((Meroktenos,(Ledumahadi,Kholumolumo)),(Lessemsaurus,(Ingentia,Antetonitrus)))),(Pulanesaura,(Gongxianosaurus,(Schleitheimia,(Isanosaurus,((Tazoudasaurus,Vulcanodon),((Shunosaurus,(Spinophorosaurus,(Mamenchisaurus,(Omeisaurus,(Cetiosaurus,Neosauropoda))))),(Patagosaurus,Barapasaurus))))))))))))))),(Mussaurus,Leonerasaurus)))),(Xingxiulong,(Jingshanosaurus,Chuxiongosaurus)))))),(Eucnemesaurus_fortis,(Riojasaurus,Eucnemesaurus_entaxonis)))),(Plateosaurus_gracilis,(Plateosaurus_ingens,Plateosaurus_engelhardti)))))))))))))))),(Staurikosaurus,Herrerasaurus))))))));

(Euparkeria,(Crurotarsi,(Marasuchus,(Silesaurus,(Agnosphitys,(Ornithischia,(((Guaibasaurus,(Neotheropoda,Chindesaurus)),(Buriolestes,(Pampadromaeus,(Panphagia,(Eoraptor,((Saturnalia,Chromogisaurus),(Bagualosaurus,(Jaklapalisaurus,(Nambalia,((Thecodontosaurus,Pantydraco),(Efraasia,(Plateosauravus,(Ruehleia,(((Unaysaurus,(Pradhania,Macrocollum)),((Sarahsaurus,((Ngwevu,(Xixipiosaurus,((Coloradisaurus,(Glacialisaurus,Lufengosaurus)),(Massospondylus_carinatus,(Adeopapposaurus,Leyesaurus))))),(Yunnanosaurus_huangi,((Seitaad,(Anchisaurus,((Irisosaurus,(Yizhousaurus,((Sefapanosaurus,Aardonyx),(NMQR1551,(NMRQ3314,(Blikanasaurus,((Camelotia,((Meroktenos,(Ledumahadi,Kholumolumo)),(Lessemsaurus,(Ingentia,Antetonitrus)))),((Schleitheimia,(Isanosaurus,((Tazoudasaurus,Vulcanodon),((Shunosaurus,(Spinophorosaurus,(Mamenchisaurus,(Omeisaurus,(Cetiosaurus,Neosauropoda))))),(Patagosaurus,Barapasaurus))))),(Pulanesaura,Gongxianosaurus))))))))),(Mussaurus,Leonerasaurus)))),(Xingxiulong,(Jingshanosaurus,Chuxiongosaurus)))))),(Eucnemesaurus_fortis,(Riojasaurus,Eucnemesaurus_entaxonis)))),(Plateosaurus_gracilis,(Plateosaurus_ingens,Plateosaurus_engelhardti)))))))))))))))),(Staurikosaurus,Herrerasaurus))))))));

(Euparkeria,(Crurotarsi,(Marasuchus,(Silesaurus,(Agnosphitys,(Ornithischia,(((Guaibasaurus,(Neotheropoda,Chindesaurus)),(Buriolestes,(Pampadromaeus,(Panphagia,(Eoraptor,((Saturnalia,Chromogisaurus),(Bagualosaurus,(Jaklapalisaurus,(Nambalia,((Thecodontosaurus,Pantydraco),(Efraasia,(Plateosauravus,(Ruehleia,(((Unaysaurus,(Pradhania,Macrocollum)),((Sarahsaurus,((Ngwevu,(Xixipiosaurus,((Coloradisaurus,(Glacialisaurus,Lufengosaurus)),(Massospondylus_carinatus,(Adeopapposaurus,Leyesaurus))))),(Yunnanosaurus_huangi,((Seitaad,(Anchisaurus,((Irisosaurus,(Yizhousaurus,((Sefapanosaurus,Aardonyx),(NMQR1551,(NMRQ3314,(Blikanasaurus,((Camelotia,((Meroktenos,(Ledumahadi,Kholumolumo)),(Lessemsaurus,(Ingentia,Antetonitrus)))),(Pulanesaura,(Gongxianosaurus,(Schleitheimia,(Isanosaurus,((Tazoudasaurus,Vulcanodon),((Shunosaurus,(Spinophorosaurus,(Omeisaurus,(Mamenchisaurus,(Cetiosaurus,Neosauropoda))))),(Patagosaurus,Barapasaurus)))))))))))))),(Mussaurus,Leonerasaurus)))),(Xingxiulong,(Jingshanosaurus,Chuxiongosaurus)))))),(Eucnemesaurus_fortis,(Riojasaurus,Eucnemesaurus_entaxonis)))),(Plateosaurus_gracilis,(Plateosaurus_ingens,Plateosaurus_engelhardti)))))))))))))))),(Staurikosaurus,Herrerasaurus))))))));

(Euparkeria,(Crurotarsi,(Marasuchus,(Silesaurus,(Agnosphitys,(Ornithischia,(((Guaibasaurus,(Neotheropoda,Chindesaurus)),(Buriolestes,(Pampadromaeus,(Panphagia,(Eoraptor,((Saturnalia,Chromogisaurus),(Bagualosaurus,(Jaklapalisaurus,(Nambalia,((Thecodontosaurus,Pantydraco),(Efraasia,(Plateosauravus,(Ruehleia,(((Unaysaurus,(Pradhania,Macrocollum)),((Sarahsaurus,((Ngwevu,(Xixipiosaurus,((Coloradisaurus,(Glacialisaurus,Lufengosaurus)),(Massospondylus_carinatus,(Adeopapposaurus,Leyesaurus))))),(Yunnanosaurus_huangi,((Seitaad,(Anchisaurus,((Irisosaurus,(Yizhousaurus,((Sefapanosaurus,Aardonyx),(NMQR1551,(NMRQ3314,(Blikanasaurus,((Camelotia,((Meroktenos,(Ledumahadi,Kholumolumo)),(Lessemsaurus,(Ingentia,Antetonitrus)))),(Pulanesaura,(Gongxianosaurus,(Schleitheimia,(Isanosaurus,((Tazoudasaurus,Vulcanodon),((Shunosaurus,((Spinophorosaurus,Omeisaurus),(Mamenchisaurus,(Cetiosaurus,Neosauropoda)))),(Patagosaurus,Barapasaurus)))))))))))))),(Mussaurus,Leonerasaurus)))),(Xingxiulong,(Jingshanosaurus,Chuxiongosaurus)))))),(Eucnemesaurus_fortis,(Riojasaurus,Eucnemesaurus_entaxonis)))),(Plateosaurus_gracilis,(Plateosaurus_ingens,Plateosaurus_engelhardti)))))))))))))))),(Staurikosaurus,Herrerasaurus))))))));

(Euparkeria,(Crurotarsi,(Marasuchus,(Silesaurus,(Agnosphitys,(Ornithischia,(((Guaibasaurus,(Neotheropoda,Chindesaurus)),(Buriolestes,(Pampadromaeus,(Panphagia,(Eoraptor,((Saturnalia,Chromogisaurus),(Bagualosaurus,(Jaklapalisaurus,(Nambalia,((Thecodontosaurus,Pantydraco),(Efraasia,(Plateosauravus,(Ruehleia,(((Unaysaurus,(Pradhania,Macrocollum)),((Sarahsaurus,((Ngwevu,(Xixipiosaurus,((Coloradisaurus,(Glacialisaurus,Lufengosaurus)),(Massospondylus_carinatus,(Adeopapposaurus,Leyesaurus))))),(Yunnanosaurus_huangi,((Seitaad,(Anchisaurus,((Irisosaurus,(Yizhousaurus,((Sefapanosaurus,Aardonyx),(NMQR1551,(NMRQ3314,(Blikanasaurus,((Camelotia,((Meroktenos,(Ledumahadi,Kholumolumo)),(Lessemsaurus,(Ingentia,Antetonitrus)))),(Pulanesaura,(Gongxianosaurus,(Schleitheimia,(Isanosaurus,((Tazoudasaurus,Vulcanodon),((Shunosaurus,((Spinophorosaurus,(Omeisaurus,Mamenchisaurus)),(Cetiosaurus,Neosauropoda))),(Patagosaurus,Barapasaurus)))))))))))))),(Mussaurus,Leonerasaurus)))),(Xingxiulong,(Jingshanosaurus,Chuxiongosaurus)))))),(Eucnemesaurus_fortis,(Riojasaurus,Eucnemesaurus_entaxonis)))),(Plateosaurus_gracilis,(Plateosaurus_ingens,Plateosaurus_engelhardti)))))))))))))))),(Staurikosaurus,Herrerasaurus))))))));

(Euparkeria,(Crurotarsi,(Marasuchus,(Silesaurus,(Ornithischia,(Agnosphitys,(((Guaibasaurus,(Neotheropoda,Chindesaurus)),(Buriolestes,(Pampadromaeus,(Panphagia,(Eoraptor,((Saturnalia,Chromogisaurus),(Bagualosaurus,(Jaklapalisaurus,(Nambalia,((Thecodontosaurus,Pantydraco),(Efraasia,(Plateosauravus,(Ruehleia,(((Unaysaurus,(Pradhania,Macrocollum)),((Sarahsaurus,((Ngwevu,(Xixipiosaurus,((Coloradisaurus,(Glacialisaurus,Lufengosaurus)),(Massospondylus_carinatus,(Adeopapposaurus,Leyesaurus))))),(Yunnanosaurus_huangi,((Seitaad,(Anchisaurus,((Yizhousaurus,(Sefapanosaurus,Aardonyx)),((Mussaurus,Leonerasaurus),(Irisosaurus,(NMQR1551,(NMRQ3314,(Blikanasaurus,((Camelotia,((Meroktenos,(Ledumahadi,Kholumolumo)),(Lessemsaurus,(Ingentia,Antetonitrus)))),(Pulanesaura,(Gongxianosaurus,(Schleitheimia,(Isanosaurus,((Tazoudasaurus,Vulcanodon),((Shunosaurus,(Spinophorosaurus,(Mamenchisaurus,(Omeisaurus,(Cetiosaurus,Neosauropoda))))),(Patagosaurus,Barapasaurus)))))))))))))))),(Xingxiulong,(Jingshanosaurus,Chuxiongosaurus)))))),(Eucnemesaurus_fortis,(Riojasaurus,Eucnemesaurus_entaxonis)))),(Plateosaurus_gracilis,(Plateosaurus_ingens,Plateosaurus_engelhardti)))))))))))))))),(Staurikosaurus,Herrerasaurus))))))));

(Euparkeria,(Crurotarsi,(Marasuchus,(Silesaurus,(Ornithischia,((Agnosphitys,((Guaibasaurus,(Neotheropoda,Chindesaurus)),(Buriolestes,(Pampadromaeus,(Panphagia,(Eoraptor,((Saturnalia,Chromogisaurus),(Bagualosaurus,(Jaklapalisaurus,(Nambalia,((Thecodontosaurus,Pantydraco),(Efraasia,(Plateosauravus,(Ruehleia,(((Unaysaurus,(Pradhania,Macrocollum)),((Sarahsaurus,((Ngwevu,(Xixipiosaurus,((Coloradisaurus,(Glacialisaurus,Lufengosaurus)),(Massospondylus_carinatus,(Adeopapposaurus,Leyesaurus))))),(Yunnanosaurus_huangi,((Seitaad,(Anchisaurus,((Yizhousaurus,(Sefapanosaurus,Aardonyx)),((Mussaurus,Leonerasaurus),(Irisosaurus,(NMQR1551,(NMRQ3314,(Blikanasaurus,((Camelotia,((Meroktenos,(Ledumahadi,Kholumolumo)),(Lessemsaurus,(Ingentia,Antetonitrus)))),(Pulanesaura,(Gongxianosaurus,(Schleitheimia,(Isanosaurus,((Tazoudasaurus,Vulcanodon),((Shunosaurus,(Spinophorosaurus,(Mamenchisaurus,(Omeisaurus,(Cetiosaurus,Neosauropoda))))),(Patagosaurus,Barapasaurus)))))))))))))))),(Xingxiulong,(Jingshanosaurus,Chuxiongosaurus)))))),(Eucnemesaurus_fortis,(Riojasaurus,Eucnemesaurus_entaxonis)))),(Plateosaurus_gracilis,(Plateosaurus_ingens,Plateosaurus_engelhardti))))))))))))))))),(Staurikosaurus,Herrerasaurus)))))));

(Euparkeria,(Crurotarsi,(Marasuchus,(Silesaurus,(Agnosphitys,(Ornithischia,(((Guaibasaurus,(Neotheropoda,Chindesaurus)),((Panphagia,(Eoraptor,((Saturnalia,Chromogisaurus),(Bagualosaurus,(Jaklapalisaurus,(Nambalia,((Thecodontosaurus,Pantydraco),(Efraasia,(Plateosauravus,(Ruehleia,(((Unaysaurus,(Pradhania,Macrocollum)),((Sarahsaurus,((Ngwevu,(Xixipiosaurus,((Coloradisaurus,(Glacialisaurus,Lufengosaurus)),(Massospondylus_carinatus,(Adeopapposaurus,Leyesaurus))))),(Yunnanosaurus_huangi,((Seitaad,(Anchisaurus,((Yizhousaurus,(Sefapanosaurus,Aardonyx)),((Mussaurus,Leonerasaurus),(Irisosaurus,(NMQR1551,(NMRQ3314,(Blikanasaurus,((Camelotia,((Meroktenos,(Ledumahadi,Kholumolumo)),(Lessemsaurus,(Ingentia,Antetonitrus)))),(Pulanesaura,(Gongxianosaurus,(Schleitheimia,(Isanosaurus,((Tazoudasaurus,Vulcanodon),((Shunosaurus,(Spinophorosaurus,(Mamenchisaurus,(Omeisaurus,(Cetiosaurus,Neosauropoda))))),(Patagosaurus,Barapasaurus)))))))))))))))),(Xingxiulong,(Jingshanosaurus,Chuxiongosaurus)))))),(Eucnemesaurus_fortis,(Riojasaurus,Eucnemesaurus_entaxonis)))),(Plateosaurus_gracilis,(Plateosaurus_ingens,Plateosaurus_engelhardti))))))))))))),(Buriolestes,Pampadromaeus))),(Staurikosaurus,Herrerasaurus))))))));

(Euparkeria,(Crurotarsi,(Marasuchus,(Silesaurus,(Agnosphitys,(Ornithischia,(((Guaibasaurus,(Neotheropoda,Chindesaurus)),(Buriolestes,(Pampadromaeus,(Panphagia,(Eoraptor,((Saturnalia,Chromogisaurus),(Bagualosaurus,(Jaklapalisaurus,(Nambalia,((Thecodontosaurus,Pantydraco),(Efraasia,(Plateosauravus,(Ruehleia,(((Unaysaurus,(Pradhania,Macrocollum)),((Sarahsaurus,((Ngwevu,((Coloradisaurus,(Glacialisaurus,Lufengosaurus)),(Xixipiosaurus,(Massospondylus_carinatus,(Adeopapposaurus,Leyesaurus))))),(Yunnanosaurus_huangi,((Seitaad,(Anchisaurus,((Yizhousaurus,(Sefapanosaurus,Aardonyx)),((Mussaurus,Leonerasaurus),(Irisosaurus,(NMQR1551,(NMRQ3314,(Blikanasaurus,((Camelotia,((Meroktenos,(Ledumahadi,Kholumolumo)),(Lessemsaurus,(Ingentia,Antetonitrus)))),(Pulanesaura,(Gongxianosaurus,(Schleitheimia,(Isanosaurus,((Tazoudasaurus,Vulcanodon),((Shunosaurus,(Spinophorosaurus,(Mamenchisaurus,(Omeisaurus,(Cetiosaurus,Neosauropoda))))),(Patagosaurus,Barapasaurus)))))))))))))))),(Xingxiulong,(Jingshanosaurus,Chuxiongosaurus)))))),(Eucnemesaurus_fortis,(Riojasaurus,Eucnemesaurus_entaxonis)))),(Plateosaurus_gracilis,(Plateosaurus_ingens,Plateosaurus_engelhardti)))))))))))))))),(Staurikosaurus,Herrerasaurus))))))));

(Euparkeria,(Crurotarsi,(Marasuchus,(Silesaurus,(Agnosphitys,(Ornithischia,(((Guaibasaurus,(Neotheropoda,Chindesaurus)),(Buriolestes,(Pampadromaeus,(Panphagia,(Eoraptor,((Saturnalia,Chromogisaurus),(Bagualosaurus,(Jaklapalisaurus,(Nambalia,((Thecodontosaurus,Pantydraco),(Efraasia,(Plateosauravus,(Ruehleia,(((Unaysaurus,(Pradhania,Macrocollum)),((Sarahsaurus,((Ngwevu,(Xixipiosaurus,((Coloradisaurus,(Glacialisaurus,Lufengosaurus)),(Massospondylus_carinatus,(Adeopapposaurus,Leyesaurus))))),(Yunnanosaurus_huangi,((Seitaad,(Anchisaurus,((Yizhousaurus,Aardonyx),((Leonerasaurus,(Mussaurus,Sefapanosaurus)),(Irisosaurus,(NMQR1551,(NMRQ3314,(Blikanasaurus,((Camelotia,((Meroktenos,(Ledumahadi,Kholumolumo)),(Lessemsaurus,(Ingentia,Antetonitrus)))),(Pulanesaura,(Gongxianosaurus,(Schleitheimia,(Isanosaurus,((Tazoudasaurus,Vulcanodon),((Shunosaurus,(Spinophorosaurus,(Mamenchisaurus,(Omeisaurus,(Cetiosaurus,Neosauropoda))))),(Patagosaurus,Barapasaurus)))))))))))))))),(Xingxiulong,(Jingshanosaurus,Chuxiongosaurus)))))),(Eucnemesaurus_fortis,(Riojasaurus,Eucnemesaurus_entaxonis)))),(Plateosaurus_gracilis,(Plateosaurus_ingens,Plateosaurus_engelhardti)))))))))))))))),(Staurikosaurus,Herrerasaurus))))))));

(Euparkeria,(Crurotarsi,(Marasuchus,(Silesaurus,(Agnosphitys,(Ornithischia,(((Guaibasaurus,(Neotheropoda,Chindesaurus)),(Buriolestes,(Pampadromaeus,(Panphagia,(Eoraptor,((Saturnalia,Chromogisaurus),(Bagualosaurus,(Jaklapalisaurus,(Nambalia,((Thecodontosaurus,Pantydraco),(Efraasia,(Plateosauravus,(Ruehleia,(((Unaysaurus,(Pradhania,Macrocollum)),((Sarahsaurus,((Ngwevu,(Xixipiosaurus,((Coloradisaurus,(Glacialisaurus,Lufengosaurus)),(Massospondylus_carinatus,(Adeopapposaurus,Leyesaurus))))),(Yunnanosaurus_huangi,((Seitaad,(Anchisaurus,((Yizhousaurus,(Sefapanosaurus,Aardonyx)),((Mussaurus,Leonerasaurus),(Irisosaurus,(NMQR1551,(NMRQ3314,(Blikanasaurus,((Camelotia,((Meroktenos,(Ledumahadi,Kholumolumo)),(Lessemsaurus,(Ingentia,Antetonitrus)))),((Schleitheimia,(Isanosaurus,((Tazoudasaurus,Vulcanodon),((Shunosaurus,(Spinophorosaurus,(Mamenchisaurus,(Omeisaurus,(Cetiosaurus,Neosauropoda))))),(Patagosaurus,Barapasaurus))))),(Pulanesaura,Gongxianosaurus))))))))))),(Xingxiulong,(Jingshanosaurus,Chuxiongosaurus)))))),(Eucnemesaurus_fortis,(Riojasaurus,Eucnemesaurus_entaxonis)))),(Plateosaurus_gracilis,(Plateosaurus_ingens,Plateosaurus_engelhardti)))))))))))))))),(Staurikosaurus,Herrerasaurus))))))));

(Euparkeria,(Crurotarsi,(Marasuchus,(Silesaurus,(Agnosphitys,(Ornithischia,(((Guaibasaurus,(Neotheropoda,Chindesaurus)),(Buriolestes,(Pampadromaeus,(Panphagia,(Eoraptor,((Saturnalia,Chromogisaurus),(Bagualosaurus,(Jaklapalisaurus,(Nambalia,((Thecodontosaurus,Pantydraco),(Efraasia,(Plateosauravus,(Ruehleia,(((Unaysaurus,(Pradhania,Macrocollum)),((Sarahsaurus,((Ngwevu,(Xixipiosaurus,((Coloradisaurus,(Glacialisaurus,Lufengosaurus)),(Massospondylus_carinatus,(Adeopapposaurus,Leyesaurus))))),(Yunnanosaurus_huangi,((Seitaad,(Anchisaurus,((Yizhousaurus,(Sefapanosaurus,Aardonyx)),((Mussaurus,Leonerasaurus),((Irisosaurus,(NMRQ3314,NMQR1551)),(Blikanasaurus,((Camelotia,((Meroktenos,(Ledumahadi,Kholumolumo)),(Lessemsaurus,(Ingentia,Antetonitrus)))),(Pulanesaura,(Gongxianosaurus,(Schleitheimia,(Isanosaurus,((Tazoudasaurus,Vulcanodon),((Shunosaurus,(Spinophorosaurus,(Mamenchisaurus,(Omeisaurus,(Cetiosaurus,Neosauropoda))))),(Patagosaurus,Barapasaurus)))))))))))))),(Xingxiulong,(Jingshanosaurus,Chuxiongosaurus)))))),(Eucnemesaurus_fortis,(Riojasaurus,Eucnemesaurus_entaxonis)))),(Plateosaurus_gracilis,(Plateosaurus_ingens,Plateosaurus_engelhardti)))))))))))))))),(Staurikosaurus,Herrerasaurus))))))));

(Euparkeria,(Crurotarsi,(Marasuchus,(Silesaurus,(Ornithischia,(Agnosphitys,(((Guaibasaurus,(Neotheropoda,Chindesaurus)),(Buriolestes,(Pampadromaeus,(Panphagia,(Eoraptor,((Saturnalia,Chromogisaurus),(Bagualosaurus,(Jaklapalisaurus,(Nambalia,((Thecodontosaurus,Pantydraco),(Efraasia,(Plateosauravus,(Ruehleia,(((Unaysaurus,(Pradhania,Macrocollum)),((Sarahsaurus,((Ngwevu,(Xixipiosaurus,((Coloradisaurus,(Glacialisaurus,Lufengosaurus)),(Massospondylus_carinatus,(Adeopapposaurus,Leyesaurus))))),(Yunnanosaurus_huangi,((Seitaad,(Anchisaurus,((Yizhousaurus,((Sefapanosaurus,Aardonyx),(NMQR1551,(NMRQ3314,(Blikanasaurus,((Camelotia,((Meroktenos,(Ledumahadi,Kholumolumo)),(Lessemsaurus,(Ingentia,Antetonitrus)))),((Schleitheimia,(Isanosaurus,((Tazoudasaurus,Vulcanodon),((Shunosaurus,(Spinophorosaurus,(Mamenchisaurus,(Omeisaurus,(Cetiosaurus,Neosauropoda))))),(Patagosaurus,Barapasaurus))))),(Pulanesaura,Gongxianosaurus)))))))),(Irisosaurus,(Mussaurus,Leonerasaurus))))),(Xingxiulong,(Jingshanosaurus,Chuxiongosaurus)))))),(Eucnemesaurus_fortis,(Riojasaurus,Eucnemesaurus_entaxonis)))),(Plateosaurus_gracilis,(Plateosaurus_ingens,Plateosaurus_engelhardti)))))))))))))))),(Staurikosaurus,Herrerasaurus))))))));

(Euparkeria,(Crurotarsi,(Marasuchus,(Silesaurus,(Ornithischia,((Agnosphitys,((Guaibasaurus,(Neotheropoda,Chindesaurus)),(Buriolestes,(Pampadromaeus,(Panphagia,(Eoraptor,((Saturnalia,Chromogisaurus),(Bagualosaurus,(Jaklapalisaurus,(Nambalia,((Thecodontosaurus,Pantydraco),(Efraasia,(Plateosauravus,(Ruehleia,(((Unaysaurus,(Pradhania,Macrocollum)),((Sarahsaurus,((Ngwevu,(Xixipiosaurus,((Coloradisaurus,(Glacialisaurus,Lufengosaurus)),(Massospondylus_carinatus,(Adeopapposaurus,Leyesaurus))))),(Yunnanosaurus_huangi,((Seitaad,(Anchisaurus,((Yizhousaurus,((Sefapanosaurus,Aardonyx),(NMQR1551,(NMRQ3314,(Blikanasaurus,((Camelotia,((Meroktenos,(Ledumahadi,Kholumolumo)),(Lessemsaurus,(Ingentia,Antetonitrus)))),((Schleitheimia,(Isanosaurus,((Tazoudasaurus,Vulcanodon),((Shunosaurus,(Spinophorosaurus,(Mamenchisaurus,(Omeisaurus,(Cetiosaurus,Neosauropoda))))),(Patagosaurus,Barapasaurus))))),(Pulanesaura,Gongxianosaurus)))))))),(Irisosaurus,(Mussaurus,Leonerasaurus))))),(Xingxiulong,(Jingshanosaurus,Chuxiongosaurus)))))),(Eucnemesaurus_fortis,(Riojasaurus,Eucnemesaurus_entaxonis)))),(Plateosaurus_gracilis,(Plateosaurus_ingens,Plateosaurus_engelhardti))))))))))))))))),(Staurikosaurus,Herrerasaurus)))))));

(Euparkeria,(Crurotarsi,(Marasuchus,(Silesaurus,(Agnosphitys,(Ornithischia,(((Guaibasaurus,(Neotheropoda,Chindesaurus)),((Panphagia,(Eoraptor,((Saturnalia,Chromogisaurus),(Bagualosaurus,(Jaklapalisaurus,(Nambalia,((Thecodontosaurus,Pantydraco),(Efraasia,(Plateosauravus,(Ruehleia,(((Unaysaurus,(Pradhania,Macrocollum)),((Sarahsaurus,((Ngwevu,(Xixipiosaurus,((Coloradisaurus,(Glacialisaurus,Lufengosaurus)),(Massospondylus_carinatus,(Adeopapposaurus,Leyesaurus))))),(Yunnanosaurus_huangi,((Seitaad,(Anchisaurus,((Yizhousaurus,((Sefapanosaurus,Aardonyx),(NMQR1551,(NMRQ3314,(Blikanasaurus,((Camelotia,((Meroktenos,(Ledumahadi,Kholumolumo)),(Lessemsaurus,(Ingentia,Antetonitrus)))),((Schleitheimia,(Isanosaurus,((Tazoudasaurus,Vulcanodon),((Shunosaurus,(Spinophorosaurus,(Mamenchisaurus,(Omeisaurus,(Cetiosaurus,Neosauropoda))))),(Patagosaurus,Barapasaurus))))),(Pulanesaura,Gongxianosaurus)))))))),(Irisosaurus,(Mussaurus,Leonerasaurus))))),(Xingxiulong,(Jingshanosaurus,Chuxiongosaurus)))))),(Eucnemesaurus_fortis,(Riojasaurus,Eucnemesaurus_entaxonis)))),(Plateosaurus_gracilis,(Plateosaurus_ingens,Plateosaurus_engelhardti))))))))))))),(Buriolestes,Pampadromaeus))),(Staurikosaurus,Herrerasaurus))))))));

(Euparkeria,(Crurotarsi,(Marasuchus,(Silesaurus,(Agnosphitys,(Ornithischia,(((Guaibasaurus,(Neotheropoda,Chindesaurus)),(Buriolestes,(Pampadromaeus,(Panphagia,(Eoraptor,((Saturnalia,Chromogisaurus),(Bagualosaurus,(Jaklapalisaurus,(Nambalia,((Thecodontosaurus,Pantydraco),(Efraasia,(Plateosauravus,(Ruehleia,(((Unaysaurus,(Pradhania,Macrocollum)),((Sarahsaurus,((Ngwevu,((Coloradisaurus,(Glacialisaurus,Lufengosaurus)),(Xixipiosaurus,(Massospondylus_carinatus,(Adeopapposaurus,Leyesaurus))))),(Yunnanosaurus_huangi,((Seitaad,(Anchisaurus,((Yizhousaurus,((Sefapanosaurus,Aardonyx),(NMQR1551,(NMRQ3314,(Blikanasaurus,((Camelotia,((Meroktenos,(Ledumahadi,Kholumolumo)),(Lessemsaurus,(Ingentia,Antetonitrus)))),((Schleitheimia,(Isanosaurus,((Tazoudasaurus,Vulcanodon),((Shunosaurus,(Spinophorosaurus,(Mamenchisaurus,(Omeisaurus,(Cetiosaurus,Neosauropoda))))),(Patagosaurus,Barapasaurus))))),(Pulanesaura,Gongxianosaurus)))))))),(Irisosaurus,(Mussaurus,Leonerasaurus))))),(Xingxiulong,(Jingshanosaurus,Chuxiongosaurus)))))),(Eucnemesaurus_fortis,(Riojasaurus,Eucnemesaurus_entaxonis)))),(Plateosaurus_gracilis,(Plateosaurus_ingens,Plateosaurus_engelhardti)))))))))))))))),(Staurikosaurus,Herrerasaurus))))))));

(Euparkeria,(Crurotarsi,(Marasuchus,(Silesaurus,(Agnosphitys,(Ornithischia,(((Guaibasaurus,(Neotheropoda,Chindesaurus)),(Buriolestes,(Pampadromaeus,(Panphagia,(Eoraptor,((Saturnalia,Chromogisaurus),(Bagualosaurus,(Jaklapalisaurus,(Nambalia,((Thecodontosaurus,Pantydraco),(Efraasia,(Plateosauravus,(Ruehleia,(((Unaysaurus,(Pradhania,Macrocollum)),((Sarahsaurus,((Ngwevu,(Xixipiosaurus,((Coloradisaurus,(Glacialisaurus,Lufengosaurus)),(Massospondylus_carinatus,(Adeopapposaurus,Leyesaurus))))),(Yunnanosaurus_huangi,((Seitaad,(Anchisaurus,((Yizhousaurus,((Sefapanosaurus,Aardonyx),(NMQR1551,(NMRQ3314,(Blikanasaurus,((Camelotia,((Meroktenos,(Ledumahadi,Kholumolumo)),(Lessemsaurus,(Ingentia,Antetonitrus)))),((Schleitheimia,(Isanosaurus,((Tazoudasaurus,Vulcanodon),((Shunosaurus,(Spinophorosaurus,(Omeisaurus,(Mamenchisaurus,(Cetiosaurus,Neosauropoda))))),(Patagosaurus,Barapasaurus))))),(Pulanesaura,Gongxianosaurus)))))))),(Irisosaurus,(Mussaurus,Leonerasaurus))))),(Xingxiulong,(Jingshanosaurus,Chuxiongosaurus)))))),(Eucnemesaurus_fortis,(Riojasaurus,Eucnemesaurus_entaxonis)))),(Plateosaurus_gracilis,(Plateosaurus_ingens,Plateosaurus_engelhardti)))))))))))))))),(Staurikosaurus,Herrerasaurus))))))));

(Euparkeria,(Crurotarsi,(Marasuchus,(Silesaurus,(Agnosphitys,(Ornithischia,(((Guaibasaurus,(Neotheropoda,Chindesaurus)),(Buriolestes,(Pampadromaeus,(Panphagia,(Eoraptor,((Saturnalia,Chromogisaurus),(Bagualosaurus,(Jaklapalisaurus,(Nambalia,((Thecodontosaurus,Pantydraco),(Efraasia,(Plateosauravus,(Ruehleia,(((Unaysaurus,(Pradhania,Macrocollum)),((Sarahsaurus,((Ngwevu,(Xixipiosaurus,((Coloradisaurus,(Glacialisaurus,Lufengosaurus)),(Massospondylus_carinatus,(Adeopapposaurus,Leyesaurus))))),(Yunnanosaurus_huangi,((Seitaad,(Anchisaurus,((Yizhousaurus,((Sefapanosaurus,Aardonyx),(NMQR1551,(NMRQ3314,(Blikanasaurus,((Camelotia,((Meroktenos,(Ledumahadi,Kholumolumo)),(Lessemsaurus,(Ingentia,Antetonitrus)))),((Schleitheimia,(Isanosaurus,((Tazoudasaurus,Vulcanodon),((Shunosaurus,((Spinophorosaurus,Omeisaurus),(Mamenchisaurus,(Cetiosaurus,Neosauropoda)))),(Patagosaurus,Barapasaurus))))),(Pulanesaura,Gongxianosaurus)))))))),(Irisosaurus,(Mussaurus,Leonerasaurus))))),(Xingxiulong,(Jingshanosaurus,Chuxiongosaurus)))))),(Eucnemesaurus_fortis,(Riojasaurus,Eucnemesaurus_entaxonis)))),(Plateosaurus_gracilis,(Plateosaurus_ingens,Plateosaurus_engelhardti)))))))))))))))),(Staurikosaurus,Herrerasaurus))))))));

(Euparkeria,(Crurotarsi,(Marasuchus,(Silesaurus,(Agnosphitys,(Ornithischia,(((Guaibasaurus,(Neotheropoda,Chindesaurus)),(Buriolestes,(Pampadromaeus,(Panphagia,(Eoraptor,((Saturnalia,Chromogisaurus),(Bagualosaurus,(Jaklapalisaurus,(Nambalia,((Thecodontosaurus,Pantydraco),(Efraasia,(Plateosauravus,(Ruehleia,(((Unaysaurus,(Pradhania,Macrocollum)),((Sarahsaurus,((Ngwevu,(Xixipiosaurus,((Coloradisaurus,(Glacialisaurus,Lufengosaurus)),(Massospondylus_carinatus,(Adeopapposaurus,Leyesaurus))))),(Yunnanosaurus_huangi,((Seitaad,(Anchisaurus,((Yizhousaurus,((Sefapanosaurus,Aardonyx),(NMQR1551,(NMRQ3314,(Blikanasaurus,((Camelotia,((Meroktenos,(Ledumahadi,Kholumolumo)),(Lessemsaurus,(Ingentia,Antetonitrus)))),((Schleitheimia,(Isanosaurus,((Tazoudasaurus,Vulcanodon),((Shunosaurus,((Spinophorosaurus,(Omeisaurus,Mamenchisaurus)),(Cetiosaurus,Neosauropoda))),(Patagosaurus,Barapasaurus))))),(Pulanesaura,Gongxianosaurus)))))))),(Irisosaurus,(Mussaurus,Leonerasaurus))))),(Xingxiulong,(Jingshanosaurus,Chuxiongosaurus)))))),(Eucnemesaurus_fortis,(Riojasaurus,Eucnemesaurus_entaxonis)))),(Plateosaurus_gracilis,(Plateosaurus_ingens,Plateosaurus_engelhardti)))))))))))))))),(Staurikosaurus,Herrerasaurus))))))));

(Euparkeria,(Crurotarsi,(Marasuchus,(Silesaurus,(Ornithischia,(Agnosphitys,(((Guaibasaurus,(Neotheropoda,Chindesaurus)),(Buriolestes,(Pampadromaeus,(Panphagia,(Eoraptor,((Saturnalia,Chromogisaurus),(Bagualosaurus,(Jaklapalisaurus,(Nambalia,((Thecodontosaurus,Pantydraco),(Efraasia,(Plateosauravus,(Ruehleia,(((Unaysaurus,(Pradhania,Macrocollum)),((Sarahsaurus,((Ngwevu,(Xixipiosaurus,((Coloradisaurus,(Glacialisaurus,Lufengosaurus)),(Massospondylus_carinatus,(Adeopapposaurus,Leyesaurus))))),(Yunnanosaurus_huangi,((Seitaad,(Anchisaurus,((Yizhousaurus,((Sefapanosaurus,Aardonyx),(NMQR1551,(NMRQ3314,(Blikanasaurus,((Camelotia,((Meroktenos,(Ledumahadi,Kholumolumo)),(Lessemsaurus,(Ingentia,Antetonitrus)))),(Pulanesaura,(Gongxianosaurus,(Schleitheimia,(Isanosaurus,((Tazoudasaurus,Vulcanodon),((Shunosaurus,((Spinophorosaurus,(Omeisaurus,Mamenchisaurus)),(Cetiosaurus,Neosauropoda))),(Patagosaurus,Barapasaurus))))))))))))),(Irisosaurus,(Mussaurus,Leonerasaurus))))),(Xingxiulong,(Jingshanosaurus,Chuxiongosaurus)))))),(Eucnemesaurus_fortis,(Riojasaurus,Eucnemesaurus_entaxonis)))),(Plateosaurus_gracilis,(Plateosaurus_ingens,Plateosaurus_engelhardti)))))))))))))))),(Staurikosaurus,Herrerasaurus))))))));

(Euparkeria,(Crurotarsi,(Marasuchus,(Silesaurus,(Ornithischia,((Agnosphitys,((Guaibasaurus,(Neotheropoda,Chindesaurus)),(Buriolestes,(Pampadromaeus,(Panphagia,(Eoraptor,((Saturnalia,Chromogisaurus),(Bagualosaurus,(Jaklapalisaurus,(Nambalia,((Thecodontosaurus,Pantydraco),(Efraasia,(Plateosauravus,(Ruehleia,(((Unaysaurus,(Pradhania,Macrocollum)),((Sarahsaurus,((Ngwevu,(Xixipiosaurus,((Coloradisaurus,(Glacialisaurus,Lufengosaurus)),(Massospondylus_carinatus,(Adeopapposaurus,Leyesaurus))))),(Yunnanosaurus_huangi,((Seitaad,(Anchisaurus,((Yizhousaurus,((Sefapanosaurus,Aardonyx),(NMQR1551,(NMRQ3314,(Blikanasaurus,((Camelotia,((Meroktenos,(Ledumahadi,Kholumolumo)),(Lessemsaurus,(Ingentia,Antetonitrus)))),(Pulanesaura,(Gongxianosaurus,(Schleitheimia,(Isanosaurus,((Tazoudasaurus,Vulcanodon),((Shunosaurus,((Spinophorosaurus,(Omeisaurus,Mamenchisaurus)),(Cetiosaurus,Neosauropoda))),(Patagosaurus,Barapasaurus))))))))))))),(Irisosaurus,(Mussaurus,Leonerasaurus))))),(Xingxiulong,(Jingshanosaurus,Chuxiongosaurus)))))),(Eucnemesaurus_fortis,(Riojasaurus,Eucnemesaurus_entaxonis)))),(Plateosaurus_gracilis,(Plateosaurus_ingens,Plateosaurus_engelhardti))))))))))))))))),(Staurikosaurus,Herrerasaurus)))))));

(Euparkeria,(Crurotarsi,(Marasuchus,(Silesaurus,(Agnosphitys,(Ornithischia,(((Guaibasaurus,(Neotheropoda,Chindesaurus)),((Panphagia,(Eoraptor,((Saturnalia,Chromogisaurus),(Bagualosaurus,(Jaklapalisaurus,(Nambalia,((Thecodontosaurus,Pantydraco),(Efraasia,(Plateosauravus,(Ruehleia,(((Unaysaurus,(Pradhania,Macrocollum)),((Sarahsaurus,((Ngwevu,(Xixipiosaurus,((Coloradisaurus,(Glacialisaurus,Lufengosaurus)),(Massospondylus_carinatus,(Adeopapposaurus,Leyesaurus))))),(Yunnanosaurus_huangi,((Seitaad,(Anchisaurus,((Yizhousaurus,((Sefapanosaurus,Aardonyx),(NMQR1551,(NMRQ3314,(Blikanasaurus,((Camelotia,((Meroktenos,(Ledumahadi,Kholumolumo)),(Lessemsaurus,(Ingentia,Antetonitrus)))),(Pulanesaura,(Gongxianosaurus,(Schleitheimia,(Isanosaurus,((Tazoudasaurus,Vulcanodon),((Shunosaurus,((Spinophorosaurus,(Omeisaurus,Mamenchisaurus)),(Cetiosaurus,Neosauropoda))),(Patagosaurus,Barapasaurus))))))))))))),(Irisosaurus,(Mussaurus,Leonerasaurus))))),(Xingxiulong,(Jingshanosaurus,Chuxiongosaurus)))))),(Eucnemesaurus_fortis,(Riojasaurus,Eucnemesaurus_entaxonis)))),(Plateosaurus_gracilis,(Plateosaurus_ingens,Plateosaurus_engelhardti))))))))))))),(Buriolestes,Pampadromaeus))),(Staurikosaurus,Herrerasaurus))))))));

(Euparkeria,(Crurotarsi,(Marasuchus,(Silesaurus,(Agnosphitys,(Ornithischia,(((Guaibasaurus,(Neotheropoda,Chindesaurus)),(Buriolestes,(Pampadromaeus,(Panphagia,(Eoraptor,((Saturnalia,Chromogisaurus),(Bagualosaurus,(Jaklapalisaurus,(Nambalia,((Thecodontosaurus,Pantydraco),(Efraasia,(Plateosauravus,(Ruehleia,(((Unaysaurus,(Pradhania,Macrocollum)),((Sarahsaurus,((Ngwevu,((Coloradisaurus,(Glacialisaurus,Lufengosaurus)),(Xixipiosaurus,(Massospondylus_carinatus,(Adeopapposaurus,Leyesaurus))))),(Yunnanosaurus_huangi,((Seitaad,(Anchisaurus,((Yizhousaurus,((Sefapanosaurus,Aardonyx),(NMQR1551,(NMRQ3314,(Blikanasaurus,((Camelotia,((Meroktenos,(Ledumahadi,Kholumolumo)),(Lessemsaurus,(Ingentia,Antetonitrus)))),(Pulanesaura,(Gongxianosaurus,(Schleitheimia,(Isanosaurus,((Tazoudasaurus,Vulcanodon),((Shunosaurus,((Spinophorosaurus,(Omeisaurus,Mamenchisaurus)),(Cetiosaurus,Neosauropoda))),(Patagosaurus,Barapasaurus))))))))))))),(Irisosaurus,(Mussaurus,Leonerasaurus))))),(Xingxiulong,(Jingshanosaurus,Chuxiongosaurus)))))),(Eucnemesaurus_fortis,(Riojasaurus,Eucnemesaurus_entaxonis)))),(Plateosaurus_gracilis,(Plateosaurus_ingens,Plateosaurus_engelhardti)))))))))))))))),(Staurikosaurus,Herrerasaurus))))))));

(Euparkeria,(Crurotarsi,(Marasuchus,(Silesaurus,(Ornithischia,(Agnosphitys,(((Guaibasaurus,(Neotheropoda,Chindesaurus)),((Panphagia,(Eoraptor,((Saturnalia,Chromogisaurus),(Bagualosaurus,(Jaklapalisaurus,(Nambalia,((Thecodontosaurus,Pantydraco),(Efraasia,(Plateosauravus,(Ruehleia,(((Unaysaurus,(Pradhania,Macrocollum)),((Sarahsaurus,((Ngwevu,(Xixipiosaurus,((Coloradisaurus,(Glacialisaurus,Lufengosaurus)),(Massospondylus_carinatus,(Adeopapposaurus,Leyesaurus))))),(Yunnanosaurus_huangi,((Seitaad,(Anchisaurus,((Yizhousaurus,((Sefapanosaurus,Aardonyx),(NMQR1551,(NMRQ3314,(Blikanasaurus,((Camelotia,((Meroktenos,(Ledumahadi,Kholumolumo)),(Lessemsaurus,(Ingentia,Antetonitrus)))),(Pulanesaura,(Gongxianosaurus,(Schleitheimia,(Isanosaurus,((Tazoudasaurus,Vulcanodon),((Shunosaurus,(Spinophorosaurus,(Mamenchisaurus,(Omeisaurus,(Cetiosaurus,Neosauropoda))))),(Patagosaurus,Barapasaurus))))))))))))),(Irisosaurus,(Mussaurus,Leonerasaurus))))),(Xingxiulong,(Jingshanosaurus,Chuxiongosaurus)))))),(Eucnemesaurus_fortis,(Riojasaurus,Eucnemesaurus_entaxonis)))),(Plateosaurus_gracilis,(Plateosaurus_ingens,Plateosaurus_engelhardti))))))))))))),(Buriolestes,Pampadromaeus))),(Staurikosaurus,Herrerasaurus))))))));

(Euparkeria,(Crurotarsi,(Marasuchus,(Silesaurus,(Ornithischia,(Agnosphitys,(((Guaibasaurus,(Neotheropoda,Chindesaurus)),(Buriolestes,(Pampadromaeus,(Panphagia,(Eoraptor,((Saturnalia,Chromogisaurus),(Bagualosaurus,(Jaklapalisaurus,(Nambalia,((Thecodontosaurus,Pantydraco),(Efraasia,(Plateosauravus,(Ruehleia,(((Unaysaurus,(Pradhania,Macrocollum)),((Sarahsaurus,((Ngwevu,((Coloradisaurus,(Glacialisaurus,Lufengosaurus)),(Xixipiosaurus,(Massospondylus_carinatus,(Adeopapposaurus,Leyesaurus))))),(Yunnanosaurus_huangi,((Seitaad,(Anchisaurus,((Yizhousaurus,((Sefapanosaurus,Aardonyx),(NMQR1551,(NMRQ3314,(Blikanasaurus,((Camelotia,((Meroktenos,(Ledumahadi,Kholumolumo)),(Lessemsaurus,(Ingentia,Antetonitrus)))),(Pulanesaura,(Gongxianosaurus,(Schleitheimia,(Isanosaurus,((Tazoudasaurus,Vulcanodon),((Shunosaurus,(Spinophorosaurus,(Mamenchisaurus,(Omeisaurus,(Cetiosaurus,Neosauropoda))))),(Patagosaurus,Barapasaurus))))))))))))),(Irisosaurus,(Mussaurus,Leonerasaurus))))),(Xingxiulong,(Jingshanosaurus,Chuxiongosaurus)))))),(Eucnemesaurus_fortis,(Riojasaurus,Eucnemesaurus_entaxonis)))),(Plateosaurus_gracilis,(Plateosaurus_ingens,Plateosaurus_engelhardti)))))))))))))))),(Staurikosaurus,Herrerasaurus))))))));

(Euparkeria,(Crurotarsi,(Marasuchus,(Silesaurus,(Ornithischia,(Agnosphitys,(((Guaibasaurus,(Neotheropoda,Chindesaurus)),(Buriolestes,(Pampadromaeus,(Panphagia,(Eoraptor,((Saturnalia,Chromogisaurus),(Bagualosaurus,(Jaklapalisaurus,(Nambalia,((Thecodontosaurus,Pantydraco),(Efraasia,(Plateosauravus,(Ruehleia,(((Unaysaurus,(Pradhania,Macrocollum)),((Sarahsaurus,((Ngwevu,(Xixipiosaurus,((Coloradisaurus,(Glacialisaurus,Lufengosaurus)),(Massospondylus_carinatus,(Adeopapposaurus,Leyesaurus))))),(Yunnanosaurus_huangi,((Seitaad,(Anchisaurus,((Yizhousaurus,((Sefapanosaurus,Aardonyx),(NMQR1551,(NMRQ3314,(Blikanasaurus,((Camelotia,((Meroktenos,(Ledumahadi,Kholumolumo)),(Lessemsaurus,(Ingentia,Antetonitrus)))),(Pulanesaura,(Gongxianosaurus,(Schleitheimia,(Isanosaurus,((Tazoudasaurus,Vulcanodon),((Shunosaurus,(Spinophorosaurus,(Omeisaurus,(Mamenchisaurus,(Cetiosaurus,Neosauropoda))))),(Patagosaurus,Barapasaurus))))))))))))),(Irisosaurus,(Mussaurus,Leonerasaurus))))),(Xingxiulong,(Jingshanosaurus,Chuxiongosaurus)))))),(Eucnemesaurus_fortis,(Riojasaurus,Eucnemesaurus_entaxonis)))),(Plateosaurus_gracilis,(Plateosaurus_ingens,Plateosaurus_engelhardti)))))))))))))))),(Staurikosaurus,Herrerasaurus))))))));

(Euparkeria,(Crurotarsi,(Marasuchus,(Silesaurus,(Ornithischia,(Agnosphitys,(((Guaibasaurus,(Neotheropoda,Chindesaurus)),(Buriolestes,(Pampadromaeus,(Panphagia,(Eoraptor,((Saturnalia,Chromogisaurus),(Bagualosaurus,(Jaklapalisaurus,(Nambalia,((Thecodontosaurus,Pantydraco),(Efraasia,(Plateosauravus,(Ruehleia,(((Unaysaurus,(Pradhania,Macrocollum)),((Sarahsaurus,((Ngwevu,(Xixipiosaurus,((Coloradisaurus,(Glacialisaurus,Lufengosaurus)),(Massospondylus_carinatus,(Adeopapposaurus,Leyesaurus))))),(Yunnanosaurus_huangi,((Seitaad,(Anchisaurus,((Yizhousaurus,((Sefapanosaurus,Aardonyx),(NMQR1551,(NMRQ3314,(Blikanasaurus,((Camelotia,((Meroktenos,(Ledumahadi,Kholumolumo)),(Lessemsaurus,(Ingentia,Antetonitrus)))),(Pulanesaura,(Gongxianosaurus,(Schleitheimia,(Isanosaurus,((Tazoudasaurus,Vulcanodon),((Shunosaurus,((Spinophorosaurus,Omeisaurus),(Mamenchisaurus,(Cetiosaurus,Neosauropoda)))),(Patagosaurus,Barapasaurus))))))))))))),(Irisosaurus,(Mussaurus,Leonerasaurus))))),(Xingxiulong,(Jingshanosaurus,Chuxiongosaurus)))))),(Eucnemesaurus_fortis,(Riojasaurus,Eucnemesaurus_entaxonis)))),(Plateosaurus_gracilis,(Plateosaurus_ingens,Plateosaurus_engelhardti)))))))))))))))),(Staurikosaurus,Herrerasaurus))))))));

(Euparkeria,(Crurotarsi,(Marasuchus,(Silesaurus,(Ornithischia,((Agnosphitys,((Guaibasaurus,(Neotheropoda,Chindesaurus)),((Panphagia,(Eoraptor,((Saturnalia,Chromogisaurus),(Bagualosaurus,(Jaklapalisaurus,(Nambalia,((Thecodontosaurus,Pantydraco),(Efraasia,(Plateosauravus,(Ruehleia,(((Unaysaurus,(Pradhania,Macrocollum)),((Sarahsaurus,((Ngwevu,(Xixipiosaurus,((Coloradisaurus,(Glacialisaurus,Lufengosaurus)),(Massospondylus_carinatus,(Adeopapposaurus,Leyesaurus))))),(Yunnanosaurus_huangi,((Seitaad,(Anchisaurus,((Yizhousaurus,((Sefapanosaurus,Aardonyx),(NMQR1551,(NMRQ3314,(Blikanasaurus,((Camelotia,((Meroktenos,(Ledumahadi,Kholumolumo)),(Lessemsaurus,(Ingentia,Antetonitrus)))),(Pulanesaura,(Gongxianosaurus,(Schleitheimia,(Isanosaurus,((Tazoudasaurus,Vulcanodon),((Shunosaurus,(Spinophorosaurus,(Mamenchisaurus,(Omeisaurus,(Cetiosaurus,Neosauropoda))))),(Patagosaurus,Barapasaurus))))))))))))),(Irisosaurus,(Mussaurus,Leonerasaurus))))),(Xingxiulong,(Jingshanosaurus,Chuxiongosaurus)))))),(Eucnemesaurus_fortis,(Riojasaurus,Eucnemesaurus_entaxonis)))),(Plateosaurus_gracilis,(Plateosaurus_ingens,Plateosaurus_engelhardti))))))))))))),(Buriolestes,Pampadromaeus)))),(Staurikosaurus,Herrerasaurus)))))));

(Euparkeria,(Crurotarsi,(Marasuchus,(Silesaurus,(Ornithischia,((Agnosphitys,((Guaibasaurus,(Neotheropoda,Chindesaurus)),(Buriolestes,((Eoraptor,((Saturnalia,Chromogisaurus),(Bagualosaurus,(Jaklapalisaurus,(Nambalia,((Thecodontosaurus,Pantydraco),(Efraasia,(Plateosauravus,(Ruehleia,(((Unaysaurus,(Pradhania,Macrocollum)),((Sarahsaurus,((Ngwevu,(Xixipiosaurus,((Coloradisaurus,(Glacialisaurus,Lufengosaurus)),(Massospondylus_carinatus,(Adeopapposaurus,Leyesaurus))))),(Yunnanosaurus_huangi,((Seitaad,(Anchisaurus,((Yizhousaurus,((Sefapanosaurus,Aardonyx),(NMQR1551,(NMRQ3314,(Blikanasaurus,((Camelotia,((Meroktenos,(Ledumahadi,Kholumolumo)),(Lessemsaurus,(Ingentia,Antetonitrus)))),(Pulanesaura,(Gongxianosaurus,(Schleitheimia,(Isanosaurus,((Tazoudasaurus,Vulcanodon),((Shunosaurus,(Spinophorosaurus,(Mamenchisaurus,(Omeisaurus,(Cetiosaurus,Neosauropoda))))),(Patagosaurus,Barapasaurus))))))))))))),(Irisosaurus,(Mussaurus,Leonerasaurus))))),(Xingxiulong,(Jingshanosaurus,Chuxiongosaurus)))))),(Eucnemesaurus_fortis,(Riojasaurus,Eucnemesaurus_entaxonis)))),(Plateosaurus_gracilis,(Plateosaurus_ingens,Plateosaurus_engelhardti)))))))))))),(Panphagia,Pampadromaeus))))),(Staurikosaurus,Herrerasaurus)))))));

(Euparkeria,(Crurotarsi,(Marasuchus,(Silesaurus,(Ornithischia,((Agnosphitys,((Guaibasaurus,(Neotheropoda,Chindesaurus)),(Buriolestes,(Panphagia,(Eoraptor,((Saturnalia,Chromogisaurus),(Pampadromaeus,(Bagualosaurus,(Jaklapalisaurus,(Nambalia,((Thecodontosaurus,Pantydraco),(Efraasia,(Plateosauravus,(Ruehleia,(((Unaysaurus,(Pradhania,Macrocollum)),((Sarahsaurus,((Ngwevu,(Xixipiosaurus,((Coloradisaurus,(Glacialisaurus,Lufengosaurus)),(Massospondylus_carinatus,(Adeopapposaurus,Leyesaurus))))),(Yunnanosaurus_huangi,((Seitaad,(Anchisaurus,((Yizhousaurus,((Sefapanosaurus,Aardonyx),(NMQR1551,(NMRQ3314,(Blikanasaurus,((Camelotia,((Meroktenos,(Ledumahadi,Kholumolumo)),(Lessemsaurus,(Ingentia,Antetonitrus)))),(Pulanesaura,(Gongxianosaurus,(Schleitheimia,(Isanosaurus,((Tazoudasaurus,Vulcanodon),((Shunosaurus,(Spinophorosaurus,(Mamenchisaurus,(Omeisaurus,(Cetiosaurus,Neosauropoda))))),(Patagosaurus,Barapasaurus))))))))))))),(Irisosaurus,(Mussaurus,Leonerasaurus))))),(Xingxiulong,(Jingshanosaurus,Chuxiongosaurus)))))),(Eucnemesaurus_fortis,(Riojasaurus,Eucnemesaurus_entaxonis)))),(Plateosaurus_gracilis,(Plateosaurus_ingens,Plateosaurus_engelhardti))))))))))))))))),(Staurikosaurus,Herrerasaurus)))))));

(Euparkeria,(Crurotarsi,(Marasuchus,(Silesaurus,(Ornithischia,((Agnosphitys,((Guaibasaurus,(Neotheropoda,Chindesaurus)),(Buriolestes,(Panphagia,(Eoraptor,((Pampadromaeus,(Saturnalia,Chromogisaurus)),(Bagualosaurus,(Jaklapalisaurus,(Nambalia,((Thecodontosaurus,Pantydraco),(Efraasia,(Plateosauravus,(Ruehleia,(((Unaysaurus,(Pradhania,Macrocollum)),((Sarahsaurus,((Ngwevu,(Xixipiosaurus,((Coloradisaurus,(Glacialisaurus,Lufengosaurus)),(Massospondylus_carinatus,(Adeopapposaurus,Leyesaurus))))),(Yunnanosaurus_huangi,((Seitaad,(Anchisaurus,((Yizhousaurus,((Sefapanosaurus,Aardonyx),(NMQR1551,(NMRQ3314,(Blikanasaurus,((Camelotia,((Meroktenos,(Ledumahadi,Kholumolumo)),(Lessemsaurus,(Ingentia,Antetonitrus)))),(Pulanesaura,(Gongxianosaurus,(Schleitheimia,(Isanosaurus,((Tazoudasaurus,Vulcanodon),((Shunosaurus,(Spinophorosaurus,(Mamenchisaurus,(Omeisaurus,(Cetiosaurus,Neosauropoda))))),(Patagosaurus,Barapasaurus))))))))))))),(Irisosaurus,(Mussaurus,Leonerasaurus))))),(Xingxiulong,(Jingshanosaurus,Chuxiongosaurus)))))),(Eucnemesaurus_fortis,(Riojasaurus,Eucnemesaurus_entaxonis)))),(Plateosaurus_gracilis,(Plateosaurus_ingens,Plateosaurus_engelhardti)))))))))))))))),(Staurikosaurus,Herrerasaurus)))))));

(Euparkeria,(Crurotarsi,(Marasuchus,(Silesaurus,(Ornithischia,((Agnosphitys,((Guaibasaurus,(Neotheropoda,Chindesaurus)),(Buriolestes,(Pampadromaeus,(Panphagia,(Eoraptor,((Saturnalia,Chromogisaurus),(Bagualosaurus,(Jaklapalisaurus,(Nambalia,((Thecodontosaurus,Pantydraco),(Efraasia,(Plateosauravus,(Ruehleia,(((Unaysaurus,(Pradhania,Macrocollum)),((Sarahsaurus,((Ngwevu,((Coloradisaurus,(Glacialisaurus,Lufengosaurus)),(Xixipiosaurus,(Massospondylus_carinatus,(Adeopapposaurus,Leyesaurus))))),(Yunnanosaurus_huangi,((Seitaad,(Anchisaurus,((Yizhousaurus,((Sefapanosaurus,Aardonyx),(NMQR1551,(NMRQ3314,(Blikanasaurus,((Camelotia,((Meroktenos,(Ledumahadi,Kholumolumo)),(Lessemsaurus,(Ingentia,Antetonitrus)))),(Pulanesaura,(Gongxianosaurus,(Schleitheimia,(Isanosaurus,((Tazoudasaurus,Vulcanodon),((Shunosaurus,(Spinophorosaurus,(Mamenchisaurus,(Omeisaurus,(Cetiosaurus,Neosauropoda))))),(Patagosaurus,Barapasaurus))))))))))))),(Irisosaurus,(Mussaurus,Leonerasaurus))))),(Xingxiulong,(Jingshanosaurus,Chuxiongosaurus)))))),(Eucnemesaurus_fortis,(Riojasaurus,Eucnemesaurus_entaxonis)))),(Plateosaurus_gracilis,(Plateosaurus_ingens,Plateosaurus_engelhardti))))))))))))))))),(Staurikosaurus,Herrerasaurus)))))));

(Euparkeria,(Crurotarsi,(Marasuchus,(Silesaurus,(Ornithischia,((Agnosphitys,((Guaibasaurus,(Neotheropoda,Chindesaurus)),(Buriolestes,(Pampadromaeus,(Panphagia,(Eoraptor,((Saturnalia,Chromogisaurus),(Bagualosaurus,(Jaklapalisaurus,(Nambalia,((Thecodontosaurus,Pantydraco),(Efraasia,(Plateosauravus,(Ruehleia,(((Unaysaurus,(Pradhania,Macrocollum)),((Sarahsaurus,((Ngwevu,(Xixipiosaurus,((Coloradisaurus,(Glacialisaurus,Lufengosaurus)),(Massospondylus_carinatus,(Adeopapposaurus,Leyesaurus))))),(Yunnanosaurus_huangi,((Seitaad,(Anchisaurus,((Yizhousaurus,((Sefapanosaurus,Aardonyx),(NMQR1551,(NMRQ3314,(Blikanasaurus,((Camelotia,((Meroktenos,(Ledumahadi,Kholumolumo)),(Lessemsaurus,(Ingentia,Antetonitrus)))),(Pulanesaura,(Gongxianosaurus,(Schleitheimia,(Isanosaurus,((Tazoudasaurus,Vulcanodon),((Shunosaurus,(Spinophorosaurus,(Omeisaurus,(Mamenchisaurus,(Cetiosaurus,Neosauropoda))))),(Patagosaurus,Barapasaurus))))))))))))),(Irisosaurus,(Mussaurus,Leonerasaurus))))),(Xingxiulong,(Jingshanosaurus,Chuxiongosaurus)))))),(Eucnemesaurus_fortis,(Riojasaurus,Eucnemesaurus_entaxonis)))),(Plateosaurus_gracilis,(Plateosaurus_ingens,Plateosaurus_engelhardti))))))))))))))))),(Staurikosaurus,Herrerasaurus)))))));

(Euparkeria,(Crurotarsi,(Marasuchus,(Silesaurus,(Ornithischia,((Agnosphitys,((Guaibasaurus,(Neotheropoda,Chindesaurus)),(Buriolestes,(Pampadromaeus,(Panphagia,(Eoraptor,((Saturnalia,Chromogisaurus),(Bagualosaurus,(Jaklapalisaurus,(Nambalia,((Thecodontosaurus,Pantydraco),(Efraasia,(Plateosauravus,(Ruehleia,(((Unaysaurus,(Pradhania,Macrocollum)),((Sarahsaurus,((Ngwevu,(Xixipiosaurus,((Coloradisaurus,(Glacialisaurus,Lufengosaurus)),(Massospondylus_carinatus,(Adeopapposaurus,Leyesaurus))))),(Yunnanosaurus_huangi,((Seitaad,(Anchisaurus,((Yizhousaurus,((Sefapanosaurus,Aardonyx),(NMQR1551,(NMRQ3314,(Blikanasaurus,((Camelotia,((Meroktenos,(Ledumahadi,Kholumolumo)),(Lessemsaurus,(Ingentia,Antetonitrus)))),(Pulanesaura,(Gongxianosaurus,(Schleitheimia,(Isanosaurus,((Tazoudasaurus,Vulcanodon),((Shunosaurus,((Spinophorosaurus,Omeisaurus),(Mamenchisaurus,(Cetiosaurus,Neosauropoda)))),(Patagosaurus,Barapasaurus))))))))))))),(Irisosaurus,(Mussaurus,Leonerasaurus))))),(Xingxiulong,(Jingshanosaurus,Chuxiongosaurus)))))),(Eucnemesaurus_fortis,(Riojasaurus,Eucnemesaurus_entaxonis)))),(Plateosaurus_gracilis,(Plateosaurus_ingens,Plateosaurus_engelhardti))))))))))))))))),(Staurikosaurus,Herrerasaurus)))))));

(Euparkeria,(Crurotarsi,(Marasuchus,(Silesaurus,(Ornithischia,((Agnosphitys,((Guaibasaurus,(Neotheropoda,Chindesaurus)),((Eoraptor,((Saturnalia,Chromogisaurus),(Bagualosaurus,(Jaklapalisaurus,(Nambalia,((Thecodontosaurus,Pantydraco),(Efraasia,(Plateosauravus,(Ruehleia,(((Unaysaurus,(Pradhania,Macrocollum)),((Sarahsaurus,((Ngwevu,(Xixipiosaurus,((Coloradisaurus,(Glacialisaurus,Lufengosaurus)),(Massospondylus_carinatus,(Adeopapposaurus,Leyesaurus))))),(Yunnanosaurus_huangi,((Seitaad,(Anchisaurus,((Yizhousaurus,((Sefapanosaurus,Aardonyx),(NMQR1551,(NMRQ3314,(Blikanasaurus,((Camelotia,((Meroktenos,(Ledumahadi,Kholumolumo)),(Lessemsaurus,(Ingentia,Antetonitrus)))),(Pulanesaura,(Gongxianosaurus,(Schleitheimia,(Isanosaurus,((Tazoudasaurus,Vulcanodon),((Shunosaurus,(Spinophorosaurus,(Mamenchisaurus,(Omeisaurus,(Cetiosaurus,Neosauropoda))))),(Patagosaurus,Barapasaurus))))))))))))),(Irisosaurus,(Mussaurus,Leonerasaurus))))),(Xingxiulong,(Jingshanosaurus,Chuxiongosaurus)))))),(Eucnemesaurus_fortis,(Riojasaurus,Eucnemesaurus_entaxonis)))),(Plateosaurus_gracilis,(Plateosaurus_ingens,Plateosaurus_engelhardti)))))))))))),(Buriolestes,(Panphagia,Pampadromaeus))))),(Staurikosaurus,Herrerasaurus)))))));

(Euparkeria,(Crurotarsi,(Marasuchus,(Silesaurus,(Agnosphitys,(Ornithischia,(((Guaibasaurus,(Neotheropoda,Chindesaurus)),((Panphagia,(Eoraptor,((Saturnalia,Chromogisaurus),(Bagualosaurus,(Jaklapalisaurus,(Nambalia,((Thecodontosaurus,Pantydraco),(Efraasia,(Plateosauravus,(Ruehleia,(((Unaysaurus,(Pradhania,Macrocollum)),((Sarahsaurus,((Ngwevu,((Coloradisaurus,(Glacialisaurus,Lufengosaurus)),(Xixipiosaurus,(Massospondylus_carinatus,(Adeopapposaurus,Leyesaurus))))),(Yunnanosaurus_huangi,((Seitaad,(Anchisaurus,((Yizhousaurus,((Sefapanosaurus,Aardonyx),(NMQR1551,(NMRQ3314,(Blikanasaurus,((Camelotia,((Meroktenos,(Ledumahadi,Kholumolumo)),(Lessemsaurus,(Ingentia,Antetonitrus)))),(Pulanesaura,(Gongxianosaurus,(Schleitheimia,(Isanosaurus,((Tazoudasaurus,Vulcanodon),((Shunosaurus,(Spinophorosaurus,(Mamenchisaurus,(Omeisaurus,(Cetiosaurus,Neosauropoda))))),(Patagosaurus,Barapasaurus))))))))))))),(Irisosaurus,(Mussaurus,Leonerasaurus))))),(Xingxiulong,(Jingshanosaurus,Chuxiongosaurus)))))),(Eucnemesaurus_fortis,(Riojasaurus,Eucnemesaurus_entaxonis)))),(Plateosaurus_gracilis,(Plateosaurus_ingens,Plateosaurus_engelhardti))))))))))))),(Buriolestes,Pampadromaeus))),(Staurikosaurus,Herrerasaurus))))))));

(Euparkeria,(Crurotarsi,(Marasuchus,(Silesaurus,(Agnosphitys,(Ornithischia,(((Guaibasaurus,(Neotheropoda,Chindesaurus)),((Panphagia,(Eoraptor,((Saturnalia,Chromogisaurus),(Bagualosaurus,(Jaklapalisaurus,(Nambalia,((Thecodontosaurus,Pantydraco),(Efraasia,(Plateosauravus,(Ruehleia,(((Unaysaurus,(Pradhania,Macrocollum)),((Sarahsaurus,((Ngwevu,(Xixipiosaurus,((Coloradisaurus,(Glacialisaurus,Lufengosaurus)),(Massospondylus_carinatus,(Adeopapposaurus,Leyesaurus))))),(Yunnanosaurus_huangi,((Seitaad,(Anchisaurus,((Yizhousaurus,((Sefapanosaurus,Aardonyx),(NMQR1551,(NMRQ3314,(Blikanasaurus,((Camelotia,((Meroktenos,(Ledumahadi,Kholumolumo)),(Lessemsaurus,(Ingentia,Antetonitrus)))),(Pulanesaura,(Gongxianosaurus,(Schleitheimia,(Isanosaurus,((Tazoudasaurus,Vulcanodon),((Shunosaurus,(Spinophorosaurus,(Omeisaurus,(Mamenchisaurus,(Cetiosaurus,Neosauropoda))))),(Patagosaurus,Barapasaurus))))))))))))),(Irisosaurus,(Mussaurus,Leonerasaurus))))),(Xingxiulong,(Jingshanosaurus,Chuxiongosaurus)))))),(Eucnemesaurus_fortis,(Riojasaurus,Eucnemesaurus_entaxonis)))),(Plateosaurus_gracilis,(Plateosaurus_ingens,Plateosaurus_engelhardti))))))))))))),(Buriolestes,Pampadromaeus))),(Staurikosaurus,Herrerasaurus))))))));

(Euparkeria,(Crurotarsi,(Marasuchus,(Silesaurus,(Agnosphitys,(Ornithischia,(((Guaibasaurus,(Neotheropoda,Chindesaurus)),((Panphagia,(Eoraptor,((Saturnalia,Chromogisaurus),(Bagualosaurus,(Jaklapalisaurus,(Nambalia,((Thecodontosaurus,Pantydraco),(Efraasia,(Plateosauravus,(Ruehleia,(((Unaysaurus,(Pradhania,Macrocollum)),((Sarahsaurus,((Ngwevu,(Xixipiosaurus,((Coloradisaurus,(Glacialisaurus,Lufengosaurus)),(Massospondylus_carinatus,(Adeopapposaurus,Leyesaurus))))),(Yunnanosaurus_huangi,((Seitaad,(Anchisaurus,((Yizhousaurus,((Sefapanosaurus,Aardonyx),(NMQR1551,(NMRQ3314,(Blikanasaurus,((Camelotia,((Meroktenos,(Ledumahadi,Kholumolumo)),(Lessemsaurus,(Ingentia,Antetonitrus)))),(Pulanesaura,(Gongxianosaurus,(Schleitheimia,(Isanosaurus,((Tazoudasaurus,Vulcanodon),((Shunosaurus,((Spinophorosaurus,Omeisaurus),(Mamenchisaurus,(Cetiosaurus,Neosauropoda)))),(Patagosaurus,Barapasaurus))))))))))))),(Irisosaurus,(Mussaurus,Leonerasaurus))))),(Xingxiulong,(Jingshanosaurus,Chuxiongosaurus)))))),(Eucnemesaurus_fortis,(Riojasaurus,Eucnemesaurus_entaxonis)))),(Plateosaurus_gracilis,(Plateosaurus_ingens,Plateosaurus_engelhardti))))))))))))),(Buriolestes,Pampadromaeus))),(Staurikosaurus,Herrerasaurus))))))));

(Euparkeria,(Crurotarsi,(Marasuchus,(Silesaurus,(Agnosphitys,(Ornithischia,(((Guaibasaurus,(Neotheropoda,Chindesaurus)),(Buriolestes,(Pampadromaeus,(Panphagia,(Eoraptor,((Saturnalia,Chromogisaurus),(Bagualosaurus,(Jaklapalisaurus,(Nambalia,((Thecodontosaurus,Pantydraco),(Efraasia,(Plateosauravus,(Ruehleia,(((Unaysaurus,(Pradhania,Macrocollum)),((Sarahsaurus,((Ngwevu,((Coloradisaurus,(Glacialisaurus,Lufengosaurus)),(Xixipiosaurus,(Massospondylus_carinatus,(Adeopapposaurus,Leyesaurus))))),(Yunnanosaurus_huangi,((Seitaad,(Anchisaurus,((Yizhousaurus,((Sefapanosaurus,Aardonyx),(NMQR1551,(NMRQ3314,(Blikanasaurus,((Camelotia,((Meroktenos,(Ledumahadi,Kholumolumo)),(Lessemsaurus,(Ingentia,Antetonitrus)))),(Pulanesaura,(Gongxianosaurus,(Schleitheimia,(Isanosaurus,((Tazoudasaurus,Vulcanodon),((Shunosaurus,(Spinophorosaurus,(Omeisaurus,(Mamenchisaurus,(Cetiosaurus,Neosauropoda))))),(Patagosaurus,Barapasaurus))))))))))))),(Irisosaurus,(Mussaurus,Leonerasaurus))))),(Xingxiulong,(Jingshanosaurus,Chuxiongosaurus)))))),(Eucnemesaurus_fortis,(Riojasaurus,Eucnemesaurus_entaxonis)))),(Plateosaurus_gracilis,(Plateosaurus_ingens,Plateosaurus_engelhardti)))))))))))))))),(Staurikosaurus,Herrerasaurus))))))));

(Euparkeria,(Crurotarsi,(Marasuchus,(Silesaurus,(Agnosphitys,(Ornithischia,(((Guaibasaurus,(Neotheropoda,Chindesaurus)),(Buriolestes,(Pampadromaeus,(Panphagia,(Eoraptor,((Saturnalia,Chromogisaurus),(Bagualosaurus,(Jaklapalisaurus,(Nambalia,((Thecodontosaurus,Pantydraco),(Efraasia,(Plateosauravus,(Ruehleia,(((Unaysaurus,(Pradhania,Macrocollum)),((Sarahsaurus,((Ngwevu,((Coloradisaurus,(Glacialisaurus,Lufengosaurus)),(Xixipiosaurus,(Massospondylus_carinatus,(Adeopapposaurus,Leyesaurus))))),(Yunnanosaurus_huangi,((Seitaad,(Anchisaurus,((Yizhousaurus,((Sefapanosaurus,Aardonyx),(NMQR1551,(NMRQ3314,(Blikanasaurus,((Camelotia,((Meroktenos,(Ledumahadi,Kholumolumo)),(Lessemsaurus,(Ingentia,Antetonitrus)))),(Pulanesaura,(Gongxianosaurus,(Schleitheimia,(Isanosaurus,((Tazoudasaurus,Vulcanodon),((Shunosaurus,((Spinophorosaurus,Omeisaurus),(Mamenchisaurus,(Cetiosaurus,Neosauropoda)))),(Patagosaurus,Barapasaurus))))))))))))),(Irisosaurus,(Mussaurus,Leonerasaurus))))),(Xingxiulong,(Jingshanosaurus,Chuxiongosaurus)))))),(Eucnemesaurus_fortis,(Riojasaurus,Eucnemesaurus_entaxonis)))),(Plateosaurus_gracilis,(Plateosaurus_ingens,Plateosaurus_engelhardti)))))))))))))))),(Staurikosaurus,Herrerasaurus))))))));

(Euparkeria,(Crurotarsi,(Marasuchus,(Silesaurus,(Agnosphitys,(Ornithischia,(((Guaibasaurus,(Neotheropoda,Chindesaurus)),(Buriolestes,(Pampadromaeus,(Panphagia,(Eoraptor,((Saturnalia,Chromogisaurus),(Bagualosaurus,(Jaklapalisaurus,(Nambalia,((Thecodontosaurus,Pantydraco),(Efraasia,(Plateosauravus,(Ruehleia,(((Unaysaurus,(Pradhania,Macrocollum)),((Sarahsaurus,((Ngwevu,(Xixipiosaurus,((Coloradisaurus,(Glacialisaurus,Lufengosaurus)),(Massospondylus_carinatus,(Adeopapposaurus,Leyesaurus))))),(Yunnanosaurus_huangi,((Seitaad,(Anchisaurus,((Yizhousaurus,((Sefapanosaurus,Aardonyx),(NMQR1551,(NMRQ3314,((Camelotia,((Meroktenos,(Ledumahadi,Kholumolumo)),(Lessemsaurus,(Blikanasaurus,(Ingentia,Antetonitrus))))),(Pulanesaura,(Gongxianosaurus,(Schleitheimia,(Isanosaurus,((Tazoudasaurus,Vulcanodon),((Shunosaurus,(Spinophorosaurus,(Omeisaurus,(Mamenchisaurus,(Cetiosaurus,Neosauropoda))))),(Patagosaurus,Barapasaurus)))))))))))),(Irisosaurus,(Mussaurus,Leonerasaurus))))),(Xingxiulong,(Jingshanosaurus,Chuxiongosaurus)))))),(Eucnemesaurus_fortis,(Riojasaurus,Eucnemesaurus_entaxonis)))),(Plateosaurus_gracilis,(Plateosaurus_ingens,Plateosaurus_engelhardti)))))))))))))))),(Staurikosaurus,Herrerasaurus))))))));

(Euparkeria,(Crurotarsi,(Marasuchus,(Silesaurus,(Ornithischia,(Agnosphitys,(((Guaibasaurus,(Neotheropoda,Chindesaurus)),((Panphagia,(Eoraptor,((Saturnalia,Chromogisaurus),(Bagualosaurus,(Jaklapalisaurus,(Nambalia,((Thecodontosaurus,Pantydraco),(Efraasia,(Plateosauravus,(Ruehleia,(((Unaysaurus,(Pradhania,Macrocollum)),((Sarahsaurus,((Ngwevu,(Xixipiosaurus,((Coloradisaurus,(Glacialisaurus,Lufengosaurus)),(Massospondylus_carinatus,(Adeopapposaurus,Leyesaurus))))),(Yunnanosaurus_huangi,((Seitaad,(Anchisaurus,((Irisosaurus,(Yizhousaurus,((Sefapanosaurus,Aardonyx),(NMQR1551,(NMRQ3314,(Blikanasaurus,((Camelotia,((Meroktenos,(Ledumahadi,Kholumolumo)),(Lessemsaurus,(Ingentia,Antetonitrus)))),(Pulanesaura,(Gongxianosaurus,(Schleitheimia,(Isanosaurus,((Tazoudasaurus,Vulcanodon),((Shunosaurus,((Spinophorosaurus,(Omeisaurus,Mamenchisaurus)),(Cetiosaurus,Neosauropoda))),(Patagosaurus,Barapasaurus)))))))))))))),(Mussaurus,Leonerasaurus)))),(Xingxiulong,(Jingshanosaurus,Chuxiongosaurus)))))),(Eucnemesaurus_entaxonis,(Riojasaurus,Eucnemesaurus_fortis)))),(Plateosaurus_gracilis,(Plateosaurus_ingens,Plateosaurus_engelhardti))))))))))))),(Buriolestes,Pampadromaeus))),(Staurikosaurus,Herrerasaurus))))))));

(Euparkeria,(Crurotarsi,(Marasuchus,(Silesaurus,(Ornithischia,((Agnosphitys,((Guaibasaurus,(Neotheropoda,Chindesaurus)),((Panphagia,(Eoraptor,((Saturnalia,Chromogisaurus),(Bagualosaurus,(Jaklapalisaurus,(Nambalia,((Thecodontosaurus,Pantydraco),(Efraasia,(Plateosauravus,(Ruehleia,(((Unaysaurus,(Pradhania,Macrocollum)),((Sarahsaurus,((Ngwevu,(Xixipiosaurus,((Coloradisaurus,(Glacialisaurus,Lufengosaurus)),(Massospondylus_carinatus,(Adeopapposaurus,Leyesaurus))))),(Yunnanosaurus_huangi,((Seitaad,(Anchisaurus,((Irisosaurus,(Yizhousaurus,((Sefapanosaurus,Aardonyx),(NMQR1551,(NMRQ3314,(Blikanasaurus,((Camelotia,((Meroktenos,(Ledumahadi,Kholumolumo)),(Lessemsaurus,(Ingentia,Antetonitrus)))),(Pulanesaura,(Gongxianosaurus,(Schleitheimia,(Isanosaurus,((Tazoudasaurus,Vulcanodon),((Shunosaurus,((Spinophorosaurus,(Omeisaurus,Mamenchisaurus)),(Cetiosaurus,Neosauropoda))),(Patagosaurus,Barapasaurus)))))))))))))),(Mussaurus,Leonerasaurus)))),(Xingxiulong,(Jingshanosaurus,Chuxiongosaurus)))))),(Eucnemesaurus_entaxonis,(Riojasaurus,Eucnemesaurus_fortis)))),(Plateosaurus_gracilis,(Plateosaurus_ingens,Plateosaurus_engelhardti))))))))))))),(Buriolestes,Pampadromaeus)))),(Staurikosaurus,Herrerasaurus)))))));

(Euparkeria,(Crurotarsi,(Marasuchus,(Silesaurus,(Agnosphitys,(Ornithischia,(((Guaibasaurus,(Neotheropoda,Chindesaurus)),((Panphagia,(Eoraptor,((Saturnalia,Chromogisaurus),(Bagualosaurus,(Jaklapalisaurus,(Nambalia,((Thecodontosaurus,Pantydraco),(Efraasia,(Plateosauravus,(Ruehleia,(((Unaysaurus,(Pradhania,Macrocollum)),((Sarahsaurus,((Ngwevu,((Coloradisaurus,(Glacialisaurus,Lufengosaurus)),(Xixipiosaurus,(Massospondylus_carinatus,(Adeopapposaurus,Leyesaurus))))),(Yunnanosaurus_huangi,((Seitaad,(Anchisaurus,((Irisosaurus,(Yizhousaurus,((Sefapanosaurus,Aardonyx),(NMQR1551,(NMRQ3314,(Blikanasaurus,((Camelotia,((Meroktenos,(Ledumahadi,Kholumolumo)),(Lessemsaurus,(Ingentia,Antetonitrus)))),(Pulanesaura,(Gongxianosaurus,(Schleitheimia,(Isanosaurus,((Tazoudasaurus,Vulcanodon),((Shunosaurus,((Spinophorosaurus,(Omeisaurus,Mamenchisaurus)),(Cetiosaurus,Neosauropoda))),(Patagosaurus,Barapasaurus)))))))))))))),(Mussaurus,Leonerasaurus)))),(Xingxiulong,(Jingshanosaurus,Chuxiongosaurus)))))),(Eucnemesaurus_entaxonis,(Riojasaurus,Eucnemesaurus_fortis)))),(Plateosaurus_gracilis,(Plateosaurus_ingens,Plateosaurus_engelhardti))))))))))))),(Buriolestes,Pampadromaeus))),(Staurikosaurus,Herrerasaurus))))))));

(Euparkeria,(Crurotarsi,(Marasuchus,(Silesaurus,(Agnosphitys,(Ornithischia,(((Guaibasaurus,(Neotheropoda,Chindesaurus)),((Panphagia,(Eoraptor,((Saturnalia,Chromogisaurus),(Bagualosaurus,(Jaklapalisaurus,(Nambalia,((Thecodontosaurus,Pantydraco),(Efraasia,(Plateosauravus,(Ruehleia,(((Unaysaurus,(Pradhania,Macrocollum)),((Sarahsaurus,((Ngwevu,(Xixipiosaurus,((Coloradisaurus,(Glacialisaurus,Lufengosaurus)),(Massospondylus_carinatus,(Adeopapposaurus,Leyesaurus))))),(Yunnanosaurus_huangi,((Seitaad,(Anchisaurus,((Irisosaurus,(Yizhousaurus,((Sefapanosaurus,Aardonyx),(NMQR1551,(NMRQ3314,(Blikanasaurus,((Camelotia,((Meroktenos,(Ledumahadi,Kholumolumo)),(Lessemsaurus,(Ingentia,Antetonitrus)))),((Schleitheimia,(Isanosaurus,((Tazoudasaurus,Vulcanodon),((Shunosaurus,((Spinophorosaurus,(Omeisaurus,Mamenchisaurus)),(Cetiosaurus,Neosauropoda))),(Patagosaurus,Barapasaurus))))),(Pulanesaura,Gongxianosaurus))))))))),(Mussaurus,Leonerasaurus)))),(Xingxiulong,(Jingshanosaurus,Chuxiongosaurus)))))),(Eucnemesaurus_entaxonis,(Riojasaurus,Eucnemesaurus_fortis)))),(Plateosaurus_gracilis,(Plateosaurus_ingens,Plateosaurus_engelhardti))))))))))))),(Buriolestes,Pampadromaeus))),(Staurikosaurus,Herrerasaurus))))))));

(Euparkeria,(Crurotarsi,(Marasuchus,(Silesaurus,(Agnosphitys,(Ornithischia,(((Guaibasaurus,(Neotheropoda,Chindesaurus)),((Panphagia,(Eoraptor,((Saturnalia,Chromogisaurus),(Bagualosaurus,(Jaklapalisaurus,(Nambalia,((Thecodontosaurus,Pantydraco),(Efraasia,(Plateosauravus,(Ruehleia,(((Unaysaurus,(Pradhania,Macrocollum)),((Sarahsaurus,((Ngwevu,(Xixipiosaurus,((Coloradisaurus,(Glacialisaurus,Lufengosaurus)),(Massospondylus_carinatus,(Adeopapposaurus,Leyesaurus))))),(Yunnanosaurus_huangi,((Seitaad,(Anchisaurus,((Irisosaurus,(Yizhousaurus,((Sefapanosaurus,Aardonyx),(NMQR1551,(NMRQ3314,(Blikanasaurus,((Camelotia,((Meroktenos,(Ledumahadi,Kholumolumo)),(Lessemsaurus,(Ingentia,Antetonitrus)))),(Pulanesaura,(Gongxianosaurus,(Schleitheimia,(Isanosaurus,((Tazoudasaurus,Vulcanodon),((Shunosaurus,((Spinophorosaurus,Omeisaurus),(Mamenchisaurus,(Cetiosaurus,Neosauropoda)))),(Patagosaurus,Barapasaurus)))))))))))))),(Mussaurus,Leonerasaurus)))),(Xingxiulong,(Jingshanosaurus,Chuxiongosaurus)))))),(Eucnemesaurus_entaxonis,(Riojasaurus,Eucnemesaurus_fortis)))),(Plateosaurus_gracilis,(Plateosaurus_ingens,Plateosaurus_engelhardti))))))))))))),(Buriolestes,Pampadromaeus))),(Staurikosaurus,Herrerasaurus))))))));

(Euparkeria,(Crurotarsi,(Marasuchus,(Silesaurus,(Agnosphitys,(Ornithischia,(((Guaibasaurus,(Neotheropoda,Chindesaurus)),((Panphagia,(Eoraptor,((Saturnalia,Chromogisaurus),(Bagualosaurus,(Jaklapalisaurus,(Nambalia,((Thecodontosaurus,Pantydraco),(Efraasia,(Plateosauravus,(Ruehleia,(((Unaysaurus,(Pradhania,Macrocollum)),((Sarahsaurus,((Ngwevu,(Xixipiosaurus,((Coloradisaurus,(Glacialisaurus,Lufengosaurus)),(Massospondylus_carinatus,(Adeopapposaurus,Leyesaurus))))),(Yunnanosaurus_huangi,((Seitaad,(Anchisaurus,((Irisosaurus,(Yizhousaurus,((Sefapanosaurus,Aardonyx),(NMQR1551,(NMRQ3314,(Blikanasaurus,((Camelotia,((Meroktenos,(Ledumahadi,Kholumolumo)),(Lessemsaurus,(Ingentia,Antetonitrus)))),(Pulanesaura,(Gongxianosaurus,(Schleitheimia,(Isanosaurus,((Tazoudasaurus,Vulcanodon),((Shunosaurus,(Spinophorosaurus,(Omeisaurus,(Mamenchisaurus,(Cetiosaurus,Neosauropoda))))),(Patagosaurus,Barapasaurus)))))))))))))),(Mussaurus,Leonerasaurus)))),(Xingxiulong,(Jingshanosaurus,Chuxiongosaurus)))))),(Eucnemesaurus_entaxonis,(Riojasaurus,Eucnemesaurus_fortis)))),(Plateosaurus_gracilis,(Plateosaurus_ingens,Plateosaurus_engelhardti))))))))))))),(Buriolestes,Pampadromaeus))),(Staurikosaurus,Herrerasaurus))))))));

(Euparkeria,(Crurotarsi,(Marasuchus,(Silesaurus,(Agnosphitys,(Ornithischia,(((Guaibasaurus,(Neotheropoda,Chindesaurus)),((Panphagia,(Eoraptor,((Saturnalia,Chromogisaurus),(Bagualosaurus,(Jaklapalisaurus,(Nambalia,((Thecodontosaurus,Pantydraco),(Efraasia,(Plateosauravus,(Ruehleia,(((Unaysaurus,(Pradhania,Macrocollum)),((Sarahsaurus,((Ngwevu,((Coloradisaurus,(Glacialisaurus,Lufengosaurus)),(Xixipiosaurus,(Massospondylus_carinatus,(Adeopapposaurus,Leyesaurus))))),(Yunnanosaurus_huangi,((Seitaad,(Anchisaurus,((Irisosaurus,(Sefapanosaurus,(Yizhousaurus,(Aardonyx,(NMQR1551,(NMRQ3314,(Blikanasaurus,((Camelotia,((Meroktenos,(Ledumahadi,Kholumolumo)),(Lessemsaurus,(Ingentia,Antetonitrus)))),((Schleitheimia,(Isanosaurus,((Tazoudasaurus,Vulcanodon),((Shunosaurus,((Spinophorosaurus,(Omeisaurus,Mamenchisaurus)),(Cetiosaurus,Neosauropoda))),(Patagosaurus,Barapasaurus))))),(Pulanesaura,Gongxianosaurus)))))))))),(Mussaurus,Leonerasaurus)))),(Xingxiulong,(Jingshanosaurus,Chuxiongosaurus)))))),(Eucnemesaurus_entaxonis,(Riojasaurus,Eucnemesaurus_fortis)))),(Plateosaurus_gracilis,(Plateosaurus_ingens,Plateosaurus_engelhardti))))))))))))),(Buriolestes,Pampadromaeus))),(Staurikosaurus,Herrerasaurus))))))));

(Euparkeria,(Crurotarsi,(Marasuchus,(Silesaurus,(Agnosphitys,(Ornithischia,(((Guaibasaurus,(Neotheropoda,Chindesaurus)),((Panphagia,(Eoraptor,((Saturnalia,Chromogisaurus),(Bagualosaurus,(Jaklapalisaurus,(Nambalia,((Thecodontosaurus,Pantydraco),(Efraasia,(Plateosauravus,(Ruehleia,(((Unaysaurus,(Pradhania,Macrocollum)),((Sarahsaurus,((Ngwevu,(Xixipiosaurus,((Coloradisaurus,(Glacialisaurus,Lufengosaurus)),(Massospondylus_carinatus,(Adeopapposaurus,Leyesaurus))))),(Yunnanosaurus_huangi,((Seitaad,(Anchisaurus,((Irisosaurus,(Sefapanosaurus,(Yizhousaurus,(Aardonyx,(NMQR1551,(NMRQ3314,(Blikanasaurus,((Camelotia,((Meroktenos,(Ledumahadi,Kholumolumo)),(Lessemsaurus,(Ingentia,Antetonitrus)))),((Schleitheimia,(Isanosaurus,((Tazoudasaurus,Vulcanodon),((Shunosaurus,((Spinophorosaurus,Omeisaurus),(Mamenchisaurus,(Cetiosaurus,Neosauropoda)))),(Patagosaurus,Barapasaurus))))),(Pulanesaura,Gongxianosaurus)))))))))),(Mussaurus,Leonerasaurus)))),(Xingxiulong,(Jingshanosaurus,Chuxiongosaurus)))))),(Eucnemesaurus_entaxonis,(Riojasaurus,Eucnemesaurus_fortis)))),(Plateosaurus_gracilis,(Plateosaurus_ingens,Plateosaurus_engelhardti))))))))))))),(Buriolestes,Pampadromaeus))),(Staurikosaurus,Herrerasaurus))))))));

(Euparkeria,(Crurotarsi,(Marasuchus,(Silesaurus,(Agnosphitys,(Ornithischia,(((Guaibasaurus,(Neotheropoda,Chindesaurus)),((Panphagia,(Eoraptor,((Saturnalia,Chromogisaurus),(Bagualosaurus,(Jaklapalisaurus,(Nambalia,((Thecodontosaurus,Pantydraco),(Efraasia,(Plateosauravus,(Ruehleia,(((Unaysaurus,(Pradhania,Macrocollum)),((Sarahsaurus,((Ngwevu,(Xixipiosaurus,((Coloradisaurus,(Glacialisaurus,Lufengosaurus)),(Massospondylus_carinatus,(Adeopapposaurus,Leyesaurus))))),(Yunnanosaurus_huangi,((Seitaad,(Anchisaurus,((Irisosaurus,(Sefapanosaurus,(Yizhousaurus,(Aardonyx,(NMQR1551,(NMRQ3314,(Blikanasaurus,((Camelotia,((Meroktenos,(Ledumahadi,Kholumolumo)),(Lessemsaurus,(Ingentia,Antetonitrus)))),((Schleitheimia,(Isanosaurus,((Tazoudasaurus,Vulcanodon),((Shunosaurus,(Spinophorosaurus,(Mamenchisaurus,(Omeisaurus,(Cetiosaurus,Neosauropoda))))),(Patagosaurus,Barapasaurus))))),(Pulanesaura,Gongxianosaurus)))))))))),(Mussaurus,Leonerasaurus)))),(Xingxiulong,(Jingshanosaurus,Chuxiongosaurus)))))),(Eucnemesaurus_entaxonis,(Riojasaurus,Eucnemesaurus_fortis)))),(Plateosaurus_gracilis,(Plateosaurus_ingens,Plateosaurus_engelhardti))))))))))))),(Buriolestes,Pampadromaeus))),(Staurikosaurus,Herrerasaurus))))))));

(Euparkeria,(Crurotarsi,(Marasuchus,(Silesaurus,(Agnosphitys,(Ornithischia,(((Guaibasaurus,(Neotheropoda,Chindesaurus)),((Panphagia,(Eoraptor,((Saturnalia,Chromogisaurus),(Bagualosaurus,(Jaklapalisaurus,(Nambalia,((Thecodontosaurus,Pantydraco),(Efraasia,(Plateosauravus,(Ruehleia,(((Unaysaurus,(Pradhania,Macrocollum)),((Sarahsaurus,((Ngwevu,(Xixipiosaurus,((Coloradisaurus,(Glacialisaurus,Lufengosaurus)),(Massospondylus_carinatus,(Adeopapposaurus,Leyesaurus))))),(Yunnanosaurus_huangi,((Seitaad,(Anchisaurus,((Irisosaurus,(Sefapanosaurus,(Yizhousaurus,(Aardonyx,(NMQR1551,(NMRQ3314,(Blikanasaurus,((Camelotia,((Meroktenos,(Ledumahadi,Kholumolumo)),(Lessemsaurus,(Ingentia,Antetonitrus)))),((Schleitheimia,(Isanosaurus,((Tazoudasaurus,Vulcanodon),((Shunosaurus,(Spinophorosaurus,(Omeisaurus,(Mamenchisaurus,(Cetiosaurus,Neosauropoda))))),(Patagosaurus,Barapasaurus))))),(Pulanesaura,Gongxianosaurus)))))))))),(Mussaurus,Leonerasaurus)))),(Xingxiulong,(Jingshanosaurus,Chuxiongosaurus)))))),(Eucnemesaurus_entaxonis,(Riojasaurus,Eucnemesaurus_fortis)))),(Plateosaurus_gracilis,(Plateosaurus_ingens,Plateosaurus_engelhardti))))))))))))),(Buriolestes,Pampadromaeus))),(Staurikosaurus,Herrerasaurus))))))));

(Euparkeria,(Crurotarsi,(Marasuchus,(Silesaurus,(Ornithischia,(Agnosphitys,(((Guaibasaurus,(Neotheropoda,Chindesaurus)),((Panphagia,(Eoraptor,((Saturnalia,Chromogisaurus),(Bagualosaurus,(Jaklapalisaurus,(Nambalia,((Thecodontosaurus,Pantydraco),(Efraasia,(Plateosauravus,(Ruehleia,(((Unaysaurus,(Pradhania,Macrocollum)),((Sarahsaurus,((Ngwevu,(Xixipiosaurus,((Coloradisaurus,(Glacialisaurus,Lufengosaurus)),(Massospondylus_carinatus,(Adeopapposaurus,Leyesaurus))))),(Yunnanosaurus_huangi,((Seitaad,(Anchisaurus,((Irisosaurus,(Sefapanosaurus,(Yizhousaurus,(Aardonyx,(NMQR1551,(NMRQ3314,(Blikanasaurus,((Camelotia,((Meroktenos,(Ledumahadi,Kholumolumo)),(Lessemsaurus,(Ingentia,Antetonitrus)))),(Pulanesaura,(Gongxianosaurus,(Schleitheimia,(Isanosaurus,((Tazoudasaurus,Vulcanodon),((Shunosaurus,((Spinophorosaurus,Omeisaurus),(Mamenchisaurus,(Cetiosaurus,Neosauropoda)))),(Patagosaurus,Barapasaurus))))))))))))))),(Mussaurus,Leonerasaurus)))),(Xingxiulong,(Jingshanosaurus,Chuxiongosaurus)))))),(Eucnemesaurus_entaxonis,(Riojasaurus,Eucnemesaurus_fortis)))),(Plateosaurus_gracilis,(Plateosaurus_ingens,Plateosaurus_engelhardti))))))))))))),(Buriolestes,Pampadromaeus))),(Staurikosaurus,Herrerasaurus))))))));

(Euparkeria,(Crurotarsi,(Marasuchus,(Silesaurus,(Ornithischia,((Agnosphitys,((Guaibasaurus,(Neotheropoda,Chindesaurus)),((Panphagia,(Eoraptor,((Saturnalia,Chromogisaurus),(Bagualosaurus,(Jaklapalisaurus,(Nambalia,((Thecodontosaurus,Pantydraco),(Efraasia,(Plateosauravus,(Ruehleia,(((Unaysaurus,(Pradhania,Macrocollum)),((Sarahsaurus,((Ngwevu,(Xixipiosaurus,((Coloradisaurus,(Glacialisaurus,Lufengosaurus)),(Massospondylus_carinatus,(Adeopapposaurus,Leyesaurus))))),(Yunnanosaurus_huangi,((Seitaad,(Anchisaurus,((Irisosaurus,(Sefapanosaurus,(Yizhousaurus,(Aardonyx,(NMQR1551,(NMRQ3314,(Blikanasaurus,((Camelotia,((Meroktenos,(Ledumahadi,Kholumolumo)),(Lessemsaurus,(Ingentia,Antetonitrus)))),(Pulanesaura,(Gongxianosaurus,(Schleitheimia,(Isanosaurus,((Tazoudasaurus,Vulcanodon),((Shunosaurus,((Spinophorosaurus,Omeisaurus),(Mamenchisaurus,(Cetiosaurus,Neosauropoda)))),(Patagosaurus,Barapasaurus))))))))))))))),(Mussaurus,Leonerasaurus)))),(Xingxiulong,(Jingshanosaurus,Chuxiongosaurus)))))),(Eucnemesaurus_entaxonis,(Riojasaurus,Eucnemesaurus_fortis)))),(Plateosaurus_gracilis,(Plateosaurus_ingens,Plateosaurus_engelhardti))))))))))))),(Buriolestes,Pampadromaeus)))),(Staurikosaurus,Herrerasaurus)))))));

(Euparkeria,(Crurotarsi,(Marasuchus,(Silesaurus,(Agnosphitys,(Ornithischia,(((Guaibasaurus,(Neotheropoda,Chindesaurus)),(Buriolestes,(Pampadromaeus,(Panphagia,(Eoraptor,((Saturnalia,Chromogisaurus),(Bagualosaurus,(Jaklapalisaurus,(Nambalia,((Thecodontosaurus,Pantydraco),(Efraasia,(Plateosauravus,(Ruehleia,(((Unaysaurus,(Pradhania,Macrocollum)),((Sarahsaurus,((Ngwevu,(Xixipiosaurus,((Coloradisaurus,(Glacialisaurus,Lufengosaurus)),(Massospondylus_carinatus,(Adeopapposaurus,Leyesaurus))))),(Yunnanosaurus_huangi,((Seitaad,(Anchisaurus,((Irisosaurus,(Sefapanosaurus,(Yizhousaurus,(Aardonyx,(NMQR1551,(NMRQ3314,(Blikanasaurus,((Camelotia,((Meroktenos,(Ledumahadi,Kholumolumo)),(Lessemsaurus,(Ingentia,Antetonitrus)))),(Pulanesaura,(Gongxianosaurus,(Schleitheimia,(Isanosaurus,((Tazoudasaurus,Vulcanodon),((Shunosaurus,((Spinophorosaurus,Omeisaurus),(Mamenchisaurus,(Cetiosaurus,Neosauropoda)))),(Patagosaurus,Barapasaurus))))))))))))))),(Mussaurus,Leonerasaurus)))),(Xingxiulong,(Jingshanosaurus,Chuxiongosaurus)))))),(Eucnemesaurus_entaxonis,(Riojasaurus,Eucnemesaurus_fortis)))),(Plateosaurus_gracilis,(Plateosaurus_ingens,Plateosaurus_engelhardti)))))))))))))))),(Staurikosaurus,Herrerasaurus))))))));

(Euparkeria,(Crurotarsi,(Marasuchus,(Silesaurus,(Agnosphitys,(Ornithischia,(((Guaibasaurus,(Neotheropoda,Chindesaurus)),((Panphagia,(Eoraptor,((Saturnalia,Chromogisaurus),(Bagualosaurus,(Jaklapalisaurus,(Nambalia,((Thecodontosaurus,Pantydraco),(Efraasia,(Plateosauravus,(Ruehleia,(((Unaysaurus,(Pradhania,Macrocollum)),((Sarahsaurus,((Ngwevu,((Coloradisaurus,(Glacialisaurus,Lufengosaurus)),(Xixipiosaurus,(Massospondylus_carinatus,(Adeopapposaurus,Leyesaurus))))),(Yunnanosaurus_huangi,((Seitaad,(Anchisaurus,((Irisosaurus,(Sefapanosaurus,(Yizhousaurus,(Aardonyx,(NMQR1551,(NMRQ3314,(Blikanasaurus,((Camelotia,((Meroktenos,(Ledumahadi,Kholumolumo)),(Lessemsaurus,(Ingentia,Antetonitrus)))),(Pulanesaura,(Gongxianosaurus,(Schleitheimia,(Isanosaurus,((Tazoudasaurus,Vulcanodon),((Shunosaurus,((Spinophorosaurus,Omeisaurus),(Mamenchisaurus,(Cetiosaurus,Neosauropoda)))),(Patagosaurus,Barapasaurus))))))))))))))),(Mussaurus,Leonerasaurus)))),(Xingxiulong,(Jingshanosaurus,Chuxiongosaurus)))))),(Eucnemesaurus_entaxonis,(Riojasaurus,Eucnemesaurus_fortis)))),(Plateosaurus_gracilis,(Plateosaurus_ingens,Plateosaurus_engelhardti))))))))))))),(Buriolestes,Pampadromaeus))),(Staurikosaurus,Herrerasaurus))))))));

(Euparkeria,(Crurotarsi,(Marasuchus,(Silesaurus,(Ornithischia,(Agnosphitys,(((Guaibasaurus,(Neotheropoda,Chindesaurus)),(Buriolestes,(Pampadromaeus,(Panphagia,(Eoraptor,((Saturnalia,Chromogisaurus),(Bagualosaurus,(Jaklapalisaurus,(Nambalia,((Thecodontosaurus,Pantydraco),(Efraasia,(Plateosauravus,(Ruehleia,(((Unaysaurus,(Pradhania,Macrocollum)),((Sarahsaurus,((Ngwevu,(Xixipiosaurus,((Coloradisaurus,(Glacialisaurus,Lufengosaurus)),(Massospondylus_carinatus,(Adeopapposaurus,Leyesaurus))))),(Yunnanosaurus_huangi,((Seitaad,(Anchisaurus,((Irisosaurus,(Sefapanosaurus,(Yizhousaurus,(Aardonyx,(NMQR1551,(NMRQ3314,(Blikanasaurus,((Camelotia,((Meroktenos,(Ledumahadi,Kholumolumo)),(Lessemsaurus,(Ingentia,Antetonitrus)))),(Pulanesaura,(Gongxianosaurus,(Schleitheimia,(Isanosaurus,((Tazoudasaurus,Vulcanodon),((Shunosaurus,((Spinophorosaurus,(Omeisaurus,Mamenchisaurus)),(Cetiosaurus,Neosauropoda))),(Patagosaurus,Barapasaurus))))))))))))))),(Mussaurus,Leonerasaurus)))),(Xingxiulong,(Jingshanosaurus,Chuxiongosaurus)))))),(Eucnemesaurus_entaxonis,(Riojasaurus,Eucnemesaurus_fortis)))),(Plateosaurus_gracilis,(Plateosaurus_ingens,Plateosaurus_engelhardti)))))))))))))))),(Staurikosaurus,Herrerasaurus))))))));

(Euparkeria,(Crurotarsi,(Marasuchus,(Silesaurus,(Agnosphitys,(Ornithischia,(((Guaibasaurus,(Neotheropoda,Chindesaurus)),(Buriolestes,(Pampadromaeus,(Panphagia,(Eoraptor,((Saturnalia,Chromogisaurus),(Bagualosaurus,(Jaklapalisaurus,(Nambalia,((Thecodontosaurus,Pantydraco),(Efraasia,(Plateosauravus,(Ruehleia,(((Unaysaurus,(Pradhania,Macrocollum)),((Sarahsaurus,((Ngwevu,((Coloradisaurus,(Glacialisaurus,Lufengosaurus)),(Xixipiosaurus,(Massospondylus_carinatus,(Adeopapposaurus,Leyesaurus))))),(Yunnanosaurus_huangi,((Seitaad,(Anchisaurus,((Irisosaurus,(Sefapanosaurus,(Yizhousaurus,(Aardonyx,(NMQR1551,(NMRQ3314,(Blikanasaurus,((Camelotia,((Meroktenos,(Ledumahadi,Kholumolumo)),(Lessemsaurus,(Ingentia,Antetonitrus)))),(Pulanesaura,(Gongxianosaurus,(Schleitheimia,(Isanosaurus,((Tazoudasaurus,Vulcanodon),((Shunosaurus,((Spinophorosaurus,(Omeisaurus,Mamenchisaurus)),(Cetiosaurus,Neosauropoda))),(Patagosaurus,Barapasaurus))))))))))))))),(Mussaurus,Leonerasaurus)))),(Xingxiulong,(Jingshanosaurus,Chuxiongosaurus)))))),(Eucnemesaurus_entaxonis,(Riojasaurus,Eucnemesaurus_fortis)))),(Plateosaurus_gracilis,(Plateosaurus_ingens,Plateosaurus_engelhardti)))))))))))))))),(Staurikosaurus,Herrerasaurus))))))));

(Euparkeria,(Crurotarsi,(Marasuchus,(Silesaurus,(Agnosphitys,(Ornithischia,(((Guaibasaurus,(Neotheropoda,Chindesaurus)),(Buriolestes,(Pampadromaeus,(Panphagia,(Eoraptor,((Saturnalia,Chromogisaurus),(Bagualosaurus,(Jaklapalisaurus,(Nambalia,((Thecodontosaurus,Pantydraco),(Efraasia,(Plateosauravus,(Ruehleia,(((Unaysaurus,(Pradhania,Macrocollum)),((Sarahsaurus,((Ngwevu,(Xixipiosaurus,((Coloradisaurus,(Glacialisaurus,Lufengosaurus)),(Massospondylus_carinatus,(Adeopapposaurus,Leyesaurus))))),(Yunnanosaurus_huangi,((Seitaad,(Anchisaurus,((Irisosaurus,(Sefapanosaurus,(Yizhousaurus,(Aardonyx,(NMQR1551,(NMRQ3314,(Blikanasaurus,((Camelotia,((Meroktenos,(Ledumahadi,Kholumolumo)),(Lessemsaurus,(Ingentia,Antetonitrus)))),(Pulanesaura,(Gongxianosaurus,(Schleitheimia,(Isanosaurus,((Tazoudasaurus,Vulcanodon),((Shunosaurus,(Spinophorosaurus,(Omeisaurus,(Mamenchisaurus,(Cetiosaurus,Neosauropoda))))),(Patagosaurus,Barapasaurus))))))))))))))),(Mussaurus,Leonerasaurus)))),(Xingxiulong,(Jingshanosaurus,Chuxiongosaurus)))))),(Eucnemesaurus_entaxonis,(Riojasaurus,Eucnemesaurus_fortis)))),(Plateosaurus_gracilis,(Plateosaurus_ingens,Plateosaurus_engelhardti)))))))))))))))),(Staurikosaurus,Herrerasaurus))))))));

(Euparkeria,(Crurotarsi,(Marasuchus,(Silesaurus,(Ornithischia,(Agnosphitys,(((Guaibasaurus,(Neotheropoda,Chindesaurus)),((Panphagia,(Eoraptor,((Saturnalia,Chromogisaurus),(Bagualosaurus,(Jaklapalisaurus,(Nambalia,((Thecodontosaurus,Pantydraco),(Efraasia,(Plateosauravus,(Ruehleia,(((Unaysaurus,(Pradhania,Macrocollum)),((Sarahsaurus,((Ngwevu,(Xixipiosaurus,((Coloradisaurus,(Glacialisaurus,Lufengosaurus)),(Massospondylus_carinatus,(Adeopapposaurus,Leyesaurus))))),(Yunnanosaurus_huangi,((Seitaad,(Anchisaurus,((Irisosaurus,(Sefapanosaurus,(Yizhousaurus,(Aardonyx,(NMQR1551,(NMRQ3314,(Blikanasaurus,((Camelotia,((Meroktenos,(Ledumahadi,Kholumolumo)),(Lessemsaurus,(Ingentia,Antetonitrus)))),(Pulanesaura,(Gongxianosaurus,(Schleitheimia,(Isanosaurus,((Tazoudasaurus,Vulcanodon),((Shunosaurus,(Spinophorosaurus,(Mamenchisaurus,(Omeisaurus,(Cetiosaurus,Neosauropoda))))),(Patagosaurus,Barapasaurus))))))))))))))),(Mussaurus,Leonerasaurus)))),(Xingxiulong,(Jingshanosaurus,Chuxiongosaurus)))))),(Eucnemesaurus_entaxonis,(Riojasaurus,Eucnemesaurus_fortis)))),(Plateosaurus_gracilis,(Plateosaurus_ingens,Plateosaurus_engelhardti))))))))))))),(Buriolestes,Pampadromaeus))),(Staurikosaurus,Herrerasaurus))))))));

(Euparkeria,(Crurotarsi,(Marasuchus,(Silesaurus,(Ornithischia,((Agnosphitys,((Guaibasaurus,(Neotheropoda,Chindesaurus)),((Panphagia,(Eoraptor,((Saturnalia,Chromogisaurus),(Bagualosaurus,(Jaklapalisaurus,(Nambalia,((Thecodontosaurus,Pantydraco),(Efraasia,(Plateosauravus,(Ruehleia,(((Unaysaurus,(Pradhania,Macrocollum)),((Sarahsaurus,((Ngwevu,(Xixipiosaurus,((Coloradisaurus,(Glacialisaurus,Lufengosaurus)),(Massospondylus_carinatus,(Adeopapposaurus,Leyesaurus))))),(Yunnanosaurus_huangi,((Seitaad,(Anchisaurus,((Irisosaurus,(Sefapanosaurus,(Yizhousaurus,(Aardonyx,(NMQR1551,(NMRQ3314,(Blikanasaurus,((Camelotia,((Meroktenos,(Ledumahadi,Kholumolumo)),(Lessemsaurus,(Ingentia,Antetonitrus)))),(Pulanesaura,(Gongxianosaurus,(Schleitheimia,(Isanosaurus,((Tazoudasaurus,Vulcanodon),((Shunosaurus,(Spinophorosaurus,(Mamenchisaurus,(Omeisaurus,(Cetiosaurus,Neosauropoda))))),(Patagosaurus,Barapasaurus))))))))))))))),(Mussaurus,Leonerasaurus)))),(Xingxiulong,(Jingshanosaurus,Chuxiongosaurus)))))),(Eucnemesaurus_entaxonis,(Riojasaurus,Eucnemesaurus_fortis)))),(Plateosaurus_gracilis,(Plateosaurus_ingens,Plateosaurus_engelhardti))))))))))))),(Buriolestes,Pampadromaeus)))),(Staurikosaurus,Herrerasaurus)))))));

(Euparkeria,(Crurotarsi,(Marasuchus,(Silesaurus,(Agnosphitys,(Ornithischia,(((Guaibasaurus,(Neotheropoda,Chindesaurus)),((Panphagia,(Eoraptor,((Saturnalia,Chromogisaurus),(Bagualosaurus,(Jaklapalisaurus,(Nambalia,((Thecodontosaurus,Pantydraco),(Efraasia,(Plateosauravus,(Ruehleia,(((Unaysaurus,(Pradhania,Macrocollum)),((Sarahsaurus,((Ngwevu,((Coloradisaurus,(Glacialisaurus,Lufengosaurus)),(Xixipiosaurus,(Massospondylus_carinatus,(Adeopapposaurus,Leyesaurus))))),(Yunnanosaurus_huangi,((Seitaad,(Anchisaurus,((Irisosaurus,(Sefapanosaurus,(Yizhousaurus,(Aardonyx,(NMQR1551,(NMRQ3314,(Blikanasaurus,((Camelotia,((Meroktenos,(Ledumahadi,Kholumolumo)),(Lessemsaurus,(Ingentia,Antetonitrus)))),(Pulanesaura,(Gongxianosaurus,(Schleitheimia,(Isanosaurus,((Tazoudasaurus,Vulcanodon),((Shunosaurus,(Spinophorosaurus,(Mamenchisaurus,(Omeisaurus,(Cetiosaurus,Neosauropoda))))),(Patagosaurus,Barapasaurus))))))))))))))),(Mussaurus,Leonerasaurus)))),(Xingxiulong,(Jingshanosaurus,Chuxiongosaurus)))))),(Eucnemesaurus_entaxonis,(Riojasaurus,Eucnemesaurus_fortis)))),(Plateosaurus_gracilis,(Plateosaurus_ingens,Plateosaurus_engelhardti))))))))))))),(Buriolestes,Pampadromaeus))),(Staurikosaurus,Herrerasaurus))))))));

(Euparkeria,(Crurotarsi,(Marasuchus,(Silesaurus,(Ornithischia,(Agnosphitys,(((Guaibasaurus,(Neotheropoda,Chindesaurus)),((Panphagia,(Eoraptor,((Saturnalia,Chromogisaurus),(Bagualosaurus,(Jaklapalisaurus,(Nambalia,((Thecodontosaurus,Pantydraco),(Efraasia,(Plateosauravus,(Ruehleia,(((Unaysaurus,(Pradhania,Macrocollum)),((Sarahsaurus,((Ngwevu,(Xixipiosaurus,((Coloradisaurus,(Glacialisaurus,Lufengosaurus)),(Massospondylus_carinatus,(Adeopapposaurus,Leyesaurus))))),(Yunnanosaurus_huangi,((Seitaad,(Anchisaurus,((Irisosaurus,(Sefapanosaurus,(Yizhousaurus,(Aardonyx,(NMQR1551,(NMRQ3314,(Blikanasaurus,((Camelotia,((Meroktenos,(Ledumahadi,Kholumolumo)),(Lessemsaurus,(Ingentia,Antetonitrus)))),(Pulanesaura,(Gongxianosaurus,(Schleitheimia,(Isanosaurus,((Tazoudasaurus,Vulcanodon),((Shunosaurus,(Spinophorosaurus,(Omeisaurus,(Mamenchisaurus,(Cetiosaurus,Neosauropoda))))),(Patagosaurus,Barapasaurus))))))))))))))),(Mussaurus,Leonerasaurus)))),(Xingxiulong,(Jingshanosaurus,Chuxiongosaurus)))))),(Eucnemesaurus_entaxonis,(Riojasaurus,Eucnemesaurus_fortis)))),(Plateosaurus_gracilis,(Plateosaurus_ingens,Plateosaurus_engelhardti))))))))))))),(Buriolestes,Pampadromaeus))),(Staurikosaurus,Herrerasaurus))))))));

(Euparkeria,(Crurotarsi,(Marasuchus,(Silesaurus,(Ornithischia,((Agnosphitys,((Guaibasaurus,(Neotheropoda,Chindesaurus)),((Panphagia,(Eoraptor,((Saturnalia,Chromogisaurus),(Bagualosaurus,(Jaklapalisaurus,(Nambalia,((Thecodontosaurus,Pantydraco),(Efraasia,(Plateosauravus,(Ruehleia,(((Unaysaurus,(Pradhania,Macrocollum)),((Sarahsaurus,((Ngwevu,(Xixipiosaurus,((Coloradisaurus,(Glacialisaurus,Lufengosaurus)),(Massospondylus_carinatus,(Adeopapposaurus,Leyesaurus))))),(Yunnanosaurus_huangi,((Seitaad,(Anchisaurus,((Irisosaurus,(Sefapanosaurus,(Yizhousaurus,(Aardonyx,(NMQR1551,(NMRQ3314,(Blikanasaurus,((Camelotia,((Meroktenos,(Ledumahadi,Kholumolumo)),(Lessemsaurus,(Ingentia,Antetonitrus)))),(Pulanesaura,(Gongxianosaurus,(Schleitheimia,(Isanosaurus,((Tazoudasaurus,Vulcanodon),((Shunosaurus,(Spinophorosaurus,(Omeisaurus,(Mamenchisaurus,(Cetiosaurus,Neosauropoda))))),(Patagosaurus,Barapasaurus))))))))))))))),(Mussaurus,Leonerasaurus)))),(Xingxiulong,(Jingshanosaurus,Chuxiongosaurus)))))),(Eucnemesaurus_entaxonis,(Riojasaurus,Eucnemesaurus_fortis)))),(Plateosaurus_gracilis,(Plateosaurus_ingens,Plateosaurus_engelhardti))))))))))))),(Buriolestes,Pampadromaeus)))),(Staurikosaurus,Herrerasaurus)))))));

(Euparkeria,(Crurotarsi,(Marasuchus,(Silesaurus,(Agnosphitys,(Ornithischia,(((Guaibasaurus,(Neotheropoda,Chindesaurus)),((Panphagia,(Eoraptor,((Saturnalia,Chromogisaurus),(Bagualosaurus,(Jaklapalisaurus,(Nambalia,((Thecodontosaurus,Pantydraco),(Efraasia,(Plateosauravus,(Ruehleia,(((Unaysaurus,(Pradhania,Macrocollum)),((Sarahsaurus,((Ngwevu,((Coloradisaurus,(Glacialisaurus,Lufengosaurus)),(Xixipiosaurus,(Massospondylus_carinatus,(Adeopapposaurus,Leyesaurus))))),(Yunnanosaurus_huangi,((Seitaad,(Anchisaurus,((Irisosaurus,(Sefapanosaurus,(Yizhousaurus,(Aardonyx,(NMQR1551,(NMRQ3314,(Blikanasaurus,((Camelotia,((Meroktenos,(Ledumahadi,Kholumolumo)),(Lessemsaurus,(Ingentia,Antetonitrus)))),(Pulanesaura,(Gongxianosaurus,(Schleitheimia,(Isanosaurus,((Tazoudasaurus,Vulcanodon),((Shunosaurus,(Spinophorosaurus,(Omeisaurus,(Mamenchisaurus,(Cetiosaurus,Neosauropoda))))),(Patagosaurus,Barapasaurus))))))))))))))),(Mussaurus,Leonerasaurus)))),(Xingxiulong,(Jingshanosaurus,Chuxiongosaurus)))))),(Eucnemesaurus_entaxonis,(Riojasaurus,Eucnemesaurus_fortis)))),(Plateosaurus_gracilis,(Plateosaurus_ingens,Plateosaurus_engelhardti))))))))))))),(Buriolestes,Pampadromaeus))),(Staurikosaurus,Herrerasaurus))))))));

(Euparkeria,(Crurotarsi,(Marasuchus,(Silesaurus,(Agnosphitys,(Ornithischia,(((Guaibasaurus,(Neotheropoda,Chindesaurus)),((Panphagia,(Eoraptor,((Saturnalia,Chromogisaurus),(Bagualosaurus,(Jaklapalisaurus,(Nambalia,((Thecodontosaurus,Pantydraco),(Efraasia,(Plateosauravus,(Ruehleia,(((Unaysaurus,(Pradhania,Macrocollum)),((Sarahsaurus,((Ngwevu,(Xixipiosaurus,((Coloradisaurus,(Glacialisaurus,Lufengosaurus)),(Massospondylus_carinatus,(Adeopapposaurus,Leyesaurus))))),(Yunnanosaurus_huangi,((Seitaad,(Anchisaurus,((Irisosaurus,(Sefapanosaurus,(Yizhousaurus,(Aardonyx,(NMQR1551,(NMRQ3314,((Camelotia,((Meroktenos,(Ledumahadi,Kholumolumo)),(Lessemsaurus,(Blikanasaurus,(Ingentia,Antetonitrus))))),(Pulanesaura,(Gongxianosaurus,(Schleitheimia,(Isanosaurus,((Tazoudasaurus,Vulcanodon),((Shunosaurus,(Spinophorosaurus,(Omeisaurus,(Mamenchisaurus,(Cetiosaurus,Neosauropoda))))),(Patagosaurus,Barapasaurus)))))))))))))),(Mussaurus,Leonerasaurus)))),(Xingxiulong,(Jingshanosaurus,Chuxiongosaurus)))))),(Eucnemesaurus_entaxonis,(Riojasaurus,Eucnemesaurus_fortis)))),(Plateosaurus_gracilis,(Plateosaurus_ingens,Plateosaurus_engelhardti))))))))))))),(Buriolestes,Pampadromaeus))),(Staurikosaurus,Herrerasaurus))))))));

(Euparkeria,(Crurotarsi,(Marasuchus,(Silesaurus,(Ornithischia,(Agnosphitys,(((Guaibasaurus,(Neotheropoda,Chindesaurus)),((Panphagia,(Eoraptor,((Saturnalia,Chromogisaurus),(Bagualosaurus,(Jaklapalisaurus,(Nambalia,((Thecodontosaurus,Pantydraco),(Efraasia,(Plateosauravus,(Ruehleia,(((Unaysaurus,(Pradhania,Macrocollum)),((Sarahsaurus,((Ngwevu,((Coloradisaurus,(Glacialisaurus,Lufengosaurus)),(Xixipiosaurus,(Massospondylus_carinatus,(Adeopapposaurus,Leyesaurus))))),(Yunnanosaurus_huangi,((Seitaad,(Anchisaurus,((Irisosaurus,(Sefapanosaurus,(Yizhousaurus,(Aardonyx,(NMQR1551,(NMRQ3314,(Blikanasaurus,((Camelotia,((Meroktenos,(Ledumahadi,Kholumolumo)),(Lessemsaurus,(Ingentia,Antetonitrus)))),(Pulanesaura,(Gongxianosaurus,(Schleitheimia,(Isanosaurus,((Tazoudasaurus,Vulcanodon),((Shunosaurus,((Spinophorosaurus,(Omeisaurus,Mamenchisaurus)),(Cetiosaurus,Neosauropoda))),(Patagosaurus,Barapasaurus))))))))))))))),(Mussaurus,Leonerasaurus)))),(Xingxiulong,(Jingshanosaurus,Chuxiongosaurus)))))),(Eucnemesaurus_entaxonis,(Riojasaurus,Eucnemesaurus_fortis)))),(Plateosaurus_gracilis,(Plateosaurus_ingens,Plateosaurus_engelhardti))))))))))))),(Buriolestes,Pampadromaeus))),(Staurikosaurus,Herrerasaurus))))))));

(Euparkeria,(Crurotarsi,(Marasuchus,(Silesaurus,(Ornithischia,((Agnosphitys,(Staurikosaurus,Herrerasaurus)),((Guaibasaurus,(Neotheropoda,Chindesaurus)),((Panphagia,(Eoraptor,((Saturnalia,Chromogisaurus),(Bagualosaurus,(Jaklapalisaurus,(Nambalia,((Thecodontosaurus,Pantydraco),(Efraasia,(Plateosauravus,(Ruehleia,(((Unaysaurus,(Pradhania,Macrocollum)),((Sarahsaurus,((Ngwevu,((Coloradisaurus,(Glacialisaurus,Lufengosaurus)),(Xixipiosaurus,(Massospondylus_carinatus,(Adeopapposaurus,Leyesaurus))))),(Yunnanosaurus_huangi,((Seitaad,(Anchisaurus,((Irisosaurus,(Sefapanosaurus,(Yizhousaurus,(Aardonyx,(NMQR1551,(NMRQ3314,(Blikanasaurus,((Camelotia,((Meroktenos,(Ledumahadi,Kholumolumo)),(Lessemsaurus,(Ingentia,Antetonitrus)))),(Pulanesaura,(Gongxianosaurus,(Schleitheimia,(Isanosaurus,((Tazoudasaurus,Vulcanodon),((Shunosaurus,((Spinophorosaurus,(Omeisaurus,Mamenchisaurus)),(Cetiosaurus,Neosauropoda))),(Patagosaurus,Barapasaurus))))))))))))))),(Mussaurus,Leonerasaurus)))),(Xingxiulong,(Jingshanosaurus,Chuxiongosaurus)))))),(Eucnemesaurus_entaxonis,(Riojasaurus,Eucnemesaurus_fortis)))),(Plateosaurus_gracilis,(Plateosaurus_ingens,Plateosaurus_engelhardti))))))))))))),(Buriolestes,Pampadromaeus)))))))));

(Euparkeria,(Crurotarsi,(Marasuchus,(Silesaurus,(Agnosphitys,(Ornithischia,(((Guaibasaurus,(Neotheropoda,Chindesaurus)),(Buriolestes,(Pampadromaeus,(Panphagia,(Eoraptor,((Saturnalia,Chromogisaurus),(Bagualosaurus,(Jaklapalisaurus,(Nambalia,((Thecodontosaurus,Pantydraco),(Efraasia,(Plateosauravus,(Ruehleia,(((Unaysaurus,(Pradhania,Macrocollum)),((Sarahsaurus,((Ngwevu,(Xixipiosaurus,((Coloradisaurus,(Glacialisaurus,Lufengosaurus)),(Massospondylus_carinatus,(Adeopapposaurus,Leyesaurus))))),(Yunnanosaurus_huangi,((Seitaad,(Anchisaurus,((Irisosaurus,(Yizhousaurus,((Sefapanosaurus,Aardonyx),(NMQR1551,(NMRQ3314,(Blikanasaurus,((Camelotia,((Meroktenos,(Ledumahadi,Kholumolumo)),(Lessemsaurus,(Ingentia,Antetonitrus)))),((Schleitheimia,(Isanosaurus,((Tazoudasaurus,Vulcanodon),((Shunosaurus,((Spinophorosaurus,(Omeisaurus,Mamenchisaurus)),(Cetiosaurus,Neosauropoda))),(Patagosaurus,Barapasaurus))))),(Pulanesaura,Gongxianosaurus))))))))),(Mussaurus,Leonerasaurus)))),(Xingxiulong,(Jingshanosaurus,Chuxiongosaurus)))))),(Eucnemesaurus_fortis,(Riojasaurus,Eucnemesaurus_entaxonis)))),(Plateosaurus_gracilis,(Plateosaurus_ingens,Plateosaurus_engelhardti)))))))))))))))),(Staurikosaurus,Herrerasaurus))))))));

(Euparkeria,(Crurotarsi,(Marasuchus,(Silesaurus,(Ornithischia,(Agnosphitys,(((Guaibasaurus,(Neotheropoda,Chindesaurus)),(Buriolestes,(Pampadromaeus,(Panphagia,(Eoraptor,((Saturnalia,Chromogisaurus),(Bagualosaurus,(Jaklapalisaurus,(Nambalia,((Thecodontosaurus,Pantydraco),(Efraasia,(Plateosauravus,(Ruehleia,(((Unaysaurus,(Pradhania,Macrocollum)),((Sarahsaurus,((Ngwevu,(Xixipiosaurus,((Coloradisaurus,(Glacialisaurus,Lufengosaurus)),(Massospondylus_carinatus,(Adeopapposaurus,Leyesaurus))))),(Yunnanosaurus_huangi,((Seitaad,(Anchisaurus,((Irisosaurus,(Yizhousaurus,((Sefapanosaurus,Aardonyx),(NMQR1551,(NMRQ3314,(Blikanasaurus,((Camelotia,((Meroktenos,(Ledumahadi,Kholumolumo)),(Lessemsaurus,(Ingentia,Antetonitrus)))),((Schleitheimia,(Isanosaurus,((Tazoudasaurus,Vulcanodon),((Shunosaurus,((Spinophorosaurus,(Omeisaurus,Mamenchisaurus)),(Cetiosaurus,Neosauropoda))),(Patagosaurus,Barapasaurus))))),(Pulanesaura,Gongxianosaurus))))))))),(Mussaurus,Leonerasaurus)))),(Xingxiulong,(Jingshanosaurus,Chuxiongosaurus)))))),(Eucnemesaurus_fortis,(Riojasaurus,Eucnemesaurus_entaxonis)))),(Plateosaurus_gracilis,(Plateosaurus_ingens,Plateosaurus_engelhardti)))))))))))))))),(Staurikosaurus,Herrerasaurus))))))));

(Euparkeria,(Crurotarsi,(Marasuchus,(Silesaurus,(Ornithischia,((Agnosphitys,((Guaibasaurus,(Neotheropoda,Chindesaurus)),(Buriolestes,(Pampadromaeus,(Panphagia,(Eoraptor,((Saturnalia,Chromogisaurus),(Bagualosaurus,(Jaklapalisaurus,(Nambalia,((Thecodontosaurus,Pantydraco),(Efraasia,(Plateosauravus,(Ruehleia,(((Unaysaurus,(Pradhania,Macrocollum)),((Sarahsaurus,((Ngwevu,((Coloradisaurus,(Glacialisaurus,Lufengosaurus)),(Xixipiosaurus,(Massospondylus_carinatus,(Adeopapposaurus,Leyesaurus))))),(Yunnanosaurus_huangi,((Seitaad,(Anchisaurus,((Irisosaurus,(Yizhousaurus,((Sefapanosaurus,Aardonyx),(NMQR1551,(NMRQ3314,(Blikanasaurus,((Camelotia,((Meroktenos,(Ledumahadi,Kholumolumo)),(Lessemsaurus,(Ingentia,Antetonitrus)))),((Schleitheimia,(Isanosaurus,((Tazoudasaurus,Vulcanodon),((Shunosaurus,((Spinophorosaurus,(Omeisaurus,Mamenchisaurus)),(Cetiosaurus,Neosauropoda))),(Patagosaurus,Barapasaurus))))),(Pulanesaura,Gongxianosaurus))))))))),(Mussaurus,Leonerasaurus)))),(Xingxiulong,(Jingshanosaurus,Chuxiongosaurus)))))),(Eucnemesaurus_fortis,(Riojasaurus,Eucnemesaurus_entaxonis)))),(Plateosaurus_gracilis,(Plateosaurus_ingens,Plateosaurus_engelhardti))))))))))))))))),(Staurikosaurus,Herrerasaurus)))))));

(Euparkeria,(Crurotarsi,(Marasuchus,(Silesaurus,(Ornithischia,((Agnosphitys,((Guaibasaurus,(Neotheropoda,Chindesaurus)),(Buriolestes,(Pampadromaeus,(Panphagia,(Eoraptor,((Saturnalia,Chromogisaurus),(Bagualosaurus,(Jaklapalisaurus,(Nambalia,((Thecodontosaurus,Pantydraco),(Efraasia,(Plateosauravus,(Ruehleia,(((Unaysaurus,(Pradhania,Macrocollum)),((Sarahsaurus,((Ngwevu,(Xixipiosaurus,((Coloradisaurus,(Glacialisaurus,Lufengosaurus)),(Massospondylus_carinatus,(Adeopapposaurus,Leyesaurus))))),(Yunnanosaurus_huangi,((Seitaad,(Anchisaurus,((Yizhousaurus,((Sefapanosaurus,Aardonyx),(NMQR1551,(NMRQ3314,(Blikanasaurus,((Camelotia,((Meroktenos,(Ledumahadi,Kholumolumo)),(Lessemsaurus,(Ingentia,Antetonitrus)))),((Schleitheimia,(Isanosaurus,((Tazoudasaurus,Vulcanodon),((Shunosaurus,((Spinophorosaurus,(Omeisaurus,Mamenchisaurus)),(Cetiosaurus,Neosauropoda))),(Patagosaurus,Barapasaurus))))),(Pulanesaura,Gongxianosaurus)))))))),(Leonerasaurus,(Mussaurus,Irisosaurus))))),(Xingxiulong,(Jingshanosaurus,Chuxiongosaurus)))))),(Eucnemesaurus_fortis,(Riojasaurus,Eucnemesaurus_entaxonis)))),(Plateosaurus_gracilis,(Plateosaurus_ingens,Plateosaurus_engelhardti))))))))))))))))),(Staurikosaurus,Herrerasaurus)))))));

(Euparkeria,(Crurotarsi,(Marasuchus,(Silesaurus,(Ornithischia,((Agnosphitys,((Guaibasaurus,(Neotheropoda,Chindesaurus)),(Buriolestes,(Pampadromaeus,(Panphagia,(Eoraptor,((Saturnalia,Chromogisaurus),(Bagualosaurus,(Jaklapalisaurus,(Nambalia,((Thecodontosaurus,Pantydraco),(Efraasia,(Plateosauravus,(Ruehleia,(((Unaysaurus,(Pradhania,Macrocollum)),((Sarahsaurus,((Ngwevu,(Xixipiosaurus,((Coloradisaurus,(Glacialisaurus,Lufengosaurus)),(Massospondylus_carinatus,(Adeopapposaurus,Leyesaurus))))),(Yunnanosaurus_huangi,((Seitaad,(Anchisaurus,((Irisosaurus,(Yizhousaurus,((Sefapanosaurus,Aardonyx),(NMQR1551,(NMRQ3314,(Blikanasaurus,((Camelotia,((Meroktenos,(Ledumahadi,Kholumolumo)),(Lessemsaurus,(Ingentia,Antetonitrus)))),(Pulanesaura,(Gongxianosaurus,(Schleitheimia,(Isanosaurus,((Tazoudasaurus,Vulcanodon),((Shunosaurus,((Spinophorosaurus,(Omeisaurus,Mamenchisaurus)),(Cetiosaurus,Neosauropoda))),(Patagosaurus,Barapasaurus)))))))))))))),(Mussaurus,Leonerasaurus)))),(Xingxiulong,(Jingshanosaurus,Chuxiongosaurus)))))),(Eucnemesaurus_fortis,(Riojasaurus,Eucnemesaurus_entaxonis)))),(Plateosaurus_gracilis,(Plateosaurus_ingens,Plateosaurus_engelhardti))))))))))))))))),(Staurikosaurus,Herrerasaurus)))))));

(Euparkeria,(Crurotarsi,(Marasuchus,(Silesaurus,(Ornithischia,((Agnosphitys,((Guaibasaurus,(Neotheropoda,Chindesaurus)),(Buriolestes,(Pampadromaeus,(Panphagia,(Eoraptor,((Saturnalia,Chromogisaurus),(Bagualosaurus,(Jaklapalisaurus,(Nambalia,((Thecodontosaurus,Pantydraco),(Efraasia,(Plateosauravus,(Ruehleia,(((Unaysaurus,(Pradhania,Macrocollum)),((Sarahsaurus,((Ngwevu,(Xixipiosaurus,((Coloradisaurus,(Glacialisaurus,Lufengosaurus)),(Massospondylus_carinatus,(Adeopapposaurus,Leyesaurus))))),(Yunnanosaurus_huangi,((Seitaad,(Anchisaurus,((Irisosaurus,(Yizhousaurus,((Sefapanosaurus,Aardonyx),(NMQR1551,(NMRQ3314,(Blikanasaurus,((Camelotia,((Meroktenos,(Ledumahadi,Kholumolumo)),(Lessemsaurus,(Ingentia,Antetonitrus)))),((Schleitheimia,(Isanosaurus,((Tazoudasaurus,Vulcanodon),((Shunosaurus,((Spinophorosaurus,Omeisaurus),(Mamenchisaurus,(Cetiosaurus,Neosauropoda)))),(Patagosaurus,Barapasaurus))))),(Pulanesaura,Gongxianosaurus))))))))),(Mussaurus,Leonerasaurus)))),(Xingxiulong,(Jingshanosaurus,Chuxiongosaurus)))))),(Eucnemesaurus_fortis,(Riojasaurus,Eucnemesaurus_entaxonis)))),(Plateosaurus_gracilis,(Plateosaurus_ingens,Plateosaurus_engelhardti))))))))))))))))),(Staurikosaurus,Herrerasaurus)))))));

(Euparkeria,(Crurotarsi,(Marasuchus,(Silesaurus,(Ornithischia,((Agnosphitys,((Guaibasaurus,(Neotheropoda,Chindesaurus)),((Eoraptor,((Saturnalia,Chromogisaurus),(Bagualosaurus,(Jaklapalisaurus,(Nambalia,((Thecodontosaurus,Pantydraco),(Efraasia,(Plateosauravus,(Ruehleia,(((Unaysaurus,(Pradhania,Macrocollum)),((Sarahsaurus,((Ngwevu,(Xixipiosaurus,((Coloradisaurus,(Glacialisaurus,Lufengosaurus)),(Massospondylus_carinatus,(Adeopapposaurus,Leyesaurus))))),(Yunnanosaurus_huangi,((Seitaad,(Anchisaurus,((Irisosaurus,(Yizhousaurus,((Sefapanosaurus,Aardonyx),(NMQR1551,(NMRQ3314,(Blikanasaurus,((Camelotia,((Meroktenos,(Ledumahadi,Kholumolumo)),(Lessemsaurus,(Ingentia,Antetonitrus)))),((Schleitheimia,(Isanosaurus,((Tazoudasaurus,Vulcanodon),((Shunosaurus,((Spinophorosaurus,(Omeisaurus,Mamenchisaurus)),(Cetiosaurus,Neosauropoda))),(Patagosaurus,Barapasaurus))))),(Pulanesaura,Gongxianosaurus))))))))),(Mussaurus,Leonerasaurus)))),(Xingxiulong,(Jingshanosaurus,Chuxiongosaurus)))))),(Eucnemesaurus_fortis,(Riojasaurus,Eucnemesaurus_entaxonis)))),(Plateosaurus_gracilis,(Plateosaurus_ingens,Plateosaurus_engelhardti)))))))))))),(Buriolestes,(Panphagia,Pampadromaeus))))),(Staurikosaurus,Herrerasaurus)))))));

(Euparkeria,(Crurotarsi,(Marasuchus,(Silesaurus,(Ornithischia,((Agnosphitys,((Guaibasaurus,(Neotheropoda,Chindesaurus)),(Buriolestes,(Pampadromaeus,(Panphagia,(Eoraptor,((Saturnalia,Chromogisaurus),(Bagualosaurus,(Jaklapalisaurus,(Nambalia,((Thecodontosaurus,Pantydraco),(Efraasia,(Plateosauravus,(Ruehleia,(((Unaysaurus,(Pradhania,Macrocollum)),((Sarahsaurus,((Ngwevu,(Xixipiosaurus,((Coloradisaurus,(Glacialisaurus,Lufengosaurus)),(Massospondylus_carinatus,(Adeopapposaurus,Leyesaurus))))),(Yunnanosaurus_huangi,((Seitaad,(Anchisaurus,((Irisosaurus,(Yizhousaurus,((Sefapanosaurus,Aardonyx),(NMQR1551,(NMRQ3314,(Blikanasaurus,((Camelotia,((Meroktenos,(Ledumahadi,Kholumolumo)),(Lessemsaurus,(Ingentia,Antetonitrus)))),((Schleitheimia,(Isanosaurus,((Tazoudasaurus,Vulcanodon),((Shunosaurus,(Spinophorosaurus,(Mamenchisaurus,(Omeisaurus,(Cetiosaurus,Neosauropoda))))),(Patagosaurus,Barapasaurus))))),(Pulanesaura,Gongxianosaurus))))))))),(Mussaurus,Leonerasaurus)))),(Xingxiulong,(Jingshanosaurus,Chuxiongosaurus)))))),(Eucnemesaurus_fortis,(Riojasaurus,Eucnemesaurus_entaxonis)))),(Plateosaurus_gracilis,(Plateosaurus_ingens,Plateosaurus_engelhardti))))))))))))))))),(Staurikosaurus,Herrerasaurus)))))));

(Euparkeria,(Crurotarsi,(Marasuchus,(Silesaurus,(Ornithischia,((Agnosphitys,((Guaibasaurus,(Neotheropoda,Chindesaurus)),(Buriolestes,(Pampadromaeus,(Panphagia,(Eoraptor,((Saturnalia,Chromogisaurus),(Bagualosaurus,(Jaklapalisaurus,(Nambalia,((Thecodontosaurus,Pantydraco),(Efraasia,(Plateosauravus,(Ruehleia,(((Unaysaurus,(Pradhania,Macrocollum)),((Sarahsaurus,((Ngwevu,(Xixipiosaurus,((Coloradisaurus,(Glacialisaurus,Lufengosaurus)),(Massospondylus_carinatus,(Adeopapposaurus,Leyesaurus))))),(Yunnanosaurus_huangi,((Seitaad,(Anchisaurus,((Irisosaurus,(Yizhousaurus,((Sefapanosaurus,Aardonyx),(NMQR1551,(NMRQ3314,(Blikanasaurus,((Camelotia,((Meroktenos,(Ledumahadi,Kholumolumo)),(Lessemsaurus,(Ingentia,Antetonitrus)))),((Schleitheimia,(Isanosaurus,((Tazoudasaurus,Vulcanodon),((Shunosaurus,(Spinophorosaurus,(Omeisaurus,(Mamenchisaurus,(Cetiosaurus,Neosauropoda))))),(Patagosaurus,Barapasaurus))))),(Pulanesaura,Gongxianosaurus))))))))),(Mussaurus,Leonerasaurus)))),(Xingxiulong,(Jingshanosaurus,Chuxiongosaurus)))))),(Eucnemesaurus_fortis,(Riojasaurus,Eucnemesaurus_entaxonis)))),(Plateosaurus_gracilis,(Plateosaurus_ingens,Plateosaurus_engelhardti))))))))))))))))),(Staurikosaurus,Herrerasaurus)))))));

(Euparkeria,(Crurotarsi,(Marasuchus,(Silesaurus,(Ornithischia,((Agnosphitys,((Guaibasaurus,(Neotheropoda,Chindesaurus)),(Buriolestes,((Eoraptor,((Saturnalia,Chromogisaurus),(Bagualosaurus,(Jaklapalisaurus,(Nambalia,((Thecodontosaurus,Pantydraco),(Efraasia,(Plateosauravus,(Ruehleia,(((Unaysaurus,(Pradhania,Macrocollum)),((Sarahsaurus,((Ngwevu,((Coloradisaurus,(Glacialisaurus,Lufengosaurus)),(Xixipiosaurus,(Massospondylus_carinatus,(Adeopapposaurus,Leyesaurus))))),(Yunnanosaurus_huangi,((Seitaad,(Anchisaurus,((Irisosaurus,(Yizhousaurus,((Sefapanosaurus,Aardonyx),(NMQR1551,(NMRQ3314,(Blikanasaurus,((Camelotia,((Meroktenos,(Ledumahadi,Kholumolumo)),(Lessemsaurus,(Ingentia,Antetonitrus)))),((Schleitheimia,(Isanosaurus,((Tazoudasaurus,Vulcanodon),((Shunosaurus,((Spinophorosaurus,(Omeisaurus,Mamenchisaurus)),(Cetiosaurus,Neosauropoda))),(Patagosaurus,Barapasaurus))))),(Pulanesaura,Gongxianosaurus))))))))),(Mussaurus,Leonerasaurus)))),(Xingxiulong,(Jingshanosaurus,Chuxiongosaurus)))))),(Eucnemesaurus_fortis,(Riojasaurus,Eucnemesaurus_entaxonis)))),(Plateosaurus_gracilis,(Plateosaurus_ingens,Plateosaurus_engelhardti)))))))))))),(Panphagia,Pampadromaeus))))),(Staurikosaurus,Herrerasaurus)))))));

(Euparkeria,(Crurotarsi,(Marasuchus,(Silesaurus,(Ornithischia,((Agnosphitys,((Guaibasaurus,(Neotheropoda,Chindesaurus)),(Buriolestes,((Eoraptor,((Saturnalia,Chromogisaurus),(Bagualosaurus,(Jaklapalisaurus,(Nambalia,((Thecodontosaurus,Pantydraco),(Efraasia,(Plateosauravus,(Ruehleia,(((Unaysaurus,(Pradhania,Macrocollum)),((Sarahsaurus,((Ngwevu,(Xixipiosaurus,((Coloradisaurus,(Glacialisaurus,Lufengosaurus)),(Massospondylus_carinatus,(Adeopapposaurus,Leyesaurus))))),(Yunnanosaurus_huangi,((Seitaad,(Anchisaurus,((Yizhousaurus,((Sefapanosaurus,Aardonyx),(NMQR1551,(NMRQ3314,(Blikanasaurus,((Camelotia,((Meroktenos,(Ledumahadi,Kholumolumo)),(Lessemsaurus,(Ingentia,Antetonitrus)))),((Schleitheimia,(Isanosaurus,((Tazoudasaurus,Vulcanodon),((Shunosaurus,((Spinophorosaurus,(Omeisaurus,Mamenchisaurus)),(Cetiosaurus,Neosauropoda))),(Patagosaurus,Barapasaurus))))),(Pulanesaura,Gongxianosaurus)))))))),(Leonerasaurus,(Mussaurus,Irisosaurus))))),(Xingxiulong,(Jingshanosaurus,Chuxiongosaurus)))))),(Eucnemesaurus_fortis,(Riojasaurus,Eucnemesaurus_entaxonis)))),(Plateosaurus_gracilis,(Plateosaurus_ingens,Plateosaurus_engelhardti)))))))))))),(Panphagia,Pampadromaeus))))),(Staurikosaurus,Herrerasaurus)))))));

(Euparkeria,(Crurotarsi,(Marasuchus,(Silesaurus,(Ornithischia,((Agnosphitys,((Guaibasaurus,(Neotheropoda,Chindesaurus)),(Buriolestes,((Eoraptor,((Saturnalia,Chromogisaurus),(Bagualosaurus,(Jaklapalisaurus,(Nambalia,((Thecodontosaurus,Pantydraco),(Efraasia,(Plateosauravus,(Ruehleia,(((Unaysaurus,(Pradhania,Macrocollum)),((Sarahsaurus,((Ngwevu,(Xixipiosaurus,((Coloradisaurus,(Glacialisaurus,Lufengosaurus)),(Massospondylus_carinatus,(Adeopapposaurus,Leyesaurus))))),(Yunnanosaurus_huangi,((Seitaad,(Anchisaurus,((Irisosaurus,(Yizhousaurus,((Sefapanosaurus,Aardonyx),(NMQR1551,(NMRQ3314,(Blikanasaurus,((Camelotia,((Meroktenos,(Ledumahadi,Kholumolumo)),(Lessemsaurus,(Ingentia,Antetonitrus)))),(Pulanesaura,(Gongxianosaurus,(Schleitheimia,(Isanosaurus,((Tazoudasaurus,Vulcanodon),((Shunosaurus,((Spinophorosaurus,(Omeisaurus,Mamenchisaurus)),(Cetiosaurus,Neosauropoda))),(Patagosaurus,Barapasaurus)))))))))))))),(Mussaurus,Leonerasaurus)))),(Xingxiulong,(Jingshanosaurus,Chuxiongosaurus)))))),(Eucnemesaurus_fortis,(Riojasaurus,Eucnemesaurus_entaxonis)))),(Plateosaurus_gracilis,(Plateosaurus_ingens,Plateosaurus_engelhardti)))))))))))),(Panphagia,Pampadromaeus))))),(Staurikosaurus,Herrerasaurus)))))));

(Euparkeria,(Crurotarsi,(Marasuchus,(Silesaurus,(Ornithischia,((Agnosphitys,((Guaibasaurus,(Neotheropoda,Chindesaurus)),(Buriolestes,((Eoraptor,((Saturnalia,Chromogisaurus),(Bagualosaurus,(Jaklapalisaurus,(Nambalia,((Thecodontosaurus,Pantydraco),(Efraasia,(Plateosauravus,(Ruehleia,(((Unaysaurus,(Pradhania,Macrocollum)),((Sarahsaurus,((Ngwevu,(Xixipiosaurus,((Coloradisaurus,(Glacialisaurus,Lufengosaurus)),(Massospondylus_carinatus,(Adeopapposaurus,Leyesaurus))))),(Yunnanosaurus_huangi,((Seitaad,(Anchisaurus,((Irisosaurus,(Yizhousaurus,((Sefapanosaurus,Aardonyx),(NMQR1551,(NMRQ3314,(Blikanasaurus,((Camelotia,((Meroktenos,(Ledumahadi,Kholumolumo)),(Lessemsaurus,(Ingentia,Antetonitrus)))),((Schleitheimia,(Isanosaurus,((Tazoudasaurus,Vulcanodon),((Shunosaurus,((Spinophorosaurus,Omeisaurus),(Mamenchisaurus,(Cetiosaurus,Neosauropoda)))),(Patagosaurus,Barapasaurus))))),(Pulanesaura,Gongxianosaurus))))))))),(Mussaurus,Leonerasaurus)))),(Xingxiulong,(Jingshanosaurus,Chuxiongosaurus)))))),(Eucnemesaurus_fortis,(Riojasaurus,Eucnemesaurus_entaxonis)))),(Plateosaurus_gracilis,(Plateosaurus_ingens,Plateosaurus_engelhardti)))))))))))),(Panphagia,Pampadromaeus))))),(Staurikosaurus,Herrerasaurus)))))));

(Euparkeria,(Crurotarsi,(Marasuchus,(Silesaurus,(Ornithischia,((Agnosphitys,((Guaibasaurus,(Neotheropoda,Chindesaurus)),(Buriolestes,((Eoraptor,((Saturnalia,Chromogisaurus),(Bagualosaurus,(Jaklapalisaurus,(Nambalia,((Thecodontosaurus,Pantydraco),(Efraasia,(Plateosauravus,(Ruehleia,(((Unaysaurus,(Pradhania,Macrocollum)),((Sarahsaurus,((Ngwevu,(Xixipiosaurus,((Coloradisaurus,(Glacialisaurus,Lufengosaurus)),(Massospondylus_carinatus,(Adeopapposaurus,Leyesaurus))))),(Yunnanosaurus_huangi,((Seitaad,(Anchisaurus,((Irisosaurus,(Yizhousaurus,((Sefapanosaurus,Aardonyx),(NMQR1551,(NMRQ3314,(Blikanasaurus,((Camelotia,((Meroktenos,(Ledumahadi,Kholumolumo)),(Lessemsaurus,(Ingentia,Antetonitrus)))),((Schleitheimia,(Isanosaurus,((Tazoudasaurus,Vulcanodon),((Shunosaurus,(Spinophorosaurus,(Mamenchisaurus,(Omeisaurus,(Cetiosaurus,Neosauropoda))))),(Patagosaurus,Barapasaurus))))),(Pulanesaura,Gongxianosaurus))))))))),(Mussaurus,Leonerasaurus)))),(Xingxiulong,(Jingshanosaurus,Chuxiongosaurus)))))),(Eucnemesaurus_fortis,(Riojasaurus,Eucnemesaurus_entaxonis)))),(Plateosaurus_gracilis,(Plateosaurus_ingens,Plateosaurus_engelhardti)))))))))))),(Panphagia,Pampadromaeus))))),(Staurikosaurus,Herrerasaurus)))))));

(Euparkeria,(Crurotarsi,(Marasuchus,(Silesaurus,(Ornithischia,((Agnosphitys,((Guaibasaurus,(Neotheropoda,Chindesaurus)),(Buriolestes,((Eoraptor,((Saturnalia,Chromogisaurus),(Bagualosaurus,(Jaklapalisaurus,(Nambalia,((Thecodontosaurus,Pantydraco),(Efraasia,(Plateosauravus,(Ruehleia,(((Unaysaurus,(Pradhania,Macrocollum)),((Sarahsaurus,((Ngwevu,(Xixipiosaurus,((Coloradisaurus,(Glacialisaurus,Lufengosaurus)),(Massospondylus_carinatus,(Adeopapposaurus,Leyesaurus))))),(Yunnanosaurus_huangi,((Seitaad,(Anchisaurus,((Irisosaurus,(Yizhousaurus,((Sefapanosaurus,Aardonyx),(NMQR1551,(NMRQ3314,(Blikanasaurus,((Camelotia,((Meroktenos,(Ledumahadi,Kholumolumo)),(Lessemsaurus,(Ingentia,Antetonitrus)))),((Schleitheimia,(Isanosaurus,((Tazoudasaurus,Vulcanodon),((Shunosaurus,(Spinophorosaurus,(Omeisaurus,(Mamenchisaurus,(Cetiosaurus,Neosauropoda))))),(Patagosaurus,Barapasaurus))))),(Pulanesaura,Gongxianosaurus))))))))),(Mussaurus,Leonerasaurus)))),(Xingxiulong,(Jingshanosaurus,Chuxiongosaurus)))))),(Eucnemesaurus_fortis,(Riojasaurus,Eucnemesaurus_entaxonis)))),(Plateosaurus_gracilis,(Plateosaurus_ingens,Plateosaurus_engelhardti)))))))))))),(Panphagia,Pampadromaeus))))),(Staurikosaurus,Herrerasaurus)))))));

(Euparkeria,(Crurotarsi,(Marasuchus,(Silesaurus,(Ornithischia,(Agnosphitys,(((Guaibasaurus,(Neotheropoda,Chindesaurus)),((Panphagia,(Eoraptor,((Saturnalia,Chromogisaurus),(Bagualosaurus,(Jaklapalisaurus,(Nambalia,((Thecodontosaurus,Pantydraco),(Efraasia,(Plateosauravus,(Ruehleia,(((Unaysaurus,(Pradhania,Macrocollum)),((Sarahsaurus,((Ngwevu,(Xixipiosaurus,((Coloradisaurus,(Glacialisaurus,Lufengosaurus)),(Massospondylus_carinatus,(Adeopapposaurus,Leyesaurus))))),(Yunnanosaurus_huangi,((Seitaad,(Anchisaurus,((Irisosaurus,(Yizhousaurus,((Sefapanosaurus,Aardonyx),(NMQR1551,(NMRQ3314,(Blikanasaurus,((Camelotia,((Meroktenos,(Ledumahadi,Kholumolumo)),(Lessemsaurus,(Ingentia,Antetonitrus)))),((Schleitheimia,(Isanosaurus,((Tazoudasaurus,Vulcanodon),((Shunosaurus,((Spinophorosaurus,(Omeisaurus,Mamenchisaurus)),(Cetiosaurus,Neosauropoda))),(Patagosaurus,Barapasaurus))))),(Pulanesaura,Gongxianosaurus))))))))),(Mussaurus,Leonerasaurus)))),(Xingxiulong,(Jingshanosaurus,Chuxiongosaurus)))))),(Eucnemesaurus_fortis,(Riojasaurus,Eucnemesaurus_entaxonis)))),(Plateosaurus_gracilis,(Plateosaurus_ingens,Plateosaurus_engelhardti))))))))))))),(Buriolestes,Pampadromaeus))),(Staurikosaurus,Herrerasaurus))))))));

(Euparkeria,(Crurotarsi,(Marasuchus,(Silesaurus,(Ornithischia,((Agnosphitys,((Guaibasaurus,(Neotheropoda,Chindesaurus)),((Panphagia,(Eoraptor,((Saturnalia,Chromogisaurus),(Bagualosaurus,(Jaklapalisaurus,(Nambalia,((Thecodontosaurus,Pantydraco),(Efraasia,(Plateosauravus,(Ruehleia,(((Unaysaurus,(Pradhania,Macrocollum)),((Sarahsaurus,((Ngwevu,((Coloradisaurus,(Glacialisaurus,Lufengosaurus)),(Xixipiosaurus,(Massospondylus_carinatus,(Adeopapposaurus,Leyesaurus))))),(Yunnanosaurus_huangi,((Seitaad,(Anchisaurus,((Irisosaurus,(Yizhousaurus,((Sefapanosaurus,Aardonyx),(NMQR1551,(NMRQ3314,(Blikanasaurus,((Camelotia,((Meroktenos,(Ledumahadi,Kholumolumo)),(Lessemsaurus,(Ingentia,Antetonitrus)))),((Schleitheimia,(Isanosaurus,((Tazoudasaurus,Vulcanodon),((Shunosaurus,((Spinophorosaurus,(Omeisaurus,Mamenchisaurus)),(Cetiosaurus,Neosauropoda))),(Patagosaurus,Barapasaurus))))),(Pulanesaura,Gongxianosaurus))))))))),(Mussaurus,Leonerasaurus)))),(Xingxiulong,(Jingshanosaurus,Chuxiongosaurus)))))),(Eucnemesaurus_fortis,(Riojasaurus,Eucnemesaurus_entaxonis)))),(Plateosaurus_gracilis,(Plateosaurus_ingens,Plateosaurus_engelhardti))))))))))))),(Buriolestes,Pampadromaeus)))),(Staurikosaurus,Herrerasaurus)))))));

(Euparkeria,(Crurotarsi,(Marasuchus,(Silesaurus,(Ornithischia,((Agnosphitys,((Guaibasaurus,(Neotheropoda,Chindesaurus)),((Panphagia,(Eoraptor,((Saturnalia,Chromogisaurus),(Bagualosaurus,(Jaklapalisaurus,(Nambalia,((Thecodontosaurus,Pantydraco),(Efraasia,(Plateosauravus,(Ruehleia,(((Unaysaurus,(Pradhania,Macrocollum)),((Sarahsaurus,((Ngwevu,(Xixipiosaurus,((Coloradisaurus,(Glacialisaurus,Lufengosaurus)),(Massospondylus_carinatus,(Adeopapposaurus,Leyesaurus))))),(Yunnanosaurus_huangi,((Seitaad,(Anchisaurus,((Yizhousaurus,((Sefapanosaurus,Aardonyx),(NMQR1551,(NMRQ3314,(Blikanasaurus,((Camelotia,((Meroktenos,(Ledumahadi,Kholumolumo)),(Lessemsaurus,(Ingentia,Antetonitrus)))),((Schleitheimia,(Isanosaurus,((Tazoudasaurus,Vulcanodon),((Shunosaurus,((Spinophorosaurus,(Omeisaurus,Mamenchisaurus)),(Cetiosaurus,Neosauropoda))),(Patagosaurus,Barapasaurus))))),(Pulanesaura,Gongxianosaurus)))))))),(Leonerasaurus,(Mussaurus,Irisosaurus))))),(Xingxiulong,(Jingshanosaurus,Chuxiongosaurus)))))),(Eucnemesaurus_fortis,(Riojasaurus,Eucnemesaurus_entaxonis)))),(Plateosaurus_gracilis,(Plateosaurus_ingens,Plateosaurus_engelhardti))))))))))))),(Buriolestes,Pampadromaeus)))),(Staurikosaurus,Herrerasaurus)))))));

(Euparkeria,(Crurotarsi,(Marasuchus,(Silesaurus,(Ornithischia,((Agnosphitys,((Guaibasaurus,(Neotheropoda,Chindesaurus)),((Panphagia,(Eoraptor,((Saturnalia,Chromogisaurus),(Bagualosaurus,(Jaklapalisaurus,(Nambalia,((Thecodontosaurus,Pantydraco),(Efraasia,(Plateosauravus,(Ruehleia,(((Unaysaurus,(Pradhania,Macrocollum)),((Sarahsaurus,((Ngwevu,(Xixipiosaurus,((Coloradisaurus,(Glacialisaurus,Lufengosaurus)),(Massospondylus_carinatus,(Adeopapposaurus,Leyesaurus))))),(Yunnanosaurus_huangi,((Seitaad,(Anchisaurus,((Irisosaurus,(Yizhousaurus,((Sefapanosaurus,Aardonyx),(NMQR1551,(NMRQ3314,(Blikanasaurus,((Camelotia,((Meroktenos,(Ledumahadi,Kholumolumo)),(Lessemsaurus,(Ingentia,Antetonitrus)))),((Schleitheimia,(Isanosaurus,((Tazoudasaurus,Vulcanodon),((Shunosaurus,((Spinophorosaurus,Omeisaurus),(Mamenchisaurus,(Cetiosaurus,Neosauropoda)))),(Patagosaurus,Barapasaurus))))),(Pulanesaura,Gongxianosaurus))))))))),(Mussaurus,Leonerasaurus)))),(Xingxiulong,(Jingshanosaurus,Chuxiongosaurus)))))),(Eucnemesaurus_fortis,(Riojasaurus,Eucnemesaurus_entaxonis)))),(Plateosaurus_gracilis,(Plateosaurus_ingens,Plateosaurus_engelhardti))))))))))))),(Buriolestes,Pampadromaeus)))),(Staurikosaurus,Herrerasaurus)))))));

(Euparkeria,(Crurotarsi,(Marasuchus,(Silesaurus,(Ornithischia,((Agnosphitys,((Guaibasaurus,(Neotheropoda,Chindesaurus)),((Panphagia,(Eoraptor,((Saturnalia,Chromogisaurus),(Bagualosaurus,(Jaklapalisaurus,(Nambalia,((Thecodontosaurus,Pantydraco),(Efraasia,(Plateosauravus,(Ruehleia,(((Unaysaurus,(Pradhania,Macrocollum)),((Sarahsaurus,((Ngwevu,(Xixipiosaurus,((Coloradisaurus,(Glacialisaurus,Lufengosaurus)),(Massospondylus_carinatus,(Adeopapposaurus,Leyesaurus))))),(Yunnanosaurus_huangi,((Seitaad,(Anchisaurus,((Irisosaurus,(Yizhousaurus,((Sefapanosaurus,Aardonyx),(NMQR1551,(NMRQ3314,(Blikanasaurus,((Camelotia,((Meroktenos,(Ledumahadi,Kholumolumo)),(Lessemsaurus,(Ingentia,Antetonitrus)))),((Schleitheimia,(Isanosaurus,((Tazoudasaurus,Vulcanodon),((Shunosaurus,(Spinophorosaurus,(Mamenchisaurus,(Omeisaurus,(Cetiosaurus,Neosauropoda))))),(Patagosaurus,Barapasaurus))))),(Pulanesaura,Gongxianosaurus))))))))),(Mussaurus,Leonerasaurus)))),(Xingxiulong,(Jingshanosaurus,Chuxiongosaurus)))))),(Eucnemesaurus_fortis,(Riojasaurus,Eucnemesaurus_entaxonis)))),(Plateosaurus_gracilis,(Plateosaurus_ingens,Plateosaurus_engelhardti))))))))))))),(Buriolestes,Pampadromaeus)))),(Staurikosaurus,Herrerasaurus)))))));

(Euparkeria,(Crurotarsi,(Marasuchus,(Silesaurus,(Ornithischia,((Agnosphitys,((Guaibasaurus,(Neotheropoda,Chindesaurus)),((Panphagia,(Eoraptor,((Saturnalia,Chromogisaurus),(Bagualosaurus,(Jaklapalisaurus,(Nambalia,((Thecodontosaurus,Pantydraco),(Efraasia,(Plateosauravus,(Ruehleia,(((Unaysaurus,(Pradhania,Macrocollum)),((Sarahsaurus,((Ngwevu,(Xixipiosaurus,((Coloradisaurus,(Glacialisaurus,Lufengosaurus)),(Massospondylus_carinatus,(Adeopapposaurus,Leyesaurus))))),(Yunnanosaurus_huangi,((Seitaad,(Anchisaurus,((Irisosaurus,(Yizhousaurus,((Sefapanosaurus,Aardonyx),(NMQR1551,(NMRQ3314,(Blikanasaurus,((Camelotia,((Meroktenos,(Ledumahadi,Kholumolumo)),(Lessemsaurus,(Ingentia,Antetonitrus)))),((Schleitheimia,(Isanosaurus,((Tazoudasaurus,Vulcanodon),((Shunosaurus,(Spinophorosaurus,(Omeisaurus,(Mamenchisaurus,(Cetiosaurus,Neosauropoda))))),(Patagosaurus,Barapasaurus))))),(Pulanesaura,Gongxianosaurus))))))))),(Mussaurus,Leonerasaurus)))),(Xingxiulong,(Jingshanosaurus,Chuxiongosaurus)))))),(Eucnemesaurus_fortis,(Riojasaurus,Eucnemesaurus_entaxonis)))),(Plateosaurus_gracilis,(Plateosaurus_ingens,Plateosaurus_engelhardti))))))))))))),(Buriolestes,Pampadromaeus)))),(Staurikosaurus,Herrerasaurus)))))));

(Euparkeria,(Crurotarsi,(Marasuchus,(Silesaurus,(Ornithischia,((Agnosphitys,((Guaibasaurus,(Neotheropoda,Chindesaurus)),(Buriolestes,(Panphagia,(Eoraptor,((Pampadromaeus,(Saturnalia,Chromogisaurus)),(Bagualosaurus,(Jaklapalisaurus,(Nambalia,((Thecodontosaurus,Pantydraco),(Efraasia,(Plateosauravus,(Ruehleia,(((Unaysaurus,(Pradhania,Macrocollum)),((Sarahsaurus,((Ngwevu,((Coloradisaurus,(Glacialisaurus,Lufengosaurus)),(Xixipiosaurus,(Massospondylus_carinatus,(Adeopapposaurus,Leyesaurus))))),(Yunnanosaurus_huangi,((Seitaad,(Anchisaurus,((Yizhousaurus,((Sefapanosaurus,Aardonyx),(NMQR1551,(NMRQ3314,(Blikanasaurus,((Camelotia,((Meroktenos,(Ledumahadi,Kholumolumo)),(Lessemsaurus,(Ingentia,Antetonitrus)))),((Schleitheimia,(Isanosaurus,((Tazoudasaurus,Vulcanodon),((Shunosaurus,((Spinophorosaurus,(Omeisaurus,Mamenchisaurus)),(Cetiosaurus,Neosauropoda))),(Patagosaurus,Barapasaurus))))),(Pulanesaura,Gongxianosaurus)))))))),(Leonerasaurus,(Mussaurus,Irisosaurus))))),(Xingxiulong,(Jingshanosaurus,Chuxiongosaurus)))))),(Eucnemesaurus_fortis,(Riojasaurus,Eucnemesaurus_entaxonis)))),(Plateosaurus_gracilis,(Plateosaurus_ingens,Plateosaurus_engelhardti)))))))))))))))),(Staurikosaurus,Herrerasaurus)))))));

(Euparkeria,(Crurotarsi,(Marasuchus,(Silesaurus,(Ornithischia,((Agnosphitys,((Guaibasaurus,(Neotheropoda,Chindesaurus)),(Buriolestes,(Panphagia,(Eoraptor,((Pampadromaeus,(Saturnalia,Chromogisaurus)),(Bagualosaurus,(Jaklapalisaurus,(Nambalia,((Thecodontosaurus,Pantydraco),(Efraasia,(Plateosauravus,(Ruehleia,(((Unaysaurus,(Pradhania,Macrocollum)),((Sarahsaurus,((Ngwevu,((Coloradisaurus,(Glacialisaurus,Lufengosaurus)),(Xixipiosaurus,(Massospondylus_carinatus,(Adeopapposaurus,Leyesaurus))))),(Yunnanosaurus_huangi,((Seitaad,(Anchisaurus,((Irisosaurus,(Yizhousaurus,((Sefapanosaurus,Aardonyx),(NMQR1551,(NMRQ3314,(Blikanasaurus,((Camelotia,((Meroktenos,(Ledumahadi,Kholumolumo)),(Lessemsaurus,(Ingentia,Antetonitrus)))),(Pulanesaura,(Gongxianosaurus,(Schleitheimia,(Isanosaurus,((Tazoudasaurus,Vulcanodon),((Shunosaurus,((Spinophorosaurus,(Omeisaurus,Mamenchisaurus)),(Cetiosaurus,Neosauropoda))),(Patagosaurus,Barapasaurus)))))))))))))),(Mussaurus,Leonerasaurus)))),(Xingxiulong,(Jingshanosaurus,Chuxiongosaurus)))))),(Eucnemesaurus_fortis,(Riojasaurus,Eucnemesaurus_entaxonis)))),(Plateosaurus_gracilis,(Plateosaurus_ingens,Plateosaurus_engelhardti)))))))))))))))),(Staurikosaurus,Herrerasaurus)))))));

(Euparkeria,(Crurotarsi,(Marasuchus,(Silesaurus,(Ornithischia,((Agnosphitys,((Guaibasaurus,(Neotheropoda,Chindesaurus)),(Buriolestes,(Panphagia,(Eoraptor,((Pampadromaeus,(Saturnalia,Chromogisaurus)),(Bagualosaurus,(Jaklapalisaurus,(Nambalia,((Thecodontosaurus,Pantydraco),(Efraasia,(Plateosauravus,(Ruehleia,(((Unaysaurus,(Pradhania,Macrocollum)),((Sarahsaurus,((Ngwevu,((Coloradisaurus,(Glacialisaurus,Lufengosaurus)),(Xixipiosaurus,(Massospondylus_carinatus,(Adeopapposaurus,Leyesaurus))))),(Yunnanosaurus_huangi,((Seitaad,(Anchisaurus,((Irisosaurus,(Yizhousaurus,((Sefapanosaurus,Aardonyx),(NMQR1551,(NMRQ3314,(Blikanasaurus,((Camelotia,((Meroktenos,(Ledumahadi,Kholumolumo)),(Lessemsaurus,(Ingentia,Antetonitrus)))),((Schleitheimia,(Isanosaurus,((Tazoudasaurus,Vulcanodon),((Shunosaurus,((Spinophorosaurus,Omeisaurus),(Mamenchisaurus,(Cetiosaurus,Neosauropoda)))),(Patagosaurus,Barapasaurus))))),(Pulanesaura,Gongxianosaurus))))))))),(Mussaurus,Leonerasaurus)))),(Xingxiulong,(Jingshanosaurus,Chuxiongosaurus)))))),(Eucnemesaurus_fortis,(Riojasaurus,Eucnemesaurus_entaxonis)))),(Plateosaurus_gracilis,(Plateosaurus_ingens,Plateosaurus_engelhardti)))))))))))))))),(Staurikosaurus,Herrerasaurus)))))));

(Euparkeria,(Crurotarsi,(Marasuchus,(Silesaurus,(Ornithischia,((Agnosphitys,((Guaibasaurus,(Neotheropoda,Chindesaurus)),(Buriolestes,(Panphagia,(Eoraptor,((Saturnalia,Chromogisaurus),(Pampadromaeus,(Bagualosaurus,(Jaklapalisaurus,(Nambalia,((Thecodontosaurus,Pantydraco),(Efraasia,(Plateosauravus,(Ruehleia,(((Unaysaurus,(Pradhania,Macrocollum)),((Sarahsaurus,((Ngwevu,((Coloradisaurus,(Glacialisaurus,Lufengosaurus)),(Xixipiosaurus,(Massospondylus_carinatus,(Adeopapposaurus,Leyesaurus))))),(Yunnanosaurus_huangi,((Seitaad,(Anchisaurus,((Irisosaurus,(Yizhousaurus,((Sefapanosaurus,Aardonyx),(NMQR1551,(NMRQ3314,(Blikanasaurus,((Camelotia,((Meroktenos,(Ledumahadi,Kholumolumo)),(Lessemsaurus,(Ingentia,Antetonitrus)))),((Schleitheimia,(Isanosaurus,((Tazoudasaurus,Vulcanodon),((Shunosaurus,((Spinophorosaurus,(Omeisaurus,Mamenchisaurus)),(Cetiosaurus,Neosauropoda))),(Patagosaurus,Barapasaurus))))),(Pulanesaura,Gongxianosaurus))))))))),(Mussaurus,Leonerasaurus)))),(Xingxiulong,(Jingshanosaurus,Chuxiongosaurus)))))),(Eucnemesaurus_fortis,(Riojasaurus,Eucnemesaurus_entaxonis)))),(Plateosaurus_gracilis,(Plateosaurus_ingens,Plateosaurus_engelhardti))))))))))))))))),(Staurikosaurus,Herrerasaurus)))))));

(Euparkeria,(Crurotarsi,(Marasuchus,(Silesaurus,(Ornithischia,((Agnosphitys,((Guaibasaurus,(Neotheropoda,Chindesaurus)),(Buriolestes,(Panphagia,(Eoraptor,((Pampadromaeus,(Saturnalia,Chromogisaurus)),(Bagualosaurus,(Jaklapalisaurus,(Nambalia,((Thecodontosaurus,Pantydraco),(Efraasia,(Plateosauravus,(Ruehleia,(((Unaysaurus,(Pradhania,Macrocollum)),((Sarahsaurus,((Ngwevu,((Coloradisaurus,(Glacialisaurus,Lufengosaurus)),(Xixipiosaurus,(Massospondylus_carinatus,(Adeopapposaurus,Leyesaurus))))),(Yunnanosaurus_huangi,((Seitaad,(Anchisaurus,((Irisosaurus,(Yizhousaurus,((Sefapanosaurus,Aardonyx),(NMQR1551,(NMRQ3314,(Blikanasaurus,((Camelotia,((Meroktenos,(Ledumahadi,Kholumolumo)),(Lessemsaurus,(Ingentia,Antetonitrus)))),((Schleitheimia,(Isanosaurus,((Tazoudasaurus,Vulcanodon),((Shunosaurus,(Spinophorosaurus,(Mamenchisaurus,(Omeisaurus,(Cetiosaurus,Neosauropoda))))),(Patagosaurus,Barapasaurus))))),(Pulanesaura,Gongxianosaurus))))))))),(Mussaurus,Leonerasaurus)))),(Xingxiulong,(Jingshanosaurus,Chuxiongosaurus)))))),(Eucnemesaurus_fortis,(Riojasaurus,Eucnemesaurus_entaxonis)))),(Plateosaurus_gracilis,(Plateosaurus_ingens,Plateosaurus_engelhardti)))))))))))))))),(Staurikosaurus,Herrerasaurus)))))));

(Euparkeria,(Crurotarsi,(Marasuchus,(Silesaurus,(Ornithischia,((Agnosphitys,((Guaibasaurus,(Neotheropoda,Chindesaurus)),(Buriolestes,(Panphagia,(Eoraptor,((Pampadromaeus,(Saturnalia,Chromogisaurus)),(Bagualosaurus,(Jaklapalisaurus,(Nambalia,((Thecodontosaurus,Pantydraco),(Efraasia,(Plateosauravus,(Ruehleia,(((Unaysaurus,(Pradhania,Macrocollum)),((Sarahsaurus,((Ngwevu,((Coloradisaurus,(Glacialisaurus,Lufengosaurus)),(Xixipiosaurus,(Massospondylus_carinatus,(Adeopapposaurus,Leyesaurus))))),(Yunnanosaurus_huangi,((Seitaad,(Anchisaurus,((Irisosaurus,(Yizhousaurus,((Sefapanosaurus,Aardonyx),(NMQR1551,(NMRQ3314,(Blikanasaurus,((Camelotia,((Meroktenos,(Ledumahadi,Kholumolumo)),(Lessemsaurus,(Ingentia,Antetonitrus)))),((Schleitheimia,(Isanosaurus,((Tazoudasaurus,Vulcanodon),((Shunosaurus,(Spinophorosaurus,(Omeisaurus,(Mamenchisaurus,(Cetiosaurus,Neosauropoda))))),(Patagosaurus,Barapasaurus))))),(Pulanesaura,Gongxianosaurus))))))))),(Mussaurus,Leonerasaurus)))),(Xingxiulong,(Jingshanosaurus,Chuxiongosaurus)))))),(Eucnemesaurus_fortis,(Riojasaurus,Eucnemesaurus_entaxonis)))),(Plateosaurus_gracilis,(Plateosaurus_ingens,Plateosaurus_engelhardti)))))))))))))))),(Staurikosaurus,Herrerasaurus)))))));

(Euparkeria,(Crurotarsi,(Marasuchus,(Silesaurus,(Ornithischia,((Agnosphitys,((Guaibasaurus,(Neotheropoda,Chindesaurus)),(Buriolestes,(Panphagia,(Eoraptor,((Pampadromaeus,(Saturnalia,Chromogisaurus)),(Bagualosaurus,(Jaklapalisaurus,(Nambalia,((Thecodontosaurus,Pantydraco),(Efraasia,(Plateosauravus,(Ruehleia,(((Unaysaurus,(Pradhania,Macrocollum)),((Sarahsaurus,((Ngwevu,(Xixipiosaurus,((Coloradisaurus,(Glacialisaurus,Lufengosaurus)),(Massospondylus_carinatus,(Adeopapposaurus,Leyesaurus))))),(Yunnanosaurus_huangi,((Seitaad,(Anchisaurus,((Yizhousaurus,((Sefapanosaurus,Aardonyx),(NMQR1551,(NMRQ3314,(Blikanasaurus,((Camelotia,((Meroktenos,(Ledumahadi,Kholumolumo)),(Lessemsaurus,(Ingentia,Antetonitrus)))),(Pulanesaura,(Gongxianosaurus,(Schleitheimia,(Isanosaurus,((Tazoudasaurus,Vulcanodon),((Shunosaurus,((Spinophorosaurus,(Omeisaurus,Mamenchisaurus)),(Cetiosaurus,Neosauropoda))),(Patagosaurus,Barapasaurus))))))))))))),(Leonerasaurus,(Mussaurus,Irisosaurus))))),(Xingxiulong,(Jingshanosaurus,Chuxiongosaurus)))))),(Eucnemesaurus_fortis,(Riojasaurus,Eucnemesaurus_entaxonis)))),(Plateosaurus_gracilis,(Plateosaurus_ingens,Plateosaurus_engelhardti)))))))))))))))),(Staurikosaurus,Herrerasaurus)))))));

(Euparkeria,(Crurotarsi,(Marasuchus,(Silesaurus,(Ornithischia,((Agnosphitys,((Guaibasaurus,(Neotheropoda,Chindesaurus)),(Buriolestes,(Panphagia,(Eoraptor,((Pampadromaeus,(Saturnalia,Chromogisaurus)),(Bagualosaurus,(Jaklapalisaurus,(Nambalia,((Thecodontosaurus,Pantydraco),(Efraasia,(Plateosauravus,(Ruehleia,(((Unaysaurus,(Pradhania,Macrocollum)),((Sarahsaurus,((Ngwevu,(Xixipiosaurus,((Coloradisaurus,(Glacialisaurus,Lufengosaurus)),(Massospondylus_carinatus,(Adeopapposaurus,Leyesaurus))))),(Yunnanosaurus_huangi,((Seitaad,(Anchisaurus,((Yizhousaurus,((Sefapanosaurus,Aardonyx),(NMQR1551,(NMRQ3314,(Blikanasaurus,((Camelotia,((Meroktenos,(Ledumahadi,Kholumolumo)),(Lessemsaurus,(Ingentia,Antetonitrus)))),((Schleitheimia,(Isanosaurus,((Tazoudasaurus,Vulcanodon),((Shunosaurus,((Spinophorosaurus,Omeisaurus),(Mamenchisaurus,(Cetiosaurus,Neosauropoda)))),(Patagosaurus,Barapasaurus))))),(Pulanesaura,Gongxianosaurus)))))))),(Leonerasaurus,(Mussaurus,Irisosaurus))))),(Xingxiulong,(Jingshanosaurus,Chuxiongosaurus)))))),(Eucnemesaurus_fortis,(Riojasaurus,Eucnemesaurus_entaxonis)))),(Plateosaurus_gracilis,(Plateosaurus_ingens,Plateosaurus_engelhardti)))))))))))))))),(Staurikosaurus,Herrerasaurus)))))));

(Euparkeria,(Crurotarsi,(Marasuchus,(Silesaurus,(Ornithischia,((Agnosphitys,((Guaibasaurus,(Neotheropoda,Chindesaurus)),(Buriolestes,(Panphagia,(Eoraptor,((Saturnalia,Chromogisaurus),(Pampadromaeus,(Bagualosaurus,(Jaklapalisaurus,(Nambalia,((Thecodontosaurus,Pantydraco),(Efraasia,(Plateosauravus,(Ruehleia,(((Unaysaurus,(Pradhania,Macrocollum)),((Sarahsaurus,((Ngwevu,(Xixipiosaurus,((Coloradisaurus,(Glacialisaurus,Lufengosaurus)),(Massospondylus_carinatus,(Adeopapposaurus,Leyesaurus))))),(Yunnanosaurus_huangi,((Seitaad,(Anchisaurus,((Yizhousaurus,((Sefapanosaurus,Aardonyx),(NMQR1551,(NMRQ3314,(Blikanasaurus,((Camelotia,((Meroktenos,(Ledumahadi,Kholumolumo)),(Lessemsaurus,(Ingentia,Antetonitrus)))),((Schleitheimia,(Isanosaurus,((Tazoudasaurus,Vulcanodon),((Shunosaurus,((Spinophorosaurus,(Omeisaurus,Mamenchisaurus)),(Cetiosaurus,Neosauropoda))),(Patagosaurus,Barapasaurus))))),(Pulanesaura,Gongxianosaurus)))))))),(Leonerasaurus,(Mussaurus,Irisosaurus))))),(Xingxiulong,(Jingshanosaurus,Chuxiongosaurus)))))),(Eucnemesaurus_fortis,(Riojasaurus,Eucnemesaurus_entaxonis)))),(Plateosaurus_gracilis,(Plateosaurus_ingens,Plateosaurus_engelhardti))))))))))))))))),(Staurikosaurus,Herrerasaurus)))))));

(Euparkeria,(Crurotarsi,(Marasuchus,(Silesaurus,(Ornithischia,((Agnosphitys,((Guaibasaurus,(Neotheropoda,Chindesaurus)),(Buriolestes,(Panphagia,(Eoraptor,((Pampadromaeus,(Saturnalia,Chromogisaurus)),(Bagualosaurus,(Jaklapalisaurus,(Nambalia,((Thecodontosaurus,Pantydraco),(Efraasia,(Plateosauravus,(Ruehleia,(((Unaysaurus,(Pradhania,Macrocollum)),((Sarahsaurus,((Ngwevu,(Xixipiosaurus,((Coloradisaurus,(Glacialisaurus,Lufengosaurus)),(Massospondylus_carinatus,(Adeopapposaurus,Leyesaurus))))),(Yunnanosaurus_huangi,((Seitaad,(Anchisaurus,((Yizhousaurus,((Sefapanosaurus,Aardonyx),(NMQR1551,(NMRQ3314,(Blikanasaurus,((Camelotia,((Meroktenos,(Ledumahadi,Kholumolumo)),(Lessemsaurus,(Ingentia,Antetonitrus)))),((Schleitheimia,(Isanosaurus,((Tazoudasaurus,Vulcanodon),((Shunosaurus,(Spinophorosaurus,(Mamenchisaurus,(Omeisaurus,(Cetiosaurus,Neosauropoda))))),(Patagosaurus,Barapasaurus))))),(Pulanesaura,Gongxianosaurus)))))))),(Leonerasaurus,(Mussaurus,Irisosaurus))))),(Xingxiulong,(Jingshanosaurus,Chuxiongosaurus)))))),(Eucnemesaurus_fortis,(Riojasaurus,Eucnemesaurus_entaxonis)))),(Plateosaurus_gracilis,(Plateosaurus_ingens,Plateosaurus_engelhardti)))))))))))))))),(Staurikosaurus,Herrerasaurus)))))));

(Euparkeria,(Crurotarsi,(Marasuchus,(Silesaurus,(Ornithischia,((Agnosphitys,((Guaibasaurus,(Neotheropoda,Chindesaurus)),(Buriolestes,(Panphagia,(Eoraptor,((Pampadromaeus,(Saturnalia,Chromogisaurus)),(Bagualosaurus,(Jaklapalisaurus,(Nambalia,((Thecodontosaurus,Pantydraco),(Efraasia,(Plateosauravus,(Ruehleia,(((Unaysaurus,(Pradhania,Macrocollum)),((Sarahsaurus,((Ngwevu,(Xixipiosaurus,((Coloradisaurus,(Glacialisaurus,Lufengosaurus)),(Massospondylus_carinatus,(Adeopapposaurus,Leyesaurus))))),(Yunnanosaurus_huangi,((Seitaad,(Anchisaurus,((Yizhousaurus,((Sefapanosaurus,Aardonyx),(NMQR1551,(NMRQ3314,(Blikanasaurus,((Camelotia,((Meroktenos,(Ledumahadi,Kholumolumo)),(Lessemsaurus,(Ingentia,Antetonitrus)))),((Schleitheimia,(Isanosaurus,((Tazoudasaurus,Vulcanodon),((Shunosaurus,(Spinophorosaurus,(Omeisaurus,(Mamenchisaurus,(Cetiosaurus,Neosauropoda))))),(Patagosaurus,Barapasaurus))))),(Pulanesaura,Gongxianosaurus)))))))),(Leonerasaurus,(Mussaurus,Irisosaurus))))),(Xingxiulong,(Jingshanosaurus,Chuxiongosaurus)))))),(Eucnemesaurus_fortis,(Riojasaurus,Eucnemesaurus_entaxonis)))),(Plateosaurus_gracilis,(Plateosaurus_ingens,Plateosaurus_engelhardti)))))))))))))))),(Staurikosaurus,Herrerasaurus)))))));

(Euparkeria,(Crurotarsi,(Marasuchus,(Silesaurus,(Ornithischia,((Agnosphitys,((Guaibasaurus,(Neotheropoda,Chindesaurus)),(Buriolestes,(Panphagia,(Eoraptor,((Pampadromaeus,(Saturnalia,Chromogisaurus)),(Bagualosaurus,(Jaklapalisaurus,(Nambalia,((Thecodontosaurus,Pantydraco),(Efraasia,(Plateosauravus,(Ruehleia,(((Unaysaurus,(Pradhania,Macrocollum)),((Sarahsaurus,((Ngwevu,(Xixipiosaurus,((Coloradisaurus,(Glacialisaurus,Lufengosaurus)),(Massospondylus_carinatus,(Adeopapposaurus,Leyesaurus))))),(Yunnanosaurus_huangi,((Seitaad,(Anchisaurus,((Irisosaurus,(Yizhousaurus,((Sefapanosaurus,Aardonyx),(NMQR1551,(NMRQ3314,(Blikanasaurus,((Camelotia,((Meroktenos,(Ledumahadi,Kholumolumo)),(Lessemsaurus,(Ingentia,Antetonitrus)))),(Pulanesaura,(Gongxianosaurus,(Schleitheimia,(Isanosaurus,((Tazoudasaurus,Vulcanodon),((Shunosaurus,((Spinophorosaurus,Omeisaurus),(Mamenchisaurus,(Cetiosaurus,Neosauropoda)))),(Patagosaurus,Barapasaurus)))))))))))))),(Mussaurus,Leonerasaurus)))),(Xingxiulong,(Jingshanosaurus,Chuxiongosaurus)))))),(Eucnemesaurus_fortis,(Riojasaurus,Eucnemesaurus_entaxonis)))),(Plateosaurus_gracilis,(Plateosaurus_ingens,Plateosaurus_engelhardti)))))))))))))))),(Staurikosaurus,Herrerasaurus)))))));

(Euparkeria,(Crurotarsi,(Marasuchus,(Silesaurus,(Ornithischia,((Agnosphitys,((Guaibasaurus,(Neotheropoda,Chindesaurus)),(Buriolestes,(Panphagia,(Eoraptor,((Saturnalia,Chromogisaurus),(Pampadromaeus,(Bagualosaurus,(Jaklapalisaurus,(Nambalia,((Thecodontosaurus,Pantydraco),(Efraasia,(Plateosauravus,(Ruehleia,(((Unaysaurus,(Pradhania,Macrocollum)),((Sarahsaurus,((Ngwevu,(Xixipiosaurus,((Coloradisaurus,(Glacialisaurus,Lufengosaurus)),(Massospondylus_carinatus,(Adeopapposaurus,Leyesaurus))))),(Yunnanosaurus_huangi,((Seitaad,(Anchisaurus,((Irisosaurus,(Yizhousaurus,((Sefapanosaurus,Aardonyx),(NMQR1551,(NMRQ3314,(Blikanasaurus,((Camelotia,((Meroktenos,(Ledumahadi,Kholumolumo)),(Lessemsaurus,(Ingentia,Antetonitrus)))),(Pulanesaura,(Gongxianosaurus,(Schleitheimia,(Isanosaurus,((Tazoudasaurus,Vulcanodon),((Shunosaurus,((Spinophorosaurus,(Omeisaurus,Mamenchisaurus)),(Cetiosaurus,Neosauropoda))),(Patagosaurus,Barapasaurus)))))))))))))),(Mussaurus,Leonerasaurus)))),(Xingxiulong,(Jingshanosaurus,Chuxiongosaurus)))))),(Eucnemesaurus_fortis,(Riojasaurus,Eucnemesaurus_entaxonis)))),(Plateosaurus_gracilis,(Plateosaurus_ingens,Plateosaurus_engelhardti))))))))))))))))),(Staurikosaurus,Herrerasaurus)))))));

(Euparkeria,(Crurotarsi,(Marasuchus,(Silesaurus,(Ornithischia,((Agnosphitys,((Guaibasaurus,(Neotheropoda,Chindesaurus)),(Buriolestes,(Panphagia,(Eoraptor,((Pampadromaeus,(Saturnalia,Chromogisaurus)),(Bagualosaurus,(Jaklapalisaurus,(Nambalia,((Thecodontosaurus,Pantydraco),(Efraasia,(Plateosauravus,(Ruehleia,(((Unaysaurus,(Pradhania,Macrocollum)),((Sarahsaurus,((Ngwevu,(Xixipiosaurus,((Coloradisaurus,(Glacialisaurus,Lufengosaurus)),(Massospondylus_carinatus,(Adeopapposaurus,Leyesaurus))))),(Yunnanosaurus_huangi,((Seitaad,(Anchisaurus,((Irisosaurus,(Yizhousaurus,((Sefapanosaurus,Aardonyx),(NMQR1551,(NMRQ3314,(Blikanasaurus,((Camelotia,((Meroktenos,(Ledumahadi,Kholumolumo)),(Lessemsaurus,(Ingentia,Antetonitrus)))),(Pulanesaura,(Gongxianosaurus,(Schleitheimia,(Isanosaurus,((Tazoudasaurus,Vulcanodon),((Shunosaurus,(Spinophorosaurus,(Mamenchisaurus,(Omeisaurus,(Cetiosaurus,Neosauropoda))))),(Patagosaurus,Barapasaurus)))))))))))))),(Mussaurus,Leonerasaurus)))),(Xingxiulong,(Jingshanosaurus,Chuxiongosaurus)))))),(Eucnemesaurus_fortis,(Riojasaurus,Eucnemesaurus_entaxonis)))),(Plateosaurus_gracilis,(Plateosaurus_ingens,Plateosaurus_engelhardti)))))))))))))))),(Staurikosaurus,Herrerasaurus)))))));

(Euparkeria,(Crurotarsi,(Marasuchus,(Silesaurus,(Ornithischia,((Agnosphitys,((Guaibasaurus,(Neotheropoda,Chindesaurus)),(Buriolestes,(Panphagia,(Eoraptor,((Pampadromaeus,(Saturnalia,Chromogisaurus)),(Bagualosaurus,(Jaklapalisaurus,(Nambalia,((Thecodontosaurus,Pantydraco),(Efraasia,(Plateosauravus,(Ruehleia,(((Unaysaurus,(Pradhania,Macrocollum)),((Sarahsaurus,((Ngwevu,(Xixipiosaurus,((Coloradisaurus,(Glacialisaurus,Lufengosaurus)),(Massospondylus_carinatus,(Adeopapposaurus,Leyesaurus))))),(Yunnanosaurus_huangi,((Seitaad,(Anchisaurus,((Irisosaurus,(Yizhousaurus,((Sefapanosaurus,Aardonyx),(NMQR1551,(NMRQ3314,(Blikanasaurus,((Camelotia,((Meroktenos,(Ledumahadi,Kholumolumo)),(Lessemsaurus,(Ingentia,Antetonitrus)))),(Pulanesaura,(Gongxianosaurus,(Schleitheimia,(Isanosaurus,((Tazoudasaurus,Vulcanodon),((Shunosaurus,(Spinophorosaurus,(Omeisaurus,(Mamenchisaurus,(Cetiosaurus,Neosauropoda))))),(Patagosaurus,Barapasaurus)))))))))))))),(Mussaurus,Leonerasaurus)))),(Xingxiulong,(Jingshanosaurus,Chuxiongosaurus)))))),(Eucnemesaurus_fortis,(Riojasaurus,Eucnemesaurus_entaxonis)))),(Plateosaurus_gracilis,(Plateosaurus_ingens,Plateosaurus_engelhardti)))))))))))))))),(Staurikosaurus,Herrerasaurus)))))));

(Euparkeria,(Crurotarsi,(Marasuchus,(Silesaurus,(Ornithischia,((Agnosphitys,((Guaibasaurus,(Neotheropoda,Chindesaurus)),(Buriolestes,(Panphagia,(Eoraptor,((Saturnalia,Chromogisaurus),(Pampadromaeus,(Bagualosaurus,(Jaklapalisaurus,(Nambalia,((Thecodontosaurus,Pantydraco),(Efraasia,(Plateosauravus,(Ruehleia,(((Unaysaurus,(Pradhania,Macrocollum)),((Sarahsaurus,((Ngwevu,(Xixipiosaurus,((Coloradisaurus,(Glacialisaurus,Lufengosaurus)),(Massospondylus_carinatus,(Adeopapposaurus,Leyesaurus))))),(Yunnanosaurus_huangi,((Seitaad,(Anchisaurus,((Irisosaurus,(Yizhousaurus,((Sefapanosaurus,Aardonyx),(NMQR1551,(NMRQ3314,(Blikanasaurus,((Camelotia,((Meroktenos,(Ledumahadi,Kholumolumo)),(Lessemsaurus,(Ingentia,Antetonitrus)))),((Schleitheimia,(Isanosaurus,((Tazoudasaurus,Vulcanodon),((Shunosaurus,((Spinophorosaurus,Omeisaurus),(Mamenchisaurus,(Cetiosaurus,Neosauropoda)))),(Patagosaurus,Barapasaurus))))),(Pulanesaura,Gongxianosaurus))))))))),(Mussaurus,Leonerasaurus)))),(Xingxiulong,(Jingshanosaurus,Chuxiongosaurus)))))),(Eucnemesaurus_fortis,(Riojasaurus,Eucnemesaurus_entaxonis)))),(Plateosaurus_gracilis,(Plateosaurus_ingens,Plateosaurus_engelhardti))))))))))))))))),(Staurikosaurus,Herrerasaurus)))))));

(Euparkeria,(Crurotarsi,(Marasuchus,(Silesaurus,(Ornithischia,((Agnosphitys,((Guaibasaurus,(Neotheropoda,Chindesaurus)),(Buriolestes,(Panphagia,(Eoraptor,((Saturnalia,Chromogisaurus),(Pampadromaeus,(Bagualosaurus,(Jaklapalisaurus,(Nambalia,((Thecodontosaurus,Pantydraco),(Efraasia,(Plateosauravus,(Ruehleia,(((Unaysaurus,(Pradhania,Macrocollum)),((Sarahsaurus,((Ngwevu,(Xixipiosaurus,((Coloradisaurus,(Glacialisaurus,Lufengosaurus)),(Massospondylus_carinatus,(Adeopapposaurus,Leyesaurus))))),(Yunnanosaurus_huangi,((Seitaad,(Anchisaurus,((Irisosaurus,(Yizhousaurus,((Sefapanosaurus,Aardonyx),(NMQR1551,(NMRQ3314,(Blikanasaurus,((Camelotia,((Meroktenos,(Ledumahadi,Kholumolumo)),(Lessemsaurus,(Ingentia,Antetonitrus)))),((Schleitheimia,(Isanosaurus,((Tazoudasaurus,Vulcanodon),((Shunosaurus,(Spinophorosaurus,(Mamenchisaurus,(Omeisaurus,(Cetiosaurus,Neosauropoda))))),(Patagosaurus,Barapasaurus))))),(Pulanesaura,Gongxianosaurus))))))))),(Mussaurus,Leonerasaurus)))),(Xingxiulong,(Jingshanosaurus,Chuxiongosaurus)))))),(Eucnemesaurus_fortis,(Riojasaurus,Eucnemesaurus_entaxonis)))),(Plateosaurus_gracilis,(Plateosaurus_ingens,Plateosaurus_engelhardti))))))))))))))))),(Staurikosaurus,Herrerasaurus)))))));

(Euparkeria,(Crurotarsi,(Marasuchus,(Silesaurus,(Ornithischia,((Agnosphitys,((Guaibasaurus,(Neotheropoda,Chindesaurus)),(Buriolestes,(Panphagia,(Eoraptor,((Saturnalia,Chromogisaurus),(Pampadromaeus,(Bagualosaurus,(Jaklapalisaurus,(Nambalia,((Thecodontosaurus,Pantydraco),(Efraasia,(Plateosauravus,(Ruehleia,(((Unaysaurus,(Pradhania,Macrocollum)),((Sarahsaurus,((Ngwevu,(Xixipiosaurus,((Coloradisaurus,(Glacialisaurus,Lufengosaurus)),(Massospondylus_carinatus,(Adeopapposaurus,Leyesaurus))))),(Yunnanosaurus_huangi,((Seitaad,(Anchisaurus,((Irisosaurus,(Yizhousaurus,((Sefapanosaurus,Aardonyx),(NMQR1551,(NMRQ3314,(Blikanasaurus,((Camelotia,((Meroktenos,(Ledumahadi,Kholumolumo)),(Lessemsaurus,(Ingentia,Antetonitrus)))),((Schleitheimia,(Isanosaurus,((Tazoudasaurus,Vulcanodon),((Shunosaurus,(Spinophorosaurus,(Omeisaurus,(Mamenchisaurus,(Cetiosaurus,Neosauropoda))))),(Patagosaurus,Barapasaurus))))),(Pulanesaura,Gongxianosaurus))))))))),(Mussaurus,Leonerasaurus)))),(Xingxiulong,(Jingshanosaurus,Chuxiongosaurus)))))),(Eucnemesaurus_fortis,(Riojasaurus,Eucnemesaurus_entaxonis)))),(Plateosaurus_gracilis,(Plateosaurus_ingens,Plateosaurus_engelhardti))))))))))))))))),(Staurikosaurus,Herrerasaurus)))))));

(Euparkeria,(Crurotarsi,(Marasuchus,(Silesaurus,(Ornithischia,((Agnosphitys,((Guaibasaurus,(Neotheropoda,Chindesaurus)),(Buriolestes,(Panphagia,(Eoraptor,((Pampadromaeus,(Saturnalia,Chromogisaurus)),(Bagualosaurus,(Jaklapalisaurus,(Nambalia,((Thecodontosaurus,Pantydraco),(Efraasia,(Plateosauravus,(Ruehleia,(((Unaysaurus,(Pradhania,Macrocollum)),((Sarahsaurus,((Ngwevu,(Xixipiosaurus,((Coloradisaurus,(Glacialisaurus,Lufengosaurus)),(Massospondylus_carinatus,(Adeopapposaurus,Leyesaurus))))),(Yunnanosaurus_huangi,((Seitaad,(Anchisaurus,((Irisosaurus,(Yizhousaurus,((Sefapanosaurus,Aardonyx),(NMQR1551,(NMRQ3314,((Camelotia,((Meroktenos,(Ledumahadi,Kholumolumo)),(Lessemsaurus,(Blikanasaurus,(Ingentia,Antetonitrus))))),((Schleitheimia,(Isanosaurus,((Tazoudasaurus,Vulcanodon),((Shunosaurus,(Spinophorosaurus,(Omeisaurus,(Mamenchisaurus,(Cetiosaurus,Neosauropoda))))),(Patagosaurus,Barapasaurus))))),(Pulanesaura,Gongxianosaurus)))))))),(Mussaurus,Leonerasaurus)))),(Xingxiulong,(Jingshanosaurus,Chuxiongosaurus)))))),(Eucnemesaurus_fortis,(Riojasaurus,Eucnemesaurus_entaxonis)))),(Plateosaurus_gracilis,(Plateosaurus_ingens,Plateosaurus_engelhardti)))))))))))))))),(Staurikosaurus,Herrerasaurus)))))));

(Euparkeria,(Crurotarsi,(Marasuchus,(Silesaurus,(Ornithischia,((Agnosphitys,((Guaibasaurus,(Neotheropoda,Chindesaurus)),(Buriolestes,(Pampadromaeus,(Panphagia,(Eoraptor,((Saturnalia,Chromogisaurus),(Bagualosaurus,(Jaklapalisaurus,(Nambalia,((Thecodontosaurus,Pantydraco),(Efraasia,(Plateosauravus,(Ruehleia,(((Unaysaurus,(Pradhania,Macrocollum)),((Sarahsaurus,((Ngwevu,((Coloradisaurus,(Glacialisaurus,Lufengosaurus)),(Xixipiosaurus,(Massospondylus_carinatus,(Adeopapposaurus,Leyesaurus))))),(Yunnanosaurus_huangi,((Seitaad,(Anchisaurus,((Irisosaurus,(Sefapanosaurus,(Yizhousaurus,(Aardonyx,(NMQR1551,(NMRQ3314,(Blikanasaurus,((Camelotia,((Meroktenos,(Ledumahadi,Kholumolumo)),(Lessemsaurus,(Ingentia,Antetonitrus)))),(Pulanesaura,(Gongxianosaurus,(Schleitheimia,(Isanosaurus,((Tazoudasaurus,Vulcanodon),((Shunosaurus,((Spinophorosaurus,(Omeisaurus,Mamenchisaurus)),(Cetiosaurus,Neosauropoda))),(Patagosaurus,Barapasaurus))))))))))))))),(Mussaurus,Leonerasaurus)))),(Xingxiulong,(Jingshanosaurus,Chuxiongosaurus)))))),(Eucnemesaurus_fortis,(Riojasaurus,Eucnemesaurus_entaxonis)))),(Plateosaurus_gracilis,(Plateosaurus_ingens,Plateosaurus_engelhardti))))))))))))))))),(Staurikosaurus,Herrerasaurus)))))));

(Euparkeria,(Crurotarsi,(Marasuchus,(Silesaurus,(Ornithischia,((Agnosphitys,((Guaibasaurus,(Neotheropoda,Chindesaurus)),(Buriolestes,(Pampadromaeus,(Panphagia,(Eoraptor,((Saturnalia,Chromogisaurus),(Bagualosaurus,(Jaklapalisaurus,(Nambalia,((Thecodontosaurus,Pantydraco),(Efraasia,(Plateosauravus,(Ruehleia,(((Unaysaurus,(Pradhania,Macrocollum)),((Sarahsaurus,((Ngwevu,(Xixipiosaurus,((Coloradisaurus,(Glacialisaurus,Lufengosaurus)),(Massospondylus_carinatus,(Adeopapposaurus,Leyesaurus))))),(Yunnanosaurus_huangi,((Seitaad,(Anchisaurus,((Irisosaurus,(Sefapanosaurus,(Yizhousaurus,(Aardonyx,(NMQR1551,(NMRQ3314,(Blikanasaurus,((Camelotia,((Meroktenos,(Ledumahadi,Kholumolumo)),(Lessemsaurus,(Ingentia,Antetonitrus)))),(Pulanesaura,(Gongxianosaurus,(Schleitheimia,(Isanosaurus,((Tazoudasaurus,Vulcanodon),((Shunosaurus,((Spinophorosaurus,Omeisaurus),(Mamenchisaurus,(Cetiosaurus,Neosauropoda)))),(Patagosaurus,Barapasaurus))))))))))))))),(Mussaurus,Leonerasaurus)))),(Xingxiulong,(Jingshanosaurus,Chuxiongosaurus)))))),(Eucnemesaurus_fortis,(Riojasaurus,Eucnemesaurus_entaxonis)))),(Plateosaurus_gracilis,(Plateosaurus_ingens,Plateosaurus_engelhardti))))))))))))))))),(Staurikosaurus,Herrerasaurus)))))));

(Euparkeria,(Crurotarsi,(Marasuchus,(Silesaurus,(Ornithischia,((Agnosphitys,((Guaibasaurus,(Neotheropoda,Chindesaurus)),((Eoraptor,((Saturnalia,Chromogisaurus),(Bagualosaurus,(Jaklapalisaurus,(Nambalia,((Thecodontosaurus,Pantydraco),(Efraasia,(Plateosauravus,(Ruehleia,(((Unaysaurus,(Pradhania,Macrocollum)),((Sarahsaurus,((Ngwevu,(Xixipiosaurus,((Coloradisaurus,(Glacialisaurus,Lufengosaurus)),(Massospondylus_carinatus,(Adeopapposaurus,Leyesaurus))))),(Yunnanosaurus_huangi,((Seitaad,(Anchisaurus,((Irisosaurus,(Sefapanosaurus,(Yizhousaurus,(Aardonyx,(NMQR1551,(NMRQ3314,(Blikanasaurus,((Camelotia,((Meroktenos,(Ledumahadi,Kholumolumo)),(Lessemsaurus,(Ingentia,Antetonitrus)))),(Pulanesaura,(Gongxianosaurus,(Schleitheimia,(Isanosaurus,((Tazoudasaurus,Vulcanodon),((Shunosaurus,((Spinophorosaurus,(Omeisaurus,Mamenchisaurus)),(Cetiosaurus,Neosauropoda))),(Patagosaurus,Barapasaurus))))))))))))))),(Mussaurus,Leonerasaurus)))),(Xingxiulong,(Jingshanosaurus,Chuxiongosaurus)))))),(Eucnemesaurus_fortis,(Riojasaurus,Eucnemesaurus_entaxonis)))),(Plateosaurus_gracilis,(Plateosaurus_ingens,Plateosaurus_engelhardti)))))))))))),(Buriolestes,(Panphagia,Pampadromaeus))))),(Staurikosaurus,Herrerasaurus)))))));

(Euparkeria,(Crurotarsi,(Marasuchus,(Silesaurus,(Ornithischia,((Agnosphitys,((Guaibasaurus,(Neotheropoda,Chindesaurus)),(Buriolestes,(Pampadromaeus,(Panphagia,(Eoraptor,((Saturnalia,Chromogisaurus),(Bagualosaurus,(Jaklapalisaurus,(Nambalia,((Thecodontosaurus,Pantydraco),(Efraasia,(Plateosauravus,(Ruehleia,(((Unaysaurus,(Pradhania,Macrocollum)),((Sarahsaurus,((Ngwevu,(Xixipiosaurus,((Coloradisaurus,(Glacialisaurus,Lufengosaurus)),(Massospondylus_carinatus,(Adeopapposaurus,Leyesaurus))))),(Yunnanosaurus_huangi,((Seitaad,(Anchisaurus,((Irisosaurus,(Sefapanosaurus,(Yizhousaurus,(Aardonyx,(NMQR1551,(NMRQ3314,(Blikanasaurus,((Camelotia,((Meroktenos,(Ledumahadi,Kholumolumo)),(Lessemsaurus,(Ingentia,Antetonitrus)))),(Pulanesaura,(Gongxianosaurus,(Schleitheimia,(Isanosaurus,((Tazoudasaurus,Vulcanodon),((Shunosaurus,(Spinophorosaurus,(Mamenchisaurus,(Omeisaurus,(Cetiosaurus,Neosauropoda))))),(Patagosaurus,Barapasaurus))))))))))))))),(Mussaurus,Leonerasaurus)))),(Xingxiulong,(Jingshanosaurus,Chuxiongosaurus)))))),(Eucnemesaurus_fortis,(Riojasaurus,Eucnemesaurus_entaxonis)))),(Plateosaurus_gracilis,(Plateosaurus_ingens,Plateosaurus_engelhardti))))))))))))))))),(Staurikosaurus,Herrerasaurus)))))));

(Euparkeria,(Crurotarsi,(Marasuchus,(Silesaurus,(Ornithischia,((Agnosphitys,((Guaibasaurus,(Neotheropoda,Chindesaurus)),(Buriolestes,(Pampadromaeus,(Panphagia,(Eoraptor,((Saturnalia,Chromogisaurus),(Bagualosaurus,(Jaklapalisaurus,(Nambalia,((Thecodontosaurus,Pantydraco),(Efraasia,(Plateosauravus,(Ruehleia,(((Unaysaurus,(Pradhania,Macrocollum)),((Sarahsaurus,((Ngwevu,(Xixipiosaurus,((Coloradisaurus,(Glacialisaurus,Lufengosaurus)),(Massospondylus_carinatus,(Adeopapposaurus,Leyesaurus))))),(Yunnanosaurus_huangi,((Seitaad,(Anchisaurus,((Irisosaurus,(Sefapanosaurus,(Yizhousaurus,(Aardonyx,(NMQR1551,(NMRQ3314,(Blikanasaurus,((Camelotia,((Meroktenos,(Ledumahadi,Kholumolumo)),(Lessemsaurus,(Ingentia,Antetonitrus)))),(Pulanesaura,(Gongxianosaurus,(Schleitheimia,(Isanosaurus,((Tazoudasaurus,Vulcanodon),((Shunosaurus,(Spinophorosaurus,(Omeisaurus,(Mamenchisaurus,(Cetiosaurus,Neosauropoda))))),(Patagosaurus,Barapasaurus))))))))))))))),(Mussaurus,Leonerasaurus)))),(Xingxiulong,(Jingshanosaurus,Chuxiongosaurus)))))),(Eucnemesaurus_fortis,(Riojasaurus,Eucnemesaurus_entaxonis)))),(Plateosaurus_gracilis,(Plateosaurus_ingens,Plateosaurus_engelhardti))))))))))))))))),(Staurikosaurus,Herrerasaurus)))))));

(Euparkeria,(Crurotarsi,(Marasuchus,(Silesaurus,(Ornithischia,((Agnosphitys,((Guaibasaurus,(Neotheropoda,Chindesaurus)),(Buriolestes,((Eoraptor,((Saturnalia,Chromogisaurus),(Bagualosaurus,(Jaklapalisaurus,(Nambalia,((Thecodontosaurus,Pantydraco),(Efraasia,(Plateosauravus,(Ruehleia,(((Unaysaurus,(Pradhania,Macrocollum)),((Sarahsaurus,((Ngwevu,((Coloradisaurus,(Glacialisaurus,Lufengosaurus)),(Xixipiosaurus,(Massospondylus_carinatus,(Adeopapposaurus,Leyesaurus))))),(Yunnanosaurus_huangi,((Seitaad,(Anchisaurus,((Irisosaurus,(Sefapanosaurus,(Yizhousaurus,(Aardonyx,(NMQR1551,(NMRQ3314,(Blikanasaurus,((Camelotia,((Meroktenos,(Ledumahadi,Kholumolumo)),(Lessemsaurus,(Ingentia,Antetonitrus)))),(Pulanesaura,(Gongxianosaurus,(Schleitheimia,(Isanosaurus,((Tazoudasaurus,Vulcanodon),((Shunosaurus,((Spinophorosaurus,(Omeisaurus,Mamenchisaurus)),(Cetiosaurus,Neosauropoda))),(Patagosaurus,Barapasaurus))))))))))))))),(Mussaurus,Leonerasaurus)))),(Xingxiulong,(Jingshanosaurus,Chuxiongosaurus)))))),(Eucnemesaurus_fortis,(Riojasaurus,Eucnemesaurus_entaxonis)))),(Plateosaurus_gracilis,(Plateosaurus_ingens,Plateosaurus_engelhardti)))))))))))),(Panphagia,Pampadromaeus))))),(Staurikosaurus,Herrerasaurus)))))));

(Euparkeria,(Crurotarsi,(Marasuchus,(Silesaurus,(Ornithischia,((Agnosphitys,((Guaibasaurus,(Neotheropoda,Chindesaurus)),(Buriolestes,((Eoraptor,((Saturnalia,Chromogisaurus),(Bagualosaurus,(Jaklapalisaurus,(Nambalia,((Thecodontosaurus,Pantydraco),(Efraasia,(Plateosauravus,(Ruehleia,(((Unaysaurus,(Pradhania,Macrocollum)),((Sarahsaurus,((Ngwevu,(Xixipiosaurus,((Coloradisaurus,(Glacialisaurus,Lufengosaurus)),(Massospondylus_carinatus,(Adeopapposaurus,Leyesaurus))))),(Yunnanosaurus_huangi,((Seitaad,(Anchisaurus,((Irisosaurus,(Sefapanosaurus,(Yizhousaurus,(Aardonyx,(NMQR1551,(NMRQ3314,(Blikanasaurus,((Camelotia,((Meroktenos,(Ledumahadi,Kholumolumo)),(Lessemsaurus,(Ingentia,Antetonitrus)))),(Pulanesaura,(Gongxianosaurus,(Schleitheimia,(Isanosaurus,((Tazoudasaurus,Vulcanodon),((Shunosaurus,((Spinophorosaurus,Omeisaurus),(Mamenchisaurus,(Cetiosaurus,Neosauropoda)))),(Patagosaurus,Barapasaurus))))))))))))))),(Mussaurus,Leonerasaurus)))),(Xingxiulong,(Jingshanosaurus,Chuxiongosaurus)))))),(Eucnemesaurus_fortis,(Riojasaurus,Eucnemesaurus_entaxonis)))),(Plateosaurus_gracilis,(Plateosaurus_ingens,Plateosaurus_engelhardti)))))))))))),(Panphagia,Pampadromaeus))))),(Staurikosaurus,Herrerasaurus)))))));

(Euparkeria,(Crurotarsi,(Marasuchus,(Silesaurus,(Ornithischia,((Agnosphitys,((Guaibasaurus,(Neotheropoda,Chindesaurus)),(Buriolestes,((Eoraptor,((Saturnalia,Chromogisaurus),(Bagualosaurus,(Jaklapalisaurus,(Nambalia,((Thecodontosaurus,Pantydraco),(Efraasia,(Plateosauravus,(Ruehleia,(((Unaysaurus,(Pradhania,Macrocollum)),((Sarahsaurus,((Ngwevu,(Xixipiosaurus,((Coloradisaurus,(Glacialisaurus,Lufengosaurus)),(Massospondylus_carinatus,(Adeopapposaurus,Leyesaurus))))),(Yunnanosaurus_huangi,((Seitaad,(Anchisaurus,((Irisosaurus,(Sefapanosaurus,(Yizhousaurus,(Aardonyx,(NMQR1551,(NMRQ3314,(Blikanasaurus,((Camelotia,((Meroktenos,(Ledumahadi,Kholumolumo)),(Lessemsaurus,(Ingentia,Antetonitrus)))),(Pulanesaura,(Gongxianosaurus,(Schleitheimia,(Isanosaurus,((Tazoudasaurus,Vulcanodon),((Shunosaurus,(Spinophorosaurus,(Mamenchisaurus,(Omeisaurus,(Cetiosaurus,Neosauropoda))))),(Patagosaurus,Barapasaurus))))))))))))))),(Mussaurus,Leonerasaurus)))),(Xingxiulong,(Jingshanosaurus,Chuxiongosaurus)))))),(Eucnemesaurus_fortis,(Riojasaurus,Eucnemesaurus_entaxonis)))),(Plateosaurus_gracilis,(Plateosaurus_ingens,Plateosaurus_engelhardti)))))))))))),(Panphagia,Pampadromaeus))))),(Staurikosaurus,Herrerasaurus)))))));

(Euparkeria,(Crurotarsi,(Marasuchus,(Silesaurus,(Ornithischia,((Agnosphitys,((Guaibasaurus,(Neotheropoda,Chindesaurus)),(Buriolestes,((Eoraptor,((Saturnalia,Chromogisaurus),(Bagualosaurus,(Jaklapalisaurus,(Nambalia,((Thecodontosaurus,Pantydraco),(Efraasia,(Plateosauravus,(Ruehleia,(((Unaysaurus,(Pradhania,Macrocollum)),((Sarahsaurus,((Ngwevu,(Xixipiosaurus,((Coloradisaurus,(Glacialisaurus,Lufengosaurus)),(Massospondylus_carinatus,(Adeopapposaurus,Leyesaurus))))),(Yunnanosaurus_huangi,((Seitaad,(Anchisaurus,((Irisosaurus,(Sefapanosaurus,(Yizhousaurus,(Aardonyx,(NMQR1551,(NMRQ3314,(Blikanasaurus,((Camelotia,((Meroktenos,(Ledumahadi,Kholumolumo)),(Lessemsaurus,(Ingentia,Antetonitrus)))),(Pulanesaura,(Gongxianosaurus,(Schleitheimia,(Isanosaurus,((Tazoudasaurus,Vulcanodon),((Shunosaurus,(Spinophorosaurus,(Omeisaurus,(Mamenchisaurus,(Cetiosaurus,Neosauropoda))))),(Patagosaurus,Barapasaurus))))))))))))))),(Mussaurus,Leonerasaurus)))),(Xingxiulong,(Jingshanosaurus,Chuxiongosaurus)))))),(Eucnemesaurus_fortis,(Riojasaurus,Eucnemesaurus_entaxonis)))),(Plateosaurus_gracilis,(Plateosaurus_ingens,Plateosaurus_engelhardti)))))))))))),(Panphagia,Pampadromaeus))))),(Staurikosaurus,Herrerasaurus)))))));

(Euparkeria,(Crurotarsi,(Marasuchus,(Silesaurus,(Ornithischia,((Agnosphitys,((Guaibasaurus,(Neotheropoda,Chindesaurus)),(Buriolestes,(Panphagia,(Eoraptor,((Pampadromaeus,(Saturnalia,Chromogisaurus)),(Bagualosaurus,(Jaklapalisaurus,(Nambalia,((Thecodontosaurus,Pantydraco),(Efraasia,(Plateosauravus,(Ruehleia,(((Unaysaurus,(Pradhania,Macrocollum)),((Sarahsaurus,((Ngwevu,((Coloradisaurus,(Glacialisaurus,Lufengosaurus)),(Xixipiosaurus,(Massospondylus_carinatus,(Adeopapposaurus,Leyesaurus))))),(Yunnanosaurus_huangi,((Seitaad,(Anchisaurus,((Irisosaurus,(Sefapanosaurus,(Yizhousaurus,(Aardonyx,(NMQR1551,(NMRQ3314,(Blikanasaurus,((Camelotia,((Meroktenos,(Ledumahadi,Kholumolumo)),(Lessemsaurus,(Ingentia,Antetonitrus)))),(Pulanesaura,(Gongxianosaurus,(Schleitheimia,(Isanosaurus,((Tazoudasaurus,Vulcanodon),((Shunosaurus,((Spinophorosaurus,Omeisaurus),(Mamenchisaurus,(Cetiosaurus,Neosauropoda)))),(Patagosaurus,Barapasaurus))))))))))))))),(Mussaurus,Leonerasaurus)))),(Xingxiulong,(Jingshanosaurus,Chuxiongosaurus)))))),(Eucnemesaurus_fortis,(Riojasaurus,Eucnemesaurus_entaxonis)))),(Plateosaurus_gracilis,(Plateosaurus_ingens,Plateosaurus_engelhardti)))))))))))))))),(Staurikosaurus,Herrerasaurus)))))));

(Euparkeria,(Crurotarsi,(Marasuchus,(Silesaurus,(Ornithischia,((Agnosphitys,((Guaibasaurus,(Neotheropoda,Chindesaurus)),(Buriolestes,(Panphagia,(Eoraptor,((Saturnalia,Chromogisaurus),(Pampadromaeus,(Bagualosaurus,(Jaklapalisaurus,(Nambalia,((Thecodontosaurus,Pantydraco),(Efraasia,(Plateosauravus,(Ruehleia,(((Unaysaurus,(Pradhania,Macrocollum)),((Sarahsaurus,((Ngwevu,((Coloradisaurus,(Glacialisaurus,Lufengosaurus)),(Xixipiosaurus,(Massospondylus_carinatus,(Adeopapposaurus,Leyesaurus))))),(Yunnanosaurus_huangi,((Seitaad,(Anchisaurus,((Irisosaurus,(Sefapanosaurus,(Yizhousaurus,(Aardonyx,(NMQR1551,(NMRQ3314,(Blikanasaurus,((Camelotia,((Meroktenos,(Ledumahadi,Kholumolumo)),(Lessemsaurus,(Ingentia,Antetonitrus)))),(Pulanesaura,(Gongxianosaurus,(Schleitheimia,(Isanosaurus,((Tazoudasaurus,Vulcanodon),((Shunosaurus,((Spinophorosaurus,(Omeisaurus,Mamenchisaurus)),(Cetiosaurus,Neosauropoda))),(Patagosaurus,Barapasaurus))))))))))))))),(Mussaurus,Leonerasaurus)))),(Xingxiulong,(Jingshanosaurus,Chuxiongosaurus)))))),(Eucnemesaurus_fortis,(Riojasaurus,Eucnemesaurus_entaxonis)))),(Plateosaurus_gracilis,(Plateosaurus_ingens,Plateosaurus_engelhardti))))))))))))))))),(Staurikosaurus,Herrerasaurus)))))));

(Euparkeria,(Crurotarsi,(Marasuchus,(Silesaurus,(Ornithischia,((Agnosphitys,((Guaibasaurus,(Neotheropoda,Chindesaurus)),(Buriolestes,(Panphagia,(Eoraptor,((Pampadromaeus,(Saturnalia,Chromogisaurus)),(Bagualosaurus,(Jaklapalisaurus,(Nambalia,((Thecodontosaurus,Pantydraco),(Efraasia,(Plateosauravus,(Ruehleia,(((Unaysaurus,(Pradhania,Macrocollum)),((Sarahsaurus,((Ngwevu,((Coloradisaurus,(Glacialisaurus,Lufengosaurus)),(Xixipiosaurus,(Massospondylus_carinatus,(Adeopapposaurus,Leyesaurus))))),(Yunnanosaurus_huangi,((Seitaad,(Anchisaurus,((Irisosaurus,(Sefapanosaurus,(Yizhousaurus,(Aardonyx,(NMQR1551,(NMRQ3314,(Blikanasaurus,((Camelotia,((Meroktenos,(Ledumahadi,Kholumolumo)),(Lessemsaurus,(Ingentia,Antetonitrus)))),(Pulanesaura,(Gongxianosaurus,(Schleitheimia,(Isanosaurus,((Tazoudasaurus,Vulcanodon),((Shunosaurus,(Spinophorosaurus,(Mamenchisaurus,(Omeisaurus,(Cetiosaurus,Neosauropoda))))),(Patagosaurus,Barapasaurus))))))))))))))),(Mussaurus,Leonerasaurus)))),(Xingxiulong,(Jingshanosaurus,Chuxiongosaurus)))))),(Eucnemesaurus_fortis,(Riojasaurus,Eucnemesaurus_entaxonis)))),(Plateosaurus_gracilis,(Plateosaurus_ingens,Plateosaurus_engelhardti)))))))))))))))),(Staurikosaurus,Herrerasaurus)))))));

(Euparkeria,(Crurotarsi,(Marasuchus,(Silesaurus,(Ornithischia,((Agnosphitys,((Guaibasaurus,(Neotheropoda,Chindesaurus)),(Buriolestes,(Panphagia,(Eoraptor,((Pampadromaeus,(Saturnalia,Chromogisaurus)),(Bagualosaurus,(Jaklapalisaurus,(Nambalia,((Thecodontosaurus,Pantydraco),(Efraasia,(Plateosauravus,(Ruehleia,(((Unaysaurus,(Pradhania,Macrocollum)),((Sarahsaurus,((Ngwevu,((Coloradisaurus,(Glacialisaurus,Lufengosaurus)),(Xixipiosaurus,(Massospondylus_carinatus,(Adeopapposaurus,Leyesaurus))))),(Yunnanosaurus_huangi,((Seitaad,(Anchisaurus,((Irisosaurus,(Sefapanosaurus,(Yizhousaurus,(Aardonyx,(NMQR1551,(NMRQ3314,(Blikanasaurus,((Camelotia,((Meroktenos,(Ledumahadi,Kholumolumo)),(Lessemsaurus,(Ingentia,Antetonitrus)))),(Pulanesaura,(Gongxianosaurus,(Schleitheimia,(Isanosaurus,((Tazoudasaurus,Vulcanodon),((Shunosaurus,(Spinophorosaurus,(Omeisaurus,(Mamenchisaurus,(Cetiosaurus,Neosauropoda))))),(Patagosaurus,Barapasaurus))))))))))))))),(Mussaurus,Leonerasaurus)))),(Xingxiulong,(Jingshanosaurus,Chuxiongosaurus)))))),(Eucnemesaurus_fortis,(Riojasaurus,Eucnemesaurus_entaxonis)))),(Plateosaurus_gracilis,(Plateosaurus_ingens,Plateosaurus_engelhardti)))))))))))))))),(Staurikosaurus,Herrerasaurus)))))));

(Euparkeria,(Crurotarsi,(Marasuchus,(Silesaurus,(Ornithischia,((Agnosphitys,((Guaibasaurus,(Neotheropoda,Chindesaurus)),(Buriolestes,(Panphagia,(Eoraptor,((Saturnalia,Chromogisaurus),(Pampadromaeus,(Bagualosaurus,(Jaklapalisaurus,(Nambalia,((Thecodontosaurus,Pantydraco),(Efraasia,(Plateosauravus,(Ruehleia,(((Unaysaurus,(Pradhania,Macrocollum)),((Sarahsaurus,((Ngwevu,(Xixipiosaurus,((Coloradisaurus,(Glacialisaurus,Lufengosaurus)),(Massospondylus_carinatus,(Adeopapposaurus,Leyesaurus))))),(Yunnanosaurus_huangi,((Seitaad,(Anchisaurus,((Irisosaurus,(Sefapanosaurus,(Yizhousaurus,(Aardonyx,(NMQR1551,(NMRQ3314,(Blikanasaurus,((Camelotia,((Meroktenos,(Ledumahadi,Kholumolumo)),(Lessemsaurus,(Ingentia,Antetonitrus)))),(Pulanesaura,(Gongxianosaurus,(Schleitheimia,(Isanosaurus,((Tazoudasaurus,Vulcanodon),((Shunosaurus,((Spinophorosaurus,Omeisaurus),(Mamenchisaurus,(Cetiosaurus,Neosauropoda)))),(Patagosaurus,Barapasaurus))))))))))))))),(Mussaurus,Leonerasaurus)))),(Xingxiulong,(Jingshanosaurus,Chuxiongosaurus)))))),(Eucnemesaurus_fortis,(Riojasaurus,Eucnemesaurus_entaxonis)))),(Plateosaurus_gracilis,(Plateosaurus_ingens,Plateosaurus_engelhardti))))))))))))))))),(Staurikosaurus,Herrerasaurus)))))));

(Euparkeria,(Crurotarsi,(Marasuchus,(Silesaurus,(Ornithischia,((Agnosphitys,((Guaibasaurus,(Neotheropoda,Chindesaurus)),(Buriolestes,(Panphagia,(Eoraptor,((Saturnalia,Chromogisaurus),(Pampadromaeus,(Bagualosaurus,(Jaklapalisaurus,(Nambalia,((Thecodontosaurus,Pantydraco),(Efraasia,(Plateosauravus,(Ruehleia,(((Unaysaurus,(Pradhania,Macrocollum)),((Sarahsaurus,((Ngwevu,(Xixipiosaurus,((Coloradisaurus,(Glacialisaurus,Lufengosaurus)),(Massospondylus_carinatus,(Adeopapposaurus,Leyesaurus))))),(Yunnanosaurus_huangi,((Seitaad,(Anchisaurus,((Irisosaurus,(Sefapanosaurus,(Yizhousaurus,(Aardonyx,(NMQR1551,(NMRQ3314,(Blikanasaurus,((Camelotia,((Meroktenos,(Ledumahadi,Kholumolumo)),(Lessemsaurus,(Ingentia,Antetonitrus)))),(Pulanesaura,(Gongxianosaurus,(Schleitheimia,(Isanosaurus,((Tazoudasaurus,Vulcanodon),((Shunosaurus,(Spinophorosaurus,(Mamenchisaurus,(Omeisaurus,(Cetiosaurus,Neosauropoda))))),(Patagosaurus,Barapasaurus))))))))))))))),(Mussaurus,Leonerasaurus)))),(Xingxiulong,(Jingshanosaurus,Chuxiongosaurus)))))),(Eucnemesaurus_fortis,(Riojasaurus,Eucnemesaurus_entaxonis)))),(Plateosaurus_gracilis,(Plateosaurus_ingens,Plateosaurus_engelhardti))))))))))))))))),(Staurikosaurus,Herrerasaurus)))))));

(Euparkeria,(Crurotarsi,(Marasuchus,(Silesaurus,(Ornithischia,((Agnosphitys,((Guaibasaurus,(Neotheropoda,Chindesaurus)),(Buriolestes,(Panphagia,(Eoraptor,((Saturnalia,Chromogisaurus),(Pampadromaeus,(Bagualosaurus,(Jaklapalisaurus,(Nambalia,((Thecodontosaurus,Pantydraco),(Efraasia,(Plateosauravus,(Ruehleia,(((Unaysaurus,(Pradhania,Macrocollum)),((Sarahsaurus,((Ngwevu,(Xixipiosaurus,((Coloradisaurus,(Glacialisaurus,Lufengosaurus)),(Massospondylus_carinatus,(Adeopapposaurus,Leyesaurus))))),(Yunnanosaurus_huangi,((Seitaad,(Anchisaurus,((Irisosaurus,(Sefapanosaurus,(Yizhousaurus,(Aardonyx,(NMQR1551,(NMRQ3314,(Blikanasaurus,((Camelotia,((Meroktenos,(Ledumahadi,Kholumolumo)),(Lessemsaurus,(Ingentia,Antetonitrus)))),(Pulanesaura,(Gongxianosaurus,(Schleitheimia,(Isanosaurus,((Tazoudasaurus,Vulcanodon),((Shunosaurus,(Spinophorosaurus,(Omeisaurus,(Mamenchisaurus,(Cetiosaurus,Neosauropoda))))),(Patagosaurus,Barapasaurus))))))))))))))),(Mussaurus,Leonerasaurus)))),(Xingxiulong,(Jingshanosaurus,Chuxiongosaurus)))))),(Eucnemesaurus_fortis,(Riojasaurus,Eucnemesaurus_entaxonis)))),(Plateosaurus_gracilis,(Plateosaurus_ingens,Plateosaurus_engelhardti))))))))))))))))),(Staurikosaurus,Herrerasaurus)))))));

(Euparkeria,(Crurotarsi,(Marasuchus,(Silesaurus,(Ornithischia,((Agnosphitys,((Guaibasaurus,(Neotheropoda,Chindesaurus)),(Buriolestes,(Panphagia,(Eoraptor,((Pampadromaeus,(Saturnalia,Chromogisaurus)),(Bagualosaurus,(Jaklapalisaurus,(Nambalia,((Thecodontosaurus,Pantydraco),(Efraasia,(Plateosauravus,(Ruehleia,(((Unaysaurus,(Pradhania,Macrocollum)),((Sarahsaurus,((Ngwevu,(Xixipiosaurus,((Coloradisaurus,(Glacialisaurus,Lufengosaurus)),(Massospondylus_carinatus,(Adeopapposaurus,Leyesaurus))))),(Yunnanosaurus_huangi,((Seitaad,(Anchisaurus,((Irisosaurus,(Sefapanosaurus,(Yizhousaurus,(Aardonyx,(NMQR1551,(NMRQ3314,((Camelotia,((Meroktenos,(Ledumahadi,Kholumolumo)),(Lessemsaurus,(Blikanasaurus,(Ingentia,Antetonitrus))))),(Pulanesaura,(Gongxianosaurus,(Schleitheimia,(Isanosaurus,((Tazoudasaurus,Vulcanodon),((Shunosaurus,(Spinophorosaurus,(Omeisaurus,(Mamenchisaurus,(Cetiosaurus,Neosauropoda))))),(Patagosaurus,Barapasaurus)))))))))))))),(Mussaurus,Leonerasaurus)))),(Xingxiulong,(Jingshanosaurus,Chuxiongosaurus)))))),(Eucnemesaurus_fortis,(Riojasaurus,Eucnemesaurus_entaxonis)))),(Plateosaurus_gracilis,(Plateosaurus_ingens,Plateosaurus_engelhardti)))))))))))))))),(Staurikosaurus,Herrerasaurus)))))));

(Euparkeria,(Crurotarsi,(Marasuchus,(Silesaurus,(Agnosphitys,(Ornithischia,(((Guaibasaurus,(Neotheropoda,Chindesaurus)),(Buriolestes,(Pampadromaeus,(Panphagia,(Eoraptor,((Saturnalia,Chromogisaurus),(Bagualosaurus,(Jaklapalisaurus,(Nambalia,((Thecodontosaurus,Pantydraco),(Efraasia,(Plateosauravus,(Ruehleia,(((Unaysaurus,(Pradhania,Macrocollum)),((Sarahsaurus,((Ngwevu,(Xixipiosaurus,((Coloradisaurus,(Glacialisaurus,Lufengosaurus)),(Massospondylus_carinatus,(Adeopapposaurus,Leyesaurus))))),(Yunnanosaurus_huangi,((Seitaad,(Anchisaurus,((Irisosaurus,(Sefapanosaurus,(Yizhousaurus,(Aardonyx,(NMQR1551,(NMRQ3314,(Blikanasaurus,((Camelotia,((Meroktenos,(Ledumahadi,Kholumolumo)),(Lessemsaurus,(Ingentia,Antetonitrus)))),((Schleitheimia,(Isanosaurus,((Tazoudasaurus,Vulcanodon),((Shunosaurus,((Spinophorosaurus,Omeisaurus),(Mamenchisaurus,(Cetiosaurus,Neosauropoda)))),(Patagosaurus,Barapasaurus))))),(Pulanesaura,Gongxianosaurus)))))))))),(Mussaurus,Leonerasaurus)))),(Xingxiulong,(Jingshanosaurus,Chuxiongosaurus)))))),(Eucnemesaurus_fortis,(Riojasaurus,Eucnemesaurus_entaxonis)))),(Plateosaurus_gracilis,(Plateosaurus_ingens,Plateosaurus_engelhardti)))))))))))))))),(Staurikosaurus,Herrerasaurus))))))));

(Euparkeria,(Crurotarsi,(Marasuchus,(Silesaurus,(Ornithischia,(Agnosphitys,(((Guaibasaurus,(Neotheropoda,Chindesaurus)),(Buriolestes,(Pampadromaeus,(Panphagia,(Eoraptor,((Saturnalia,Chromogisaurus),(Bagualosaurus,(Jaklapalisaurus,(Nambalia,((Thecodontosaurus,Pantydraco),(Efraasia,(Plateosauravus,(Ruehleia,(((Unaysaurus,(Pradhania,Macrocollum)),((Sarahsaurus,((Ngwevu,(Xixipiosaurus,((Coloradisaurus,(Glacialisaurus,Lufengosaurus)),(Massospondylus_carinatus,(Adeopapposaurus,Leyesaurus))))),(Yunnanosaurus_huangi,((Seitaad,(Anchisaurus,((Irisosaurus,(Sefapanosaurus,(Yizhousaurus,(Aardonyx,(NMQR1551,(NMRQ3314,(Blikanasaurus,((Camelotia,((Meroktenos,(Ledumahadi,Kholumolumo)),(Lessemsaurus,(Ingentia,Antetonitrus)))),((Schleitheimia,(Isanosaurus,((Tazoudasaurus,Vulcanodon),((Shunosaurus,((Spinophorosaurus,Omeisaurus),(Mamenchisaurus,(Cetiosaurus,Neosauropoda)))),(Patagosaurus,Barapasaurus))))),(Pulanesaura,Gongxianosaurus)))))))))),(Mussaurus,Leonerasaurus)))),(Xingxiulong,(Jingshanosaurus,Chuxiongosaurus)))))),(Eucnemesaurus_fortis,(Riojasaurus,Eucnemesaurus_entaxonis)))),(Plateosaurus_gracilis,(Plateosaurus_ingens,Plateosaurus_engelhardti)))))))))))))))),(Staurikosaurus,Herrerasaurus))))))));

(Euparkeria,(Crurotarsi,(Marasuchus,(Silesaurus,(Ornithischia,((Agnosphitys,((Guaibasaurus,(Neotheropoda,Chindesaurus)),(Buriolestes,(Pampadromaeus,(Panphagia,(Eoraptor,((Saturnalia,Chromogisaurus),(Bagualosaurus,(Jaklapalisaurus,(Nambalia,((Thecodontosaurus,Pantydraco),(Efraasia,(Plateosauravus,(Ruehleia,(((Unaysaurus,(Pradhania,Macrocollum)),((Sarahsaurus,((Ngwevu,((Coloradisaurus,(Glacialisaurus,Lufengosaurus)),(Xixipiosaurus,(Massospondylus_carinatus,(Adeopapposaurus,Leyesaurus))))),(Yunnanosaurus_huangi,((Seitaad,(Anchisaurus,((Irisosaurus,(Sefapanosaurus,(Yizhousaurus,(Aardonyx,(NMQR1551,(NMRQ3314,(Blikanasaurus,((Camelotia,((Meroktenos,(Ledumahadi,Kholumolumo)),(Lessemsaurus,(Ingentia,Antetonitrus)))),((Schleitheimia,(Isanosaurus,((Tazoudasaurus,Vulcanodon),((Shunosaurus,((Spinophorosaurus,Omeisaurus),(Mamenchisaurus,(Cetiosaurus,Neosauropoda)))),(Patagosaurus,Barapasaurus))))),(Pulanesaura,Gongxianosaurus)))))))))),(Mussaurus,Leonerasaurus)))),(Xingxiulong,(Jingshanosaurus,Chuxiongosaurus)))))),(Eucnemesaurus_fortis,(Riojasaurus,Eucnemesaurus_entaxonis)))),(Plateosaurus_gracilis,(Plateosaurus_ingens,Plateosaurus_engelhardti))))))))))))))))),(Staurikosaurus,Herrerasaurus)))))));

(Euparkeria,(Crurotarsi,(Marasuchus,(Silesaurus,(Ornithischia,((Agnosphitys,((Guaibasaurus,(Neotheropoda,Chindesaurus)),((Eoraptor,((Saturnalia,Chromogisaurus),(Bagualosaurus,(Jaklapalisaurus,(Nambalia,((Thecodontosaurus,Pantydraco),(Efraasia,(Plateosauravus,(Ruehleia,(((Unaysaurus,(Pradhania,Macrocollum)),((Sarahsaurus,((Ngwevu,(Xixipiosaurus,((Coloradisaurus,(Glacialisaurus,Lufengosaurus)),(Massospondylus_carinatus,(Adeopapposaurus,Leyesaurus))))),(Yunnanosaurus_huangi,((Seitaad,(Anchisaurus,((Irisosaurus,(Sefapanosaurus,(Yizhousaurus,(Aardonyx,(NMQR1551,(NMRQ3314,(Blikanasaurus,((Camelotia,((Meroktenos,(Ledumahadi,Kholumolumo)),(Lessemsaurus,(Ingentia,Antetonitrus)))),((Schleitheimia,(Isanosaurus,((Tazoudasaurus,Vulcanodon),((Shunosaurus,((Spinophorosaurus,Omeisaurus),(Mamenchisaurus,(Cetiosaurus,Neosauropoda)))),(Patagosaurus,Barapasaurus))))),(Pulanesaura,Gongxianosaurus)))))))))),(Mussaurus,Leonerasaurus)))),(Xingxiulong,(Jingshanosaurus,Chuxiongosaurus)))))),(Eucnemesaurus_fortis,(Riojasaurus,Eucnemesaurus_entaxonis)))),(Plateosaurus_gracilis,(Plateosaurus_ingens,Plateosaurus_engelhardti)))))))))))),(Buriolestes,(Panphagia,Pampadromaeus))))),(Staurikosaurus,Herrerasaurus)))))));

(Euparkeria,(Crurotarsi,(Marasuchus,(Silesaurus,(Ornithischia,((Agnosphitys,((Guaibasaurus,(Neotheropoda,Chindesaurus)),(Buriolestes,((Eoraptor,((Saturnalia,Chromogisaurus),(Bagualosaurus,(Jaklapalisaurus,(Nambalia,((Thecodontosaurus,Pantydraco),(Efraasia,(Plateosauravus,(Ruehleia,(((Unaysaurus,(Pradhania,Macrocollum)),((Sarahsaurus,((Ngwevu,((Coloradisaurus,(Glacialisaurus,Lufengosaurus)),(Xixipiosaurus,(Massospondylus_carinatus,(Adeopapposaurus,Leyesaurus))))),(Yunnanosaurus_huangi,((Seitaad,(Anchisaurus,((Irisosaurus,(Sefapanosaurus,(Yizhousaurus,(Aardonyx,(NMQR1551,(NMRQ3314,(Blikanasaurus,((Camelotia,((Meroktenos,(Ledumahadi,Kholumolumo)),(Lessemsaurus,(Ingentia,Antetonitrus)))),((Schleitheimia,(Isanosaurus,((Tazoudasaurus,Vulcanodon),((Shunosaurus,((Spinophorosaurus,Omeisaurus),(Mamenchisaurus,(Cetiosaurus,Neosauropoda)))),(Patagosaurus,Barapasaurus))))),(Pulanesaura,Gongxianosaurus)))))))))),(Mussaurus,Leonerasaurus)))),(Xingxiulong,(Jingshanosaurus,Chuxiongosaurus)))))),(Eucnemesaurus_fortis,(Riojasaurus,Eucnemesaurus_entaxonis)))),(Plateosaurus_gracilis,(Plateosaurus_ingens,Plateosaurus_engelhardti)))))))))))),(Panphagia,Pampadromaeus))))),(Staurikosaurus,Herrerasaurus)))))));

(Euparkeria,(Crurotarsi,(Marasuchus,(Silesaurus,(Ornithischia,(Agnosphitys,(((Guaibasaurus,(Neotheropoda,Chindesaurus)),((Panphagia,(Eoraptor,((Saturnalia,Chromogisaurus),(Bagualosaurus,(Jaklapalisaurus,(Nambalia,((Thecodontosaurus,Pantydraco),(Efraasia,(Plateosauravus,(Ruehleia,(((Unaysaurus,(Pradhania,Macrocollum)),((Sarahsaurus,((Ngwevu,(Xixipiosaurus,((Coloradisaurus,(Glacialisaurus,Lufengosaurus)),(Massospondylus_carinatus,(Adeopapposaurus,Leyesaurus))))),(Yunnanosaurus_huangi,((Seitaad,(Anchisaurus,((Irisosaurus,(Sefapanosaurus,(Yizhousaurus,(Aardonyx,(NMQR1551,(NMRQ3314,(Blikanasaurus,((Camelotia,((Meroktenos,(Ledumahadi,Kholumolumo)),(Lessemsaurus,(Ingentia,Antetonitrus)))),((Schleitheimia,(Isanosaurus,((Tazoudasaurus,Vulcanodon),((Shunosaurus,((Spinophorosaurus,Omeisaurus),(Mamenchisaurus,(Cetiosaurus,Neosauropoda)))),(Patagosaurus,Barapasaurus))))),(Pulanesaura,Gongxianosaurus)))))))))),(Mussaurus,Leonerasaurus)))),(Xingxiulong,(Jingshanosaurus,Chuxiongosaurus)))))),(Eucnemesaurus_fortis,(Riojasaurus,Eucnemesaurus_entaxonis)))),(Plateosaurus_gracilis,(Plateosaurus_ingens,Plateosaurus_engelhardti))))))))))))),(Buriolestes,Pampadromaeus))),(Staurikosaurus,Herrerasaurus))))))));

(Euparkeria,(Crurotarsi,(Marasuchus,(Silesaurus,(Ornithischia,((Agnosphitys,((Guaibasaurus,(Neotheropoda,Chindesaurus)),((Panphagia,(Eoraptor,((Saturnalia,Chromogisaurus),(Bagualosaurus,(Jaklapalisaurus,(Nambalia,((Thecodontosaurus,Pantydraco),(Efraasia,(Plateosauravus,(Ruehleia,(((Unaysaurus,(Pradhania,Macrocollum)),((Sarahsaurus,((Ngwevu,((Coloradisaurus,(Glacialisaurus,Lufengosaurus)),(Xixipiosaurus,(Massospondylus_carinatus,(Adeopapposaurus,Leyesaurus))))),(Yunnanosaurus_huangi,((Seitaad,(Anchisaurus,((Irisosaurus,(Sefapanosaurus,(Yizhousaurus,(Aardonyx,(NMQR1551,(NMRQ3314,(Blikanasaurus,((Camelotia,((Meroktenos,(Ledumahadi,Kholumolumo)),(Lessemsaurus,(Ingentia,Antetonitrus)))),((Schleitheimia,(Isanosaurus,((Tazoudasaurus,Vulcanodon),((Shunosaurus,((Spinophorosaurus,Omeisaurus),(Mamenchisaurus,(Cetiosaurus,Neosauropoda)))),(Patagosaurus,Barapasaurus))))),(Pulanesaura,Gongxianosaurus)))))))))),(Mussaurus,Leonerasaurus)))),(Xingxiulong,(Jingshanosaurus,Chuxiongosaurus)))))),(Eucnemesaurus_fortis,(Riojasaurus,Eucnemesaurus_entaxonis)))),(Plateosaurus_gracilis,(Plateosaurus_ingens,Plateosaurus_engelhardti))))))))))))),(Buriolestes,Pampadromaeus)))),(Staurikosaurus,Herrerasaurus)))))));

(Euparkeria,(Crurotarsi,(Marasuchus,(Silesaurus,(Ornithischia,((Agnosphitys,((Guaibasaurus,(Neotheropoda,Chindesaurus)),(Buriolestes,(Panphagia,(Eoraptor,((Saturnalia,Chromogisaurus),(Pampadromaeus,(Bagualosaurus,(Jaklapalisaurus,(Nambalia,((Thecodontosaurus,Pantydraco),(Efraasia,(Plateosauravus,(Ruehleia,(((Unaysaurus,(Pradhania,Macrocollum)),((Sarahsaurus,((Ngwevu,((Coloradisaurus,(Glacialisaurus,Lufengosaurus)),(Xixipiosaurus,(Massospondylus_carinatus,(Adeopapposaurus,Leyesaurus))))),(Yunnanosaurus_huangi,((Seitaad,(Anchisaurus,((Irisosaurus,(Sefapanosaurus,(Yizhousaurus,(Aardonyx,(NMQR1551,(NMRQ3314,(Blikanasaurus,((Camelotia,((Meroktenos,(Ledumahadi,Kholumolumo)),(Lessemsaurus,(Ingentia,Antetonitrus)))),((Schleitheimia,(Isanosaurus,((Tazoudasaurus,Vulcanodon),((Shunosaurus,((Spinophorosaurus,Omeisaurus),(Mamenchisaurus,(Cetiosaurus,Neosauropoda)))),(Patagosaurus,Barapasaurus))))),(Pulanesaura,Gongxianosaurus)))))))))),(Mussaurus,Leonerasaurus)))),(Xingxiulong,(Jingshanosaurus,Chuxiongosaurus)))))),(Eucnemesaurus_fortis,(Riojasaurus,Eucnemesaurus_entaxonis)))),(Plateosaurus_gracilis,(Plateosaurus_ingens,Plateosaurus_engelhardti))))))))))))))))),(Staurikosaurus,Herrerasaurus)))))));

(Euparkeria,(Crurotarsi,(Marasuchus,(Silesaurus,(Ornithischia,((Agnosphitys,((Guaibasaurus,(Neotheropoda,Chindesaurus)),(Buriolestes,(Panphagia,(Eoraptor,((Saturnalia,Chromogisaurus),(Pampadromaeus,(Bagualosaurus,(Jaklapalisaurus,(Nambalia,((Thecodontosaurus,Pantydraco),(Efraasia,(Plateosauravus,(Ruehleia,(((Unaysaurus,(Pradhania,Macrocollum)),((Sarahsaurus,((Ngwevu,((Coloradisaurus,(Glacialisaurus,Lufengosaurus)),(Xixipiosaurus,(Massospondylus_carinatus,(Adeopapposaurus,Leyesaurus))))),(Yunnanosaurus_huangi,((Seitaad,(Anchisaurus,((Irisosaurus,(Sefapanosaurus,(Yizhousaurus,(Aardonyx,(NMQR1551,(NMRQ3314,(Blikanasaurus,((Camelotia,((Meroktenos,(Ledumahadi,Kholumolumo)),(Lessemsaurus,(Ingentia,Antetonitrus)))),((Schleitheimia,(Isanosaurus,((Tazoudasaurus,Vulcanodon),((Shunosaurus,(Spinophorosaurus,(Mamenchisaurus,(Omeisaurus,(Cetiosaurus,Neosauropoda))))),(Patagosaurus,Barapasaurus))))),(Pulanesaura,Gongxianosaurus)))))))))),(Mussaurus,Leonerasaurus)))),(Xingxiulong,(Jingshanosaurus,Chuxiongosaurus)))))),(Eucnemesaurus_fortis,(Riojasaurus,Eucnemesaurus_entaxonis)))),(Plateosaurus_gracilis,(Plateosaurus_ingens,Plateosaurus_engelhardti))))))))))))))))),(Staurikosaurus,Herrerasaurus)))))));

(Euparkeria,(Crurotarsi,(Marasuchus,(Silesaurus,(Ornithischia,((Agnosphitys,((Guaibasaurus,(Neotheropoda,Chindesaurus)),(Buriolestes,(Panphagia,(Eoraptor,((Saturnalia,Chromogisaurus),(Pampadromaeus,(Bagualosaurus,(Jaklapalisaurus,(Nambalia,((Thecodontosaurus,Pantydraco),(Efraasia,(Plateosauravus,(Ruehleia,(((Unaysaurus,(Pradhania,Macrocollum)),((Sarahsaurus,((Ngwevu,((Coloradisaurus,(Glacialisaurus,Lufengosaurus)),(Xixipiosaurus,(Massospondylus_carinatus,(Adeopapposaurus,Leyesaurus))))),(Yunnanosaurus_huangi,((Seitaad,(Anchisaurus,((Irisosaurus,(Sefapanosaurus,(Yizhousaurus,(Aardonyx,(NMQR1551,(NMRQ3314,(Blikanasaurus,((Camelotia,((Meroktenos,(Ledumahadi,Kholumolumo)),(Lessemsaurus,(Ingentia,Antetonitrus)))),((Schleitheimia,(Isanosaurus,((Tazoudasaurus,Vulcanodon),((Shunosaurus,(Spinophorosaurus,(Omeisaurus,(Mamenchisaurus,(Cetiosaurus,Neosauropoda))))),(Patagosaurus,Barapasaurus))))),(Pulanesaura,Gongxianosaurus)))))))))),(Mussaurus,Leonerasaurus)))),(Xingxiulong,(Jingshanosaurus,Chuxiongosaurus)))))),(Eucnemesaurus_fortis,(Riojasaurus,Eucnemesaurus_entaxonis)))),(Plateosaurus_gracilis,(Plateosaurus_ingens,Plateosaurus_engelhardti))))))))))))))))),(Staurikosaurus,Herrerasaurus)))))));

(Euparkeria,(Crurotarsi,(Marasuchus,(Silesaurus,(Ornithischia,((Agnosphitys,((Guaibasaurus,(Neotheropoda,Chindesaurus)),(Buriolestes,(Panphagia,(Eoraptor,((Saturnalia,Chromogisaurus),(Pampadromaeus,(Bagualosaurus,(Jaklapalisaurus,(Nambalia,((Thecodontosaurus,Pantydraco),(Efraasia,(Plateosauravus,(Ruehleia,(((Unaysaurus,(Pradhania,Macrocollum)),((Sarahsaurus,((Ngwevu,(Xixipiosaurus,((Coloradisaurus,(Glacialisaurus,Lufengosaurus)),(Massospondylus_carinatus,(Adeopapposaurus,Leyesaurus))))),(Yunnanosaurus_huangi,((Seitaad,(Anchisaurus,((Irisosaurus,(Sefapanosaurus,(Yizhousaurus,(Aardonyx,(NMQR1551,(NMRQ3314,((Camelotia,((Meroktenos,(Ledumahadi,Kholumolumo)),(Lessemsaurus,(Blikanasaurus,(Ingentia,Antetonitrus))))),((Schleitheimia,(Isanosaurus,((Tazoudasaurus,Vulcanodon),((Shunosaurus,(Spinophorosaurus,(Omeisaurus,(Mamenchisaurus,(Cetiosaurus,Neosauropoda))))),(Patagosaurus,Barapasaurus))))),(Pulanesaura,Gongxianosaurus))))))))),(Mussaurus,Leonerasaurus)))),(Xingxiulong,(Jingshanosaurus,Chuxiongosaurus)))))),(Eucnemesaurus_fortis,(Riojasaurus,Eucnemesaurus_entaxonis)))),(Plateosaurus_gracilis,(Plateosaurus_ingens,Plateosaurus_engelhardti))))))))))))))))),(Staurikosaurus,Herrerasaurus)))))));

(Euparkeria,(Crurotarsi,(Marasuchus,(Silesaurus,(Agnosphitys,(Ornithischia,(((Guaibasaurus,(Neotheropoda,Chindesaurus)),(Buriolestes,(Pampadromaeus,(Panphagia,(Eoraptor,((Saturnalia,Chromogisaurus),(Bagualosaurus,(Jaklapalisaurus,(Nambalia,((Thecodontosaurus,Pantydraco),(Efraasia,(Plateosauravus,(Ruehleia,(((Unaysaurus,(Pradhania,Macrocollum)),((Sarahsaurus,((Ngwevu,(Xixipiosaurus,((Coloradisaurus,(Glacialisaurus,Lufengosaurus)),(Massospondylus_carinatus,(Adeopapposaurus,Leyesaurus))))),(Yunnanosaurus_huangi,((Seitaad,(Anchisaurus,((Irisosaurus,(Sefapanosaurus,(Yizhousaurus,(Aardonyx,(NMQR1551,(NMRQ3314,(Blikanasaurus,((Camelotia,((Meroktenos,(Ledumahadi,Kholumolumo)),(Lessemsaurus,(Ingentia,Antetonitrus)))),((Schleitheimia,(Isanosaurus,((Tazoudasaurus,Vulcanodon),((Shunosaurus,(Spinophorosaurus,(Mamenchisaurus,(Omeisaurus,(Cetiosaurus,Neosauropoda))))),(Patagosaurus,Barapasaurus))))),(Pulanesaura,Gongxianosaurus)))))))))),(Mussaurus,Leonerasaurus)))),(Xingxiulong,(Jingshanosaurus,Chuxiongosaurus)))))),(Eucnemesaurus_fortis,(Riojasaurus,Eucnemesaurus_entaxonis)))),(Plateosaurus_gracilis,(Plateosaurus_ingens,Plateosaurus_engelhardti)))))))))))))))),(Staurikosaurus,Herrerasaurus))))))));

(Euparkeria,(Crurotarsi,(Marasuchus,(Silesaurus,(Ornithischia,(Agnosphitys,(((Guaibasaurus,(Neotheropoda,Chindesaurus)),(Buriolestes,(Pampadromaeus,(Panphagia,(Eoraptor,((Saturnalia,Chromogisaurus),(Bagualosaurus,(Jaklapalisaurus,(Nambalia,((Thecodontosaurus,Pantydraco),(Efraasia,(Plateosauravus,(Ruehleia,(((Unaysaurus,(Pradhania,Macrocollum)),((Sarahsaurus,((Ngwevu,(Xixipiosaurus,((Coloradisaurus,(Glacialisaurus,Lufengosaurus)),(Massospondylus_carinatus,(Adeopapposaurus,Leyesaurus))))),(Yunnanosaurus_huangi,((Seitaad,(Anchisaurus,((Irisosaurus,(Sefapanosaurus,(Yizhousaurus,(Aardonyx,(NMQR1551,(NMRQ3314,(Blikanasaurus,((Camelotia,((Meroktenos,(Ledumahadi,Kholumolumo)),(Lessemsaurus,(Ingentia,Antetonitrus)))),((Schleitheimia,(Isanosaurus,((Tazoudasaurus,Vulcanodon),((Shunosaurus,(Spinophorosaurus,(Mamenchisaurus,(Omeisaurus,(Cetiosaurus,Neosauropoda))))),(Patagosaurus,Barapasaurus))))),(Pulanesaura,Gongxianosaurus)))))))))),(Mussaurus,Leonerasaurus)))),(Xingxiulong,(Jingshanosaurus,Chuxiongosaurus)))))),(Eucnemesaurus_fortis,(Riojasaurus,Eucnemesaurus_entaxonis)))),(Plateosaurus_gracilis,(Plateosaurus_ingens,Plateosaurus_engelhardti)))))))))))))))),(Staurikosaurus,Herrerasaurus))))))));

(Euparkeria,(Crurotarsi,(Marasuchus,(Silesaurus,(Ornithischia,((Agnosphitys,((Guaibasaurus,(Neotheropoda,Chindesaurus)),(Buriolestes,(Pampadromaeus,(Panphagia,(Eoraptor,((Saturnalia,Chromogisaurus),(Bagualosaurus,(Jaklapalisaurus,(Nambalia,((Thecodontosaurus,Pantydraco),(Efraasia,(Plateosauravus,(Ruehleia,(((Unaysaurus,(Pradhania,Macrocollum)),((Sarahsaurus,((Ngwevu,((Coloradisaurus,(Glacialisaurus,Lufengosaurus)),(Xixipiosaurus,(Massospondylus_carinatus,(Adeopapposaurus,Leyesaurus))))),(Yunnanosaurus_huangi,((Seitaad,(Anchisaurus,((Irisosaurus,(Sefapanosaurus,(Yizhousaurus,(Aardonyx,(NMQR1551,(NMRQ3314,(Blikanasaurus,((Camelotia,((Meroktenos,(Ledumahadi,Kholumolumo)),(Lessemsaurus,(Ingentia,Antetonitrus)))),((Schleitheimia,(Isanosaurus,((Tazoudasaurus,Vulcanodon),((Shunosaurus,(Spinophorosaurus,(Mamenchisaurus,(Omeisaurus,(Cetiosaurus,Neosauropoda))))),(Patagosaurus,Barapasaurus))))),(Pulanesaura,Gongxianosaurus)))))))))),(Mussaurus,Leonerasaurus)))),(Xingxiulong,(Jingshanosaurus,Chuxiongosaurus)))))),(Eucnemesaurus_fortis,(Riojasaurus,Eucnemesaurus_entaxonis)))),(Plateosaurus_gracilis,(Plateosaurus_ingens,Plateosaurus_engelhardti))))))))))))))))),(Staurikosaurus,Herrerasaurus)))))));

(Euparkeria,(Crurotarsi,(Marasuchus,(Silesaurus,(Ornithischia,((Agnosphitys,((Guaibasaurus,(Neotheropoda,Chindesaurus)),((Eoraptor,((Saturnalia,Chromogisaurus),(Bagualosaurus,(Jaklapalisaurus,(Nambalia,((Thecodontosaurus,Pantydraco),(Efraasia,(Plateosauravus,(Ruehleia,(((Unaysaurus,(Pradhania,Macrocollum)),((Sarahsaurus,((Ngwevu,(Xixipiosaurus,((Coloradisaurus,(Glacialisaurus,Lufengosaurus)),(Massospondylus_carinatus,(Adeopapposaurus,Leyesaurus))))),(Yunnanosaurus_huangi,((Seitaad,(Anchisaurus,((Irisosaurus,(Sefapanosaurus,(Yizhousaurus,(Aardonyx,(NMQR1551,(NMRQ3314,(Blikanasaurus,((Camelotia,((Meroktenos,(Ledumahadi,Kholumolumo)),(Lessemsaurus,(Ingentia,Antetonitrus)))),((Schleitheimia,(Isanosaurus,((Tazoudasaurus,Vulcanodon),((Shunosaurus,(Spinophorosaurus,(Mamenchisaurus,(Omeisaurus,(Cetiosaurus,Neosauropoda))))),(Patagosaurus,Barapasaurus))))),(Pulanesaura,Gongxianosaurus)))))))))),(Mussaurus,Leonerasaurus)))),(Xingxiulong,(Jingshanosaurus,Chuxiongosaurus)))))),(Eucnemesaurus_fortis,(Riojasaurus,Eucnemesaurus_entaxonis)))),(Plateosaurus_gracilis,(Plateosaurus_ingens,Plateosaurus_engelhardti)))))))))))),(Buriolestes,(Panphagia,Pampadromaeus))))),(Staurikosaurus,Herrerasaurus)))))));

(Euparkeria,(Crurotarsi,(Marasuchus,(Silesaurus,(Ornithischia,((Agnosphitys,((Guaibasaurus,(Neotheropoda,Chindesaurus)),(Buriolestes,((Eoraptor,((Saturnalia,Chromogisaurus),(Bagualosaurus,(Jaklapalisaurus,(Nambalia,((Thecodontosaurus,Pantydraco),(Efraasia,(Plateosauravus,(Ruehleia,(((Unaysaurus,(Pradhania,Macrocollum)),((Sarahsaurus,((Ngwevu,((Coloradisaurus,(Glacialisaurus,Lufengosaurus)),(Xixipiosaurus,(Massospondylus_carinatus,(Adeopapposaurus,Leyesaurus))))),(Yunnanosaurus_huangi,((Seitaad,(Anchisaurus,((Irisosaurus,(Sefapanosaurus,(Yizhousaurus,(Aardonyx,(NMQR1551,(NMRQ3314,(Blikanasaurus,((Camelotia,((Meroktenos,(Ledumahadi,Kholumolumo)),(Lessemsaurus,(Ingentia,Antetonitrus)))),((Schleitheimia,(Isanosaurus,((Tazoudasaurus,Vulcanodon),((Shunosaurus,(Spinophorosaurus,(Mamenchisaurus,(Omeisaurus,(Cetiosaurus,Neosauropoda))))),(Patagosaurus,Barapasaurus))))),(Pulanesaura,Gongxianosaurus)))))))))),(Mussaurus,Leonerasaurus)))),(Xingxiulong,(Jingshanosaurus,Chuxiongosaurus)))))),(Eucnemesaurus_fortis,(Riojasaurus,Eucnemesaurus_entaxonis)))),(Plateosaurus_gracilis,(Plateosaurus_ingens,Plateosaurus_engelhardti)))))))))))),(Panphagia,Pampadromaeus))))),(Staurikosaurus,Herrerasaurus)))))));

(Euparkeria,(Crurotarsi,(Marasuchus,(Silesaurus,(Ornithischia,(Agnosphitys,(((Guaibasaurus,(Neotheropoda,Chindesaurus)),((Panphagia,(Eoraptor,((Saturnalia,Chromogisaurus),(Bagualosaurus,(Jaklapalisaurus,(Nambalia,((Thecodontosaurus,Pantydraco),(Efraasia,(Plateosauravus,(Ruehleia,(((Unaysaurus,(Pradhania,Macrocollum)),((Sarahsaurus,((Ngwevu,(Xixipiosaurus,((Coloradisaurus,(Glacialisaurus,Lufengosaurus)),(Massospondylus_carinatus,(Adeopapposaurus,Leyesaurus))))),(Yunnanosaurus_huangi,((Seitaad,(Anchisaurus,((Irisosaurus,(Sefapanosaurus,(Yizhousaurus,(Aardonyx,(NMQR1551,(NMRQ3314,(Blikanasaurus,((Camelotia,((Meroktenos,(Ledumahadi,Kholumolumo)),(Lessemsaurus,(Ingentia,Antetonitrus)))),((Schleitheimia,(Isanosaurus,((Tazoudasaurus,Vulcanodon),((Shunosaurus,(Spinophorosaurus,(Mamenchisaurus,(Omeisaurus,(Cetiosaurus,Neosauropoda))))),(Patagosaurus,Barapasaurus))))),(Pulanesaura,Gongxianosaurus)))))))))),(Mussaurus,Leonerasaurus)))),(Xingxiulong,(Jingshanosaurus,Chuxiongosaurus)))))),(Eucnemesaurus_fortis,(Riojasaurus,Eucnemesaurus_entaxonis)))),(Plateosaurus_gracilis,(Plateosaurus_ingens,Plateosaurus_engelhardti))))))))))))),(Buriolestes,Pampadromaeus))),(Staurikosaurus,Herrerasaurus))))))));

(Euparkeria,(Crurotarsi,(Marasuchus,(Silesaurus,(Ornithischia,((Agnosphitys,((Guaibasaurus,(Neotheropoda,Chindesaurus)),((Panphagia,(Eoraptor,((Saturnalia,Chromogisaurus),(Bagualosaurus,(Jaklapalisaurus,(Nambalia,((Thecodontosaurus,Pantydraco),(Efraasia,(Plateosauravus,(Ruehleia,(((Unaysaurus,(Pradhania,Macrocollum)),((Sarahsaurus,((Ngwevu,((Coloradisaurus,(Glacialisaurus,Lufengosaurus)),(Xixipiosaurus,(Massospondylus_carinatus,(Adeopapposaurus,Leyesaurus))))),(Yunnanosaurus_huangi,((Seitaad,(Anchisaurus,((Irisosaurus,(Sefapanosaurus,(Yizhousaurus,(Aardonyx,(NMQR1551,(NMRQ3314,(Blikanasaurus,((Camelotia,((Meroktenos,(Ledumahadi,Kholumolumo)),(Lessemsaurus,(Ingentia,Antetonitrus)))),((Schleitheimia,(Isanosaurus,((Tazoudasaurus,Vulcanodon),((Shunosaurus,(Spinophorosaurus,(Mamenchisaurus,(Omeisaurus,(Cetiosaurus,Neosauropoda))))),(Patagosaurus,Barapasaurus))))),(Pulanesaura,Gongxianosaurus)))))))))),(Mussaurus,Leonerasaurus)))),(Xingxiulong,(Jingshanosaurus,Chuxiongosaurus)))))),(Eucnemesaurus_fortis,(Riojasaurus,Eucnemesaurus_entaxonis)))),(Plateosaurus_gracilis,(Plateosaurus_ingens,Plateosaurus_engelhardti))))))))))))),(Buriolestes,Pampadromaeus)))),(Staurikosaurus,Herrerasaurus)))))));

(Euparkeria,(Crurotarsi,(Marasuchus,(Silesaurus,(Agnosphitys,(Ornithischia,(((Guaibasaurus,(Neotheropoda,Chindesaurus)),(Buriolestes,(Pampadromaeus,(Panphagia,(Eoraptor,((Saturnalia,Chromogisaurus),(Bagualosaurus,(Jaklapalisaurus,(Nambalia,((Thecodontosaurus,Pantydraco),(Efraasia,(Plateosauravus,(Ruehleia,(((Unaysaurus,(Pradhania,Macrocollum)),((Sarahsaurus,((Ngwevu,(Xixipiosaurus,((Coloradisaurus,(Glacialisaurus,Lufengosaurus)),(Massospondylus_carinatus,(Adeopapposaurus,Leyesaurus))))),(Yunnanosaurus_huangi,((Seitaad,(Anchisaurus,((Irisosaurus,(Sefapanosaurus,(Yizhousaurus,(Aardonyx,(NMQR1551,(NMRQ3314,(Blikanasaurus,((Camelotia,((Meroktenos,(Ledumahadi,Kholumolumo)),(Lessemsaurus,(Ingentia,Antetonitrus)))),((Schleitheimia,(Isanosaurus,((Tazoudasaurus,Vulcanodon),((Shunosaurus,(Spinophorosaurus,(Omeisaurus,(Mamenchisaurus,(Cetiosaurus,Neosauropoda))))),(Patagosaurus,Barapasaurus))))),(Pulanesaura,Gongxianosaurus)))))))))),(Mussaurus,Leonerasaurus)))),(Xingxiulong,(Jingshanosaurus,Chuxiongosaurus)))))),(Eucnemesaurus_fortis,(Riojasaurus,Eucnemesaurus_entaxonis)))),(Plateosaurus_gracilis,(Plateosaurus_ingens,Plateosaurus_engelhardti)))))))))))))))),(Staurikosaurus,Herrerasaurus))))))));

(Euparkeria,(Crurotarsi,(Marasuchus,(Silesaurus,(Ornithischia,(Agnosphitys,(((Guaibasaurus,(Neotheropoda,Chindesaurus)),(Buriolestes,(Pampadromaeus,(Panphagia,(Eoraptor,((Saturnalia,Chromogisaurus),(Bagualosaurus,(Jaklapalisaurus,(Nambalia,((Thecodontosaurus,Pantydraco),(Efraasia,(Plateosauravus,(Ruehleia,(((Unaysaurus,(Pradhania,Macrocollum)),((Sarahsaurus,((Ngwevu,(Xixipiosaurus,((Coloradisaurus,(Glacialisaurus,Lufengosaurus)),(Massospondylus_carinatus,(Adeopapposaurus,Leyesaurus))))),(Yunnanosaurus_huangi,((Seitaad,(Anchisaurus,((Irisosaurus,(Sefapanosaurus,(Yizhousaurus,(Aardonyx,(NMQR1551,(NMRQ3314,(Blikanasaurus,((Camelotia,((Meroktenos,(Ledumahadi,Kholumolumo)),(Lessemsaurus,(Ingentia,Antetonitrus)))),((Schleitheimia,(Isanosaurus,((Tazoudasaurus,Vulcanodon),((Shunosaurus,(Spinophorosaurus,(Omeisaurus,(Mamenchisaurus,(Cetiosaurus,Neosauropoda))))),(Patagosaurus,Barapasaurus))))),(Pulanesaura,Gongxianosaurus)))))))))),(Mussaurus,Leonerasaurus)))),(Xingxiulong,(Jingshanosaurus,Chuxiongosaurus)))))),(Eucnemesaurus_fortis,(Riojasaurus,Eucnemesaurus_entaxonis)))),(Plateosaurus_gracilis,(Plateosaurus_ingens,Plateosaurus_engelhardti)))))))))))))))),(Staurikosaurus,Herrerasaurus))))))));

(Euparkeria,(Crurotarsi,(Marasuchus,(Silesaurus,(Ornithischia,((Agnosphitys,((Guaibasaurus,(Neotheropoda,Chindesaurus)),(Buriolestes,(Pampadromaeus,(Panphagia,(Eoraptor,((Saturnalia,Chromogisaurus),(Bagualosaurus,(Jaklapalisaurus,(Nambalia,((Thecodontosaurus,Pantydraco),(Efraasia,(Plateosauravus,(Ruehleia,(((Unaysaurus,(Pradhania,Macrocollum)),((Sarahsaurus,((Ngwevu,((Coloradisaurus,(Glacialisaurus,Lufengosaurus)),(Xixipiosaurus,(Massospondylus_carinatus,(Adeopapposaurus,Leyesaurus))))),(Yunnanosaurus_huangi,((Seitaad,(Anchisaurus,((Irisosaurus,(Sefapanosaurus,(Yizhousaurus,(Aardonyx,(NMQR1551,(NMRQ3314,(Blikanasaurus,((Camelotia,((Meroktenos,(Ledumahadi,Kholumolumo)),(Lessemsaurus,(Ingentia,Antetonitrus)))),((Schleitheimia,(Isanosaurus,((Tazoudasaurus,Vulcanodon),((Shunosaurus,(Spinophorosaurus,(Omeisaurus,(Mamenchisaurus,(Cetiosaurus,Neosauropoda))))),(Patagosaurus,Barapasaurus))))),(Pulanesaura,Gongxianosaurus)))))))))),(Mussaurus,Leonerasaurus)))),(Xingxiulong,(Jingshanosaurus,Chuxiongosaurus)))))),(Eucnemesaurus_fortis,(Riojasaurus,Eucnemesaurus_entaxonis)))),(Plateosaurus_gracilis,(Plateosaurus_ingens,Plateosaurus_engelhardti))))))))))))))))),(Staurikosaurus,Herrerasaurus)))))));

(Euparkeria,(Crurotarsi,(Marasuchus,(Silesaurus,(Ornithischia,((Agnosphitys,((Guaibasaurus,(Neotheropoda,Chindesaurus)),(Buriolestes,(Pampadromaeus,(Panphagia,(Eoraptor,((Saturnalia,Chromogisaurus),(Bagualosaurus,(Jaklapalisaurus,(Nambalia,((Thecodontosaurus,Pantydraco),(Efraasia,(Plateosauravus,(Ruehleia,(((Unaysaurus,(Pradhania,Macrocollum)),((Sarahsaurus,((Ngwevu,(Xixipiosaurus,((Coloradisaurus,(Glacialisaurus,Lufengosaurus)),(Massospondylus_carinatus,(Adeopapposaurus,Leyesaurus))))),(Yunnanosaurus_huangi,((Seitaad,(Anchisaurus,((Irisosaurus,(Sefapanosaurus,(Yizhousaurus,(Aardonyx,(NMQR1551,(NMRQ3314,((Camelotia,((Meroktenos,(Ledumahadi,Kholumolumo)),(Lessemsaurus,(Blikanasaurus,(Ingentia,Antetonitrus))))),((Schleitheimia,(Isanosaurus,((Tazoudasaurus,Vulcanodon),((Shunosaurus,(Spinophorosaurus,(Omeisaurus,(Mamenchisaurus,(Cetiosaurus,Neosauropoda))))),(Patagosaurus,Barapasaurus))))),(Pulanesaura,Gongxianosaurus))))))))),(Mussaurus,Leonerasaurus)))),(Xingxiulong,(Jingshanosaurus,Chuxiongosaurus)))))),(Eucnemesaurus_fortis,(Riojasaurus,Eucnemesaurus_entaxonis)))),(Plateosaurus_gracilis,(Plateosaurus_ingens,Plateosaurus_engelhardti))))))))))))))))),(Staurikosaurus,Herrerasaurus)))))));

(Euparkeria,(Crurotarsi,(Marasuchus,(Silesaurus,(Ornithischia,((Agnosphitys,((Guaibasaurus,(Neotheropoda,Chindesaurus)),((Eoraptor,((Saturnalia,Chromogisaurus),(Bagualosaurus,(Jaklapalisaurus,(Nambalia,((Thecodontosaurus,Pantydraco),(Efraasia,(Plateosauravus,(Ruehleia,(((Unaysaurus,(Pradhania,Macrocollum)),((Sarahsaurus,((Ngwevu,(Xixipiosaurus,((Coloradisaurus,(Glacialisaurus,Lufengosaurus)),(Massospondylus_carinatus,(Adeopapposaurus,Leyesaurus))))),(Yunnanosaurus_huangi,((Seitaad,(Anchisaurus,((Irisosaurus,(Sefapanosaurus,(Yizhousaurus,(Aardonyx,(NMQR1551,(NMRQ3314,(Blikanasaurus,((Camelotia,((Meroktenos,(Ledumahadi,Kholumolumo)),(Lessemsaurus,(Ingentia,Antetonitrus)))),((Schleitheimia,(Isanosaurus,((Tazoudasaurus,Vulcanodon),((Shunosaurus,(Spinophorosaurus,(Omeisaurus,(Mamenchisaurus,(Cetiosaurus,Neosauropoda))))),(Patagosaurus,Barapasaurus))))),(Pulanesaura,Gongxianosaurus)))))))))),(Mussaurus,Leonerasaurus)))),(Xingxiulong,(Jingshanosaurus,Chuxiongosaurus)))))),(Eucnemesaurus_fortis,(Riojasaurus,Eucnemesaurus_entaxonis)))),(Plateosaurus_gracilis,(Plateosaurus_ingens,Plateosaurus_engelhardti)))))))))))),(Buriolestes,(Panphagia,Pampadromaeus))))),(Staurikosaurus,Herrerasaurus)))))));

(Euparkeria,(Crurotarsi,(Marasuchus,(Silesaurus,(Ornithischia,((Agnosphitys,((Guaibasaurus,(Neotheropoda,Chindesaurus)),(Buriolestes,((Eoraptor,((Saturnalia,Chromogisaurus),(Bagualosaurus,(Jaklapalisaurus,(Nambalia,((Thecodontosaurus,Pantydraco),(Efraasia,(Plateosauravus,(Ruehleia,(((Unaysaurus,(Pradhania,Macrocollum)),((Sarahsaurus,((Ngwevu,((Coloradisaurus,(Glacialisaurus,Lufengosaurus)),(Xixipiosaurus,(Massospondylus_carinatus,(Adeopapposaurus,Leyesaurus))))),(Yunnanosaurus_huangi,((Seitaad,(Anchisaurus,((Irisosaurus,(Sefapanosaurus,(Yizhousaurus,(Aardonyx,(NMQR1551,(NMRQ3314,(Blikanasaurus,((Camelotia,((Meroktenos,(Ledumahadi,Kholumolumo)),(Lessemsaurus,(Ingentia,Antetonitrus)))),((Schleitheimia,(Isanosaurus,((Tazoudasaurus,Vulcanodon),((Shunosaurus,(Spinophorosaurus,(Omeisaurus,(Mamenchisaurus,(Cetiosaurus,Neosauropoda))))),(Patagosaurus,Barapasaurus))))),(Pulanesaura,Gongxianosaurus)))))))))),(Mussaurus,Leonerasaurus)))),(Xingxiulong,(Jingshanosaurus,Chuxiongosaurus)))))),(Eucnemesaurus_fortis,(Riojasaurus,Eucnemesaurus_entaxonis)))),(Plateosaurus_gracilis,(Plateosaurus_ingens,Plateosaurus_engelhardti)))))))))))),(Panphagia,Pampadromaeus))))),(Staurikosaurus,Herrerasaurus)))))));

(Euparkeria,(Crurotarsi,(Marasuchus,(Silesaurus,(Ornithischia,((Agnosphitys,((Guaibasaurus,(Neotheropoda,Chindesaurus)),(Buriolestes,((Eoraptor,((Saturnalia,Chromogisaurus),(Bagualosaurus,(Jaklapalisaurus,(Nambalia,((Thecodontosaurus,Pantydraco),(Efraasia,(Plateosauravus,(Ruehleia,(((Unaysaurus,(Pradhania,Macrocollum)),((Sarahsaurus,((Ngwevu,(Xixipiosaurus,((Coloradisaurus,(Glacialisaurus,Lufengosaurus)),(Massospondylus_carinatus,(Adeopapposaurus,Leyesaurus))))),(Yunnanosaurus_huangi,((Seitaad,(Anchisaurus,((Irisosaurus,(Sefapanosaurus,(Yizhousaurus,(Aardonyx,(NMQR1551,(NMRQ3314,((Camelotia,((Meroktenos,(Ledumahadi,Kholumolumo)),(Lessemsaurus,(Blikanasaurus,(Ingentia,Antetonitrus))))),((Schleitheimia,(Isanosaurus,((Tazoudasaurus,Vulcanodon),((Shunosaurus,(Spinophorosaurus,(Omeisaurus,(Mamenchisaurus,(Cetiosaurus,Neosauropoda))))),(Patagosaurus,Barapasaurus))))),(Pulanesaura,Gongxianosaurus))))))))),(Mussaurus,Leonerasaurus)))),(Xingxiulong,(Jingshanosaurus,Chuxiongosaurus)))))),(Eucnemesaurus_fortis,(Riojasaurus,Eucnemesaurus_entaxonis)))),(Plateosaurus_gracilis,(Plateosaurus_ingens,Plateosaurus_engelhardti)))))))))))),(Panphagia,Pampadromaeus))))),(Staurikosaurus,Herrerasaurus)))))));

(Euparkeria,(Crurotarsi,(Marasuchus,(Silesaurus,(Ornithischia,(Agnosphitys,(((Guaibasaurus,(Neotheropoda,Chindesaurus)),((Panphagia,(Eoraptor,((Saturnalia,Chromogisaurus),(Bagualosaurus,(Jaklapalisaurus,(Nambalia,((Thecodontosaurus,Pantydraco),(Efraasia,(Plateosauravus,(Ruehleia,(((Unaysaurus,(Pradhania,Macrocollum)),((Sarahsaurus,((Ngwevu,(Xixipiosaurus,((Coloradisaurus,(Glacialisaurus,Lufengosaurus)),(Massospondylus_carinatus,(Adeopapposaurus,Leyesaurus))))),(Yunnanosaurus_huangi,((Seitaad,(Anchisaurus,((Irisosaurus,(Sefapanosaurus,(Yizhousaurus,(Aardonyx,(NMQR1551,(NMRQ3314,(Blikanasaurus,((Camelotia,((Meroktenos,(Ledumahadi,Kholumolumo)),(Lessemsaurus,(Ingentia,Antetonitrus)))),((Schleitheimia,(Isanosaurus,((Tazoudasaurus,Vulcanodon),((Shunosaurus,(Spinophorosaurus,(Omeisaurus,(Mamenchisaurus,(Cetiosaurus,Neosauropoda))))),(Patagosaurus,Barapasaurus))))),(Pulanesaura,Gongxianosaurus)))))))))),(Mussaurus,Leonerasaurus)))),(Xingxiulong,(Jingshanosaurus,Chuxiongosaurus)))))),(Eucnemesaurus_fortis,(Riojasaurus,Eucnemesaurus_entaxonis)))),(Plateosaurus_gracilis,(Plateosaurus_ingens,Plateosaurus_engelhardti))))))))))))),(Buriolestes,Pampadromaeus))),(Staurikosaurus,Herrerasaurus))))))));

(Euparkeria,(Crurotarsi,(Marasuchus,(Silesaurus,(Ornithischia,((Agnosphitys,((Guaibasaurus,(Neotheropoda,Chindesaurus)),((Panphagia,(Eoraptor,((Saturnalia,Chromogisaurus),(Bagualosaurus,(Jaklapalisaurus,(Nambalia,((Thecodontosaurus,Pantydraco),(Efraasia,(Plateosauravus,(Ruehleia,(((Unaysaurus,(Pradhania,Macrocollum)),((Sarahsaurus,((Ngwevu,((Coloradisaurus,(Glacialisaurus,Lufengosaurus)),(Xixipiosaurus,(Massospondylus_carinatus,(Adeopapposaurus,Leyesaurus))))),(Yunnanosaurus_huangi,((Seitaad,(Anchisaurus,((Irisosaurus,(Sefapanosaurus,(Yizhousaurus,(Aardonyx,(NMQR1551,(NMRQ3314,(Blikanasaurus,((Camelotia,((Meroktenos,(Ledumahadi,Kholumolumo)),(Lessemsaurus,(Ingentia,Antetonitrus)))),((Schleitheimia,(Isanosaurus,((Tazoudasaurus,Vulcanodon),((Shunosaurus,(Spinophorosaurus,(Omeisaurus,(Mamenchisaurus,(Cetiosaurus,Neosauropoda))))),(Patagosaurus,Barapasaurus))))),(Pulanesaura,Gongxianosaurus)))))))))),(Mussaurus,Leonerasaurus)))),(Xingxiulong,(Jingshanosaurus,Chuxiongosaurus)))))),(Eucnemesaurus_fortis,(Riojasaurus,Eucnemesaurus_entaxonis)))),(Plateosaurus_gracilis,(Plateosaurus_ingens,Plateosaurus_engelhardti))))))))))))),(Buriolestes,Pampadromaeus)))),(Staurikosaurus,Herrerasaurus)))))));

(Euparkeria,(Crurotarsi,(Marasuchus,(Silesaurus,(Ornithischia,((Agnosphitys,((Guaibasaurus,(Neotheropoda,Chindesaurus)),((Panphagia,(Eoraptor,((Saturnalia,Chromogisaurus),(Bagualosaurus,(Jaklapalisaurus,(Nambalia,((Thecodontosaurus,Pantydraco),(Efraasia,(Plateosauravus,(Ruehleia,(((Unaysaurus,(Pradhania,Macrocollum)),((Sarahsaurus,((Ngwevu,(Xixipiosaurus,((Coloradisaurus,(Glacialisaurus,Lufengosaurus)),(Massospondylus_carinatus,(Adeopapposaurus,Leyesaurus))))),(Yunnanosaurus_huangi,((Seitaad,(Anchisaurus,((Irisosaurus,(Sefapanosaurus,(Yizhousaurus,(Aardonyx,(NMQR1551,(NMRQ3314,((Camelotia,((Meroktenos,(Ledumahadi,Kholumolumo)),(Lessemsaurus,(Blikanasaurus,(Ingentia,Antetonitrus))))),((Schleitheimia,(Isanosaurus,((Tazoudasaurus,Vulcanodon),((Shunosaurus,(Spinophorosaurus,(Omeisaurus,(Mamenchisaurus,(Cetiosaurus,Neosauropoda))))),(Patagosaurus,Barapasaurus))))),(Pulanesaura,Gongxianosaurus))))))))),(Mussaurus,Leonerasaurus)))),(Xingxiulong,(Jingshanosaurus,Chuxiongosaurus)))))),(Eucnemesaurus_fortis,(Riojasaurus,Eucnemesaurus_entaxonis)))),(Plateosaurus_gracilis,(Plateosaurus_ingens,Plateosaurus_engelhardti))))))))))))),(Buriolestes,Pampadromaeus)))),(Staurikosaurus,Herrerasaurus)))))));

(Euparkeria,(Crurotarsi,(Marasuchus,(Silesaurus,(Ornithischia,((Agnosphitys,((Guaibasaurus,(Neotheropoda,Chindesaurus)),(Buriolestes,(Panphagia,(Eoraptor,((Pampadromaeus,(Saturnalia,Chromogisaurus)),(Bagualosaurus,(Jaklapalisaurus,(Nambalia,((Thecodontosaurus,Pantydraco),(Efraasia,(Plateosauravus,(Ruehleia,(((Unaysaurus,(Pradhania,Macrocollum)),((Sarahsaurus,((Ngwevu,((Coloradisaurus,(Glacialisaurus,Lufengosaurus)),(Xixipiosaurus,(Massospondylus_carinatus,(Adeopapposaurus,Leyesaurus))))),(Yunnanosaurus_huangi,((Seitaad,(Anchisaurus,((Irisosaurus,(Sefapanosaurus,(Yizhousaurus,(Aardonyx,(NMQR1551,(NMRQ3314,((Camelotia,((Meroktenos,(Ledumahadi,Kholumolumo)),(Lessemsaurus,(Blikanasaurus,(Ingentia,Antetonitrus))))),((Schleitheimia,(Isanosaurus,((Tazoudasaurus,Vulcanodon),((Shunosaurus,(Spinophorosaurus,(Omeisaurus,(Mamenchisaurus,(Cetiosaurus,Neosauropoda))))),(Patagosaurus,Barapasaurus))))),(Pulanesaura,Gongxianosaurus))))))))),(Mussaurus,Leonerasaurus)))),(Xingxiulong,(Jingshanosaurus,Chuxiongosaurus)))))),(Eucnemesaurus_fortis,(Riojasaurus,Eucnemesaurus_entaxonis)))),(Plateosaurus_gracilis,(Plateosaurus_ingens,Plateosaurus_engelhardti)))))))))))))))),(Staurikosaurus,Herrerasaurus)))))));

(Euparkeria,(Crurotarsi,(Marasuchus,(Silesaurus,(Agnosphitys,(Ornithischia,(((Guaibasaurus,(Neotheropoda,Chindesaurus)),(Buriolestes,(Pampadromaeus,(Panphagia,(Eoraptor,((Saturnalia,Chromogisaurus),(Bagualosaurus,(Jaklapalisaurus,(Nambalia,((Thecodontosaurus,Pantydraco),(Efraasia,(Plateosauravus,(Ruehleia,(((Unaysaurus,(Pradhania,Macrocollum)),((Sarahsaurus,((Ngwevu,((Coloradisaurus,(Glacialisaurus,Lufengosaurus)),(Xixipiosaurus,(Massospondylus_carinatus,(Adeopapposaurus,Leyesaurus))))),(Yunnanosaurus_huangi,((Seitaad,(Anchisaurus,((Irisosaurus,(Sefapanosaurus,(Yizhousaurus,(Aardonyx,(NMQR1551,(NMRQ3314,(Blikanasaurus,((Camelotia,((Meroktenos,(Ledumahadi,Kholumolumo)),(Lessemsaurus,(Ingentia,Antetonitrus)))),((Schleitheimia,(Isanosaurus,((Tazoudasaurus,Vulcanodon),((Shunosaurus,((Spinophorosaurus,(Omeisaurus,Mamenchisaurus)),(Cetiosaurus,Neosauropoda))),(Patagosaurus,Barapasaurus))))),(Pulanesaura,Gongxianosaurus)))))))))),(Mussaurus,Leonerasaurus)))),(Xingxiulong,(Jingshanosaurus,Chuxiongosaurus)))))),(Eucnemesaurus_fortis,(Riojasaurus,Eucnemesaurus_entaxonis)))),(Plateosaurus_gracilis,(Plateosaurus_ingens,Plateosaurus_engelhardti)))))))))))))))),(Staurikosaurus,Herrerasaurus))))))));

(Euparkeria,(Crurotarsi,(Marasuchus,(Silesaurus,(Ornithischia,(Agnosphitys,(((Guaibasaurus,(Neotheropoda,Chindesaurus)),(Buriolestes,(Pampadromaeus,(Panphagia,(Eoraptor,((Saturnalia,Chromogisaurus),(Bagualosaurus,(Jaklapalisaurus,(Nambalia,((Thecodontosaurus,Pantydraco),(Efraasia,(Plateosauravus,(Ruehleia,(((Unaysaurus,(Pradhania,Macrocollum)),((Sarahsaurus,((Ngwevu,((Coloradisaurus,(Glacialisaurus,Lufengosaurus)),(Xixipiosaurus,(Massospondylus_carinatus,(Adeopapposaurus,Leyesaurus))))),(Yunnanosaurus_huangi,((Seitaad,(Anchisaurus,((Irisosaurus,(Sefapanosaurus,(Yizhousaurus,(Aardonyx,(NMQR1551,(NMRQ3314,(Blikanasaurus,((Camelotia,((Meroktenos,(Ledumahadi,Kholumolumo)),(Lessemsaurus,(Ingentia,Antetonitrus)))),((Schleitheimia,(Isanosaurus,((Tazoudasaurus,Vulcanodon),((Shunosaurus,((Spinophorosaurus,(Omeisaurus,Mamenchisaurus)),(Cetiosaurus,Neosauropoda))),(Patagosaurus,Barapasaurus))))),(Pulanesaura,Gongxianosaurus)))))))))),(Mussaurus,Leonerasaurus)))),(Xingxiulong,(Jingshanosaurus,Chuxiongosaurus)))))),(Eucnemesaurus_fortis,(Riojasaurus,Eucnemesaurus_entaxonis)))),(Plateosaurus_gracilis,(Plateosaurus_ingens,Plateosaurus_engelhardti)))))))))))))))),(Staurikosaurus,Herrerasaurus))))))));

(Euparkeria,(Crurotarsi,(Marasuchus,(Silesaurus,(Ornithischia,((Agnosphitys,((Guaibasaurus,(Neotheropoda,Chindesaurus)),((Eoraptor,((Saturnalia,Chromogisaurus),(Bagualosaurus,(Jaklapalisaurus,(Nambalia,((Thecodontosaurus,Pantydraco),(Efraasia,(Plateosauravus,(Ruehleia,(((Unaysaurus,(Pradhania,Macrocollum)),((Sarahsaurus,((Ngwevu,((Coloradisaurus,(Glacialisaurus,Lufengosaurus)),(Xixipiosaurus,(Massospondylus_carinatus,(Adeopapposaurus,Leyesaurus))))),(Yunnanosaurus_huangi,((Seitaad,(Anchisaurus,((Irisosaurus,(Sefapanosaurus,(Yizhousaurus,(Aardonyx,(NMQR1551,(NMRQ3314,(Blikanasaurus,((Camelotia,((Meroktenos,(Ledumahadi,Kholumolumo)),(Lessemsaurus,(Ingentia,Antetonitrus)))),((Schleitheimia,(Isanosaurus,((Tazoudasaurus,Vulcanodon),((Shunosaurus,((Spinophorosaurus,(Omeisaurus,Mamenchisaurus)),(Cetiosaurus,Neosauropoda))),(Patagosaurus,Barapasaurus))))),(Pulanesaura,Gongxianosaurus)))))))))),(Mussaurus,Leonerasaurus)))),(Xingxiulong,(Jingshanosaurus,Chuxiongosaurus)))))),(Eucnemesaurus_fortis,(Riojasaurus,Eucnemesaurus_entaxonis)))),(Plateosaurus_gracilis,(Plateosaurus_ingens,Plateosaurus_engelhardti)))))))))))),(Buriolestes,(Panphagia,Pampadromaeus))))),(Staurikosaurus,Herrerasaurus)))))));

(Euparkeria,(Crurotarsi,(Marasuchus,(Silesaurus,(Ornithischia,(Agnosphitys,(((Guaibasaurus,(Neotheropoda,Chindesaurus)),((Panphagia,(Eoraptor,((Saturnalia,Chromogisaurus),(Bagualosaurus,(Jaklapalisaurus,(Nambalia,((Thecodontosaurus,Pantydraco),(Efraasia,(Plateosauravus,(Ruehleia,(((Unaysaurus,(Pradhania,Macrocollum)),((Sarahsaurus,((Ngwevu,((Coloradisaurus,(Glacialisaurus,Lufengosaurus)),(Xixipiosaurus,(Massospondylus_carinatus,(Adeopapposaurus,Leyesaurus))))),(Yunnanosaurus_huangi,((Seitaad,(Anchisaurus,((Irisosaurus,(Sefapanosaurus,(Yizhousaurus,(Aardonyx,(NMQR1551,(NMRQ3314,(Blikanasaurus,((Camelotia,((Meroktenos,(Ledumahadi,Kholumolumo)),(Lessemsaurus,(Ingentia,Antetonitrus)))),((Schleitheimia,(Isanosaurus,((Tazoudasaurus,Vulcanodon),((Shunosaurus,((Spinophorosaurus,(Omeisaurus,Mamenchisaurus)),(Cetiosaurus,Neosauropoda))),(Patagosaurus,Barapasaurus))))),(Pulanesaura,Gongxianosaurus)))))))))),(Mussaurus,Leonerasaurus)))),(Xingxiulong,(Jingshanosaurus,Chuxiongosaurus)))))),(Eucnemesaurus_fortis,(Riojasaurus,Eucnemesaurus_entaxonis)))),(Plateosaurus_gracilis,(Plateosaurus_ingens,Plateosaurus_engelhardti))))))))))))),(Buriolestes,Pampadromaeus))),(Staurikosaurus,Herrerasaurus))))))));

(Euparkeria,(Crurotarsi,(Marasuchus,(Silesaurus,(Ornithischia,(Agnosphitys,(((Guaibasaurus,(Neotheropoda,Chindesaurus)),((Panphagia,(Eoraptor,((Saturnalia,Chromogisaurus),(Bagualosaurus,(Jaklapalisaurus,(Nambalia,((Thecodontosaurus,Pantydraco),(Efraasia,(Plateosauravus,(Ruehleia,(((Unaysaurus,(Pradhania,Macrocollum)),((Sarahsaurus,((Ngwevu,(Xixipiosaurus,((Coloradisaurus,(Glacialisaurus,Lufengosaurus)),(Massospondylus_carinatus,(Adeopapposaurus,Leyesaurus))))),(Yunnanosaurus_huangi,((Seitaad,(Anchisaurus,((Irisosaurus,(Yizhousaurus,((Sefapanosaurus,Aardonyx),(NMQR1551,(NMRQ3314,(Blikanasaurus,((Camelotia,((Meroktenos,(Ledumahadi,Kholumolumo)),(Lessemsaurus,(Ingentia,Antetonitrus)))),(Pulanesaura,(Gongxianosaurus,(Schleitheimia,(Isanosaurus,((Tazoudasaurus,Vulcanodon),((Shunosaurus,(Spinophorosaurus,(Mamenchisaurus,(Omeisaurus,(Cetiosaurus,Neosauropoda))))),(Patagosaurus,Barapasaurus)))))))))))))),(Mussaurus,Leonerasaurus)))),(Xingxiulong,(Jingshanosaurus,Chuxiongosaurus)))))),(Eucnemesaurus_fortis,(Riojasaurus,Eucnemesaurus_entaxonis)))),(Plateosaurus_gracilis,(Plateosaurus_ingens,Plateosaurus_engelhardti))))))))))))),(Buriolestes,Pampadromaeus))),(Staurikosaurus,Herrerasaurus))))))));

(Euparkeria,(Crurotarsi,(Marasuchus,(Silesaurus,(Ornithischia,(Agnosphitys,(((Guaibasaurus,(Neotheropoda,Chindesaurus)),(Buriolestes,(Pampadromaeus,(Panphagia,(Eoraptor,((Saturnalia,Chromogisaurus),(Bagualosaurus,(Jaklapalisaurus,(Nambalia,((Thecodontosaurus,Pantydraco),(Efraasia,(Plateosauravus,(Ruehleia,(((Unaysaurus,(Pradhania,Macrocollum)),((Sarahsaurus,((Ngwevu,((Coloradisaurus,(Glacialisaurus,Lufengosaurus)),(Xixipiosaurus,(Massospondylus_carinatus,(Adeopapposaurus,Leyesaurus))))),(Yunnanosaurus_huangi,((Seitaad,(Anchisaurus,((Irisosaurus,(Yizhousaurus,((Sefapanosaurus,Aardonyx),(NMQR1551,(NMRQ3314,(Blikanasaurus,((Camelotia,((Meroktenos,(Ledumahadi,Kholumolumo)),(Lessemsaurus,(Ingentia,Antetonitrus)))),(Pulanesaura,(Gongxianosaurus,(Schleitheimia,(Isanosaurus,((Tazoudasaurus,Vulcanodon),((Shunosaurus,(Spinophorosaurus,(Mamenchisaurus,(Omeisaurus,(Cetiosaurus,Neosauropoda))))),(Patagosaurus,Barapasaurus)))))))))))))),(Mussaurus,Leonerasaurus)))),(Xingxiulong,(Jingshanosaurus,Chuxiongosaurus)))))),(Eucnemesaurus_fortis,(Riojasaurus,Eucnemesaurus_entaxonis)))),(Plateosaurus_gracilis,(Plateosaurus_ingens,Plateosaurus_engelhardti)))))))))))))))),(Staurikosaurus,Herrerasaurus))))))));

(Euparkeria,(Crurotarsi,(Marasuchus,(Silesaurus,(Ornithischia,(Agnosphitys,(((Guaibasaurus,(Neotheropoda,Chindesaurus)),(Buriolestes,(Pampadromaeus,(Panphagia,(Eoraptor,((Saturnalia,Chromogisaurus),(Bagualosaurus,(Jaklapalisaurus,(Nambalia,((Thecodontosaurus,Pantydraco),(Efraasia,(Plateosauravus,(Ruehleia,(((Unaysaurus,(Pradhania,Macrocollum)),((Sarahsaurus,((Ngwevu,(Xixipiosaurus,((Coloradisaurus,(Glacialisaurus,Lufengosaurus)),(Massospondylus_carinatus,(Adeopapposaurus,Leyesaurus))))),(Yunnanosaurus_huangi,((Seitaad,(Anchisaurus,((Irisosaurus,(Sefapanosaurus,(Yizhousaurus,(Aardonyx,(NMQR1551,(NMRQ3314,(Blikanasaurus,((Camelotia,((Meroktenos,(Ledumahadi,Kholumolumo)),(Lessemsaurus,(Ingentia,Antetonitrus)))),(Pulanesaura,(Gongxianosaurus,(Schleitheimia,(Isanosaurus,((Tazoudasaurus,Vulcanodon),((Shunosaurus,(Spinophorosaurus,(Mamenchisaurus,(Omeisaurus,(Cetiosaurus,Neosauropoda))))),(Patagosaurus,Barapasaurus))))))))))))))),(Mussaurus,Leonerasaurus)))),(Xingxiulong,(Jingshanosaurus,Chuxiongosaurus)))))),(Eucnemesaurus_fortis,(Riojasaurus,Eucnemesaurus_entaxonis)))),(Plateosaurus_gracilis,(Plateosaurus_ingens,Plateosaurus_engelhardti)))))))))))))))),(Staurikosaurus,Herrerasaurus))))))));

(Euparkeria,(Crurotarsi,(Marasuchus,(Silesaurus,(Ornithischia,(Agnosphitys,(((Guaibasaurus,(Neotheropoda,Chindesaurus)),(Buriolestes,(Pampadromaeus,(Panphagia,(Eoraptor,((Saturnalia,Chromogisaurus),(Bagualosaurus,(Jaklapalisaurus,(Nambalia,((Thecodontosaurus,Pantydraco),(Efraasia,(Plateosauravus,(Ruehleia,(((Unaysaurus,(Pradhania,Macrocollum)),((Sarahsaurus,((Ngwevu,(Xixipiosaurus,((Coloradisaurus,(Glacialisaurus,Lufengosaurus)),(Massospondylus_carinatus,(Adeopapposaurus,Leyesaurus))))),(Yunnanosaurus_huangi,((Seitaad,(Anchisaurus,((Irisosaurus,(Yizhousaurus,((Sefapanosaurus,Aardonyx),(NMQR1551,(NMRQ3314,(Blikanasaurus,((Camelotia,((Meroktenos,(Ledumahadi,Kholumolumo)),(Lessemsaurus,(Ingentia,Antetonitrus)))),((Schleitheimia,(Isanosaurus,((Tazoudasaurus,Vulcanodon),((Shunosaurus,(Spinophorosaurus,(Mamenchisaurus,(Omeisaurus,(Cetiosaurus,Neosauropoda))))),(Patagosaurus,Barapasaurus))))),(Pulanesaura,Gongxianosaurus))))))))),(Mussaurus,Leonerasaurus)))),(Xingxiulong,(Jingshanosaurus,Chuxiongosaurus)))))),(Eucnemesaurus_fortis,(Riojasaurus,Eucnemesaurus_entaxonis)))),(Plateosaurus_gracilis,(Plateosaurus_ingens,Plateosaurus_engelhardti)))))))))))))))),(Staurikosaurus,Herrerasaurus))))))));

(Euparkeria,(Crurotarsi,(Marasuchus,(Silesaurus,(Ornithischia,(Agnosphitys,(((Guaibasaurus,(Neotheropoda,Chindesaurus)),(Buriolestes,(Pampadromaeus,(Panphagia,(Eoraptor,((Saturnalia,Chromogisaurus),(Bagualosaurus,(Jaklapalisaurus,(Nambalia,((Thecodontosaurus,Pantydraco),(Efraasia,(Plateosauravus,(Ruehleia,(((Unaysaurus,(Pradhania,Macrocollum)),((Sarahsaurus,((Ngwevu,(Xixipiosaurus,((Coloradisaurus,(Glacialisaurus,Lufengosaurus)),(Massospondylus_carinatus,(Adeopapposaurus,Leyesaurus))))),(Yunnanosaurus_huangi,((Seitaad,(Anchisaurus,((Irisosaurus,(Yizhousaurus,((Sefapanosaurus,Aardonyx),(NMQR1551,(NMRQ3314,(Blikanasaurus,((Camelotia,((Meroktenos,(Ledumahadi,Kholumolumo)),(Lessemsaurus,(Ingentia,Antetonitrus)))),(Pulanesaura,(Gongxianosaurus,(Schleitheimia,(Isanosaurus,((Tazoudasaurus,Vulcanodon),((Shunosaurus,(Spinophorosaurus,(Omeisaurus,(Mamenchisaurus,(Cetiosaurus,Neosauropoda))))),(Patagosaurus,Barapasaurus)))))))))))))),(Mussaurus,Leonerasaurus)))),(Xingxiulong,(Jingshanosaurus,Chuxiongosaurus)))))),(Eucnemesaurus_fortis,(Riojasaurus,Eucnemesaurus_entaxonis)))),(Plateosaurus_gracilis,(Plateosaurus_ingens,Plateosaurus_engelhardti)))))))))))))))),(Staurikosaurus,Herrerasaurus))))))));

(Euparkeria,(Crurotarsi,(Marasuchus,(Silesaurus,(Ornithischia,(Agnosphitys,(((Guaibasaurus,(Neotheropoda,Chindesaurus)),(Buriolestes,(Pampadromaeus,(Panphagia,(Eoraptor,((Saturnalia,Chromogisaurus),(Bagualosaurus,(Jaklapalisaurus,(Nambalia,((Thecodontosaurus,Pantydraco),(Efraasia,(Plateosauravus,(Ruehleia,(((Unaysaurus,(Pradhania,Macrocollum)),((Sarahsaurus,((Ngwevu,(Xixipiosaurus,((Coloradisaurus,(Glacialisaurus,Lufengosaurus)),(Massospondylus_carinatus,(Adeopapposaurus,Leyesaurus))))),(Yunnanosaurus_huangi,((Seitaad,(Anchisaurus,((Irisosaurus,(Yizhousaurus,((Sefapanosaurus,Aardonyx),(NMQR1551,(NMRQ3314,(Blikanasaurus,((Camelotia,((Meroktenos,(Ledumahadi,Kholumolumo)),(Lessemsaurus,(Ingentia,Antetonitrus)))),(Pulanesaura,(Gongxianosaurus,(Schleitheimia,(Isanosaurus,((Tazoudasaurus,Vulcanodon),((Shunosaurus,((Spinophorosaurus,Omeisaurus),(Mamenchisaurus,(Cetiosaurus,Neosauropoda)))),(Patagosaurus,Barapasaurus)))))))))))))),(Mussaurus,Leonerasaurus)))),(Xingxiulong,(Jingshanosaurus,Chuxiongosaurus)))))),(Eucnemesaurus_fortis,(Riojasaurus,Eucnemesaurus_entaxonis)))),(Plateosaurus_gracilis,(Plateosaurus_ingens,Plateosaurus_engelhardti)))))))))))))))),(Staurikosaurus,Herrerasaurus))))))));

(Euparkeria,(Crurotarsi,(Marasuchus,(Silesaurus,(Ornithischia,(Agnosphitys,(((Guaibasaurus,(Neotheropoda,Chindesaurus)),(Buriolestes,(Pampadromaeus,(Panphagia,(Eoraptor,((Saturnalia,Chromogisaurus),(Bagualosaurus,(Jaklapalisaurus,(Nambalia,((Thecodontosaurus,Pantydraco),(Efraasia,(Plateosauravus,(Ruehleia,(((Unaysaurus,(Pradhania,Macrocollum)),((Sarahsaurus,((Ngwevu,(Xixipiosaurus,((Coloradisaurus,(Glacialisaurus,Lufengosaurus)),(Massospondylus_carinatus,(Adeopapposaurus,Leyesaurus))))),(Yunnanosaurus_huangi,((Seitaad,(Anchisaurus,((Irisosaurus,(Yizhousaurus,((Sefapanosaurus,Aardonyx),(NMQR1551,(NMRQ3314,(Blikanasaurus,((Camelotia,((Meroktenos,(Ledumahadi,Kholumolumo)),(Lessemsaurus,(Ingentia,Antetonitrus)))),(Pulanesaura,(Gongxianosaurus,(Schleitheimia,(Isanosaurus,((Tazoudasaurus,Vulcanodon),((Shunosaurus,((Spinophorosaurus,(Omeisaurus,Mamenchisaurus)),(Cetiosaurus,Neosauropoda))),(Patagosaurus,Barapasaurus)))))))))))))),(Mussaurus,Leonerasaurus)))),(Xingxiulong,(Jingshanosaurus,Chuxiongosaurus)))))),(Eucnemesaurus_fortis,(Riojasaurus,Eucnemesaurus_entaxonis)))),(Plateosaurus_gracilis,(Plateosaurus_ingens,Plateosaurus_engelhardti)))))))))))))))),(Staurikosaurus,Herrerasaurus))))))));

(Euparkeria,(Crurotarsi,(Marasuchus,(Silesaurus,(Ornithischia,((Agnosphitys,((Guaibasaurus,(Neotheropoda,Chindesaurus)),((Panphagia,(Eoraptor,((Saturnalia,Chromogisaurus),(Bagualosaurus,(Jaklapalisaurus,(Nambalia,((Thecodontosaurus,Pantydraco),(Efraasia,(Plateosauravus,(Ruehleia,(((Unaysaurus,(Pradhania,Macrocollum)),((Sarahsaurus,((Ngwevu,(Xixipiosaurus,((Coloradisaurus,(Glacialisaurus,Lufengosaurus)),(Massospondylus_carinatus,(Adeopapposaurus,Leyesaurus))))),(Yunnanosaurus_huangi,((Seitaad,(Anchisaurus,((Irisosaurus,(Yizhousaurus,((Sefapanosaurus,Aardonyx),(NMQR1551,(NMRQ3314,(Blikanasaurus,((Camelotia,((Meroktenos,(Ledumahadi,Kholumolumo)),(Lessemsaurus,(Ingentia,Antetonitrus)))),(Pulanesaura,(Gongxianosaurus,(Schleitheimia,(Isanosaurus,((Tazoudasaurus,Vulcanodon),((Shunosaurus,(Spinophorosaurus,(Mamenchisaurus,(Omeisaurus,(Cetiosaurus,Neosauropoda))))),(Patagosaurus,Barapasaurus)))))))))))))),(Mussaurus,Leonerasaurus)))),(Xingxiulong,(Jingshanosaurus,Chuxiongosaurus)))))),(Eucnemesaurus_fortis,(Riojasaurus,Eucnemesaurus_entaxonis)))),(Plateosaurus_gracilis,(Plateosaurus_ingens,Plateosaurus_engelhardti))))))))))))),(Buriolestes,Pampadromaeus)))),(Staurikosaurus,Herrerasaurus)))))));

(Euparkeria,(Crurotarsi,(Marasuchus,(Silesaurus,(Ornithischia,((Agnosphitys,((Guaibasaurus,(Neotheropoda,Chindesaurus)),(Buriolestes,((Eoraptor,((Saturnalia,Chromogisaurus),(Bagualosaurus,(Jaklapalisaurus,(Nambalia,((Thecodontosaurus,Pantydraco),(Efraasia,(Plateosauravus,(Ruehleia,(((Unaysaurus,(Pradhania,Macrocollum)),((Sarahsaurus,((Ngwevu,(Xixipiosaurus,((Coloradisaurus,(Glacialisaurus,Lufengosaurus)),(Massospondylus_carinatus,(Adeopapposaurus,Leyesaurus))))),(Yunnanosaurus_huangi,((Seitaad,(Anchisaurus,((Irisosaurus,(Yizhousaurus,((Sefapanosaurus,Aardonyx),(NMQR1551,(NMRQ3314,(Blikanasaurus,((Camelotia,((Meroktenos,(Ledumahadi,Kholumolumo)),(Lessemsaurus,(Ingentia,Antetonitrus)))),(Pulanesaura,(Gongxianosaurus,(Schleitheimia,(Isanosaurus,((Tazoudasaurus,Vulcanodon),((Shunosaurus,(Spinophorosaurus,(Mamenchisaurus,(Omeisaurus,(Cetiosaurus,Neosauropoda))))),(Patagosaurus,Barapasaurus)))))))))))))),(Mussaurus,Leonerasaurus)))),(Xingxiulong,(Jingshanosaurus,Chuxiongosaurus)))))),(Eucnemesaurus_fortis,(Riojasaurus,Eucnemesaurus_entaxonis)))),(Plateosaurus_gracilis,(Plateosaurus_ingens,Plateosaurus_engelhardti)))))))))))),(Panphagia,Pampadromaeus))))),(Staurikosaurus,Herrerasaurus)))))));

(Euparkeria,(Crurotarsi,(Marasuchus,(Silesaurus,(Ornithischia,((Agnosphitys,((Guaibasaurus,(Neotheropoda,Chindesaurus)),(Buriolestes,(Panphagia,(Eoraptor,((Saturnalia,Chromogisaurus),(Pampadromaeus,(Bagualosaurus,(Jaklapalisaurus,(Nambalia,((Thecodontosaurus,Pantydraco),(Efraasia,(Plateosauravus,(Ruehleia,(((Unaysaurus,(Pradhania,Macrocollum)),((Sarahsaurus,((Ngwevu,(Xixipiosaurus,((Coloradisaurus,(Glacialisaurus,Lufengosaurus)),(Massospondylus_carinatus,(Adeopapposaurus,Leyesaurus))))),(Yunnanosaurus_huangi,((Seitaad,(Anchisaurus,((Irisosaurus,(Yizhousaurus,((Sefapanosaurus,Aardonyx),(NMQR1551,(NMRQ3314,(Blikanasaurus,((Camelotia,((Meroktenos,(Ledumahadi,Kholumolumo)),(Lessemsaurus,(Ingentia,Antetonitrus)))),(Pulanesaura,(Gongxianosaurus,(Schleitheimia,(Isanosaurus,((Tazoudasaurus,Vulcanodon),((Shunosaurus,(Spinophorosaurus,(Mamenchisaurus,(Omeisaurus,(Cetiosaurus,Neosauropoda))))),(Patagosaurus,Barapasaurus)))))))))))))),(Mussaurus,Leonerasaurus)))),(Xingxiulong,(Jingshanosaurus,Chuxiongosaurus)))))),(Eucnemesaurus_fortis,(Riojasaurus,Eucnemesaurus_entaxonis)))),(Plateosaurus_gracilis,(Plateosaurus_ingens,Plateosaurus_engelhardti))))))))))))))))),(Staurikosaurus,Herrerasaurus)))))));

(Euparkeria,(Crurotarsi,(Marasuchus,(Silesaurus,(Ornithischia,((Agnosphitys,((Guaibasaurus,(Neotheropoda,Chindesaurus)),(Buriolestes,(Pampadromaeus,(Panphagia,(Eoraptor,((Saturnalia,Chromogisaurus),(Bagualosaurus,(Jaklapalisaurus,(Nambalia,((Thecodontosaurus,Pantydraco),(Efraasia,(Plateosauravus,(Ruehleia,(((Unaysaurus,(Pradhania,Macrocollum)),((Sarahsaurus,((Ngwevu,((Coloradisaurus,(Glacialisaurus,Lufengosaurus)),(Xixipiosaurus,(Massospondylus_carinatus,(Adeopapposaurus,Leyesaurus))))),(Yunnanosaurus_huangi,((Seitaad,(Anchisaurus,((Irisosaurus,(Yizhousaurus,((Sefapanosaurus,Aardonyx),(NMQR1551,(NMRQ3314,(Blikanasaurus,((Camelotia,((Meroktenos,(Ledumahadi,Kholumolumo)),(Lessemsaurus,(Ingentia,Antetonitrus)))),(Pulanesaura,(Gongxianosaurus,(Schleitheimia,(Isanosaurus,((Tazoudasaurus,Vulcanodon),((Shunosaurus,(Spinophorosaurus,(Mamenchisaurus,(Omeisaurus,(Cetiosaurus,Neosauropoda))))),(Patagosaurus,Barapasaurus)))))))))))))),(Mussaurus,Leonerasaurus)))),(Xingxiulong,(Jingshanosaurus,Chuxiongosaurus)))))),(Eucnemesaurus_fortis,(Riojasaurus,Eucnemesaurus_entaxonis)))),(Plateosaurus_gracilis,(Plateosaurus_ingens,Plateosaurus_engelhardti))))))))))))))))),(Staurikosaurus,Herrerasaurus)))))));

(Euparkeria,(Crurotarsi,(Marasuchus,(Silesaurus,(Ornithischia,((Agnosphitys,((Guaibasaurus,(Neotheropoda,Chindesaurus)),(Buriolestes,(Pampadromaeus,(Panphagia,(Eoraptor,((Saturnalia,Chromogisaurus),(Bagualosaurus,(Jaklapalisaurus,(Nambalia,((Thecodontosaurus,Pantydraco),(Efraasia,(Plateosauravus,(Ruehleia,(((Unaysaurus,(Pradhania,Macrocollum)),((Sarahsaurus,((Ngwevu,(Xixipiosaurus,((Coloradisaurus,(Glacialisaurus,Lufengosaurus)),(Massospondylus_carinatus,(Adeopapposaurus,Leyesaurus))))),(Yunnanosaurus_huangi,((Seitaad,(Anchisaurus,((Irisosaurus,(Yizhousaurus,((Sefapanosaurus,Aardonyx),(NMQR1551,(NMRQ3314,(Blikanasaurus,((Camelotia,((Meroktenos,(Ledumahadi,Kholumolumo)),(Lessemsaurus,(Ingentia,Antetonitrus)))),(Pulanesaura,(Gongxianosaurus,(Schleitheimia,(Isanosaurus,((Tazoudasaurus,Vulcanodon),((Shunosaurus,(Spinophorosaurus,(Omeisaurus,(Mamenchisaurus,(Cetiosaurus,Neosauropoda))))),(Patagosaurus,Barapasaurus)))))))))))))),(Mussaurus,Leonerasaurus)))),(Xingxiulong,(Jingshanosaurus,Chuxiongosaurus)))))),(Eucnemesaurus_fortis,(Riojasaurus,Eucnemesaurus_entaxonis)))),(Plateosaurus_gracilis,(Plateosaurus_ingens,Plateosaurus_engelhardti))))))))))))))))),(Staurikosaurus,Herrerasaurus)))))));

(Euparkeria,(Crurotarsi,(Marasuchus,(Silesaurus,(Ornithischia,((Agnosphitys,((Guaibasaurus,(Neotheropoda,Chindesaurus)),(Buriolestes,(Pampadromaeus,(Panphagia,(Eoraptor,((Saturnalia,Chromogisaurus),(Bagualosaurus,(Jaklapalisaurus,(Nambalia,((Thecodontosaurus,Pantydraco),(Efraasia,(Plateosauravus,(Ruehleia,(((Unaysaurus,(Pradhania,Macrocollum)),((Sarahsaurus,((Ngwevu,(Xixipiosaurus,((Coloradisaurus,(Glacialisaurus,Lufengosaurus)),(Massospondylus_carinatus,(Adeopapposaurus,Leyesaurus))))),(Yunnanosaurus_huangi,((Seitaad,(Anchisaurus,((Irisosaurus,(Yizhousaurus,((Sefapanosaurus,Aardonyx),(NMQR1551,(NMRQ3314,(Blikanasaurus,((Camelotia,((Meroktenos,(Ledumahadi,Kholumolumo)),(Lessemsaurus,(Ingentia,Antetonitrus)))),(Pulanesaura,(Gongxianosaurus,(Schleitheimia,(Isanosaurus,((Tazoudasaurus,Vulcanodon),((Shunosaurus,((Spinophorosaurus,Omeisaurus),(Mamenchisaurus,(Cetiosaurus,Neosauropoda)))),(Patagosaurus,Barapasaurus)))))))))))))),(Mussaurus,Leonerasaurus)))),(Xingxiulong,(Jingshanosaurus,Chuxiongosaurus)))))),(Eucnemesaurus_fortis,(Riojasaurus,Eucnemesaurus_entaxonis)))),(Plateosaurus_gracilis,(Plateosaurus_ingens,Plateosaurus_engelhardti))))))))))))))))),(Staurikosaurus,Herrerasaurus)))))));

(Euparkeria,(Crurotarsi,(Marasuchus,(Silesaurus,(Ornithischia,((Agnosphitys,((Guaibasaurus,(Neotheropoda,Chindesaurus)),((Eoraptor,((Saturnalia,Chromogisaurus),(Bagualosaurus,(Jaklapalisaurus,(Nambalia,((Thecodontosaurus,Pantydraco),(Efraasia,(Plateosauravus,(Ruehleia,(((Unaysaurus,(Pradhania,Macrocollum)),((Sarahsaurus,((Ngwevu,(Xixipiosaurus,((Coloradisaurus,(Glacialisaurus,Lufengosaurus)),(Massospondylus_carinatus,(Adeopapposaurus,Leyesaurus))))),(Yunnanosaurus_huangi,((Seitaad,(Anchisaurus,((Irisosaurus,(Yizhousaurus,((Sefapanosaurus,Aardonyx),(NMQR1551,(NMRQ3314,(Blikanasaurus,((Camelotia,((Meroktenos,(Ledumahadi,Kholumolumo)),(Lessemsaurus,(Ingentia,Antetonitrus)))),(Pulanesaura,(Gongxianosaurus,(Schleitheimia,(Isanosaurus,((Tazoudasaurus,Vulcanodon),((Shunosaurus,(Spinophorosaurus,(Mamenchisaurus,(Omeisaurus,(Cetiosaurus,Neosauropoda))))),(Patagosaurus,Barapasaurus)))))))))))))),(Mussaurus,Leonerasaurus)))),(Xingxiulong,(Jingshanosaurus,Chuxiongosaurus)))))),(Eucnemesaurus_fortis,(Riojasaurus,Eucnemesaurus_entaxonis)))),(Plateosaurus_gracilis,(Plateosaurus_ingens,Plateosaurus_engelhardti)))))))))))),(Buriolestes,(Panphagia,Pampadromaeus))))),(Staurikosaurus,Herrerasaurus)))))));

(Euparkeria,(Crurotarsi,(Marasuchus,(Silesaurus,(Agnosphitys,(Ornithischia,(((Guaibasaurus,(Neotheropoda,Chindesaurus)),((Panphagia,(Eoraptor,((Saturnalia,Chromogisaurus),(Bagualosaurus,(Jaklapalisaurus,(Nambalia,((Thecodontosaurus,Pantydraco),(Efraasia,(Plateosauravus,(Ruehleia,(((Unaysaurus,(Pradhania,Macrocollum)),((Sarahsaurus,((Ngwevu,((Coloradisaurus,(Glacialisaurus,Lufengosaurus)),(Xixipiosaurus,(Massospondylus_carinatus,(Adeopapposaurus,Leyesaurus))))),(Yunnanosaurus_huangi,((Seitaad,(Anchisaurus,((Irisosaurus,(Yizhousaurus,((Sefapanosaurus,Aardonyx),(NMQR1551,(NMRQ3314,(Blikanasaurus,((Camelotia,((Meroktenos,(Ledumahadi,Kholumolumo)),(Lessemsaurus,(Ingentia,Antetonitrus)))),(Pulanesaura,(Gongxianosaurus,(Schleitheimia,(Isanosaurus,((Tazoudasaurus,Vulcanodon),((Shunosaurus,(Spinophorosaurus,(Mamenchisaurus,(Omeisaurus,(Cetiosaurus,Neosauropoda))))),(Patagosaurus,Barapasaurus)))))))))))))),(Mussaurus,Leonerasaurus)))),(Xingxiulong,(Jingshanosaurus,Chuxiongosaurus)))))),(Eucnemesaurus_fortis,(Riojasaurus,Eucnemesaurus_entaxonis)))),(Plateosaurus_gracilis,(Plateosaurus_ingens,Plateosaurus_engelhardti))))))))))))),(Buriolestes,Pampadromaeus))),(Staurikosaurus,Herrerasaurus))))))));

(Euparkeria,(Crurotarsi,(Marasuchus,(Silesaurus,(Agnosphitys,(Ornithischia,(((Guaibasaurus,(Neotheropoda,Chindesaurus)),((Panphagia,(Eoraptor,((Saturnalia,Chromogisaurus),(Bagualosaurus,(Jaklapalisaurus,(Nambalia,((Thecodontosaurus,Pantydraco),(Efraasia,(Plateosauravus,(Ruehleia,(((Unaysaurus,(Pradhania,Macrocollum)),((Sarahsaurus,((Ngwevu,(Xixipiosaurus,((Coloradisaurus,(Glacialisaurus,Lufengosaurus)),(Massospondylus_carinatus,(Adeopapposaurus,Leyesaurus))))),(Yunnanosaurus_huangi,((Seitaad,(Anchisaurus,((Irisosaurus,(Yizhousaurus,((Sefapanosaurus,Aardonyx),(NMQR1551,(NMRQ3314,(Blikanasaurus,((Camelotia,((Meroktenos,(Ledumahadi,Kholumolumo)),(Lessemsaurus,(Ingentia,Antetonitrus)))),((Schleitheimia,(Isanosaurus,((Tazoudasaurus,Vulcanodon),((Shunosaurus,(Spinophorosaurus,(Mamenchisaurus,(Omeisaurus,(Cetiosaurus,Neosauropoda))))),(Patagosaurus,Barapasaurus))))),(Pulanesaura,Gongxianosaurus))))))))),(Mussaurus,Leonerasaurus)))),(Xingxiulong,(Jingshanosaurus,Chuxiongosaurus)))))),(Eucnemesaurus_fortis,(Riojasaurus,Eucnemesaurus_entaxonis)))),(Plateosaurus_gracilis,(Plateosaurus_ingens,Plateosaurus_engelhardti))))))))))))),(Buriolestes,Pampadromaeus))),(Staurikosaurus,Herrerasaurus))))))));

(Euparkeria,(Crurotarsi,(Marasuchus,(Silesaurus,(Agnosphitys,(Ornithischia,(((Guaibasaurus,(Neotheropoda,Chindesaurus)),(Buriolestes,(Pampadromaeus,(Panphagia,(Eoraptor,((Saturnalia,Chromogisaurus),(Bagualosaurus,(Jaklapalisaurus,(Nambalia,((Thecodontosaurus,Pantydraco),(Efraasia,(Plateosauravus,(Ruehleia,(((Unaysaurus,(Pradhania,Macrocollum)),((Sarahsaurus,((Ngwevu,((Coloradisaurus,(Glacialisaurus,Lufengosaurus)),(Xixipiosaurus,(Massospondylus_carinatus,(Adeopapposaurus,Leyesaurus))))),(Yunnanosaurus_huangi,((Seitaad,(Anchisaurus,((Irisosaurus,(Sefapanosaurus,(Yizhousaurus,(Aardonyx,(NMQR1551,(NMRQ3314,(Blikanasaurus,((Camelotia,((Meroktenos,(Ledumahadi,Kholumolumo)),(Lessemsaurus,(Ingentia,Antetonitrus)))),(Pulanesaura,(Gongxianosaurus,(Schleitheimia,(Isanosaurus,((Tazoudasaurus,Vulcanodon),((Shunosaurus,(Spinophorosaurus,(Mamenchisaurus,(Omeisaurus,(Cetiosaurus,Neosauropoda))))),(Patagosaurus,Barapasaurus))))))))))))))),(Mussaurus,Leonerasaurus)))),(Xingxiulong,(Jingshanosaurus,Chuxiongosaurus)))))),(Eucnemesaurus_fortis,(Riojasaurus,Eucnemesaurus_entaxonis)))),(Plateosaurus_gracilis,(Plateosaurus_ingens,Plateosaurus_engelhardti)))))))))))))))),(Staurikosaurus,Herrerasaurus))))))));

(Euparkeria,(Crurotarsi,(Marasuchus,(Silesaurus,(Agnosphitys,(Ornithischia,(((Guaibasaurus,(Neotheropoda,Chindesaurus)),(Buriolestes,(Pampadromaeus,(Panphagia,(Eoraptor,((Saturnalia,Chromogisaurus),(Bagualosaurus,(Jaklapalisaurus,(Nambalia,((Thecodontosaurus,Pantydraco),(Efraasia,(Plateosauravus,(Ruehleia,(((Unaysaurus,(Pradhania,Macrocollum)),((Sarahsaurus,((Ngwevu,((Coloradisaurus,(Glacialisaurus,Lufengosaurus)),(Xixipiosaurus,(Massospondylus_carinatus,(Adeopapposaurus,Leyesaurus))))),(Yunnanosaurus_huangi,((Seitaad,(Anchisaurus,((Irisosaurus,(Yizhousaurus,((Sefapanosaurus,Aardonyx),(NMQR1551,(NMRQ3314,(Blikanasaurus,((Camelotia,((Meroktenos,(Ledumahadi,Kholumolumo)),(Lessemsaurus,(Ingentia,Antetonitrus)))),((Schleitheimia,(Isanosaurus,((Tazoudasaurus,Vulcanodon),((Shunosaurus,(Spinophorosaurus,(Mamenchisaurus,(Omeisaurus,(Cetiosaurus,Neosauropoda))))),(Patagosaurus,Barapasaurus))))),(Pulanesaura,Gongxianosaurus))))))))),(Mussaurus,Leonerasaurus)))),(Xingxiulong,(Jingshanosaurus,Chuxiongosaurus)))))),(Eucnemesaurus_fortis,(Riojasaurus,Eucnemesaurus_entaxonis)))),(Plateosaurus_gracilis,(Plateosaurus_ingens,Plateosaurus_engelhardti)))))))))))))))),(Staurikosaurus,Herrerasaurus))))))));

(Euparkeria,(Crurotarsi,(Marasuchus,(Silesaurus,(Agnosphitys,(Ornithischia,(((Guaibasaurus,(Neotheropoda,Chindesaurus)),(Buriolestes,(Pampadromaeus,(Panphagia,(Eoraptor,((Saturnalia,Chromogisaurus),(Bagualosaurus,(Jaklapalisaurus,(Nambalia,((Thecodontosaurus,Pantydraco),(Efraasia,(Plateosauravus,(Ruehleia,(((Unaysaurus,(Pradhania,Macrocollum)),((Sarahsaurus,((Ngwevu,((Coloradisaurus,(Glacialisaurus,Lufengosaurus)),(Xixipiosaurus,(Massospondylus_carinatus,(Adeopapposaurus,Leyesaurus))))),(Yunnanosaurus_huangi,((Seitaad,(Anchisaurus,((Irisosaurus,(Yizhousaurus,((Sefapanosaurus,Aardonyx),(NMQR1551,(NMRQ3314,(Blikanasaurus,((Camelotia,((Meroktenos,(Ledumahadi,Kholumolumo)),(Lessemsaurus,(Ingentia,Antetonitrus)))),(Pulanesaura,(Gongxianosaurus,(Schleitheimia,(Isanosaurus,((Tazoudasaurus,Vulcanodon),((Shunosaurus,(Spinophorosaurus,(Omeisaurus,(Mamenchisaurus,(Cetiosaurus,Neosauropoda))))),(Patagosaurus,Barapasaurus)))))))))))))),(Mussaurus,Leonerasaurus)))),(Xingxiulong,(Jingshanosaurus,Chuxiongosaurus)))))),(Eucnemesaurus_fortis,(Riojasaurus,Eucnemesaurus_entaxonis)))),(Plateosaurus_gracilis,(Plateosaurus_ingens,Plateosaurus_engelhardti)))))))))))))))),(Staurikosaurus,Herrerasaurus))))))));

(Euparkeria,(Crurotarsi,(Marasuchus,(Silesaurus,(Agnosphitys,(Ornithischia,(((Guaibasaurus,(Neotheropoda,Chindesaurus)),(Buriolestes,(Pampadromaeus,(Panphagia,(Eoraptor,((Saturnalia,Chromogisaurus),(Bagualosaurus,(Jaklapalisaurus,(Nambalia,((Thecodontosaurus,Pantydraco),(Efraasia,(Plateosauravus,(Ruehleia,(((Unaysaurus,(Pradhania,Macrocollum)),((Sarahsaurus,((Ngwevu,((Coloradisaurus,(Glacialisaurus,Lufengosaurus)),(Xixipiosaurus,(Massospondylus_carinatus,(Adeopapposaurus,Leyesaurus))))),(Yunnanosaurus_huangi,((Seitaad,(Anchisaurus,((Irisosaurus,(Yizhousaurus,((Sefapanosaurus,Aardonyx),(NMQR1551,(NMRQ3314,(Blikanasaurus,((Camelotia,((Meroktenos,(Ledumahadi,Kholumolumo)),(Lessemsaurus,(Ingentia,Antetonitrus)))),(Pulanesaura,(Gongxianosaurus,(Schleitheimia,(Isanosaurus,((Tazoudasaurus,Vulcanodon),((Shunosaurus,((Spinophorosaurus,Omeisaurus),(Mamenchisaurus,(Cetiosaurus,Neosauropoda)))),(Patagosaurus,Barapasaurus)))))))))))))),(Mussaurus,Leonerasaurus)))),(Xingxiulong,(Jingshanosaurus,Chuxiongosaurus)))))),(Eucnemesaurus_fortis,(Riojasaurus,Eucnemesaurus_entaxonis)))),(Plateosaurus_gracilis,(Plateosaurus_ingens,Plateosaurus_engelhardti)))))))))))))))),(Staurikosaurus,Herrerasaurus))))))));

(Euparkeria,(Crurotarsi,(Marasuchus,(Silesaurus,(Agnosphitys,(Ornithischia,(((Guaibasaurus,(Neotheropoda,Chindesaurus)),(Buriolestes,(Pampadromaeus,(Panphagia,(Eoraptor,((Saturnalia,Chromogisaurus),(Bagualosaurus,(Jaklapalisaurus,(Nambalia,((Thecodontosaurus,Pantydraco),(Efraasia,(Plateosauravus,(Ruehleia,(((Unaysaurus,(Pradhania,Macrocollum)),((Sarahsaurus,((Ngwevu,((Coloradisaurus,(Glacialisaurus,Lufengosaurus)),(Xixipiosaurus,(Massospondylus_carinatus,(Adeopapposaurus,Leyesaurus))))),(Yunnanosaurus_huangi,((Seitaad,(Anchisaurus,((Irisosaurus,(Yizhousaurus,((Sefapanosaurus,Aardonyx),(NMQR1551,(NMRQ3314,(Blikanasaurus,((Camelotia,((Meroktenos,(Ledumahadi,Kholumolumo)),(Lessemsaurus,(Ingentia,Antetonitrus)))),(Pulanesaura,(Gongxianosaurus,(Schleitheimia,(Isanosaurus,((Tazoudasaurus,Vulcanodon),((Shunosaurus,((Spinophorosaurus,(Omeisaurus,Mamenchisaurus)),(Cetiosaurus,Neosauropoda))),(Patagosaurus,Barapasaurus)))))))))))))),(Mussaurus,Leonerasaurus)))),(Xingxiulong,(Jingshanosaurus,Chuxiongosaurus)))))),(Eucnemesaurus_fortis,(Riojasaurus,Eucnemesaurus_entaxonis)))),(Plateosaurus_gracilis,(Plateosaurus_ingens,Plateosaurus_engelhardti)))))))))))))))),(Staurikosaurus,Herrerasaurus))))))));

(Euparkeria,(Crurotarsi,(Marasuchus,(Silesaurus,(Agnosphitys,(Ornithischia,(((Guaibasaurus,(Neotheropoda,Chindesaurus)),(Buriolestes,(Pampadromaeus,(Panphagia,(Eoraptor,((Saturnalia,Chromogisaurus),(Bagualosaurus,(Jaklapalisaurus,(Nambalia,((Thecodontosaurus,Pantydraco),(Efraasia,(Plateosauravus,(Ruehleia,(((Unaysaurus,(Pradhania,Macrocollum)),((Sarahsaurus,((Ngwevu,(Xixipiosaurus,((Coloradisaurus,(Glacialisaurus,Lufengosaurus)),(Massospondylus_carinatus,(Adeopapposaurus,Leyesaurus))))),(Yunnanosaurus_huangi,((Seitaad,(Anchisaurus,((Irisosaurus,(Yizhousaurus,((Sefapanosaurus,Aardonyx),(NMQR1551,(NMRQ3314,(Blikanasaurus,((Camelotia,((Meroktenos,(Ledumahadi,Kholumolumo)),(Lessemsaurus,(Ingentia,Antetonitrus)))),((Schleitheimia,(Isanosaurus,((Tazoudasaurus,Vulcanodon),((Shunosaurus,(Spinophorosaurus,(Omeisaurus,(Mamenchisaurus,(Cetiosaurus,Neosauropoda))))),(Patagosaurus,Barapasaurus))))),(Pulanesaura,Gongxianosaurus))))))))),(Mussaurus,Leonerasaurus)))),(Xingxiulong,(Jingshanosaurus,Chuxiongosaurus)))))),(Eucnemesaurus_fortis,(Riojasaurus,Eucnemesaurus_entaxonis)))),(Plateosaurus_gracilis,(Plateosaurus_ingens,Plateosaurus_engelhardti)))))))))))))))),(Staurikosaurus,Herrerasaurus))))))));

(Euparkeria,(Crurotarsi,(Marasuchus,(Silesaurus,(Agnosphitys,(Ornithischia,(((Guaibasaurus,(Neotheropoda,Chindesaurus)),(Buriolestes,(Pampadromaeus,(Panphagia,(Eoraptor,((Saturnalia,Chromogisaurus),(Bagualosaurus,(Jaklapalisaurus,(Nambalia,((Thecodontosaurus,Pantydraco),(Efraasia,(Plateosauravus,(Ruehleia,(((Unaysaurus,(Pradhania,Macrocollum)),((Sarahsaurus,((Ngwevu,(Xixipiosaurus,((Coloradisaurus,(Glacialisaurus,Lufengosaurus)),(Massospondylus_carinatus,(Adeopapposaurus,Leyesaurus))))),(Yunnanosaurus_huangi,((Seitaad,(Anchisaurus,((Irisosaurus,(Yizhousaurus,((Sefapanosaurus,Aardonyx),(NMQR1551,(NMRQ3314,(Blikanasaurus,((Camelotia,((Meroktenos,(Ledumahadi,Kholumolumo)),(Lessemsaurus,(Ingentia,Antetonitrus)))),((Schleitheimia,(Isanosaurus,((Tazoudasaurus,Vulcanodon),((Shunosaurus,((Spinophorosaurus,Omeisaurus),(Mamenchisaurus,(Cetiosaurus,Neosauropoda)))),(Patagosaurus,Barapasaurus))))),(Pulanesaura,Gongxianosaurus))))))))),(Mussaurus,Leonerasaurus)))),(Xingxiulong,(Jingshanosaurus,Chuxiongosaurus)))))),(Eucnemesaurus_fortis,(Riojasaurus,Eucnemesaurus_entaxonis)))),(Plateosaurus_gracilis,(Plateosaurus_ingens,Plateosaurus_engelhardti)))))))))))))))),(Staurikosaurus,Herrerasaurus))))))));

(Euparkeria,(Crurotarsi,(Marasuchus,(Silesaurus,(Agnosphitys,(Ornithischia,(((Guaibasaurus,(Neotheropoda,Chindesaurus)),(Buriolestes,(Pampadromaeus,(Panphagia,(Eoraptor,((Saturnalia,Chromogisaurus),(Bagualosaurus,(Jaklapalisaurus,(Nambalia,((Thecodontosaurus,Pantydraco),(Efraasia,(Plateosauravus,(Ruehleia,(((Unaysaurus,(Pradhania,Macrocollum)),((Sarahsaurus,((Ngwevu,(Xixipiosaurus,((Coloradisaurus,(Glacialisaurus,Lufengosaurus)),(Massospondylus_carinatus,(Adeopapposaurus,Leyesaurus))))),(Yunnanosaurus_huangi,((Seitaad,(Anchisaurus,((Irisosaurus,(Yizhousaurus,((Sefapanosaurus,Aardonyx),(NMQR1551,(NMRQ3314,((Camelotia,((Meroktenos,(Ledumahadi,Kholumolumo)),(Lessemsaurus,(Blikanasaurus,(Ingentia,Antetonitrus))))),(Pulanesaura,(Gongxianosaurus,(Schleitheimia,(Isanosaurus,((Tazoudasaurus,Vulcanodon),((Shunosaurus,(Spinophorosaurus,(Omeisaurus,(Mamenchisaurus,(Cetiosaurus,Neosauropoda))))),(Patagosaurus,Barapasaurus))))))))))))),(Mussaurus,Leonerasaurus)))),(Xingxiulong,(Jingshanosaurus,Chuxiongosaurus)))))),(Eucnemesaurus_fortis,(Riojasaurus,Eucnemesaurus_entaxonis)))),(Plateosaurus_gracilis,(Plateosaurus_ingens,Plateosaurus_engelhardti)))))))))))))))),(Staurikosaurus,Herrerasaurus))))))));

(Euparkeria,(Crurotarsi,(Marasuchus,(Silesaurus,(Ornithischia,(Agnosphitys,(((Guaibasaurus,(Neotheropoda,Chindesaurus)),((Panphagia,(Eoraptor,((Saturnalia,Chromogisaurus),(Bagualosaurus,(Jaklapalisaurus,(Nambalia,((Thecodontosaurus,Pantydraco),(Efraasia,(Plateosauravus,(Ruehleia,(((Unaysaurus,(Pradhania,Macrocollum)),((Sarahsaurus,((Ngwevu,(Xixipiosaurus,((Coloradisaurus,(Glacialisaurus,Lufengosaurus)),(Massospondylus_carinatus,(Adeopapposaurus,Leyesaurus))))),(Yunnanosaurus_huangi,((Seitaad,(Anchisaurus,((Yizhousaurus,(Sefapanosaurus,Aardonyx)),((Mussaurus,Leonerasaurus),(Irisosaurus,(NMQR1551,(NMRQ3314,(Blikanasaurus,((Camelotia,((Meroktenos,(Ledumahadi,Kholumolumo)),(Lessemsaurus,(Ingentia,Antetonitrus)))),(Pulanesaura,(Gongxianosaurus,(Schleitheimia,(Isanosaurus,((Tazoudasaurus,Vulcanodon),((Shunosaurus,(Spinophorosaurus,(Mamenchisaurus,(Omeisaurus,(Cetiosaurus,Neosauropoda))))),(Patagosaurus,Barapasaurus)))))))))))))))),(Xingxiulong,(Jingshanosaurus,Chuxiongosaurus)))))),(Eucnemesaurus_fortis,(Riojasaurus,Eucnemesaurus_entaxonis)))),(Plateosaurus_gracilis,(Plateosaurus_ingens,Plateosaurus_engelhardti))))))))))))),(Buriolestes,Pampadromaeus))),(Staurikosaurus,Herrerasaurus))))))));

(Euparkeria,(Crurotarsi,(Marasuchus,(Silesaurus,(Ornithischia,(Agnosphitys,(((Guaibasaurus,(Neotheropoda,Chindesaurus)),(Buriolestes,(Pampadromaeus,(Panphagia,(Eoraptor,((Saturnalia,Chromogisaurus),(Bagualosaurus,(Jaklapalisaurus,(Nambalia,((Thecodontosaurus,Pantydraco),(Efraasia,(Plateosauravus,(Ruehleia,(((Unaysaurus,(Pradhania,Macrocollum)),((Sarahsaurus,((Ngwevu,((Coloradisaurus,(Glacialisaurus,Lufengosaurus)),(Xixipiosaurus,(Massospondylus_carinatus,(Adeopapposaurus,Leyesaurus))))),(Yunnanosaurus_huangi,((Seitaad,(Anchisaurus,((Yizhousaurus,(Sefapanosaurus,Aardonyx)),((Mussaurus,Leonerasaurus),(Irisosaurus,(NMQR1551,(NMRQ3314,(Blikanasaurus,((Camelotia,((Meroktenos,(Ledumahadi,Kholumolumo)),(Lessemsaurus,(Ingentia,Antetonitrus)))),(Pulanesaura,(Gongxianosaurus,(Schleitheimia,(Isanosaurus,((Tazoudasaurus,Vulcanodon),((Shunosaurus,(Spinophorosaurus,(Mamenchisaurus,(Omeisaurus,(Cetiosaurus,Neosauropoda))))),(Patagosaurus,Barapasaurus)))))))))))))))),(Xingxiulong,(Jingshanosaurus,Chuxiongosaurus)))))),(Eucnemesaurus_fortis,(Riojasaurus,Eucnemesaurus_entaxonis)))),(Plateosaurus_gracilis,(Plateosaurus_ingens,Plateosaurus_engelhardti)))))))))))))))),(Staurikosaurus,Herrerasaurus))))))));

(Euparkeria,(Crurotarsi,(Marasuchus,(Silesaurus,(Ornithischia,(Agnosphitys,(((Guaibasaurus,(Neotheropoda,Chindesaurus)),(Buriolestes,(Pampadromaeus,(Panphagia,(Eoraptor,((Saturnalia,Chromogisaurus),(Bagualosaurus,(Jaklapalisaurus,(Nambalia,((Thecodontosaurus,Pantydraco),(Efraasia,(Plateosauravus,(Ruehleia,(((Unaysaurus,(Pradhania,Macrocollum)),((Sarahsaurus,((Ngwevu,(Xixipiosaurus,((Coloradisaurus,(Glacialisaurus,Lufengosaurus)),(Massospondylus_carinatus,(Adeopapposaurus,Leyesaurus))))),(Yunnanosaurus_huangi,((Seitaad,(Anchisaurus,((Yizhousaurus,Aardonyx),((Leonerasaurus,(Mussaurus,Sefapanosaurus)),(Irisosaurus,(NMQR1551,(NMRQ3314,(Blikanasaurus,((Camelotia,((Meroktenos,(Ledumahadi,Kholumolumo)),(Lessemsaurus,(Ingentia,Antetonitrus)))),(Pulanesaura,(Gongxianosaurus,(Schleitheimia,(Isanosaurus,((Tazoudasaurus,Vulcanodon),((Shunosaurus,(Spinophorosaurus,(Mamenchisaurus,(Omeisaurus,(Cetiosaurus,Neosauropoda))))),(Patagosaurus,Barapasaurus)))))))))))))))),(Xingxiulong,(Jingshanosaurus,Chuxiongosaurus)))))),(Eucnemesaurus_fortis,(Riojasaurus,Eucnemesaurus_entaxonis)))),(Plateosaurus_gracilis,(Plateosaurus_ingens,Plateosaurus_engelhardti)))))))))))))))),(Staurikosaurus,Herrerasaurus))))))));

(Euparkeria,(Crurotarsi,(Marasuchus,(Silesaurus,(Ornithischia,(Agnosphitys,(((Guaibasaurus,(Neotheropoda,Chindesaurus)),(Buriolestes,(Pampadromaeus,(Panphagia,(Eoraptor,((Saturnalia,Chromogisaurus),(Bagualosaurus,(Jaklapalisaurus,(Nambalia,((Thecodontosaurus,Pantydraco),(Efraasia,(Plateosauravus,(Ruehleia,(((Unaysaurus,(Pradhania,Macrocollum)),((Sarahsaurus,((Ngwevu,(Xixipiosaurus,((Coloradisaurus,(Glacialisaurus,Lufengosaurus)),(Massospondylus_carinatus,(Adeopapposaurus,Leyesaurus))))),(Yunnanosaurus_huangi,((Seitaad,(Anchisaurus,((Yizhousaurus,(Sefapanosaurus,Aardonyx)),((Mussaurus,Leonerasaurus),(Irisosaurus,(NMQR1551,(NMRQ3314,(Blikanasaurus,((Camelotia,((Meroktenos,(Ledumahadi,Kholumolumo)),(Lessemsaurus,(Ingentia,Antetonitrus)))),((Schleitheimia,(Isanosaurus,((Tazoudasaurus,Vulcanodon),((Shunosaurus,(Spinophorosaurus,(Mamenchisaurus,(Omeisaurus,(Cetiosaurus,Neosauropoda))))),(Patagosaurus,Barapasaurus))))),(Pulanesaura,Gongxianosaurus))))))))))),(Xingxiulong,(Jingshanosaurus,Chuxiongosaurus)))))),(Eucnemesaurus_fortis,(Riojasaurus,Eucnemesaurus_entaxonis)))),(Plateosaurus_gracilis,(Plateosaurus_ingens,Plateosaurus_engelhardti)))))))))))))))),(Staurikosaurus,Herrerasaurus))))))));

(Euparkeria,(Crurotarsi,(Marasuchus,(Silesaurus,(Ornithischia,(Agnosphitys,(((Guaibasaurus,(Neotheropoda,Chindesaurus)),(Buriolestes,(Pampadromaeus,(Panphagia,(Eoraptor,((Saturnalia,Chromogisaurus),(Bagualosaurus,(Jaklapalisaurus,(Nambalia,((Thecodontosaurus,Pantydraco),(Efraasia,(Plateosauravus,(Ruehleia,(((Unaysaurus,(Pradhania,Macrocollum)),((Sarahsaurus,((Ngwevu,(Xixipiosaurus,((Coloradisaurus,(Glacialisaurus,Lufengosaurus)),(Massospondylus_carinatus,(Adeopapposaurus,Leyesaurus))))),(Yunnanosaurus_huangi,((Seitaad,(Anchisaurus,((Yizhousaurus,(Sefapanosaurus,Aardonyx)),((Mussaurus,Leonerasaurus),((Irisosaurus,(NMRQ3314,NMQR1551)),(Blikanasaurus,((Camelotia,((Meroktenos,(Ledumahadi,Kholumolumo)),(Lessemsaurus,(Ingentia,Antetonitrus)))),(Pulanesaura,(Gongxianosaurus,(Schleitheimia,(Isanosaurus,((Tazoudasaurus,Vulcanodon),((Shunosaurus,(Spinophorosaurus,(Mamenchisaurus,(Omeisaurus,(Cetiosaurus,Neosauropoda))))),(Patagosaurus,Barapasaurus)))))))))))))),(Xingxiulong,(Jingshanosaurus,Chuxiongosaurus)))))),(Eucnemesaurus_fortis,(Riojasaurus,Eucnemesaurus_entaxonis)))),(Plateosaurus_gracilis,(Plateosaurus_ingens,Plateosaurus_engelhardti)))))))))))))))),(Staurikosaurus,Herrerasaurus))))))));

(Euparkeria,(Crurotarsi,(Marasuchus,(Silesaurus,(Ornithischia,((Agnosphitys,((Guaibasaurus,(Neotheropoda,Chindesaurus)),((Panphagia,(Eoraptor,((Saturnalia,Chromogisaurus),(Bagualosaurus,(Jaklapalisaurus,(Nambalia,((Thecodontosaurus,Pantydraco),(Efraasia,(Plateosauravus,(Ruehleia,(((Unaysaurus,(Pradhania,Macrocollum)),((Sarahsaurus,((Ngwevu,(Xixipiosaurus,((Coloradisaurus,(Glacialisaurus,Lufengosaurus)),(Massospondylus_carinatus,(Adeopapposaurus,Leyesaurus))))),(Yunnanosaurus_huangi,((Seitaad,(Anchisaurus,((Yizhousaurus,(Sefapanosaurus,Aardonyx)),((Mussaurus,Leonerasaurus),(Irisosaurus,(NMQR1551,(NMRQ3314,(Blikanasaurus,((Camelotia,((Meroktenos,(Ledumahadi,Kholumolumo)),(Lessemsaurus,(Ingentia,Antetonitrus)))),(Pulanesaura,(Gongxianosaurus,(Schleitheimia,(Isanosaurus,((Tazoudasaurus,Vulcanodon),((Shunosaurus,(Spinophorosaurus,(Mamenchisaurus,(Omeisaurus,(Cetiosaurus,Neosauropoda))))),(Patagosaurus,Barapasaurus)))))))))))))))),(Xingxiulong,(Jingshanosaurus,Chuxiongosaurus)))))),(Eucnemesaurus_fortis,(Riojasaurus,Eucnemesaurus_entaxonis)))),(Plateosaurus_gracilis,(Plateosaurus_ingens,Plateosaurus_engelhardti))))))))))))),(Buriolestes,Pampadromaeus)))),(Staurikosaurus,Herrerasaurus)))))));

(Euparkeria,(Crurotarsi,(Marasuchus,(Silesaurus,(Ornithischia,((Agnosphitys,((Guaibasaurus,(Neotheropoda,Chindesaurus)),(Buriolestes,((Eoraptor,((Saturnalia,Chromogisaurus),(Bagualosaurus,(Jaklapalisaurus,(Nambalia,((Thecodontosaurus,Pantydraco),(Efraasia,(Plateosauravus,(Ruehleia,(((Unaysaurus,(Pradhania,Macrocollum)),((Sarahsaurus,((Ngwevu,(Xixipiosaurus,((Coloradisaurus,(Glacialisaurus,Lufengosaurus)),(Massospondylus_carinatus,(Adeopapposaurus,Leyesaurus))))),(Yunnanosaurus_huangi,((Seitaad,(Anchisaurus,((Yizhousaurus,(Sefapanosaurus,Aardonyx)),((Mussaurus,Leonerasaurus),(Irisosaurus,(NMQR1551,(NMRQ3314,(Blikanasaurus,((Camelotia,((Meroktenos,(Ledumahadi,Kholumolumo)),(Lessemsaurus,(Ingentia,Antetonitrus)))),(Pulanesaura,(Gongxianosaurus,(Schleitheimia,(Isanosaurus,((Tazoudasaurus,Vulcanodon),((Shunosaurus,(Spinophorosaurus,(Mamenchisaurus,(Omeisaurus,(Cetiosaurus,Neosauropoda))))),(Patagosaurus,Barapasaurus)))))))))))))))),(Xingxiulong,(Jingshanosaurus,Chuxiongosaurus)))))),(Eucnemesaurus_fortis,(Riojasaurus,Eucnemesaurus_entaxonis)))),(Plateosaurus_gracilis,(Plateosaurus_ingens,Plateosaurus_engelhardti)))))))))))),(Panphagia,Pampadromaeus))))),(Staurikosaurus,Herrerasaurus)))))));

(Euparkeria,(Crurotarsi,(Marasuchus,(Silesaurus,(Ornithischia,((Agnosphitys,((Guaibasaurus,(Neotheropoda,Chindesaurus)),(Buriolestes,(Panphagia,(Eoraptor,((Saturnalia,Chromogisaurus),(Pampadromaeus,(Bagualosaurus,(Jaklapalisaurus,(Nambalia,((Thecodontosaurus,Pantydraco),(Efraasia,(Plateosauravus,(Ruehleia,(((Unaysaurus,(Pradhania,Macrocollum)),((Sarahsaurus,((Ngwevu,(Xixipiosaurus,((Coloradisaurus,(Glacialisaurus,Lufengosaurus)),(Massospondylus_carinatus,(Adeopapposaurus,Leyesaurus))))),(Yunnanosaurus_huangi,((Seitaad,(Anchisaurus,((Yizhousaurus,(Sefapanosaurus,Aardonyx)),((Mussaurus,Leonerasaurus),(Irisosaurus,(NMQR1551,(NMRQ3314,(Blikanasaurus,((Camelotia,((Meroktenos,(Ledumahadi,Kholumolumo)),(Lessemsaurus,(Ingentia,Antetonitrus)))),(Pulanesaura,(Gongxianosaurus,(Schleitheimia,(Isanosaurus,((Tazoudasaurus,Vulcanodon),((Shunosaurus,(Spinophorosaurus,(Mamenchisaurus,(Omeisaurus,(Cetiosaurus,Neosauropoda))))),(Patagosaurus,Barapasaurus)))))))))))))))),(Xingxiulong,(Jingshanosaurus,Chuxiongosaurus)))))),(Eucnemesaurus_fortis,(Riojasaurus,Eucnemesaurus_entaxonis)))),(Plateosaurus_gracilis,(Plateosaurus_ingens,Plateosaurus_engelhardti))))))))))))))))),(Staurikosaurus,Herrerasaurus)))))));

(Euparkeria,(Crurotarsi,(Marasuchus,(Silesaurus,(Ornithischia,((Agnosphitys,((Guaibasaurus,(Neotheropoda,Chindesaurus)),(Buriolestes,(Panphagia,(Eoraptor,((Pampadromaeus,(Saturnalia,Chromogisaurus)),(Bagualosaurus,(Jaklapalisaurus,(Nambalia,((Thecodontosaurus,Pantydraco),(Efraasia,(Plateosauravus,(Ruehleia,(((Unaysaurus,(Pradhania,Macrocollum)),((Sarahsaurus,((Ngwevu,(Xixipiosaurus,((Coloradisaurus,(Glacialisaurus,Lufengosaurus)),(Massospondylus_carinatus,(Adeopapposaurus,Leyesaurus))))),(Yunnanosaurus_huangi,((Seitaad,(Anchisaurus,((Yizhousaurus,(Sefapanosaurus,Aardonyx)),((Mussaurus,Leonerasaurus),(Irisosaurus,(NMQR1551,(NMRQ3314,(Blikanasaurus,((Camelotia,((Meroktenos,(Ledumahadi,Kholumolumo)),(Lessemsaurus,(Ingentia,Antetonitrus)))),(Pulanesaura,(Gongxianosaurus,(Schleitheimia,(Isanosaurus,((Tazoudasaurus,Vulcanodon),((Shunosaurus,(Spinophorosaurus,(Mamenchisaurus,(Omeisaurus,(Cetiosaurus,Neosauropoda))))),(Patagosaurus,Barapasaurus)))))))))))))))),(Xingxiulong,(Jingshanosaurus,Chuxiongosaurus)))))),(Eucnemesaurus_fortis,(Riojasaurus,Eucnemesaurus_entaxonis)))),(Plateosaurus_gracilis,(Plateosaurus_ingens,Plateosaurus_engelhardti)))))))))))))))),(Staurikosaurus,Herrerasaurus)))))));

(Euparkeria,(Crurotarsi,(Marasuchus,(Silesaurus,(Ornithischia,((Agnosphitys,((Guaibasaurus,(Neotheropoda,Chindesaurus)),(Buriolestes,(Pampadromaeus,(Panphagia,(Eoraptor,((Saturnalia,Chromogisaurus),(Bagualosaurus,(Jaklapalisaurus,(Nambalia,((Thecodontosaurus,Pantydraco),(Efraasia,(Plateosauravus,(Ruehleia,(((Unaysaurus,(Pradhania,Macrocollum)),((Sarahsaurus,((Ngwevu,((Coloradisaurus,(Glacialisaurus,Lufengosaurus)),(Xixipiosaurus,(Massospondylus_carinatus,(Adeopapposaurus,Leyesaurus))))),(Yunnanosaurus_huangi,((Seitaad,(Anchisaurus,((Yizhousaurus,(Sefapanosaurus,Aardonyx)),((Mussaurus,Leonerasaurus),(Irisosaurus,(NMQR1551,(NMRQ3314,(Blikanasaurus,((Camelotia,((Meroktenos,(Ledumahadi,Kholumolumo)),(Lessemsaurus,(Ingentia,Antetonitrus)))),(Pulanesaura,(Gongxianosaurus,(Schleitheimia,(Isanosaurus,((Tazoudasaurus,Vulcanodon),((Shunosaurus,(Spinophorosaurus,(Mamenchisaurus,(Omeisaurus,(Cetiosaurus,Neosauropoda))))),(Patagosaurus,Barapasaurus)))))))))))))))),(Xingxiulong,(Jingshanosaurus,Chuxiongosaurus)))))),(Eucnemesaurus_fortis,(Riojasaurus,Eucnemesaurus_entaxonis)))),(Plateosaurus_gracilis,(Plateosaurus_ingens,Plateosaurus_engelhardti))))))))))))))))),(Staurikosaurus,Herrerasaurus)))))));

(Euparkeria,(Crurotarsi,(Marasuchus,(Silesaurus,(Ornithischia,((Agnosphitys,((Guaibasaurus,(Neotheropoda,Chindesaurus)),(Buriolestes,(Pampadromaeus,(Panphagia,(Eoraptor,((Saturnalia,Chromogisaurus),(Bagualosaurus,(Jaklapalisaurus,(Nambalia,((Thecodontosaurus,Pantydraco),(Efraasia,(Plateosauravus,(Ruehleia,(((Unaysaurus,(Pradhania,Macrocollum)),((Sarahsaurus,((Ngwevu,(Xixipiosaurus,((Coloradisaurus,(Glacialisaurus,Lufengosaurus)),(Massospondylus_carinatus,(Adeopapposaurus,Leyesaurus))))),(Yunnanosaurus_huangi,((Seitaad,(Anchisaurus,((Yizhousaurus,Aardonyx),((Leonerasaurus,(Mussaurus,Sefapanosaurus)),(Irisosaurus,(NMQR1551,(NMRQ3314,(Blikanasaurus,((Camelotia,((Meroktenos,(Ledumahadi,Kholumolumo)),(Lessemsaurus,(Ingentia,Antetonitrus)))),(Pulanesaura,(Gongxianosaurus,(Schleitheimia,(Isanosaurus,((Tazoudasaurus,Vulcanodon),((Shunosaurus,(Spinophorosaurus,(Mamenchisaurus,(Omeisaurus,(Cetiosaurus,Neosauropoda))))),(Patagosaurus,Barapasaurus)))))))))))))))),(Xingxiulong,(Jingshanosaurus,Chuxiongosaurus)))))),(Eucnemesaurus_fortis,(Riojasaurus,Eucnemesaurus_entaxonis)))),(Plateosaurus_gracilis,(Plateosaurus_ingens,Plateosaurus_engelhardti))))))))))))))))),(Staurikosaurus,Herrerasaurus)))))));

(Euparkeria,(Crurotarsi,(Marasuchus,(Silesaurus,(Ornithischia,((Agnosphitys,((Guaibasaurus,(Neotheropoda,Chindesaurus)),(Buriolestes,(Pampadromaeus,(Panphagia,(Eoraptor,((Saturnalia,Chromogisaurus),(Bagualosaurus,(Jaklapalisaurus,(Nambalia,((Thecodontosaurus,Pantydraco),(Efraasia,(Plateosauravus,(Ruehleia,(((Unaysaurus,(Pradhania,Macrocollum)),((Sarahsaurus,((Ngwevu,(Xixipiosaurus,((Coloradisaurus,(Glacialisaurus,Lufengosaurus)),(Massospondylus_carinatus,(Adeopapposaurus,Leyesaurus))))),(Yunnanosaurus_huangi,((Seitaad,(Anchisaurus,((Yizhousaurus,(Sefapanosaurus,Aardonyx)),((Mussaurus,Leonerasaurus),(Irisosaurus,(NMQR1551,(NMRQ3314,(Blikanasaurus,((Camelotia,((Meroktenos,(Ledumahadi,Kholumolumo)),(Lessemsaurus,(Ingentia,Antetonitrus)))),((Schleitheimia,(Isanosaurus,((Tazoudasaurus,Vulcanodon),((Shunosaurus,(Spinophorosaurus,(Mamenchisaurus,(Omeisaurus,(Cetiosaurus,Neosauropoda))))),(Patagosaurus,Barapasaurus))))),(Pulanesaura,Gongxianosaurus))))))))))),(Xingxiulong,(Jingshanosaurus,Chuxiongosaurus)))))),(Eucnemesaurus_fortis,(Riojasaurus,Eucnemesaurus_entaxonis)))),(Plateosaurus_gracilis,(Plateosaurus_ingens,Plateosaurus_engelhardti))))))))))))))))),(Staurikosaurus,Herrerasaurus)))))));

(Euparkeria,(Crurotarsi,(Marasuchus,(Silesaurus,(Ornithischia,((Agnosphitys,((Guaibasaurus,(Neotheropoda,Chindesaurus)),((Eoraptor,((Saturnalia,Chromogisaurus),(Bagualosaurus,(Jaklapalisaurus,(Nambalia,((Thecodontosaurus,Pantydraco),(Efraasia,(Plateosauravus,(Ruehleia,(((Unaysaurus,(Pradhania,Macrocollum)),((Sarahsaurus,((Ngwevu,(Xixipiosaurus,((Coloradisaurus,(Glacialisaurus,Lufengosaurus)),(Massospondylus_carinatus,(Adeopapposaurus,Leyesaurus))))),(Yunnanosaurus_huangi,((Seitaad,(Anchisaurus,((Yizhousaurus,(Sefapanosaurus,Aardonyx)),((Mussaurus,Leonerasaurus),(Irisosaurus,(NMQR1551,(NMRQ3314,(Blikanasaurus,((Camelotia,((Meroktenos,(Ledumahadi,Kholumolumo)),(Lessemsaurus,(Ingentia,Antetonitrus)))),(Pulanesaura,(Gongxianosaurus,(Schleitheimia,(Isanosaurus,((Tazoudasaurus,Vulcanodon),((Shunosaurus,(Spinophorosaurus,(Mamenchisaurus,(Omeisaurus,(Cetiosaurus,Neosauropoda))))),(Patagosaurus,Barapasaurus)))))))))))))))),(Xingxiulong,(Jingshanosaurus,Chuxiongosaurus)))))),(Eucnemesaurus_fortis,(Riojasaurus,Eucnemesaurus_entaxonis)))),(Plateosaurus_gracilis,(Plateosaurus_ingens,Plateosaurus_engelhardti)))))))))))),(Buriolestes,(Panphagia,Pampadromaeus))))),(Staurikosaurus,Herrerasaurus)))))));

(Euparkeria,(Crurotarsi,(Marasuchus,(Silesaurus,(Ornithischia,((Agnosphitys,((Guaibasaurus,(Neotheropoda,Chindesaurus)),(Buriolestes,(Pampadromaeus,(Panphagia,(Eoraptor,((Saturnalia,Chromogisaurus),(Bagualosaurus,(Jaklapalisaurus,(Nambalia,((Thecodontosaurus,Pantydraco),(Efraasia,(Plateosauravus,(Ruehleia,(((Unaysaurus,(Pradhania,Macrocollum)),((Sarahsaurus,((Ngwevu,(Xixipiosaurus,((Coloradisaurus,(Glacialisaurus,Lufengosaurus)),(Massospondylus_carinatus,(Adeopapposaurus,Leyesaurus))))),(Yunnanosaurus_huangi,((Seitaad,(Anchisaurus,((Yizhousaurus,(Sefapanosaurus,Aardonyx)),((Mussaurus,Leonerasaurus),((Irisosaurus,(NMRQ3314,NMQR1551)),(Blikanasaurus,((Camelotia,((Meroktenos,(Ledumahadi,Kholumolumo)),(Lessemsaurus,(Ingentia,Antetonitrus)))),(Pulanesaura,(Gongxianosaurus,(Schleitheimia,(Isanosaurus,((Tazoudasaurus,Vulcanodon),((Shunosaurus,(Spinophorosaurus,(Mamenchisaurus,(Omeisaurus,(Cetiosaurus,Neosauropoda))))),(Patagosaurus,Barapasaurus)))))))))))))),(Xingxiulong,(Jingshanosaurus,Chuxiongosaurus)))))),(Eucnemesaurus_fortis,(Riojasaurus,Eucnemesaurus_entaxonis)))),(Plateosaurus_gracilis,(Plateosaurus_ingens,Plateosaurus_engelhardti))))))))))))))))),(Staurikosaurus,Herrerasaurus)))))));

(Euparkeria,(Crurotarsi,(Marasuchus,(Silesaurus,(Agnosphitys,(Ornithischia,(((Guaibasaurus,(Neotheropoda,Chindesaurus)),((Panphagia,(Eoraptor,((Saturnalia,Chromogisaurus),(Bagualosaurus,(Jaklapalisaurus,(Nambalia,((Thecodontosaurus,Pantydraco),(Efraasia,(Plateosauravus,(Ruehleia,(((Unaysaurus,(Pradhania,Macrocollum)),((Sarahsaurus,((Ngwevu,((Coloradisaurus,(Glacialisaurus,Lufengosaurus)),(Xixipiosaurus,(Massospondylus_carinatus,(Adeopapposaurus,Leyesaurus))))),(Yunnanosaurus_huangi,((Seitaad,(Anchisaurus,((Yizhousaurus,(Sefapanosaurus,Aardonyx)),((Mussaurus,Leonerasaurus),(Irisosaurus,(NMQR1551,(NMRQ3314,(Blikanasaurus,((Camelotia,((Meroktenos,(Ledumahadi,Kholumolumo)),(Lessemsaurus,(Ingentia,Antetonitrus)))),(Pulanesaura,(Gongxianosaurus,(Schleitheimia,(Isanosaurus,((Tazoudasaurus,Vulcanodon),((Shunosaurus,(Spinophorosaurus,(Mamenchisaurus,(Omeisaurus,(Cetiosaurus,Neosauropoda))))),(Patagosaurus,Barapasaurus)))))))))))))))),(Xingxiulong,(Jingshanosaurus,Chuxiongosaurus)))))),(Eucnemesaurus_fortis,(Riojasaurus,Eucnemesaurus_entaxonis)))),(Plateosaurus_gracilis,(Plateosaurus_ingens,Plateosaurus_engelhardti))))))))))))),(Buriolestes,Pampadromaeus))),(Staurikosaurus,Herrerasaurus))))))));

(Euparkeria,(Crurotarsi,(Marasuchus,(Silesaurus,(Agnosphitys,(Ornithischia,(((Guaibasaurus,(Neotheropoda,Chindesaurus)),((Panphagia,(Eoraptor,((Saturnalia,Chromogisaurus),(Bagualosaurus,(Jaklapalisaurus,(Nambalia,((Thecodontosaurus,Pantydraco),(Efraasia,(Plateosauravus,(Ruehleia,(((Unaysaurus,(Pradhania,Macrocollum)),((Sarahsaurus,((Ngwevu,(Xixipiosaurus,((Coloradisaurus,(Glacialisaurus,Lufengosaurus)),(Massospondylus_carinatus,(Adeopapposaurus,Leyesaurus))))),(Yunnanosaurus_huangi,((Seitaad,(Anchisaurus,((Yizhousaurus,Aardonyx),((Leonerasaurus,(Mussaurus,Sefapanosaurus)),(Irisosaurus,(NMQR1551,(NMRQ3314,(Blikanasaurus,((Camelotia,((Meroktenos,(Ledumahadi,Kholumolumo)),(Lessemsaurus,(Ingentia,Antetonitrus)))),(Pulanesaura,(Gongxianosaurus,(Schleitheimia,(Isanosaurus,((Tazoudasaurus,Vulcanodon),((Shunosaurus,(Spinophorosaurus,(Mamenchisaurus,(Omeisaurus,(Cetiosaurus,Neosauropoda))))),(Patagosaurus,Barapasaurus)))))))))))))))),(Xingxiulong,(Jingshanosaurus,Chuxiongosaurus)))))),(Eucnemesaurus_fortis,(Riojasaurus,Eucnemesaurus_entaxonis)))),(Plateosaurus_gracilis,(Plateosaurus_ingens,Plateosaurus_engelhardti))))))))))))),(Buriolestes,Pampadromaeus))),(Staurikosaurus,Herrerasaurus))))))));

(Euparkeria,(Crurotarsi,(Marasuchus,(Silesaurus,(Agnosphitys,(Ornithischia,(((Guaibasaurus,(Neotheropoda,Chindesaurus)),((Panphagia,(Eoraptor,((Saturnalia,Chromogisaurus),(Bagualosaurus,(Jaklapalisaurus,(Nambalia,((Thecodontosaurus,Pantydraco),(Efraasia,(Plateosauravus,(Ruehleia,(((Unaysaurus,(Pradhania,Macrocollum)),((Sarahsaurus,((Ngwevu,(Xixipiosaurus,((Coloradisaurus,(Glacialisaurus,Lufengosaurus)),(Massospondylus_carinatus,(Adeopapposaurus,Leyesaurus))))),(Yunnanosaurus_huangi,((Seitaad,(Anchisaurus,((Yizhousaurus,(Sefapanosaurus,Aardonyx)),((Mussaurus,Leonerasaurus),(Irisosaurus,(NMQR1551,(NMRQ3314,(Blikanasaurus,((Camelotia,((Meroktenos,(Ledumahadi,Kholumolumo)),(Lessemsaurus,(Ingentia,Antetonitrus)))),((Schleitheimia,(Isanosaurus,((Tazoudasaurus,Vulcanodon),((Shunosaurus,(Spinophorosaurus,(Mamenchisaurus,(Omeisaurus,(Cetiosaurus,Neosauropoda))))),(Patagosaurus,Barapasaurus))))),(Pulanesaura,Gongxianosaurus))))))))))),(Xingxiulong,(Jingshanosaurus,Chuxiongosaurus)))))),(Eucnemesaurus_fortis,(Riojasaurus,Eucnemesaurus_entaxonis)))),(Plateosaurus_gracilis,(Plateosaurus_ingens,Plateosaurus_engelhardti))))))))))))),(Buriolestes,Pampadromaeus))),(Staurikosaurus,Herrerasaurus))))))));

(Euparkeria,(Crurotarsi,(Marasuchus,(Silesaurus,(Agnosphitys,(Ornithischia,(((Guaibasaurus,(Neotheropoda,Chindesaurus)),((Panphagia,(Eoraptor,((Saturnalia,Chromogisaurus),(Bagualosaurus,(Jaklapalisaurus,(Nambalia,((Thecodontosaurus,Pantydraco),(Efraasia,(Plateosauravus,(Ruehleia,(((Unaysaurus,(Pradhania,Macrocollum)),((Sarahsaurus,((Ngwevu,(Xixipiosaurus,((Coloradisaurus,(Glacialisaurus,Lufengosaurus)),(Massospondylus_carinatus,(Adeopapposaurus,Leyesaurus))))),(Yunnanosaurus_huangi,((Seitaad,(Anchisaurus,((Yizhousaurus,(Sefapanosaurus,Aardonyx)),((Mussaurus,Leonerasaurus),((Irisosaurus,(NMRQ3314,NMQR1551)),(Blikanasaurus,((Camelotia,((Meroktenos,(Ledumahadi,Kholumolumo)),(Lessemsaurus,(Ingentia,Antetonitrus)))),(Pulanesaura,(Gongxianosaurus,(Schleitheimia,(Isanosaurus,((Tazoudasaurus,Vulcanodon),((Shunosaurus,(Spinophorosaurus,(Mamenchisaurus,(Omeisaurus,(Cetiosaurus,Neosauropoda))))),(Patagosaurus,Barapasaurus)))))))))))))),(Xingxiulong,(Jingshanosaurus,Chuxiongosaurus)))))),(Eucnemesaurus_fortis,(Riojasaurus,Eucnemesaurus_entaxonis)))),(Plateosaurus_gracilis,(Plateosaurus_ingens,Plateosaurus_engelhardti))))))))))))),(Buriolestes,Pampadromaeus))),(Staurikosaurus,Herrerasaurus))))))));

(Euparkeria,(Crurotarsi,(Marasuchus,(Silesaurus,(Agnosphitys,(Ornithischia,(((Guaibasaurus,(Neotheropoda,Chindesaurus)),(Buriolestes,(Pampadromaeus,(Panphagia,(Eoraptor,((Saturnalia,Chromogisaurus),(Bagualosaurus,(Jaklapalisaurus,(Nambalia,((Thecodontosaurus,Pantydraco),(Efraasia,(Plateosauravus,(Ruehleia,(((Unaysaurus,(Pradhania,Macrocollum)),((Sarahsaurus,((Ngwevu,((Coloradisaurus,(Glacialisaurus,Lufengosaurus)),(Xixipiosaurus,(Massospondylus_carinatus,(Adeopapposaurus,Leyesaurus))))),(Yunnanosaurus_huangi,((Seitaad,(Anchisaurus,((Yizhousaurus,Aardonyx),((Leonerasaurus,(Mussaurus,Sefapanosaurus)),(Irisosaurus,(NMQR1551,(NMRQ3314,(Blikanasaurus,((Camelotia,((Meroktenos,(Ledumahadi,Kholumolumo)),(Lessemsaurus,(Ingentia,Antetonitrus)))),(Pulanesaura,(Gongxianosaurus,(Schleitheimia,(Isanosaurus,((Tazoudasaurus,Vulcanodon),((Shunosaurus,(Spinophorosaurus,(Mamenchisaurus,(Omeisaurus,(Cetiosaurus,Neosauropoda))))),(Patagosaurus,Barapasaurus)))))))))))))))),(Xingxiulong,(Jingshanosaurus,Chuxiongosaurus)))))),(Eucnemesaurus_fortis,(Riojasaurus,Eucnemesaurus_entaxonis)))),(Plateosaurus_gracilis,(Plateosaurus_ingens,Plateosaurus_engelhardti)))))))))))))))),(Staurikosaurus,Herrerasaurus))))))));

(Euparkeria,(Crurotarsi,(Marasuchus,(Silesaurus,(Agnosphitys,(Ornithischia,(((Guaibasaurus,(Neotheropoda,Chindesaurus)),(Buriolestes,(Pampadromaeus,(Panphagia,(Eoraptor,((Saturnalia,Chromogisaurus),(Bagualosaurus,(Jaklapalisaurus,(Nambalia,((Thecodontosaurus,Pantydraco),(Efraasia,(Plateosauravus,(Ruehleia,(((Unaysaurus,(Pradhania,Macrocollum)),((Sarahsaurus,((Ngwevu,((Coloradisaurus,(Glacialisaurus,Lufengosaurus)),(Xixipiosaurus,(Massospondylus_carinatus,(Adeopapposaurus,Leyesaurus))))),(Yunnanosaurus_huangi,((Seitaad,(Anchisaurus,((Yizhousaurus,(Sefapanosaurus,Aardonyx)),((Mussaurus,Leonerasaurus),(Irisosaurus,(NMQR1551,(NMRQ3314,(Blikanasaurus,((Camelotia,((Meroktenos,(Ledumahadi,Kholumolumo)),(Lessemsaurus,(Ingentia,Antetonitrus)))),((Schleitheimia,(Isanosaurus,((Tazoudasaurus,Vulcanodon),((Shunosaurus,(Spinophorosaurus,(Mamenchisaurus,(Omeisaurus,(Cetiosaurus,Neosauropoda))))),(Patagosaurus,Barapasaurus))))),(Pulanesaura,Gongxianosaurus))))))))))),(Xingxiulong,(Jingshanosaurus,Chuxiongosaurus)))))),(Eucnemesaurus_fortis,(Riojasaurus,Eucnemesaurus_entaxonis)))),(Plateosaurus_gracilis,(Plateosaurus_ingens,Plateosaurus_engelhardti)))))))))))))))),(Staurikosaurus,Herrerasaurus))))))));

(Euparkeria,(Crurotarsi,(Marasuchus,(Silesaurus,(Agnosphitys,(Ornithischia,(((Guaibasaurus,(Neotheropoda,Chindesaurus)),(Buriolestes,(Pampadromaeus,(Panphagia,(Eoraptor,((Saturnalia,Chromogisaurus),(Bagualosaurus,(Jaklapalisaurus,(Nambalia,((Thecodontosaurus,Pantydraco),(Efraasia,(Plateosauravus,(Ruehleia,(((Unaysaurus,(Pradhania,Macrocollum)),((Sarahsaurus,((Ngwevu,((Coloradisaurus,(Glacialisaurus,Lufengosaurus)),(Xixipiosaurus,(Massospondylus_carinatus,(Adeopapposaurus,Leyesaurus))))),(Yunnanosaurus_huangi,((Seitaad,(Anchisaurus,((Yizhousaurus,(Sefapanosaurus,Aardonyx)),((Mussaurus,Leonerasaurus),((Irisosaurus,(NMRQ3314,NMQR1551)),(Blikanasaurus,((Camelotia,((Meroktenos,(Ledumahadi,Kholumolumo)),(Lessemsaurus,(Ingentia,Antetonitrus)))),(Pulanesaura,(Gongxianosaurus,(Schleitheimia,(Isanosaurus,((Tazoudasaurus,Vulcanodon),((Shunosaurus,(Spinophorosaurus,(Mamenchisaurus,(Omeisaurus,(Cetiosaurus,Neosauropoda))))),(Patagosaurus,Barapasaurus)))))))))))))),(Xingxiulong,(Jingshanosaurus,Chuxiongosaurus)))))),(Eucnemesaurus_fortis,(Riojasaurus,Eucnemesaurus_entaxonis)))),(Plateosaurus_gracilis,(Plateosaurus_ingens,Plateosaurus_engelhardti)))))))))))))))),(Staurikosaurus,Herrerasaurus))))))));

(Euparkeria,(Crurotarsi,(Marasuchus,(Silesaurus,(Agnosphitys,(Ornithischia,(((Guaibasaurus,(Neotheropoda,Chindesaurus)),(Buriolestes,(Pampadromaeus,(Panphagia,(Eoraptor,((Saturnalia,Chromogisaurus),(Bagualosaurus,(Jaklapalisaurus,(Nambalia,((Thecodontosaurus,Pantydraco),(Efraasia,(Plateosauravus,(Ruehleia,(((Unaysaurus,(Pradhania,Macrocollum)),((Sarahsaurus,((Ngwevu,(Xixipiosaurus,((Coloradisaurus,(Glacialisaurus,Lufengosaurus)),(Massospondylus_carinatus,(Adeopapposaurus,Leyesaurus))))),(Yunnanosaurus_huangi,((Seitaad,(Anchisaurus,((Yizhousaurus,Aardonyx),((Leonerasaurus,(Mussaurus,Sefapanosaurus)),(Irisosaurus,(NMQR1551,(NMRQ3314,(Blikanasaurus,((Camelotia,((Meroktenos,(Ledumahadi,Kholumolumo)),(Lessemsaurus,(Ingentia,Antetonitrus)))),((Schleitheimia,(Isanosaurus,((Tazoudasaurus,Vulcanodon),((Shunosaurus,(Spinophorosaurus,(Mamenchisaurus,(Omeisaurus,(Cetiosaurus,Neosauropoda))))),(Patagosaurus,Barapasaurus))))),(Pulanesaura,Gongxianosaurus))))))))))),(Xingxiulong,(Jingshanosaurus,Chuxiongosaurus)))))),(Eucnemesaurus_fortis,(Riojasaurus,Eucnemesaurus_entaxonis)))),(Plateosaurus_gracilis,(Plateosaurus_ingens,Plateosaurus_engelhardti)))))))))))))))),(Staurikosaurus,Herrerasaurus))))))));

(Euparkeria,(Crurotarsi,(Marasuchus,(Silesaurus,(Agnosphitys,(Ornithischia,(((Guaibasaurus,(Neotheropoda,Chindesaurus)),(Buriolestes,(Pampadromaeus,(Panphagia,(Eoraptor,((Saturnalia,Chromogisaurus),(Bagualosaurus,(Jaklapalisaurus,(Nambalia,((Thecodontosaurus,Pantydraco),(Efraasia,(Plateosauravus,(Ruehleia,(((Unaysaurus,(Pradhania,Macrocollum)),((Sarahsaurus,((Ngwevu,(Xixipiosaurus,((Coloradisaurus,(Glacialisaurus,Lufengosaurus)),(Massospondylus_carinatus,(Adeopapposaurus,Leyesaurus))))),(Yunnanosaurus_huangi,((Seitaad,(Anchisaurus,((Yizhousaurus,Aardonyx),((Leonerasaurus,(Mussaurus,Sefapanosaurus)),((Irisosaurus,(NMRQ3314,NMQR1551)),(Blikanasaurus,((Camelotia,((Meroktenos,(Ledumahadi,Kholumolumo)),(Lessemsaurus,(Ingentia,Antetonitrus)))),(Pulanesaura,(Gongxianosaurus,(Schleitheimia,(Isanosaurus,((Tazoudasaurus,Vulcanodon),((Shunosaurus,(Spinophorosaurus,(Mamenchisaurus,(Omeisaurus,(Cetiosaurus,Neosauropoda))))),(Patagosaurus,Barapasaurus)))))))))))))),(Xingxiulong,(Jingshanosaurus,Chuxiongosaurus)))))),(Eucnemesaurus_fortis,(Riojasaurus,Eucnemesaurus_entaxonis)))),(Plateosaurus_gracilis,(Plateosaurus_ingens,Plateosaurus_engelhardti)))))))))))))))),(Staurikosaurus,Herrerasaurus))))))));

(Euparkeria,(Crurotarsi,(Marasuchus,(Silesaurus,(Agnosphitys,(Ornithischia,(((Guaibasaurus,(Neotheropoda,Chindesaurus)),(Buriolestes,(Pampadromaeus,(Panphagia,(Eoraptor,((Saturnalia,Chromogisaurus),(Bagualosaurus,(Jaklapalisaurus,(Nambalia,((Thecodontosaurus,Pantydraco),(Efraasia,(Plateosauravus,(Ruehleia,(((Unaysaurus,(Pradhania,Macrocollum)),((Sarahsaurus,((Ngwevu,(Xixipiosaurus,((Coloradisaurus,(Glacialisaurus,Lufengosaurus)),(Massospondylus_carinatus,(Adeopapposaurus,Leyesaurus))))),(Yunnanosaurus_huangi,((Seitaad,(Anchisaurus,(((Yizhousaurus,((Irisosaurus,(NMRQ3314,NMQR1551)),(Blikanasaurus,((Camelotia,((Meroktenos,(Ledumahadi,Kholumolumo)),(Lessemsaurus,(Ingentia,Antetonitrus)))),(Pulanesaura,(Gongxianosaurus,(Schleitheimia,(Isanosaurus,((Tazoudasaurus,Vulcanodon),((Shunosaurus,(Spinophorosaurus,(Mamenchisaurus,(Omeisaurus,(Cetiosaurus,Neosauropoda))))),(Patagosaurus,Barapasaurus))))))))))),(Sefapanosaurus,Aardonyx)),(Mussaurus,Leonerasaurus)))),(Xingxiulong,(Jingshanosaurus,Chuxiongosaurus)))))),(Eucnemesaurus_fortis,(Riojasaurus,Eucnemesaurus_entaxonis)))),(Plateosaurus_gracilis,(Plateosaurus_ingens,Plateosaurus_engelhardti)))))))))))))))),(Staurikosaurus,Herrerasaurus))))))));

(Euparkeria,(Crurotarsi,(Marasuchus,(Silesaurus,(Ornithischia,(Agnosphitys,(((Guaibasaurus,(Neotheropoda,Chindesaurus)),((Panphagia,(Eoraptor,((Saturnalia,Chromogisaurus),(Bagualosaurus,(Jaklapalisaurus,(Nambalia,((Thecodontosaurus,Pantydraco),(Efraasia,(Plateosauravus,(Ruehleia,(((Unaysaurus,(Pradhania,Macrocollum)),((Sarahsaurus,((Ngwevu,(Xixipiosaurus,((Coloradisaurus,(Glacialisaurus,Lufengosaurus)),(Massospondylus_carinatus,(Adeopapposaurus,Leyesaurus))))),(Yunnanosaurus_huangi,((Seitaad,(Anchisaurus,((Yizhousaurus,((Sefapanosaurus,Aardonyx),(NMQR1551,(NMRQ3314,(Blikanasaurus,((Camelotia,((Meroktenos,(Ledumahadi,Kholumolumo)),(Lessemsaurus,(Ingentia,Antetonitrus)))),((Schleitheimia,(Isanosaurus,((Tazoudasaurus,Vulcanodon),((Shunosaurus,(Spinophorosaurus,(Mamenchisaurus,(Omeisaurus,(Cetiosaurus,Neosauropoda))))),(Patagosaurus,Barapasaurus))))),(Pulanesaura,Gongxianosaurus)))))))),(Irisosaurus,(Mussaurus,Leonerasaurus))))),(Xingxiulong,(Jingshanosaurus,Chuxiongosaurus)))))),(Eucnemesaurus_fortis,(Riojasaurus,Eucnemesaurus_entaxonis)))),(Plateosaurus_gracilis,(Plateosaurus_ingens,Plateosaurus_engelhardti))))))))))))),(Buriolestes,Pampadromaeus))),(Staurikosaurus,Herrerasaurus))))))));

(Euparkeria,(Crurotarsi,(Marasuchus,(Silesaurus,(Ornithischia,(Agnosphitys,(((Guaibasaurus,(Neotheropoda,Chindesaurus)),(Buriolestes,(Pampadromaeus,(Panphagia,(Eoraptor,((Saturnalia,Chromogisaurus),(Bagualosaurus,(Jaklapalisaurus,(Nambalia,((Thecodontosaurus,Pantydraco),(Efraasia,(Plateosauravus,(Ruehleia,(((Unaysaurus,(Pradhania,Macrocollum)),((Sarahsaurus,((Ngwevu,((Coloradisaurus,(Glacialisaurus,Lufengosaurus)),(Xixipiosaurus,(Massospondylus_carinatus,(Adeopapposaurus,Leyesaurus))))),(Yunnanosaurus_huangi,((Seitaad,(Anchisaurus,((Yizhousaurus,((Sefapanosaurus,Aardonyx),(NMQR1551,(NMRQ3314,(Blikanasaurus,((Camelotia,((Meroktenos,(Ledumahadi,Kholumolumo)),(Lessemsaurus,(Ingentia,Antetonitrus)))),((Schleitheimia,(Isanosaurus,((Tazoudasaurus,Vulcanodon),((Shunosaurus,(Spinophorosaurus,(Mamenchisaurus,(Omeisaurus,(Cetiosaurus,Neosauropoda))))),(Patagosaurus,Barapasaurus))))),(Pulanesaura,Gongxianosaurus)))))))),(Irisosaurus,(Mussaurus,Leonerasaurus))))),(Xingxiulong,(Jingshanosaurus,Chuxiongosaurus)))))),(Eucnemesaurus_fortis,(Riojasaurus,Eucnemesaurus_entaxonis)))),(Plateosaurus_gracilis,(Plateosaurus_ingens,Plateosaurus_engelhardti)))))))))))))))),(Staurikosaurus,Herrerasaurus))))))));

(Euparkeria,(Crurotarsi,(Marasuchus,(Silesaurus,(Ornithischia,(Agnosphitys,(((Guaibasaurus,(Neotheropoda,Chindesaurus)),(Buriolestes,(Pampadromaeus,(Panphagia,(Eoraptor,((Saturnalia,Chromogisaurus),(Bagualosaurus,(Jaklapalisaurus,(Nambalia,((Thecodontosaurus,Pantydraco),(Efraasia,(Plateosauravus,(Ruehleia,(((Unaysaurus,(Pradhania,Macrocollum)),((Sarahsaurus,((Ngwevu,(Xixipiosaurus,((Coloradisaurus,(Glacialisaurus,Lufengosaurus)),(Massospondylus_carinatus,(Adeopapposaurus,Leyesaurus))))),(Yunnanosaurus_huangi,((Seitaad,(Anchisaurus,((Yizhousaurus,((Sefapanosaurus,Aardonyx),(NMQR1551,(NMRQ3314,(Blikanasaurus,((Camelotia,((Meroktenos,(Ledumahadi,Kholumolumo)),(Lessemsaurus,(Ingentia,Antetonitrus)))),((Schleitheimia,(Isanosaurus,((Tazoudasaurus,Vulcanodon),((Shunosaurus,(Spinophorosaurus,(Omeisaurus,(Mamenchisaurus,(Cetiosaurus,Neosauropoda))))),(Patagosaurus,Barapasaurus))))),(Pulanesaura,Gongxianosaurus)))))))),(Irisosaurus,(Mussaurus,Leonerasaurus))))),(Xingxiulong,(Jingshanosaurus,Chuxiongosaurus)))))),(Eucnemesaurus_fortis,(Riojasaurus,Eucnemesaurus_entaxonis)))),(Plateosaurus_gracilis,(Plateosaurus_ingens,Plateosaurus_engelhardti)))))))))))))))),(Staurikosaurus,Herrerasaurus))))))));

(Euparkeria,(Crurotarsi,(Marasuchus,(Silesaurus,(Ornithischia,(Agnosphitys,(((Guaibasaurus,(Neotheropoda,Chindesaurus)),(Buriolestes,(Pampadromaeus,(Panphagia,(Eoraptor,((Saturnalia,Chromogisaurus),(Bagualosaurus,(Jaklapalisaurus,(Nambalia,((Thecodontosaurus,Pantydraco),(Efraasia,(Plateosauravus,(Ruehleia,(((Unaysaurus,(Pradhania,Macrocollum)),((Sarahsaurus,((Ngwevu,(Xixipiosaurus,((Coloradisaurus,(Glacialisaurus,Lufengosaurus)),(Massospondylus_carinatus,(Adeopapposaurus,Leyesaurus))))),(Yunnanosaurus_huangi,((Seitaad,(Anchisaurus,((Yizhousaurus,((Sefapanosaurus,Aardonyx),(NMQR1551,(NMRQ3314,(Blikanasaurus,((Camelotia,((Meroktenos,(Ledumahadi,Kholumolumo)),(Lessemsaurus,(Ingentia,Antetonitrus)))),((Schleitheimia,(Isanosaurus,((Tazoudasaurus,Vulcanodon),((Shunosaurus,((Spinophorosaurus,Omeisaurus),(Mamenchisaurus,(Cetiosaurus,Neosauropoda)))),(Patagosaurus,Barapasaurus))))),(Pulanesaura,Gongxianosaurus)))))))),(Irisosaurus,(Mussaurus,Leonerasaurus))))),(Xingxiulong,(Jingshanosaurus,Chuxiongosaurus)))))),(Eucnemesaurus_fortis,(Riojasaurus,Eucnemesaurus_entaxonis)))),(Plateosaurus_gracilis,(Plateosaurus_ingens,Plateosaurus_engelhardti)))))))))))))))),(Staurikosaurus,Herrerasaurus))))))));

(Euparkeria,(Crurotarsi,(Marasuchus,(Silesaurus,(Ornithischia,(Agnosphitys,(((Guaibasaurus,(Neotheropoda,Chindesaurus)),(Buriolestes,(Pampadromaeus,(Panphagia,(Eoraptor,((Saturnalia,Chromogisaurus),(Bagualosaurus,(Jaklapalisaurus,(Nambalia,((Thecodontosaurus,Pantydraco),(Efraasia,(Plateosauravus,(Ruehleia,(((Unaysaurus,(Pradhania,Macrocollum)),((Sarahsaurus,((Ngwevu,(Xixipiosaurus,((Coloradisaurus,(Glacialisaurus,Lufengosaurus)),(Massospondylus_carinatus,(Adeopapposaurus,Leyesaurus))))),(Yunnanosaurus_huangi,((Seitaad,(Anchisaurus,((Yizhousaurus,((Sefapanosaurus,Aardonyx),(NMQR1551,(NMRQ3314,(Blikanasaurus,((Camelotia,((Meroktenos,(Ledumahadi,Kholumolumo)),(Lessemsaurus,(Ingentia,Antetonitrus)))),((Schleitheimia,(Isanosaurus,((Tazoudasaurus,Vulcanodon),((Shunosaurus,((Spinophorosaurus,(Omeisaurus,Mamenchisaurus)),(Cetiosaurus,Neosauropoda))),(Patagosaurus,Barapasaurus))))),(Pulanesaura,Gongxianosaurus)))))))),(Irisosaurus,(Mussaurus,Leonerasaurus))))),(Xingxiulong,(Jingshanosaurus,Chuxiongosaurus)))))),(Eucnemesaurus_fortis,(Riojasaurus,Eucnemesaurus_entaxonis)))),(Plateosaurus_gracilis,(Plateosaurus_ingens,Plateosaurus_engelhardti)))))))))))))))),(Staurikosaurus,Herrerasaurus))))))));

(Euparkeria,(Crurotarsi,(Marasuchus,(Silesaurus,(Ornithischia,((Agnosphitys,((Guaibasaurus,(Neotheropoda,Chindesaurus)),((Panphagia,(Eoraptor,((Saturnalia,Chromogisaurus),(Bagualosaurus,(Jaklapalisaurus,(Nambalia,((Thecodontosaurus,Pantydraco),(Efraasia,(Plateosauravus,(Ruehleia,(((Unaysaurus,(Pradhania,Macrocollum)),((Sarahsaurus,((Ngwevu,(Xixipiosaurus,((Coloradisaurus,(Glacialisaurus,Lufengosaurus)),(Massospondylus_carinatus,(Adeopapposaurus,Leyesaurus))))),(Yunnanosaurus_huangi,((Seitaad,(Anchisaurus,((Yizhousaurus,((Sefapanosaurus,Aardonyx),(NMQR1551,(NMRQ3314,(Blikanasaurus,((Camelotia,((Meroktenos,(Ledumahadi,Kholumolumo)),(Lessemsaurus,(Ingentia,Antetonitrus)))),((Schleitheimia,(Isanosaurus,((Tazoudasaurus,Vulcanodon),((Shunosaurus,(Spinophorosaurus,(Mamenchisaurus,(Omeisaurus,(Cetiosaurus,Neosauropoda))))),(Patagosaurus,Barapasaurus))))),(Pulanesaura,Gongxianosaurus)))))))),(Irisosaurus,(Mussaurus,Leonerasaurus))))),(Xingxiulong,(Jingshanosaurus,Chuxiongosaurus)))))),(Eucnemesaurus_fortis,(Riojasaurus,Eucnemesaurus_entaxonis)))),(Plateosaurus_gracilis,(Plateosaurus_ingens,Plateosaurus_engelhardti))))))))))))),(Buriolestes,Pampadromaeus)))),(Staurikosaurus,Herrerasaurus)))))));

(Euparkeria,(Crurotarsi,(Marasuchus,(Silesaurus,(Ornithischia,((Agnosphitys,((Guaibasaurus,(Neotheropoda,Chindesaurus)),(Buriolestes,((Eoraptor,((Saturnalia,Chromogisaurus),(Bagualosaurus,(Jaklapalisaurus,(Nambalia,((Thecodontosaurus,Pantydraco),(Efraasia,(Plateosauravus,(Ruehleia,(((Unaysaurus,(Pradhania,Macrocollum)),((Sarahsaurus,((Ngwevu,(Xixipiosaurus,((Coloradisaurus,(Glacialisaurus,Lufengosaurus)),(Massospondylus_carinatus,(Adeopapposaurus,Leyesaurus))))),(Yunnanosaurus_huangi,((Seitaad,(Anchisaurus,((Yizhousaurus,((Sefapanosaurus,Aardonyx),(NMQR1551,(NMRQ3314,(Blikanasaurus,((Camelotia,((Meroktenos,(Ledumahadi,Kholumolumo)),(Lessemsaurus,(Ingentia,Antetonitrus)))),((Schleitheimia,(Isanosaurus,((Tazoudasaurus,Vulcanodon),((Shunosaurus,(Spinophorosaurus,(Mamenchisaurus,(Omeisaurus,(Cetiosaurus,Neosauropoda))))),(Patagosaurus,Barapasaurus))))),(Pulanesaura,Gongxianosaurus)))))))),(Irisosaurus,(Mussaurus,Leonerasaurus))))),(Xingxiulong,(Jingshanosaurus,Chuxiongosaurus)))))),(Eucnemesaurus_fortis,(Riojasaurus,Eucnemesaurus_entaxonis)))),(Plateosaurus_gracilis,(Plateosaurus_ingens,Plateosaurus_engelhardti)))))))))))),(Panphagia,Pampadromaeus))))),(Staurikosaurus,Herrerasaurus)))))));

(Euparkeria,(Crurotarsi,(Marasuchus,(Silesaurus,(Ornithischia,((Agnosphitys,((Guaibasaurus,(Neotheropoda,Chindesaurus)),(Buriolestes,(Panphagia,(Eoraptor,((Saturnalia,Chromogisaurus),(Pampadromaeus,(Bagualosaurus,(Jaklapalisaurus,(Nambalia,((Thecodontosaurus,Pantydraco),(Efraasia,(Plateosauravus,(Ruehleia,(((Unaysaurus,(Pradhania,Macrocollum)),((Sarahsaurus,((Ngwevu,(Xixipiosaurus,((Coloradisaurus,(Glacialisaurus,Lufengosaurus)),(Massospondylus_carinatus,(Adeopapposaurus,Leyesaurus))))),(Yunnanosaurus_huangi,((Seitaad,(Anchisaurus,((Yizhousaurus,((Sefapanosaurus,Aardonyx),(NMQR1551,(NMRQ3314,(Blikanasaurus,((Camelotia,((Meroktenos,(Ledumahadi,Kholumolumo)),(Lessemsaurus,(Ingentia,Antetonitrus)))),((Schleitheimia,(Isanosaurus,((Tazoudasaurus,Vulcanodon),((Shunosaurus,(Spinophorosaurus,(Mamenchisaurus,(Omeisaurus,(Cetiosaurus,Neosauropoda))))),(Patagosaurus,Barapasaurus))))),(Pulanesaura,Gongxianosaurus)))))))),(Irisosaurus,(Mussaurus,Leonerasaurus))))),(Xingxiulong,(Jingshanosaurus,Chuxiongosaurus)))))),(Eucnemesaurus_fortis,(Riojasaurus,Eucnemesaurus_entaxonis)))),(Plateosaurus_gracilis,(Plateosaurus_ingens,Plateosaurus_engelhardti))))))))))))))))),(Staurikosaurus,Herrerasaurus)))))));

(Euparkeria,(Crurotarsi,(Marasuchus,(Silesaurus,(Ornithischia,((Agnosphitys,((Guaibasaurus,(Neotheropoda,Chindesaurus)),(Buriolestes,(Pampadromaeus,(Panphagia,(Eoraptor,((Saturnalia,Chromogisaurus),(Bagualosaurus,(Jaklapalisaurus,(Nambalia,((Thecodontosaurus,Pantydraco),(Efraasia,(Plateosauravus,(Ruehleia,(((Unaysaurus,(Pradhania,Macrocollum)),((Sarahsaurus,((Ngwevu,((Coloradisaurus,(Glacialisaurus,Lufengosaurus)),(Xixipiosaurus,(Massospondylus_carinatus,(Adeopapposaurus,Leyesaurus))))),(Yunnanosaurus_huangi,((Seitaad,(Anchisaurus,((Yizhousaurus,((Sefapanosaurus,Aardonyx),(NMQR1551,(NMRQ3314,(Blikanasaurus,((Camelotia,((Meroktenos,(Ledumahadi,Kholumolumo)),(Lessemsaurus,(Ingentia,Antetonitrus)))),((Schleitheimia,(Isanosaurus,((Tazoudasaurus,Vulcanodon),((Shunosaurus,(Spinophorosaurus,(Mamenchisaurus,(Omeisaurus,(Cetiosaurus,Neosauropoda))))),(Patagosaurus,Barapasaurus))))),(Pulanesaura,Gongxianosaurus)))))))),(Irisosaurus,(Mussaurus,Leonerasaurus))))),(Xingxiulong,(Jingshanosaurus,Chuxiongosaurus)))))),(Eucnemesaurus_fortis,(Riojasaurus,Eucnemesaurus_entaxonis)))),(Plateosaurus_gracilis,(Plateosaurus_ingens,Plateosaurus_engelhardti))))))))))))))))),(Staurikosaurus,Herrerasaurus)))))));

(Euparkeria,(Crurotarsi,(Marasuchus,(Silesaurus,(Ornithischia,((Agnosphitys,((Guaibasaurus,(Neotheropoda,Chindesaurus)),(Buriolestes,(Pampadromaeus,(Panphagia,(Eoraptor,((Saturnalia,Chromogisaurus),(Bagualosaurus,(Jaklapalisaurus,(Nambalia,((Thecodontosaurus,Pantydraco),(Efraasia,(Plateosauravus,(Ruehleia,(((Unaysaurus,(Pradhania,Macrocollum)),((Sarahsaurus,((Ngwevu,(Xixipiosaurus,((Coloradisaurus,(Glacialisaurus,Lufengosaurus)),(Massospondylus_carinatus,(Adeopapposaurus,Leyesaurus))))),(Yunnanosaurus_huangi,((Seitaad,(Anchisaurus,((Yizhousaurus,((Sefapanosaurus,Aardonyx),(NMQR1551,(NMRQ3314,(Blikanasaurus,((Camelotia,((Meroktenos,(Ledumahadi,Kholumolumo)),(Lessemsaurus,(Ingentia,Antetonitrus)))),((Schleitheimia,(Isanosaurus,((Tazoudasaurus,Vulcanodon),((Shunosaurus,(Spinophorosaurus,(Omeisaurus,(Mamenchisaurus,(Cetiosaurus,Neosauropoda))))),(Patagosaurus,Barapasaurus))))),(Pulanesaura,Gongxianosaurus)))))))),(Irisosaurus,(Mussaurus,Leonerasaurus))))),(Xingxiulong,(Jingshanosaurus,Chuxiongosaurus)))))),(Eucnemesaurus_fortis,(Riojasaurus,Eucnemesaurus_entaxonis)))),(Plateosaurus_gracilis,(Plateosaurus_ingens,Plateosaurus_engelhardti))))))))))))))))),(Staurikosaurus,Herrerasaurus)))))));

(Euparkeria,(Crurotarsi,(Marasuchus,(Silesaurus,(Ornithischia,((Agnosphitys,((Guaibasaurus,(Neotheropoda,Chindesaurus)),(Buriolestes,(Pampadromaeus,(Panphagia,(Eoraptor,((Saturnalia,Chromogisaurus),(Bagualosaurus,(Jaklapalisaurus,(Nambalia,((Thecodontosaurus,Pantydraco),(Efraasia,(Plateosauravus,(Ruehleia,(((Unaysaurus,(Pradhania,Macrocollum)),((Sarahsaurus,((Ngwevu,(Xixipiosaurus,((Coloradisaurus,(Glacialisaurus,Lufengosaurus)),(Massospondylus_carinatus,(Adeopapposaurus,Leyesaurus))))),(Yunnanosaurus_huangi,((Seitaad,(Anchisaurus,((Yizhousaurus,((Sefapanosaurus,Aardonyx),(NMQR1551,(NMRQ3314,(Blikanasaurus,((Camelotia,((Meroktenos,(Ledumahadi,Kholumolumo)),(Lessemsaurus,(Ingentia,Antetonitrus)))),((Schleitheimia,(Isanosaurus,((Tazoudasaurus,Vulcanodon),((Shunosaurus,((Spinophorosaurus,Omeisaurus),(Mamenchisaurus,(Cetiosaurus,Neosauropoda)))),(Patagosaurus,Barapasaurus))))),(Pulanesaura,Gongxianosaurus)))))))),(Irisosaurus,(Mussaurus,Leonerasaurus))))),(Xingxiulong,(Jingshanosaurus,Chuxiongosaurus)))))),(Eucnemesaurus_fortis,(Riojasaurus,Eucnemesaurus_entaxonis)))),(Plateosaurus_gracilis,(Plateosaurus_ingens,Plateosaurus_engelhardti))))))))))))))))),(Staurikosaurus,Herrerasaurus)))))));

(Euparkeria,(Crurotarsi,(Marasuchus,(Silesaurus,(Ornithischia,((Agnosphitys,((Guaibasaurus,(Neotheropoda,Chindesaurus)),((Eoraptor,((Saturnalia,Chromogisaurus),(Bagualosaurus,(Jaklapalisaurus,(Nambalia,((Thecodontosaurus,Pantydraco),(Efraasia,(Plateosauravus,(Ruehleia,(((Unaysaurus,(Pradhania,Macrocollum)),((Sarahsaurus,((Ngwevu,(Xixipiosaurus,((Coloradisaurus,(Glacialisaurus,Lufengosaurus)),(Massospondylus_carinatus,(Adeopapposaurus,Leyesaurus))))),(Yunnanosaurus_huangi,((Seitaad,(Anchisaurus,((Yizhousaurus,((Sefapanosaurus,Aardonyx),(NMQR1551,(NMRQ3314,(Blikanasaurus,((Camelotia,((Meroktenos,(Ledumahadi,Kholumolumo)),(Lessemsaurus,(Ingentia,Antetonitrus)))),((Schleitheimia,(Isanosaurus,((Tazoudasaurus,Vulcanodon),((Shunosaurus,(Spinophorosaurus,(Mamenchisaurus,(Omeisaurus,(Cetiosaurus,Neosauropoda))))),(Patagosaurus,Barapasaurus))))),(Pulanesaura,Gongxianosaurus)))))))),(Irisosaurus,(Mussaurus,Leonerasaurus))))),(Xingxiulong,(Jingshanosaurus,Chuxiongosaurus)))))),(Eucnemesaurus_fortis,(Riojasaurus,Eucnemesaurus_entaxonis)))),(Plateosaurus_gracilis,(Plateosaurus_ingens,Plateosaurus_engelhardti)))))))))))),(Buriolestes,(Panphagia,Pampadromaeus))))),(Staurikosaurus,Herrerasaurus)))))));

(Euparkeria,(Crurotarsi,(Marasuchus,(Silesaurus,(Agnosphitys,(Ornithischia,(((Guaibasaurus,(Neotheropoda,Chindesaurus)),((Panphagia,(Eoraptor,((Saturnalia,Chromogisaurus),(Bagualosaurus,(Jaklapalisaurus,(Nambalia,((Thecodontosaurus,Pantydraco),(Efraasia,(Plateosauravus,(Ruehleia,(((Unaysaurus,(Pradhania,Macrocollum)),((Sarahsaurus,((Ngwevu,((Coloradisaurus,(Glacialisaurus,Lufengosaurus)),(Xixipiosaurus,(Massospondylus_carinatus,(Adeopapposaurus,Leyesaurus))))),(Yunnanosaurus_huangi,((Seitaad,(Anchisaurus,((Yizhousaurus,((Sefapanosaurus,Aardonyx),(NMQR1551,(NMRQ3314,(Blikanasaurus,((Camelotia,((Meroktenos,(Ledumahadi,Kholumolumo)),(Lessemsaurus,(Ingentia,Antetonitrus)))),((Schleitheimia,(Isanosaurus,((Tazoudasaurus,Vulcanodon),((Shunosaurus,(Spinophorosaurus,(Mamenchisaurus,(Omeisaurus,(Cetiosaurus,Neosauropoda))))),(Patagosaurus,Barapasaurus))))),(Pulanesaura,Gongxianosaurus)))))))),(Irisosaurus,(Mussaurus,Leonerasaurus))))),(Xingxiulong,(Jingshanosaurus,Chuxiongosaurus)))))),(Eucnemesaurus_fortis,(Riojasaurus,Eucnemesaurus_entaxonis)))),(Plateosaurus_gracilis,(Plateosaurus_ingens,Plateosaurus_engelhardti))))))))))))),(Buriolestes,Pampadromaeus))),(Staurikosaurus,Herrerasaurus))))))));

(Euparkeria,(Crurotarsi,(Marasuchus,(Silesaurus,(Agnosphitys,(Ornithischia,(((Guaibasaurus,(Neotheropoda,Chindesaurus)),((Panphagia,(Eoraptor,((Saturnalia,Chromogisaurus),(Bagualosaurus,(Jaklapalisaurus,(Nambalia,((Thecodontosaurus,Pantydraco),(Efraasia,(Plateosauravus,(Ruehleia,(((Unaysaurus,(Pradhania,Macrocollum)),((Sarahsaurus,((Ngwevu,(Xixipiosaurus,((Coloradisaurus,(Glacialisaurus,Lufengosaurus)),(Massospondylus_carinatus,(Adeopapposaurus,Leyesaurus))))),(Yunnanosaurus_huangi,((Seitaad,(Anchisaurus,((Yizhousaurus,((Sefapanosaurus,Aardonyx),(NMQR1551,(NMRQ3314,(Blikanasaurus,((Camelotia,((Meroktenos,(Ledumahadi,Kholumolumo)),(Lessemsaurus,(Ingentia,Antetonitrus)))),((Schleitheimia,(Isanosaurus,((Tazoudasaurus,Vulcanodon),((Shunosaurus,(Spinophorosaurus,(Omeisaurus,(Mamenchisaurus,(Cetiosaurus,Neosauropoda))))),(Patagosaurus,Barapasaurus))))),(Pulanesaura,Gongxianosaurus)))))))),(Irisosaurus,(Mussaurus,Leonerasaurus))))),(Xingxiulong,(Jingshanosaurus,Chuxiongosaurus)))))),(Eucnemesaurus_fortis,(Riojasaurus,Eucnemesaurus_entaxonis)))),(Plateosaurus_gracilis,(Plateosaurus_ingens,Plateosaurus_engelhardti))))))))))))),(Buriolestes,Pampadromaeus))),(Staurikosaurus,Herrerasaurus))))))));

(Euparkeria,(Crurotarsi,(Marasuchus,(Silesaurus,(Agnosphitys,(Ornithischia,(((Guaibasaurus,(Neotheropoda,Chindesaurus)),((Panphagia,(Eoraptor,((Saturnalia,Chromogisaurus),(Bagualosaurus,(Jaklapalisaurus,(Nambalia,((Thecodontosaurus,Pantydraco),(Efraasia,(Plateosauravus,(Ruehleia,(((Unaysaurus,(Pradhania,Macrocollum)),((Sarahsaurus,((Ngwevu,(Xixipiosaurus,((Coloradisaurus,(Glacialisaurus,Lufengosaurus)),(Massospondylus_carinatus,(Adeopapposaurus,Leyesaurus))))),(Yunnanosaurus_huangi,((Seitaad,(Anchisaurus,((Yizhousaurus,((Sefapanosaurus,Aardonyx),(NMQR1551,(NMRQ3314,(Blikanasaurus,((Camelotia,((Meroktenos,(Ledumahadi,Kholumolumo)),(Lessemsaurus,(Ingentia,Antetonitrus)))),((Schleitheimia,(Isanosaurus,((Tazoudasaurus,Vulcanodon),((Shunosaurus,((Spinophorosaurus,Omeisaurus),(Mamenchisaurus,(Cetiosaurus,Neosauropoda)))),(Patagosaurus,Barapasaurus))))),(Pulanesaura,Gongxianosaurus)))))))),(Irisosaurus,(Mussaurus,Leonerasaurus))))),(Xingxiulong,(Jingshanosaurus,Chuxiongosaurus)))))),(Eucnemesaurus_fortis,(Riojasaurus,Eucnemesaurus_entaxonis)))),(Plateosaurus_gracilis,(Plateosaurus_ingens,Plateosaurus_engelhardti))))))))))))),(Buriolestes,Pampadromaeus))),(Staurikosaurus,Herrerasaurus))))))));

(Euparkeria,(Crurotarsi,(Marasuchus,(Silesaurus,(Agnosphitys,(Ornithischia,(((Guaibasaurus,(Neotheropoda,Chindesaurus)),((Panphagia,(Eoraptor,((Saturnalia,Chromogisaurus),(Bagualosaurus,(Jaklapalisaurus,(Nambalia,((Thecodontosaurus,Pantydraco),(Efraasia,(Plateosauravus,(Ruehleia,(((Unaysaurus,(Pradhania,Macrocollum)),((Sarahsaurus,((Ngwevu,(Xixipiosaurus,((Coloradisaurus,(Glacialisaurus,Lufengosaurus)),(Massospondylus_carinatus,(Adeopapposaurus,Leyesaurus))))),(Yunnanosaurus_huangi,((Seitaad,(Anchisaurus,((Yizhousaurus,((Sefapanosaurus,Aardonyx),(NMQR1551,(NMRQ3314,(Blikanasaurus,((Camelotia,((Meroktenos,(Ledumahadi,Kholumolumo)),(Lessemsaurus,(Ingentia,Antetonitrus)))),((Schleitheimia,(Isanosaurus,((Tazoudasaurus,Vulcanodon),((Shunosaurus,((Spinophorosaurus,(Omeisaurus,Mamenchisaurus)),(Cetiosaurus,Neosauropoda))),(Patagosaurus,Barapasaurus))))),(Pulanesaura,Gongxianosaurus)))))))),(Irisosaurus,(Mussaurus,Leonerasaurus))))),(Xingxiulong,(Jingshanosaurus,Chuxiongosaurus)))))),(Eucnemesaurus_fortis,(Riojasaurus,Eucnemesaurus_entaxonis)))),(Plateosaurus_gracilis,(Plateosaurus_ingens,Plateosaurus_engelhardti))))))))))))),(Buriolestes,Pampadromaeus))),(Staurikosaurus,Herrerasaurus))))))));

(Euparkeria,(Crurotarsi,(Marasuchus,(Silesaurus,(Agnosphitys,(Ornithischia,(((Guaibasaurus,(Neotheropoda,Chindesaurus)),(Buriolestes,(Pampadromaeus,(Panphagia,(Eoraptor,((Saturnalia,Chromogisaurus),(Bagualosaurus,(Jaklapalisaurus,(Nambalia,((Thecodontosaurus,Pantydraco),(Efraasia,(Plateosauravus,(Ruehleia,(((Unaysaurus,(Pradhania,Macrocollum)),((Sarahsaurus,((Ngwevu,((Coloradisaurus,(Glacialisaurus,Lufengosaurus)),(Xixipiosaurus,(Massospondylus_carinatus,(Adeopapposaurus,Leyesaurus))))),(Yunnanosaurus_huangi,((Seitaad,(Anchisaurus,((Yizhousaurus,((Sefapanosaurus,Aardonyx),(NMQR1551,(NMRQ3314,(Blikanasaurus,((Camelotia,((Meroktenos,(Ledumahadi,Kholumolumo)),(Lessemsaurus,(Ingentia,Antetonitrus)))),((Schleitheimia,(Isanosaurus,((Tazoudasaurus,Vulcanodon),((Shunosaurus,(Spinophorosaurus,(Omeisaurus,(Mamenchisaurus,(Cetiosaurus,Neosauropoda))))),(Patagosaurus,Barapasaurus))))),(Pulanesaura,Gongxianosaurus)))))))),(Irisosaurus,(Mussaurus,Leonerasaurus))))),(Xingxiulong,(Jingshanosaurus,Chuxiongosaurus)))))),(Eucnemesaurus_fortis,(Riojasaurus,Eucnemesaurus_entaxonis)))),(Plateosaurus_gracilis,(Plateosaurus_ingens,Plateosaurus_engelhardti)))))))))))))))),(Staurikosaurus,Herrerasaurus))))))));

(Euparkeria,(Crurotarsi,(Marasuchus,(Silesaurus,(Agnosphitys,(Ornithischia,(((Guaibasaurus,(Neotheropoda,Chindesaurus)),(Buriolestes,(Pampadromaeus,(Panphagia,(Eoraptor,((Saturnalia,Chromogisaurus),(Bagualosaurus,(Jaklapalisaurus,(Nambalia,((Thecodontosaurus,Pantydraco),(Efraasia,(Plateosauravus,(Ruehleia,(((Unaysaurus,(Pradhania,Macrocollum)),((Sarahsaurus,((Ngwevu,((Coloradisaurus,(Glacialisaurus,Lufengosaurus)),(Xixipiosaurus,(Massospondylus_carinatus,(Adeopapposaurus,Leyesaurus))))),(Yunnanosaurus_huangi,((Seitaad,(Anchisaurus,((Yizhousaurus,((Sefapanosaurus,Aardonyx),(NMQR1551,(NMRQ3314,(Blikanasaurus,((Camelotia,((Meroktenos,(Ledumahadi,Kholumolumo)),(Lessemsaurus,(Ingentia,Antetonitrus)))),((Schleitheimia,(Isanosaurus,((Tazoudasaurus,Vulcanodon),((Shunosaurus,((Spinophorosaurus,Omeisaurus),(Mamenchisaurus,(Cetiosaurus,Neosauropoda)))),(Patagosaurus,Barapasaurus))))),(Pulanesaura,Gongxianosaurus)))))))),(Irisosaurus,(Mussaurus,Leonerasaurus))))),(Xingxiulong,(Jingshanosaurus,Chuxiongosaurus)))))),(Eucnemesaurus_fortis,(Riojasaurus,Eucnemesaurus_entaxonis)))),(Plateosaurus_gracilis,(Plateosaurus_ingens,Plateosaurus_engelhardti)))))))))))))))),(Staurikosaurus,Herrerasaurus))))))));

(Euparkeria,(Crurotarsi,(Marasuchus,(Silesaurus,(Agnosphitys,(Ornithischia,(((Guaibasaurus,(Neotheropoda,Chindesaurus)),(Buriolestes,(Pampadromaeus,(Panphagia,(Eoraptor,((Saturnalia,Chromogisaurus),(Bagualosaurus,(Jaklapalisaurus,(Nambalia,((Thecodontosaurus,Pantydraco),(Efraasia,(Plateosauravus,(Ruehleia,(((Unaysaurus,(Pradhania,Macrocollum)),((Sarahsaurus,((Ngwevu,((Coloradisaurus,(Glacialisaurus,Lufengosaurus)),(Xixipiosaurus,(Massospondylus_carinatus,(Adeopapposaurus,Leyesaurus))))),(Yunnanosaurus_huangi,((Seitaad,(Anchisaurus,((Yizhousaurus,((Sefapanosaurus,Aardonyx),(NMQR1551,(NMRQ3314,(Blikanasaurus,((Camelotia,((Meroktenos,(Ledumahadi,Kholumolumo)),(Lessemsaurus,(Ingentia,Antetonitrus)))),((Schleitheimia,(Isanosaurus,((Tazoudasaurus,Vulcanodon),((Shunosaurus,((Spinophorosaurus,(Omeisaurus,Mamenchisaurus)),(Cetiosaurus,Neosauropoda))),(Patagosaurus,Barapasaurus))))),(Pulanesaura,Gongxianosaurus)))))))),(Irisosaurus,(Mussaurus,Leonerasaurus))))),(Xingxiulong,(Jingshanosaurus,Chuxiongosaurus)))))),(Eucnemesaurus_fortis,(Riojasaurus,Eucnemesaurus_entaxonis)))),(Plateosaurus_gracilis,(Plateosaurus_ingens,Plateosaurus_engelhardti)))))))))))))))),(Staurikosaurus,Herrerasaurus))))))));

(Euparkeria,(Crurotarsi,(Marasuchus,(Silesaurus,(Agnosphitys,(Ornithischia,(((Guaibasaurus,(Neotheropoda,Chindesaurus)),(Buriolestes,(Pampadromaeus,(Panphagia,(Eoraptor,((Saturnalia,Chromogisaurus),(Bagualosaurus,(Jaklapalisaurus,(Nambalia,((Thecodontosaurus,Pantydraco),(Efraasia,(Plateosauravus,(Ruehleia,(((Unaysaurus,(Pradhania,Macrocollum)),((Sarahsaurus,((Ngwevu,(Xixipiosaurus,((Coloradisaurus,(Glacialisaurus,Lufengosaurus)),(Massospondylus_carinatus,(Adeopapposaurus,Leyesaurus))))),(Yunnanosaurus_huangi,((Seitaad,(Anchisaurus,((Yizhousaurus,((Sefapanosaurus,Aardonyx),(NMQR1551,(NMRQ3314,((Camelotia,((Meroktenos,(Ledumahadi,Kholumolumo)),(Lessemsaurus,(Blikanasaurus,(Ingentia,Antetonitrus))))),((Schleitheimia,(Isanosaurus,((Tazoudasaurus,Vulcanodon),((Shunosaurus,(Spinophorosaurus,(Omeisaurus,(Mamenchisaurus,(Cetiosaurus,Neosauropoda))))),(Patagosaurus,Barapasaurus))))),(Pulanesaura,Gongxianosaurus))))))),(Irisosaurus,(Mussaurus,Leonerasaurus))))),(Xingxiulong,(Jingshanosaurus,Chuxiongosaurus)))))),(Eucnemesaurus_fortis,(Riojasaurus,Eucnemesaurus_entaxonis)))),(Plateosaurus_gracilis,(Plateosaurus_ingens,Plateosaurus_engelhardti)))))))))))))))),(Staurikosaurus,Herrerasaurus))))))));

(Euparkeria,(Crurotarsi,(Marasuchus,(Silesaurus,(Ornithischia,(Agnosphitys,(((Guaibasaurus,(Neotheropoda,Chindesaurus)),((Panphagia,(Eoraptor,((Saturnalia,Chromogisaurus),(Bagualosaurus,(Jaklapalisaurus,(Nambalia,((Thecodontosaurus,Pantydraco),(Efraasia,(Plateosauravus,(Ruehleia,(((Unaysaurus,(Pradhania,Macrocollum)),((Sarahsaurus,((Ngwevu,(Xixipiosaurus,((Coloradisaurus,(Glacialisaurus,Lufengosaurus)),(Massospondylus_carinatus,(Adeopapposaurus,Leyesaurus))))),(Yunnanosaurus_huangi,((Seitaad,(Anchisaurus,((Yizhousaurus,((Sefapanosaurus,Aardonyx),(NMQR1551,(NMRQ3314,(Blikanasaurus,((Camelotia,((Meroktenos,(Ledumahadi,Kholumolumo)),(Lessemsaurus,(Ingentia,Antetonitrus)))),(Pulanesaura,(Gongxianosaurus,(Schleitheimia,(Isanosaurus,((Tazoudasaurus,Vulcanodon),((Shunosaurus,((Spinophorosaurus,(Omeisaurus,Mamenchisaurus)),(Cetiosaurus,Neosauropoda))),(Patagosaurus,Barapasaurus))))))))))))),(Irisosaurus,(Mussaurus,Leonerasaurus))))),(Xingxiulong,(Jingshanosaurus,Chuxiongosaurus)))))),(Eucnemesaurus_fortis,(Riojasaurus,Eucnemesaurus_entaxonis)))),(Plateosaurus_gracilis,(Plateosaurus_ingens,Plateosaurus_engelhardti))))))))))))),(Buriolestes,Pampadromaeus))),(Staurikosaurus,Herrerasaurus))))))));

(Euparkeria,(Crurotarsi,(Marasuchus,(Silesaurus,(Ornithischia,(Agnosphitys,(((Guaibasaurus,(Neotheropoda,Chindesaurus)),(Buriolestes,(Pampadromaeus,(Panphagia,(Eoraptor,((Saturnalia,Chromogisaurus),(Bagualosaurus,(Jaklapalisaurus,(Nambalia,((Thecodontosaurus,Pantydraco),(Efraasia,(Plateosauravus,(Ruehleia,(((Unaysaurus,(Pradhania,Macrocollum)),((Sarahsaurus,((Ngwevu,((Coloradisaurus,(Glacialisaurus,Lufengosaurus)),(Xixipiosaurus,(Massospondylus_carinatus,(Adeopapposaurus,Leyesaurus))))),(Yunnanosaurus_huangi,((Seitaad,(Anchisaurus,((Yizhousaurus,((Sefapanosaurus,Aardonyx),(NMQR1551,(NMRQ3314,(Blikanasaurus,((Camelotia,((Meroktenos,(Ledumahadi,Kholumolumo)),(Lessemsaurus,(Ingentia,Antetonitrus)))),(Pulanesaura,(Gongxianosaurus,(Schleitheimia,(Isanosaurus,((Tazoudasaurus,Vulcanodon),((Shunosaurus,((Spinophorosaurus,(Omeisaurus,Mamenchisaurus)),(Cetiosaurus,Neosauropoda))),(Patagosaurus,Barapasaurus))))))))))))),(Irisosaurus,(Mussaurus,Leonerasaurus))))),(Xingxiulong,(Jingshanosaurus,Chuxiongosaurus)))))),(Eucnemesaurus_fortis,(Riojasaurus,Eucnemesaurus_entaxonis)))),(Plateosaurus_gracilis,(Plateosaurus_ingens,Plateosaurus_engelhardti)))))))))))))))),(Staurikosaurus,Herrerasaurus))))))));

(Euparkeria,(Crurotarsi,(Marasuchus,(Silesaurus,(Ornithischia,((Agnosphitys,((Guaibasaurus,(Neotheropoda,Chindesaurus)),((Panphagia,(Eoraptor,((Saturnalia,Chromogisaurus),(Bagualosaurus,(Jaklapalisaurus,(Nambalia,((Thecodontosaurus,Pantydraco),(Efraasia,(Plateosauravus,(Ruehleia,(((Unaysaurus,(Pradhania,Macrocollum)),((Sarahsaurus,((Ngwevu,(Xixipiosaurus,((Coloradisaurus,(Glacialisaurus,Lufengosaurus)),(Massospondylus_carinatus,(Adeopapposaurus,Leyesaurus))))),(Yunnanosaurus_huangi,((Seitaad,(Anchisaurus,((Yizhousaurus,((Sefapanosaurus,Aardonyx),(NMQR1551,(NMRQ3314,(Blikanasaurus,((Camelotia,((Meroktenos,(Ledumahadi,Kholumolumo)),(Lessemsaurus,(Ingentia,Antetonitrus)))),(Pulanesaura,(Gongxianosaurus,(Schleitheimia,(Isanosaurus,((Tazoudasaurus,Vulcanodon),((Shunosaurus,((Spinophorosaurus,(Omeisaurus,Mamenchisaurus)),(Cetiosaurus,Neosauropoda))),(Patagosaurus,Barapasaurus))))))))))))),(Irisosaurus,(Mussaurus,Leonerasaurus))))),(Xingxiulong,(Jingshanosaurus,Chuxiongosaurus)))))),(Eucnemesaurus_fortis,(Riojasaurus,Eucnemesaurus_entaxonis)))),(Plateosaurus_gracilis,(Plateosaurus_ingens,Plateosaurus_engelhardti))))))))))))),(Buriolestes,Pampadromaeus)))),(Staurikosaurus,Herrerasaurus)))))));

(Euparkeria,(Crurotarsi,(Marasuchus,(Silesaurus,(Ornithischia,((Agnosphitys,((Guaibasaurus,(Neotheropoda,Chindesaurus)),(Buriolestes,((Eoraptor,((Saturnalia,Chromogisaurus),(Bagualosaurus,(Jaklapalisaurus,(Nambalia,((Thecodontosaurus,Pantydraco),(Efraasia,(Plateosauravus,(Ruehleia,(((Unaysaurus,(Pradhania,Macrocollum)),((Sarahsaurus,((Ngwevu,(Xixipiosaurus,((Coloradisaurus,(Glacialisaurus,Lufengosaurus)),(Massospondylus_carinatus,(Adeopapposaurus,Leyesaurus))))),(Yunnanosaurus_huangi,((Seitaad,(Anchisaurus,((Yizhousaurus,((Sefapanosaurus,Aardonyx),(NMQR1551,(NMRQ3314,(Blikanasaurus,((Camelotia,((Meroktenos,(Ledumahadi,Kholumolumo)),(Lessemsaurus,(Ingentia,Antetonitrus)))),(Pulanesaura,(Gongxianosaurus,(Schleitheimia,(Isanosaurus,((Tazoudasaurus,Vulcanodon),((Shunosaurus,((Spinophorosaurus,(Omeisaurus,Mamenchisaurus)),(Cetiosaurus,Neosauropoda))),(Patagosaurus,Barapasaurus))))))))))))),(Irisosaurus,(Mussaurus,Leonerasaurus))))),(Xingxiulong,(Jingshanosaurus,Chuxiongosaurus)))))),(Eucnemesaurus_fortis,(Riojasaurus,Eucnemesaurus_entaxonis)))),(Plateosaurus_gracilis,(Plateosaurus_ingens,Plateosaurus_engelhardti)))))))))))),(Panphagia,Pampadromaeus))))),(Staurikosaurus,Herrerasaurus)))))));

(Euparkeria,(Crurotarsi,(Marasuchus,(Silesaurus,(Ornithischia,((Agnosphitys,((Guaibasaurus,(Neotheropoda,Chindesaurus)),(Buriolestes,(Panphagia,(Eoraptor,((Saturnalia,Chromogisaurus),(Pampadromaeus,(Bagualosaurus,(Jaklapalisaurus,(Nambalia,((Thecodontosaurus,Pantydraco),(Efraasia,(Plateosauravus,(Ruehleia,(((Unaysaurus,(Pradhania,Macrocollum)),((Sarahsaurus,((Ngwevu,(Xixipiosaurus,((Coloradisaurus,(Glacialisaurus,Lufengosaurus)),(Massospondylus_carinatus,(Adeopapposaurus,Leyesaurus))))),(Yunnanosaurus_huangi,((Seitaad,(Anchisaurus,((Yizhousaurus,((Sefapanosaurus,Aardonyx),(NMQR1551,(NMRQ3314,(Blikanasaurus,((Camelotia,((Meroktenos,(Ledumahadi,Kholumolumo)),(Lessemsaurus,(Ingentia,Antetonitrus)))),(Pulanesaura,(Gongxianosaurus,(Schleitheimia,(Isanosaurus,((Tazoudasaurus,Vulcanodon),((Shunosaurus,((Spinophorosaurus,(Omeisaurus,Mamenchisaurus)),(Cetiosaurus,Neosauropoda))),(Patagosaurus,Barapasaurus))))))))))))),(Irisosaurus,(Mussaurus,Leonerasaurus))))),(Xingxiulong,(Jingshanosaurus,Chuxiongosaurus)))))),(Eucnemesaurus_fortis,(Riojasaurus,Eucnemesaurus_entaxonis)))),(Plateosaurus_gracilis,(Plateosaurus_ingens,Plateosaurus_engelhardti))))))))))))))))),(Staurikosaurus,Herrerasaurus)))))));

(Euparkeria,(Crurotarsi,(Marasuchus,(Silesaurus,(Ornithischia,((Agnosphitys,((Guaibasaurus,(Neotheropoda,Chindesaurus)),(Buriolestes,(Pampadromaeus,(Panphagia,(Eoraptor,((Saturnalia,Chromogisaurus),(Bagualosaurus,(Jaklapalisaurus,(Nambalia,((Thecodontosaurus,Pantydraco),(Efraasia,(Plateosauravus,(Ruehleia,(((Unaysaurus,(Pradhania,Macrocollum)),((Sarahsaurus,((Ngwevu,((Coloradisaurus,(Glacialisaurus,Lufengosaurus)),(Xixipiosaurus,(Massospondylus_carinatus,(Adeopapposaurus,Leyesaurus))))),(Yunnanosaurus_huangi,((Seitaad,(Anchisaurus,((Yizhousaurus,((Sefapanosaurus,Aardonyx),(NMQR1551,(NMRQ3314,(Blikanasaurus,((Camelotia,((Meroktenos,(Ledumahadi,Kholumolumo)),(Lessemsaurus,(Ingentia,Antetonitrus)))),(Pulanesaura,(Gongxianosaurus,(Schleitheimia,(Isanosaurus,((Tazoudasaurus,Vulcanodon),((Shunosaurus,((Spinophorosaurus,(Omeisaurus,Mamenchisaurus)),(Cetiosaurus,Neosauropoda))),(Patagosaurus,Barapasaurus))))))))))))),(Irisosaurus,(Mussaurus,Leonerasaurus))))),(Xingxiulong,(Jingshanosaurus,Chuxiongosaurus)))))),(Eucnemesaurus_fortis,(Riojasaurus,Eucnemesaurus_entaxonis)))),(Plateosaurus_gracilis,(Plateosaurus_ingens,Plateosaurus_engelhardti))))))))))))))))),(Staurikosaurus,Herrerasaurus)))))));

(Euparkeria,(Crurotarsi,(Marasuchus,(Silesaurus,(Ornithischia,((Agnosphitys,((Guaibasaurus,(Neotheropoda,Chindesaurus)),((Eoraptor,((Saturnalia,Chromogisaurus),(Bagualosaurus,(Jaklapalisaurus,(Nambalia,((Thecodontosaurus,Pantydraco),(Efraasia,(Plateosauravus,(Ruehleia,(((Unaysaurus,(Pradhania,Macrocollum)),((Sarahsaurus,((Ngwevu,(Xixipiosaurus,((Coloradisaurus,(Glacialisaurus,Lufengosaurus)),(Massospondylus_carinatus,(Adeopapposaurus,Leyesaurus))))),(Yunnanosaurus_huangi,((Seitaad,(Anchisaurus,((Yizhousaurus,((Sefapanosaurus,Aardonyx),(NMQR1551,(NMRQ3314,(Blikanasaurus,((Camelotia,((Meroktenos,(Ledumahadi,Kholumolumo)),(Lessemsaurus,(Ingentia,Antetonitrus)))),(Pulanesaura,(Gongxianosaurus,(Schleitheimia,(Isanosaurus,((Tazoudasaurus,Vulcanodon),((Shunosaurus,((Spinophorosaurus,(Omeisaurus,Mamenchisaurus)),(Cetiosaurus,Neosauropoda))),(Patagosaurus,Barapasaurus))))))))))))),(Irisosaurus,(Mussaurus,Leonerasaurus))))),(Xingxiulong,(Jingshanosaurus,Chuxiongosaurus)))))),(Eucnemesaurus_fortis,(Riojasaurus,Eucnemesaurus_entaxonis)))),(Plateosaurus_gracilis,(Plateosaurus_ingens,Plateosaurus_engelhardti)))))))))))),(Buriolestes,(Panphagia,Pampadromaeus))))),(Staurikosaurus,Herrerasaurus)))))));

(Euparkeria,(Crurotarsi,(Marasuchus,(Silesaurus,(Agnosphitys,(Ornithischia,(((Guaibasaurus,(Neotheropoda,Chindesaurus)),((Panphagia,(Eoraptor,((Saturnalia,Chromogisaurus),(Bagualosaurus,(Jaklapalisaurus,(Nambalia,((Thecodontosaurus,Pantydraco),(Efraasia,(Plateosauravus,(Ruehleia,(((Unaysaurus,(Pradhania,Macrocollum)),((Sarahsaurus,((Ngwevu,((Coloradisaurus,(Glacialisaurus,Lufengosaurus)),(Xixipiosaurus,(Massospondylus_carinatus,(Adeopapposaurus,Leyesaurus))))),(Yunnanosaurus_huangi,((Seitaad,(Anchisaurus,((Yizhousaurus,((Sefapanosaurus,Aardonyx),(NMQR1551,(NMRQ3314,(Blikanasaurus,((Camelotia,((Meroktenos,(Ledumahadi,Kholumolumo)),(Lessemsaurus,(Ingentia,Antetonitrus)))),(Pulanesaura,(Gongxianosaurus,(Schleitheimia,(Isanosaurus,((Tazoudasaurus,Vulcanodon),((Shunosaurus,((Spinophorosaurus,(Omeisaurus,Mamenchisaurus)),(Cetiosaurus,Neosauropoda))),(Patagosaurus,Barapasaurus))))))))))))),(Irisosaurus,(Mussaurus,Leonerasaurus))))),(Xingxiulong,(Jingshanosaurus,Chuxiongosaurus)))))),(Eucnemesaurus_fortis,(Riojasaurus,Eucnemesaurus_entaxonis)))),(Plateosaurus_gracilis,(Plateosaurus_ingens,Plateosaurus_engelhardti))))))))))))),(Buriolestes,Pampadromaeus))),(Staurikosaurus,Herrerasaurus))))))));

(Euparkeria,(Crurotarsi,(Marasuchus,(Silesaurus,(Ornithischia,(Agnosphitys,(((Guaibasaurus,(Neotheropoda,Chindesaurus)),((Panphagia,(Eoraptor,((Saturnalia,Chromogisaurus),(Bagualosaurus,(Jaklapalisaurus,(Nambalia,((Thecodontosaurus,Pantydraco),(Efraasia,(Plateosauravus,(Ruehleia,(((Unaysaurus,(Pradhania,Macrocollum)),((Sarahsaurus,((Ngwevu,((Coloradisaurus,(Glacialisaurus,Lufengosaurus)),(Xixipiosaurus,(Massospondylus_carinatus,(Adeopapposaurus,Leyesaurus))))),(Yunnanosaurus_huangi,((Seitaad,(Anchisaurus,((Yizhousaurus,((Sefapanosaurus,Aardonyx),(NMQR1551,(NMRQ3314,(Blikanasaurus,((Camelotia,((Meroktenos,(Ledumahadi,Kholumolumo)),(Lessemsaurus,(Ingentia,Antetonitrus)))),(Pulanesaura,(Gongxianosaurus,(Schleitheimia,(Isanosaurus,((Tazoudasaurus,Vulcanodon),((Shunosaurus,(Spinophorosaurus,(Mamenchisaurus,(Omeisaurus,(Cetiosaurus,Neosauropoda))))),(Patagosaurus,Barapasaurus))))))))))))),(Irisosaurus,(Mussaurus,Leonerasaurus))))),(Xingxiulong,(Jingshanosaurus,Chuxiongosaurus)))))),(Eucnemesaurus_fortis,(Riojasaurus,Eucnemesaurus_entaxonis)))),(Plateosaurus_gracilis,(Plateosaurus_ingens,Plateosaurus_engelhardti))))))))))))),(Buriolestes,Pampadromaeus))),(Staurikosaurus,Herrerasaurus))))))));

(Euparkeria,(Crurotarsi,(Marasuchus,(Silesaurus,(Ornithischia,(Agnosphitys,(((Guaibasaurus,(Neotheropoda,Chindesaurus)),((Panphagia,(Eoraptor,((Saturnalia,Chromogisaurus),(Bagualosaurus,(Jaklapalisaurus,(Nambalia,((Thecodontosaurus,Pantydraco),(Efraasia,(Plateosauravus,(Ruehleia,(((Unaysaurus,(Pradhania,Macrocollum)),((Sarahsaurus,((Ngwevu,(Xixipiosaurus,((Coloradisaurus,(Glacialisaurus,Lufengosaurus)),(Massospondylus_carinatus,(Adeopapposaurus,Leyesaurus))))),(Yunnanosaurus_huangi,((Seitaad,(Anchisaurus,((Yizhousaurus,((Sefapanosaurus,Aardonyx),(NMQR1551,(NMRQ3314,(Blikanasaurus,((Camelotia,((Meroktenos,(Ledumahadi,Kholumolumo)),(Lessemsaurus,(Ingentia,Antetonitrus)))),(Pulanesaura,(Gongxianosaurus,(Schleitheimia,(Isanosaurus,((Tazoudasaurus,Vulcanodon),((Shunosaurus,(Spinophorosaurus,(Omeisaurus,(Mamenchisaurus,(Cetiosaurus,Neosauropoda))))),(Patagosaurus,Barapasaurus))))))))))))),(Irisosaurus,(Mussaurus,Leonerasaurus))))),(Xingxiulong,(Jingshanosaurus,Chuxiongosaurus)))))),(Eucnemesaurus_fortis,(Riojasaurus,Eucnemesaurus_entaxonis)))),(Plateosaurus_gracilis,(Plateosaurus_ingens,Plateosaurus_engelhardti))))))))))))),(Buriolestes,Pampadromaeus))),(Staurikosaurus,Herrerasaurus))))))));

(Euparkeria,(Crurotarsi,(Marasuchus,(Silesaurus,(Ornithischia,(Agnosphitys,(((Guaibasaurus,(Neotheropoda,Chindesaurus)),((Panphagia,(Eoraptor,((Saturnalia,Chromogisaurus),(Bagualosaurus,(Jaklapalisaurus,(Nambalia,((Thecodontosaurus,Pantydraco),(Efraasia,(Plateosauravus,(Ruehleia,(((Unaysaurus,(Pradhania,Macrocollum)),((Sarahsaurus,((Ngwevu,(Xixipiosaurus,((Coloradisaurus,(Glacialisaurus,Lufengosaurus)),(Massospondylus_carinatus,(Adeopapposaurus,Leyesaurus))))),(Yunnanosaurus_huangi,((Seitaad,(Anchisaurus,((Yizhousaurus,((Sefapanosaurus,Aardonyx),(NMQR1551,(NMRQ3314,(Blikanasaurus,((Camelotia,((Meroktenos,(Ledumahadi,Kholumolumo)),(Lessemsaurus,(Ingentia,Antetonitrus)))),(Pulanesaura,(Gongxianosaurus,(Schleitheimia,(Isanosaurus,((Tazoudasaurus,Vulcanodon),((Shunosaurus,((Spinophorosaurus,Omeisaurus),(Mamenchisaurus,(Cetiosaurus,Neosauropoda)))),(Patagosaurus,Barapasaurus))))))))))))),(Irisosaurus,(Mussaurus,Leonerasaurus))))),(Xingxiulong,(Jingshanosaurus,Chuxiongosaurus)))))),(Eucnemesaurus_fortis,(Riojasaurus,Eucnemesaurus_entaxonis)))),(Plateosaurus_gracilis,(Plateosaurus_ingens,Plateosaurus_engelhardti))))))))))))),(Buriolestes,Pampadromaeus))),(Staurikosaurus,Herrerasaurus))))))));

(Euparkeria,(Crurotarsi,(Marasuchus,(Silesaurus,(Ornithischia,(Agnosphitys,(((Guaibasaurus,(Neotheropoda,Chindesaurus)),(Buriolestes,(Pampadromaeus,(Panphagia,(Eoraptor,((Saturnalia,Chromogisaurus),(Bagualosaurus,(Jaklapalisaurus,(Nambalia,((Thecodontosaurus,Pantydraco),(Efraasia,(Plateosauravus,(Ruehleia,(((Unaysaurus,(Pradhania,Macrocollum)),((Sarahsaurus,((Ngwevu,((Coloradisaurus,(Glacialisaurus,Lufengosaurus)),(Xixipiosaurus,(Massospondylus_carinatus,(Adeopapposaurus,Leyesaurus))))),(Yunnanosaurus_huangi,((Seitaad,(Anchisaurus,((Yizhousaurus,((Sefapanosaurus,Aardonyx),(NMQR1551,(NMRQ3314,(Blikanasaurus,((Camelotia,((Meroktenos,(Ledumahadi,Kholumolumo)),(Lessemsaurus,(Ingentia,Antetonitrus)))),(Pulanesaura,(Gongxianosaurus,(Schleitheimia,(Isanosaurus,((Tazoudasaurus,Vulcanodon),((Shunosaurus,(Spinophorosaurus,(Omeisaurus,(Mamenchisaurus,(Cetiosaurus,Neosauropoda))))),(Patagosaurus,Barapasaurus))))))))))))),(Irisosaurus,(Mussaurus,Leonerasaurus))))),(Xingxiulong,(Jingshanosaurus,Chuxiongosaurus)))))),(Eucnemesaurus_fortis,(Riojasaurus,Eucnemesaurus_entaxonis)))),(Plateosaurus_gracilis,(Plateosaurus_ingens,Plateosaurus_engelhardti)))))))))))))))),(Staurikosaurus,Herrerasaurus))))))));

(Euparkeria,(Crurotarsi,(Marasuchus,(Silesaurus,(Ornithischia,(Agnosphitys,(((Guaibasaurus,(Neotheropoda,Chindesaurus)),(Buriolestes,(Pampadromaeus,(Panphagia,(Eoraptor,((Saturnalia,Chromogisaurus),(Bagualosaurus,(Jaklapalisaurus,(Nambalia,((Thecodontosaurus,Pantydraco),(Efraasia,(Plateosauravus,(Ruehleia,(((Unaysaurus,(Pradhania,Macrocollum)),((Sarahsaurus,((Ngwevu,((Coloradisaurus,(Glacialisaurus,Lufengosaurus)),(Xixipiosaurus,(Massospondylus_carinatus,(Adeopapposaurus,Leyesaurus))))),(Yunnanosaurus_huangi,((Seitaad,(Anchisaurus,((Yizhousaurus,((Sefapanosaurus,Aardonyx),(NMQR1551,(NMRQ3314,(Blikanasaurus,((Camelotia,((Meroktenos,(Ledumahadi,Kholumolumo)),(Lessemsaurus,(Ingentia,Antetonitrus)))),(Pulanesaura,(Gongxianosaurus,(Schleitheimia,(Isanosaurus,((Tazoudasaurus,Vulcanodon),((Shunosaurus,((Spinophorosaurus,Omeisaurus),(Mamenchisaurus,(Cetiosaurus,Neosauropoda)))),(Patagosaurus,Barapasaurus))))))))))))),(Irisosaurus,(Mussaurus,Leonerasaurus))))),(Xingxiulong,(Jingshanosaurus,Chuxiongosaurus)))))),(Eucnemesaurus_fortis,(Riojasaurus,Eucnemesaurus_entaxonis)))),(Plateosaurus_gracilis,(Plateosaurus_ingens,Plateosaurus_engelhardti)))))))))))))))),(Staurikosaurus,Herrerasaurus))))))));

(Euparkeria,(Crurotarsi,(Marasuchus,(Silesaurus,(Ornithischia,(Agnosphitys,(((Guaibasaurus,(Neotheropoda,Chindesaurus)),(Buriolestes,(Pampadromaeus,(Panphagia,(Eoraptor,((Saturnalia,Chromogisaurus),(Bagualosaurus,(Jaklapalisaurus,(Nambalia,((Thecodontosaurus,Pantydraco),(Efraasia,(Plateosauravus,(Ruehleia,(((Unaysaurus,(Pradhania,Macrocollum)),((Sarahsaurus,((Ngwevu,(Xixipiosaurus,((Coloradisaurus,(Glacialisaurus,Lufengosaurus)),(Massospondylus_carinatus,(Adeopapposaurus,Leyesaurus))))),(Yunnanosaurus_huangi,((Seitaad,(Anchisaurus,((Yizhousaurus,((Sefapanosaurus,Aardonyx),(NMQR1551,(NMRQ3314,((Camelotia,((Meroktenos,(Ledumahadi,Kholumolumo)),(Lessemsaurus,(Blikanasaurus,(Ingentia,Antetonitrus))))),(Pulanesaura,(Gongxianosaurus,(Schleitheimia,(Isanosaurus,((Tazoudasaurus,Vulcanodon),((Shunosaurus,(Spinophorosaurus,(Omeisaurus,(Mamenchisaurus,(Cetiosaurus,Neosauropoda))))),(Patagosaurus,Barapasaurus)))))))))))),(Irisosaurus,(Mussaurus,Leonerasaurus))))),(Xingxiulong,(Jingshanosaurus,Chuxiongosaurus)))))),(Eucnemesaurus_fortis,(Riojasaurus,Eucnemesaurus_entaxonis)))),(Plateosaurus_gracilis,(Plateosaurus_ingens,Plateosaurus_engelhardti)))))))))))))))),(Staurikosaurus,Herrerasaurus))))))));

(Euparkeria,(Crurotarsi,(Marasuchus,(Silesaurus,(Ornithischia,((Agnosphitys,((Guaibasaurus,(Neotheropoda,Chindesaurus)),((Panphagia,(Eoraptor,((Saturnalia,Chromogisaurus),(Bagualosaurus,(Jaklapalisaurus,(Nambalia,((Thecodontosaurus,Pantydraco),(Efraasia,(Plateosauravus,(Ruehleia,(((Unaysaurus,(Pradhania,Macrocollum)),((Sarahsaurus,((Ngwevu,((Coloradisaurus,(Glacialisaurus,Lufengosaurus)),(Xixipiosaurus,(Massospondylus_carinatus,(Adeopapposaurus,Leyesaurus))))),(Yunnanosaurus_huangi,((Seitaad,(Anchisaurus,((Yizhousaurus,((Sefapanosaurus,Aardonyx),(NMQR1551,(NMRQ3314,(Blikanasaurus,((Camelotia,((Meroktenos,(Ledumahadi,Kholumolumo)),(Lessemsaurus,(Ingentia,Antetonitrus)))),(Pulanesaura,(Gongxianosaurus,(Schleitheimia,(Isanosaurus,((Tazoudasaurus,Vulcanodon),((Shunosaurus,(Spinophorosaurus,(Mamenchisaurus,(Omeisaurus,(Cetiosaurus,Neosauropoda))))),(Patagosaurus,Barapasaurus))))))))))))),(Irisosaurus,(Mussaurus,Leonerasaurus))))),(Xingxiulong,(Jingshanosaurus,Chuxiongosaurus)))))),(Eucnemesaurus_fortis,(Riojasaurus,Eucnemesaurus_entaxonis)))),(Plateosaurus_gracilis,(Plateosaurus_ingens,Plateosaurus_engelhardti))))))))))))),(Buriolestes,Pampadromaeus)))),(Staurikosaurus,Herrerasaurus)))))));

(Euparkeria,(Crurotarsi,(Marasuchus,(Silesaurus,(Ornithischia,((Agnosphitys,((Guaibasaurus,(Neotheropoda,Chindesaurus)),((Panphagia,(Eoraptor,((Saturnalia,Chromogisaurus),(Bagualosaurus,(Jaklapalisaurus,(Nambalia,((Thecodontosaurus,Pantydraco),(Efraasia,(Plateosauravus,(Ruehleia,(((Unaysaurus,(Pradhania,Macrocollum)),((Sarahsaurus,((Ngwevu,(Xixipiosaurus,((Coloradisaurus,(Glacialisaurus,Lufengosaurus)),(Massospondylus_carinatus,(Adeopapposaurus,Leyesaurus))))),(Yunnanosaurus_huangi,((Seitaad,(Anchisaurus,((Yizhousaurus,((Sefapanosaurus,Aardonyx),(NMQR1551,(NMRQ3314,(Blikanasaurus,((Camelotia,((Meroktenos,(Ledumahadi,Kholumolumo)),(Lessemsaurus,(Ingentia,Antetonitrus)))),(Pulanesaura,(Gongxianosaurus,(Schleitheimia,(Isanosaurus,((Tazoudasaurus,Vulcanodon),((Shunosaurus,(Spinophorosaurus,(Omeisaurus,(Mamenchisaurus,(Cetiosaurus,Neosauropoda))))),(Patagosaurus,Barapasaurus))))))))))))),(Irisosaurus,(Mussaurus,Leonerasaurus))))),(Xingxiulong,(Jingshanosaurus,Chuxiongosaurus)))))),(Eucnemesaurus_fortis,(Riojasaurus,Eucnemesaurus_entaxonis)))),(Plateosaurus_gracilis,(Plateosaurus_ingens,Plateosaurus_engelhardti))))))))))))),(Buriolestes,Pampadromaeus)))),(Staurikosaurus,Herrerasaurus)))))));

(Euparkeria,(Crurotarsi,(Marasuchus,(Silesaurus,(Ornithischia,((Agnosphitys,((Guaibasaurus,(Neotheropoda,Chindesaurus)),((Panphagia,(Eoraptor,((Saturnalia,Chromogisaurus),(Bagualosaurus,(Jaklapalisaurus,(Nambalia,((Thecodontosaurus,Pantydraco),(Efraasia,(Plateosauravus,(Ruehleia,(((Unaysaurus,(Pradhania,Macrocollum)),((Sarahsaurus,((Ngwevu,(Xixipiosaurus,((Coloradisaurus,(Glacialisaurus,Lufengosaurus)),(Massospondylus_carinatus,(Adeopapposaurus,Leyesaurus))))),(Yunnanosaurus_huangi,((Seitaad,(Anchisaurus,((Yizhousaurus,((Sefapanosaurus,Aardonyx),(NMQR1551,(NMRQ3314,(Blikanasaurus,((Camelotia,((Meroktenos,(Ledumahadi,Kholumolumo)),(Lessemsaurus,(Ingentia,Antetonitrus)))),(Pulanesaura,(Gongxianosaurus,(Schleitheimia,(Isanosaurus,((Tazoudasaurus,Vulcanodon),((Shunosaurus,((Spinophorosaurus,Omeisaurus),(Mamenchisaurus,(Cetiosaurus,Neosauropoda)))),(Patagosaurus,Barapasaurus))))))))))))),(Irisosaurus,(Mussaurus,Leonerasaurus))))),(Xingxiulong,(Jingshanosaurus,Chuxiongosaurus)))))),(Eucnemesaurus_fortis,(Riojasaurus,Eucnemesaurus_entaxonis)))),(Plateosaurus_gracilis,(Plateosaurus_ingens,Plateosaurus_engelhardti))))))))))))),(Buriolestes,Pampadromaeus)))),(Staurikosaurus,Herrerasaurus)))))));

(Euparkeria,(Crurotarsi,(Marasuchus,(Silesaurus,(Ornithischia,((Agnosphitys,((Guaibasaurus,(Neotheropoda,Chindesaurus)),(Buriolestes,((Eoraptor,((Saturnalia,Chromogisaurus),(Bagualosaurus,(Jaklapalisaurus,(Nambalia,((Thecodontosaurus,Pantydraco),(Efraasia,(Plateosauravus,(Ruehleia,(((Unaysaurus,(Pradhania,Macrocollum)),((Sarahsaurus,((Ngwevu,((Coloradisaurus,(Glacialisaurus,Lufengosaurus)),(Xixipiosaurus,(Massospondylus_carinatus,(Adeopapposaurus,Leyesaurus))))),(Yunnanosaurus_huangi,((Seitaad,(Anchisaurus,((Yizhousaurus,((Sefapanosaurus,Aardonyx),(NMQR1551,(NMRQ3314,(Blikanasaurus,((Camelotia,((Meroktenos,(Ledumahadi,Kholumolumo)),(Lessemsaurus,(Ingentia,Antetonitrus)))),(Pulanesaura,(Gongxianosaurus,(Schleitheimia,(Isanosaurus,((Tazoudasaurus,Vulcanodon),((Shunosaurus,(Spinophorosaurus,(Mamenchisaurus,(Omeisaurus,(Cetiosaurus,Neosauropoda))))),(Patagosaurus,Barapasaurus))))))))))))),(Irisosaurus,(Mussaurus,Leonerasaurus))))),(Xingxiulong,(Jingshanosaurus,Chuxiongosaurus)))))),(Eucnemesaurus_fortis,(Riojasaurus,Eucnemesaurus_entaxonis)))),(Plateosaurus_gracilis,(Plateosaurus_ingens,Plateosaurus_engelhardti)))))))))))),(Panphagia,Pampadromaeus))))),(Staurikosaurus,Herrerasaurus)))))));

(Euparkeria,(Crurotarsi,(Marasuchus,(Silesaurus,(Ornithischia,((Agnosphitys,((Guaibasaurus,(Neotheropoda,Chindesaurus)),(Buriolestes,((Eoraptor,((Saturnalia,Chromogisaurus),(Bagualosaurus,(Jaklapalisaurus,(Nambalia,((Thecodontosaurus,Pantydraco),(Efraasia,(Plateosauravus,(Ruehleia,(((Unaysaurus,(Pradhania,Macrocollum)),((Sarahsaurus,((Ngwevu,(Xixipiosaurus,((Coloradisaurus,(Glacialisaurus,Lufengosaurus)),(Massospondylus_carinatus,(Adeopapposaurus,Leyesaurus))))),(Yunnanosaurus_huangi,((Seitaad,(Anchisaurus,((Yizhousaurus,((Sefapanosaurus,Aardonyx),(NMQR1551,(NMRQ3314,(Blikanasaurus,((Camelotia,((Meroktenos,(Ledumahadi,Kholumolumo)),(Lessemsaurus,(Ingentia,Antetonitrus)))),(Pulanesaura,(Gongxianosaurus,(Schleitheimia,(Isanosaurus,((Tazoudasaurus,Vulcanodon),((Shunosaurus,(Spinophorosaurus,(Omeisaurus,(Mamenchisaurus,(Cetiosaurus,Neosauropoda))))),(Patagosaurus,Barapasaurus))))))))))))),(Irisosaurus,(Mussaurus,Leonerasaurus))))),(Xingxiulong,(Jingshanosaurus,Chuxiongosaurus)))))),(Eucnemesaurus_fortis,(Riojasaurus,Eucnemesaurus_entaxonis)))),(Plateosaurus_gracilis,(Plateosaurus_ingens,Plateosaurus_engelhardti)))))))))))),(Panphagia,Pampadromaeus))))),(Staurikosaurus,Herrerasaurus)))))));

(Euparkeria,(Crurotarsi,(Marasuchus,(Silesaurus,(Ornithischia,((Agnosphitys,((Guaibasaurus,(Neotheropoda,Chindesaurus)),(Buriolestes,((Eoraptor,((Saturnalia,Chromogisaurus),(Bagualosaurus,(Jaklapalisaurus,(Nambalia,((Thecodontosaurus,Pantydraco),(Efraasia,(Plateosauravus,(Ruehleia,(((Unaysaurus,(Pradhania,Macrocollum)),((Sarahsaurus,((Ngwevu,(Xixipiosaurus,((Coloradisaurus,(Glacialisaurus,Lufengosaurus)),(Massospondylus_carinatus,(Adeopapposaurus,Leyesaurus))))),(Yunnanosaurus_huangi,((Seitaad,(Anchisaurus,((Yizhousaurus,((Sefapanosaurus,Aardonyx),(NMQR1551,(NMRQ3314,(Blikanasaurus,((Camelotia,((Meroktenos,(Ledumahadi,Kholumolumo)),(Lessemsaurus,(Ingentia,Antetonitrus)))),(Pulanesaura,(Gongxianosaurus,(Schleitheimia,(Isanosaurus,((Tazoudasaurus,Vulcanodon),((Shunosaurus,((Spinophorosaurus,Omeisaurus),(Mamenchisaurus,(Cetiosaurus,Neosauropoda)))),(Patagosaurus,Barapasaurus))))))))))))),(Irisosaurus,(Mussaurus,Leonerasaurus))))),(Xingxiulong,(Jingshanosaurus,Chuxiongosaurus)))))),(Eucnemesaurus_fortis,(Riojasaurus,Eucnemesaurus_entaxonis)))),(Plateosaurus_gracilis,(Plateosaurus_ingens,Plateosaurus_engelhardti)))))))))))),(Panphagia,Pampadromaeus))))),(Staurikosaurus,Herrerasaurus)))))));

(Euparkeria,(Crurotarsi,(Marasuchus,(Silesaurus,(Ornithischia,((Agnosphitys,((Guaibasaurus,(Neotheropoda,Chindesaurus)),(Buriolestes,(Panphagia,(Eoraptor,((Saturnalia,Chromogisaurus),(Pampadromaeus,(Bagualosaurus,(Jaklapalisaurus,(Nambalia,((Thecodontosaurus,Pantydraco),(Efraasia,(Plateosauravus,(Ruehleia,(((Unaysaurus,(Pradhania,Macrocollum)),((Sarahsaurus,((Ngwevu,((Coloradisaurus,(Glacialisaurus,Lufengosaurus)),(Xixipiosaurus,(Massospondylus_carinatus,(Adeopapposaurus,Leyesaurus))))),(Yunnanosaurus_huangi,((Seitaad,(Anchisaurus,((Yizhousaurus,((Sefapanosaurus,Aardonyx),(NMQR1551,(NMRQ3314,(Blikanasaurus,((Camelotia,((Meroktenos,(Ledumahadi,Kholumolumo)),(Lessemsaurus,(Ingentia,Antetonitrus)))),(Pulanesaura,(Gongxianosaurus,(Schleitheimia,(Isanosaurus,((Tazoudasaurus,Vulcanodon),((Shunosaurus,(Spinophorosaurus,(Mamenchisaurus,(Omeisaurus,(Cetiosaurus,Neosauropoda))))),(Patagosaurus,Barapasaurus))))))))))))),(Irisosaurus,(Mussaurus,Leonerasaurus))))),(Xingxiulong,(Jingshanosaurus,Chuxiongosaurus)))))),(Eucnemesaurus_fortis,(Riojasaurus,Eucnemesaurus_entaxonis)))),(Plateosaurus_gracilis,(Plateosaurus_ingens,Plateosaurus_engelhardti))))))))))))))))),(Staurikosaurus,Herrerasaurus)))))));

(Euparkeria,(Crurotarsi,(Marasuchus,(Silesaurus,(Ornithischia,((Agnosphitys,((Guaibasaurus,(Neotheropoda,Chindesaurus)),(Buriolestes,(Panphagia,(Eoraptor,((Saturnalia,Chromogisaurus),(Pampadromaeus,(Bagualosaurus,(Jaklapalisaurus,(Nambalia,((Thecodontosaurus,Pantydraco),(Efraasia,(Plateosauravus,(Ruehleia,(((Unaysaurus,(Pradhania,Macrocollum)),((Sarahsaurus,((Ngwevu,(Xixipiosaurus,((Coloradisaurus,(Glacialisaurus,Lufengosaurus)),(Massospondylus_carinatus,(Adeopapposaurus,Leyesaurus))))),(Yunnanosaurus_huangi,((Seitaad,(Anchisaurus,((Yizhousaurus,((Sefapanosaurus,Aardonyx),(NMQR1551,(NMRQ3314,(Blikanasaurus,((Camelotia,((Meroktenos,(Ledumahadi,Kholumolumo)),(Lessemsaurus,(Ingentia,Antetonitrus)))),(Pulanesaura,(Gongxianosaurus,(Schleitheimia,(Isanosaurus,((Tazoudasaurus,Vulcanodon),((Shunosaurus,(Spinophorosaurus,(Omeisaurus,(Mamenchisaurus,(Cetiosaurus,Neosauropoda))))),(Patagosaurus,Barapasaurus))))))))))))),(Irisosaurus,(Mussaurus,Leonerasaurus))))),(Xingxiulong,(Jingshanosaurus,Chuxiongosaurus)))))),(Eucnemesaurus_fortis,(Riojasaurus,Eucnemesaurus_entaxonis)))),(Plateosaurus_gracilis,(Plateosaurus_ingens,Plateosaurus_engelhardti))))))))))))))))),(Staurikosaurus,Herrerasaurus)))))));

(Euparkeria,(Crurotarsi,(Marasuchus,(Silesaurus,(Ornithischia,((Agnosphitys,((Guaibasaurus,(Neotheropoda,Chindesaurus)),(Buriolestes,(Panphagia,(Eoraptor,((Saturnalia,Chromogisaurus),(Pampadromaeus,(Bagualosaurus,(Jaklapalisaurus,(Nambalia,((Thecodontosaurus,Pantydraco),(Efraasia,(Plateosauravus,(Ruehleia,(((Unaysaurus,(Pradhania,Macrocollum)),((Sarahsaurus,((Ngwevu,(Xixipiosaurus,((Coloradisaurus,(Glacialisaurus,Lufengosaurus)),(Massospondylus_carinatus,(Adeopapposaurus,Leyesaurus))))),(Yunnanosaurus_huangi,((Seitaad,(Anchisaurus,((Yizhousaurus,((Sefapanosaurus,Aardonyx),(NMQR1551,(NMRQ3314,(Blikanasaurus,((Camelotia,((Meroktenos,(Ledumahadi,Kholumolumo)),(Lessemsaurus,(Ingentia,Antetonitrus)))),(Pulanesaura,(Gongxianosaurus,(Schleitheimia,(Isanosaurus,((Tazoudasaurus,Vulcanodon),((Shunosaurus,((Spinophorosaurus,Omeisaurus),(Mamenchisaurus,(Cetiosaurus,Neosauropoda)))),(Patagosaurus,Barapasaurus))))))))))))),(Irisosaurus,(Mussaurus,Leonerasaurus))))),(Xingxiulong,(Jingshanosaurus,Chuxiongosaurus)))))),(Eucnemesaurus_fortis,(Riojasaurus,Eucnemesaurus_entaxonis)))),(Plateosaurus_gracilis,(Plateosaurus_ingens,Plateosaurus_engelhardti))))))))))))))))),(Staurikosaurus,Herrerasaurus)))))));

(Euparkeria,(Crurotarsi,(Marasuchus,(Silesaurus,(Ornithischia,((Agnosphitys,((Guaibasaurus,(Neotheropoda,Chindesaurus)),(Buriolestes,(Panphagia,(Eoraptor,((Pampadromaeus,(Saturnalia,Chromogisaurus)),(Bagualosaurus,(Jaklapalisaurus,(Nambalia,((Thecodontosaurus,Pantydraco),(Efraasia,(Plateosauravus,(Ruehleia,(((Unaysaurus,(Pradhania,Macrocollum)),((Sarahsaurus,((Ngwevu,((Coloradisaurus,(Glacialisaurus,Lufengosaurus)),(Xixipiosaurus,(Massospondylus_carinatus,(Adeopapposaurus,Leyesaurus))))),(Yunnanosaurus_huangi,((Seitaad,(Anchisaurus,((Yizhousaurus,((Sefapanosaurus,Aardonyx),(NMQR1551,(NMRQ3314,(Blikanasaurus,((Camelotia,((Meroktenos,(Ledumahadi,Kholumolumo)),(Lessemsaurus,(Ingentia,Antetonitrus)))),(Pulanesaura,(Gongxianosaurus,(Schleitheimia,(Isanosaurus,((Tazoudasaurus,Vulcanodon),((Shunosaurus,(Spinophorosaurus,(Mamenchisaurus,(Omeisaurus,(Cetiosaurus,Neosauropoda))))),(Patagosaurus,Barapasaurus))))))))))))),(Irisosaurus,(Mussaurus,Leonerasaurus))))),(Xingxiulong,(Jingshanosaurus,Chuxiongosaurus)))))),(Eucnemesaurus_fortis,(Riojasaurus,Eucnemesaurus_entaxonis)))),(Plateosaurus_gracilis,(Plateosaurus_ingens,Plateosaurus_engelhardti)))))))))))))))),(Staurikosaurus,Herrerasaurus)))))));

(Euparkeria,(Crurotarsi,(Marasuchus,(Silesaurus,(Ornithischia,((Agnosphitys,((Guaibasaurus,(Neotheropoda,Chindesaurus)),(Buriolestes,(Panphagia,(Eoraptor,((Pampadromaeus,(Saturnalia,Chromogisaurus)),(Bagualosaurus,(Jaklapalisaurus,(Nambalia,((Thecodontosaurus,Pantydraco),(Efraasia,(Plateosauravus,(Ruehleia,(((Unaysaurus,(Pradhania,Macrocollum)),((Sarahsaurus,((Ngwevu,(Xixipiosaurus,((Coloradisaurus,(Glacialisaurus,Lufengosaurus)),(Massospondylus_carinatus,(Adeopapposaurus,Leyesaurus))))),(Yunnanosaurus_huangi,((Seitaad,(Anchisaurus,((Yizhousaurus,((Sefapanosaurus,Aardonyx),(NMQR1551,(NMRQ3314,(Blikanasaurus,((Camelotia,((Meroktenos,(Ledumahadi,Kholumolumo)),(Lessemsaurus,(Ingentia,Antetonitrus)))),(Pulanesaura,(Gongxianosaurus,(Schleitheimia,(Isanosaurus,((Tazoudasaurus,Vulcanodon),((Shunosaurus,(Spinophorosaurus,(Omeisaurus,(Mamenchisaurus,(Cetiosaurus,Neosauropoda))))),(Patagosaurus,Barapasaurus))))))))))))),(Irisosaurus,(Mussaurus,Leonerasaurus))))),(Xingxiulong,(Jingshanosaurus,Chuxiongosaurus)))))),(Eucnemesaurus_fortis,(Riojasaurus,Eucnemesaurus_entaxonis)))),(Plateosaurus_gracilis,(Plateosaurus_ingens,Plateosaurus_engelhardti)))))))))))))))),(Staurikosaurus,Herrerasaurus)))))));

(Euparkeria,(Crurotarsi,(Marasuchus,(Silesaurus,(Ornithischia,((Agnosphitys,((Guaibasaurus,(Neotheropoda,Chindesaurus)),(Buriolestes,(Panphagia,(Eoraptor,((Pampadromaeus,(Saturnalia,Chromogisaurus)),(Bagualosaurus,(Jaklapalisaurus,(Nambalia,((Thecodontosaurus,Pantydraco),(Efraasia,(Plateosauravus,(Ruehleia,(((Unaysaurus,(Pradhania,Macrocollum)),((Sarahsaurus,((Ngwevu,(Xixipiosaurus,((Coloradisaurus,(Glacialisaurus,Lufengosaurus)),(Massospondylus_carinatus,(Adeopapposaurus,Leyesaurus))))),(Yunnanosaurus_huangi,((Seitaad,(Anchisaurus,((Yizhousaurus,((Sefapanosaurus,Aardonyx),(NMQR1551,(NMRQ3314,(Blikanasaurus,((Camelotia,((Meroktenos,(Ledumahadi,Kholumolumo)),(Lessemsaurus,(Ingentia,Antetonitrus)))),(Pulanesaura,(Gongxianosaurus,(Schleitheimia,(Isanosaurus,((Tazoudasaurus,Vulcanodon),((Shunosaurus,((Spinophorosaurus,Omeisaurus),(Mamenchisaurus,(Cetiosaurus,Neosauropoda)))),(Patagosaurus,Barapasaurus))))))))))))),(Irisosaurus,(Mussaurus,Leonerasaurus))))),(Xingxiulong,(Jingshanosaurus,Chuxiongosaurus)))))),(Eucnemesaurus_fortis,(Riojasaurus,Eucnemesaurus_entaxonis)))),(Plateosaurus_gracilis,(Plateosaurus_ingens,Plateosaurus_engelhardti)))))))))))))))),(Staurikosaurus,Herrerasaurus)))))));

(Euparkeria,(Crurotarsi,(Marasuchus,(Silesaurus,(Ornithischia,((Agnosphitys,((Guaibasaurus,(Neotheropoda,Chindesaurus)),(Buriolestes,(Pampadromaeus,(Panphagia,(Eoraptor,((Saturnalia,Chromogisaurus),(Bagualosaurus,(Jaklapalisaurus,(Nambalia,((Thecodontosaurus,Pantydraco),(Efraasia,(Plateosauravus,(Ruehleia,(((Unaysaurus,(Pradhania,Macrocollum)),((Sarahsaurus,((Ngwevu,((Coloradisaurus,(Glacialisaurus,Lufengosaurus)),(Xixipiosaurus,(Massospondylus_carinatus,(Adeopapposaurus,Leyesaurus))))),(Yunnanosaurus_huangi,((Seitaad,(Anchisaurus,((Yizhousaurus,((Sefapanosaurus,Aardonyx),(NMQR1551,(NMRQ3314,(Blikanasaurus,((Camelotia,((Meroktenos,(Ledumahadi,Kholumolumo)),(Lessemsaurus,(Ingentia,Antetonitrus)))),(Pulanesaura,(Gongxianosaurus,(Schleitheimia,(Isanosaurus,((Tazoudasaurus,Vulcanodon),((Shunosaurus,(Spinophorosaurus,(Omeisaurus,(Mamenchisaurus,(Cetiosaurus,Neosauropoda))))),(Patagosaurus,Barapasaurus))))))))))))),(Irisosaurus,(Mussaurus,Leonerasaurus))))),(Xingxiulong,(Jingshanosaurus,Chuxiongosaurus)))))),(Eucnemesaurus_fortis,(Riojasaurus,Eucnemesaurus_entaxonis)))),(Plateosaurus_gracilis,(Plateosaurus_ingens,Plateosaurus_engelhardti))))))))))))))))),(Staurikosaurus,Herrerasaurus)))))));

(Euparkeria,(Crurotarsi,(Marasuchus,(Silesaurus,(Ornithischia,((Agnosphitys,((Guaibasaurus,(Neotheropoda,Chindesaurus)),(Buriolestes,(Pampadromaeus,(Panphagia,(Eoraptor,((Saturnalia,Chromogisaurus),(Bagualosaurus,(Jaklapalisaurus,(Nambalia,((Thecodontosaurus,Pantydraco),(Efraasia,(Plateosauravus,(Ruehleia,(((Unaysaurus,(Pradhania,Macrocollum)),((Sarahsaurus,((Ngwevu,((Coloradisaurus,(Glacialisaurus,Lufengosaurus)),(Xixipiosaurus,(Massospondylus_carinatus,(Adeopapposaurus,Leyesaurus))))),(Yunnanosaurus_huangi,((Seitaad,(Anchisaurus,((Yizhousaurus,((Sefapanosaurus,Aardonyx),(NMQR1551,(NMRQ3314,(Blikanasaurus,((Camelotia,((Meroktenos,(Ledumahadi,Kholumolumo)),(Lessemsaurus,(Ingentia,Antetonitrus)))),(Pulanesaura,(Gongxianosaurus,(Schleitheimia,(Isanosaurus,((Tazoudasaurus,Vulcanodon),((Shunosaurus,((Spinophorosaurus,Omeisaurus),(Mamenchisaurus,(Cetiosaurus,Neosauropoda)))),(Patagosaurus,Barapasaurus))))))))))))),(Irisosaurus,(Mussaurus,Leonerasaurus))))),(Xingxiulong,(Jingshanosaurus,Chuxiongosaurus)))))),(Eucnemesaurus_fortis,(Riojasaurus,Eucnemesaurus_entaxonis)))),(Plateosaurus_gracilis,(Plateosaurus_ingens,Plateosaurus_engelhardti))))))))))))))))),(Staurikosaurus,Herrerasaurus)))))));

(Euparkeria,(Crurotarsi,(Marasuchus,(Silesaurus,(Ornithischia,((Agnosphitys,((Guaibasaurus,(Neotheropoda,Chindesaurus)),((Eoraptor,((Saturnalia,Chromogisaurus),(Bagualosaurus,(Jaklapalisaurus,(Nambalia,((Thecodontosaurus,Pantydraco),(Efraasia,(Plateosauravus,(Ruehleia,(((Unaysaurus,(Pradhania,Macrocollum)),((Sarahsaurus,((Ngwevu,((Coloradisaurus,(Glacialisaurus,Lufengosaurus)),(Xixipiosaurus,(Massospondylus_carinatus,(Adeopapposaurus,Leyesaurus))))),(Yunnanosaurus_huangi,((Seitaad,(Anchisaurus,((Yizhousaurus,((Sefapanosaurus,Aardonyx),(NMQR1551,(NMRQ3314,(Blikanasaurus,((Camelotia,((Meroktenos,(Ledumahadi,Kholumolumo)),(Lessemsaurus,(Ingentia,Antetonitrus)))),(Pulanesaura,(Gongxianosaurus,(Schleitheimia,(Isanosaurus,((Tazoudasaurus,Vulcanodon),((Shunosaurus,(Spinophorosaurus,(Mamenchisaurus,(Omeisaurus,(Cetiosaurus,Neosauropoda))))),(Patagosaurus,Barapasaurus))))))))))))),(Irisosaurus,(Mussaurus,Leonerasaurus))))),(Xingxiulong,(Jingshanosaurus,Chuxiongosaurus)))))),(Eucnemesaurus_fortis,(Riojasaurus,Eucnemesaurus_entaxonis)))),(Plateosaurus_gracilis,(Plateosaurus_ingens,Plateosaurus_engelhardti)))))))))))),(Buriolestes,(Panphagia,Pampadromaeus))))),(Staurikosaurus,Herrerasaurus)))))));
[truncated: 589,733 more chars]
